# Antibodies to a CA 19-9 Related Antigen Complex Identify SOX9 Expressing Progenitor Cells In Human Foetal Pancreas and Pancreatic Adenocarcinoma

**DOI:** 10.1038/s41598-019-38988-8

**Published:** 2019-02-27

**Authors:** Alison M. Farley, David R. Braxton, Jonathan Li, Karl Trounson, Subhanwita Sakar-Dey, Bhavana Nayer, Tatsuhiko Ikeda, Kevin X. Lau, Winita Hardikar, Kouichi Hasegawa, Martin F. Pera

**Affiliations:** 1 Department of Anatomy and Neuroscience, University of Melbourne, Melbourne, Victoria Australia; 2 Department of Pathology and Laboratory Medicine, Emory University School of Medicine, Atlanta, GA USA; 3 Institute for Stem Cell Biology and Regenerative Medicine, Bangalore, India; 4 Institute for Integrated Cell-Materials Science, Kyoto University, Kyoto, Japan; 5 Royal Childrens Hospital, Parkville, Victoria Australia; 6 Childrens Medical Research Institute, Parkville, Victoria Australia; 7 Florey Neuroscience and Mental Health Institute, Parkville, Victoria Australia; 8 The Walter and Eliza Hall Institute of Medical Research, Parkville, Victoria Australia

## Abstract

The Sialyl Lewis A antigen, or CA 19-9, is the prototype serum biomarker for adenocarcinoma of the pancreas. Despite extensive clinical study of CA 19-9 in gastrointestinal malignancies, surprisingly little is known concerning the specific cell types that express this marker during development, tissue regeneration and neoplasia. SOX9 is a transcription factor that plays a key role in these processes in foregut tissues. We report the biochemistry and tissue expression of the GCTM-5 antigen, a pancreatic cancer marker related to, but distinct from, CA19-9. This antigen, defined by two monoclonal antibodies recognising separate epitopes on a large glycoconjugate protein complex, is co-expressed with SOX9 by foregut ductal progenitors in the developing human liver and pancreas, and in pancreatic adenocarcinoma. These progenitors are distinct from cell populations identified by DCLK1, LGR5, or canonical markers of liver and pancreatic progenitor cells. Co-expression of this antigen complex and SOX9 also characterises the ductal metaplasia of submucosal glands that occurs during the development of Barrett’s oesophagus. The GCTM-5 antigen complex can be detected in the sera of patients with pancreatic adenocarcinoma. The GCTM-5 epitope shows a much more restricted pattern of expression in the normal adult pancreas relative to CA19-9. Our findings will aid in the identification, characterisation, and monitoring of ductal progenitor cells during development and progression of pancreatic adenocarcinoma in man.

## Introduction

The Sialyl Lewis A antigen CA 19-9 (review^1^) was one of the first cancer markers defined by a monoclonal antibody, and it remains the most widely used serum marker for pancreatic adenocarcinoma today. However, the shortcomings of CA 19-9 for screening applications or detection of early stage disease are widely recognised, and there is an ongoing effort to identify novel biomarkers that might enable better early diagnosis and monitoring of this devastating cancer. In recent years, proteomics analyses have revealed that many proteins are capable of carrying the CA 19-9 epitope^2,3^, and glycomics studies have shown that the specific variants of the Sialyl Lewis A antigen are recognised with varying affinities by different monoclonal antibodies^4^. Some studies have indicated that improved specificity and sensitivity for diagnostic and monitoring purposes can be achieved by combining the use of CA19-9 with the use of other markers^5,6^, such as MUC5AC^7^ or thrombospondin2^8^, or metabolomic profiles^9,10^, or through the application of multiple antibody panels directed against Sialyl Lewis A antigen^4^.

Despite extensive clinical study of the use of CA 19-9 as a serum cancer marker, and the increasing appreciation of the complexity of its biochemistry, there have been fewer investigations into the cell type specificity of expression of the CA 19-9 family of glycotopes during development, regeneration and neoplasia. In pancreatic adenocarcinoma, recent studies in experimental model systems have strongly implicated acinar to ductal metaplasia as a key step in cancer development (review^11,12^). However, the precise nature of the ductular cells that comprise this metaplastic response remains uncertain. Some investigators regard the ductular metaplastic cells in the pancreas as equivalent to the ducts of biliary epithelium^13^, whilst others regard these cells as equivalent to the early multipotent progenitors of all the pancreatic epithelial lineages (review^14^). Duct-like cell populations are implicated in development, repair and pathogenesis in multiple foregut lineages, and these populations often express the transcription factor SOX9^15,16^. The biliary reaction in liver is a proliferation of bile duct-like cells that occurs in response to multiple forms of liver damage in which hepatocyte proliferation is compromised^17^, and a large body of evidence supports the identification of liver progenitor cells as the cell of origin of cholangiocarcinoma and hepatocellular carcinoma^18^. In the pancreas, acinar to ductular metaplasia is now recognised as both a response to tissue damage and a precursor to neoplasia, and SOX9 plays a key role in this process^19^. And in Barrett’s oesophagus, several recent studies have recognised that ductal metaplasia of the submucosal glands is a common feature of damage arising from gastroesophageal reflux disease associated with this condition^20,21^, though the relationship between these ductular cells and the columnar epithelium characteristic of Barrett’ oesophagus is not clear at present.

Our understanding of the origin and fate of these ductular populations in human disease is hampered by the fact that they are almost certainly heterogeneous collections of cells with distinct developmental potentials, and by a lack of appropriate biomarkers to track their activity in tissue regeneration, metaplasia, and neoplasia. However, recent research has identified a number of candidate markers of progenitors in pancreatic cancer. These molecules include LGR5^22^ and DCLK1^23,24^, in addition to canonical epithelial stem cell markers like EPCAM, CD133, and NCAM, which mark bipotential foregut progenitor cells in a heterogeneous fashion^25^. In liver and pancreas, ductal progenitors that proliferate in response to damage express SOX9^19,26^, a marker of primitive embryonic precursors in both of these tissues^15,16^. More precise delineation of the key cellular intermediates in pancreatic cancer development and progression will undoubtedly lead to identification of better secreted biomarkers for early diagnosis and patient monitoring. In this context, a static picture may not be as informative as longitudinal analysis of the dynamics of expression of biomarkers for specific progenitor cell types. Recent longitudinal studies of CA 19-9 support the concept that longitudinal monitoring can detect disease at an earlier stage^27^.

We have previously described a monoclonal antibody reactive with a cell surface glyconjugate on human endodermal progenitor cells^28,29^. The antibody reacts with proliferating bile duct cells in the liver, with premalignant and malignant cells in the pancreas, and with Barrett’s metaplasia. Antibody positive cells from the non-parenchymal population of adult liver showed expression of mRNA encoding markers of primitive foregut endoderm including PDX1, HNF1 HNF4A and SOX17.

In this study, we assessed the potential for the use of this marker in noninvasive monitoring of ductal progenitor cell populations in metaplasia and neoplasia, focusing on adenocarcinoma of the pancreas. We carried out biochemical analysis to show the antigen was related to but distinct from Sialyl Lewis A, then studied its expression in normal foetal tissues and disease states, comparing it to SOX9 and other markers of foregut progenitor cells in liver and pancreas. We demonstrated that the antigen could be detected in the sera of patients with pancreatic adenocarcinoma.

## Results

### GCTM-5 antibody recognises a large secreted glycoprotein complex containing the CA 19-9 epitope

The GCTM-5 antigen is sialidase-sensitive, and its expression overlaps to some degree with that of CA 19-9^28,29^. In order to define the relationship between the GCTM-5 antigen and CA 19-9, we characterised its biochemistry further and developed a second-generation antibody against it.

We first examined GCTM-5 antigen expression on a panel of cell lines derived from the pancreatic adenocarcinoma. Five out of seven cell lines expressed the antigen on their surface (Fig. S1A) and secreted the antigen into the culture medium (below), but expression was heterogeneous within cell lines, as described previously for CFPAC-1. We cloned cell line SW1990 to obtain sublines that showed high levels of expression in ~95% of cells (Fig. S1B), and used culture supernatant from this cell line in affinity purification.

**Figure 1:**
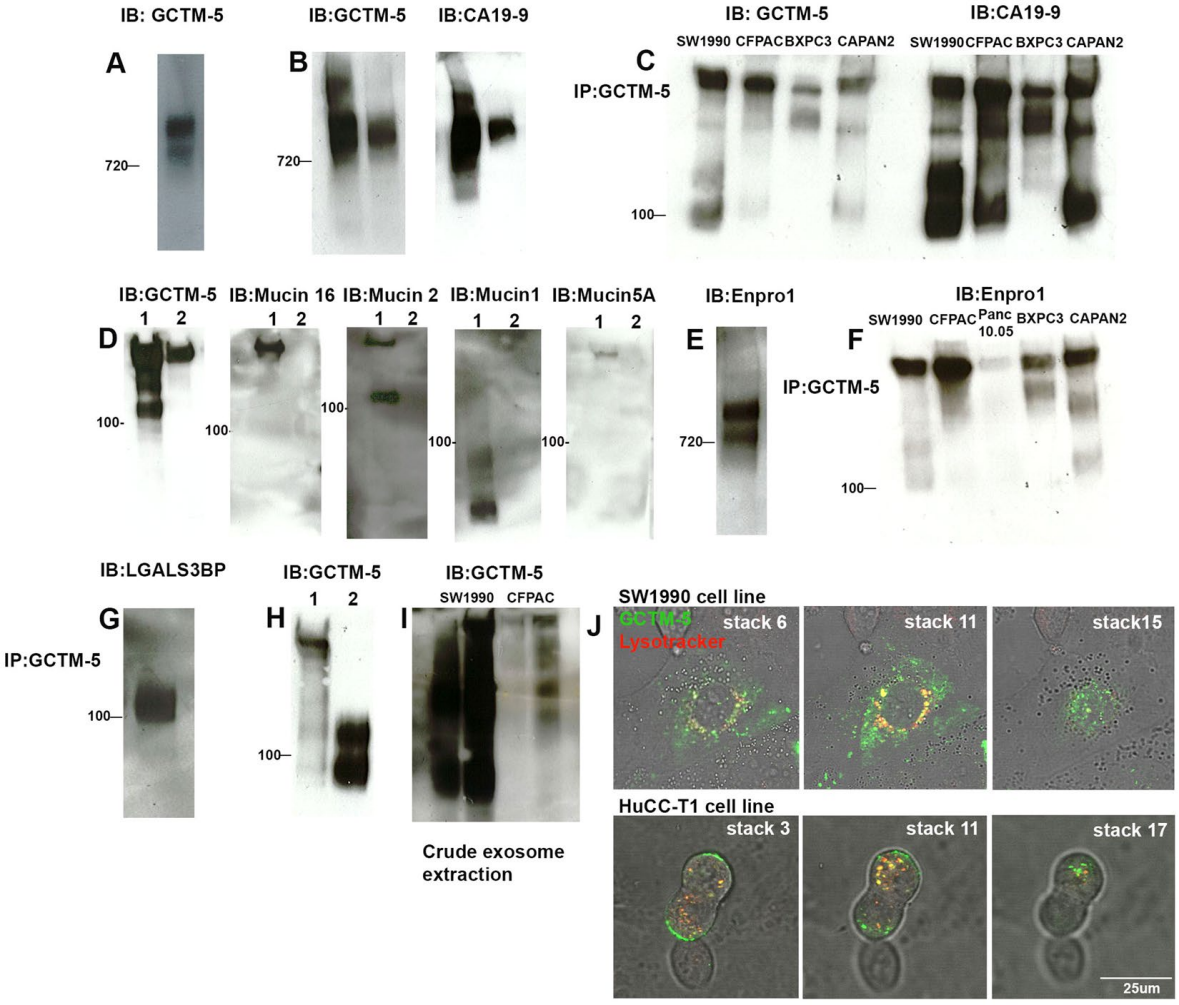
Biochemical analysis of GCTM-5 antigen complex. (**A**) Native PAGE immunoblot of SW1990 cell line supernatant with GCTM5. Position of 720 kDa marker is indicated. (**B**) Native PAGE immunoblots of SW1990 cell line supernatant (left hand track in each panel) and purified GCTM5 antigen complex (right hand track in each panel) probed with either anti-GCTM5 or CA19-9. Position of 720 kDa marker is indicated. (**C**) Immunoblot of immunoprecipitated GCTM5 antigen complex from SW1990, CFPAC-1, BXPC-3 and Capan-2 cell line supernatants separated on SDS-PAGE reducing gel, and probed with GCTM-5 or CA19-9. Position of 100 kDa marker is indicated. (**D**) Immunoblots of SW1990 cell line supernatant (lane 1) or purified GCTM-5 antigen complex (lane 2) separated on SDS-PAGE reducing gel and probed with GCTM-5, or antibodies to Mucin 16, Mucin 2, Mucin 1 and Mucin 5 A. Position of 100 kDa marker is indicated. (**E**) Native PAGE immunoblot of SW1990 cell line supernatant probed with ENPRO1. Position of 720 kDa marker is indicated. (**F**) Immunoblot of GCTM-5 immunoprecipitated antigen complexes from SW1990, CFPAC-1, Panc 10.05, BXPC-3 and Capan-2 cell line supernatants separated on SDS-PAGE reducing gels and probed with ENPRO1. Position of 100 kDa marker is indicated. (**G**) Immunoblot of immunoprecipitated SW1990 cell line GCTM-5 antigen complex separated on SDS-PAGE reducing gels and probed with anti- LGALS3BP. Positon of 100 KDa marker is indicated. (**H**) Immunoblot of SW1990 cell line supernatant (left hand track) and anti-LGALS3BP immunoprecipitate (right hand track) separated on SDS-PAGE reducing gel and probed with GCTM-5. Position of 100 kDa marker is indicated. (**I**) Immunoblot of SW1990 and CFPAC-1 cell line supernatants (left hand track) or extracellular vesicle preparations (right hand track) probed with GCTM-5. (**J**) Serial confocal microscope z-sections of live cell images of SW1990 or HuCC-TI cells incubated with Lysotracker dye and GCTM-5.

Conventional SDS-PAGE did not fully resolve the antigen complex obtained from the SW1990 clone, but only showed a large blur at the top of the gel in immunoblots. Therefore, we used non-denaturing native Coomassie PAGE gels to fractionate the antigen from cell culture supernatants and from affinity-purified preparations. The native complex migrated as two entities with a main band corresponding to an apparent molecular mass of around 800 kDa and a secondary band of 720 kDa (Fig. 1A).

We tested whether the GCTM-5 antigen and CA 19-9 could be distinguished biochemically. Using denaturing SDS-PAGE, we found that the purified GCTM-5 antigen complex from SW1990 cells reacted with the CA19-9 antibody (Fig. 1B), as did the antigen complex from three other pancreatic cancer cell lines (Fig. 1C). However, analysis of glycan binding activity of the GCTM-5 antibody, carried out by the Center for Functional Genomics Core H on Glycan Array 5.1, showed clearly that the reactivity of GCTM-5 was distinct from that of CA 19-9. Indeed, GCTM-5 failed to react above the background cut-off with Sialyl-Lewis A or any of the six hundred glycans present on the array. Interestingly however GCTM-5 displayed weak reactivity (above background but below the cut-off for a genuine positive reaction) with several Sialyl Lewis A related structures (Table S2). Double label indirect immunofluorescence assays of antigen expression on pancreatic adenocarcinoma cell lines provided further evidence for distinction between the CA 19-9 and GCTM-5 epitopes, since cells that were strongly positive for only one marker were readily identified (Fig. S1C). Moreover, we confirmed that GCTM-5, but not CA 19-9, reacted with normal colonic mucosa (Fig. S1D). Lastly, although the GCTM-5 antigen complex was reactive with CA19-9, it did not contain any of the known mucins commonly associated with Sialyl Lewis A, including MUC1, MUC2, MUC5A, and MUC16, though all of these mucins were readily detected in cell culture supernatants from these cell lines (Fig. 1D).

We detected an antigen complex of similar size to that found in pancreatic adenocarcinoma cell lines in the cell culture supernatant of the human cholangiocarcinoma cell line HuCCT1 (Fig. S1E). This finding suggests strongly that the antigens produced by biliary and pancreatic cells are similar.

### Characterisation of a second-generation antibody ENPRO1 confirms relationship of the GCTM-5 antigen complex and CA 19-9

To generate second generation antibodies, we immunised mice with a slurry of Protein G beads containing Protein G-Sepharose, GCTM-5 antibody and the bound antigen complex. Using this approach, we obtained one antibody of desired specificity, called ENPRO1 (IgG1). In immunofluorescence assays on pancreatic cancer cell lines, this antibody co-localised precisely with GCTM-5 (Fig. S2). ENPRO1 reacted with SW1990 cell culture supernatants separated on native Coomassie PAGE gels (Fig. 1E), and the GCTM-5 immunoprecipitates from all GCTM-5 positive pancreatic cancer cell lines (Fig. 1F). Glycan array analysis indicated that ENPRO-1, unlike GCTM-5, showed reactivity with the CA 19-9 related structures (Table S3). However, like GCTM-5, ENPRO1 showed a distinct specificity to CA19-9, since it too reacted with normal colonic mucosa, unlike CA 19-9 (Fig. S1D).

### The GCTM-5 antigen complex contains exosome-associated proteins and is shed into extracellular vesicles by cultured cell lines

Since the GCTM-5 antigen complex appeared to lack mucins associated with CA 19-9, we analysed its components further. Mass spectrometry (Table S4) confirmed that the antigen complex from CFPAC-1 contained FCGBP (Mascot score p < 0.05 on 4/4 independent analyses), as we previously reported, and further identified HPX as a component of the complex from this cell line (Mascot score p < 0.05 on 4/4 independent analyses). Analysis of the antigen complex from the SW1990 subclone did not detect FCGBP, but found LGALS3BP, a galectin 3 binding protein and a cancer marker widely expressed in a range of malignancies (Mascot score p < 0.05 in 3/3 independent runs), along with C3 (p < 0.05 in 2/3 analyses) and HPX (p < 0.05 in 1/3 analyses). LGALS3BP was also detected in CFPAC-1 immunoprecipitates (p < 0.05 in 1/4 analyses), and its presence in the antigen complex was confirmed by immunoblotting of GCTM-5 immunoprecipitates from SW1990 (Fig. 1G), and by GCTM-5 immunoblot of LGAS3BP pulldowns (Fig. 1H).

FCGBP, LGALS3BP, HPX, and C3 are all exosome components (www.exocarta.org). Their presence in the GCTM-5 antigen complex suggested that it was secreted into extracellular vesicles, and indeed the GCTM-5 antigen was found in 100,000 × g pellets obtained from conditioned medium from either CFPAC-1 or the SW1990 clone (Fig. 1I). In addition to staining of the cell surface, indirect immunofluorescence examination of cells pre-incubated with GCTM-5 and LysoTracker dye revealed co-localisation of the dye with the antibody in SW1990 cells or HuCCT1 cholangiocarcinoma cells, indicating uptake of the complex into the endocytic/lysosomal pathway (Fig. 1J).

### Cultured pancreatic adenocarcinoma cells lacking the GCTM-5 antigen have a tumour stromal phenotype

The cultured pancreatic adenocarcinoma cell lines that we studied were heterogeneous for GCTM-5 cell surface staining, with a substantial fraction of cells lacking antigen expression. To investigate this further, we separated antigen positive and negative cell populations of SW1990 and CFPAC-1 cell lines using fluorescence activated cell sorting, and carried out RNA-seq analysis of the two populations from each parent. 125 genes were upregulated in GCTM-5 positive cells from both cell lines and 259 genes were downregulated in positive cells from both cell lines (>2x change in expression and p < 0.01, Table S5). The positive cells had higher levels of transcripts for *FUT3* and *FUT6*. *FUT3* is involved in the biosynthesis of Sialyl Lewis A, and *FUT6* in biosynthesis of Sialyl Lewis X. GCTM-5 positive cells also showed higher expression of many epithelial related genes, including transcription factors (*GRHL1, GRHL2, GRHL3*), cytokeratins (*KRT6A, KRT6B, KRT81*, and *KRT86*), intercellular junction associated proteins (*DSG3, GJB2, JPH1, JPH2, PKP2, UPK2*) and genes related to keratinocyte differentiation (*IVL, EPPK*). By contrast, negative cells expressed higher levels of genes associated with epithelial to mesenchymal transitions, including transcription factors (*ZEB1, ZEB2, RUNX2* and *RUNX3*), extracellular matrix factors (*SPARC, GPC6, FBN1, VCAN, FLRT1, COL6A2, SPP1, FN1)*, and extracellular matrix remodelling molecules (*ADAMTS10, MMP7, MMP11, MMP13, MMP19, CTSF, MRC2, TNXB*), along with members of the insulin/IGF pathway and TGFβ pathways (*INS-IGF2, IGF2, INSR, TGFB2*). These data are consistent with the interpretation that the antigen negative cells have lost epithelial character and have upregulated genes associated with tumour stroma, and are in agreement with findings on expression of the GCTM-5 antigen in patient biopsies of pancreatic adenocarcinoma, where only epithelial cells were found to express the antigen (below).

### Foetal foregut progenitor cells express the GCTM-5 antigen

Previously we reported the expression of GCTM-5 in human embryonic hepatoblasts at 7wpc, in the foetal pancreas, and in the Canals of Hering in the adult liver. We performed more detailed assessment of the GCTM-5 antigen during foregut development, to understand how its expression related to expression of other foregut progenitor cell markers including SOX9. We used double label staining to compare expression ENPRO1 with other developmental markers of ductal cells (ENPRO1 provided more robust staining in formalin-fixed, paraffin-embedded specimens, but identical specificity was observed with GCTM-5).

**Figure 2:**
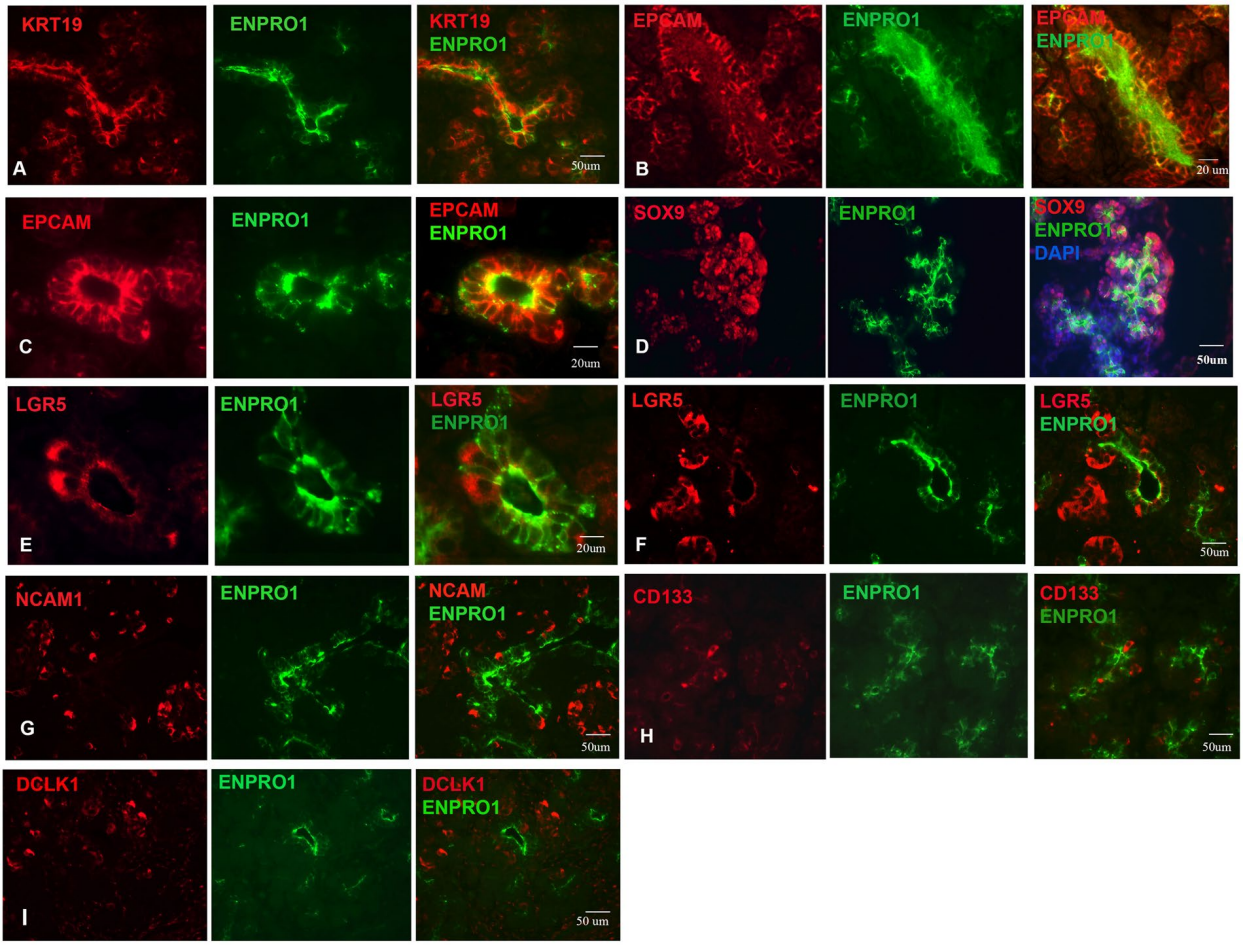
Double label indirect immunofluorescence micrographs showing staining for ENPRO1, the ductal cell marker KRT19, and progenitor cell markers EPCAM, SOX9, LGR5, SOX9, NCAM, CD133, and DCLK1 in the human fetal pancreas. (**A**) KRT19/ENPRO1; (**B**,**C**), EPCAM/ENPRO1; (**D**) SOX9/ENPRO1; (**E**,**F**) LGR5/ENPRO1; (**G**) NACM/ENPRO1; (**H**) CD133/ENPRO1; (**I**) DCLK1/ENPRO1. A, B, F = 16 week embryo; C, D, E = 13.5 week embryo; G, H, I = 15.5 week embryo.

In the foetal pancreas, ENPRO1 marked most of the bipotential progenitor population in the pancreatic epithelium, including all of the trunk and the more proximal but not distal ends of the tips of the budding pancreatic epithelial structures (Fig. 2). KRT19 was expressed in a broad population of epithelial cells (Fig. 2A) and all KRT19 expressing cells were ENRPO1 positive. Antibodies to EPCAM stained a majority of ENPRO-1 positive cells in the primitive epithelium (Fig. 2B) but also decorated cells budding off from the ducts (Fig. 2C). SOX9 was expressed in the nuclei of ENPRO1 positive cells, but also in a broader population of the developing epithelium, with staining extending out into the terminal portion of the tips (Fig. 2D). LGR5 expression was found in a minority subset of cells within both the trunks and tips of the epithelium, plus clusters of cells that have budded off from these structures (Fig. 2E,F). NCAM immunoreactivity was found in some cells budding off from the ENPRO1 positive epithelium, and cells at the periphery of islets, but not in the ductal epithelium itself (Fig. 2G). There was little staining of the ductal epithelium by CD133 antibody (Fig. 2H). DCLK1, which has been suggested as a marker for ductular cells during pancreatic cancer development, did not stain the epithelial branching structures, but instead decorated clusters of cells outside of them (Fig. 2I). The pancreatic mesenchyme, islets, and terminal tips were all negative for ENPRO1. The staining patterns that we observed for SOX9, NCAM, and EPCAM are broadly similar to previous results obtained on human pancreata at similar stages of development with these markers^30–38^. Our comparative study suggests that amongst the cell surface markers examined, the GCTM-5/ENPRO1 antigen is most specific for the multipotent epithelium of the ductal pancreatic anlage.

**Figure 3:**
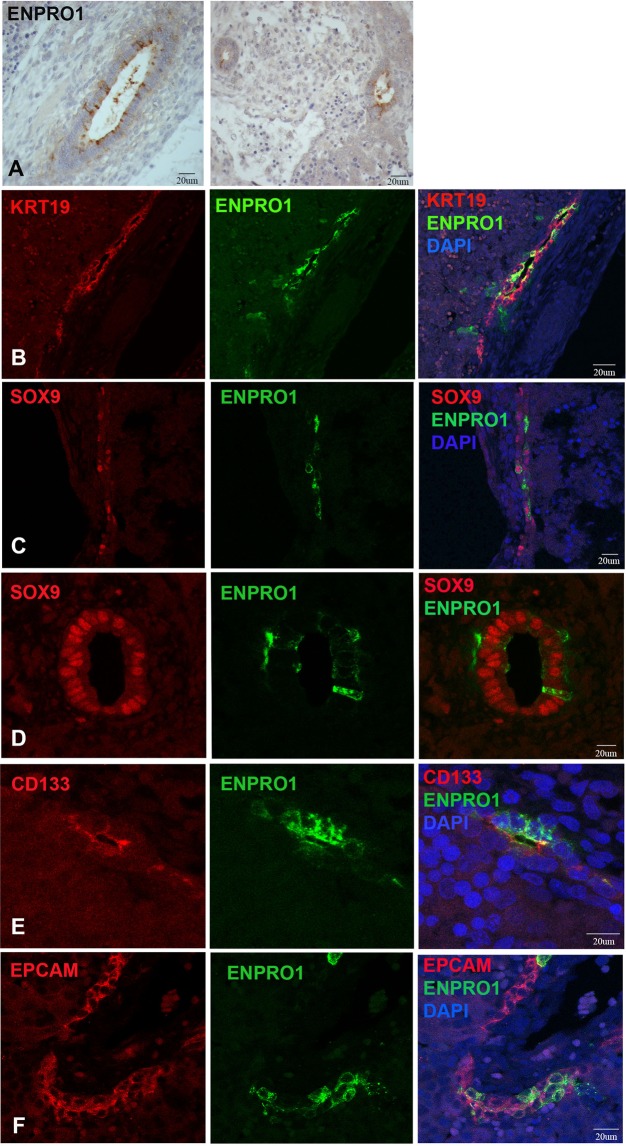
Double label indirect immunofluorescence micrographs showing staining for ENPRO1, KRT19 and progenitor cell markers SOX9, CD133, and EPCAM in the human fetal liver. (**A**) Expression of ENPRO1 by common bile duct and intrahepatic ductal progenitor cells of the Week 7–8 fetal liver; (**B**) week 15.0 fetal liver stained with KRT19 and ENPRO1; (**C**,**D**) week 15.0 fetal liver ductal plate stained with SOX9/ENPRO1; (**E**) 13. 5 week fetal liver with newly forming ductal structures stained with CD133/ENPRO1; (**F**) 13.5 week fetal liver ductal plate stained with EPCAM/ENPRO1.

We also examined expression of ENPRO1 in the human embryonic foetal liver through a broader developmental time series than that covered in our previous work. The extrahepatic bile duct and the common bile duct reacted with ENPRO1 at week 7 (Fig. 3A). Ductal plate hepatoblasts are bipotent, capable of differentiation into both bile ducts and periportal hepatocytes. ENPRO1 consistently stained cells of the ductal plate (Fig. 3B,C,E,F), though only a subset of cells in the structure were reactive with the antibody. All ductal plate cells were KRT19 positive (Fig. 3B). SOX9 was detected in the nuclei of cells of the ductal plate and developing ductules that branched off from it, and a subset of SOX9 positive cells co-stained with ENPRO1 (Fig. 3C,D). CD133 was absent from most of the ductal plate, but stained some cells in the lumen of newly developed ductules (Fig. 3E). EPCAM stained all the cells of the ductal plate (Fig. 3F); only a subset of these cells was ENPRO1 positive. These findings on KRT 19, SOX9, and EPCAM expression in the ductal plate are in agreement with previous studies (e.g.^38^). Thus, similar to the findings in the foetal pancreas, ENPRO1 marks a primitive ductal progenitor in liver.

### GCTM-5/ENPRO1 define a distinct SOX9 positive population of pancreatic adenocarcinoma cells

We reported previously that GCTM-5 was expressed in acinar to ductular metaplasia, pancreatic intraepithelial neoplasia, and pancreatic ductal adenocarcinoma. Using tissue microarrays, we carried out a more extensive analysis and showed that 100/116 pancreatic adenocarcinomas stained strongly for GCTM-5 at the cell surface. Most of the negative specimens were of the micropapillary histological subtype.

**Figure 4:**
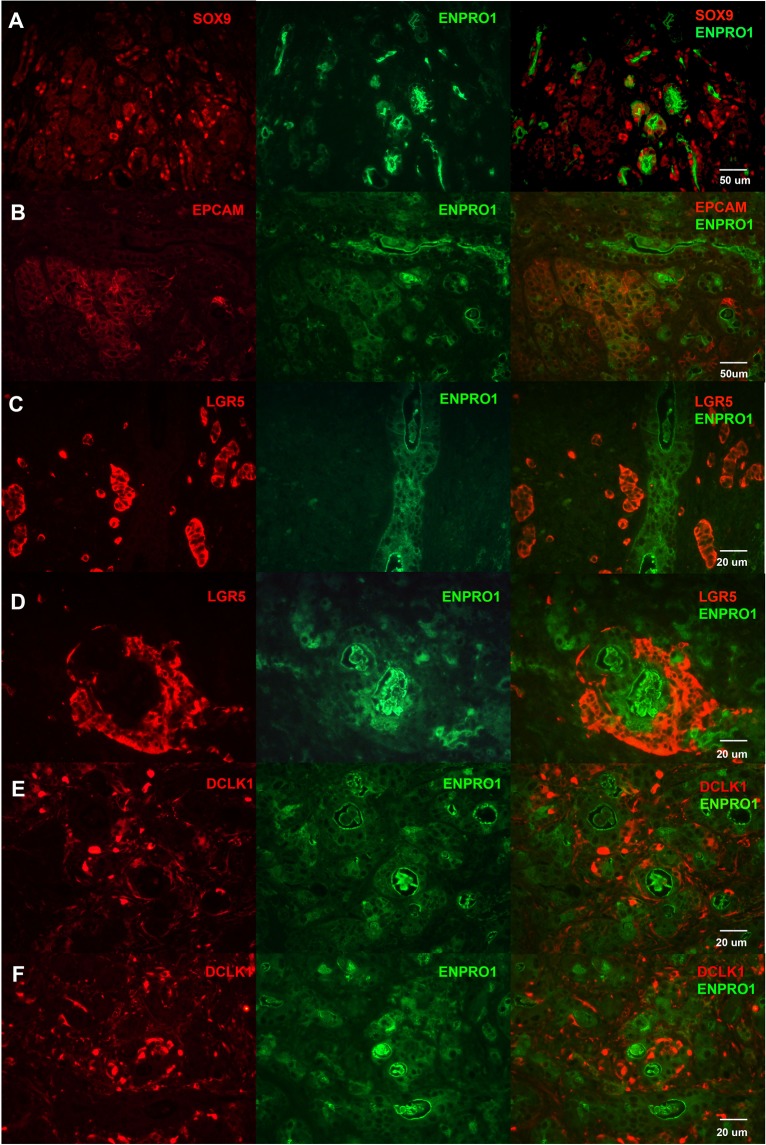
Double label indirect immunofluorescence micrographs showing staining for ENPRO1 and SOX9, EPCAM, LGR5, and DCLK1 in pancreatic ductal adenocarcinoma tissue. (**A**) SOX9/ENPRO1; (**B**) EPCAM/ENPRO1; (**C**,**D**) LGR5/ENPRO1; (**E**,**F**) DCLK1/ENPRO1.

We further examined co-staining of pancreatic carcinoma with GCTM-5 and ENPRO1 and other candidate markers of pancreatic cancer stem cells. The patterns of reactivity with these stem cell markers in pancreatic cancer were broadly reminiscent of those observed in the foetal pancreas. In tumour sections showing moderate degrees of differentiation, ENPRO1 or GCTM-5 stained cells lining duct-like structures and amorphous extracellular material contained within these structures (ENPRO1, Fig. 4A–F middle panels; GCTM-5, Supplementary Fig. S1F). ENPRO1 positive cells comprised a subset of SOX9 (Fig. 4A) and EPCAM (Fig. 4B) positive cells. LGR5 positive cells were excluded from glands stained by ENPRO1 (Fig. 4C,D) but were found adjacent to the glands, similar to the pattern we observed in the foetal pancreas. Likewise, DCLK1 immunoreactive cells were found mostly on the periphery of the ENPRO1 positive glands (Fig. 4E,F), as they were in normal foetal pancreas.

### GCTM-5 antigen expression is activated in SOX9 positive cells in acinar to ductal metaplasia in Barrett’s Oesophagus

**Figure 5:**
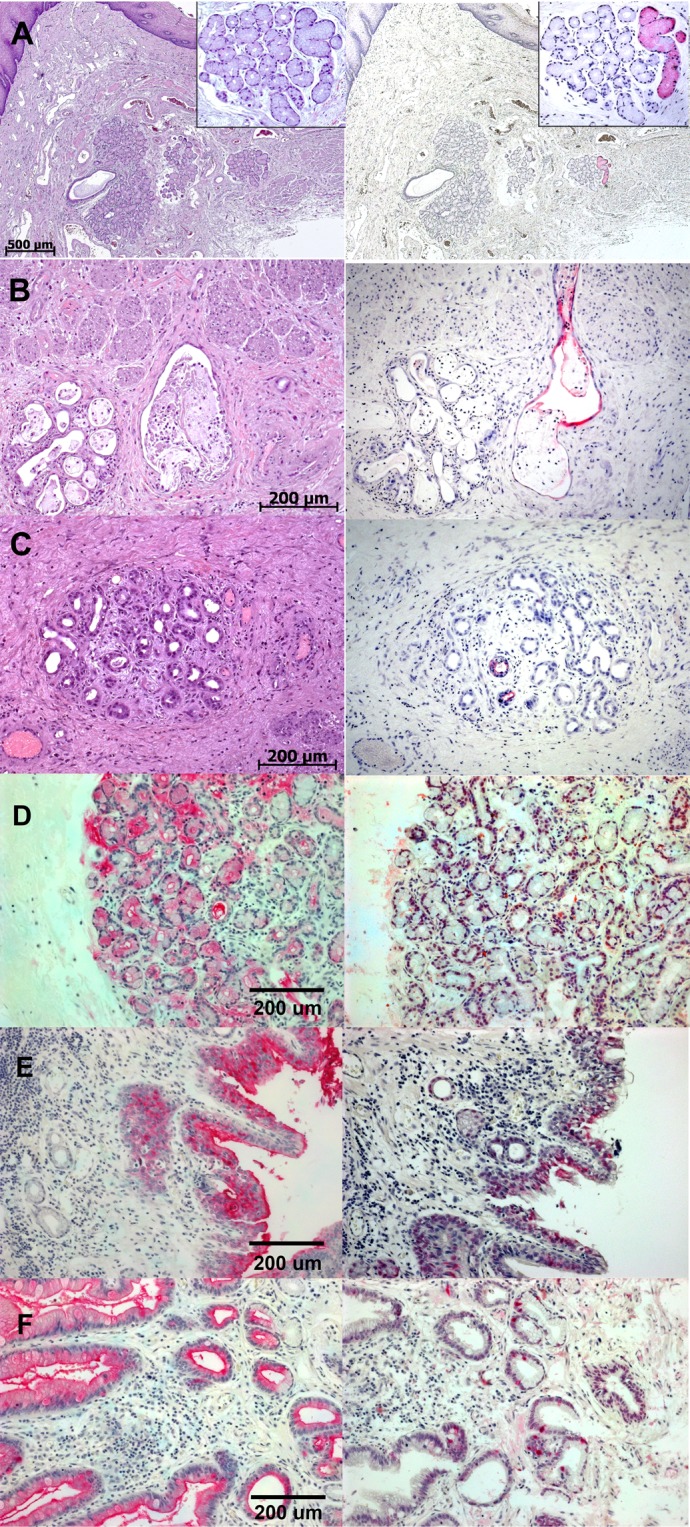
Immunohistochemistry showing expression of the GCTM-5 antigen and SOX9 in normal and abnormal human oesophagus. (**A**) Normal oesophagus with submucosal gland shown at higher magnification in the inset (left panel, hematoxylin and eosin; right panel, GCTM-5 visualised in a serial section with alkaline phosphatase/Fast red): (**B**) necrosis of submucosal gland and ductal metaplasia (left panel, hematoxylin and eosin; right panel, GCTM-5 visualised in a serial section with alkaline phosphatase/Fast red); (**C**) acinar to ductal metaplasia in submucosal gland (left panel, hematoxylin and eosin; right panel, GCTM-5 visualised in a serial section with alkaline phosphatase/Fast red); (**D**) acinar to ductal metaplasia (left panel, GCTM-5 visualised with alkaline phosphatase/Fast red; right panel, serial section with anti-SOX9 antibody visualised with alkaline phosphatase/Fast red); (**E**) multilayered epithelium (left panel, GCTM-5 visualised with alkaline phosphatase/Fast red; right panel, serial section with anti-SOX9 antibody visualised with alkaline phosphatase/Fast red); (**F**) Barrett’s glands ((left panel, GCTM-5 visualised with alkaline phosphatase/Fast red; right panel, serial section with anti-SOX9 antibody visualised with alkaline phosphatase/Fast red).

Recent studies have shown that Barrett’s glands differ in phenotype from normal small intestine^39^, and that the condition is often accompanied by an acinar to ductular metaplasia of the oesophageal submucosal glands, which have been implicated as a source of Barrett’s metaplasia^20,21^. In our previous work^29^, we demonstrated that although the GCTM-5 antigen was absent from normal oesophageal and small intestinal mucosa, it was found in intestinal and cardiac metaplasia, as well as in a putative precursor lesion to Barrett’s oesophagus, the multilayered epithelium. We re-examined expression of GCTM-5 along with SOX9 in normal submucosal glands and in Barrett’s tissue. There was faint, sporadic GCTM-5 staining in the cytoplasm of a subset of submucosal gland cells in normal oesophageal tissue (Fig. 5A). In gastrointestinal reflux disease, necrosis of submucosal glands (Fig. 5B) was associated with appearance of ductal epithelium, which was strongly GCTM-5 positive, displaying staining at the cell surface and in the ductal lumen. Ducts lined by cuboidal epithelium were also strongly positive on the lumenal surface for GCTM-5 (Fig. 5C), as were ductules and acini undergoing ductular transition (Fig. 5D), Areas with the strongest GCTM-5 reactivity (cell surface and lumenal) also showed a greater proportion of SOX9 positive cells compared to GCTM-5 low or negative areas (Fig. 5D). The multilayered epithelium, considered to be an intermediate in the development of Barrett’s glands, was strongly positive for SOX9 and the GCTM-5 antigen (Fig. 5E), which localised predominantly at the apical surface in this tissue. As reported previously, Barrett’s glands (Fig. 5F) were positive for GCTM-5 throughout their length. SOX9 positive cells were found mainly in the crypts and in the lower sections of the glands. Thus, the GCTM-5 antigen marks SOX9 positive cells including metaplastic ductal cells in Barrett’s metaplasia.

### The GCTM-5 antigen is detected in the sera of patients with pancreatic carcinoma

Because the GCTM-5 antigen was secreted or shed into the supernatant of pancreatic adenocarcinoma cell lines, and we observed positive immunoreactivity in biopsy specimens in the vast majority of cases, we decided to determine if we could detect the antigen in the serum of patients with pancreatic adenocarcinoma.

**Figure 6:**
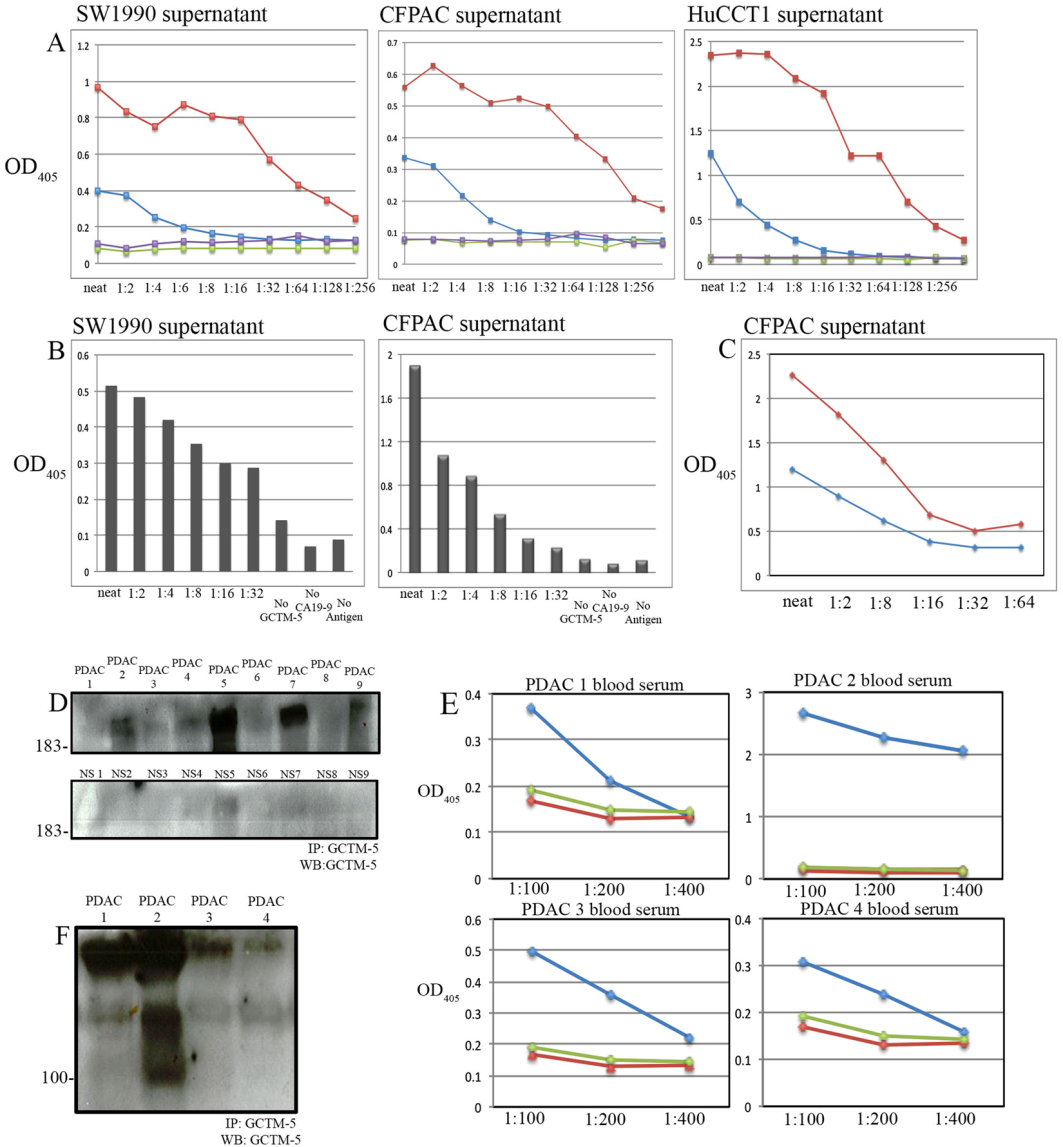
Detection of GCTM-5 antigen complex by ELISA in pancreatic ductal adenocarcinoma and cholangiocarcinoma cell line supernatants and by GCTM-5/CA 19-9 sandwich ELISA in patient serum samples. (**A**) GCTM-5 (blue) and ENPRO1(red) antibody capture assays in SW1990, CFPAC-1 and HuCC-T1 cell line supernatants. No primary antibody control (purple) and no antigen control (green) are shown. (**B**) Sandwich Elisa Assays with GCTM-5 used as the capture antibody and CA19-9 as the detection antibody on SW1990 and CFPAC-1 cell line supernatants. (**C**) Sandwich Elisa Assay comparing GCTM-5 (blue) and Enpro1(red) as the capture antibodies and CA19-9 as the detection antibody on CFPAC-1 cell line supernatant. (**D**) Immunoprecipitation followed by reducing SDS-PAGE and immunoblotting using GCTM-5 on pancreatic ductal adenocarcinoma patient serum samples and normal patient serum (NS). The position of the 183 kDa marker is indicated. Image of full length gel is shown in Fig. S3. (**E**) GCTM-5 and CA19-9 Sandwich Elisa assays on four pancreatic ductal adenocarcinoma patient serum samples (blue), and two control sera (green and red). (**F**) GCTM-5 immunoprecipitates separated by reducing SDS-PAGE then immunoblotted using GCTM-5 on patient blood serum samples 1–4 corresponding to ELISA analyses shown in (**E**).

The antigen was detected in cell culture supernatants from pancreatic adenocarcinoma and cholangiocarcinoma cell lines by antibody capture ELISA using GCTM-5 or ENPRO1, the latter showing enhanced sensitivity relative to the parent antibody (Fig. 6A). Since CA19-9 is carried on the GCTM-5 antigen but reacts with a distinct epitope, we were able to develop a sandwich antigen capture ELISA with the combination of the two antibodies (Fig. 6B); as in the antibody capture ELISA, the use of ENPRO1 with CA19-9 provided for greater sensitivity compared with the parent antibody (Fig. 6C). We used immunoprecipitation followed by immunoblotting to detect the antigen in 10/14 patient sera by immunoprecipitation and immunoblotting (Fig. 6D,F); one of nine control sera showed a band above background. Using a combination of GCTM-5 to capture the antigen and CA19-9 to detect it with better specificity in serum samples, we were able to measure levels of antigen in patient sera readily, typically down to titres below 1:200 (Fig. 6E). Control sera showed variable background reactivity that did not titrate down.

### The GCTM-5 antigen shows a restricted pattern of expression in the adult non-malignant pancreas compared to CA-19-9

**Figure 7:**
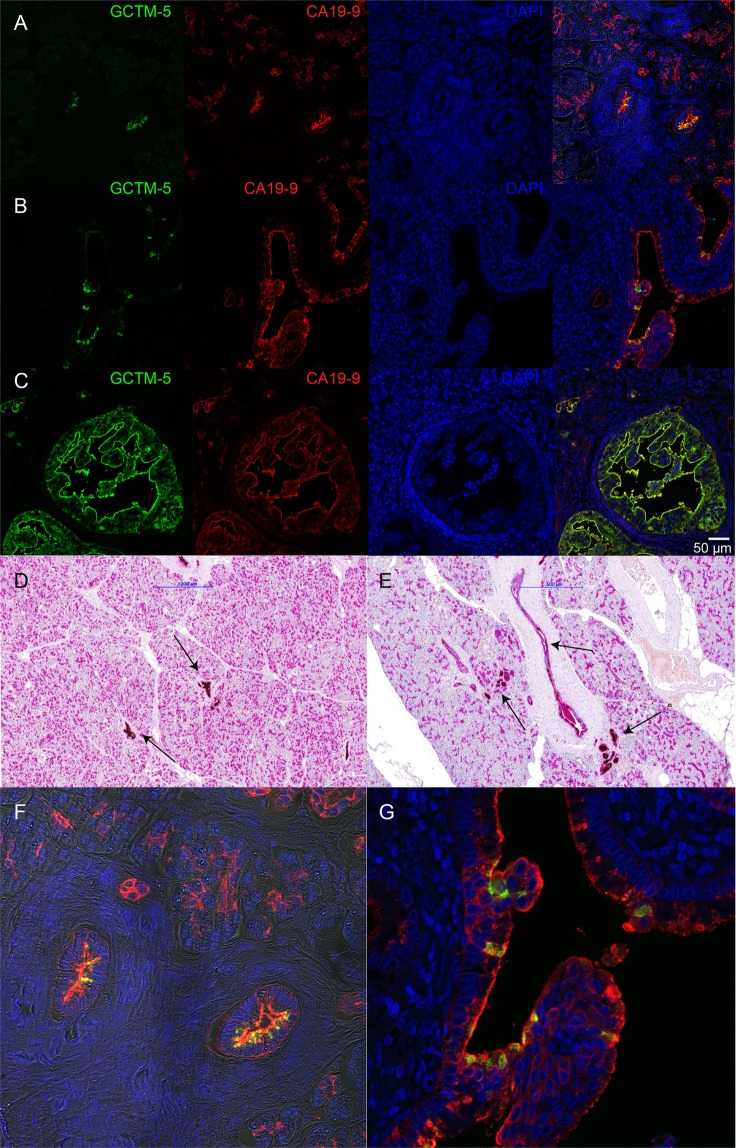
Double label immunofluorescence and immunohistochemical staining of adult pancreas with GCTM-5 and CA19-9. (**A**,**B**) Immunofluorescence staining of interlobular and intralobular ducts and ductules in non-malignant tissue with GCTM-5 and CA19-9. (**C**) Immunofluorescence staining of pancreatic intraepithelial neoplasia with GCTM-5 and CA19-9. In **(A**–**C)** GCTM-5 is green, CA19-9 is red, and DAPI nuclear counterstain is blue; scale bar in C = 50 µM indicates magnification in all panels in A-C. (**D**,**E**) Immunohistochemical staining for CA19-9 (magenta) and GCTM-5 (brown) in non-maligant tissue. Arrows indicate positive GCTM-5 staining in interlobular ducts and main pancreatic duct, and pancreatic ductular glands. (**F**,**G**) Show detail from double label stains in (**A**,**B**) respectively. Scale bar in B = 1000 µM; in C 500 µM.

The studies above indicate that while CA19-9 and GCTM-5 recognise distinct epitopes, both can be detected in sera from patients with pancreatic cancer. To investigate whether the GCTM-5 epitope might show greater specificity for malignant or premalignant pancreatic cells than CA19-9, we examined the expression of both markers in uninvolved pancreatic tissue from tumour biopsies in greater detail. As noted in previous studies^40–42^, CA19-9 is widely expressed at high levels in all ductal populations of non-malignant pancreatic tissue, including small ductules, interlobular ducts, and the main pancreatic duct (Fig. 7A,B,D–G). The majority of ductular cells in these structures expressed the CA19-9 antigen. In striking contrast, little or no staining with GCTM-5 was detected within the small ducts and intralobular ducts. Subsets of cells within the interlobular ducts reacted with GCTM-5, as did pancreatic ductular glands (Fig. 7A,B,D–G). As we previously observed^29^, pancreatic intraepithelial neoplasia stained strongly for GCTM-5, and these structures were also positive for CA19-9 (Fig. 7C). Thus, staining in non-malignant adult pancreatic tissue with GCTM-5 is restricted mainly to a subset of cells within interlobular ducts. Others have previously noted that the interlobular ducts contain subpopulations of cells that show antigen expression patterns consistent with their identification as progenitor cells^43,44^.

## Discussion

Damage to the liver, pancreas and oesophagus calls into play reparative mechanisms that recapitulate many aspects of developmental pathways. These reparative mechanisms often fail, or become subverted by the processes of metaplasia or neoplastic transformation. The precise nature of the cells that are involved in regeneration and metaplasia of human liver, pancreas and oesophagus remains uncertain, but a better understanding the dynamics of these cell populations would inform efforts to detect and monitor disease in these organs.

This study expands on our previous work characterising a marker of primitive foregut progenitor cells that is also is prominently expressed during repair and regeneration of liver, pancreas and oesophagus. SOX9 expression characterises ductular endodermal cell populations during development and tissue repair^15,16^. SOX9 is required for the development of the biliary system^45^, and the pancreas^46–48^, and for acinar to ductal metaplasia during pancreatic cancer development^19^. We show here that there is substantial overlap of the GCTM-5 antigen positive cells with SOX9 expression in developing liver and pancreas^49^. In the pancreas, multipotent precursor cells of the acinar, ductal and endocrine lineages are found in primitive ductal epithelial structures that arise from the pancreatic bud. These cells are SOX9 positive^36,37,49^. As the pancreatic lineages emerge, cells specified for the acinar lineage sprout off from the tips of the ducts, whereas cells destined to become endocrine and ductal cells bud off from the trunks. The expression of the GCTM-5/ENPRO1 antigen in the body of the primitive ducts is consistent with its expression on a multipotent progenitor cell. We found that by contrast, other markers of pancreatic progenitors, including LGR5, DCLK1, and NCAM, stain subsets of cells within the tips or trunks of the primitive epithelium, as well as clusters of cells outside the multipotent progenitor domain. Previous work has shown that NCAM and EPCAM marks precursors of the endocrine lineage as they branch off from the trunks^33,34^. These findings suggest that the GCTM-5/SOX9 positive population in the pancreas represents an early progenitor cell in the pancreatic lineage.

The GCTM-5 antigen is expressed in ductal progenitors in the liver. Here we showed that ENPRO1 marks foetal extrahepatic and common bile ducts, and progenitor cells in the ductal plate, the precursor of the biliary system including adult stem cells in the Canal of Hering, and periportal hepatocytes, which express SOX9 and some bile duct markers and have recently implicated in liver repair in chronic liver damage models^50^. The GCTM-5 antigen marks a subset of SOX9 positive cells in the ductal plate. Antibodies against the antigen complex will enable isolation and comparison of the properties of SOX9 positive ductal progenitor cells in normal and developing liver and pancreas with those in metaplastic and neoplastic conditions in oesophagus and pancreas.

Our studies show clearly that the SOX9 positive populations in foregut development and regeneration and pancreatic cancer are heterogeneous. Prospective isolation and molecular and biological characterisation of subpopulations of putative progenitor cells in pancreatic adenocarcinomas will be required to obtain a better understanding of the relationship between progenitor populations marked by GCTM-5, SOX9, LGR5, and DCLK1. In this context, GCTM-5 and ENPRO1 provide powerful tools for the identification and monitoring of progenitor cells during disease progression.

Our analyses of the expression of the GCTM-5/ENPRO1 antigen in the sera of patients with pancreatic adenocarcinoma are preliminary. Only a modest number of cases and controls were examined, and we have not assayed the sera of patients with related non-malignant conditions such as chronic pancreatitis. Large scale studies are required to determine whether measurement of the GCTM-5 antigen in patient sera (alone or in combination with other biomarkers), or isolation of exosomes or circulating tumour cells using GCTM-5 or ENPRO1, can provide useful diagnostic or prognostic information for patients with pancreatic adenocarcinoma. Our glycomics analysis, along with our studies of antigen expression in the adult pancreas, show that GCTM-5 recognizes a distinct epitope with a more restricted pattern of expression in the adult pancreas compared to that of CA19-9. These findings argue for more extensive evaluation of the potential of GCTM-5 as a biomarker in the diagnosis and therapy of pancreatic adenocarcinoma. The GCTM-5 antigen complex might be suitable for antibody targeted therapy, since the antigen is exposed only on the lumenal surfaces of small subpopulations of cells in normal epithelia; because the antigen/antibody complexes are internalised, they have the potential to deliver cytotoxic conjugates to the cell. The longitudinal, non-invasive use of secreted markers of specific stem or progenitor cell populations such as the GCTM-5 antigen could provide important information about the biology of progression of pancreatic cancer in man and the relationship of the cancer cells to foregut endoderm progenitors.

## Methods

### Cancer Cell Lines

Pancreatic ductal adenocarcinoma cell lines CFPAC-1, SW1990, Panc10.05, BXPC-3, Capan-2, AsPC-1 and HPAF-II were obtained from ATCC, USA, and liver cholangiocarcinoma cell line HuCCT1^51^, was obtained from the Riken Bioresource Center, Ibaraki, JP. Cell lines were authenticated by the providers using short tandem repeat profiling, and were used within six months of removal from stocks cryopreserved following two passages in our laboratory. Cells were cultured using standard methodologies (Supplementary Materials and Methods).

To separate GCTM-5 positive and negative cell populations, cell lines were sorted in a fluorescence activated cell sorter (BD FacsAria III, Becton Dickinson) following incubation with primary antibody GCTM-5 and Alexa 488 secondary antibody (ThermoFisher Scientific) and DAPI nuclear staining. GCTM5 positive single cells were sorted into 96-well plates, and following microscopic confirmation of single cell transfer, were grown out to yield clonal cell lines.

### Protein Purification, Immunoprecipitation and Mass Spectometry

Pancreatic ductal adenocarcinoma cell lines CFPAC-1, SW1990, Panc10.05, BXPC-3, Capan-2, AsPC-1 and HPAF-II were grown to 70% confluency then cultured for 3 days in serum-free medium. The medium was collected, spun down at 300xg and cellular debris was removed using a 0.22 µm filter. For large volume protein purification of the GCTM-5 complex, a Vivaflow 200 system VFS202 (Sartorius AG) with a 100,000 molecular weight cut-off PES filter was used to concentrate large proteins and protein complexes within the supernatant.

For large scale protein purification, 2 ml of IgG Protein Sepharose beads (Sigma-Aldrich) coupled with 4 mg/ml of purified GCTM-5 antibody were incubated overnight at 4 degrees with 40 ml of concentrated SW1990 cell line supernatant. The beads were washed 4–5 times in 100 mM Tris-buffered saline (pH 8) and incubated in 0.2 M glycine pH 11.5 for 10 minutes with shaking. This was repeated 4 times. 0.2 M glycine pH 2.5 was used to neutralize the protein elutions.

Immunoprecipitations were performed using GCTM-5 coupled IgG Sepharose beads in protein pull downs as above. Protein complexes bound to the beads were either eluted using 0.2 M glycine pH11.5 buffer, or the beads and protein were boiled in Laemmli sample buffer, then run on 4–12% reducing sodium dodecyl sulphate polyacrylamide gel electrophoresis (SDS-PAGE). Proteins were transferred to a polyvinylidene difluoride (PVDF) membrane and stained with primary antibodies (Table S1).

To separate the purified native GCTM-5 protein complex, a NativePAGE Bis-Tris Gel system (ThermoFisher Scientific) was used according to manufacturer’s protocol. Proteins were transferred to PVDF membrane and stained for either GCTM-5 or ENPRO1.

Mass spectrometry was performed using a Sciex 5600 Triple TOF system on tryptic digests of antigen complexes purified from the CFPAC-1 or SW1990 cloned cell lines. Mascot software was used to interpret mass spectral data.

Glycan array screening of purified GCTM-5 and ENPRO1 (final concentrations, 50 μg/ml) was performed by the Center for Functional Glycomics Core H of the Protein-Glycan Interaction Resource (supporting grant R24 GM098791) and the National Center for Functional Glycomics (NCFG) at Beth Israel Deaconess Medical Center, Harvard Medical School (supporting grant P41 GM103694).

### Preparation of a Second-Generation Antibody Against the GCTM-5 Antigen Complex

Immunisations and hybridoma production were performed by Paratopes Ltd. (London, UK.) using proprietary modifications of conventional methodology (Supplementary Materials and Methods).

### Extracellular Vesicle Preparation

Supernatant from serum-free SWI990 or CFPAC-1 cell cultures was spun down at 20,000 × g for 20 minutes in an ultracentrifuge (ThermoFisher Scientific WX ultra Series). The 20,000 × g supernatant was then transferred to a new 15 ml conical tube and spun at 100,000 × g for 70 minutes. The 100,000 × g supernatant was removed and the pellet was taken up in 30 μl of RIPA extraction buffer and 10 μl of loading dye. Samples were run on 4–12% reducing SDS-PAGE, transferred to a PVDF membrane, and blotted with GCTM-5 antibody. A Western Breeze chemiluminescent kit (ThermoFisher Scientific) was used to detect proteins.

### RNA Sequencing and Gene Expression Analysis

CFPAC-1 and SW-1990 cells were sorted into GCTM-5 positive and negative subpopulations as described above. A total of 12 samples from triplicate sorts, including three GCTM-5 positive and negative CFPAC-1 and SW1990 cell samples, were subjected to total RNA extraction (Supplementary Materials and Methods). poly A^+^ mRNA purification and cDNA library construction were performed using a TruSeq RNA sample preparation kit (Illumina Inc.) according to manufacturer’s instructions. The libraries were sequenced in paired end 100 base runs on the HiSeq. 1000 platform (Illumina Inc). Analysis and alignment of sequencing reads, and bioinformatics analyses, were carried out using standard software packages (Supplementary Materials and Methods).

### Human Tissue and Serum Samples

First and second trimester human foetal tissues were obtained for this study from Novogenix, USA. Additional archival first and second trimester tissues were originally obtained with patient informed consent through Monash Medical Centre, Clayton, Victoria, Australia with review by the Human Ethics Review Committee of Monash Health. Sera from patients with pancreatic ductal adenocarcinoma and from normal control subjects, as well as carcinoma tissue sections, were obtained from the Victorian Cancer Biobank. Ethical approval for use of all human foetal and tumour tissue in these studies was granted by the University of Melbourne (Biomedical Sciences Human Ethics Advisory Group); all experimental procedures were conducted according to University of Melbourne guidelines and in accordance with all applicable regulations and laws. For tissue microarray and oesophagus specimens, formalin fixed, paraffin embedded tissue blocks were obtained from Emory University Hospital Department of Pathology archives after local Institutional Review Board approval (Emory Institutional Review Board).

### Immunohistochemistry

Immunohistochemistry was performed on foetal liver and pancreas and pancreatic adenocarcinoma as previously described^28^ or with the addition of antigen retrieval on paraffin sections (Supplementary Materials and Methods). For sources of primary antibodies used, see Table S1. Application of secondary antibodies alone provided negative controls in all experiments. DAPI was used as a nuclear counterstain. Images were captured on a Zeiss Axioplan 2 or on a Leica SP8 confocal microscope.

In tissue microarray studies, GCTM-5 was tested on a range of different tumour types including pancreatic adenocarcinoma. Immunohistochemistry for GCTM-5 was performed with purified GCTM-5 monoclonal antibody (concentration 2.9 mg/ml) at a dilution 1:10,000 (vol/vol). The Bond Polymer Refine Detection Kit (using a DAB chromogen; Leica Microsystems, Bannock-burn, IL) was used as a secondary visualization system on the Leica Bond Maxx III automated system, and sections were counterstained with hematoxylin.

For double label staining of GCTM-5 and CA19-9 by immunohistochemistry, slides were dewaxed and retrieval of antigens were performed using Envision FLex Target Retrieval Solution (DM848). Primary antibodies CA19-9 (diluted at 1:250) and GCTM-5 Supernatant (neat) were applied sequentially. Antibodies were detected with EnVision Flex/HRP (Dako Omnis DM842) and Envision Flex DAB and Magenta chromogen (Dako Omnis DM847).

For studies of antigen internalisation, SW1190 and HuCCT1 cells were plated in 96 well plates designed for imaging (ThermoFisher Scientific, Nunc brand) and grown overnight to 50% confluency. ENPRO1 antibody and secondary antibodies were added to cells and incubated overnight, aspirated off, and cells were washed twice with PBS. Lysotracker dye (ThermoFisher Scientific) was added to cells, which were incubated for 30 minutes and then washed twice and imaged on a Leica SP8 confocal Microscope.

### ELISA

For antibody capture ELISA, we prepared antigen coated plates by incubation with SW1990 conditioned supernatant in serial dilutions with sodium bicarbonate (pH6) coating buffer. Capture of GCTM-5 or ENPRO1 antibody and subsequent detection was performed using standard methodology.

For sandwich antigen capture ELISA assays of cell culture supernatant or patient sera, we employed a combination of GCTM-5 and CA 19-9, using standard methodologies (Supplementary Materials and Methods).

## Supplementary information

Supplementary Information

## Data Availability

RNA-Seq data were deposited in GEO (Accession Number GSE95176).
