# Supplementary material for: Antibodies to a CA 19-9 Related Antigen Complex Identify SOX9 Expressing Progenitor Cells In Human Foetal Pancreas and Pancreatic Adenocarcinoma
Source: Sci Rep. 2019 Feb 27;9:2876. doi: 10.1038/s41598-019-38988-8 (PMC6393509; doi:10.1038/s41598-019-38988-8)
Supplement: Supplementary file 1 — Supplementary Information [file 41598_2019_38988_MOESM1_ESM.pdf]

Antibodies to a CA 19-9 Related Antigen Complex Identify SOX9 Expressing Cells In Human Foetal Pancreas and Pancreatic Adenocarcinoma. Alison M. Farley, David R. Braxton, Jonathan Li, Karl Trounson, Subhanwita Sakar-Dey, Bhavana Nayer, Tatsuhiko Ikeda, Kevin X. Lau, Winita Hardikar, Kouichi Hasegawa, and Martin F. Pera

Supplementary Materials and Methods

Cancer Cell Lines

Cell cultures were grown and maintained in RPMI 1640 (ThermoFisher Scientific, Life Technologies), containing 10% foetal bovine serum (Scientifix Life), and supplemented with 200mM GlutaMAX, 1/100 Insulin-Transferrin-Selenium, and 1/100 Penicillin-Streptomycin 10,00U/ml (all from Life Technologies). For serum-free culture, we used the same medium without foetal bovine serum.

Preparation of a Second-Generation Antibody Against the GCTM-5 Antigen Complex

GCTM-5 antigen complex from SW1190 clonal cell line was purified as described in the main text, and mice were inoculated with GCTM-5 antigen complex bound to IgG Sepharose beads. Serum samples from immunised mice were tested by antibody capture ELISA assay for reactivity against the complex. Following booster immunisations, fusion was carried out and hybridomas found positive by ELISA were cloned. The hybridomas were isotyped using a commercially available kit (Pierce Rapid Isotyping Kit, ThermoFisher Scientific).

RNA sequencing and gene expression analysis

Total RNA extractions were performed using an RNeasy Mini Kit (Qiagen) with DNase I (Qiagen). The quantity and quality of RNAs were examined with Qubit Fluorometer (Thermo Fisher Scientific) and Bioanalyzer (Agilent).

Library construction and RNA sequencing were performed by C-CAMP (India) and Agrigenome (India).

The raw RNAseq reads were analyzed by Bioconductor (Bioconductor.org). The reads were aligned to hg19/GRCh37 (NCBI), and quantified and normalized by library size and gene length by QuasR. Principal component analysis (PCA) plots of all samples were generated using the Prcomp function of the R stats. Differentially expressed genes (DEGs) were detected by TCC, and separated into up-regulated genes and down-regulated genes. The gene ontology analysis (GO) of biological processes was performed by goProfiles. The R package Pathview was used for pathway analyses. The gene sets of signal transduction and metabolism were extracted from the KEGG Pathway database (www.genome.jp/kegg/pathway). The $\log_2$ fold expression changes of DEGs were mapped to the pathway maps.

Immunohistochemistry

For antigen retrieval, sections were microwaved in 10mM Tri-sodium citrate buffer containing 0.1% Tween 20 pH 6 until boiling, then incubated for 20 minutes at 80 degrees, then left at room temperature for 20 minutes before adding 100mM Tris-buffered saline pH

7.4, 5% Goat Serum, and 2% Bovine Serum Albumin blocking buffer and application of primary antibodies.

ELISA Assays

Antibody capture ELISA was performed as follows. Following addition of antigen, 96 well plates were left overnight at 4 degrees. Subsequently the plates were washed 3x with 100mM Tris-buffered saline (pH 8), followed by addition of 200ul of blocking buffer (5% powdered skim milk in 100mM Tris-buffered saline with 0 .1% Tween 20) for two hours. Following 3 washes with TBS (pH 8), 200 µl of primary antibody was added in the form of either neat hybridoma supernatant or 10 µg/ml purified GCTM-5 or ENPRO1 and incubated for two hours, followed by four washes with TBS (pH 8) and addition of 200ul of 1/2000 goat anti-mouse Alkaline phosphatase secondary (Abcam Corporation) in blocking buffer. After a one hour incubation and four washes in TBS (pH 8), 200ul of developing solution (SigmaFAST containing p-Nitrophenyl phosphate (Sigma-Aldrich Co.)), was added for 15-30 minute after which the optical density at 405nm was measured in a Polarstar Omega Plate reader. Antigen capture sandwich ELISA was performed as follows. 100-200 mg/ml GCTM-5 in sodium bicarbonate pH 9.6 coating buffer was bound to plates for 6 hours which were then washed as described above for antibody capture assays. Plates were blocked for 1 hour or overnight at 4 degrees and washed as above. Pancreatic ductal adenocarcinoma cell line supernatants or serum specimens (1:25 vol/vol dilution) were plated and serially diluted across wells and incubated for 2 hours. Again, plates were washed as above and CA19-9 (1/250) (clone 121SLE; Cat. No. NBP2-15182[29]) was added for 1.5 hours. After washing the secondary antibody (Goat anti-mouse IgM- Alkaline Phosphatase (Sigma-Aldrich Com)) was added and detection was performed as above.

Antibodies to a CA 19-9 Related Antigen Complex Identify a Subset of SOX9 Expressing Cells In Human Foetal Pancreas and Pancreatic Adenocarcinoma. Alison M. Farley, David R. Braxton, Jonathan Li, Karl Trounson, Subhanwita Sakar-Dey, Bhavana Nayer, Tatsuhiko Ikeda, Kevin X. Lau, Winita Hardikar, Kouichi Hasegawa, and Martin F. Pera

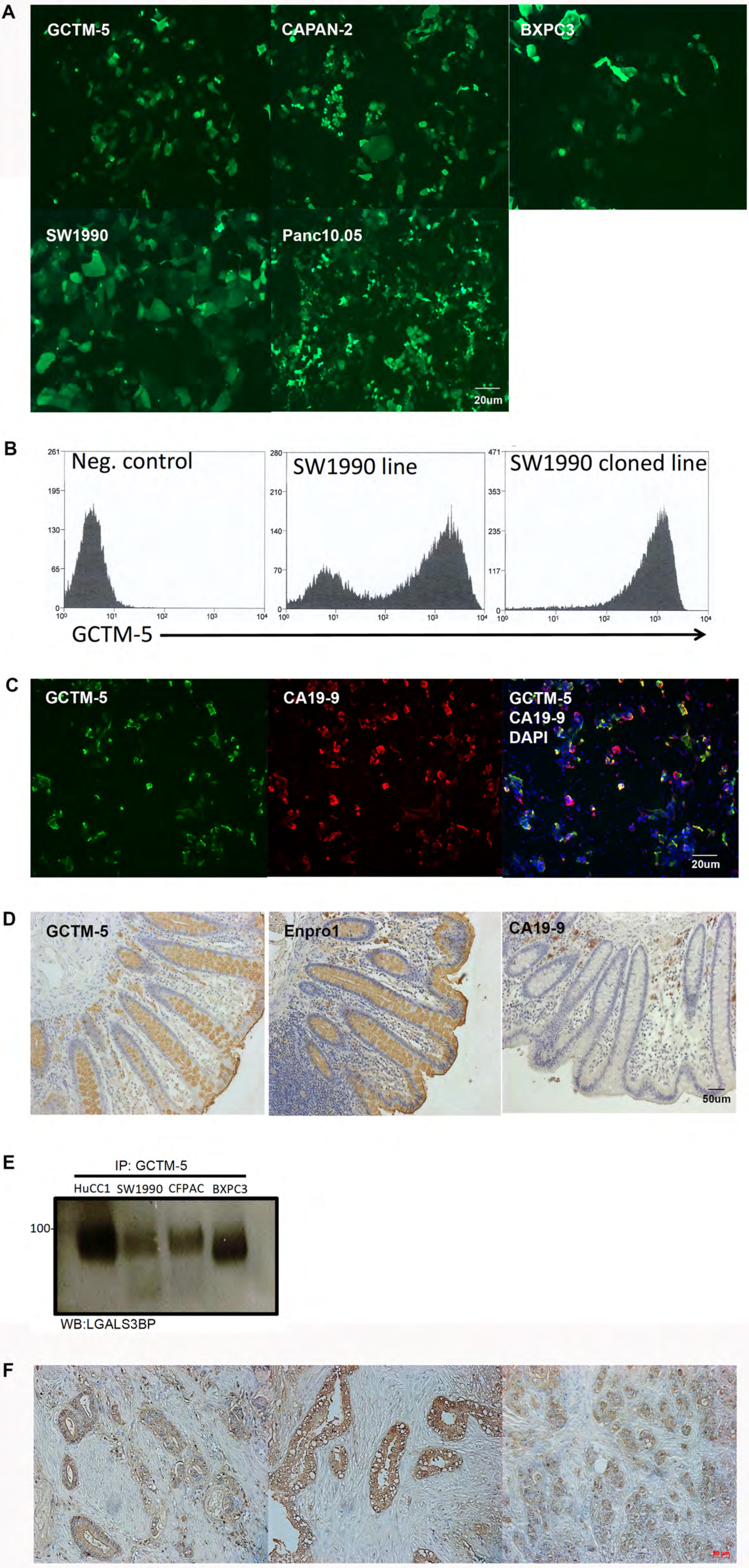

Supplementary Figure S1. Expression of GCTM-5 antigen complex in cultured cell lines and tissues. A, indirect immunofluorescence micrographs of cultured pancreatic adenocarcinoma cells lines stained with GCTM5. Cell lines AsPC-1 and HPAF-II were negative in this assay. B, flow cytometry profiles of SW1990 parent cell line and cloned GCTM-5 positive subline (left panel, isotype control; middle panel, parent cell line; right panel, GCTM-5 positive subclone). C, double label indirect immunofluorescence micrographs of SW1990 parent cell line stained with GCTM-5 or CA 19-9. D, foetal colonic mucosa stained with GCTM-5, ENPRO1, and CA 19-9 and visualised with horseradish peroxidase/DAB. E, GCTM-5 immunoprecipitates of supernatant from cultured cell lines separated by reducing SDS-PAGE gels and blotted with antibody to LGALSBP3. F, sections of pancreatic adenocarcinoma stained with GCTM-5 and visualised with horseradish peroxidase/DAB.

Antibodies to a CA 19-9 Related Antigen Complex Identify a Subset of SOX9 Expressing Cells In Human Foetal Pancreas and Pancreatic Adenocarcinoma. Alison M. Farley, David R. Braxton, Jonathan Li, Karl Trounson, Subhanwita Sakar-Dey, Bhavana Nayer, Tatsuhiko Ikeda, Kevin X. Lau, Winita Hardikar, Kouichi Hasegawa, and Martin F. Pera

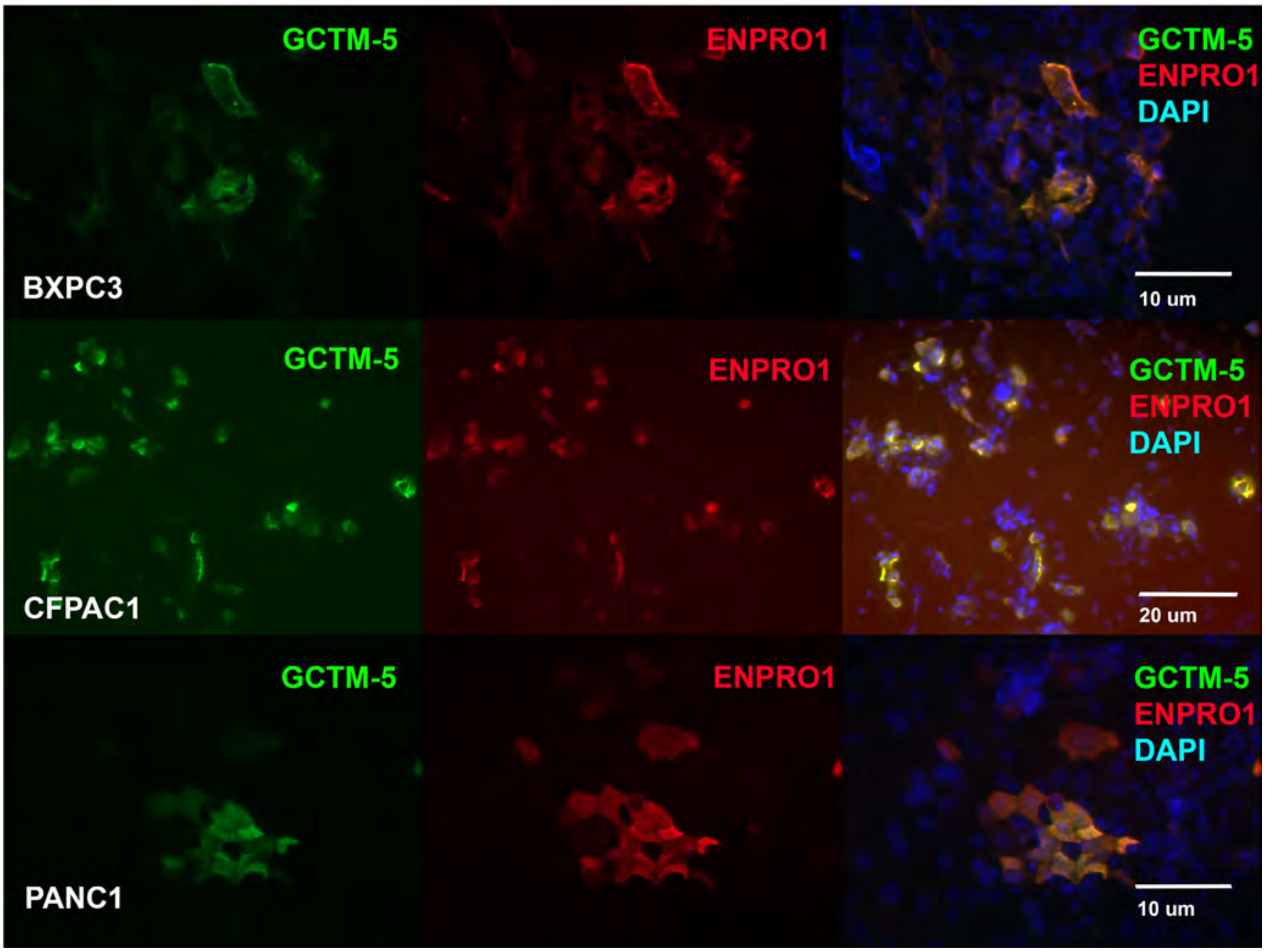

Supplementary Figure S2. Double label indirect immunofluorescence micrographs of cultured pancreatic adenocarcinoma cell lines stained with GCTM-5 and ENPRO1.

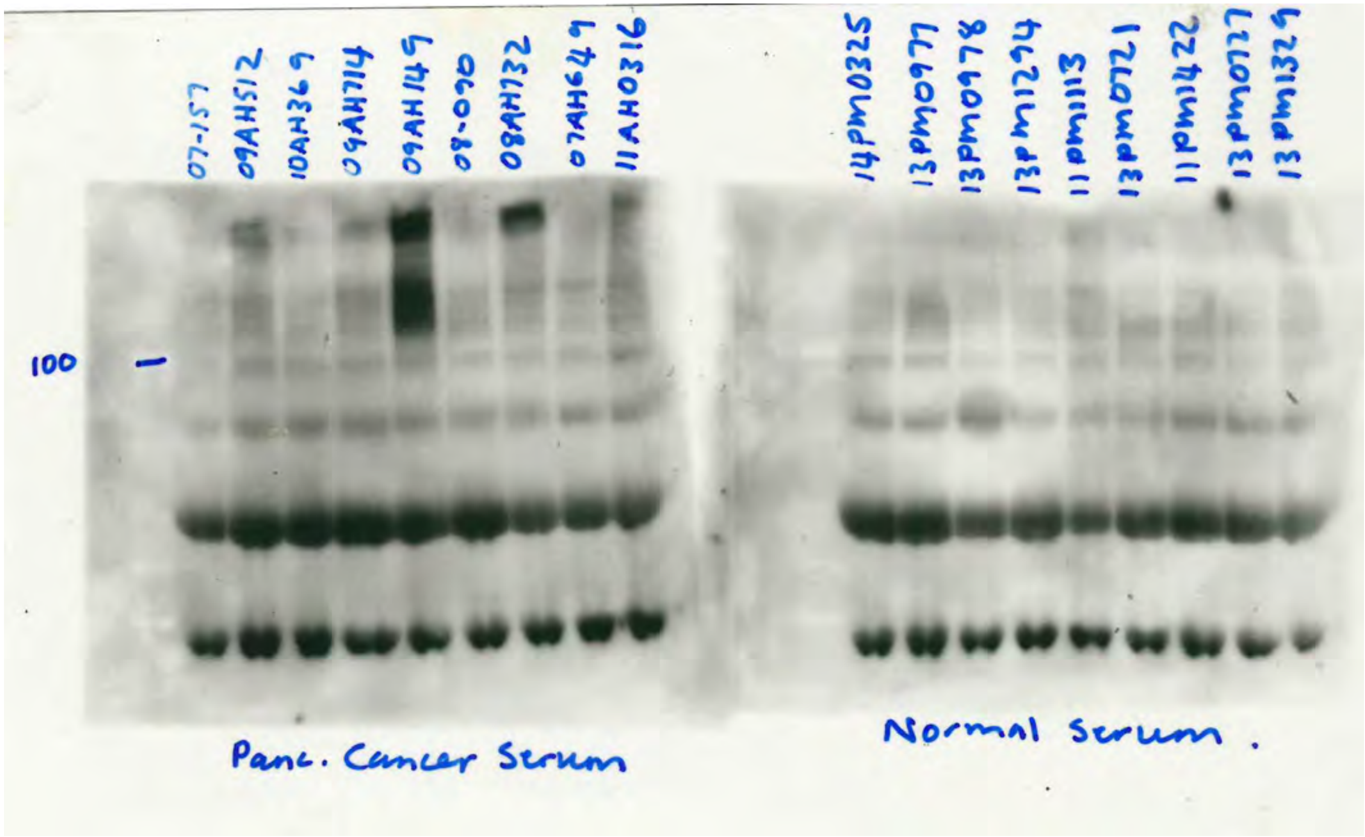

Supplementary Figure S3. Immunoprecipitaton followed by reducing SDS-PAGE and immunoblotting with GCTM-5 on pancreatic cancer patient and control sera (left and right blots respectively). The entire blot from the cropped image in Figure 6 is shown. The top band represents specific staining of the high molecular weight antigen complex in sera; other bands are background staining seen in normal sera.

Antibodies to a CA 19-9 Related Antigen Complex Identify a Subset of SOX9 Expressing Cells In Human Foetal Pancreas and Pancreatic Adenocarcinoma.

Alison M. Farley, David R. Braxton, Jonathan Li, Karl Trounson, Subhanwita Sakar-Dey, Bhavana Nayer, Tatsuhiko Ikeda, Kevin X. Lau, Winita Hardikar, Kouichi Hasegawa, and Martin F. Pera

Supplementary Table S1. Primary Antibodies Used in this Study

Antibody	Source	code	Host	Dilution
GCTM-5 Hybridoma	Pera Lab	N/A	Mouse IgG1	neat
Enpro1 Hybridoma	Pera Lab	N/A	Mouse IgG1	neat
CA19-9	Novus Biological	NBP2-15182	Mouse IgM	1/200
Sox9	Milipore	AB5535	Rabbit IgG	1/200
Ncam	Abcam	ab75813	Rabbit IgG	1/100
Lgr5	Abcam	ab75732	Rabbit polyclonal	1/100
Epcam	Abcam	ab124825	Rabbit polyclonal	1/100
Cytokeratin19	Abcam	ab15463	Rabbit IgG	1/100
CD133	Aviva Systems Biologicals	OAA100379	Rabbit IgG	1/100
Beta Catenin	Abcam	ab32572	Rabbit IgG	1/200
Dckl1	Abcam	ab31704	Rabbit IgG	1/100
Mucin1	Abcam	ab28081	Mouse IgG3	1/250
Mucin2	Abcam	ab134119	Rabbit 1gG	1/250
Mucin5A	Abcam	ab3649	mouse 1gG1	1/250
Mucin16	Abcam	ab1003	mouse 1gG1	1/250
Galectin-3BP/MAC-2BP	R&D systems	AF2226	Goat IgG	1/250

Supplementary Table S2. Glycan Array Analysis of Binding Specificity of GCTM-5

	Structure on Masterlist	Average RFU	StDev	% CV
1	Gala-Sp8	3	7	221
2	Glca-Sp8	2	10	429
3	Mana-Sp8	9	11	119
4	GalNAca-Sp8	6	4	65
5	GalNAca-Sp15	-1	6	-486
6	Fuca-Sp8	11	7	64
7	Fuca-Sp9	1	4	360
8	Rhaa-Sp8	5	3	74
9	Neu5Aca-Sp8	4	2	42
10	Neu5Aca-Sp11	12	11	92
11	Neu5Acb-Sp8	4	3	85
12	Galb-Sp8	9	8	87
13	Glcb-Sp8	8	8	100
14	Manb-Sp8	13	12	98
15	GalNAcb-Sp8	8	6	76
16	GlcNAcb-Sp0	8	3	40
17	GlcNAcb-Sp8	-2	4	-240
18	GlcN(Gc)b-Sp8	13	5	41
19	Galb1-4GlcNAcb1-6(Galb1-4GlcNAcb1-3)GalNAca-Sp8	10	14	144
20	Galb1-4GlcNAcb1-6(Galb1-4GlcNAcb1-3)GalNAc-Sp14	9	15	158
21	GlcNAcb1-6(GlcNAcb1-4)(GlcNAcb1-3)GlcNAc-Sp8	0	9	4333
22	6S(3S)Galb1-4(6S)GlcNAcb-Sp0	3	5	161
23	6S(3S)Galb1-4GlcNAcb-Sp0	-1	2	-117
24	(3S)Galb1-4(Fuca1-3)(6S)Glc-Sp0	8	4	43
25	(3S)Galb1-4Glcb-Sp8	10	7	70
26	(3S)Galb1-4(6S)Glcb-Sp0	-1	4	-393
27	(3S)Galb1-4(6S)Glcb-Sp8	5	8	166
28	(3S)Galb1-3(Fuca1-4)GlcNAcb-Sp8	28	23	83
29	(3S)Galb1-3GalNAca-Sp8	9	10	121
30	(3S)Galb1-3GlcNAcb-Sp0	2	3	134
31	(3S)Galb1-3GlcNAcb-Sp8	7	8	104
32	(3S)Galb1-4(Fuca1-3)GlcNAc-Sp0	8	7	92
33	(3S)Galb1-4(Fuca1-3)GlcNAc-Sp8	5	4	79
34	(3S)Galb1-4(6S)GlcNAcb-Sp0	0	3	-12877
35	(3S)Galb1-4(6S)GlcNAcb-Sp8	5	3	52
36	(3S)Galb1-4GlcNAcb-Sp0	5	4	81
37	(3S)Galb1-4GlcNAcb-Sp8	5	4	71
38	(3S)Galb-Sp8	4	3	80
39	(6S)(4S)Galb1-4GlcNAcb-Sp0	0	2	-20578
40	(4S)Galb1-4GlcNAcb-Sp8	3	2	81
41	(6P)Mana-Sp8	1	3	200
42	(6S)Galb1-4Glcb-Sp0	2	2	79
43	(6S)Galb1-4Glcb-Sp8	9	4	43
44	(6S)Galb1-4GlcNAcb-Sp8	3	5	138
45	(6S)Galb1-4(6S)Glcb-Sp8	5	2	43
46	Neu5Aca2-3(6S)Galb1-4GlcNAcb-Sp8	1	9	1037
47	(6S)GlcNAcb-Sp8	6	4	73
48	Neu5,9Ac$_2$a-Sp8	10	4	38
49	Neu5,9Ac2a2-6Galb1-4GlcNAcb-Sp8	11	3	29
50	Mana1-6(Mana1-3)Manb1-4GlcNAcb1-4GlcNAcb-Sp12	4	5	103
51	Mana1-6(Mana1-3)Manb1-4GlcNAcb1-4GlcNAcb-Sp13	1	5	338
52	GlcNAcb1-2Mana1-6(GlcNAcb1-2Mana1-3)Manb1-4GlcNAcb1-4GlcNAcb-Sp12	16	5	33
53	GlcNAcb1-2Mana1-6(GlcNAcb1-2Mana1-3)Manb1-4GlcNAcb1-4GlcNAcb-Sp13	2	4	200
54	Galb1-4GlcNAcb1-2Mana1-6(Galb1-4GlcNAcb1-2Mana1-3)Manb1-4GlcNAcb1-4GlcNAcb-Sp12	7	5	71
55	Neu5Aca2-6Galb1-4GlcNAcb1-2Mana1-6(Neu5Aca2-6Galb1-4GlcNAcb1-2Mana1-3)Manb1-4GlcNAcb1-4GlcNAcb-Sp12	12	5	38
56	Neu5Aca2-6Galb1-4GlcNAcb1-2Mana1-6(Neu5Aca2-6Galb1-4GlcNAcb1-2Man-a1-3)Manb1-4GlcNAcb1-4GlcNAcb-Sp21	3	5	142
57	Neu5Aca2-6Galb1-4GlcNAcb1-2Mana1-6(Neu5Aca2-6Galb1-4GlcNAcb1-2Mana1-3)Manb1-4GlcNAcb1-4GlcNAcb-Sp24	3	1	29
58	Fuca1-2Galb1-3GalNAcb1-3Gala-Sp9	2	2	78
59	Fuca1-2Galb1-3GalNAcb1-3Gala1-4Galb1-4Glcb-Sp9	4	6	164
60	Fuca1-2Galb1-3(Fuca1-4)GlcNAcb-Sp8	7	6	89
61	Fuca1-2Galb1-3GalNAca-Sp8	7	1	18
62	Fuca1-2Galb1-3GalNAca-Sp14	6	2	25
63	Fuca1-2Galb1-3GalNAcb1-4(Neu5Aca2-3)Galb1-4Glcb-Sp0	2	6	287
64	Fuca1-2Galb1-3GalNAcb1-4(Neu5Aca2-3)Galb1-4Glcb-Sp9	1	2	182
65	Fuca1-2Galb1-3GlcNAcb1-3Galb1-4Glcb-Sp8	10	6	58
66	Fuca1-2Galb1-3GlcNAcb1-3Galb1-4Glcb-Sp10	6	3	50
67	Fuca1-2Galb1-3GlcNAcb-Sp0	-2	2	-103
68	Fuca1-2Galb1-3GlcNAcb-Sp8	2	3	130
69	Fuca1-2Galb1-4(Fuca1-3)GlcNAcb1-3Galb1-4(Fuca1-3)GlcNAcb-Sp0	6	6	106
70	Fuca1-2Galb1-4(Fuca1-3)GlcNAcb1-3Galb1-4(Fuca1-3)GlcNAcb1-3Galb1-4(Fuca1-3)GlcNAcb-Sp0	-4	5	-122
71	Fuca1-2Galb1-4(Fuca1-3)GlcNAcb-Sp0	6	4	63
72	Fuca1-2Galb1-4(Fuca1-3)GlcNAcb-Sp8	8	4	43
73	Fuca1-2Galb1-4GlcNAcb1-3Galb1-4GlcNAcb-Sp0	0	3	-1275
74	Fuca1-2Galb1-4GlcNAcb1-3Galb1-4GlcNAcb1-3Galb1-4GlcNAcb-Sp0	14	7	51
75	Fuca1-2Galb1-4GlcNAcb-Sp0	6	2	34
76	Fuca1-2Galb1-4GlcNAcb-Sp8	10	3	28
77	Fuca1-2Galb1-4Glcb-Sp0	6	9	153
78	Fuca1-2Galb-Sp8	5	3	65
79	Fuca1-3GlcNAcb-Sp8	6	7	124
80	Fuca1-4GlcNAcb-Sp8	11	7	69
81	Fucb1-3GlcNAcb-Sp8	2	4	175
82	GalNAca1-3(Fuca1-2)Galb1-3GlcNAcb-Sp0	12	2	14
83	GalNAca1-3(Fuca1-2)Galb1-4(Fuca1-3)GlcNAcb-Sp0	2	4	206
84	(3S)Galb1-4(Fuca1-3)Glcb-Sp0	6	6	98
85	GalNAca1-3(Fuca1-2)Galb1-4GlcNAcb-Sp0	2	8	320
86	GalNAca1-3(Fuca1-2)Galb1-4GlcNAcb-Sp8	11	3	25
87	GalNAca1-3(Fuca1-2)Galb1-4Glcb-Sp0	9	2	18
88	GlcNAcb1-3Galb1-3GalNAca-Sp8	8	5	60
89	GalNAca1-3(Fuca1-2)Galb-Sp8	10	9	90
90	GalNAca1-3(Fuca1-2)Galb-Sp18	9	2	19
91	GalNAca1-3GalNAcb-Sp8	20	10	52
92	GalNAca1-3Galb-Sp8	4	3	80
93	GalNAca1-4(Fuca1-2)Galb1-4GlcNAcb-Sp8	-6	7	-119
94	GalNAcb1-3GalNAca-Sp8	8	11	133

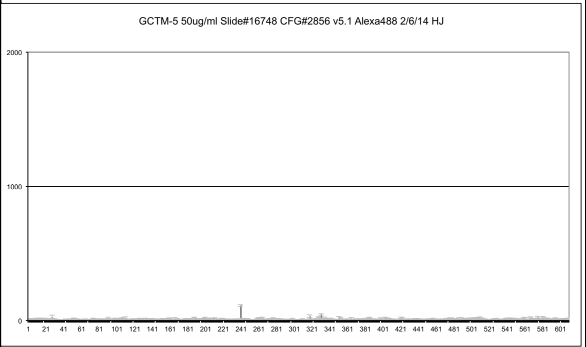

Chart Number	GCTM-5 50ug/ml Slide#16748 CFG#2856 v5.1 Alexa488 2/6/14 HJ	Average RFU	StDev	% CV
240	Neu5Aca2-3Galb1-3(Fuca1-4)GlcNAcb1-3Galb1-4(Fuca1-3)GlcNAcb-Sp0	112	13	11
331	Neu5Aca2-3Galb1-3(Fuca1-4)GlcNAcb1-3Galb1-3(Fuca1-4)GlcNAcb-Sp0	43	15	34
318	Neu5Aca2-6Galb1-4GlcNAcb1-2Mana1-6(Neu5Aca2-3Galb1-4GlcNAcb1-2Mana1-3)Manb1-4GlcNAcb1-4GlcNAcb-Sp12	32	20	64
28	(3S)Galb1-3(Fuca1-4)GlcNAcb-Sp8	28	23	83
575	Galb1-4GlcNAcb1-3Galb1-4GlcNAcb1-2Mana1-6(Galb1-4GlcNAcb1-3Galb1-4GlcNAcb1-2Mana1-3)Manb1-4GlcNAcb1-4(Fuca1-6)GlcNAcb-Sp24	25	13	51
328	Neu5,9Ac2a2-3Galb1-4GlcNAcb-Sp0	23	10	42
188	GlcNAcb1-4Galb1-4GlcNAcb-Sp8	23	3	15
200	G-ol-Sp8	22	5	21
581	Galb1-4GlcNAcb1-3Galb1-4GlcNAcb1-3Galb1-4GlcNAcb1-3Galb1-4GlcNAcb1-3Galb1-4GlcNAcb1-2Mana1-6(Galb1-4GlcNAcb1-3Galb1-4GlcNAcb1-3Galb1-4GlcNAcb1-3Galb1-4GlcNAcb1-3Galb1-4GlcNAcb1-2Mana1-3)Manb1-4GlcNAcb1-4(Fuca1-6)GlcNAcb-Sp19	22	17	79
110	Gala1-3GalNAca-Sp8	21	12	56
510	Galb1-3(6S)GlcNAcb-Sp8	21	12	56
385	Galb1-4(Fuca1-3)GlcNAcb1-6(Fuca1-4(Fuca1-2Galb1-3)GlcNAcb1-3)Galb1-4Glc-Sp21	20	8	40
91	GalNAca1-3GalNAcb-Sp8	20	10	52
351	Mana1-6(Galb1-4GlcNAcb1-2Mana1-3)Manb1-4GlcNAcb1-4GlcNAcb-Sp12	20	20	103
567	Galb1-3GlcNAcb1-6(Galb1-3)GalNAc-Sp14	19	17	90
505	Fuca1-2Galb1-3GlcNAcb1-6(Fuca1-2Galb1-3GlcNAcb1-3)GalNAca-Sp14	19	9	46
353	Galb1-4GlcNAcb1-2Mana1-6(Galb1-4GlcNAcb1-2Mana1-3)Manb1-4GlcNAcb1-4(Fuca1-6)GlcNAcb-Sp22	19	9	49
265	Neu5Aca2-6GalNAca-Sp8	19	13	66
335	GalNAca1-3(Fuca1-2)Galb1-4GlcNAcb1-3Galb1-4GlcNAcb-Sp0	19	13	71
403	Gala1-4Galb1-3GlcNAcb1-2Mana1-6(Gala1-4Galb1-3GlcNAcb1-2Mana1-3)Manb1-4GlcNAcb1-4GlcNAcb-Sp19	19	12	65
421	GlcNAcb1-2(GlcNAcb1-6)Mana1-6(GlcNAcb1-2Mana1-3)Manb1-4GlcNAcb1-4GlcNAcb-Sp19	18	16	94
478	Neu5Aca2-3Galb1-4GlcNAcb1-2Mana-Sp0	17	6	37
559	Galb1-4GlcNAcb1-3Galb1-4GlcNAcb1-6(Galb1-4GlcNAcb1-3Galb1-4GlcNAcb1-2)Mana1-6(Galb1-4GlcNAcb1-3Galb1-4GlcNAcb1-2Mana1-3)Mana1-4GlcNAcb1-4GlcNAc-Sp24	17	15	91
160	Galb1-4GlcNAcb1-3GalNAc-Sp14	17	7	42
261	Neu5Aca2-3Galb1-4GlcNAcb1-3Galb1-4GlcNAcb-Sp0	16	12	74
52	GlcNAcb1-2Mana1-6(GlcNAcb1-2Mana1-3)Manb1-4GlcNAcb1-4GlcNAcb-Sp12	16	5	33
277	Galb1-3(Fuca1-4)GlcNAcb1-3Galb1-3(Fuca1-4)GlcNAcb-Sp0	16	10	64
211	Mana1-2Mana1-6(Mana1-3)Mana1-6(Mana1-2Mana1-2Mana1-3)Manb1-4GlcNAcb1-4GlcNAcb-Sp12	16	10	63
106	Gala1-3(Fuca1-2)Galb1-4Glcb-Sp0	16	9	59
476	Galb1-3GlcNAcb1-2Mana1-6(GlcNAcb1-4)(Galb1-3GlcNAcb1-2Mana1-3)Manb1-4GlcNAcb1-4GlcNAcb-Sp21	16	9	60
562	GalNAcb1-3GlcNAcb-Sp0	16	12	75
509	Galb1-3GlcNAca1-3Galb1-4GlcNAcb-Sp8	16	8	53
398	Galb1-4GlcNAcb1-2Mana1-6(GlcNAcb1-2Mana1-3)Manb1-4GlcNAcb1-4GlcNAc-Sp12	15	10	63
513	(3S)GalNAcb1-4(3S)GlcNAc-Sp8	15	6	37
99	GalNAcb1-4GlcNAcb-Sp8	15	5	34
582	Galb1-4GlcNAcb1-3Galb1-4GlcNAcb1-6(Galb1-4GlcNAcb1-3Galb1-4GlcNAb1-2)Mana1-6(Galb1-4GlcNAcb1-3Galb1-4GlcNAcb1-2Mana1-3)Manb1-4GlcNAcb1-4(Fuca1-6)GlcNAcb-Sp24	15	13	83
489	Gala1-3Galb1-3GlcNAcb1-6GalNAca-Sp14	15	18	122
259	Neu5Aca2-3Galb1-4GlcNAcb-Sp0	15	6	44
382	Galb1-3GlcNAcb1-3Galb1-4GlcNAcb1-6(Galb1-3GlcNAcb1-3)Galb1-4Glcb-Sp0	14	5	35
159	Galb1-4GlcNAcb1-3GalNAca-Sp8	14	3	17
583	GlcNAcb1-3Galb1-4GlcNAcb1-3Galb1-4GlcNAcb1-6(GlcNAcb1-3Galb1-4GlcNAcb1-3Galb1-4GlcNAb1-2)Mana1-6(GlcNAcb1-3Galb1-4GlcNAcb1-3Galb1-4GlcNAcb1-2Mana1-3)Manb1-4GlcNAcb1-4(Fuca1-6)GlcNAcb-Sp24	14	10	71
336	GalNAca1-3(Fuca1-2)Galb1-4GlcNAcb1-3Galb1-4GlcNAcb1-3Galb1-4GlcNAcb-Sp0	14	5	39
486	Neu5Aca2-3Galb1-3GlcNAcb1-2Mana1-6(GlcNAcb1-4)(Neu5Aca2-3Galb1-3GlcNAcb1-2Mana1-3)Manb1-4GlcNAcb1-4GlcNAc-Sp21	14	10	72
407	Galb1-3GlcNAca1-6Galb1-4GlcNAcb-Sp0	14	8	56
74	Fuca1-2Galb1-4GlcNAcb1-3Galb1-4GlcNAcb1-3Galb1-4GlcNAcb-Sp0	14	7	51
497	Fuca1-2(6S)Galb1-3GlcNAcb-Sp0	14	9	65
543	Neu5Gca2-3Galb1-4GlcNAcb1-3Galb1-4GlcNAcb1-2Mana1-6(Neu5Gca2-3Galb1-4GlcNAcb1-3Galb1-4GlcNAcb1-2Mana1-3)Manb1-4GlcNAcb1-4GlcNAcb-Sp24	14	12	86
423	Gala1-3(Fuca1-2)Galb1-3GlcNAcb1-3GalNAc-Sp14	14	13	97
457	GalNAca1-3(Fuca1-2)Galb1-3GlcNAcb1-2Mana1-6(GalNAca1-3(Fuca1-2)Galb1-3GlcNAcb1-2Mana1-3)Manb1-4GlcNAcb1-4(Fuca1-6)GlcNAcb-Sp22	14	7	55
219	(3S)Galb1-4(Fuca1-3)(6S)GlcNAcb-Sp8	13	8	57
242	Neu5Aca2-3Galb1-3(6S)GalNAca-Sp8	13	7	55
330	Neu5Aca2-6Galb1-4GlcNAcb1-3Galb1-3GlcNAcb-Sp0	13	10	76
501	Fuca1-2(6S)Galb1-3(6S)GlcNAcb-Sp0	13	14	103
595	GlcNAcb1-3Galb1-4GlcNAcb1-6(GlcNAcb1-3Galb1-4GlcNAcb1-3)GalNAca-Sp14	13	14	105
568	Galb1-4GlcNAcb1-3Galb1-4GlcNAcb1-3Galb1-4GlcNAcb1-3Galb1-4GlcNAcb1-3Galb1-4GlcNAcb1-3Galb1-4GlcNAcb1-2Mana1-6(Galb1-4GlcNAcb1-3Galb1-4GlcNAcb1-3Galb1-4GlcNAcb1-3Galb1-4GlcNAcb1-3Galb1-4GlcNAcb1-3Galb1-4GlcNAcb1-2Mana1-3)Manb1-4GlcNAcb1-4GlcNAcb-Sp25	13	10	78
529	Neu5Aca2-3Galb1-3GalNAcb1-4Galb1-4Glcb-Sp0	13	7	55
547	Galb1-4GlcNAcb1-3Galb1-4GlcNAcb1-3Galb1-4GlcNAcb1-2Mana1-6(Galb1-4GlcNAcb1-3Galb1-4GlcNAcb1-3Galb1-4GlcNAcb1-2Mana1-3)Manb1-4GlcNAcb1-4GlcNAcb-Sp12	13	11	84
586	Galb1-4GlcNAcb1-3Galb1-4GlcNAcb1-3Galb1-4GlcNAcb1-3Galb1-4GlcNAcb1-6(Galb1-4GlcNAcb1-3Galb1-4GlcNAcb1-3Galb1-4GlcNAcb1-3Galb1-4GlcNAb1-2)Mana1-6(Galb1-4GlcNAcb1-3Galb1-4GlcNAcb1-3Galb1-4GlcNAcb1-3Galb1-4GlcNAcb1-2Mana1-3)Manb1-4GlcNAcb1-4(Fuca1-6)GlcNAcb-Sp24	13	10	81
588	Galb1-4GlcNAcb1-3Galb1-4GlcNAcb1-3Galb1-4GlcNAcb1-3Galb1-4GlcNAcb1-3Galb1-4GlcNAcb1-6(Galb1-4GlcNAcb1-3Galb1-4GlcNAcb1-3Galb1-4GlcNAcb1-3Galb1-4GlcNAcb1-3Galb1-4GlcNAb1-2)Mana1-6(Galb1-4GlcNAcb1-3Galb1-4GlcNAcb1-3Galb1-4GlcNAcb1-3Galb1-4GlcNAcb1-3Galb1-4GlcNAcb1-2Mana1-3)Manb1-4GlcNAcb1-4(Fuca1-6)GlcNAcb-Sp24	13	3	26
141	Galb1-3GalNAca-Sp16	13	4	34
386	Galb1-3GlcNAcb1-3Galb1-4(Fuca1-3)GlcNAcb1-6(Galb1-3GlcNAcb1-3)Galb1-4Glc-Sp21	13	16	123
365	Fuca1-4(Galb1-3)GlcNAcb1-2Mana1-6(Fuca1-4(Galb1-3)GlcNAcb1-2Mana1-3)Manb1-4GlcNAcb1-4(Fuca1-6)GlcNAcb-Sp22	13	18	144
609	GlcNAcb1-3Fuca-Sp21	13	11	82
166	Galb1-4GlcNAcb1-6(Galb1-3)GalNAca-Sp8	13	18	144
540	GlcNAcb1-3Galb1-4GlcNAcb1-2Mana1-6(GlcNAcb1-3Galb1-4GlcNAcb1-2Mana1-3)Manb1-4GlcNAcb1-4GlcNAcb-Sp25	13	10	76
299	Neu5Aca2-3Galb1-4(Fuca1-3)GlcNAcb1-6(Galb1-3)GalNAca-Sp14	13	8	65
594	GlcNAcb1-3Galb1-4GlcNAcb1-6(Galb1-3)GalNAca-Sp14	13	9	70
18	GlcN(Gc)b-Sp8	13	5	41
14	Manb-Sp8	13	12	98
342	GlcNAca1-4Galb1-4GlcNAcb1-3Galb1-4(Fuca1-3)GlcNAcb1-3Galb1-4(Fuca1-3)GlcNAcb-Sp0	13	11	86
183	GlcNAcb1-3Galb1-4GlcNAcb-Sp8	12	6	47
263	Neu5Aca2-3Galb1-4Glcb-Sp0	12	10	78
494	Fuca1-2Galb1-4GlcNAcb1-6GalNAca-Sp14	12	9	72
133	GlcNAcb1-6(Galb1-3)GalNAca-Sp8	12	10	83
391	Galb1-3GlcNAcb1-3GalNAca-Sp14	12	2	14
206	KDNa2-3Galb1-4GlcNAcb-Sp0	12	13	110
102	Gala1-3(Fuca1-2)Galb1-3GlcNAcb-Sp8	12	4	34
310	GlcNAcb1-4GlcNAcb-Sp10	12	5	39
55	Neu5Aca2-6Galb1-4GlcNAcb1-2Mana1-6(Neu5Aca2-6Galb1-4GlcNAcb1-2Mana1-3)Manb1-4GlcNAcb1-4GlcNAcb-Sp12	12	5	38
179	GlcNAcb1-3GalNAca-Sp8	12	8	67
158	Galb1-4GalNAcb1-3(Fuca1-2)Galb1-4GlcNAcb-Sp8	12	4	30
10	Neu5Aca-Sp11	12	11	92
603	Neu5Aca2-3Galb1-4GlcNAcb1-3Galb1-4GlcNAcb1-2Mana1-6(Neu5Aca2-3Galb1-4GlcNAcb1-3Galb1-4GlcNAcb1-2Mana1-3)Manb1-4GlcNAcb1-4GlcNAcb-Sp12	12	7	61
499	Fuca1-2Galb1-4GlcNAcb1-2Mana-Sp0	12	2	20
169	Galb1-4GlcNAcb-Sp8	12	9	73
541	Galb1-4GlcNAcb1-3Galb1-4GlcNAcb1-2Mana1-6(Galb1-4GlcNAcb1-3Galb1-4GlcNAcb1-2Mana1-3)Manb1-4GlcNAcb1-4GlcNAcb-Sp12	12	6	53
187	GlcNAcb1-6(GlcNAcb1-4)GalNAca-Sp8	12	6	48
82	GalNAca1-3(Fuca1-2)Galb1-3GlcNAcb-Sp0	12	2	14
472	Fuca1-2Galb1-4(Fuca1-3)GlcNAcb1-2Mana1-6(Fuca1-2Galb1-4(Fuca1-3)GlcNAcb1-2Mana1-3)Manb1-4GlcNAcb1-4(Fuca1-6)GlcNAcb-Sp24	12	9	79
341	GlcNAca1-4Galb1-4GlcNAcb1-3Galb1-4Glcb-Sp0	12	6	51
132	Galb1-4GlcNAcb1-6GalNAc-Sp14	12	8	73
286	Neu5Gca2-6Galb1-4GlcNAcb-Sp0	12	2	16
475	GlcNAcb1-6(GlcNAcb1-2)Mana1-6(GlcNAcb1-2Mana1-3)Manb1-4GlcNAcb1-4(Fuca1-6)GlcNAcb-Sp24	12	6	49
482	Neu5Aca2-6Galb1-4GlcNAcb1-2Mana1-6(Neu5Aca2-6Galb1-4GlcNAcb1-2Mana1-3)Manb1-4GlcNAcb1-4(Fuca1-6)GlcNAcb-Sp24	12	6	52

95	GalNAcb1-3(Fuca1-2)Galb-Sp8	8	2	24
96	GalNAcb1-3Gala1-4Galb1-4GlcNAcb-Sp0	9	3	31
97	GalNAcb1-4(Fuca1-3)GlcNAcb-Sp0	1	3	227
98	GalNAcb1-4GlcNAcb-Sp0	6	9	146
99	GalNAcb1-4GlcNAcb-Sp8	15	5	34
100	Gala1-2Galb-Sp8	3	1	37
101	Gala1-3(Fuca1-2)Galb1-3GlcNAcb-Sp0	11	6	51
102	Gala1-3(Fuca1-2)Galb1-3GlcNAcb-Sp8	12	4	34
103	Gala1-3(Fuca1-2)Galb1-4(Fuca1-3)GlcNAcb-Sp0	5	3	61
104	Gala1-3(Fuca1-2)Galb1-4(Fuca1-3)GlcNAcb-Sp8	4	7	187
105	Gala1-3(Fuca1-2)Galb1-4GlcNAc-Sp0	3	10	364
106	Gala1-3(Fuca1-2)Galb1-4Glcb-Sp0	16	9	59
107	Gala1-3(Fuca1-2)Galb-Sp8	5	4	85
108	Gala1-3(Fuca1-2)Galb-Sp18	6	6	94
109	Gala1-4(Gala1-3)Galb1-4GlcNAcb-Sp8	8	7	91
110	Gala1-3GalNAca-Sp8	21	12	56
111	Gala1-3GalNAca-Sp16	7	6	83
112	Gala1-3GalNAcb-Sp8	4	4	103
113	Gala1-3Galb1-4(Fuca1-3)GlcNAcb-Sp8	5	4	71
114	Gala1-3Galb1-3GlcNAcb-Sp0	5	9	186
115	Gala1-3Galb1-4GlcNAcb-Sp8	9	4	45
116	Gala1-3Galb1-4Glcb-Sp0	5	5	95
117	Gala1-3Galb1-4Glc-Sp10	4	3	81
118	Gala1-3Galb-Sp8	8	8	101
119	Gala1-4(Fuca1-2)Galb1-4GlcNAcb-Sp8	10	7	72
120	Gala1-4Galb1-4GlcNAcb-Sp0	3	8	265
121	Gala1-4Galb1-4GlcNAcb-Sp8	5	4	79
122	Gala1-4Galb1-4Glcb-Sp0	5	2	36
123	Gala1-4GlcNAcb-Sp8	9	8	88
124	Gala1-6Glcb-Sp8	5	2	50
125	Galb1-2Galb-Sp8	6	6	86
126	Galb1-3(Fuca1-4)GlcNAcb1-3Galb1-4(Fuca1-3)GlcNAcb-Sp0	11	11	99
127	Galb1-3GlcNAcb1-3Galb1-4(Fuca1-3)GlcNAcb-Sp0	9	6	73
128	Galb1-3(Fuca1-4)GlcNAc-Sp0	8	7	84
129	Galb1-3(Fuca1-4)GlcNAc-Sp8	11	4	40
130	Fuca1-4(Galb1-3)GlcNAcb-Sp8	5	3	62
131	Galb1-4GlcNAcb1-6GalNAca-Sp8	6	7	128
132	Galb1-4GlcNAcb1-6GalNAc-Sp14	12	8	73
133	GlcNAcb1-6(Galb1-3)GalNAca-Sp8	12	10	83
134	GlcNAcb1-6(Galb1-3)GalNAca-Sp14	5	6	110
135	Neu5Aca2-6(Galb1-3)GalNAca-Sp8	7	2	30
136	Neu5Aca2-6(Galb1-3)GalNAca-Sp14	1	4	501
137	Neu5Acb2-6(Galb1-3)GalNAca-Sp8	10	7	71
138	Neu5Aca2-6(Galb1-3)GlcNAcb1-4Galb1-4Glcb-Sp10	6	9	134
139	Galb1-3GalNAca-Sp8	4	3	67
140	Galb1-3GalNAca-Sp14	10	10	104
141	Galb1-3GalNAca-Sp16	13	4	34
142	Galb1-3GalNAcb-Sp8	9	7	81
143	Galb1-3GalNAcb1-3Gala1-4Galb1-4Glcb-Sp0	7	5	65
144	Galb1-3GalNAcb1-4(Neu5Aca2-3)Galb1-4Glcb-Sp0	8	4	56
145	Galb1-3GalNAcb1-4Galb1-4Glcb-Sp8	0	3	852
146	Galb1-3Galb-Sp8	7	9	126
147	Galb1-3GlcNAcb1-3Galb1-4GlcNAcb-Sp0	-2	1	-65
148	Galb1-3GlcNAcb1-3Galb1-4Glcb-Sp10	1	4	421
149	Galb1-3GlcNAcb-Sp0	8	8	100
150	Galb1-3GlcNAcb-Sp8	3	3	98
151	Galb1-4(Fuca1-3)GlcNAcb-Sp0	5	3	69
152	Galb1-4(Fuca1-3)GlcNAcb-Sp8	10	11	106
153	Galb1-4(Fuca1-3)GlcNAcb1-3Galb1-4(Fuca1-3)GlcNAcb-Sp0	10	4	34
154	Galb1-4(Fuca1-3)GlcNAcb1-3Galb1-4(Fuca1-3)GlcNAcb1-3Galb1-4(Fuca1-3)GlcNAcb-Sp0	2	9	429
155	Galb1-4(6S)Glcb-Sp0	9	8	92
156	Galb1-4(6S)Glcb-Sp8	9	4	47
157	Galb1-4GalNAca1-3(Fuca1-2)Galb1-4GlcNAcb-Sp8	-1	5	-331
158	Galb1-4GalNAcb1-3(Fuca1-2)Galb1-4GlcNAcb-Sp8	12	4	30
159	Galb1-4GlcNAcb1-3GalNAca-Sp8	14	3	17
160	Galb1-4GlcNAcb1-3GalNAc-Sp14	17	7	42
161	Galb1-4GlcNAcb1-3Galb1-4(Fuca1-3)GlcNAcb1-3Galb1-4(Fuca1-3)GlcNAcb-Sp0	7	5	77
162	Galb1-4GlcNAcb1-3Galb1-4GlcNAcb1-3Galb1-4GlcNAcb-Sp0	10	4	43
163	Galb1-4GlcNAcb1-3Galb1-4GlcNAcb-Sp0	3	3	114
164	Galb1-4GlcNAcb1-3Galb1-4Glcb-Sp0	8	2	26
165	Galb1-4GlcNAcb1-3Galb1-4Glcb-Sp8	5	4	77
166	Galb1-4GlcNAcb1-6(Galb1-3)GalNAca-Sp8	13	18	144
167	Galb1-4GlcNAcb1-6(Galb1-3)GalNAc-Sp14	3	7	268
168	Galb1-4GlcNAcb-Sp0	2	4	237
169	Galb1-4GlcNAcb-Sp8	12	9	73
170	Galb1-4GlcNAcb-Sp23	6	7	128
171	Galb1-4Glcb-Sp0	1	0	33
172	Galb1-4Glcb-Sp8	1	3	242
173	GlcNAca1-3Galb1-4GlcNAcb-Sp8	7	4	53
174	GlcNAca1-6Galb1-4GlcNAcb-Sp8	5	6	102
175	GlcNAcb1-2Galb1-3GalNAca-Sp8	4	4	117
176	GlcNAcb1-6(GlcNAcb1-3)GalNAca-Sp8	5	2	43
177	GlcNAcb1-6(GlcNAcb1-3)GalNAca-Sp14	6	14	247
178	GlcNAcb1-6(GlcNAcb1-3)Galb1-4GlcNAcb-Sp8	8	6	82
179	GlcNAcb1-3GalNAca-Sp8	12	8	67
180	GlcNAcb1-3GalNAca-Sp14	5	4	83
181	GlcNAcb1-3Galb-Sp8	8	13	162
182	GlcNAcb1-3Galb1-4GlcNAcb-Sp0	0	5	-8348
183	GlcNAcb1-3Galb1-4GlcNAcb-Sp8	12	6	47
184	GlcNAcb1-3Galb1-4GlcNAcb1-3Galb1-4GlcNAcb-Sp0	-2	7	-307
185	GlcNAcb1-3Galb1-4Glcb-Sp0	2	5	264
186	GlcNAcb1-4-MDPLys	2	7	390
187	GlcNAcb1-6(GlcNAcb1-4)GalNAca-Sp8	12	6	48
188	GlcNAcb1-4Galb1-4GlcNAcb-Sp8	23	3	15
189	GlcNAcb1-4GlcNAcb1-4GlcNAcb1-4GlcNAcb1-4GlcNAcb1-4GlcNAcb1-Sp8	0	4	-1037
190	GlcNAcb1-4GlcNAcb1-4GlcNAcb1-4GlcNAcb1-4GlcNAcb1-Sp8	8	9	112
191	GlcNAcb1-4GlcNAcb1-4GlcNAcb-Sp8	9	11	130
192	GlcNAcb1-6GalNAca-Sp8	3	2	55
193	GlcNAcb1-6GalNAca-Sp14	1	7	1080
194	GlcNAcb1-6Galb1-4GlcNAcb-Sp8	9	5	53
195	Glca1-4Glcb-Sp8	10	9	96
196	Glca1-4Glca-Sp8	-1	3	-399
197	Glca1-6Glca1-6Glcb-Sp8	11	11	100
198	Glcb1-4Glcb-Sp8	1	3	300
199	Glcb1-6Glcb-Sp8	8	10	124

49	Neu5,9Ac2a2-6Galb1-4GlcNAcb-Sp8	11	3	29
334	GalNAcb1-3Gala1-4Galb1-4GlcNAcb1-3Galb1-4Glcb-Sp0	11	9	76
126	Galb1-3(Fuca1-4)GlcNAcb1-3Galb1-4(Fuca1-3)GlcNAcb-Sp0	11	11	99
6	Fuca-Sp8	11	7	64
217	Manb1-4GlcNAcb-Sp0	11	5	44
570	(3S)GlcAb1-3Galb1-4GlcNAcb1-2Mana-Sp0	11	6	58
508	Galb1-4GlcNAcb1-6(Galb1-4GlcNAcb1-2)Mana1-6(GlcNAcb1-4)Galb1-4GlcNAcb1-4(Gal b1-4GlcNAcb1-2)Mana1-3)Manb1-4GlcNAcb1-4(Fuca1-6)GlcNAc-Sp21	11	8	76
101	Gala1-3(Fuca1-2)Galb1-3GlcNAcb-Sp0	11	6	51
370	GalNAca1-3(Fuca1-2)Galb1-4GlcNAcb1-2Mana1-6(GalNAca1-3(Fuca1-2)Galb1-4GlcNAcb1-2Mana1-3)Manb1-4GlcNAcb1-4GlcNAcb-Sp20	11	4	40
453	GalNAcb1-4Galb1-4Glcb-Sp0	11	2	14
574	GlcNAcb1-3Galb1-4GlcNAcb1-2Mana1-6(GlcNAcb1-3Galb1-4GlcNAcb1-2Mana1-3)Manb1-4GlcNAcb1-4(Fuca1-6)GlcNAcb-Sp24	11	7	66
409	Gala1-3(Fuca1-2)Galb1-4(Fuca1-3)Glcb-Sp21	11	5	47
380	Galb1-3GalNAca1-3(Fuca1-2)Galb1-4Glc-Sp0	11	7	65
577	Galb1-4GlcNAcb1-3Galb1-4GlcNAcb1-3Galb1-4GlcNAcb1-2Mana1-6(Galb1-4GlcNAcb1-3Galb1-4GlcNAcb1-3Galb1-4GlcNAcb1-2Mana1-3)Manb1-4GlcNAcb1-4(Fuca1-6)GlcNAcb-Sp24	11	3	31
578	GlcNAcb1-3Galb1-4GlcNAcb1-3Galb1-4GlcNAcb1-3Galb1-4GlcNAcb1-2Mana1-6(GlcNAcb1-3Galb1-4GlcNAcb1-3Galb1-4GlcNAcb1-3Galb1-4GlcNAcb1-2Mana1-3)Manb1-4GlcNAcb1-4(Fuca1-6)GlcNAcb-Sp24	11	5	44
573	Neu5Aca2-8Neu5Aca2-3Galb1-3GalNAcb1-4(Neu5Aca2-3)Galb1-4Glc-Sp21	11	2	21
528	GalNAcb1-4GlcNAcb1-2Mana-Sp0	11	6	55
129	Galb1-3(Fuca1-4)GlcNAc-Sp8	11	4	40
580	GlcNAcb1-3Galb1-4GlcNAcb1-3Galb1-4GlcNAcb1-3Galb1-4GlcNAcb1-3Galb1-4GlcNAcb1-2Mana1-6(GlcNAcb1-3Galb1-4GlcNAcb1-3Galb1-4GlcNAcb1-3Galb1-4GlcNAcb1-3Galb1-4GlcNAcb1-2Mana1-3)Manb1-4GlcNAcb1-4(Fuca1-6)GlcNAcb-Sp19	11	4	37
197	Glca1-6Glca1-6Glcb-Sp8	11	11	100
516	(4S)GalNAcb-Sp10	11	1	10
550	Galb1-4GlcNAcb1-3Galb1-4GlcNAcb1-3Galb1-4GlcNAcb1-3Galb1-4GlcNAcb1-2Mana1-6(Galb1-4GlcNAcb1-3Galb1-4GlcNAcb1-3Galb1-4GlcNAcb1-3Galb1-4GlcNAcb1-2Mana1-3)Manb1-4GlcNAcb1-4GlcNAcb-Sp25	11	11	108
420	Fuca1-2Galb1-4GlcNAcb1-2Mana1-6(Fuca1-2Galb1-4GlcNAcb1-2Mana1-3)Manb1-4GlcNAcb1-4(Fuca1-6)GlcNAcb-Sp22	11	11	100
274	Neu5Aca2-6Galb-Sp8	11	10	93
493	Galb1-4(Fuca1-3)GlcNAcb1-6(Neu5Aca2-6(Neu5Aca2-3Galb1-3)GlcNAcb1-3)Galb1-4Glc-Sp21	11	8	80
86	GalNAca1-3(Fuca1-2)Galb1-4GlcNAcb-Sp8	11	3	25
587	GlcNAcb1-3Galb1-4GlcNAcb1-3Galb1-4GlcNAcb1-3Galb1-4GlcNAcb1-3Galb1-4GlcNAcb1-6(GlcNAcb1-3Galb1-4GlcNAcb1-3Galb1-4GlcNAcb1-3Galb1-4GlcNAcb1-3Galb1-4GlcNAb1-2)Mana1-6(GlcNAcb1-3Galb1-4GlcNAcb1-3Galb1-4GlcNAcb1-3Galb1-4GlcNAcb1-3Galb1-4GlcNAcb1-2Mana1-3)Manb1-4GlcNAcb1-4(Fuca1-6)GlcNAcb-Sp24	11	4	34
80	Fuca1-4GlcNAcb-Sp8	11	7	69
152	Galb1-4(Fuca1-3)GlcNAcb-Sp8	10	11	106
153	Galb1-4(Fuca1-3)GlcNAcb1-3Galb1-4(Fuca1-3)GlcNAcb-Sp0	10	4	34
25	(3S)Galb1-4Glcb-Sp8	10	7	70
325	Neu5Aca2-3Galb1-4GlcNAcb1-2Mana1-6(Neu5Aca2-3Galb1-4GlcNAcb1-2Mana1-3)Manb1-4GlcNAcb1-4GlcNAcb-Sp12	10	10	97
539	GlcNAcb1-3Galb1-4GlcNAcb1-2Mana1-6(GlcNAcb1-3Galb1-4GlcNAcb1-2Mana1-3)Manb1-4GlcNAcb1-4GlcNAcb-Sp12	10	4	41
119	Gala1-4(Fuca1-2)Galb1-4GlcNAcb-Sp8	10	7	72
610	Galb1-3GalNAcb1-4(Neu5Aca2-8Neu5Aca2-8Neu5Aca2-3)Galb1-4Glcb-Sp21	10	1	7
441	Neu5Aca2-3Galb1-4GlcNAcb1-3Galb-Sp8	10	10	96
371	Gala1-3(Fuca1-2)Galb1-4GlcNAcb1-2Mana1-6(Gala1-3(Fuca1-2)Galb1-4GlcNAcb1-2Mana1-3)Manb1-4GlcNAcb1-4GlcNAcb-Sp20	10	5	46
162	Galb1-4GlcNAcb1-3Galb1-4GlcNAcb1-3Galb1-4GlcNAcb-Sp0	10	4	43
76	Fuca1-2Galb1-4GlcNAcb-Sp8	10	3	28
468	Gala1-3(Fuca1-2)Galb1-3GalNAcb-Sp8	10	5	45
430	Fuca1-3GlcNAcb1-6(Galb1-4GlcNAcb1-3)Galb1-4Glc-Sp21	10	5	44
452	Neu5Aca2-8Neu5Aca2-3Galb1-3GalNAcb1-4(Neu5Aca2-8Neu5Aca2-3)Galb1-4Glcb-Sp0	10	9	84
599	Galb1-4GlcNAcb1-3Galb1-3GalNAca-Sp14	10	9	89
48	Neu5,9Ac$_2$a-Sp8	10	4	38
222	Fuca1-2(6S)Galb1-4(6S)Glcb-Sp0	10	6	64
137	Neu5Acb2-6(Galb1-3)GalNAca-Sp8	10	7	71
207	Mana1-2Mana1-2Mana1-3Mana-Sp9	10	12	120
19	Galb1-4GlcNAcb1-6(Galb1-4GlcNAcb1-3)GalNAca-Sp8	10	14	144
437	Galb1-4GlcNAcb1-6(Galb1-4GlcNAcb1-2)Mana1-6(GlcNAcb1-4)(Galb1-4GlcNAcb1-2Mana1-3)Manb1-4GlcNAcb1-4GlcNAc-Sp21	10	11	105
526	Neu5Aca2-3Galb1-3GlcNAcb1-2Mana-Sp0	10	9	87
205	KDNa2-3Galb1-3GlcNAcb-Sp0	10	7	72
201	GlcAa-Sp8	10	5	50
374	Gala1-3(Fuca1-2)Galb1-3GlcNAcb1-2Mana1-6(Gala1-3(Fuca1-2)Galb1-3GlcNAcb1-2Mana1-3)Manb1-4GlcNAcb1-4GlcNAcb-Sp20	10	9	94
140	Galb1-3GalNAca-Sp14	10	10	104
244	Neu5Aca2-6(Neu5Aca2-3Galb1-3)GalNAca-Sp14	10	4	44
272	Neu5Aca2-6Galb1-4Glcb-Sp0	10	7	68
413	Gala1-3(Fuca1-2)Galb1-4GlcNAcb1-3GalNAca-Sp14	10	4	45
399	Neu5Aca2-3Galb1-3GlcNAcb1-3GalNAca-Sp14	10	4	39
195	Glca1-4Glcb-Sp8	10	9	96
606	Neu5Aca2-6Galb1-4GlcNAcb1-3Galb1-4GlcNAcb1-3Galb1-4GlcNAcb1-2Mana1-6(Neu5Aca2-6Galb1-4GlcNAcb1-3Galb1-4GlcNAcb1-3Galb1-4GlcNAcb1-2Mana1-3)Manb1-4GlcNAcb1-4GlcNAcb-Sp12	10	12	120
89	GalNAca1-3(Fuca1-2)Galb-Sp8	10	9	90
65	Fuca1-2Galb1-3GlcNAcb1-3Galb1-4Glcb-Sp8	10	6	58
43	(6S)Galb1-4Glcb-Sp8	9	4	43
248	Fuca1-2(6S)Galb1-4Glcb-Sp0	9	12	133
20	Galb1-4GlcNAcb1-6(Galb1-4GlcNAcb1-3)GalNAc-Sp14	9	15	158
87	GalNAca1-3(Fuca1-2)Galb1-4Glcb-Sp0	9	2	18
458	Galb1-4GlcNAcb1-6(Galb1-4GlcNAcb1-2)Mana1-6(Galb1-4GlcNAcb1-2Mana1-3)Manb1-4GlcNAcb1-4GlcNAcb-Sp19	9	5	55
202	GlcAb-Sp8	9	5	55
12	Galb-Sp8	9	8	87
3	Mana-Sp8	9	11	119
405	Gala1-3Galb1-4GlcNAcb1-3GalNAca-Sp14	9	20	213
361	Fuca1-2Galb1-4GlcNAcb1-2Mana1-6(Fuca1-2Galb1-4GlcNAcb1-2Mana1-3)Manb1-4GlcNAcb1-4GlcNAcb-Sp20	9	8	83
527	Gala1-3Galb1-3GlcNAcb1-2Mana-Sp0	9	2	27
283	Neu5Gca2-3Galb1-4GlcNAcb-Sp0	9	13	141
194	GlcNAcb1-6Galb1-4GlcNAcb-Sp8	9	5	53
431	GlcNAcb1-2Mana1-6(GlcNAcb1-4)(GlcNAcb1-2Mana1-3)Manb1-4GlcNAcb1-4GlcNAc-Sp21	9	8	83
537	Gala1-3(Fuca1-2)Galb1-3GalNAcb1-3Gala1-4Galb1-4Glc-Sp21	9	8	90
446	Fuca1-2Galb1-4(Fuca1-3)GlcNAcb1-2Mana1-6(Fuca1-2Galb1-4(Fuca1-3)GlcNAcb1-4(Fuca1-2Galb1-4(Fuca1-3)GlcNAcb1-2)Mana1-3)Manb1-4GlcNAcb1-4GlcNAcb-Sp12	9	3	37
249	Neu5Aca2-3Galb1-3GlcNAcb-Sp0	9	5	51
432	GlcNAcb1-2Mana1-6(GlcNAcb1-4)(GlcNAcb1-4(GlcNAcb1-2)Mana1-3)Manb1-4GlcNAcb1-4GlcNAc-Sp21	9	4	48
545	GlcNAcb1-3Galb1-4GlcNAcb1-3Galb1-4GlcNAcb1-2Mana1-6(GlcNAcb1-3Galb1-4GlcNAcb1-3Galb1-4GlcNAcb1-2Mana1-3)Manb1-4GlcNAcb1-4GlcNAcb-Sp12	9	4	40
142	Galb1-3GalNAcb-Sp8	9	7	81
246	Neu5Aca2-3Galb1-3GalNAcb1-3Gala1-4Galb1-4Glcb-Sp0	9	9	103
90	GalNAca1-3(Fuca1-2)Galb-Sp18	9	2	19
96	GalNAcb1-3Gala1-4Galb1-4GlcNAcb-Sp0	9	3	31
213	Mana1-6(Mana1-3)Mana-Sp9	9	2	27
592	Neu5Aca2-3Galb1-4GlcNAcb1-3Galb1-4GlcNAcb1-3GalNAca-Sp14	9	8	90
155	Galb1-4(6S)Glcb-Sp0	9	8	92
372	Gala1-3Galb1-4(Fuca1-3)GlcNAcb1-2Mana1-6(Gala1-3Galb1-4(Fuca1-3)GlcNAcb1-2Mana1-3)Manb1-4GlcNAcb1-4GlcNAcb-Sp20	9	5	58
123	Gala1-4GlcNAcb-Sp8	9	8	88
560	Gala1-3Galb1-4GlcNAcb1-2Mana1-6(Gala1-3Galb1-4GlcNAcb1-2Mana1-3)Manb1-4GlcNAcb1-4GlcNAc-Sp24	9	9	104
538	Galb1-3GalNAcb1-3Gal-Sp21	9	4	42
235	Neu5Aca2-6(Neu5Aca2-3)GalNAca-Sp8	9	6	71
389	Fuca1-2Galb1-3GalNAca1-3(Fuca1-2)Galb1-4Glcb-Sp0	9	5	61
191	GlcNAcb1-4GlcNAcb1-4GlcNAcb-Sp8	9	11	130
115	Gala1-3Galb1-4GlcNAcb-Sp8	9	4	45
598	GlcNAcb1-3Galb1-4GlcNAcb1-3Galb1-4GlcNAcb1-3GalNAca-Sp14	9	6	70
29	(3S)Galb1-3GalNAca-Sp8	9	10	121
156	Galb1-4(6S)Glcb-Sp8	9	4	47
225	GalNAcb1-4(Neu5Aca2-8Neu5Aca2-8Neu5Aca2-8Neu5Aca2-3)Galb1-4Glcb-Sp0	9	8	89
127	Galb1-3GlcNAcb1-3Galb1-4(Fuca1-3)GlcNAcb-Sp0	9	6	73
281	Neu5Gca2-3Galb1-3GlcNAcb-Sp0	8	8	95
591	Galb1-4GlcNAcb1-3Galb1-4GlcNAcb1-6(Galb1-4GlcNAcb1-3Galb1-4GlcNAcb1-3)GalNAca-Sp14	8	1	17
16	GlcNAcb-Sp0	8	3	40
13	Glcb-Sp8	8	8	100

200	G-ol-Sp8	22	5	21
201	GlcAa-Sp8	10	5	50
202	GlcAb-Sp8	9	5	55
203	GlcAb1-3Galb-Sp8	4	1	35
204	GlcAb1-6Galb-Sp8	5	5	94
205	KDNa2-3Galb1-3GlcNAcb-Sp0	10	7	72
206	KDNa2-3Galb1-4GlcNAcb-Sp0	12	13	110
207	Mana1-2Mana1-2Mana1-3Mana-Sp9	10	12	120
208	Mana1-2Mana1-6(Mana1-2Mana1-3)Mana-Sp9	7	5	72
209	Mana1-2Mana1-3Mana-Sp9	3	3	97
210	Mana1-6(Mana1-2Mana1-3)Mana1-6(Mana1-2Mana1-3)Manb1-4GlcNAcb1-4GlcNAcb-Sp12	6	9	155
211	Mana1-2Mana1-6(Mana1-3)Mana1-6(Mana1-2Mana1-2Mana1-3)Manb1-4GlcNAcb1-4GlcNAcb-Sp12	16	10	63
212	Mana1-2Mana1-6(Mana1-2Mana1-3)Mana1-6(Mana1-2Mana1-2Mana1-3)Manb1-4GlcNAcb1-4GlcNAcb-Sp12	-1	4	-291
213	Mana1-6(Mana1-3)Mana-Sp9	9	2	27
214	Mana1-2Mana1-2Mana1-6(Mana1-3)Mana-Sp9	6	4	67
215	Mana1-6(Mana1-3)Mana1-6(Mana1-2Mana1-3)Manb1-4GlcNAcb1-4GlcNAcb-Sp12	2	4	265
216	Mana1-6(Mana1-3)Mana1-6(Mana1-3)Manb1-4GlcNAcb1-4GlcNAcb-Sp12	8	7	93
217	Manb1-4GlcNAcb-Sp0	11	5	44
218	Neu5Aca2-3Galb1-4GlcNAcb1-3Galb1-4(Fuca1-3)GlcNAcb-Sp0	4	3	91
219	(3S)Galb1-4(Fuca1-3)(6S)GlcNAcb-Sp8	13	8	57
220	Fuca1-2(6S)Galb1-4GlcNAcb-Sp0	3	6	236
221	Fuca1-2Galb1-4(6S)GlcNAcb-Sp8	7	3	45
222	Fuca1-2(6S)Galb1-4(6S)Glcb-Sp0	10	6	64
223	Neu5Aca2-3Galb1-3GalNAca-Sp8	3	5	191
224	Neu5Aca2-3Galb1-3GalNAca-Sp14	1	2	163
225	GalNAcb1-4(Neu5Aca2-8Neu5Aca2-8Neu5Aca2-8Neu5Aca2-3)Galb1-4Glcb-Sp0	9	8	89
226	GalNAcb1-4(Neu5Aca2-8Neu5Aca2-8Neu5Aca2-3)Galb1-4Glcb-Sp0	1	5	408
227	Neu5Aca2-8Neu5Aca2-8Neu5Aca2-3Galb1-4Glcb-Sp0	6	6	106
228	GalNAcb1-4(Neu5Aca2-8Neu5Aca2-3)Galb1-4Glcb-Sp0	5	8	162
229	Neu5Aca2-8Neu5Aca2-8Neu5Aca-Sp8	8	4	49
230	Neu5Aca2-3(6S)Galb1-4(Fuca1-3)GlcNAcb-Sp8	-1	4	-276
231	GalNAcb1-4(Neu5Aca2-3)Galb1-4GlcNAcb-Sp0	8	6	67
232	GalNAcb1-4(Neu5Aca2-3)Galb1-4GlcNAcb-Sp8	0	1	419
233	GalNAcb1-4(Neu5Aca2-3)Galb1-4Glcb-Sp0	7	4	53
234	Neu5Aca2-3Galb1-3GalNAcb1-4(Neu5Aca2-3)Galb1-4Glcb-Sp0	1	4	298
235	Neu5Aca2-6(Neu5Aca2-3)GalNAca-Sp8	9	6	71
236	Neu5Aca2-3GalNAca-Sp8	4	7	164
237	Neu5Aca2-3GalNAcb1-4GlcNAcb-Sp0	6	4	70
238	Neu5Aca2-3Galb1-3(6S)GlcNAc-Sp8	8	11	138
239	Neu5Aca2-3Galb1-3(Fuca1-4)GlcNAcb-Sp8	4	6	146
240	Neu5Aca2-3Galb1-3(Fuca1-4)GlcNAcb1-3Galb1-4(Fuca1-3)GlcNAcb-Sp0	112	13	11
241	Neu5Aca2-3Galb1-4(Neu5Aca2-3Galb1-3)GlcNAcb-Sp8	6	6	100
242	Neu5Aca2-3Galb1-3(6S)GalNAca-Sp8	13	7	55
243	Neu5Aca2-6(Neu5Aca2-3Galb1-3)GalNAca-Sp8	0	3	-1972
244	Neu5Aca2-6(Neu5Aca2-3Galb1-3)GalNAca-Sp14	10	4	44
245	Neu5Aca2-3Galb-Sp8	3	5	197
246	Neu5Aca2-3Galb1-3GalNAcb1-3Gala1-4Galb1-4Glcb-Sp0	9	9	103
247	Neu5Aca2-3Galb1-3GlcNAcb1-3Galb1-4GlcNAcb-Sp0	-4	4	-100
248	Fuca1-2(6S)Galb1-4Glcb-Sp0	9	12	133
249	Neu5Aca2-3Galb1-3GlcNAcb-Sp0	9	5	51
250	Neu5Aca2-3Galb1-3GlcNAcb-Sp8	6	3	58
251	Neu5Aca2-3Galb1-4(6S)GlcNAcb-Sp8	3	2	68
252	Neu5Aca2-3Galb1-4(Fuca1-3)(6S)GlcNAcb-Sp8	-1	4	-544
253	Neu5Aca2-3Galb1-4(Fuca1-3)GlcNAcb1-3Galb1-4(Fuca1-3)GlcNAcb1-3Galb1-4(Fuca1-3)GlcNAcb-Sp0	2	2	82
254	Neu5Aca2-3Galb1-4(Fuca1-3)GlcNAcb-Sp0	1	2	138
255	Neu5Aca2-3Galb1-4(Fuca1-3)GlcNAcb-Sp8	4	7	170
256	Neu5Aca2-3Galb1-4(Fuca1-3)GlcNAcb1-3Galb-Sp8	2	5	234
257	Neu5Aca2-3Galb1-4(Fuca1-3)GlcNAcb1-3Galb1-4GlcNAcb-Sp8	5	3	47
258	Neu5Aca2-3Galb1-4GlcNAcb1-3Galb1-4GlcNAcb1-3Galb1-4GlcNAcb-Sp0	-5	7	-141
259	Neu5Aca2-3Galb1-4GlcNAcb-Sp0	15	6	44
260	Neu5Aca2-3Galb1-4GlcNAcb-Sp8	-5	12	-213
261	Neu5Aca2-3Galb1-4GlcNAcb1-3Galb1-4GlcNAcb-Sp0	16	12	74
262	Fuca1-2Galb1-4(6S)Glcb-Sp0	7	2	33
263	Neu5Aca2-3Galb1-4Glcb-Sp0	12	10	78
264	Neu5Aca2-3Galb1-4Glcb-Sp8	3	2	54
265	Neu5Aca2-6GalNAca-Sp8	19	13	66
266	Neu5Aca2-6GalNAcb1-4GlcNAcb-Sp0	6	2	39
267	Neu5Aca2-6Galb1-4(6S)GlcNAcb-Sp8	7	6	87
268	Neu5Aca2-6Galb1-4GlcNAcb-Sp0	5	6	113
269	Neu5Aca2-6Galb1-4GlcNAcb-Sp8	3	4	118
270	Neu5Aca2-6Galb1-4GlcNAcb1-3Galb1-4(Fuca1-3)GlcNAcb1-3Galb1-4(Fuca1-3)GlcNAcb-Sp0	6	10	164
271	Neu5Aca2-6Galb1-4GlcNAcb1-3Galb1-4GlcNAcb-Sp0	8	8	102
272	Neu5Aca2-6Galb1-4Glcb-Sp0	10	7	68
273	Neu5Aca2-6Galb1-4Glcb-Sp8	0	2	-421
274	Neu5Aca2-6Galb-Sp8	11	10	93
275	Neu5Aca2-8Neu5Aca-Sp8	4	5	144
276	Neu5Aca2-8Neu5Aca2-3Galb1-4Glcb-Sp0	1	3	240
277	Galb1-3(Fuca1-4)GlcNAcb1-3Galb1-3(Fuca1-4)GlcNAcb-Sp0	16	10	64
278	Neu5Acb2-6GalNAca-Sp8	7	6	80
279	Neu5Acb2-6Galb1-4GlcNAcb-Sp8	2	4	209
280	Neu5Gca2-3Galb1-3(Fuca1-4)GlcNAcb-Sp0	6	7	123
281	Neu5Gca2-3Galb1-3GlcNAcb-Sp0	8	8	95
282	Neu5Gca2-3Galb1-4(Fuca1-3)GlcNAcb-Sp0	-5	8	-142
283	Neu5Gca2-3Galb1-4GlcNAcb-Sp0	9	13	141
284	Neu5Gca2-3Galb1-4Glcb-Sp0	-3	8	-224
285	Neu5Gca2-6GalNAca-Sp0	2	7	365
286	Neu5Gca2-6Galb1-4GlcNAcb-Sp0	12	2	16
287	Neu5Gca-Sp8	2	3	153
288	Neu5Aca2-3Galb1-4GlcNAcb1-6(Galb1-3)GalNAca-Sp14	5	4	76
289	Galb1-3GlcNAcb1-3Galb1-3GlcNAcb-Sp0	2	4	173
290	Galb1-4(Fuca1-3)(6S)GlcNAcb-Sp0	7	5	74
291	Galb1-4(Fuca1-3)(6S)Glcb-Sp0	5	7	135
292	Galb1-4(Fuca1-3)GlcNAcb1-3Galb1-3(Fuca1-4)GlcNAcb-Sp0	-2	2	-82
293	Galb1-4GlcNAcb1-3Galb1-3GlcNAcb-Sp0	8	3	39
294	Neu5Aca2-3Galb1-3GlcNAcb1-3Galb1-3GlcNAcb-Sp0	8	4	43
295	Neu5Aca2-3Galb1-4GlcNAcb1-3Galb1-3GlcNAcb-Sp0	3	2	66
296	4S(3S)Galb1-4GlcNAcb-Sp0	-5	3	-61
297	(6S)Galb1-4(6S)GlcNAcb-Sp0	5	3	48
298	(6P)Glcb-Sp10	6	8	134
299	Neu5Aca2-3Galb1-4(Fuca1-3)GlcNAcb1-6(Galb1-3)GalNAca-Sp14	13	8	65
300	Galb1-3Galb1-4GlcNAcb-Sp8	4	4	103
301	Neu5Aca2-6Galb1-4GlcNAcb1-2Mana1-6(Galb1-4GlcNAcb1-2Mana1-3)Manb1-4GlcNAcb1-4GlcNAcb-Sp12	4	5	108
302	Galb1-4GlcNAcb1-6(Galb1-4GlcNAcb1-3)Galb1-4GlcNAc-Sp0	-1	3	-243
303	GlcNAcb1-6(Galb1-4GlcNAcb1-3)Galb1-4GlcNAc-Sp0	5	4	81

231	GalNAcb1-4(Neu5Aca2-3)Galb1-4GlcNAcb-Sp0	8	6	67
24	(3S)Galb1-4(Fuca1-3)(6S)Glc-Sp0	8	4	43
584	Galb1-4GlcNAcb1-3Galb1-4GlcNAcb1-3Galb1-4GlcNAcb1-6(Galb1-4GlcNAcb1-3Galb1-4GlcNAcb1-3Galb1-4GlcNAb1-2)Mana1-6(Galb1-4GlcNAcb1-3Galb1-4GlcNAcb1-3Galb1-4GlcNAcb1-2Mana1-3)Manb1-4GlcNAcb1-4(Fuca1-6)GlcNAcb-Sp24	8	8	99
463	Neu5Aca2-6Galb1-4GlcNAcb1-2Mana1-6(GlcNAcb1-4)(Neu5Aca2-6Galb1-4GlcNAcb1-2Mana1-3)Manb1-4GlcNAcb1-4GlcNAcb-Sp21	8	5	58
555	Neu5Gca2-8Neu5Gca2-3Galb1-4GlcNAcb1-3Galb1-4GlcNAc-Sp0	8	11	131
88	GlcNAcb1-3Galb1-3GalNAca-Sp8	8	5	60
94	GalNAcb1-3GalNAca-Sp8	8	11	133
294	Neu5Aca2-3Galb1-3GlcNAcb1-3Galb1-3GlcNAcb-Sp0	8	4	43
181	GlcNAcb1-3Galb-Sp8	8	13	162
72	Fuca1-2Galb1-4(Fuca1-3)GlcNAcb-Sp8	8	4	43
579	Galb1-4GlcNAcb1-3Galb1-4GlcNAcb1-3Galb1-4GlcNAcb1-3Galb1-4GlcNAcb1-2Mana1-6(Galb1-4GlcNAcb1-3Galb1-4GlcNAcb1-3Galb1-4GlcNAcb1-3Galb1-4GlcNAcb1-2Mana1-3)Manb1-4GlcNAcb1-4(Fuca1-6)GlcNAcb-Sp24	8	5	60
387	Galb1-4GlcNAcb1-6(Galb1-4GlcNAcb1-2)Mana1-6(Galb1-4GlcNAcb1-4(Galb1-4GlcNAcb1-2)Mana1-3)Manb1-4GlcNAcb1-4GlcNAcb-Sp21	8	3	38
149	Galb1-3GlcNAcb-Sp0	8	8	100
128	Galb1-3(Fuca1-4)GlcNAc-Sp0	8	7	84
462	Neu5Aca2-3Galb1-4GlcNAcb1-6(Neu5Aca2-3Galb1-4GlcNAcb1-2)Mana1-6(GlcNAcb1-4)(Neu5Aca2-3Galb1-4GlcNAcb1-4(Neu5Aca2-3Galb1-4GlcNAcb1-2)Mana1-3)Manb1-4GlcNAcb1-4GlcNAcb-Sp21	8	8	98
429	Galb1-4GlcNAcb1-6(Fuca1-2Galb1-3GlcNAcb1-3)Galb1-4Glc-Sp21	8	5	59
293	Galb1-4GlcNAcb1-3Galb1-3GlcNAcb-Sp0	8	3	39
456	Neu5Aca2-6Galb1-4GlcNAcb1-6(Fuca1-2Galb1-3GlcNAcb1-3)Galb1-4Glc-Sp21	8	7	86
109	Gala1-4(Gala1-3)Galb1-4GlcNAcb-Sp8	8	7	91
425	Gala1-3Galb1-3GlcNAcb1-3GalNAc-Sp14	8	5	66
600	Neu5Aca2-3Galb1-4GlcNAcb1-3Galb1-4GlcNAcb1-6(Galb1-3)GalNAca-Sp14	8	9	111
319	Galb1-4GlcNAcb1-2Mana1-6(Neu5Aca2-6Galb1-4GlcNAcb1-2Mana1-3)Manb1-4GlcNAcb1-4GlcNAcb-Sp12	8	8	105
216	Mana1-6(Mana1-3)Mana1-6(Mana1-3)Manb1-4GlcNAcb1-4GlcNAcb-Sp12	8	7	93
190	GlcNAcb1-4GlcNAcb1-4GlcNAcb1-4GlcNAcb1-4GlcNAcb1-Sp8	8	9	112
164	Galb1-4GlcNAcb1-3Galb1-4Glcb-Sp0	8	2	26
199	Glcb1-6Glcb-Sp8	8	10	124
15	GalNAcb-Sp8	8	6	76
443	(6S)Galb1-3GlcNAcb-Sp0	8	5	57
480	Neu5Aca2-6Galb1-4GlcNAcb1-6GalNAca-Sp14	8	12	149
530	GlcNAcb1-2 Mana1-6(GlcNAcb1-4)(GlcNAcb1-2Mana1-3)Manb1-4GlcNAcb1-4(Fuca1-6)GlcNAc-Sp21	8	3	34
229	Neu5Aca2-8Neu5Aca2-8Neu5Aca-Sp8	8	4	49
605	Neu5Aca2-6Galb1-4GlcNAcb1-3Galb1-4GlcNAcb1-6(Neu5Aca2-6Galb1-4GlcNAcb1-3Galb1-4GlcNAcb1-3)GalNAca-Sp14	8	5	68
271	Neu5Aca2-6Galb1-4GlcNAcb1-3Galb1-4GlcNAcb-Sp0	8	8	102
344	GlcNAca1-4Galb1-3GalNAc-Sp14	8	7	95
544	Fuca1-2Galb1-4GlcNAcb1-3Galb1-4GlcNAcb1-2Mana1-6(Fuca1-2Galb1-4GlcNAcb1-3Galb1-4GlcNAcb1-2Mana1-3)Manb1-4GlcNAcb1-4GlcNAcb-Sp24	8	9	120
144	Galb1-3GalNAcb1-4(Neu5Aca2-3)Galb1-4Glcb-Sp0	8	4	56
95	GalNAcb1-3(Fuca1-2)Galb-Sp8	8	2	24
238	Neu5Aca2-3Galb1-3(6S)GlcNAc-Sp8	8	11	138
118	Gala1-3Galb-Sp8	8	8	101
410	Galb1-4GlcNAcb1-6(Neu5Aca2-6Galb1-3GlcNAcb1-3)Galb1-4Glc-Sp21	8	8	105
32	(3S)Galb1-4(Fuca1-3)GlcNAc-Sp0	8	7	92
363	Gala1-3Galb1-4GlcNAcb1-2Mana1-6(Gala1-3Galb1-4GlcNAcb1-2Mana1-3)Manb1-4GlcNAcb1-4GlcNAcb-Sp20	8	6	77
572	Galb1-3GlcNAcb1-3Galb1-4GlcNAcb1-6(Galb1-3GlcNAcb1-3Galb1-4GlcNAb1-2)Mana1-6(Galb1-3GlcNAcb1-3Galb1-4GlcNAcb1-2Mana1-3)Manb1-4GlcNAcb1-4(Fuca1-6)GlcNAcb-Sp24	8	8	109
178	GlcNAcb1-6(GlcNAcb1-3)Galb1-4GlcNAcb-Sp8	8	6	82
477	Neu5Aca2-6Galb1-4GlcNAcb1-6(Galb1-3GlcNAcb1-3)Galb1-4Glcb-Sp21	8	6	85
221	Fuca1-2Galb1-4(6S)GlcNAcb-Sp8	7	3	45
31	(3S)Galb1-3GlcNAcb-Sp8	7	8	104
290	Galb1-4(Fuca1-3)(6S)GlcNAcb-Sp0	7	5	74
479	Neu5Aca2-3Galb1-4GlcNAcb1-6GalNAca-Sp14	7	8	115
208	Mana1-2Mana1-6(Mana1-2Mana1-3)Mana-Sp9	7	5	72
473	Fuca1-2Galb1-3(Fuca1-4)GlcNAcb1-2Mana1-6(Fuca1-2Galb1-3(Fuca1-4)GlcNAcb1-2Mana1-3)Manb1-4GlcNAcb1-4(Fuca1-6)GlcNAcb1-4(Fuca1-6)GlcNAcb-Sp19	7	7	99
346	Mana1-6(Neu5Aca2-6Galb1-4GlcNAcb1-2Mana1-3)Manb1-4GlcNAcb1-4GlcNAc-Sp12	7	3	41
317	Neu5Aca2-3Galb1-4GlcNAcb1-6(Neu5Aca2-3Galb1-3)GalNAca-Sp14	7	7	95
60	Fuca1-2Galb1-3(Fuca1-4)GlcNAcb-Sp8	7	6	89
557	Neu5Aca2-8Neu5Aca2-3Galb1-4GlcNAc-Sp0	7	9	124
426	Fuca1-2Galb1-3GlcNAcb1-2Mana1-6(Fuca1-2Galb1-3GlcNAcb1-2Mana1-3)Manb1-4GlcNAcb1-4(Fuca1-6)GlcNAcb-Sp22	7	3	45
146	Galb1-3Galb-Sp8	7	9	126
316	Mana1-2Mana1-6(Mana1-2Mana1-3)Mana1-6(Mana1-2Mana1-2Mana1-3)Mana-Sp9	7	1	13
393	GalNAca1-3(Fuca1-2)Galb1-3GalNAca1-3(Fuca1-2)Galb1-4GlcNAcb-Sp0	7	6	89
337	Neu5Aca2-3Galb1-4(Fuca1-3)GlcNAcb1-6(Neu5Aca2-3Galb1-3)GalNAc-Sp14	7	2	27
492	(3S)Galb1-3(Fuca1-4)GlcNAcb-Sp0	7	8	105
54	Galb1-4GlcNAcb1-2Mana1-6(Galb1-4GlcNAcb1-2Mana1-3)Manb1-4GlcNAcb1-4GlcNAcb-Sp12	7	5	71
564	GlcNAcb1-3Galb1-4GlcNAcb1-3Galb1-4GlcNAcb1-3Galb1-4GlcNAcb1-3Galb1-4GlcNAcb1-2Mana1-6(GlcNAcb1-3Galb1-4GlcNAcb1-3Galb1-4GlcNAcb1-3Galb1-4GlcNAcb1-3Galb1-4GlcNAcb1-2Mana1-3)Manb1-4GlcNAcb1-4GlcNAcb-Sp25	7	2	27
173	GlcNAca1-3Galb1-4GlcNAcb-Sp8	7	4	53
411	Galb1-3GalNAcb1-4(Neu5Aca2-8Neu5Aca2-3)Galb1-4Glcb-Sp0	7	6	90
143	Galb1-3GalNAcb1-3Gala1-4Galb1-4Glcb-Sp0	7	5	65
428	Galb1-3GlcNAcb1-6(Galb1-3GlcNAcb1-2)Mana1-6(Galb1-3GlcNAcb1-2Mana1-3)Manb1-4GlcNAcb1-4GlcNAcb-Sp19	7	4	56
233	GalNAcb1-4(Neu5Aca2-3)Galb1-4Glcb-Sp0	7	4	53
394	Gala1-3Galb1-3GlcNAcb1-2Mana1-6(Gala1-3Galb1-3GlcNAcb1-2Mana1-3)Manb1-4GlcNAcb1-4GlcNAc-Sp19	7	5	67
135	Neu5Aca2-6(Galb1-3)GalNAca-Sp8	7	2	30
471	Neu5Aca2-3Galb1-4GlcNAcb1-6(Neu5Aca2-3Galb1-4GlcNAcb1-3)GalNAca-Sp14	7	6	84
362	Fuca1-2Galb1-4(Fuca1-3)GlcNAcb1-2Mana1-6(Fuca1-2Galb1-4(Fuca1-3)GlcNAcb1-2Mana1-3)Manb1-4GlcNAcb1-4GlcNAb-Sp20	7	9	122
554	Neu5Gca2-8Neu5Aca2-3Galb1-4GlcNAc-Sp0	7	8	114
435	Galb1-4GlcNAcb1-2Mana1-6(GlcNAcb1-4)(Galb1-4GlcNAcb1-2Mana1-3)Manb1-4GlcNAcb1-4GlcNAc-Sp21	7	7	94
388	GlcNAcb1-2Mana1-6(GlcNAcb1-4(GlcNAcb1-2)Mana1-3)Manb1-4GlcNAcb1-4GlcNAc-Sp21	7	10	139
278	Neu5Acb2-6GalNAca-Sp8	7	6	80
520	Neu5Aca2-6Galb1-4GlcNAcb1-2Man-Sp0	7	3	40
161	Galb1-4GlcNAcb1-3Galb1-4(Fuca1-3)GlcNAcb1-3Galb1-4(Fuca1-3)GlcNAcb-Sp0	7	5	77
267	Neu5Aca2-6Galb1-4(6S)GlcNAcb-Sp8	7	6	87
111	Gala1-3GalNAca-Sp16	7	6	83
61	Fuca1-2Galb1-3GalNAca-Sp8	7	1	18
359	KDNa2-3Galb1-3GalNAca-Sp14	7	7	100
549	GlcNAcb1-3Galb1-4GlcNAcb1-3Galb1-4GlcNAcb1-3Galb1-4GlcNAcb1-2Mana1-6(GlcNAcb1-3Galb1-4GlcNAcb1-3Galb1-4GlcNAcb1-3Galb1-4GlcNAcb1-2Mana1-3)Manb1-4GlcNAcb1-4GlcNAcb-Sp25	7	5	73
485	Galb1-4GlcNAcb1-6(Galb1-4GlcNAcb1-2)Mana1-6(Galb1-4GlcNAcb1-2Mana1-3)Manb1-4GlcNAcb1-4(Fuca1-6)GlcNAcb-Sp24	7	5	73
467	Gala1-3(Fuca1-2)Galb1-3GalNAca-Sp8	7	5	73
438	Galb1-4GlcNAcb1-6(Galb1-4GlcNAcb1-2)Mana1-6(GlcNAcb1-4)(Galb1-4GlcNAcb1-4(Galb1-4GlcNAcb1-2)Mana1-3)Manb1-4GlcNAcb1-4GlcNAc-Sp21	7	4	54
329	Neu5,9Ac2a2-3Galb1-3GlcNAcb-Sp0	7	11	166
262	Fuca1-2Galb1-4(6S)Glcb-Sp0	7	2	33
566	GlcNAb1-3Galb1-3GalNAc-Sp14	7	1	13
454	GalNAca1-3(Fuca1-2)Galb1-4GlcNAcb1-2Mana1-6(GalNAca1-3(Fuca1-2)Galb1-4GlcNAcb1-2Mana1-3)Manb1-4GlcNAcb1-4(Fuca1-6)GlcNAcb-Sp22	7	7	101
552	Neu5Gca2-8Neu5Gca2-3Galb1-4GlcNAc-Sp0	7	6	84
367	Neu5Aca2-6GlcNAcb1-4GlcNAcb1-4GlcNAc-Sp21	7	6	91
522	Gala1-3(Fuca1-2)Galb1-4GlcNAcb1-2Mana-Sp0	7	4	55
481	Neu5Aca2-6Galb1-4 GlcNAcb1-6(Neu5Aca2-6Galb1-4GlcNAcb1-3)GalNAca-Sp14	6	7	104
125	Galb1-2Galb-Sp8	6	6	86
433	GlcNAcb1-6(GlcNAcb1-2)Mana1-6(GlcNAcb1-4)(GlcNAcb1-2Mana1-3)Manb1-4GlcNAcb1-4GlcNAc-Sp21	6	6	97
504	(3S)GalNAcb1-4(Fuca1-3)GlcNAcb-Sp8	6	3	49
138	Neu5Aca2-6(Galb1-3)GlcNAcb1-4Galb1-4Glcb-Sp10	6	9	134
75	Fuca1-2Galb1-4GlcNAcb-Sp0	6	2	34
62	Fuca1-2Galb1-3GalNAca-Sp14	6	2	25
237	Neu5Aca2-3GalNAcb1-4GlcNAcb-Sp0	6	4	70
384	Galb1-4GlcNAcb1-6(Fuca1-4(Fuca1-2Galb1-3)GlcNAcb1-3)Galb1-4Glc-Sp21	6	8	131
524	Galb1-3GlcNAcb1-2Mana-Sp0	6	9	145
214	Mana1-2Mana1-2Mana1-6(Mana1-3)Mana-Sp9	6	4	67

304	Galb1-4GlcNAca1-6Galb1-4GlcNAcb-Sp0	-1	4	-596
305	Galb1-4GlcNAcb1-6Galb1-4GlcNAcb-Sp0	-1	8	-1168
306	GalNAcb1-3Galb-Sp8	3	3	113
307	GlcAb1-3GlcNAcb-Sp8	5	6	132
308	Neu5Aca2-6Galb1-4GlcNAcb1-2Mana1-6(GlcNAcb1-2Mana1-3)Manb1-4GlcNAcb1-4GlcNAcb-Sp12	6	5	77
309	GlcNAcb1-3Man-Sp10	3	6	180
310	GlcNAcb1-4GlcNAcb-Sp10	12	5	39
311	GlcNAcb1-4GlcNAcb-Sp12	2	5	203
312	MurNAcb1-4GlcNAcb-Sp10	-1	6	-959
313	Mana1-6Manb-Sp10	4	5	119
314	Mana1-6(Mana1-3)Mana1-6(Mana1-3)Manb-Sp10	3	5	184
315	Mana1-2Mana1-6(Mana1-3)Mana1-6(Mana1-2Mana1-2Mana1-3)Mana-Sp9	0	4	-1522
316	Mana1-2Mana1-6(Mana1-2Mana1-3)Mana1-6(Mana1-2Mana1-2Mana1-3)Mana-Sp9	7	1	13
317	Neu5Aca2-3Galb1-4GlcNAcb1-6(Neu5Aca2-3Galb1-3)GalNAca-Sp14	7	7	95
318	Neu5Aca2-6Galb1-4GlcNAcb1-2Mana1-6(Neu5Aca2-3Galb1-4GlcNAcb1-2Mana1-3)Manb1-4GlcNAcb1-4GlcNAcb-Sp12	32	20	64
319	Galb1-4GlcNAcb1-2Mana1-6(Neu5Aca2-6Galb1-4GlcNAcb1-2Mana1-3)Manb1-4GlcNAcb1-4GlcNAcb-Sp12	8	8	105
320	GlcNAcb1-2Mana1-6(Neu5Aca2-6Galb1-4GlcNAcb1-2Mana1-3)Manb1-4GlcNAcb1-4GlcNAcb-Sp12	3	6	205
321	Neu5Aca2-8Neu5Acb-Sp17	5	6	135
322	Neu5Aca2-8Neu5Aca2-8Neu5Acb-Sp8	0	6	-4791
323	Neu5Gcb2-6Galb1-4GlcNAc-Sp8	6	6	111
324	Galb1-3GlcNAcb1-2Mana1-6(Galb1-3GlcNAcb1-2Mana1-3)Manb1-4GlcNAcb1-4GlcNAcb-Sp19	5	5	98
325	Neu5Aca2-3Galb1-4GlcNAcb1-2Mana1-6(Neu5Aca2-3Galb1-4GlcNAcb1-2Mana1-3)Manb1-4GlcNAcb1-4GlcNAcb-Sp12	10	10	97
326	Neu5Aca2-3Galb1-4GlcNAcb1-2Mana1-6(Neu5Aca2-6Galb1-4GlcNAcb1-2Mana1-3)Manb1-4GlcNAcb1-4GlcNAcb-Sp12	1	7	496
327	Galb1-4(Fuca1-3)GlcNAcb1-2Mana1-6(Galb1-4(Fuca1-3)GlcNAcb1-2Mana1-3)Manb1-4GlcNAcb1-4GlcNAcb-Sp20	0	4	-970
328	Neu5,9Ac2a2-3Galb1-4GlcNAcb-Sp0	23	10	42
329	Neu5,9Ac2a2-3Galb1-3GlcNAcb-Sp0	7	11	166
330	Neu5Aca2-6Galb1-4GlcNAcb1-3Galb1-3GlcNAcb-Sp0	13	10	76
331	Neu5Aca2-3Galb1-3(Fuca1-4)GlcNAcb1-3Galb1-3(Fuca1-4)GlcNAcb-Sp0	43	15	34
332	Neu5Aca2-6Galb1-4GlcNAcb1-3Galb1-4GlcNAcb1-3Galb1-4GlcNAcb-Sp0	3	5	137
333	Gala1-4Galb1-4GlcNAcb1-3Galb1-4Glcb-Sp0	0	3	-812
334	GalNAcb1-3Gala1-4Galb1-4GlcNAcb1-3Galb1-4Glcb-Sp0	11	9	76
335	GalNAca1-3(Fuca1-2)Galb1-4GlcNAcb1-3Galb1-4GlcNAcb-Sp0	19	13	71
336	GalNAca1-3(Fuca1-2)Galb1-4GlcNAcb1-3Galb1-4GlcNAcb1-3Galb1-4GlcNAcb-Sp0	14	5	39
337	Neu5Aca2-3Galb1-4(Fuca1-3)GlcNAcb1-6(Neu5Aca2-3Galb1-3)GalNAc-Sp14	7	2	27
338	GlcNAca1-4Galb1-4GlcNAcb1-3Galb1-4GlcNAcb1-3Galb1-4GlcNAcb-Sp0	4	6	162
339	GlcNAca1-4Galb1-4GlcNAcb-Sp0	1	2	199
340	GlcNAca1-4Galb1-3GlcNAcb-Sp0	3	2	59
341	GlcNAca1-4Galb1-4GlcNAcb1-3Galb1-4Glcb-Sp0	12	6	51
342	GlcNAca1-4Galb1-4GlcNAcb1-3Galb1-4(Fuca1-3)GlcNAcb1-3Galb1-4(Fuca1-3)GlcNAcb-Sp0	13	11	86
343	GlcNAca1-4Galb1-4GlcNAcb1-3Galb1-4GlcNAcb-Sp0	4	3	70
344	GlcNAca1-4Galb1-3GalNAc-Sp14	8	7	95
345	Neu5Aca2-6Galb1-4GlcNAcb1-2Mana1-6(Mana1-3)Manb1-4GlcNAcb1-4GlcNAc-Sp12	-1	3	-254
346	Mana1-6(Neu5Aca2-6Galb1-4GlcNAcb1-2Mana1-3)Manb1-4GlcNAcb1-4GlcNAc-Sp12	7	3	41
347	Neu5Aca2-6Galb1-4GlcNAcb1-2Mana1-6Manb1-4GlcNAcb1-4GlcNAc-Sp12	6	6	110
348	Neu5Aca2-6Galb1-4GlcNAcb1-2Mana1-3Manb1-4GlcNAcb1-4GlcNAc-Sp12	6	1	18
349	Galb1-4GlcNAcb1-2Mana1-3Manb1-4GlcNAcb1-4GlcNAc-Sp12	4	3	84
350	Galb1-4GlcNAcb1-2Mana1-6Manb1-4GlcNAcb1-4GlcNAc-Sp12	3	7	240
351	Mana1-6(Galb1-4GlcNAcb1-2Mana1-3)Manb1-4GlcNAcb1-4GlcNAcb-Sp12	20	20	103
352	GlcNAcb1-2Mana1-6(GlcNAcb1-2Mana1-3)Manb1-4GlcNAcb1-4(Fuca1-6)GlcNAcb-Sp22	3	2	83
353	Galb1-4GlcNAcb1-2Mana1-6(Galb1-4GlcNAcb1-2Mana1-3)Manb1-4GlcNAcb1-4(Fuca1-6)GlcNAcb-Sp22	19	9	49
354	Galb1-3GlcNAcb1-2Mana1-6(Galb1-3GlcNAcb1-2Mana1-3)Manb1-4GlcNAcb1-4(Fuca1-6)GlcNAcb-Sp22	4	12	279
355	(6S)GlcNAcb1-3Galb1-4GlcNAcb-Sp0	4	4	100
356	KDNa2-3Galb1-4(Fuca1-3)GlcNAc-Sp0	-1	3	-291
357	KDNa2-6Galb1-4GlcNAc-Sp0	6	2	40
358	KDNa2-3Galb1-4Glc-Sp0	5	6	117
359	KDNa2-3Galb1-3GalNAca-Sp14	7	7	100
360	Fuca1-2Galb1-3GlcNAcb1-2Mana1-6(Fuca1-2Galb1-3GlcNAcb1-2Mana1-3)Manb1-4GlcNAcb1-4GlcNAcb-Sp20	4	5	137
361	Fuca1-2Galb1-4GlcNAcb1-2Mana1-6(Fuca1-2Galb1-4GlcNAcb1-2Mana1-3)Manb1-4GlcNAcb1-4GlcNAcb-Sp20	9	8	83
362	Fuca1-2Galb1-4(Fuca1-3)GlcNAcb1-2Mana1-6(Fuca1-2Galb1-4(Fuca1-3)GlcNAcb1-2Mana1-3)Manb1-4GlcNAcb1-4GlcNAb-Sp20	7	9	122
363	Gala1-3Galb1-4GlcNAcb1-2Mana1-6(Gala1-3Galb1-4GlcNAcb1-2Mana1-3)Manb1-4GlcNAcb1-4GlcNAcb-Sp20	8	6	77
364	Galb1-4GlcNAcb1-2Mana1-6(Mana1-3)Manb1-4GlcNAcb1-4GlcNAcb-Sp12	2	6	343
365	Fuca1-4(Galb1-3)GlcNAcb1-2Mana1-6(Fuca1-4(Galb1-3)GlcNAcb1-2Mana1-3)Manb1-4GlcNAcb1-4(Fuca1-6)GlcNAcb-Sp22	13	18	144
366	Neu5Aca2-6GlcNAcb1-4GlcNAc-Sp21	3	7	197
367	Neu5Aca2-6GlcNAcb1-4GlcNAcb1-4GlcNAc-Sp21	7	6	91
368	Galb1-4(Fuca1-3)GlcNAcb1-6(Fuca1-2Galb1-4GlcNAcb1-3)Galb1-4Glc-Sp21	2	7	292
369	Galb1-4GlcNAcb1-2Mana1-6(Galb1-4GlcNAcb1-4(Galb1-4GlcNAcb1-2)Mana1-3)Manb1-4GlcNAcb1-4GlcNAc-Sp21	2	6	285
370	GalNAca1-3(Fuca1-2)Galb1-4GlcNAcb1-2Mana1-6(GalNAca1-3(Fuca1-2)Galb1-4GlcNAcb1-2Mana1-3)Manb1-4GlcNAcb1-4GlcNAcb-Sp20	11	4	40
371	Gala1-3(Fuca1-2)Galb1-4GlcNAcb1-2Mana1-6(Gala1-3(Fuca1-2)Galb1-4GlcNAcb1-2Mana1-3)Manb1-4GlcNAcb1-4GlcNAcb-Sp20	10	5	46
372	Gala1-3Galb1-4(Fuca1-3)GlcNAcb1-2Mana1-6(Gala1-3Galb1-4(Fuca1-3)GlcNAcb1-2Mana1-3)Manb1-4GlcNAcb1-4GlcNAcb-Sp20	9	5	58
373	GalNAca1-3(Fuca1-2)Galb1-3GlcNAcb1-2Mana1-6(GalNAca1-3(Fuca1-2)Galb1-3GlcNAcb1-2Mana1-3)Manb1-4GlcNAcb1-4GlcNAcb-Sp20	3	12	343
374	Gala1-3(Fuca1-2)Galb1-3GlcNAcb1-2Mana1-6(Gala1-3(Fuca1-2)Galb1-3GlcNAcb1-2Mana1-3)Manb1-4GlcNAcb1-4GlcNAcb-Sp20	10	9	94
375	Fuca1-4(Fuca1-2Galb1-3)GlcNAcb1-2Mana1-3(Fuca1-4(Fuca1-2Galb1-3)GlcNAcb1-2Mana1-3)Manb1-4GlcNAcb1-4GlcNAcb-Sp19	4	4	106
376	Neu5Aca2-3Galb1-4GlcNAcb1-3GalNAc-Sp14	6	3	42
377	Neu5Aca2-6Galb1-4GlcNAcb1-3GalNAc-Sp14	5	7	134
378	Neu5Aca2-3Galb1-4(Fuca1-3)GlcNAcb1-3GalNAca-Sp14	5	5	100
379	GalNAcb1-4GlcNAcb1-2Mana1-6(GalNAcb1-4GlcNAcb1-2Mana1-3)Manb1-4GlcNAcb1-4GlcNAc-Sp12	4	1	31
380	Galb1-3GalNAca1-3(Fuca1-2)Galb1-4Glc-Sp0	11	7	65
381	Galb1-3GalNAca1-3(Fuca1-2)Galb1-4GlcNAc-Sp0	1	3	560
382	Galb1-3GlcNAcb1-3Galb1-4GlcNAcb1-6(Galb1-3GlcNAcb1-3)Galb1-4Glcb-Sp0	14	5	35
383	Galb1-4(Fuca1-3)GlcNAcb1-6(Galb1-3GlcNAcb1-3)Galb1-4Glc-Sp21	3	13	531
384	Galb1-4GlcNAcb1-6(Fuca1-4(Fuca1-2Galb1-3)GlcNAcb1-3)Galb1-4Glc-Sp21	6	8	131
385	Galb1-4(Fuca1-3)GlcNAcb1-6(Fuca1-4(Fuca1-2Galb1-3)GlcNAcb1-3)Galb1-4Glc-Sp21	20	8	40
386	Galb1-3GlcNAcb1-3Galb1-4(Fuca1-3)GlcNAcb1-6(Galb1-3GlcNAcb1-3)Galb1-4Glc-Sp21	13	16	123
387	Galb1-4GlcNAcb1-6(Galb1-4GlcNAcb1-2)Mana1-6(Galb1-4GlcNAcb1-4(Galb1-4GlcNAcb1-2)Mana1-3)Manb1-4GlcNAcb1-4GlcNAcb-Sp21	8	3	38
388	GlcNAcb1-2Mana1-6(GlcNAcb1-4(GlcNAcb1-2)Mana1-3)Manb1-4GlcNAcb1-4GlcNAc-Sp21	7	10	139
389	Fuca1-2Galb1-3GalNAca1-3(Fuca1-2)Galb1-4Glcb-Sp0	9	5	61
390	Fuca1-2Galb1-3GalNAca1-3(Fuca1-2)Galb1-4GlcNAcb-Sp0	2	6	344
391	Galb1-3GlcNAcb1-3GalNAca-Sp14	12	2	14
392	GalNAcb1-4(Neu5Aca2-3)Galb1-4GlcNAcb1-3GalNAca-Sp14	3	2	67
393	GalNAca1-3(Fuca1-2)Galb1-3GalNAca1-3(Fuca1-2)Galb1-4GlcNAcb-Sp0	7	6	89
394	Gala1-3Galb1-3GlcNAcb1-2Mana1-6(Gala1-3Galb1-3GlcNAcb1-2Mana1-3)Manb1-4GlcNAcb1-4GlcNAc-Sp19	7	5	67
395	Gala1-3Galb1-3(Fuca1-4)GlcNAcb1-2Mana1-6(Gala1-3Galb1-3(Fuca1-4)GlcNAcb1-2Mana1-3)Manb1-4GlcNAcb1-4GlcNAc-Sp19	4	7	190
396	Neu5Aca2-3Galb1-3GlcNAcb1-2Mana1-6(Neu5Aca2-3Galb1-3GlcNAcb1-2Mana1-3)Manb1-4GlcNAcb1-4GlcNAc-Sp19	6	2	30
397	GlcNAcb1-2Mana1-6(Galb1-4GlcNAcb1-2Mana1-3)Manb1-4GlcNAcb1-4GlcNAc-Sp12	3	6	219
398	Galb1-4GlcNAcb1-2Mana1-6(GlcNAcb1-2Mana1-3)Manb1-4GlcNAcb1-4GlcNAc-Sp12	15	10	63
399	Neu5Aca2-3Galb1-3GlcNAcb1-3GalNAca-Sp14	10	4	39
400	Fuca1-2Galb1-4GlcNAcb1-3GalNAca-Sp14	5	2	35
401	Galb1-4(Fuca1-3)GlcNAcb1-3GalNAca-Sp14	0	5	-1667

71	Fuca1-2Galb1-4(Fuca1-3)GlcNAcb-Sp0	6	4	63
459	Neu5Aca2-3Galb1-4GlcNAcb1-2Mana1-6(GlcNAcb1-4)(Neu5Aca2-3Galb1-4GlcNAcb1-2Mana1-3)Manb1-4GlcNAcb1-4GlcNAcb-Sp21	6	3	46
608	Neu5Aca2-6Galb1-4GlcNAcb1-3Galb1-4GlcNAcb1-2Mana1-6(Neu5Aca2-6Galb1-4GlcNAcb1-3Galb1-4GlcNAcb1-2Mana1-3)Manb1-4GlcNAcb1-4GlcNAcb-Sp12	6	6	94
348	Neu5Aca2-6Galb1-4GlcNAcb1-2Mana1-3Manb1-4GlcNAcb1-4GlcNAc-Sp12	6	1	18
536	GalNAca1-3(Fuca1-2)Galb1-3GalNAcb1-3Gala1-4Galb1-4Glc-Sp21	6	7	113
589	Galb1-4GlcNAcb1-3Galb1-4GlcNAcb1-3GalNAca-Sp14	6	2	32
308	Neu5Aca2-6Galb1-4GlcNAcb1-2Mana1-6(GlcNAcb1-2Mana1-3)Manb1-4GlcNAcb1-4GlcNAcb-Sp12	6	5	77
376	Neu5Aca2-3Galb1-4GlcNAcb1-3GalNAc-Sp14	6	3	42
108	Gala1-3(Fuca1-2)Galb-Sp18	6	6	94
210	Mana1-6(Mana1-2Mana1-3)Mana1-6(Mana1-2Mana1-3)Manb1-4GlcNAcb1-4GlcNAcb-Sp12	6	9	155
98	GalNAcb1-4GlcNAcb-Sp0	6	9	146
4	GalNAca-Sp8	6	4	65
445	Fuca1-2Galb1-4 GlcNAcb1-2Mana1-6(Fuca1-2Galb1-4GlcNAcb1-2(Fuca1-2Galb1-4GlcNAcb1-4)Mana1-3)Manb1-4GlcNAcb1-4GlcNAcb-Sp12	6	8	133
241	Neu5Aca2-3Galb1-4(Neu5Aca2-3Galb1-3)GlcNAcb-Sp8	6	6	100
227	Neu5Aca2-8Neu5Aca2-8Neu5Aca2-3Galb1-4Glcb-Sp0	6	6	106
66	Fuca1-2Galb1-3GlcNAcb1-3Galb1-4Glcb-Sp10	6	3	50
280	Neu5Gca2-3Galb1-3(Fuca1-4)GlcNAcb-Sp0	6	7	123
521	Gala1-3Galb1-4GlcNAcb1-2Mana-Sp0	6	5	78
270	Neu5Aca2-6Galb1-4GlcNAcb1-3Galb1-4(Fuca1-3)GlcNAcb1-3Galb1-4(Fuca1-3)GlcNAcb-Sp0	6	10	164
79	Fuca1-3GlcNAcb-Sp8	6	7	124
491	Neu5Aca2-3Galb1-3GlcNAcb1-6GalNAca-Sp14	6	2	34
298	(6P)Glcb-Sp10	6	8	134
69	Fuca1-2Galb1-4(Fuca1-3)GlcNAcb1-3Galb1-4(Fuca1-3)GlcNAcb-Sp0	6	6	106
465	Neu5Aca2-6Galb1-4GlcNAcb1-6(Neu5Aca2-6Galb1-4GlcNAcb1-2)Mana1-6(GlcNAcb1-4)(Neu5Aca2-6Galb1-4GlcNAcb1-2Mana1-3)Manb1-4GlcNAcb1-4GlcNAcb-Sp21	6	0	8
47	(6S)GlcNAcb-Sp8	6	4	73
512	(6S)GalNAcb1-4GlcNAc-Sp8	6	6	99
131	Galb1-4GlcNAcb1-6GalNAca-Sp8	6	7	128
170	Galb1-4GlcNAcb-Sp23	6	7	128
347	Neu5Aca2-6Galb1-4GlcNAcb1-2Mana1-6Manb1-4GlcNAcb1-4GlcNAc-Sp12	6	6	110
177	GlcNAcb1-6(GlcNAcb1-3)GalNAca-Sp14	6	14	247
250	Neu5Aca2-3Galb1-3GlcNAcb-Sp8	6	3	58
266	Neu5Aca2-6GalNAcb1-4GlcNAcb-Sp0	6	2	39
357	KDNa2-6Galb1-4GlcNAc-Sp0	6	2	40
542	Galb1-4GlcNAcb1-3Galb1-4GlcNAcb1-2Mana1-6(Galb1-4GlcNAcb1-3Galb1-4GlcNAcb1-2Mana1-3)Manb1-4GlcNAcb1-4GlcNAcb-Sp24	6	12	215
396	Neu5Aca2-3Galb1-3GlcNAcb1-2Mana1-6(Neu5Aca2-3Galb1-3GlcNAcb1-2Mana1-3)Manb1-4GlcNAcb1-4GlcNAc-Sp19	6	2	30
449	Fuca1-2Galb1-4GlcNAcb1-6(Fuca1-2Galb1-4GlcNAcb1-3)GalNAc-Sp14	6	6	99
84	(3S)Galb1-4(Fuca1-3)Glcb-Sp0	6	6	98
77	Fuca1-2Galb1-4Glcb-Sp0	6	9	153
323	Neu5Gcb2-6Galb1-4GlcNAc-Sp8	6	6	111
558	GlcNAcb1-3Galb1-4GlcNAcb1-6(GlcNAcb1-3Galb1-4GlcNAcb1-2)Mana1-6(GlcNAcb1-3Galb1-4GlcNAcb1-2Man a1-3)Manb1-4GlcNAcb1-4GlcNAc-Sp24	6	6	109
176	GlcNAcb1-6(GlcNAcb1-3)GalNAca-Sp8	5	2	43
268	Neu5Aca2-6Galb1-4GlcNAcb-Sp0	5	6	113
174	GlcNAca1-6Galb1-4GlcNAcb-Sp8	5	6	102
297	(6S)Galb1-4(6S)GlcNAcb-Sp0	5	3	48
358	KDNa2-3Galb1-4Glc-Sp0	5	6	117
434	GlcNAcb1-6(GlcNAcb1-2)Mana1-6(GlcNAcb1-4)(GlcNAcb1-4(GlcNAcb1-2)Mana1-3)Manb1-4GlcNAcb1-4GlcNAc-Sp21	5	7	136
257	Neu5Aca2-3Galb1-4(Fuca1-3)GlcNAcb1-3Galb1-4GlcNAcb-Sp8	5	3	47
204	GlcAb1-6Galb-Sp8	5	5	94
303	GlcNAcb1-6(Galb1-4GlcNAcb1-3)Galb1-4GlcNAc-Sp0	5	4	81
607	Neu5Aca2-3Galb1-4GlcNAcb1-3Galb1-4GlcNAcb1-3Galb1-4GlcNAcb1-2Mana1-6(Neu5Aca2-3Galb1-4GlcNAcb1-3Galb1-4GlcNAcb1-3Galb1-4GlcNAcb1-2Mana1-3)Manb1-4GlcNAcb1-4GlcNAcb-Sp12	5	6	107
78	Fuca1-2Galb-Sp8	5	3	65
35	(3S)Galb1-4(6S)GlcNAcb-Sp8	5	3	52
113	Gala1-3Galb1-4(Fuca1-3)GlcNAcb-Sp8	5	4	71
377	Neu5Aca2-6Galb1-4GlcNAcb1-3GalNAc-Sp14	5	7	134
400	Fuca1-2Galb1-4GlcNAcb1-3GalNAca-Sp14	5	2	35
121	Gala1-4Galb1-4GlcNAcb-Sp8	5	4	79
134	GlcNAcb1-6(Galb1-3)GalNAca-Sp14	5	6	110
461	Neu5Aca2-3Galb1-4GlcNAcb1-6(Neu5Aca2-3Galb1-4GlcNAcb1-2)Mana1-6(GlcNAcb1-4)(Neu5Aca2-3Galb1-4GlcNAcb1-2Mana1-3)Manb1-4GlcNAcb1-4GlcNAcb-Sp21	5	1	26
585	GlcNAcb1-3Galb1-4GlcNAcb1-3Galb1-4GlcNAcb1-3Galb1-4GlcNAcb1-6(GlcNAcb1-3Galb1-4GlcNAcb1-3Galb1-4GlcNAcb1-3Galb1-4GlcNAb1-2)Mana1-6(GlcNAcb1-3Galb1-4GlcNAcb1-3Galb1-4GlcNAcb1-3Galb1-4GlcNAcb1-2Mana1-3)Manb1-4GlcNAcb1-4(Fuca1-6)GlcNAcb-Sp24	5	4	74
180	GlcNAcb1-3GalNAca-Sp14	5	4	83
483	Neu5Aca2-3Galb1-4GlcNAcb1-2Mana1-6(Neu5Aca2-3Galb1-4GlcNAcb1-2Mana1-3)Manb1-4GlcNAcb1-4(Fuca1-6)GlcNAcb-Sp24	5	2	39
548	Galb1-4GlcNAcb1-3Galb1-4GlcNAcb1-3Galb1-4GlcNAcb1-2Mana1-6(Galb1-4GlcNAcb1-3Galb1-4GlcNAcb1-3Galb1-4GlcNAcb1-2Mana1-3)Manb1-4GlcNAcb1-4GlcNAcb-Sp24	5	11	222
519	GalNAca1-3(Fuca1-2)Galb1-4GlcNAcb1-6GalNAc-Sp14	5	4	72
507	GlcNAcb1-6(GlcNAcb1-2)Mana1-6(GlcNAcb1-4)(GlcNAcb1-4(GlcNAcb1-2)Mana1-3)Manb1-4GlcNAcb1-4(Fuca1-6)GlcNAc-Sp21	5	5	104
291	Galb1-4(Fuca1-3)(6S)Glcb-Sp0	5	7	135
470	Glca1-4Glca1-4Glca1-4Glcb-Sp10	5	7	137
37	(3S)Galb1-4GlcNAcb-Sp8	5	4	71
288	Neu5Aca2-3Galb1-4GlcNAcb1-6(Galb1-3)GalNAca-Sp14	5	4	76
436	Galb1-4GlcNAcb1-2Mana1-6(GlcNAcb1-4)(Galb1-4GlcNAcb1-4(Galb1-4GlcNAcb1-2)Mana1-3)Manb1-4GlcNAcb1-4GlcNAc-Sp21	5	8	161
45	(6S)Galb1-4(6S)Glcb-Sp8	5	2	43
103	Gala1-3(Fuca1-2)Galb1-4(Fuca1-3)GlcNAcb-Sp0	5	3	61
378	Neu5Aca2-3Galb1-4(Fuca1-3)GlcNAcb1-3GalNAca-Sp14	5	5	100
324	Galb1-3GlcNAcb1-2Mana1-6(Galb1-3GlcNAcb1-2Mana1-3)Manb1-4GlcNAcb1-4GlcNAcb-Sp19	5	5	98
122	Gala1-4Galb1-4Glcb-Sp0	5	2	36
228	GalNAcb1-4(Neu5Aca2-8Neu5Aca2-3)Galb1-4Glcb-Sp0	5	8	162
116	Gala1-3Galb1-4Glcb-Sp0	5	5	95
165	Galb1-4GlcNAcb1-3Galb1-4Glcb-Sp8	5	4	77
36	(3S)Galb1-4GlcNAcb-Sp0	5	4	81
307	GlcAb1-3GlcNAcb-Sp8	5	6	132
532	Galb1-4GlcNAcb1-2 Mana1-6(Galb1-4GlcNAcb1-4)(Galb1-4GlcNAcb1-2Mana1-3)Manb1-4GlcNAcb1-4(Fuca1-6)GlcNAc-Sp21	5	10	204
151	Galb1-4(Fuca1-3)GlcNAcb-Sp0	5	3	69
546	GlcNAcb1-3Galb1-4GlcNAcb1-3Galb1-4GlcNAcb1-2Mana1-6(GlcNAcb1-3Galb1-4GlcNAcb1-3Galb1-4GlcNAcb1-2Mana1-3)Manb1-4GlcNAcb1-4GlcNAcb-Sp25	5	8	171
130	Fuca1-4(Galb1-3)GlcNAcb-Sp8	5	3	62
114	Gala1-3Galb1-3GlcNAcb-Sp0	5	9	186
107	Gala1-3(Fuca1-2)Galb-Sp8	5	4	85
27	(3S)Galb1-4(6S)Glcb-Sp8	5	8	166
460	Neu5Aca2-3Galb1-4GlcNAcb1-4Mana1-6(GlcNAcb1-4)(Neu5Aca2-3Galb1-4GlcNAcb1-4(Neu5Aca2-3Galb1-4GlcNAcb1-2)Mana1-3)Manb1-4GlcNAcb1-4GlcNAcb-Sp21	5	5	107
321	Neu5Aca2-8Neu5Acb-Sp17	5	6	135
8	Rhaa-Sp8	5	3	74
466	Neu5Aca2-6Galb1-4GlcNAcb1-6(Neu5Aca2-6Galb1-4GlcNAcb1-2)Mana1-6(GlcNAcb1-4)(Neu5Aca2-6Galb1-4GlcNAcb1-4(Neu5Aca2-6Galb1-4GlcNAcb1-2)Mana1-3)Manb1-4GlcNAcb1-4GlcNAcb-Sp21	5	6	122
33	(3S)Galb1-4(Fuca1-3)GlcNAc-Sp8	5	4	79
124	Gala1-6Glcb-Sp8	5	2	50
379	GalNAcb1-4GlcNAcb1-2Mana1-6(GalNAcb1-4GlcNAcb1-2Mana1-3)Manb1-4GlcNAcb1-4GlcNAc-Sp12	4	1	31
50	Mana1-6(Mana1-3)Manb1-4GlcNAcb1-4GlcNAcb-Sp12	4	5	103
354	Galb1-3GlcNAcb1-2Mana1-6(Galb1-3GlcNAcb1-2Mana1-3)Manb1-4GlcNAcb1-4(Fuca1-6)GlcNAcb-Sp22	4	12	279
551	Galb1-3GlcNAcb1-3Galb1-4GlcNAcb1-2Mana1-6(Galb1-3GlcNAcb1-3Galb1-4GlcNAcb1-2Mana1-3)Manb1-4GlcNAcb1-4GlcNAc-Sp25	4	1	25
412	Neu5Aca2-3Galb1-3GalNAcb1-4(Neu5Aca2-8Neu5Aca2-3)Galb1-4Glcb-Sp0	4	5	115
490	Galb1-3(Fuca1-4)GlcNAcb1-6GalNAca-Sp14	4	4	89

402	GalNAca1-3GalNAcb1-3Gala1-4Galb1-4GlcNAcb-Sp0	2	5	256
403	Gala1-4Galb1-3GlcNAcb1-2Mana1-6(Gala1-4Galb1-3GlcNAcb1-2Mana1-3)Manb1-4GlcNAcb1-4GlcNAcb-Sp19	19	12	65
404	Gala1-4Galb1-4GlcNAcb1-2Mana1-6(Gala1-4Galb1-4GlcNAcb1-2Mana1-3)Manb1-4GlcNAcb1-4GlcNAcb-Sp24	3	10	306
405	Gala1-3Galb1-4GlcNAcb1-3GalNAca-Sp14	9	20	213
406	Galb1-3GlcNAcb1-6Galb1-4GlcNAcb-Sp0	1	6	511
407	Galb1-3GlcNAca1-6Galb1-4GlcNAcb-Sp0	14	8	56
408	GalNAcb1-3Gala1-6Galb1-4Glcb-Sp8	-1	6	-745
409	Gala1-3(Fuca1-2)Galb1-4(Fuca1-3)Glcb-Sp21	11	5	47
410	Galb1-4GlcNAcb1-6(Neu5Aca2-6Galb1-3GlcNAcb1-3)Galb1-4Glc-Sp21	8	8	105
411	Galb1-3GalNAcb1-4(Neu5Aca2-8Neu5Aca2-3)Galb1-4Glcb-Sp0	7	6	90
412	Neu5Aca2-3Galb1-3GalNAcb1-4(Neu5Aca2-8Neu5Aca2-3)Galb1-4Glcb-Sp0	4	5	115
413	Gala1-3(Fuca1-2)Galb1-4GlcNAcb1-3GalNAca-Sp14	10	4	45
414	GalNAca1-3(Fuca1-2)Galb1-4GlcNAcb1-3GalNAca-Sp14	3	6	197
415	GalNAca1-3GalNAcb1-3Gala1-4Galb1-4Glcb-Sp0	4	5	135
416	Fuca1-2Galb1-4(Fuca1-3)GlcNAcb1-3GalNAca-Sp14	1	2	254
417	Gala1-3(Fuca1-2)Galb1-4(Fuca1-3)GlcNAcb1-3GalNAc-Sp14	0	6	-1303
418	GalNAca1-3(Fuca1-2)Galb1-4(Fuca1-3)GlcNAcb1-3GalNAc-Sp14	0	6	-6969
419	Galb1-4(Fuca1-3)GlcNAcb1-2Mana1-6(Galb1-4(Fuca1-3)GlcNAcb1-2Mana1-3)Manb1-4GlcNAcb1-4(Fuca1-6)GlcNAcb-Sp22	-2	4	-225
420	Fuca1-2Galb1-4GlcNAcb1-2Mana1-6(Fuca1-2Galb1-4GlcNAcb1-2Mana1-3)Manb1-4GlcNAcb1-4(Fuca1-6)GlcNAcb-Sp22	11	11	100
421	GlcNAcb1-2(GlcNAcb1-6)Mana1-6(GlcNAcb1-2Mana1-3)Manb1-4GlcNAcb1-4GlcNAcb-Sp19	18	16	94
422	Fuca1-2Galb1-3GlcNAcb1-3GalNAc-Sp14	3	4	163
423	Gala1-3(Fuca1-2)Galb1-3GlcNAcb1-3GalNAc-Sp14	14	13	97
424	GalNAca1-3(Fuca1-2)Galb1-3GlcNAcb1-3GalNAc-Sp14	3	3	92
425	Gala1-3Galb1-3GlcNAcb1-3GalNAc-Sp14	8	5	66
426	Fuca1-2Galb1-3GlcNAcb1-2Mana1-6(Fuca1-2Galb1-3GlcNAcb1-2Mana1-3)Manb1-4GlcNAcb1-4(Fuca1-6)GlcNAcb-Sp22	7	3	45
427	Gala1-3(Fuca1-2)Galb1-4GlcNAcb1-2Mana1-6(Gala1-3(Fuca1-2)Galb1-4GlcNAcb1-2Mana1-3)Manb1-4GlcNAcb1-4(Fuca1-6)GlcNAcb-Sp22	-9	6	-68
428	Galb1-3GlcNAcb1-6(Galb1-3GlcNAcb1-2)Mana1-6(Galb1-3GlcNAcb1-2Mana1-3)Manb1-4GlcNAcb1-4GlcNAcb-Sp19	7	4	56
429	Galb1-4GlcNAcb1-6(Fuca1-2Galb1-3GlcNAcb1-3)Galb1-4Glc-Sp21	8	5	59
430	Fuca1-3GlcNAcb1-6(Galb1-4GlcNAcb1-3)Galb1-4Glc-Sp21	10	5	44
431	GlcNAcb1-2Mana1-6(GlcNAcb1-4)(GlcNAcb1-2Mana1-3)Manb1-4GlcNAcb1-4GlcNAc-Sp21	9	8	83
432	GlcNAcb1-2Mana1-6(GlcNAcb1-4)(GlcNAcb1-4(GlcNAcb1-2)Mana1-3)Manb1-4GlcNAcb1-4GlcNAc-Sp21	9	4	48
433	GlcNAcb1-6(GlcNAcb1-2)Mana1-6(GlcNAcb1-4)(GlcNAcb1-2Mana1-3)Manb1-4GlcNAcb1-4GlcNAc-Sp21	6	6	97
434	GlcNAcb1-6(GlcNAcb1-2)Mana1-6(GlcNAcb1-4)(GlcNAcb1-4(GlcNAcb1-2)Mana1-3)Manb1-4GlcNAcb1-4GlcNAc-Sp21	5	7	136
435	Galb1-4GlcNAcb1-2Mana1-6(GlcNAcb1-4)(Galb1-4GlcNAcb1-2Mana1-3)Manb1-4GlcNAcb1-4GlcNAc-Sp21	7	7	94
436	Galb1-4GlcNAcb1-2Mana1-6(GlcNAcb1-4)(Galb1-4GlcNAcb1-4(Galb1-4GlcNAcb1-2)Mana1-3)Manb1-4GlcNAcb1-4GlcNAc-Sp21	5	8	161
437	Galb1-4GlcNAcb1-6(Galb1-4GlcNAcb1-2)Mana1-6(GlcNAcb1-4)(Galb1-4GlcNAcb1-2Mana1-3)Manb1-4GlcNAcb1-4GlcNAc-Sp21	10	11	105
438	Galb1-4GlcNAcb1-6(Galb1-4GlcNAcb1-2)Mana1-6(GlcNAcb1-4)(Galb1-4GlcNAcb1-4(Galb1-4GlcNAcb1-2)Mana1-3)Manb1-4GlcNAcb1-4GlcNAc-Sp21	7	4	54
439	Galb1-4Galb-Sp10	2	4	184
440	Galb1-6Galb-Sp10	4	4	107
441	Neu5Aca2-3Galb1-4GlcNAcb1-3Galb-Sp8	10	10	96
442	GalNAcb1-6GalNAcb-Sp8	3	6	234
443	(6S)Galb1-3GlcNAcb-Sp0	8	5	57
444	(6S)Galb1-3(6S)GlcNAc-Sp0	2	3	154
445	Fuca1-2Galb1-4 GlcNAcb1-2Mana1-6(Fuca1-2Galb1-4GlcNAcb1-2(Fuca1-2Galb1-4GlcNAcb1-4)Mana1-3)Manb1-4GlcNAcb1-4GlcNAcb-Sp12	6	8	133
446	Fuca1-2Galb1-4(Fuca1-3)GlcNAcb1-2Mana1-6(Fuca1-2Galb1-4(Fuca1-3)GlcNAcb1-4(Fuca1-2Galb1-4(Fuca1-3)GlcNAcb1-2)Mana1-3)Manb1-4GlcNAcb1-4GlcNAcb-Sp12	9	3	37
447	Galb1-4(Fuca1-3)GlcNAcb1-6GalNAc-Sp14	1	6	594
448	Galb1-4GlcNAcb1-2Mana-Sp0	3	8	236
449	Fuca1-2Galb1-4GlcNAcb1-6(Fuca1-2Galb1-4GlcNAcb1-3)GalNAc-Sp14	6	6	99
450	Gala1-3(Fuca1-2)Galb1-4GlcNAcb1-6(Gala1-3(Fuca1-2)Galb1-4GlcNAcb1-3)GalNAc-Sp14	-3	2	-59
451	GalNAca1-3(Fuca1-2)Galb1-4GlcNAcb1-6(GalNAca1-3(Fuca1-2)Galb1-4GlcNAcb1-3)GalNAc-Sp14	3	1	45
452	Neu5Aca2-8Neu5Aca2-3Galb1-3GalNAcb1-4(Neu5Aca2-8Neu5Aca2-3)Galb1-4Glcb-Sp0	10	9	84
453	GalNAcb1-4Galb1-4Glcb-Sp0	11	2	14
454	GalNAca1-3(Fuca1-2)Galb1-4GlcNAcb1-2Mana1-6(GalNAca1-3(Fuca1-2)Galb1-4GlcNAcb1-2Mana1-3)Manb1-4GlcNAcb1-4(Fuca1-6)GlcNAcb-Sp22	7	7	101
455	Gala1-3(Fuca1-2)Galb1-3GlcNAcb1-2Mana1-6(Gala1-3(Fuca1-2)Galb1-3GlcNAcb1-2Mana1-3)Manb1-4GlcNAcb1-4(Fuca1-6)GlcNAcb-Sp22	3	1	36
456	Neu5Aca2-6Galb1-4GlcNAcb1-6(Fuca1-2Galb1-3GlcNAcb1-3)Galb1-4Glc-Sp21	8	7	86
457	GalNAca1-3(Fuca1-2)Galb1-3GlcNAcb1-2Mana1-6(GalNAca1-3(Fuca1-2)Galb1-3GlcNAcb1-2Mana1-3)Manb1-4GlcNAcb1-4(Fuca1-6)GlcNAcb-Sp22	14	7	55
458	Galb1-4GlcNAcb1-6(Galb1-4GlcNAcb1-2)Mana1-6(Galb1-4GlcNAcb1-2Mana1-3)Manb1-4GlcNAcb1-4GlcNAcb-Sp19	9	5	55
459	Neu5Aca2-3Galb1-4GlcNAcb1-2Mana1-6(GlcNAcb1-4)(Neu5Aca2-3Galb1-4GlcNAcb1-2Mana1-3)Manb1-4GlcNAcb1-4GlcNAcb-Sp21	6	3	46
460	Neu5Aca2-3Galb1-4GlcNAcb1-4Mana1-6(GlcNAcb1-4)(Neu5Aca2-3Galb1-4GlcNAcb1-4(Neu5Aca2-3Galb1-4GlcNAcb1-2)Mana1-3)Manb1-4GlcNAcb1-4GlcNAcb-Sp21	5	5	107
461	Neu5Aca2-3Galb1-4GlcNAcb1-6(Neu5Aca2-3Galb1-4GlcNAcb1-2)Mana1-6(GlcNAcb1-4)(Neu5Aca2-3Galb1-4GlcNAcb1-2Mana1-3)Manb1-4GlcNAcb1-4GlcNAcb-Sp21	5	1	26
462	Neu5Aca2-3Galb1-4GlcNAcb1-6(Neu5Aca2-3Galb1-4GlcNAcb1-2)Mana1-6(GlcNAcb1-4)(Neu5Aca2-3Galb1-4GlcNAcb1-4(Neu5Aca2-3Galb1-4GlcNAcb1-2)Mana1-3)Manb1-4GlcNAcb1-4GlcNAcb-Sp21	8	8	98
463	Neu5Aca2-6Galb1-4GlcNAcb1-2Mana1-6(GlcNAcb1-4)(Neu5Aca2-6Galb1-4GlcNAcb1-2Mana1-3)Manb1-4GlcNAcb1-4GlcNAcb-Sp21	8	5	58
464	Neu5Aca2-6Galb1-4GlcNAcb1-4Mana1-6(GlcNAcb1-4)(Neu5Aca2-6Galb1-4GlcNAcb1-4(Neu5Aca2-6Galb1-4GlcNAcb1-2)Mana1-3)Manb1-4GlcNAcb1-4GlcNAcb-Sp21	1	2	281
465	Neu5Aca2-6Galb1-4GlcNAcb1-6(Neu5Aca2-6Galb1-4GlcNAcb1-2)Mana1-6(GlcNAcb1-4)(Neu5Aca2-6Galb1-4GlcNAcb1-2Mana1-3)Manb1-4GlcNAcb1-4GlcNAcb-Sp21	6	0	8
466	Neu5Aca2-6Galb1-4GlcNAcb1-6(Neu5Aca2-6Galb1-4GlcNAcb1-2)Mana1-6(GlcNAcb1-4)(Neu5Aca2-6Galb1-4GlcNAcb1-4(Neu5Aca2-6Galb1-4GlcNAcb1-2)Mana1-3)Manb1-4GlcNAcb1-4GlcNAcb-Sp21	5	6	122
467	Gala1-3(Fuca1-2)Galb1-3GalNAca-Sp8	7	5	73
468	Gala1-3(Fuca1-2)Galb1-3GalNAcb-Sp8	10	5	45
469	Glca1-6Glca1-6Glca1-6Glcb-Sp10	3	4	119
470	Glca1-4Glca1-4Glca1-4Glcb-Sp10	5	7	137
471	Neu5Aca2-3Galb1-4GlcNAcb1-6(Neu5Aca2-3Galb1-4GlcNAcb1-3)GalNAca-Sp14	7	6	84
472	Fuca1-2Galb1-4(Fuca1-3)GlcNAcb1-2Mana1-6(Fuca1-2Galb1-4(Fuca1-3)GlcNAcb1-2Mana1-3)Manb1-4GlcNAcb1-4(Fuca1-6)GlcNAcb-Sp24	12	9	79
473	Fuca1-2Galb1-3(Fuca1-4)GlcNAcb1-2Mana1-6(Fuca1-2Galb1-3(Fuca1-4)GlcNAcb1-2Mana1-3)Manb1-4GlcNAcb1-4(Fuca1-6)GlcNAcb1-4(Fuca1-6)GlcNAcb-Sp19	7	7	99
474	Neu5Aca2-3Galb1-3GlcNAcb1-6(Neu5Aca2-3Galb1-3GlcNAcb1-2)Mana1-6(Neu5Aca2-3Galb1-3GlcNAcb1-2Mana1-3)Manb1-4GlcNAcb1-4GlcNAcb-Sp19	0	6	-3711
475	GlcNAcb1-6(GlcNAcb1-2)Mana1-6(GlcNAcb1-2Mana1-3)Manb1-4GlcNAcb1-4(Fuca1-6)GlcNAcb-Sp24	12	6	49
476	Galb1-3GlcNAcb1-2Mana1-6(GlcNAcb1-4)(Galb1-3GlcNAcb1-2Mana1-3)Manb1-4GlcNAcb1-4GlcNAcb-Sp21	16	9	60
477	Neu5Aca2-6Galb1-4GlcNAcb1-6(Galb1-3GlcNAcb1-3)Galb1-4Glcb-Sp21	8	6	85
478	Neu5Aca2-3Galb1-4GlcNAcb1-2Mana-Sp0	17	6	37
479	Neu5Aca2-3Galb1-4GlcNAcb1-6GalNAca-Sp14	7	8	115
480	Neu5Aca2-6Galb1-4GlcNAcb1-6GalNAca-Sp14	8	12	149
481	Neu5Aca2-6Galb1-4 GlcNAcb1-6(Neu5Aca2-6Galb1-4GlcNAcb1-3)GalNAca-Sp14	6	7	104
482	Neu5Aca2-6Galb1-4GlcNAcb1-2Mana1-6(Neu5Aca2-6Galb1-4GlcNAcb1-2Mana1-3)Manb1-4GlcNAcb1-4(Fuca1-6)GlcNAcb-Sp24	12	6	52
483	Neu5Aca2-3Galb1-4GlcNAcb1-2Mana1-6(Neu5Aca2-3Galb1-4GlcNAcb1-2Mana1-3)Manb1-4GlcNAcb1-4(Fuca1-6)GlcNAcb-Sp24	5	2	39
484	Mana1-6(Mana1-3)Manb1-4GlcNAcb1-4(Fuca1-6)GlcNAcb-Sp19	3	7	228
485	Galb1-4GlcNAcb1-6(Galb1-4GlcNAcb1-2)Mana1-6(Galb1-4GlcNAcb1-2Mana1-3)Manb1-4GlcNAcb1-4(Fuca1-6)GlcNAcb-Sp24	7	5	73
486	Neu5Aca2-3Galb1-3GlcNAcb1-2Mana1-6(GlcNAcb1-4)(Neu5Aca2-3Galb1-3GlcNAcb1-2Mana1-3)Manb1-4GlcNAcb1-4GlcNAc-Sp21	14	10	72
487	Neu5Aca2-6Galb1-4GlcNAcb1-6(Fuca1-2Galb1-4(Fuca1-3)GlcNAcb1-3)Galb1-4Glc-Sp21	-1	4	-312
488	Galb1-3GlcNAcb1-6GalNAca-Sp14	4	7	179
489	Gala1-3Galb1-3GlcNAcb1-6GalNAca-Sp14	15	18	122
490	Galb1-3(Fuca1-4)GlcNAcb1-6GalNAca-Sp14	4	4	89
491	Neu5Aca2-3Galb1-3GlcNAcb1-6GalNAca-Sp14	6	2	34
492	(3S)Galb1-3(Fuca1-4)GlcNAcb-Sp0	7	8	105
493	Galb1-4(Fuca1-3)GlcNAcb1-6(Neu5Aca2-6(Neu5Aca2-3Galb1-3)GlcNAcb1-3)Galb1-4Glc-Sp21	11	8	80
494	Fuca1-2Galb1-4GlcNAcb1-6GalNAca-Sp14	12	9	72
495	Gala1-3Galb1-4GlcNAcb1-6GalNAca-Sp14	0	2	-632
496	Galb1-4(Fuca1-3)GlcNAcb1-2Mana-Sp0	0	4	1592
497	Fuca1-2(6S)Galb1-3GlcNAcb-Sp0	14	9	65
498	Gala1-3(Fuca1-2)Galb1-4GlcNAcb1-6GalNAca-Sp14	0	3	711

593	GlcNAcb1-3Galb1-4GlcNAcb1-3GalNAca-Sp14	4	4	83
301	Neu5Aca2-6Galb1-4GlcNAcb1-2Mana1-6(Galb1-4GlcNAcb1-2Mana1-3)Manb1-4GlcNAcb1-4GlcNAcb-Sp12	4	5	108
601	Neu5Aca2-6Galb1-4GlcNAcb1-3Galb1-4GlcNAcb1-6(Galb1-3)GalNAca-Sp14	4	4	87
488	Galb1-3GlcNAcb1-6GalNAca-Sp14	4	7	179
236	Neu5Aca2-3GalNAca-Sp8	4	7	164
343	GlcNAca1-4Galb1-4GlcNAcb1-3Galb1-4GlcNAcb-Sp0	4	3	70
9	Neu5Aca-Sp8	4	2	42
139	Galb1-3GalNAca-Sp8	4	3	67
313	Mana1-6Manb-Sp10	4	5	119
239	Neu5Aca2-3Galb1-3(Fuca1-4)GlcNAcb-Sp8	4	6	146
11	Neu5Acb-Sp8	4	3	85
440	Galb1-6Galb-Sp10	4	4	107
360	Fuca1-2Galb1-3GlcNAcb1-2Mana1-6(Fuca1-2Galb1-3GlcNAcb1-2Mana1-3)Manb1-4GlcNAcb1-4GlcNAcb-Sp20	4	5	137
375	Fuca1-4(Fuca1-2Galb1-3)GlcNAcb1-2Mana1-3(Fuca1-4(Fuca1-2Galb1-3)GlcNAcb1-2Mana1-3)Manb1-4GlcNAcb1-4GlcNAcb-Sp19	4	4	106
255	Neu5Aca2-3Galb1-4(Fuca1-3)GlcNAcb-Sp8	4	7	170
349	Galb1-4GlcNAcb1-2Mana1-3Manb1-4GlcNAcb1-4GlcNAc-Sp12	4	3	84
415	GalNAca1-3GalNAcb1-3Gala1-4Galb1-4Glcb-Sp0	4	5	135
175	GlcNAcb1-2Galb1-3GalNAca-Sp8	4	4	117
275	Neu5Aca2-8Neu5Aca-Sp8	4	5	144
355	(6S)GlcNAcb1-3Galb1-4GlcNAcb-Sp0	4	4	100
112	Gala1-3GalNAcb-Sp8	4	4	103
300	Galb1-3Galb1-4GlcNAcb-Sp8	4	4	103
117	Gala1-3Galb1-4Glc-Sp10	4	3	81
596	Neu5Aca2-3Galb1-4GlcNAcb1-3Galb1-4GlcNAcb1-6(Neu5Aca2-3Galb1-4GlcNAcb1-3Galb1-4GlcNAcb1-3)GalNAca-Sp14	4	6	176
218	Neu5Aca2-3Galb1-4GlcNAcb1-3Galb1-4(Fuca1-3)GlcNAcb-Sp0	4	3	91
338	GlcNAca1-4Galb1-4GlcNAcb1-3Galb1-4GlcNAcb1-3Galb1-4GlcNAcb-Sp0	4	6	162
59	Fuca1-2Galb1-3GalNAcb1-3Gala1-4Galb1-4Glcb-Sp9	4	6	164
395	Gala1-3Galb1-3(Fuca1-4)GlcNAcb1-2Mana1-6(Gala1-3Galb1-3(Fuca1-4)GlcNAcb1-2Mana1-3)Manb1-4GlcNAcb1-4GlcNAc-Sp19	4	7	190
104	Gala1-3(Fuca1-2)Galb1-4(Fuca1-3)GlcNAcb-Sp8	4	7	187
203	GlcAb1-3Galb-Sp8	4	1	35
531	Galb1-4GlcNAcb1-2 Mana1-6(GlcNAcb1-4)(Galb1-4GlcNAcb1-2Mana1-3)Manb1-4GlcNAcb1-4(Fuca1-6)GlcNAc-Sp21	4	6	157
514	GalNAcb1-4(6S)GlcNAc-Sp8	4	3	83
92	GalNAca1-3Galb-Sp8	4	3	80
38	(3S)Galb-Sp8	4	3	80
366	Neu5Aca2-6GlcNAcb1-4GlcNAc-Sp21	3	7	197
535	GlcNAcb1-3Galb1-4GlcNAcb1-6(GlcNAcb1-3)Galb1-4GlcNAc-Sp0	3	5	133
565	Galb1-4GlcNAcb1-3Galb1-4GlcNAcb1-3Galb1-4GlcNAcb1-3Galb1-4GlcNAcb1-3Galb1-4GlcNAcb1-2Mana1-6(Galb1-4GlcNAcb1-3Galb1-4GlcNAcb1-3Galb1-4GlcNAcb1-3Galb1-4GlcNAcb1-3Galb1-4GlcNAcb1-2Mana1-3)Manb1-4GlcNAcb1-4GlcNAcb-Sp25	3	2	52
251	Neu5Aca2-3Galb1-4(6S)GlcNAcb-Sp8	3	2	68
424	GalNAca1-3(Fuca1-2)Galb1-3GlcNAcb1-3GalNAc-Sp14	3	3	92
264	Neu5Aca2-3Galb1-4Glcb-Sp8	3	2	54
295	Neu5Aca2-3Galb1-4GlcNAcb1-3Galb1-3GlcNAcb-Sp0	3	2	66
340	GlcNAca1-4Galb1-3GlcNAcb-Sp0	3	2	59
57	Neu5Aca2-6Galb1-4GlcNAcb1-2Mana1-6(Neu5Aca2-6Galb1-4GlcNAcb1-2Mana1-3)Manb1-4GlcNAcb1-4GlcNAcb-Sp24	3	1	29
404	Gala1-4Galb1-4GlcNAcb1-2Mana1-6(Gala1-4Galb1-4GlcNAcb1-2Mana1-3)Manb1-4GlcNAcb1-4GlcNAcb-Sp24	3	10	306
373	GalNAca1-3(Fuca1-2)Galb1-3GlcNAcb1-2Mana1-6(GalNAca1-3(Fuca1-2)Galb1-3GlcNAcb1-2Mana1-3)Manb1-4GlcNAcb1-4GlcNAcb-Sp20	3	12	343
309	GlcNAcb1-3Man-Sp10	3	6	180
56	Neu5Aca2-6Galb1-4GlcNAcb1-2Mana1-6(Neu5Aca2-6Galb1-4GlcNAcb1-2Man-a1-3)Manb1-4GlcNAcb1-4GlcNAcb-Sp21	3	5	142
561	GlcNAcb1-3Galb1-4GlcNAcb1-6(GlcNAcb1-3Galb1-3)GalNAca-Sp14	3	3	86
192	GlcNAcb1-6GalNAca-Sp8	3	2	55
500	Fuca1-2Galb1-3(6S)GlcNAcb-Sp0	3	2	63
332	Neu5Aca2-6Galb1-4GlcNAcb1-3Galb1-4GlcNAcb1-3Galb1-4GlcNAcb-Sp0	3	5	137
448	Galb1-4GlcNAcb1-2Mana-Sp0	3	8	236
44	(6S)Galb1-4GlcNAcb-Sp8	3	5	138
525	Gala1-3(Fuca1-2)Galb1-3GlcNAcb1-6GalNAc-Sp14	3	2	62
571	Galb1-3GlcNAcb1-3Galb1-4GlcNAcb1-3Galb1-4GlcNAcb1-6(Galb1-3GlcNAcb1-3Galb1-4GlcNAcb1-3Galb1-4GlcNAb1-2)Mana1-6(Galb1-3GlcNAcb1-3Galb1-4GlcNAcb1-3Galb1-4GlcNAcb1-2Mana1-3)Manb1-4GlcNAcb1-4(Fuca1-6)GlcNAcb-Sp24	3	2	69
469	Glca1-6Glca1-6Glca1-6Glcb-Sp10	3	4	119
209	Mana1-2Mana1-3Mana-Sp9	3	3	97
150	Galb1-3GlcNAcb-Sp8	3	3	98
484	Mana1-6(Mana1-3)Manb1-4GlcNAcb1-4(Fuca1-6)GlcNAcb-Sp19	3	7	228
120	Gala1-4Galb1-4GlcNAcb-Sp0	3	8	265
451	GalNAca1-3(Fuca1-2)Galb1-4GlcNAcb1-6(GalNAca1-3(Fuca1-2)Galb1-4GlcNAcb1-3)GalNAc-Sp14	3	1	45
455	Gala1-3(Fuca1-2)Galb1-3GlcNAcb1-2Mana1-6(Gala1-3(Fuca1-2)Galb1-3GlcNAcb1-2Mana1-3)Manb1-4GlcNAcb1-4(Fuca1-6)GlcNAcb-Sp22	3	1	36
392	GalNAcb1-4(Neu5Aca2-3)Galb1-4GlcNAcb1-3GalNAca-Sp14	3	2	67
414	GalNAca1-3(Fuca1-2)Galb1-4GlcNAcb1-3GalNAca-Sp14	3	6	197
350	Galb1-4GlcNAcb1-2Mana1-6Manb1-4GlcNAcb1-4GlcNAc-Sp12	3	7	240
100	Gala1-2Galb-Sp8	3	1	37
1	Gala-Sp8	3	7	221
269	Neu5Aca2-6Galb1-4GlcNAcb-Sp8	3	4	118
515	(3S)GalNAcb1-4GlcNAc-Sp8	3	10	325
40	(4S)Galb1-4GlcNAcb-Sp8	3	2	81
22	6S(3S)Galb1-4(6S)GlcNAcb-Sp0	3	5	161
105	Gala1-3(Fuca1-2)Galb1-4GlcNAc-Sp0	3	10	364
320	GlcNAcb1-2Mana1-6(Neu5Aca2-6Galb1-4GlcNAcb1-2Mana1-3)Manb1-4GlcNAcb1-4GlcNAcb-Sp12	3	6	205
163	Galb1-4GlcNAcb1-3Galb1-4GlcNAcb-Sp0	3	3	114
314	Mana1-6(Mana1-3)Mana1-6(Mana1-3)Manb-Sp10	3	5	184
523	GalNAca1-3(Fuca1-2)Galb1-4 GlcNAcb1-2Mana-Sp0	3	7	247
245	Neu5Aca2-3Galb-Sp8	3	5	197
220	Fuca1-2(6S)Galb1-4GlcNAcb-Sp0	3	6	236
422	Fuca1-2Galb1-3GlcNAcb1-3GalNAc-Sp14	3	4	163
442	GalNAcb1-6GalNAcb-Sp8	3	6	234
223	Neu5Aca2-3Galb1-3GalNAca-Sp8	3	5	191
502	Neu5Aca2-6GalNAcb1-4(6S)GlcNAcb-Sp8	3	7	281
397	GlcNAcb1-2Mana1-6(Galb1-4GlcNAcb1-2Mana1-3)Manb1-4GlcNAcb1-4GlcNAc-Sp12	3	6	219
352	GlcNAcb1-2Mana1-6(GlcNAcb1-2Mana1-3)Manb1-4GlcNAcb1-4(Fuca1-6)GlcNAcb-Sp22	3	2	83
306	GalNAcb1-3Galb-Sp8	3	3	113
383	Galb1-4(Fuca1-3)GlcNAcb1-6(Galb1-3GlcNAcb1-3)Galb1-4Glc-Sp21	3	13	531
167	Galb1-4GlcNAcb1-6(Galb1-3)GalNAc-Sp14	3	7	268
368	Galb1-4(Fuca1-3)GlcNAcb1-6(Fuca1-2Galb1-4GlcNAcb1-3)Galb1-4Glc-Sp21	2	7	292
518	(6P)Galb1-4GlcNAcb-SP0	2	5	214
68	Fuca1-2Galb1-3GlcNAcb-Sp8	2	3	130
597	Neu5Aca2-6Galb1-4GlcNAcb1-3Galb1-4GlcNAcb1-3GalNAca-Sp14	2	2	102
2	Glca-Sp8	2	10	429
85	GalNAca1-3(Fuca1-2)Galb1-4GlcNAcb-Sp0	2	8	320
42	(6S)Galb1-4Glcb-Sp0	2	2	79
311	GlcNAcb1-4GlcNAcb-Sp12	2	5	203
439	Galb1-4Galb-Sp10	2	4	184
30	(3S)Galb1-3GlcNAcb-Sp0	2	3	134

499	Fuca1-2Galb1-4GlcNAcb1-2Mana-Sp0	12	2	20
500	Fuca1-2Galb1-3(6S)GlcNAcb-Sp0	3	2	63
501	Fuca1-2(6S)Galb1-3(6S)GlcNAcb-Sp0	13	14	103
502	Neu5Aca2-6GalNAcb1-4(6S)GlcNAcb-Sp8	3	7	281
503	GalNAcb1-4(Fuca1-3)(6S)GlcNAcb-Sp8	-1	4	-632
504	(3S)GalNAcb1-4(Fuca1-3)GlcNAcb-Sp8	6	3	49
505	Fuca1-2Galb1-3GlcNAcb1-6(Fuca1-2Galb1-3GlcNAcb1-3)GalNAca-Sp14	19	9	46
506	GalNAca1-3(Fuca1-2)Galb1-3GlcNAcb1-6GalNAca-Sp14	-2	4	-247
507	GlcNAcb1-6(GlcNAcb1-2)Mana1-6(GlcNAcb1-4)(GlcNAcb1-4(GlcNAcb1-2)Mana1-3)Manb1-4GlcNAcb1-4(Fuca1-6)GlcNAc-Sp21	5	5	104
508	Galb1-4GlcNAcb1-6(Galb1-4GlcNAcb1-2)Mana1-6(GlcNAcb1-4)Galb1-4GlcNAcb1-4(Gal b1-4GlcNAcb1-2)Mana1-3)Manb1-4GlcNAcb1-4(Fuca1-6)GlcNAc-Sp21	11	8	76
509	Galb1-3GlcNAca1-3Galb1-4GlcNAcb-Sp8	16	8	53
510	Galb1-3(6S)GlcNAcb-Sp8	21	12	56
511	(6S)(4S)GalNAcb1-4GlcNAc-Sp8	1	5	431
512	(6S)GalNAcb1-4GlcNAc-Sp8	6	6	99
513	(3S)GalNAcb1-4(3S)GlcNAc-Sp8	15	6	37
514	GalNAcb1-4(6S)GlcNAc-Sp8	4	3	83
515	(3S)GalNAcb1-4GlcNAc-Sp8	3	10	325
516	(4S)GalNAcb-Sp10	11	1	10
517	Galb1-4(6P)GlcNAcb-Sp0	-1	4	-403
518	(6P)Galb1-4GlcNAcb-SP0	2	5	214
519	GalNAca1-3(Fuca1-2)Galb1-4GlcNAcb1-6GalNAc-Sp14	5	4	72
520	Neu5Aca2-6Galb1-4GlcNAcb1-2Man-Sp0	7	3	40
521	Gala1-3Galb1-4GlcNAcb1-2Mana-Sp0	6	5	78
522	Gala1-3(Fuca1-2)Galb1-4GlcNAcb1-2Mana-Sp0	7	4	55
523	GalNAca1-3(Fuca1-2)Galb1-4 GlcNAcb1-2Mana-Sp0	3	7	247
524	Galb1-3GlcNAcb1-2Mana-Sp0	6	9	145
525	Gala1-3(Fuca1-2)Galb1-3GlcNAcb1-6GalNAc-Sp14	3	2	62
526	Neu5Aca2-3Galb1-3GlcNAcb1-2Mana-Sp0	10	9	87
527	Gala1-3Galb1-3GlcNAcb1-2Mana-Sp0	9	2	27
528	GalNAcb1-4GlcNAcb1-2Mana-Sp0	11	6	55
529	Neu5Aca2-3Galb1-3GalNAcb1-4Galb1-4Glcb-Sp0	13	7	55
530	GlcNAcb1-2 Mana1-6(GlcNAcb1-4)(GlcNAcb1-2Mana1-3)Manb1-4GlcNAcb1-4(Fuca1-6)GlcNAc-Sp21	8	3	34
531	Galb1-4GlcNAcb1-2 Mana1-6(GlcNAcb1-4)(Galb1-4GlcNAcb1-2Mana1-3)Manb1-4GlcNAcb1-4(Fuca1-6)GlcNAc-Sp21	4	6	157
532	Galb1-4GlcNAcb1-2 Mana1-6(Galb1-4GlcNAcb1-4)(Galb1-4GlcNAcb1-2Mana1-3)Manb1-4GlcNAcb1-4(Fuca1-6)GlcNAc-Sp21	5	10	204
533	Fuca1-4(Galb1-3)GlcNAcb1-2 Mana-Sp0	0	3	1264
534	Neu5Aca2-3Galb1-4(Fuca1-3)GlcNAcb1-2Mana-Sp0	-3	2	-62
535	GlcNAcb1-3Galb1-4GlcNAcb1-6(GlcNAcb1-3)Galb1-4GlcNAc-Sp0	3	5	133
536	GalNAca1-3(Fuca1-2)Galb1-3GalNAcb1-3Gala1-4Galb1-4Glc-Sp21	6	7	113
537	Gala1-3(Fuca1-2)Galb1-3GalNAcb1-3Gala1-4Galb1-4Glc-Sp21	9	8	90
538	Galb1-3GalNAcb1-3Gal-Sp21	9	4	42
539	GlcNAcb1-3Galb1-4GlcNAcb1-2Mana1-6(GlcNAcb1-3Galb1-4GlcNAcb1-2Mana1-3)Manb1-4GlcNAcb1-4GlcNAcb-Sp12	10	4	41
540	GlcNAcb1-3Galb1-4GlcNAcb1-2Mana1-6(GlcNAcb1-3Galb1-4GlcNAcb1-2Mana1-3)Manb1-4GlcNAcb1-4GlcNAcb-Sp25	13	10	76
541	Galb1-4GlcNAcb1-3Galb1-4GlcNAcb1-2Mana1-6(Galb1-4GlcNAcb1-3Galb1-4GlcNAcb1-2Mana1-3)Manb1-4GlcNAcb1-4GlcNAcb-Sp12	12	6	53
542	Galb1-4GlcNAcb1-3Galb1-4GlcNAcb1-2Mana1-6(Galb1-4GlcNAcb1-3Galb1-4GlcNAcb1-2Mana1-3)Manb1-4GlcNAcb1-4GlcNAcb-Sp24	6	12	215
543	Neu5Gca2-3Galb1-4GlcNAcb1-3Galb1-4GlcNAcb1-2Mana1-6(Neu5Gca2-3Galb1-4GlcNAcb1-3Galb1-4GlcNAcb1-2Mana1-3)Manb1-4GlcNAcb1-4GlcNAcb-Sp24	14	12	86
544	Fuca1-2Galb1-4GlcNAcb1-3Galb1-4GlcNAcb1-2Mana1-6(Fuca1-2Galb1-4GlcNAcb1-3Galb1-4GlcNAcb1-2Mana1-3)Manb1-4GlcNAcb1-4GlcNAcb-Sp24	8	9	120
545	GlcNAcb1-3Galb1-4GlcNAcb1-3Galb1-4GlcNAcb1-2Mana1-6(GlcNAcb1-3Galb1-4GlcNAcb1-3Galb1-4GlcNAcb1-2Mana1-3)Manb1-4GlcNAcb1-4GlcNAcb-Sp12	9	4	40
546	GlcNAcb1-3Galb1-4GlcNAcb1-3Galb1-4GlcNAcb1-2Mana1-6(GlcNAcb1-3Galb1-4GlcNAcb1-3Galb1-4GlcNAcb1-2Mana1-3)Manb1-4GlcNAcb1-4GlcNAcb-Sp25	5	8	171
547	Galb1-4GlcNAcb1-3Galb1-4GlcNAcb1-3Galb1-4GlcNAcb1-2Mana1-6(Galb1-4GlcNAcb1-3Galb1-4GlcNAcb1-3Galb1-4GlcNAcb1-2Mana1-3)Manb1-4GlcNAcb1-4GlcNAcb-Sp12	13	11	84
548	Galb1-4GlcNAcb1-3Galb1-4GlcNAcb1-3Galb1-4GlcNAcb1-2Mana1-6(Galb1-4GlcNAcb1-3Galb1-4GlcNAcb1-3Galb1-4GlcNAcb1-2Mana1-3)Manb1-4GlcNAcb1-4GlcNAcb-Sp24	5	11	222
549	GlcNAcb1-3Galb1-4GlcNAcb1-3Galb1-4GlcNAcb1-3Galb1-4GlcNAcb1-2Mana1-6(GlcNAcb1-3Galb1-4GlcNAcb1-3Galb1-4GlcNAcb1-3Galb1-4GlcNAcb1-2Mana1-3)Manb1-4GlcNAcb1-4GlcNAcb-Sp25	7	5	73
550	Galb1-4GlcNAcb1-3Galb1-4GlcNAcb1-3Galb1-4GlcNAcb1-3Galb1-4GlcNAcb1-2Mana1-6(Galb1-4GlcNAcb1-3Galb1-4GlcNAcb1-3Galb1-4GlcNAcb1-3Galb1-4GlcNAcb1-2Mana1-3)Manb1-4GlcNAcb1-4GlcNAcb-Sp25	11	11	108
551	Galb1-3GlcNAcb1-3Galb1-4GlcNAcb1-2Mana1-6(Galb1-3GlcNAcb1-3Galb1-4GlcNAcb1-2Mana1-3)Manb1-4GlcNAcb1-4GlcNAc-Sp25	4	1	25
552	Neu5Gca2-8Neu5Gca2-3Galb1-4GlcNAc-Sp0	7	6	84
553	Neu5Aca2-8Neu5Gca2-3Galb1-4GlcNAc-Sp0	1	6	475
554	Neu5Gca2-8Neu5Aca2-3Galb1-4GlcNAc-Sp0	7	8	114
555	Neu5Gca2-8Neu5Gca2-3Galb1-4GlcNAcb1-3Galb1-4GlcNAc-Sp0	8	11	131
556	Neu5Gca2-8Neu5Gca2-6Galb1-4GlcNAc-Sp0	0	2	630
557	Neu5Aca2-8Neu5Aca2-3Galb1-4GlcNAc-Sp0	7	9	124
558	GlcNAcb1-3Galb1-4GlcNAcb1-6(GlcNAcb1-3Galb1-4GlcNAcb1-2)Mana1-6(GlcNAcb1-3Galb1-4GlcNAcb1-2Man a1-3)Manb1-4GlcNAcb1-4GlcNAc-Sp24	6	6	109
559	Galb1-4GlcNAcb1-3Galb1-4GlcNAcb1-6(Galb1-4GlcNAcb1-3Galb1-4GlcNAcb1-2)Mana1-6(Galb1-4GlcNAcb1-3Galb1-4GlcNAcb1-2Mana1-3)Mana1-4GlcNAcb1-4GlcNAc-Sp24	17	15	91
560	Gala1-3Galb1-4GlcNAcb1-2Mana1-6(Gala1-3Galb1-4GlcNAcb1-2Mana1-3)Manb1-4GlcNAcb1-4GlcNAc-Sp24	9	9	104
561	GlcNAcb1-3Galb1-4GlcNAcb1-6(GlcNAcb1-3Galb1-3)GalNAca-Sp14	3	3	86
562	GalNAcb1-3GlcNAcb-Sp0	16	12	75
563	GalNAcb1-4GlcNAcb1-3GalNAcb1-4GlcNAcb-Sp0	0	2	629
564	GlcNAcb1-3Galb1-4GlcNAcb1-3Galb1-4GlcNAcb1-3Galb1-4GlcNAcb1-3Galb1-4GlcNAcb1-2Mana1-6(GlcNAcb1-3Galb1-4GlcNAcb1-3Galb1-4GlcNAcb1-3Galb1-4GlcNAcb1-3Galb1-4GlcNAcb1-2Mana1-3)Manb1-4GlcNAcb1-4GlcNAcb-Sp25	7	2	27
565	Galb1-4GlcNAcb1-3Galb1-4GlcNAcb1-3Galb1-4GlcNAcb1-3Galb1-4GlcNAcb1-3Galb1-4GlcNAcb1-2Mana1-6(Galb1-4GlcNAcb1-3Galb1-4GlcNAcb1-3Galb1-4GlcNAcb1-3Galb1-4GlcNAcb1-3Galb1-4GlcNAcb1-2Mana1-3)Manb1-4GlcNAcb1-4GlcNAcb-Sp25	3	2	52
566	GlcNAb1-3Galb1-3GalNAc-Sp14	7	1	13
567	Galb1-3GlcNAcb1-6(Galb1-3)GalNAc-Sp14	19	17	90
568	Galb1-4GlcNAcb1-3Galb1-4GlcNAcb1-3Galb1-4GlcNAcb1-3Galb1-4GlcNAcb1-3Galb1-4GlcNAcb1-3Galb1-4GlcNAcb1-2Mana1-6(Galb1-4GlcNAcb1-3Galb1-4GlcNAcb1-3Galb1-4GlcNAcb1-3Galb1-4GlcNAcb1-3Galb1-4GlcNAcb1-3Galb1-4GlcNAcb1-2Mana1-3)Manb1-4GlcNAcb1-4GlcNAcb-Sp25	13	10	78
569	(3S)GlcAb1-3Galb1-4GlcNAcb1-3Galb1-4Glc-Sp0	-1	2	-375
570	(3S)GlcAb1-3Galb1-4GlcNAcb1-2Mana-Sp0	11	6	58
571	Galb1-3GlcNAcb1-3Galb1-4GlcNAcb1-3Galb1-4GlcNAcb1-6(Galb1-3GlcNAcb1-3Galb1-4GlcNAcb1-3Galb1-4GlcNAb1-2)Mana1-6(Galb1-3GlcNAcb1-3Galb1-4GlcNAcb1-3Galb1-4GlcNAcb1-2Mana1-3)Manb1-4GlcNAcb1-4(Fuca1-6)GlcNAcb-Sp24	3	2	69
572	Galb1-3GlcNAcb1-3Galb1-4GlcNAcb1-6(Galb1-3GlcNAcb1-3Galb1-4GlcNAb1-2)Mana1-6(Galb1-3GlcNAcb1-3Galb1-4GlcNAcb1-2Mana1-3)Manb1-4GlcNAcb1-4(Fuca1-6)GlcNAcb-Sp24	8	8	109
573	Neu5Aca2-8Neu5Aca2-3Galb1-3GalNAcb1-4(Neu5Aca2-3)Galb1-4Glc-Sp21	11	2	21
574	GlcNAcb1-3Galb1-4GlcNAcb1-2Mana1-6(GlcNAcb1-3Galb1-4GlcNAcb1-2Mana1-3)Manb1-4GlcNAcb1-4(Fuca1-6)GlcNAcb-Sp24	11	7	66
575	Galb1-4GlcNAcb1-3Galb1-4GlcNAcb1-2Mana1-6(Galb1-4GlcNAcb1-3Galb1-4GlcNAcb1-2Mana1-3)Manb1-4GlcNAcb1-4(Fuca1-6)GlcNAcb-Sp24	25	13	51
576	GlcNAcb1-3Galb1-4GlcNAcb1-3Galb1-4GlcNAcb1-2Mana1-6(GlcNAcb1-3Galb1-4GlcNAcb1-3Galb1-4GlcNAcb1-2Mana1-3)Manb1-4GlcNAcb1-4(Fuca1-6)GlcNAcb-Sp24	2	7	318
577	Galb1-4GlcNAcb1-3Galb1-4GlcNAcb1-3Galb1-4GlcNAcb1-2Mana1-6(Galb1-4GlcNAcb1-3Galb1-4GlcNAcb1-3Galb1-4GlcNAcb1-2Mana1-3)Manb1-4GlcNAcb1-4(Fuca1-6)GlcNAcb-Sp24	11	3	31
578	GlcNAcb1-3Galb1-4GlcNAcb1-3Galb1-4GlcNAcb1-3Galb1-4GlcNAcb1-2Mana1-6(GlcNAcb1-3Galb1-4GlcNAcb1-3Galb1-4GlcNAcb1-3Galb1-4GlcNAcb1-2Mana1-3)Manb1-4GlcNAcb1-4(Fuca1-6)GlcNAcb-Sp24	11	5	44
579	Galb1-4GlcNAcb1-3Galb1-4GlcNAcb1-3Galb1-4GlcNAcb1-3Galb1-4GlcNAcb1-2Mana1-6(Galb1-4GlcNAcb1-3Galb1-4GlcNAcb1-3Galb1-4GlcNAcb1-3Galb1-4GlcNAcb1-2Mana1-3)Manb1-4GlcNAcb1-4(Fuca1-6)GlcNAcb-Sp24	8	5	60
580	GlcNAcb1-3Galb1-4GlcNAcb1-3Galb1-4GlcNAcb1-3Galb1-4GlcNAcb1-3Galb1-4GlcNAcb1-2Mana1-6(GlcNAcb1-3Galb1-4GlcNAcb1-3Galb1-4GlcNAcb1-3Galb1-4GlcNAcb1-3Galb1-4GlcNAcb1-2Mana1-3)Manb1-4GlcNAcb1-4(Fuca1-6)GlcNAcb-Sp19	11	4	37
581	Galb1-4GlcNAcb1-3Galb1-4GlcNAcb1-3Galb1-4GlcNAcb1-3Galb1-4GlcNAcb1-3Galb1-4GlcNAcb1-2Mana1-6(Galb1-4GlcNAcb1-3Galb1-4GlcNAcb1-3Galb1-4GlcNAcb1-3Galb1-4GlcNAcb1-3Galb1-4GlcNAcb1-2Mana1-3)Manb1-4GlcNAcb1-4(Fuca1-6)GlcNAcb-Sp19	22	17	79
582	Galb1-4GlcNAcb1-3Galb1-4GlcNAcb1-6(Galb1-4GlcNAcb1-3Galb1-4GlcNAb1-2)Mana1-6(Galb1-4GlcNAcb1-3Galb1-4GlcNAcb1-2Mana1-3)Manb1-4GlcNAcb1-4(Fuca1-6)GlcNAcb-Sp24	15	13	83

287	Neu5Gca-Sp8	2	3	153
604	GlcNAcb1-6(Neu5Aca2-3Galb1-3)GalNAca-Sp14	2	6	286
289	Galb1-3GlcNAcb1-3Galb1-3GlcNAcb-Sp0	2	4	173
256	Neu5Aca2-3Galb1-4(Fuca1-3)GlcNAcb1-3Galb-Sp8	2	5	234
63	Fuca1-2Galb1-3GalNAcb1-4(Neu5Aca2-3)Galb1-4Glcb-Sp0	2	6	287
253	Neu5Aca2-3Galb1-4(Fuca1-3)GlcNAcb1-3Galb1-4(Fuca1-3)GlcNAcb1-3Galb1-4(Fuca1-3)GlcNAcb-Sp0	2	2	82
83	GalNAca1-3(Fuca1-2)Galb1-4(Fuca1-3)GlcNAcb-Sp0	2	4	206
58	Fuca1-2Galb1-3GalNAcb1-3Gala-Sp9	2	2	78
576	GlcNAcb1-3Galb1-4GlcNAcb1-3Galb1-4GlcNAcb1-2Mana1-6(GlcNAcb1-3Galb1-4GlcNAcb1-3Galb1-4GlcNAcb1-2Mana1-3)Manb1-4GlcNAcb1-4(Fuca1-6)GlcNAcb-Sp24	2	7	318
154	Galb1-4(Fuca1-3)GlcNAcb1-3Galb1-4(Fuca1-3)GlcNAcb1-3Galb1-4(Fuca1-3)GlcNAcb-Sp0	2	9	429
402	GalNAca1-3GalNAcb1-3Gala1-4Galb1-4GlcNAcb-Sp0	2	5	256
81	Fucb1-3GlcNAcb-Sp8	2	4	175
369	Galb1-4GlcNAcb1-2Mana1-6(Galb1-4GlcNAcb1-4(Galb1-4GlcNAcb1-2)Mana1-3)Manb1-4GlcNAcb1-4GlcNAc-Sp21	2	6	285
53	GlcNAcb1-2Mana1-6(GlcNAcb1-2Mana1-3)Manb1-4GlcNAcb1-4GlcNAcb-Sp13	2	4	200
285	Neu5Gca2-6GalNAca-Sp0	2	7	365
390	Fuca1-2Galb1-3GalNAca1-3(Fuca1-2)Galb1-4GlcNAcb-Sp0	2	6	344
279	Neu5Acb2-6Galb1-4GlcNAcb-Sp8	2	4	209
185	GlcNAcb1-3Galb1-4Glcb-Sp0	2	5	264
186	GlcNAcb1-4-MDPLys	2	7	390
364	Galb1-4GlcNAcb1-2Mana1-6(Mana1-3)Manb1-4GlcNAcb1-4GlcNAcb-Sp12	2	6	343
444	(6S)Galb1-3(6S)GlcNAc-Sp0	2	3	154
168	Galb1-4GlcNAcb-Sp0	2	4	237
215	Mana1-6(Mana1-3)Mana1-6(Mana1-2Mana1-3)Manb1-4GlcNAcb1-4GlcNAcb-Sp12	2	4	265
224	Neu5Aca2-3Galb1-3GalNAca-Sp14	1	2	163
97	GalNAcb1-4(Fuca1-3)GlcNAcb-Sp0	1	3	227
41	(6P)Mana-Sp8	1	3	200
254	Neu5Aca2-3Galb1-4(Fuca1-3)GlcNAcb-Sp0	1	2	138
51	Mana1-6(Mana1-3)Manb1-4GlcNAcb1-4GlcNAcb-Sp13	1	5	338
553	Neu5Aca2-8Neu5Gca2-3Galb1-4GlcNAc-Sp0	1	6	475
326	Neu5Aca2-3Galb1-4GlcNAcb1-2Mana1-6(Neu5Aca2-6Galb1-4GlcNAcb1-2Mana1-3)Manb1-4GlcNAcb1-4GlcNAcb-Sp12	1	7	496
226	GalNAcb1-4(Neu5Aca2-8Neu5Aca2-8Neu5Aca2-3)Galb1-4Glcb-Sp0	1	5	408
234	Neu5Aca2-3Galb1-3GalNAcb1-4(Neu5Aca2-3)Galb1-4Glcb-Sp0	1	4	298
406	Galb1-3GlcNAcb1-6Galb1-4GlcNAcb-Sp0	1	6	511
339	GlcNAca1-4Galb1-4GlcNAcb-Sp0	1	2	199
7	Fuca-Sp9	1	4	360
511	(6S)(4S)GalNAcb1-4GlcNAc-Sp8	1	5	431
276	Neu5Aca2-8Neu5Aca2-3Galb1-4Glcb-Sp0	1	3	240
148	Galb1-3GlcNAcb1-3Galb1-4Glcb-Sp10	1	4	421
172	Galb1-4Glcb-Sp8	1	3	242
198	Glcb1-4Glcb-Sp8	1	3	300
447	Galb1-4(Fuca1-3)GlcNAcb1-6GalNAc-Sp14	1	6	594
64	Fuca1-2Galb1-3GalNAcb1-4(Neu5Aca2-3)Galb1-4Glcb-Sp9	1	2	182
416	Fuca1-2Galb1-4(Fuca1-3)GlcNAcb1-3GalNAca-Sp14	1	2	254
136	Neu5Aca2-6(Galb1-3)GalNAca-Sp14	1	4	501
46	Neu5Aca2-3(6S)Galb1-4GlcNAcb-Sp8	1	9	1037
193	GlcNAcb1-6GalNAca-Sp14	1	7	1080
171	Galb1-4Glcb-Sp0	1	0	33
381	Galb1-3GalNAca1-3(Fuca1-2)Galb1-4GlcNAc-Sp0	1	3	560
464	Neu5Aca2-6Galb1-4GlcNAcb1-4Mana1-6(GlcNAcb1-4)(Neu5Aca2-6Galb1-4GlcNAcb1-4(Neu5Aca2-6Galb1-4GlcNAcb1-2)Mana1-3)Manb1-4GlcNAcb1-4GlcNAcb-Sp21	1	2	281
498	Gala1-3(Fuca1-2)Galb1-4GlcNAcb1-6GalNAca-Sp14	0	3	711
556	Neu5Gca2-8Neu5Gca2-6Galb1-4GlcNAc-Sp0	0	2	630
145	Galb1-3GalNAcb1-4Galb1-4Glcb-Sp8	0	3	852
563	GalNAcb1-4GlcNAcb1-3GalNAcb1-4GlcNAcb-Sp0	0	2	629
232	GalNAcb1-4(Neu5Aca2-3)Galb1-4GlcNAcb-Sp8	0	1	419
496	Galb1-4(Fuca1-3)GlcNAcb1-2Mana-Sp0	0	4	1592
533	Fuca1-4(Galb1-3)GlcNAcb1-2 Mana-Sp0	0	3	1264
21	GlcNAcb1-6(GlcNAcb1-4)(GlcNAcb1-3)GlcNAc-Sp8	0	9	4333
39	(6S)(4S)Galb1-4GlcNAcb-Sp0	0	2	-20578
34	(3S)Galb1-4(6S)GlcNAcb-Sp0	0	3	-12877
182	GlcNAcb1-3Galb1-4GlcNAcb-Sp0	0	5	-8348
418	GalNAca1-3(Fuca1-2)Galb1-4(Fuca1-3)GlcNAcb1-3GalNAc-Sp14	0	6	-6969
322	Neu5Aca2-8Neu5Aca2-8Neu5Acb-Sp8	0	6	-4791
243	Neu5Aca2-6(Neu5Aca2-3Galb1-3)GalNAca-Sp8	0	3	-1972
474	Neu5Aca2-3Galb1-3GlcNAcb1-6(Neu5Aca2-3Galb1-3GlcNAcb1-2)Mana1-6(Neu5Aca2-3Galb1-3GlcNAcb1-2Mana1-3)Manb1-4GlcNAcb1-4GlcNAcb-Sp19	0	6	-3711
73	Fuca1-2Galb1-4GlcNAcb1-3Galb1-4GlcNAcb-Sp0	0	3	-1275
401	Galb1-4(Fuca1-3)GlcNAcb1-3GalNAca-Sp14	0	5	-1667
315	Mana1-2Mana1-6(Mana1-3)Mana1-6(Mana1-2Mana1-2Mana1-3)Mana-Sp9	0	4	-1522
495	Gala1-3Galb1-4GlcNAcb1-6GalNAca-Sp14	0	2	-632
327	Galb1-4(Fuca1-3)GlcNAcb1-2Mana1-6(Galb1-4(Fuca1-3)GlcNAcb1-2Mana1-3)Manb1-4GlcNAcb1-4GlcNAcb-Sp20	0	4	-970
273	Neu5Aca2-6Galb1-4Glcb-Sp8	0	2	-421
333	Gala1-4Galb1-4GlcNAcb1-3Galb1-4Glcb-Sp0	0	3	-812
189	GlcNAcb1-4GlcNAcb1-4GlcNAcb1-4GlcNAcb1-4GlcNAcb1-4GlcNAcb1-Sp8	0	4	-1037
417	Gala1-3(Fuca1-2)Galb1-4(Fuca1-3)GlcNAcb1-3GalNAc-Sp14	0	6	-1303
312	MurNAcb1-4GlcNAcb-Sp10	-1	6	-959
569	(3S)GlcAb1-3Galb1-4GlcNAcb1-3Galb1-4Glc-Sp0	-1	2	-375
305	Galb1-4GlcNAcb1-6Galb1-4GlcNAcb-Sp0	-1	8	-1168
304	Galb1-4GlcNAca1-6Galb1-4GlcNAcb-Sp0	-1	4	-596
503	GalNAcb1-4(Fuca1-3)(6S)GlcNAcb-Sp8	-1	4	-632
602	Neu5Aca2-6Galb1-4GlcNAcb1-6(Galb1-3)GalNAca-Sp14	-1	5	-579
252	Neu5Aca2-3Galb1-4(Fuca1-3)(6S)GlcNAcb-Sp8	-1	4	-544
196	Glca1-4Glca-Sp8	-1	3	-399
408	GalNAcb1-3Gala1-6Galb1-4Glcb-Sp8	-1	6	-745
356	KDNa2-3Galb1-4(Fuca1-3)GlcNAc-Sp0	-1	3	-291
517	Galb1-4(6P)GlcNAcb-Sp0	-1	4	-403

583	GlcNAcb1-3Galb1-4GlcNAcb1-3Galb1-4GlcNAcb1-6(GlcNAcb1-3Galb1-4GlcNAcb1-3Galb1-4GlcNAb1-2)Mana1-6(GlcNAcb1-3Galb1-4GlcNAcb1-3Galb1-4GlcNAcb1-2Mana1-3)Manb1-4GlcNAcb1-4(Fuca1-6)GlcNAcb-Sp24	14	10	71
584	Galb1-4GlcNAcb1-3Galb1-4GlcNAcb1-3Galb1-4GlcNAcb1-6(Galb1-4GlcNAcb1-3Galb1-4GlcNAcb1-3Galb1-4GlcNAb1-2)Mana1-6(Galb1-4GlcNAcb1-3Galb1-4GlcNAcb1-3Galb1-4GlcNAcb1-2Mana1-3)Manb1-4GlcNAcb1-4(Fuca1-6)GlcNAcb-Sp24	8	8	99
585	GlcNAcb1-3Galb1-4GlcNAcb1-3Galb1-4GlcNAcb1-3Galb1-4GlcNAcb1-6(GlcNAcb1-3Galb1-4GlcNAcb1-3Galb1-4GlcNAcb1-3Galb1-4GlcNAb1-2)Mana1-6(GlcNAcb1-3Galb1-4GlcNAcb1-3Galb1-4GlcNAcb1-3Galb1-4GlcNAcb1-2Mana1-3)Manb1-4GlcNAcb1-4(Fuca1-6)GlcNAcb-Sp24	5	4	74
586	Galb1-4GlcNAcb1-3Galb1-4GlcNAcb1-3Galb1-4GlcNAcb1-3Galb1-4GlcNAcb1-6(Galb1-4GlcNAcb1-3Galb1-4GlcNAcb1-3Galb1-4GlcNAcb1-3Galb1-4GlcNAb1-2)Mana1-6(Galb1-4GlcNAcb1-3Galb1-4GlcNAcb1-3Galb1-4GlcNAcb1-3Galb1-4GlcNAcb1-2Mana1-3)Manb1-4GlcNAcb1-4(Fuca1-6)GlcNAcb-Sp24	13	10	81
587	GlcNAcb1-3Galb1-4GlcNAcb1-3Galb1-4GlcNAcb1-3Galb1-4GlcNAcb1-3Galb1-4GlcNAcb1-6(GlcNAcb1-3Galb1-4GlcNAcb1-3Galb1-4GlcNAcb1-3Galb1-4GlcNAcb1-3Galb1-4GlcNAb1-2)Mana1-6(GlcNAcb1-3Galb1-4GlcNAcb1-3Galb1-4GlcNAcb1-3Galb1-4GlcNAcb1-3Galb1-4GlcNAcb1-2Mana1-3)Manb1-4GlcNAcb1-4(Fuca1-6)GlcNAcb-Sp24	11	4	34
588	Galb1-4GlcNAcb1-3Galb1-4GlcNAcb1-3Galb1-4GlcNAcb1-3Galb1-4GlcNAcb1-3Galb1-4GlcNAcb1-6(Galb1-4GlcNAcb1-3Galb1-4GlcNAcb1-3Galb1-4GlcNAcb1-3Galb1-4GlcNAcb1-3Galb1-4GlcNAb1-2)Mana1-6(Galb1-4GlcNAcb1-3Galb1-4GlcNAcb1-3Galb1-4GlcNAcb1-3Galb1-4GlcNAcb1-3Galb1-4GlcNAcb1-2Mana1-3)Manb1-4GlcNAcb1-4(Fuca1-6)GlcNAcb-Sp24	13	3	26
589	Galb1-4GlcNAcb1-3Galb1-4GlcNAcb1-3GalNAca-Sp14	6	2	32
590	Galb1-4GlcNAcb1-3Galb1-4GlcNAcb1-6(Galb1-3)GalNAca-Sp14	-3	5	-164
591	Galb1-4GlcNAcb1-3Galb1-4GlcNAcb1-6(Galb1-4GlcNAcb1-3Galb1-4GlcNAcb1-3)GalNAca-Sp14	8	1	17
592	Neu5Aca2-3Galb1-4GlcNAcb1-3Galb1-4GlcNAcb1-3GalNAca-Sp14	9	8	90
593	GlcNAcb1-3Galb1-4GlcNAcb1-3GalNAca-Sp14	4	4	83
594	GlcNAcb1-3Galb1-4GlcNAcb1-6(Galb1-3)GalNAca-Sp14	13	9	70
595	GlcNAcb1-3Galb1-4GlcNAcb1-6(GlcNAcb1-3Galb1-4GlcNAcb1-3)GalNAca-Sp14	13	14	105
596	Neu5Aca2-3Galb1-4GlcNAcb1-3Galb1-4GlcNAcb1-6(Neu5Aca2-3Galb1-4GlcNAcb1-3Galb1-4GlcNAcb1-3)GalNAca-Sp14	4	6	176
597	Neu5Aca2-6Galb1-4GlcNAcb1-3Galb1-4GlcNAcb1-3GalNAca-Sp14	2	2	102
598	GlcNAcb1-3Galb1-4GlcNAcb1-3Galb1-4GlcNAcb1-3GalNAca-Sp14	9	6	70
599	Galb1-4GlcNAcb1-3Galb1-3GalNAca-Sp14	10	9	89
600	Neu5Aca2-3Galb1-4GlcNAcb1-3Galb1-4GlcNAcb1-6(Galb1-3)GalNAca-Sp14	8	9	111
601	Neu5Aca2-6Galb1-4GlcNAcb1-3Galb1-4GlcNAcb1-6(Galb1-3)GalNAca-Sp14	4	4	87
602	Neu5Aca2-6Galb1-4GlcNAcb1-6(Galb1-3)GalNAca-Sp14	-1	5	-579
603	Neu5Aca2-3Galb1-4GlcNAcb1-3Galb1-4GlcNAcb1-2Mana1-6(Neu5Aca2-3Galb1-4GlcNAcb1-3Galb1-4GlcNAcb1-2Mana1-3)Manb1-4GlcNAcb1-4GlcNAcb-Sp12	12	7	61
604	GlcNAcb1-6(Neu5Aca2-3Galb1-3)GalNAca-Sp14	2	6	286
605	Neu5Aca2-6Galb1-4GlcNAcb1-3Galb1-4GlcNAcb1-6(Neu5Aca2-6Galb1-4GlcNAcb1-3Galb1-4GlcNAcb1-3)GalNAca-Sp14	8	5	68
606	Neu5Aca2-6Galb1-4GlcNAcb1-3Galb1-4GlcNAcb1-3Galb1-4GlcNAcb1-2Mana1-6(Neu5Aca2-6Galb1-4GlcNAcb1-3Galb1-4GlcNAcb1-3Galb1-4GlcNAcb1-2Mana1-3)Manb1-4GlcNAcb1-4GlcNAcb-Sp12	10	12	120
607	Neu5Aca2-3Galb1-4GlcNAcb1-3Galb1-4GlcNAcb1-3Galb1-4GlcNAcb1-2Mana1-6(Neu5Aca2-3Galb1-4GlcNAcb1-3Galb1-4GlcNAcb1-3Galb1-4GlcNAcb1-2Mana1-3)Manb1-4GlcNAcb1-4GlcNAcb-Sp12	5	6	107
608	Neu5Aca2-6Galb1-4GlcNAcb1-3Galb1-4GlcNAcb1-2Mana1-6(Neu5Aca2-6Galb1-4GlcNAcb1-3Galb1-4GlcNAcb1-2Mana1-3)Manb1-4GlcNAcb1-4GlcNAcb-Sp12	6	6	94
609	GlcNAcb1-3Fuca-Sp21	13	11	82
610	Galb1-3GalNAcb1-4(Neu5Aca2-8Neu5Aca2-8Neu5Aca2-3)Galb1-4Glcb-Sp21	10	1	7

26	(3S)Galb1-4(6S)Glcb-Sp0	-1	4	-393
345	Neu5Aca2-6Galb1-4GlcNAcb1-2Mana1-6(Mana1-3)Manb1-4GlcNAcb1-4GlcNAc-Sp12	-1	3	-254
487	Neu5Aca2-6Galb1-4GlcNAcb1-6(Fuca1-2Galb1-4(Fuca1-3)GlcNAcb1-3)Galb1-4Glc-Sp21	-1	4	-312
5	GalNAca-Sp15	-1	6	-486
212	Mana1-2Mana1-6(Mana1-2Mana1-3)Mana1-6(Mana1-2Mana1-2Mana1-3)Manb1-4GlcNAcb1-4GlcNAcb-Sp12	-1	4	-291
302	Galb1-4GlcNAcb1-6(Galb1-4GlcNAcb1-3)Galb1-4GlcNAc-Sp0	-1	3	-243
230	Neu5Aca2-3(6S)Galb1-4(Fuca1-3)GlcNAcb-Sp8	-1	4	-276
23	6S(3S)Galb1-4GlcNAcb-Sp0	-1	2	-117
157	Galb1-4GalNAca1-3(Fuca1-2)Galb1-4GlcNAcb-Sp8	-1	5	-331
506	GalNAca1-3(Fuca1-2)Galb1-3GlcNAcb1-6GalNAca-Sp14	-2	4	-247
17	GlcNAcb-Sp8	-2	4	-240
419	Galb1-4(Fuca1-3)GlcNAcb1-2Mana1-6(Galb1-4(Fuca1-3)GlcNAcb1-2Mana1-3)Manb1-4GlcNAcb1-4(Fuca1-6)GlcNAcb-Sp22	-2	4	-225
147	Galb1-3GlcNAcb1-3Galb1-4GlcNAcb-Sp0	-2	1	-65
67	Fuca1-2Galb1-3GlcNAcb-Sp0	-2	2	-103
184	GlcNAcb1-3Galb1-4GlcNAcb1-3Galb1-4GlcNAcb-Sp0	-2	7	-307
292	Galb1-4(Fuca1-3)GlcNAcb1-3Galb1-3(Fuca1-4)GlcNAcb-Sp0	-2	2	-82
450	Gala1-3(Fuca1-2)Galb1-4GlcNAcb1-6(Gala1-3(Fuca1-2)Galb1-4GlcNAcb1-3)GalNAc-Sp14	-3	2	-59
590	Galb1-4GlcNAcb1-3Galb1-4GlcNAcb1-6(Galb1-3)GalNAca-Sp14	-3	5	-164
534	Neu5Aca2-3Galb1-4(Fuca1-3)GlcNAcb1-2Mana-Sp0	-3	2	-62
284	Neu5Gca2-3Galb1-4Glcb-Sp0	-3	8	-224
247	Neu5Aca2-3Galb1-3GlcNAcb1-3Galb1-4GlcNAcb-Sp0	-4	4	-100
70	Fuca1-2Galb1-4(Fuca1-3)GlcNAcb1-3Galb1-4(Fuca1-3)GlcNAcb1-3Galb1-4(Fuca1-3)GlcNAcb-Sp0	-4	5	-122
258	Neu5Aca2-3Galb1-4GlcNAcb1-3Galb1-4GlcNAcb1-3Galb1-4GlcNAcb-Sp0	-5	7	-141
296	4S(3S)Galb1-4GlcNAcb-Sp0	-5	3	-61
282	Neu5Gca2-3Galb1-4(Fuca1-3)GlcNAcb-Sp0	-5	8	-142
260	Neu5Aca2-3Galb1-4GlcNAcb-Sp8	-5	12	-213
93	GalNAca1-4(Fuca1-2)Galb1-4GlcNAcb-Sp8	-6	7	-119
427	Gala1-3(Fuca1-2)Galb1-4GlcNAcb1-2Mana1-6(Gala1-3(Fuca1-2)Galb1-4GlcNAcb1-2Mana1-3)Manb1-4GlcNAcb1-4(Fuca1-6)GlcNAcb-Sp22	-9	6	-68

Chart ID		Average RFU	StDev	%CV
1	Gala-Sp8	2480	1966	79
2	Glca-Sp8	261	86	33
3	Mana-Sp8	129	16	13
4	GalNAca-Sp8	66	7	10
5	GalNAca-Sp15	118	23	19
6	Fuca-Sp8	143	38	27
7	Fuca-Sp9	88	11	12
8	Rhaa-Sp8	133	12	9
9	Neu5Aca-Sp8	75	9	12
10	Neu5Aca-Sp11	24	4	19
11	Neu5Acb-Sp8	39	1	2
12	Galb-Sp8	72	10	14
13	Glcb-Sp8	112	7	6
14	Manb-Sp8	105	9	8
15	GalNAcb-Sp8	82	14	17
16	GlcNAcb-Sp0	165	20	12
17	GlcNAcb-Sp8	78	30	38
18	GlcN(Gc)b-Sp8	120	44	37
19	Galb1-4GlcNAcb1-6(Galb1-4GlcNAcb1-3)GalNAca-Sp8	55	7	12
20	Galb1-4GlcNAcb1-6(Galb1-4GlcNAcb1-3)GalNAc-Sp14	76	12	16
21	GlcNAcb1-6(GlcNAcb1-4)(GlcNAcb1-3)GlcNAc-Sp8	75	14	19
22	6S(3S)Galb1-4(6S)GlcNAcb-Sp0	58	2	3
23	6S(3S)Galb1-4GlcNAcb-Sp0	51	2	4
24	(3S)Galb1-4(Fuca1-3)(6S)Glc-Sp0	61	9	14
25	(3S)Galb1-4Glcb-Sp8	447	161	36
26	(3S)Galb1-4(6S)Glcb-Sp0	44	2	5
27	(3S)Galb1-4(6S)Glcb-Sp8	37	6	15
28	(3S)Galb1-3(Fuca1-4)GlcNAcb-Sp8	31	2	7
29	(3S)Galb1-3GalNAca-Sp8	41	2	4
30	(3S)Galb1-3GlcNAcb-Sp0	55	4	7
31	(3S)Galb1-3GlcNAcb-Sp8	40	4	9
32	(3S)Galb1-4(Fuca1-3)GlcNAc-Sp0	61	1	2
33	(3S)Galb1-4(Fuca1-3)GlcNAc-Sp8	31	4	13
34	(3S)Galb1-4(6S)GlcNAcb-Sp0	32	4	14
35	(3S)Galb1-4(6S)GlcNAcb-Sp8	43	6	13
36	(3S)Galb1-4GlcNAcb-Sp0	28	2	9
37	(3S)Galb1-4GlcNAcb-Sp8	28	5	18
38	(3S)Galb-Sp8	30	4	13
39	(6S)(4S)Galb1-4GlcNAcb-Sp0	27	3	11
40	(4S)Galb1-4GlcNAcb-Sp8	37	4	10
41	(6P)Mana-Sp8	19	13	69
42	(6S)Galb1-4Glcb-Sp0	151	94	62
43	(6S)Galb1-4Glcb-Sp8	37	6	15
44	(6S)Galb1-4GlcNAcb-Sp8	42	4	9
45	(6S)Galb1-4(6S)Glcb-Sp8	51	6	12
46	Neu5Aca2-3(6S)Galb1-4GlcNAcb-Sp8	58	4	8
47	(6S)GlcNAcb-Sp8	36	1	4
48	Neu5,9Ac$_2$a-Sp8	28	7	26
49	Neu5,9Ac2a2-6Galb1-4GlcNAcb-Sp8	209	26	13
50	Mana1-6(Mana1-3)Manb1-4GlcNAcb1-4GlcNAcb-Sp12	22	4	19
51	Mana1-6(Mana1-3)Manb1-4GlcNAcb1-4GlcNAcb-Sp13	19	4	19
52	GlcNAcb1-2Mana1-6(GlcNAcb1-2Mana1-3)Manb1-4GlcNAcb1-4GlcNAcb-Sp12	28	6	20
53	GlcNAcb1-2Mana1-6(GlcNAcb1-2Mana1-3)Manb1-4GlcNAcb1-4GlcNAcb-Sp13	22	2	10
54	Galb1-4GlcNAcb1-2Mana1-6(Galb1-4GlcNAcb1-2Mana1-3)Manb1-4GlcNAcb1-4GlcNAcb-Sp12	56	10	19
55	Neu5Aca2-6Galb1-4GlcNAcb1-2Mana1-6(Neu5Aca2-6Galb1-4GlcNAcb1-2Mana1-3)Manb1-4GlcNAcb1-4GlcNAcb-Sp12	38	1	3
56	Neu5Aca2-6Galb1-4GlcNAcb1-2Mana1-6(Neu5Aca2-6Galb1-4GlcNAcb1-2Man-a1-3)Manb1-4GlcNAcb1-4GlcNAcb-Sp21	25	1	5
57	Neu5Aca2-6Galb1-4GlcNAcb1-2Mana1-6(Neu5Aca2-6Galb1-4GlcNAcb1-2Mana1-3)Manb1-4GlcNAcb1-4GlcNAcb-Sp24	21	2	10
58	Fuca1-2Galb1-3GalNAcb1-3Gala-Sp9	36	4	10
59	Fuca1-2Galb1-3GalNAcb1-3Gala1-4Galb1-4Glcb-Sp9	21	3	13
60	Fuca1-2Galb1-3(Fuca1-4)GlcNAcb-Sp8	28	1	4
61	Fuca1-2Galb1-3GalNAca-Sp8	42	12	28
62	Fuca1-2Galb1-3GalNAca-Sp14	25	2	7
63	Fuca1-2Galb1-3GalNAcb1-4(Neu5Aca2-3)Galb1-4Glcb-Sp0	36	5	15
64	Fuca1-2Galb1-3GalNAcb1-4(Neu5Aca2-3)Galb1-4Glcb-Sp9	22	16	73
65	Fuca1-2Galb1-3GlcNAcb1-3Galb1-4Glcb-Sp8	19	43	226
66	Fuca1-2Galb1-3GlcNAcb1-3Galb1-4Glcb-Sp10	31	1	4
67	Fuca1-2Galb1-3GlcNAcb-Sp0	41	3	6
68	Fuca1-2Galb1-3GlcNAcb-Sp8	26	2	8
69	Fuca1-2Galb1-4(Fuca1-3)GlcNAcb1-3Galb1-4(Fuca1-3)GlcNAcb-Sp0	53	5	9
70	Fuca1-2Galb1-4(Fuca1-3)GlcNAcb1-3Galb1-4(Fuca1-3)GlcNAcb1-3Galb1-4(Fuca1-3)GlcNAcb-Sp0	45	3	7
71	Fuca1-2Galb1-4(Fuca1-3)GlcNAcb-Sp0	48	6	13
72	Fuca1-2Galb1-4(Fuca1-3)GlcNAcb-Sp8	158	38	24
73	Fuca1-2Galb1-4GlcNAcb1-3Galb1-4GlcNAcb-Sp0	20	3	13
74	Fuca1-2Galb1-4GlcNAcb1-3Galb1-4GlcNAcb1-3Galb1-4GlcNAcb-Sp0	21	4	18
75	Fuca1-2Galb1-4GlcNAcb-Sp0	18	2	8
76	Fuca1-2Galb1-4GlcNAcb-Sp8	28	5	18
77	Fuca1-2Galb1-4Glcb-Sp0	84	3	3
78	Fuca1-2Galb-Sp8	21	11	50
79	Fuca1-3GlcNAcb-Sp8	27	5	18
80	Fuca1-4GlcNAcb-Sp8	27	2	6
81	Fucb1-3GlcNAcb-Sp8	24	5	23
82	GalNAca1-3(Fuca1-2)Galb1-3GlcNAcb-Sp0	32	3	10
83	GalNAca1-3(Fuca1-2)Galb1-4(Fuca1-3)GlcNAcb-Sp0	39	5	14
84	(3S)Galb1-4(Fuca1-3)Glcb-Sp0	29	3	10
85	GalNAca1-3(Fuca1-2)Galb1-4GlcNAcb-Sp0	25	1	6
86	GalNAca1-3(Fuca1-2)Galb1-4GlcNAcb-Sp8	20	3	13
87	GalNAca1-3(Fuca1-2)Galb1-4Glcb-Sp0	42	8	18
88	GlcNAcb1-3Galb1-3GalNAca-Sp8	-1	1	-40
89	GalNAca1-3(Fuca1-2)Galb-Sp8	280	173	62
90	GalNAca1-3(Fuca1-2)Galb-Sp18	23	5	21
91	GalNAca1-3GalNAcb-Sp8	27	4	15
92	GalNAca1-3Galb-Sp8	19	2	11
93	GalNAca1-4(Fuca1-2)Galb1-4GlcNAcb-Sp8	53	9	18
94	GalNAcb1-3GalNAca-Sp8	40	1	1
95	GalNAcb1-3(Fuca1-2)Galb-Sp8	63	9	15
96	GalNAcb1-3Gala1-4Galb1-4GlcNAcb-Sp0	1227	500	41
97	GalNAcb1-4(Fuca1-3)GlcNAcb-Sp0	63	5	8
98	GalNAcb1-4GlcNAcb-Sp0	80	21	26
99	GalNAcb1-4GlcNAcb-Sp8	36	3	9
100	Gala1-2Galb-Sp8	269	140	52
101	Gala1-3(Fuca1-2)Galb1-3GlcNAcb-Sp0	28	2	6
102	Gala1-3(Fuca1-2)Galb1-3GlcNAcb-Sp8	33	2	6
103	Gala1-3(Fuca1-2)Galb1-4(Fuca1-3)GlcNAcb-Sp0	27	2	6
104	Gala1-3(Fuca1-2)Galb1-4(Fuca1-3)GlcNAcb-Sp8	46	2	5
105	Gala1-3(Fuca1-2)Galb1-4GlcNAc-Sp0	52	3	6
106	Gala1-3(Fuca1-2)Galb1-4Glcb-Sp0	28	9	32
107	Gala1-3(Fuca1-2)Galb-Sp8	35	4	13
108	Gala1-3(Fuca1-2)Galb-Sp18	61	5	8
109	Gala1-4(Gala1-3)Galb1-4GlcNAcb-Sp8	105	14	13
110	Gala1-3GalNAca-Sp8	62	8	13
111	Gala1-3GalNAca-Sp16	21	2	10
112	Gala1-3GalNAcb-Sp8	35	3	7
113	Gala1-3Galb1-4(Fuca1-3)GlcNAcb-Sp8	29	6	20
114	Gala1-3Galb1-3GlcNAcb-Sp0	43	2	4
115	Gala1-3Galb1-4GlcNAcb-Sp8	1	2	177
116	Gala1-3Galb1-4Glcb-Sp0	168	45	27
117	Gala1-3Galb1-4Glc-Sp10	31	2	6
118	Gala1-3Galb-Sp8	36	23	63
119	Gala1-4(Fuca1-2)Galb1-4GlcNAcb-Sp8	239	44	19
120	Gala1-4Galb1-4GlcNAcb-Sp0	37	9	25

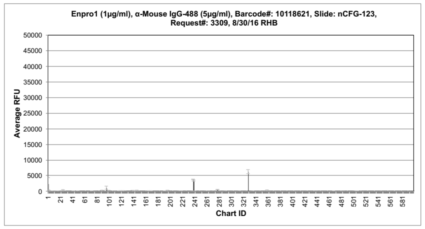

Chart ID	Enpro1 (1µg/ml), α-Mouse IgG-488 (5µg/ml), Barcode#: 10118621, Slide: nCFG-123, Request#: 3309, 8/30/16 RHB	Average RFU	StDev	%CV
328	Neu5Aca2-3Galb1-3(Fuca1-4)GlcNAcb1-3Galb1-3(Fuca1-4)GlcNAcb-Sp0	6351	661	10
238	Neu5Aca2-3Galb1-3(Fuca1-4)GlcNAcb-Sp8	3923	69	2
239	Neu5Aca2-3Galb1-3(Fuca1-4)GlcNAcb1-3Galb1-4(Fuca1-3)GlcNAcb-Sp0	3471	198	6
1	Gala-Sp8	2480	1966	79
96	GalNAcb1-3Gala1-4Galb1-4GlcNAcb-Sp0	1227	500	41
278	Neu5Gca2-3Galb1-3(Fuca1-4)GlcNAcb-Sp0	634	30	5
25	(3S)Galb1-4Glcb-Sp8	447	161	36
146	Galb1-3Galb-Sp8	326	124	38
196	Glca1-4Glca-Sp8	289	147	51
142	Galb1-3GalNAcb-Sp8	281	30	11
89	GalNAca1-3(Fuca1-2)Galb-Sp8	280	173	62
100	Gala1-2Galb-Sp8	269	140	52
2	Glca-Sp8	261	86	33
139	Galb1-3GalNAca-Sp8	247	147	60
359	Fuca1-2Galb1-4(Fuca1-3)GlcNAcb1-2Mana1-6(Fuca1-2Galb1-4(Fuca1-3)GlcNAcb1-2Mana1-3)Manb1-4GlcNAcb1-4GlcNAb-Sp20	242	295	122
119	Gala1-4(Fuca1-2)Galb1-4GlcNAcb-Sp8	239	44	19
123	Gala1-4GlcNAcb-Sp8	214	71	33
192	GlcNAcb1-6GalNAca-Sp8	210	36	17
49	Neu5,9Ac2a2-6Galb1-4GlcNAcb-Sp8	209	26	13
169	Galb1-4GlcNAcb-Sp8	192	61	32
165	Galb1-4GlcNAcb1-3Galb1-4Glcb-Sp8	172	88	51
116	Gala1-3Galb1-4Glcb-Sp0	168	45	27
16	GlcNAcb-Sp0	165	20	12
72	Fuca1-2Galb1-4(Fuca1-3)GlcNAcb-Sp8	158	38	24
507	(6S)GalNAcb1-4GlcNAc-Sp8	156	5	3
42	(6S)Galb1-4Glcb-Sp0	151	94	62
241	Neu5Aca2-3Galb1-3(6S)GalNAca-Sp8	143	23	16
6	Fuca-Sp8	143	38	27
468	Fuca1-2Galb1-4(Fuca1-3)GlcNAcb1-2Mana1-6(Fuca1-2Galb1-4(Fuca1-3)GlcNAcb1-2Mana1-3)Manb1-4GlcNAcb1-4(Fuca1-6)GlcNAcb-Sp24	138	78	57
8	Rhaa-Sp8	133	12	9
3	Mana-Sp8	129	16	13
219	(3S)Galb1-4(Fuca1-3)(6S)GlcNAcb-Sp8	126	16	13
237	Neu5Aca2-3Galb1-3(6S)GlcNAc-Sp8	120	51	43
18	GlcN(Gc)b-Sp8	120	44	37
506	(6S)(4S)GalNAcb1-4GlcNAc-Sp8	118	118	100
5	GalNAca-Sp15	118	23	19
263	Neu5Aca2-6GalNAca-Sp8	115	11	9
13	Glcb-Sp8	112	7	6
314	Mana1-2Mana1-6(Mana1-2Mana1-3)Mana1-6(Mana1-2Mana1-2Mana1-3)Mana-Sp9	106	75	70
14	Manb-Sp8	105	9	8
109	Gala1-4(Gala1-3)Galb1-4GlcNAcb-Sp8	105	14	13
215	Mana1-6(Mana1-3)Mana1-6(Mana1-2Mana1-3)Manb1-4GlcNAcb1-4GlcNAcb-Sp12	100	12	12
291	Galb1-4GlcNAcb1-3Galb1-3GlcNAcb-Sp0	95	51	54
483	Galb1-3GlcNAcb1-6GalNAca-Sp14	91	25	28
259	Neu5Aca2-3Galb1-4GlcNAcb1-3Galb1-4GlcNAcb-Sp0	88	27	30
7	Fuca-Sp9	88	11	12
77	Fuca1-2Galb1-4Glcb-Sp0	84	3	3
336	GlcNAca1-4Galb1-4GlcNAcb-Sp0	82	61	74
15	GalNAcb-Sp8	82	14	17
98	GalNAcb1-4GlcNAcb-Sp0	80	21	26
440	(6S)Galb1-3(6S)GlcNAc-Sp0	79	33	41
17	GlcNAcb-Sp8	78	30	38
20	Galb1-4GlcNAcb1-6(Galb1-4GlcNAcb1-3)GalNAc-Sp14	76	12	16
21	GlcNAcb1-6(GlcNAcb1-4)(GlcNAcb1-3)GlcNAc-Sp8	75	14	19
9	Neu5Aca-Sp8	75	9	12
12	Galb-Sp8	72	10	14
577	GlcNAcb1-3Galb1-4GlcNAcb1-3Galb1-4GlcNAcb1-3Galb1-4GlcNAcb1-3Galb1-4GlcNAcb1-6(GlcNAcb1-3Galb1-4GlcNAcb1-3Galb1-4GlcNAcb1-3Galb1-4GlcNAcb1-3Galb1-4GlcNAb1-2)Mana1-6(GlcNAcb1-3Galb1-4GlcNAcb1-3Galb1-4GlcNAcb1-3Galb1-4GlcNAcb1-3Galb1-4GlcNAcb1-2Mana1-3)Manb1-4GlcNAcb1-4(Fuca1-6)GlcNAcb-Sp24	72	11	16
178	GlcNAcb1-6(GlcNAcb1-3)Galb1-4GlcNAcb-Sp8	68	14	21
369	Gala1-3Galb1-4(Fuca1-3)GlcNAcb1-2Mana1-6(Gala1-3Galb1-4(Fuca1-3)GlcNAcb1-2Mana1-3)Manb1-4GlcNAcb1-4GlcNAcb-Sp20	67	10	16
4	GalNAca-Sp8	66	7	10
286	Neu5Aca2-3Galb1-4GlcNAcb1-6(Galb1-3)GalNAca-Sp14	65	11	18
534	GlcNAcb1-3Galb1-4GlcNAcb1-2Mana1-6(GlcNAcb1-3Galb1-4GlcNAcb1-2Mana1-3)Manb1-4GlcNAcb1-4GlcNAcb-Sp12	64	15	24
97	GalNAcb1-4(Fuca1-3)GlcNAcb-Sp0	63	5	8
95	GalNAcb1-3(Fuca1-2)Galb-Sp8	63	9	15
297	Neu5Aca2-3Galb1-4(Fuca1-3)GlcNAcb1-6(Galb1-3)GalNAca-Sp14	63	5	8
339	GlcNAca1-4Galb1-4GlcNAcb1-3Galb1-4(Fuca1-3)GlcNAcb1-3Galb1-4(Fuca1-3)GlcNAcb-Sp0	62	4	6
110	Gala1-3GalNAca-Sp8	62	8	13
121	Gala1-4Galb1-4GlcNAcb-Sp8	62	7	12
272	Neu5Aca2-6Galb-Sp8	62	6	9
24	(3S)Galb1-4(Fuca1-3)(6S)Glc-Sp0	61	9	14
108	Gala1-3(Fuca1-2)Galb-Sp18	61	5	8
32	(3S)Galb1-4(Fuca1-3)GlcNAc-Sp0	61	1	2
46	Neu5Aca2-3(6S)Galb1-4GlcNAcb-Sp8	58	4	8
22	6S(3S)Galb1-4(6S)GlcNAcb-Sp0	58	2	3
151	Galb1-4(Fuca1-3)GlcNAcb-Sp0	58	3	6
54	Galb1-4GlcNAcb1-2Mana1-6(Galb1-4GlcNAcb1-2Mana1-3)Manb1-4GlcNAcb1-4GlcNAcb-Sp12	56	10	19
309	GlcNAcb1-4GlcNAcb-Sp12	56	4	7
30	(3S)Galb1-3GlcNAcb-Sp0	55	4	7
19	Galb1-4GlcNAcb1-6(Galb1-4GlcNAcb1-3)GalNAca-Sp8	55	7	12
69	Fuca1-2Galb1-4(Fuca1-3)GlcNAcb1-3Galb1-4(Fuca1-3)GlcNAcb-Sp0	53	5	9
93	GalNAca1-4(Fuca1-2)Galb1-4GlcNAcb-Sp8	53	9	18
105	Gala1-3(Fuca1-2)Galb1-4GlcNAc-Sp0	52	3	6
201	GlcAa-Sp8	52	5	9
354	KDNa2-6Galb1-4GlcNAc-Sp0	52	13	25
23	6S(3S)Galb1-4GlcNAcb-Sp0	51	2	4
45	(6S)Galb1-4(6S)Glcb-Sp8	51	6	12
407	Galb1-3GalNAcb1-4(Neu5Aca2-8Neu5Aca2-3)Galb1-4Glcb-Sp0	51	4	8
597	Neu5Aca2-3Galb1-4GlcNAcb1-3Galb1-4GlcNAcb1-3Galb1-4GlcNAcb1-2Mana1-6(Neu5Aca2-3Galb1-4GlcNAcb1-3Galb1-4GlcNAcb1-3Galb1-4GlcNAcb1-2Mana1-3)Manb1-4GlcNAcb1-4GlcNAcb-Sp12	51	11	22
573	GlcNAcb1-3Galb1-4GlcNAcb1-3Galb1-4GlcNAcb1-6(GlcNAcb1-3Galb1-4GlcNAcb1-3Galb1-4GlcNAb1-2)Mana1-6(GlcNAcb1-3Galb1-4GlcNAcb1-3Galb1-4GlcNAcb1-2Mana1-3)Manb1-4GlcNAcb1-4(Fuca1-6)GlcNAcb-Sp24	50	19	38
132	Galb1-4GlcNAcb1-6GalNAc-Sp14	49	6	13
128	Galb1-3(Fuca1-4)GlcNAc-Sp0	49	6	13
321	Galb1-3GlcNAcb1-2Mana1-6(Galb1-3GlcNAcb1-2Mana1-3)Manb1-4GlcNAcb1-4GlcNAcb-Sp19	49	9	18
331	GalNAcb1-3Gala1-4Galb1-4GlcNAcb1-3Galb1-4Glcb-Sp0	49	7	15
249	Neu5Aca2-3Galb1-4(6S)GlcNAcb-Sp8	48	12	25
362	Fuca1-4(Galb1-3)GlcNAcb1-2Mana1-6(Fuca1-4(Galb1-3)GlcNAcb1-2Mana1-3)Manb1-4GlcNAcb1-4(Fuca1-6)GlcNAcb-Sp22	48	1	2
71	Fuca1-2Galb1-4(Fuca1-3)GlcNAcb-Sp0	48	6	13
202	GlcAb-Sp8	47	2	5
104	Gala1-3(Fuca1-2)Galb1-4(Fuca1-3)GlcNAcb-Sp8	46	2	5
167	Galb1-4GlcNAcb1-6(Galb1-3)GalNAc-Sp14	46	6	14
70	Fuca1-2Galb1-4(Fuca1-3)GlcNAcb1-3Galb1-4(Fuca1-3)GlcNAcb1-3Galb1-4(Fuca1-3)GlcNAcb-Sp0	45	3	7
232	GalNAcb1-4(Neu5Aca2-3)Galb1-4Glcb-Sp0	44	9	19
464	Gala1-3(Fuca1-2)Galb1-3GalNAcb-Sp8	44	9	21
26	(3S)Galb1-4(6S)Glcb-Sp0	44	2	5
575	GlcNAcb1-3Galb1-4GlcNAcb1-3Galb1-4GlcNAcb1-3Galb1-4GlcNAcb1-6(GlcNAcb1-3Galb1-4GlcNAcb1-3Galb1-4GlcNAcb1-3Galb1-4GlcNAb1-2)Mana1-6(GlcNAcb1-3Galb1-4GlcNAcb1-3Galb1-4GlcNAcb1-3Galb1-4GlcNAcb1-2Mana1-3)Manb1-4GlcNAcb1-4(Fuca1-6)GlcNAcb-Sp24	44	9	21
35	(3S)Galb1-4(6S)GlcNAcb-Sp8	43	6	13
368	Gala1-3(Fuca1-2)Galb1-4GlcNAcb1-2Mana1-6(Gala1-3(Fuca1-2)Galb1-4GlcNAcb1-2Mana1-3)Manb1-4GlcNAcb1-4GlcNAcb-Sp20	43	3	7
114	Gala1-3Galb1-3GlcNAcb-Sp0	43	2	4
226	GalNAcb1-4(Neu5Aca2-8Neu5Aca2-8Neu5Aca2-3)Galb1-4Glcb-Sp0	43	3	7
481	Neu5Aca2-3Galb1-3GlcNAcb1-2Mana1-6(GlcNAcb1-4)(Neu5Aca2-3Galb1-3GlcNAcb1-2Mana1-3)Manb1-4GlcNAcb1-4GlcNAc-Sp21	43	11	25
44	(6S)Galb1-4GlcNAcb-Sp8	42	4	9
61	Fuca1-2Galb1-3GalNAca-Sp8	42	12	28
357	Fuca1-2Galb1-3GlcNAcb1-2Mana1-6(Fuca1-2Galb1-3GlcNAcb1-2Mana1-3)Manb1-4GlcNAcb1-4GlcNAcb-Sp20	42	1	3
137	Neu5Acb2-6(Galb1-3)GalNAca-Sp8	42	9	22
87	GalNAca1-3(Fuca1-2)Galb1-4Glcb-Sp0	42	8	18
67	Fuca1-2Galb1-3GlcNAcb-Sp0	41	3	6
578	Galb1-4GlcNAcb1-3Galb1-4GlcNAcb1-3Galb1-4GlcNAcb1-3Galb1-4GlcNAcb1-3Galb1-4GlcNAcb1-6(Galb1-4GlcNAcb1-3Galb1-4GlcNAcb1-3Galb1-4GlcNAcb1-3Galb1-4GlcNAcb1-3Galb1-4GlcNAb1-2)Mana1-6(Galb1-4GlcNAcb1-3Galb1-4GlcNAcb1-3Galb1-4GlcNAcb1-3Galb1-4GlcNAcb1-3Galb1-4GlcNAcb1-2Mana1-3)Manb1-4GlcNAcb1-4(Fuca1-6)GlcNAcb-Sp24	41	1	2
416	Fuca1-2Galb1-4GlcNAcb1-2Mana1-6(Fuca1-2Galb1-4GlcNAcb1-2Mana1-3)Manb1-4GlcNAcb1-4(Fuca1-6)GlcNAcb-Sp22	41	3	6
469	Fuca1-2Galb1-3(Fuca1-4)GlcNAcb1-2Mana1-6(Fuca1-2Galb1-3(Fuca1-4)GlcNAcb1-2Mana1-3)Manb1-4GlcNAcb1-4(Fuca1-6)GlcNAcb1-4(Fuca1-6)GlcNAcb-Sp19	41	2	4
479	Mana1-6(Mana1-3)Manb1-4GlcNAcb1-4(Fuca1-6)GlcNAcb-Sp19	41	8	19
528	Fuca1-4(Galb1-3)GlcNAcb1-2 Mana-Sp0	41	13	32

121	Gala1-4Galb1-4GlcNAcb-Sp8	62	7	12
122	Gala1-4Galb1-4Glcb-Sp0	28	6	20
123	Gala1-4GlcNAcb-Sp8	214	71	33
124	Gala1-6Glcb-Sp8	31	4	14
125	Galb1-2Galb-Sp8	33	3	9
126	Galb1-3(Fuca1-4)GlcNAcb1-3Galb1-4(Fuca1-3)GlcNAcb-Sp0	25	3	13
127	Galb1-3GlcNAcb1-3Galb1-4(Fuca1-3)GlcNAcb-Sp0	25	2	8
128	Galb1-3(Fuca1-4)GlcNAc-Sp0	49	6	13
129	Galb1-3(Fuca1-4)GlcNAc-Sp8	27	6	21
130	Fuca1-4(Galb1-3)GlcNAcb-Sp8	29	2	7
131	Galb1-4GlcNAcb1-6GalNAca-Sp8	37	1	3
132	Galb1-4GlcNAcb1-6GalNAc-Sp14	49	6	13
133	GlcNAcb1-6(Galb1-3)GalNAca-Sp8	37	6	17
134	GlcNAcb1-6(Galb1-3)GalNAca-Sp14	17	1	5
135	Neu5Aca2-6(Galb1-3)GalNAca-Sp8	37	9	24
136	Neu5Aca2-6(Galb1-3)GalNAca-Sp14	17	3	14
137	Neu5Acb2-6(Galb1-3)GalNAca-Sp8	42	9	22
138	Neu5Aca2-6(Galb1-3)GlcNAcb1-4Galb1-4Glcb-Sp10	12	12	102
139	Galb1-3GalNAca-Sp8	247	147	60
140	Galb1-3GalNAca-Sp14	22	3	12
141	Galb1-3GalNAca-Sp16	36	7	21
142	Galb1-3GalNAcb-Sp8	281	30	11
143	Galb1-3GalNAcb1-3Gala1-4Galb1-4Glcb-Sp0	27	4	16
144	Galb1-3GalNAcb1-4(Neu5Aca2-3)Galb1-4Glcb-Sp0	40	1	3
145	Galb1-3GalNAcb1-4Galb1-4Glcb-Sp8	23	3	11
146	Galb1-3Galb-Sp8	326	124	38
147	Galb1-3GlcNAcb1-3Galb1-4GlcNAcb-Sp0	17	1	6
148	Galb1-3GlcNAcb1-3Galb1-4Glcb-Sp10	28	7	25
149	Galb1-3GlcNAcb-Sp0	21	2	10
150	Galb1-3GlcNAcb-Sp8	32	5	15
151	Galb1-4(Fuca1-3)GlcNAcb-Sp0	58	3	6
152	Galb1-4(Fuca1-3)GlcNAcb-Sp8	20	4	21
153	Galb1-4(Fuca1-3)GlcNAcb1-3Galb1-4(Fuca1-3)GlcNAcb-Sp0	28	2	7
154	Galb1-4(Fuca1-3)GlcNAcb1-3Galb1-4(Fuca1-3)GlcNAcb1-3Galb1-4(Fuca1-3)GlcNAcb-Sp0	20	1	3
155	Galb1-4(6S)Glcb-Sp0	36	4	11
156	Galb1-4(6S)Glcb-Sp8	26	1	3
157	Galb1-4GalNAca1-3(Fuca1-2)Galb1-4GlcNAcb-Sp8	22	1	5
158	Galb1-4GalNAcb1-3(Fuca1-2)Galb1-4GlcNAcb-Sp8	27	3	11
159	Galb1-4GlcNAcb1-3GalNAca-Sp8	20	4	19
160	Galb1-4GlcNAcb1-3GalNAc-Sp14	25	1	5
161	Galb1-4GlcNAcb1-3Galb1-4(Fuca1-3)GlcNAcb1-3Galb1-4(Fuca1-3)GlcNAcb-Sp0	20	3	17
162	Galb1-4GlcNAcb1-3Galb1-4GlcNAcb1-3Galb1-4GlcNAcb-Sp0	2	17	869
163	Galb1-4GlcNAcb1-3Galb1-4GlcNAcb-Sp0	16	1	7
164	Galb1-4GlcNAcb1-3Galb1-4Glcb-Sp0	16	3	18
165	Galb1-4GlcNAcb1-3Galb1-4Glcb-Sp8	172	88	51
166	Galb1-4GlcNAcb1-6(Galb1-3)GalNAca-Sp8	21	2	7
167	Galb1-4GlcNAcb1-6(Galb1-3)GalNAc-Sp14	46	6	14
168	Galb1-4GlcNAcb-Sp0	22	4	17
169	Galb1-4GlcNAcb-Sp8	192	61	32
170	Galb1-4GlcNAcb-Sp23	20	4	19
171	Galb1-4Glcb-Sp0	26	1	4
172	Galb1-4Glcb-Sp8	20	6	31
173	GlcNAca1-3Galb1-4GlcNAcb-Sp8	21	1	4
174	GlcNAca1-6Galb1-4GlcNAcb-Sp8	39	5	12
175	GlcNAcb1-2Galb1-3GalNAca-Sp8	17	4	22
176	GlcNAcb1-6(GlcNAcb1-3)GalNAca-Sp8	17	2	12
177	GlcNAcb1-6(GlcNAcb1-3)GalNAca-Sp14	22	3	13
178	GlcNAcb1-6(GlcNAcb1-3)Galb1-4GlcNAcb-Sp8	68	14	21
179	GlcNAcb1-3GalNAca-Sp8	27	2	8
180	GlcNAcb1-3GalNAca-Sp14	18	5	29
181	GlcNAcb1-3Galb-Sp8	20	3	15
182	GlcNAcb1-3Galb1-4GlcNAcb-Sp0	22	1	6
183	GlcNAcb1-3Galb1-4GlcNAcb-Sp8	35	4	13
184	GlcNAcb1-3Galb1-4GlcNAcb1-3Galb1-4GlcNAcb-Sp0	12	3	24
185	GlcNAcb1-3Galb1-4Glcb-Sp0	10	20	210
186	GlcNAcb1-4-MDPLys	20	1	5
187	GlcNAcb1-6(GlcNAcb1-4)GalNAca-Sp8	32	7	21
188	GlcNAcb1-4Galb1-4GlcNAcb-Sp8	30	7	24
189	GlcNAcb1-4GlcNAcb1-4GlcNAcb1-4GlcNAcb1-4GlcNAcb1-4GlcNAcb1-Sp8	31	5	17
190	GlcNAcb1-4GlcNAcb1-4GlcNAcb1-4GlcNAcb1-4GlcNAcb1-Sp8	21	2	8
191	GlcNAcb1-4GlcNAcb1-4GlcNAcb-Sp8	23	4	19
192	GlcNAcb1-6GalNAca-Sp8	210	36	17
193	GlcNAcb1-6GalNAca-Sp14	17	3	17
194	GlcNAcb1-6Galb1-4GlcNAcb-Sp8	28	3	11
195	Glca1-4Glcb-Sp8	25	4	16
196	Glca1-4Glca-Sp8	289	147	51
197	Glca1-6Glca1-6Glcb-Sp8	35	2	5
198	Glcb1-4Glcb-Sp8	36	5	15
199	Glcb1-6Glcb-Sp8	30	3	12
200	G-ol-Sp8	28	3	11
201	GlcAa-Sp8	52	5	9
202	GlcAb-Sp8	47	2	5
203	GlcAb1-3Galb-Sp8	35	3	9
204	GlcAb1-6Galb-Sp8	35	4	11
205	KDNa2-3Galb1-3GlcNAcb-Sp0	36	25	71
206	KDNa2-3Galb1-4GlcNAcb-Sp0	39	3	7
207	Mana1-2Mana1-2Mana1-3Mana-Sp9	17	2	10
208	Mana1-2Mana1-6(Mana1-2Mana1-3)Mana-Sp9	22	2	8
209	Mana1-2Mana1-3Mana-Sp9	24	3	12
210	Mana1-6(Mana1-2Mana1-3)Mana1-6(Mana1-2Mana1-3)Manb1-4GlcNAcb1-4GlcNAcb-Sp12	28	4	16
211	Mana1-2Mana1-6(Mana1-3)Mana1-6(Mana1-2Mana1-2Mana1-3)Manb1-4GlcNAcb1-4GlcNAcb-Sp12	29	13	45
212	Mana1-2Mana1-6(Mana1-2Mana1-3)Mana1-6(Mana1-2Mana1-2Mana1-3)Manb1-4GlcNAcb1-4GlcNAcb-Sp12	23	5	23
213	Mana1-6(Mana1-3)Mana-Sp9	23	3	14
214	Mana1-2Mana1-2Mana1-6(Mana1-3)Mana-Sp9	19	2	13
215	Mana1-6(Mana1-3)Mana1-6(Mana1-2Mana1-3)Manb1-4GlcNAcb1-4GlcNAcb-Sp12	100	12	12
216	Mana1-6(Mana1-3)Mana1-6(Mana1-3)Manb1-4GlcNAcb1-4GlcNAcb-Sp12	20	1	4
217	Manb1-4GlcNAcb-Sp0	22	1	6
218	Neu5Aca2-3Galb1-4GlcNAcb1-3Galb1-4(Fuca1-3)GlcNAcb-Sp0	11	5	43
219	(3S)Galb1-4(Fuca1-3)(6S)GlcNAcb-Sp8	126	16	13
220	Fuca1-2(6S)Galb1-4GlcNAcb-Sp0	24	1	3
221	Fuca1-2Galb1-4(6S)GlcNAcb-Sp8	33	6	17
222	Fuca1-2(6S)Galb1-4(6S)Glcb-Sp0	30	1	5
223	Neu5Aca2-3Galb1-3GalNAca-Sp8	20	2	9
224	Neu5Aca2-3Galb1-3GalNAca-Sp14	39	10	26
225	GalNAcb1-4(Neu5Aca2-8Neu5Aca2-8Neu5Aca2-8Neu5Aca2-3)Galb1-4Glcb-Sp0	26	2	6
226	GalNAcb1-4(Neu5Aca2-8Neu5Aca2-8Neu5Aca2-3)Galb1-4Glcb-Sp0	43	3	7
227	Neu5Aca2-8Neu5Aca2-8Neu5Aca2-3Galb1-4Glcb-Sp0	24	4	16
228	GalNAcb1-4(Neu5Aca2-8Neu5Aca2-3)Galb1-4Glcb-Sp0	30	6	19
229	Neu5Aca2-8Neu5Aca2-8Neu5Aca-Sp8	18	2	11
230	GalNAcb1-4(Neu5Aca2-3)Galb1-4GlcNAcb-Sp0	37	3	9
231	GalNAcb1-4(Neu5Aca2-3)Galb1-4GlcNAcb-Sp8	20	1	3
232	GalNAcb1-4(Neu5Aca2-3)Galb1-4Glcb-Sp0	44	9	19
233	Neu5Aca2-3Galb1-3GalNAcb1-4(Neu5Aca2-3)Galb1-4Glcb-Sp0	27	12	46
234	Neu5Aca2-6(Neu5Aca2-3)GalNAca-Sp8	15	2	11
235	Neu5Aca2-3GalNAca-Sp8	23	3	14
236	Neu5Aca2-3GalNAcb1-4GlcNAcb-Sp0	17	4	25
237	Neu5Aca2-3Galb1-3(6S)GlcNAc-Sp8	120	51	43
238	Neu5Aca2-3Galb1-3(Fuca1-4)GlcNAcb-Sp8	3923	69	2

29	(3S)Galb1-3GalNAca-Sp8	41	2	4
282	Neu5Gca2-3Galb1-4Glcb-Sp0	41	10	26
515	Neu5Aca2-6Galb1-4GlcNAcb1-2Man-Sp0	41	13	31
441	Fuca1-2Galb1-4 GlcNAcb1-2Mana1-6(Fuca1-2Galb1-4GlcNAcb1-2(Fuca1-2Galb1-4GlcNAcb1-4)Mana1-3)Manb1-4GlcNAcb1-4GlcNAcb-Sp12	40	1	3
536	Fuca1-2Galb1-4GlcNAcb1-3Galb1-4GlcNAcb1-2Mana1-6(Fuca1-2Galb1-4GlcNAcb1-3Galb1-4GlcNAcb1-2Mana1-3)Manb1-4GlcNAcb1-4GlcNAcb-Sp24	40	2	6
31	(3S)Galb1-3GlcNAcb-Sp8	40	4	9
391	Gala1-3Galb1-3(Fuca1-4)GlcNAcb1-2Mana1-6(Gala1-3Galb1-3(Fuca1-4)GlcNAcb1-2Mana1-3)Manb1-4GlcNAcb1-4GlcNAc-Sp19	40	6	16
94	GalNAcb1-3GalNAca-Sp8	40	1	1
144	Galb1-3GalNAcb1-4(Neu5Aca2-3)Galb1-4Glcb-Sp0	40	1	3
568	GlcNAcb1-3Galb1-4GlcNAcb1-3Galb1-4GlcNAcb1-3Galb1-4GlcNAcb1-2Mana1-6(GlcNAcb1-3Galb1-4GlcNAcb1-3Galb1-4GlcNAcb1-3Galb1-4GlcNAcb1-2Mana1-3)Manb1-4GlcNAcb1-4(Fuca1-6)GlcNAcb-Sp24	40	7	17
572	Galb1-4GlcNAcb1-3Galb1-4GlcNAcb1-6(Galb1-4GlcNAcb1-3Galb1-4GlcNAb1-2)Mana1-6(Galb1-4GlcNAcb1-3Galb1-4GlcNAcb1-2Mana1-3)Manb1-4GlcNAcb1-4(Fuca1-6)GlcNAcb-Sp24	40	4	11
449	GalNAcb1-4Galb1-4Glcb-Sp0	40	2	4
224	Neu5Aca2-3Galb1-3GalNAca-Sp14	39	10	26
471	Galb1-3GlcNAcb1-2Mana1-6(GlcNAcb1-4)(Galb1-3GlcNAcb1-2Mana1-3)Manb1-4GlcNAcb1-4GlcNAcb-Sp21	39	5	11
442	Fuca1-2Galb1-4(Fuca1-3)GlcNAcb1-2Mana1-6(Fuca1-2Galb1-4(Fuca1-3)GlcNAcb1-4(Fuca1-2Galb1-4(Fuca1-3)GlcNAcb1-2)Mana1-3)Manb1-4GlcNAcb1-4GlcNAcb-Sp12	39	4	11
11	Neu5Acb-Sp8	39	1	2
174	GlcNAca1-6Galb1-4GlcNAcb-Sp8	39	5	12
83	GalNAca1-3(Fuca1-2)Galb1-4(Fuca1-3)GlcNAcb-Sp0	39	5	14
206	KDNa2-3Galb1-4GlcNAcb-Sp0	39	3	7
243	Neu5Aca2-6(Neu5Aca2-3Galb1-3)GalNAca-Sp14	39	2	4
374	Neu5Aca2-3Galb1-4(Fuca1-3)GlcNAcb1-3GalNAca-Sp14	39	6	16
375	GalNAcb1-4GlcNAcb1-2Mana1-6(GalNAcb1-4GlcNAcb1-2Mana1-3)Manb1-4GlcNAcb1-4GlcNAc-Sp12	39	8	22
548	GlcNAcb1-3Galb1-4GlcNAcb1-6(GlcNAcb1-3Galb1-4GlcNAcb1-2)Mana1-6(GlcNAcb1-3Galb1-4GlcNAcb1-2Man a1-3)Manb1-4GlcNAcb1-4GlcNAc-Sp24	38	3	7
55	Neu5Aca2-6Galb1-4GlcNAcb1-2Mana1-6(Neu5Aca2-6Galb1-4GlcNAcb1-2Mana1-3)Manb1-4GlcNAcb1-4GlcNAcb-Sp12	38	1	3
248	Neu5Aca2-3Galb1-3GlcNAcb-Sp0	38	2	6
275	Galb1-3(Fuca1-4)GlcNAcb1-3Galb1-3(Fuca1-4)GlcNAcb-Sp0	38	7	20
27	(3S)Galb1-4(6S)Glcb-Sp8	37	6	15
133	GlcNAcb1-6(Galb1-3)GalNAca-Sp8	37	6	17
120	Gala1-4Galb1-4GlcNAcb-Sp0	37	9	25
135	Neu5Aca2-6(Galb1-3)GalNAca-Sp8	37	9	24
480	Galb1-4GlcNAcb1-6(Galb1-4GlcNAcb1-2)Mana1-6(Galb1-4GlcNAcb1-2Mana1-3)Manb1-4GlcNAcb1-4(Fuca1-6)GlcNAcb-Sp24	37	5	13
43	(6S)Galb1-4Glcb-Sp8	37	6	15
131	Galb1-4GlcNAcb1-6GalNAca-Sp8	37	1	3
261	Neu5Aca2-3Galb1-4Glcb-Sp0	37	3	8
311	Mana1-6Manb-Sp10	37	3	7
444	Galb1-4GlcNAcb1-2Mana-Sp0	37	7	19
40	(4S)Galb1-4GlcNAcb-Sp8	37	4	10
230	GalNAcb1-4(Neu5Aca2-3)Galb1-4GlcNAcb-Sp0	37	3	9
255	Neu5Aca2-3Galb1-4(Fuca1-3)GlcNAcb1-3Galb1-4GlcNAcb-Sp8	37	3	7
63	Fuca1-2Galb1-3GalNAcb1-4(Neu5Aca2-3)Galb1-4Glcb-Sp0	36	5	15
118	Gala1-3Galb-Sp8	36	23	63
155	Galb1-4(6S)Glcb-Sp0	36	4	11
361	Galb1-4GlcNAcb1-2Mana1-6(Mana1-3)Manb1-4GlcNAcb1-4GlcNAcb-Sp12	36	4	11
422	Fuca1-2Galb1-3GlcNAcb1-2Mana1-6(Fuca1-2Galb1-3GlcNAcb1-2Mana1-3)Manb1-4GlcNAcb1-4(Fuca1-6)GlcNAcb-Sp22	36	7	18
141	Galb1-3GalNAca-Sp16	36	7	21
58	Fuca1-2Galb1-3GalNAcb1-3Gala-Sp9	36	4	10
99	GalNAcb1-4GlcNAcb-Sp8	36	3	9
408	Neu5Aca2-3Galb1-3GalNAcb1-4(Neu5Aca2-8Neu5Aca2-3)Galb1-4Glcb-Sp0	36	9	24
47	(6S)GlcNAcb-Sp8	36	1	4
198	Glcb1-4Glcb-Sp8	36	5	15
205	KDNa2-3Galb1-3GlcNAcb-Sp0	36	25	71
365	Galb1-4(Fuca1-3)GlcNAcb1-6(Fuca1-2Galb1-4GlcNAcb1-3)Galb1-4Glc-Sp21	36	8	24
546	Neu5Gca2-8Neu5Gca2-6Galb1-4GlcNAc-Sp0	36	16	44
560	(3S)GlcAb1-3Galb1-4GlcNAcb1-2Mana-Sp0	36	2	6
576	Galb1-4GlcNAcb1-3Galb1-4GlcNAcb1-3Galb1-4GlcNAcb1-3Galb1-4GlcNAcb1-6(Galb1-4GlcNAcb1-3Galb1-4GlcNAcb1-3Galb1-4GlcNAcb1-3Galb1-4GlcNAb1-2)Mana1-6(Galb1-4GlcNAcb1-3Galb1-4GlcNAcb1-3Galb1-4GlcNAcb1-3Galb1-4GlcNAcb1-2Mana1-3)Manb1-4GlcNAcb1-4(Fuca1-6)GlcNAcb-Sp24	36	1	4
203	GlcAb1-3Galb-Sp8	35	3	9
417	GlcNAcb1-2(GlcNAcb1-6)Mana1-6(GlcNAcb1-2Mana1-3)Manb1-4GlcNAcb1-4GlcNAcb-Sp19	35	2	6
112	Gala1-3GalNAcb-Sp8	35	3	7
197	Glca1-6Glca1-6Glcb-Sp8	35	2	5
204	GlcAb1-6Galb-Sp8	35	4	11
257	Neu5Aca2-3Galb1-4GlcNAcb-Sp0	35	6	18
340	GlcNAca1-4Galb1-4GlcNAcb1-3Galb1-4GlcNAcb-Sp0	35	6	18
371	Fuca1-4(Fuca1-2Galb1-3)GlcNAcb1-2Mana1-3(Fuca1-4(Fuca1-2Galb1-3)GlcNAcb1-2Mana1-3)Manb1-4GlcNAcb1-4GlcNAcb-Sp19	35	7	21
107	Gala1-3(Fuca1-2)Galb-Sp8	35	4	13
183	GlcNAcb1-3Galb1-4GlcNAcb-Sp8	35	4	13
252	Neu5Aca2-3Galb1-4(Fuca1-3)GlcNAcb-Sp0	35	7	20
592	Neu5Aca2-6Galb1-4GlcNAcb1-6(Galb1-3)GalNAca-Sp14	35	3	10
324	Galb1-4(Fuca1-3)GlcNAcb1-2Mana1-6(Galb1-4(Fuca1-3)GlcNAcb1-2Mana1-3)Manb1-4GlcNAcb1-4GlcNAcb-Sp20	34	3	9
574	Galb1-4GlcNAcb1-3Galb1-4GlcNAcb1-3Galb1-4GlcNAcb1-6(Galb1-4GlcNAcb1-3Galb1-4GlcNAcb1-3Galb1-4GlcNAb1-2)Mana1-6(Galb1-4GlcNAcb1-3Galb1-4GlcNAcb1-3Galb1-4GlcNAcb1-2Mana1-3)Manb1-4GlcNAcb1-4(Fuca1-6)GlcNAcb-Sp24	34	1	4
421	Gala1-3Galb1-3GlcNAcb1-3GalNAc-Sp14	34	5	16
457	Neu5Aca2-3Galb1-4GlcNAcb1-6(Neu5Aca2-3Galb1-4GlcNAcb1-2)Mana1-6(GlcNAcb1-4)(Neu5Aca2-3Galb1-4GlcNAcb1-2Mana1-3)Manb1-4GlcNAcb1-4GlcNAcb-Sp21	34	21	61
495	Fuca1-2Galb1-3(6S)GlcNAcb-Sp0	34	5	14
270	Neu5Aca2-6Galb1-4Glcb-Sp0	34	5	15
448	Neu5Aca2-8Neu5Aca2-3Galb1-3GalNAcb1-4(Neu5Aca2-8Neu5Aca2-3)Galb1-4Glcb-Sp0	34	2	5
477	Neu5Aca2-6Galb1-4GlcNAcb1-2Mana1-6(Neu5Aca2-6Galb1-4GlcNAcb1-2Mana1-3)Manb1-4GlcNAcb1-4(Fuca1-6)GlcNAcb-Sp24	34	2	5
102	Gala1-3(Fuca1-2)Galb1-3GlcNAcb-Sp8	33	2	6
125	Galb1-2Galb-Sp8	33	3	9
279	Neu5Gca2-3Galb1-3GlcNAcb-Sp0	33	2	5
350	Galb1-4GlcNAcb1-2Mana1-6(Galb1-4GlcNAcb1-2Mana1-3)Manb1-4GlcNAcb1-4(Fuca1-6)GlcNAcb-Sp22	33	3	8
380	Galb1-4GlcNAcb1-6(Fuca1-4(Fuca1-2Galb1-3)GlcNAcb1-3)Galb1-4Glc-Sp21	33	2	7
221	Fuca1-2Galb1-4(6S)GlcNAcb-Sp8	33	6	17
338	GlcNAca1-4Galb1-4GlcNAcb1-3Galb1-4Glcb-Sp0	33	5	14
418	Fuca1-2Galb1-3GlcNAcb1-3GalNAc-Sp14	33	12	36
539	Galb1-4GlcNAcb1-3Galb1-4GlcNAcb1-3Galb1-4GlcNAcb1-2Mana1-6(Galb1-4GlcNAcb1-3Galb1-4GlcNAcb1-3Galb1-4GlcNAcb1-2Mana1-3)Manb1-4GlcNAcb1-4GlcNAcb-Sp12	33	2	7
581	Galb1-4GlcNAcb1-3Galb1-4GlcNAcb1-6(Galb1-4GlcNAcb1-3Galb1-4GlcNAcb1-3)GalNAca-Sp14	33	3	11
82	GalNAca1-3(Fuca1-2)Galb1-3GlcNAcb-Sp0	32	3	10
187	GlcNAcb1-6(GlcNAcb1-4)GalNAca-Sp8	32	7	21
277	Neu5Acb2-6Galb1-4GlcNAcb-Sp8	32	4	13
320	Neu5Gcb2-6Galb1-4GlcNAc-Sp8	32	1	4
564	GlcNAcb1-3Galb1-4GlcNAcb1-2Mana1-6(GlcNAcb1-3Galb1-4GlcNAcb1-2Mana1-3)Manb1-4GlcNAcb1-4(Fuca1-6)GlcNAcb-Sp24	32	6	19
34	(3S)Galb1-4(6S)GlcNAcb-Sp0	32	4	14
150	Galb1-3GlcNAcb-Sp8	32	5	15
280	Neu5Gca2-3Galb1-4(Fuca1-3)GlcNAcb-Sp0	32	7	23
305	GlcAb1-3GlcNAcb-Sp8	32	3	9
424	Galb1-3GlcNAcb1-6(Galb1-3GlcNAcb1-2)Mana1-6(Galb1-3GlcNAcb1-2Mana1-3)Manb1-4GlcNAcb1-4GlcNAcb-Sp19	32	5	15
472	Neu5Aca2-6Galb1-4GlcNAcb1-6(Galb1-3GlcNAcb1-3)Galb1-4Glcb-Sp21	32	5	14
509	GalNAcb1-4(6S)GlcNAc-Sp8	32	20	61
470	GlcNAcb1-6(GlcNAcb1-2)Mana1-6(GlcNAcb1-2Mana1-3)Manb1-4GlcNAcb1-4(Fuca1-6)GlcNAcb-Sp24	32	4	12
514	GalNAca1-3(Fuca1-2)Galb1-4GlcNAcb1-6GalNAc-Sp14	32	1	4
519	Galb1-3GlcNAcb1-2Mana-Sp0	32	4	11
571	Galb1-4GlcNAcb1-3Galb1-4GlcNAcb1-3Galb1-4GlcNAcb1-3Galb1-4GlcNAcb1-3Galb1-4GlcNAcb1-2Mana1-6(Galb1-4GlcNAcb1-3Galb1-4GlcNAcb1-3Galb1-4GlcNAcb1-3Galb1-4GlcNAcb1-3Galb1-4GlcNAcb1-2Mana1-3)Manb1-4GlcNAcb1-4(Fuca1-6)GlcNAcb-Sp19	32	2	5
367	GalNAca1-3(Fuca1-2)Galb1-4GlcNAcb1-2Mana1-6(GalNAca1-3(Fuca1-2)Galb1-4GlcNAcb1-2Mana1-3)Manb1-4GlcNAcb1-4GlcNAcb-Sp20	32	3	8
500	Fuca1-2Galb1-3GlcNAcb1-6(Fuca1-2Galb1-3GlcNAcb1-3)GalNAca-Sp14	32	3	8
33	(3S)Galb1-4(Fuca1-3)GlcNAc-Sp8	31	4	13
385	Fuca1-2Galb1-3GalNAca1-3(Fuca1-2)Galb1-4Glcb-Sp0	31	20	63
28	(3S)Galb1-3(Fuca1-4)GlcNAcb-Sp8	31	2	7
66	Fuca1-2Galb1-3GlcNAcb1-3Galb1-4Glcb-Sp10	31	1	4
117	Gala1-3Galb1-4Glc-Sp10	31	2	6
268	Neu5Aca2-6Galb1-4GlcNAcb1-3Galb1-4(Fuca1-3)GlcNAcb1-3Galb1-4(Fuca1-3)GlcNAcb-Sp0	31	3	9
397	Galb1-4(Fuca1-3)GlcNAcb1-3GalNAca-Sp14	31	1	4
415	Galb1-4(Fuca1-3)GlcNAcb1-2Mana1-6(Galb1-4(Fuca1-3)GlcNAcb1-2Mana1-3)Manb1-4GlcNAcb1-4(Fuca1-6)GlcNAcb-Sp22	31	5	17
124	Gala1-6Glcb-Sp8	31	4	14
189	GlcNAcb1-4GlcNAcb1-4GlcNAcb1-4GlcNAcb1-4GlcNAcb1-4GlcNAcb1-Sp8	31	5	17
302	Galb1-4GlcNAca1-6Galb1-4GlcNAcb-Sp0	31	4	12
373	Neu5Aca2-6Galb1-4GlcNAcb1-3GalNAc-Sp14	31	2	7
396	Fuca1-2Galb1-4GlcNAcb1-3GalNAca-Sp14	31	5	16
288	Galb1-4(Fuca1-3)(6S)GlcNAcb-Sp0	30	1	2
334	Neu5Aca2-3Galb1-4(Fuca1-3)GlcNAcb1-6(Neu5Aca2-3Galb1-3)GalNAc-Sp14	30	2	5

239	Neu5Aca2-3Galb1-3(Fuca1-4)GlcNAcb1-3Galb1-4(Fuca1-3)GlcNAcb-Sp0	3471	198	6
240	Neu5Aca2-3Galb1-4(Neu5Aca2-3Galb1-3)GlcNAcb-Sp8	19	4	22
241	Neu5Aca2-3Galb1-3(6S)GalNAca-Sp8	143	23	16
242	Neu5Aca2-6(Neu5Aca2-3Galb1-3)GalNAca-Sp8	19	2	8
243	Neu5Aca2-6(Neu5Aca2-3Galb1-3)GalNAca-Sp14	39	2	4
244	Neu5Aca2-3Galb-Sp8	26	4	15
245	Neu5Aca2-3Galb1-3GalNAcb1-3Gala1-4Galb1-4Glcb-Sp0	26	2	7
246	Neu5Aca2-3Galb1-3GlcNAcb1-3Galb1-4GlcNAcb-Sp0	23	3	13
247	Fuca1-2(6S)Galb1-4Glcb-Sp0	24	4	16
248	Neu5Aca2-3Galb1-3GlcNAcb-Sp0	38	2	6
249	Neu5Aca2-3Galb1-4(6S)GlcNAcb-Sp8	48	12	25
250	Neu5Aca2-3Galb1-4(Fuca1-3)(6S)GlcNAcb-Sp8	20	2	8
251	Neu5Aca2-3Galb1-4(Fuca1-3)GlcNAcb1-3Galb1-4(Fuca1-3)GlcNAcb1-3Galb1-4(Fuca1-3)GlcNAcb-Sp0	29	4	14
252	Neu5Aca2-3Galb1-4(Fuca1-3)GlcNAcb-Sp0	35	7	20
253	Neu5Aca2-3Galb1-4(Fuca1-3)GlcNAcb-Sp8	17	3	15
254	Neu5Aca2-3Galb1-4(Fuca1-3)GlcNAcb1-3Galb-Sp8	25	2	9
255	Neu5Aca2-3Galb1-4(Fuca1-3)GlcNAcb1-3Galb1-4GlcNAcb-Sp8	37	3	7
256	Neu5Aca2-3Galb1-4GlcNAcb1-3Galb1-4GlcNAcb1-3Galb1-4GlcNAcb-Sp0	13	2	13
257	Neu5Aca2-3Galb1-4GlcNAcb-Sp0	35	6	18
258	Neu5Aca2-3Galb1-4GlcNAcb-Sp8	14	4	29
259	Neu5Aca2-3Galb1-4GlcNAcb1-3Galb1-4GlcNAcb-Sp0	88	27	30
260	Fuca1-2Galb1-4(6S)Glcb-Sp0	25	1	6
261	Neu5Aca2-3Galb1-4Glcb-Sp0	37	3	8
262	Neu5Aca2-3Galb1-4Glcb-Sp8	18	2	11
263	Neu5Aca2-6GalNAca-Sp8	115	11	9
264	Neu5Aca2-6GalNAcb1-4GlcNAcb-Sp0	18	1	3
265	Neu5Aca2-6Galb1-4(6S)GlcNAcb-Sp8	29	5	17
266	Neu5Aca2-6Galb1-4GlcNAcb-Sp0	21	2	10
267	Neu5Aca2-6Galb1-4GlcNAcb-Sp8	19	1	7
268	Neu5Aca2-6Galb1-4GlcNAcb1-3Galb1-4(Fuca1-3)GlcNAcb1-3Galb1-4(Fuca1-3)GlcNAcb-Sp0	31	3	9
269	Neu5Aca2-6Galb1-4GlcNAcb1-3Galb1-4GlcNAcb-Sp0	19	1	6
270	Neu5Aca2-6Galb1-4Glcb-Sp0	34	5	15
271	Neu5Aca2-6Galb1-4Glcb-Sp8	17	5	29
272	Neu5Aca2-6Galb-Sp8	62	6	9
273	Neu5Aca2-8Neu5Aca-Sp8	20	2	10
274	Neu5Aca2-8Neu5Aca2-3Galb1-4Glcb-Sp0	15	1	8
275	Galb1-3(Fuca1-4)GlcNAcb1-3Galb1-3(Fuca1-4)GlcNAcb-Sp0	38	7	20
276	Neu5Acb2-6GalNAca-Sp8	19	6	30
277	Neu5Acb2-6Galb1-4GlcNAcb-Sp8	32	4	13
278	Neu5Gca2-3Galb1-3(Fuca1-4)GlcNAcb-Sp0	634	30	5
279	Neu5Gca2-3Galb1-3GlcNAcb-Sp0	33	2	5
280	Neu5Gca2-3Galb1-4(Fuca1-3)GlcNAcb-Sp0	32	7	23
281	Neu5Gca2-3Galb1-4GlcNAcb-Sp0	23	4	16
282	Neu5Gca2-3Galb1-4Glcb-Sp0	41	10	26
283	Neu5Gca2-6GalNAca-Sp0	27	3	10
284	Neu5Gca2-6Galb1-4GlcNAcb-Sp0	24	5	19
285	Neu5Gca-Sp8	14	5	38
286	Neu5Aca2-3Galb1-4GlcNAcb1-6(Galb1-3)GalNAca-Sp14	65	11	18
287	Galb1-3GlcNAcb1-3Galb1-3GlcNAcb-Sp0	15	3	18
288	Galb1-4(Fuca1-3)(6S)GlcNAcb-Sp0	30	1	2
289	Galb1-4(Fuca1-3)(6S)Glcb-Sp0	26	1	3
290	Galb1-4(Fuca1-3)GlcNAcb1-3Galb1-3(Fuca1-4)GlcNAcb-Sp0	14	16	116
291	Galb1-4GlcNAcb1-3Galb1-3GlcNAcb-Sp0	95	51	54
292	Neu5Aca2-3Galb1-3GlcNAcb1-3Galb1-3GlcNAcb-Sp0	16	1	6
293	Neu5Aca2-3Galb1-4GlcNAcb1-3Galb1-3GlcNAcb-Sp0	26	2	8
294	4S(3S)Galb1-4GlcNAcb-Sp0	25	1	5
295	(6S)Galb1-4(6S)GlcNAcb-Sp0	25	9	34
296	(6P)Glcb-Sp10	29	3	10
297	Neu5Aca2-3Galb1-4(Fuca1-3)GlcNAcb1-6(Galb1-3)GalNAca-Sp14	63	5	8
298	Galb1-3Galb1-4GlcNAcb-Sp8	27	3	11
299	Neu5Aca2-6Galb1-4GlcNAcb1-2Mana1-6(Galb1-4GlcNAcb1-2Mana1-3)Manb1-4GlcNAcb1-4GlcNAcb-Sp12	27	3	13
300	Galb1-4GlcNAcb1-6(Galb1-4GlcNAcb1-3)Galb1-4GlcNAc-Sp0	22	4	19
301	GlcNAcb1-6(Galb1-4GlcNAcb1-3)Galb1-4GlcNAc-Sp0	20	2	10
302	Galb1-4GlcNAca1-6Galb1-4GlcNAcb-Sp0	31	4	12
303	Galb1-4GlcNAcb1-6Galb1-4GlcNAcb-Sp0	19	5	23
304	GalNAcb1-3Galb-Sp8	13	1	11
305	GlcAb1-3GlcNAcb-Sp8	32	3	9
306	Neu5Aca2-6Galb1-4GlcNAcb1-2Mana1-6(GlcNAcb1-2Mana1-3)Manb1-4GlcNAcb1-4GlcNAcb-Sp12	15	1	9
307	GlcNAcb1-3Man-Sp10	25	3	11
308	GlcNAcb1-4GlcNAcb-Sp10	10	1	14
309	GlcNAcb1-4GlcNAcb-Sp12	56	4	7
310	MurNAcb1-4GlcNAcb-Sp10	17	1	8
311	Mana1-6Manb-Sp10	37	3	7
312	Mana1-6(Mana1-3)Mana1-6(Mana1-3)Manb-Sp10	17	2	10
313	Mana1-2Mana1-6(Mana1-3)Mana1-6(Mana1-2Mana1-2Mana1-3)Mana-Sp9	18	14	78
314	Mana1-2Mana1-6(Mana1-2Mana1-3)Mana1-6(Mana1-2Mana1-2Mana1-3)Mana-Sp9	106	75	70
315	Neu5Aca2-3Galb1-4GlcNAcb1-6(Neu5Aca2-3Galb1-3)GalNAca-Sp14	15	2	10
316	Neu5Aca2-6Galb1-4GlcNAcb1-2Mana1-6(Neu5Aca2-3Galb1-4GlcNAcb1-2Mana1-3)Manb1-4GlcNAcb1-4GlcNAcb-Sp12	22	2	8
317	Galb1-4GlcNAcb1-2Mana1-6(Neu5Aca2-6Galb1-4GlcNAcb1-2Mana1-3)Manb1-4GlcNAcb1-4GlcNAcb-Sp12	16	2	11
318	Neu5Aca2-8Neu5Acb-Sp17	23	2	11
319	Neu5Aca2-8Neu5Aca2-8Neu5Acb-Sp8	17	5	29
320	Neu5Gcb2-6Galb1-4GlcNAc-Sp8	32	1	4
321	Galb1-3GlcNAcb1-2Mana1-6(Galb1-3GlcNAcb1-2Mana1-3)Manb1-4GlcNAcb1-4GlcNAcb-Sp19	49	9	18
322	Neu5Aca2-3Galb1-4GlcNAcb1-2Mana1-6(Neu5Aca2-3Galb1-4GlcNAcb1-2Mana1-3)Manb1-4GlcNAcb1-4GlcNAcb-Sp12	18	1	8
323	Neu5Aca2-3Galb1-4GlcNAcb1-2Mana1-6(Neu5Aca2-6Galb1-4GlcNAcb1-2Mana1-3)Manb1-4GlcNAcb1-4GlcNAcb-Sp12	13	1	6
324	Galb1-4(Fuca1-3)GlcNAcb1-2Mana1-6(Galb1-4(Fuca1-3)GlcNAcb1-2Mana1-3)Manb1-4GlcNAcb1-4GlcNAcb-Sp20	34	3	9
325	Neu5,9Ac2a2-3Galb1-4GlcNAcb-Sp0	18	6	36
326	Neu5,9Ac2a2-3Galb1-3GlcNAcb-Sp0	17	4	22
327	Neu5Aca2-6Galb1-4GlcNAcb1-3Galb1-3GlcNAcb-Sp0	16	1	6
328	Neu5Aca2-3Galb1-3(Fuca1-4)GlcNAcb1-3Galb1-3(Fuca1-4)GlcNAcb-Sp0	6351	661	10
329	Neu5Aca2-6Galb1-4GlcNAcb1-3Galb1-4GlcNAcb1-3Galb1-4GlcNAcb-Sp0	13	1	6
330	Gala1-4Galb1-4GlcNAcb1-3Galb1-4Glcb-Sp0	13	1	10
331	GalNAcb1-3Gala1-4Galb1-4GlcNAcb1-3Galb1-4Glcb-Sp0	49	7	15
332	GalNAca1-3(Fuca1-2)Galb1-4GlcNAcb1-3Galb1-4GlcNAcb-Sp0	15	2	15
333	GalNAca1-3(Fuca1-2)Galb1-4GlcNAcb1-3Galb1-4GlcNAcb1-3Galb1-4GlcNAcb-Sp0	21	1	7
334	Neu5Aca2-3Galb1-4(Fuca1-3)GlcNAcb1-6(Neu5Aca2-3Galb1-3)GalNAc-Sp14	30	2	5
335	GlcNAca1-4Galb1-4GlcNAcb1-3Galb1-4GlcNAcb1-3Galb1-4GlcNAcb-Sp0	3	3	88
336	GlcNAca1-4Galb1-4GlcNAcb-Sp0	82	61	74
337	GlcNAca1-4Galb1-3GlcNAcb-Sp0	27	1	5
338	GlcNAca1-4Galb1-4GlcNAcb1-3Galb1-4Glcb-Sp0	33	5	14
339	GlcNAca1-4Galb1-4GlcNAcb1-3Galb1-4(Fuca1-3)GlcNAcb1-3Galb1-4(Fuca1-3)GlcNAcb-Sp0	62	4	6
340	GlcNAca1-4Galb1-4GlcNAcb1-3Galb1-4GlcNAcb-Sp0	35	6	18
341	GlcNAca1-4Galb1-3GalNAc-Sp14	27	3	10
342	Neu5Aca2-6Galb1-4GlcNAcb1-2Mana1-6(Mana1-3)Manb1-4GlcNAcb1-4GlcNAc-Sp12	22	2	8
343	Mana1-6(Neu5Aca2-6Galb1-4GlcNAcb1-2Mana1-3)Manb1-4GlcNAcb1-4GlcNAc-Sp12	20	2	8
344	Neu5Aca2-6Galb1-4GlcNAcb1-2Mana1-6Manb1-4GlcNAcb1-4GlcNAc-Sp12	25	5	20
345	Neu5Aca2-6Galb1-4GlcNAcb1-2Mana1-3Manb1-4GlcNAcb1-4GlcNAc-Sp12	19	2	9
346	Galb1-4GlcNAcb1-2Mana1-3Manb1-4GlcNAcb1-4GlcNAc-Sp12	19	2	9
347	Galb1-4GlcNAcb1-2Mana1-6Manb1-4GlcNAcb1-4GlcNAc-Sp12	25	3	11
348	Mana1-6(Galb1-4GlcNAcb1-2Mana1-3)Manb1-4GlcNAcb1-4GlcNAcb-Sp12	29	5	17
349	GlcNAcb1-2Mana1-6(GlcNAcb1-2Mana1-3)Manb1-4GlcNAcb1-4(Fuca1-6)GlcNAcb-Sp22	30	2	6
350	Galb1-4GlcNAcb1-2Mana1-6(Galb1-4GlcNAcb1-2Mana1-3)Manb1-4GlcNAcb1-4(Fuca1-6)GlcNAcb-Sp22	33	3	8
351	Galb1-3GlcNAcb1-2Mana1-6(Galb1-3GlcNAcb1-2Mana1-3)Manb1-4GlcNAcb1-4(Fuca1-6)GlcNAcb-Sp22	24	1	4
352	(6S)GlcNAcb1-3Galb1-4GlcNAcb-Sp0	29	2	6
353	KDNa2-3Galb1-4(Fuca1-3)GlcNAc-Sp0	18	4	23
354	KDNa2-6Galb1-4GlcNAc-Sp0	52	13	25
355	KDNa2-3Galb1-4Glc-Sp0	28	5	18

537	GlcNAcb1-3Galb1-4GlcNAcb1-3Galb1-4GlcNAcb1-2Mana1-6(GlcNAcb1-3Galb1-4GlcNAcb1-3Galb1-4GlcNAcb1-2Mana1-3)Manb1-4GlcNAcb1-4GlcNAcb-Sp12	30	4	12
561	Galb1-3GlcNAcb1-3Galb1-4GlcNAcb1-3Galb1-4GlcNAcb1-6(Galb1-3GlcNAcb1-3Galb1-4GlcNAcb1-3Galb1-4GlcNAb1-2)Mana1-6(Galb1-3GlcNAcb1-3Galb1-4GlcNAcb1-3Galb1-4GlcNAcb1-2Mana1-3)Manb1-4GlcNAcb1-4(Fuca1-6)GlcNAcb-Sp24	30	4	14
569	Galb1-4GlcNAcb1-3Galb1-4GlcNAcb1-3Galb1-4GlcNAcb1-3Galb1-4GlcNAcb1-2Mana1-6(Galb1-4GlcNAcb1-3Galb1-4GlcNAcb1-3Galb1-4GlcNAcb1-3Galb1-4GlcNAcb1-2Mana1-3)Manb1-4GlcNAcb1-4(Fuca1-6)GlcNAcb-Sp24	30	2	5
188	GlcNAcb1-4Galb1-4GlcNAcb-Sp8	30	7	24
222	Fuca1-2(6S)Galb1-4(6S)Glcb-Sp0	30	1	5
349	GlcNAcb1-2Mana1-6(GlcNAcb1-2Mana1-3)Manb1-4GlcNAcb1-4(Fuca1-6)GlcNAcb-Sp22	30	2	6
455	Neu5Aca2-3Galb1-4GlcNAcb1-2Mana1-6(GlcNAcb1-4)(Neu5Aca2-3Galb1-4GlcNAcb1-2Mana1-3)Manb1-4GlcNAcb1-4GlcNAcb-Sp21	30	21	71
558	Galb1-4GlcNAcb1-3Galb1-4GlcNAcb1-3Galb1-4GlcNAcb1-3Galb1-4GlcNAcb1-3Galb1-4GlcNAcb1-3Galb1-4GlcNAcb1-2Mana1-6(Galb1-4GlcNAcb1-3Galb1-4GlcNAcb1-3Galb1-4GlcNAcb1-3Galb1-4GlcNAcb1-3Galb1-4GlcNAcb1-3Galb1-4GlcNAcb1-2Mana1-3)Manb1-4GlcNAcb1-4GlcNAcb-Sp25	30	7	24
38	(3S)Galb-Sp8	30	4	13
228	GalNAcb1-4(Neu5Aca2-8Neu5Aca2-3)Galb1-4Glcb-Sp0	30	6	19
549	Galb1-4GlcNAcb1-3Galb1-4GlcNAcb1-6(Galb1-4GlcNAcb1-3Galb1-4GlcNAcb1-2)Mana1-6(Galb1-4GlcNAcb1-3Galb1-4GlcNAcb1-2Mana1-3)Mana1-4GlcNAcb1-4GlcNAc-Sp24	30	6	20
562	Galb1-3GlcNAcb1-3Galb1-4GlcNAcb1-6(Galb1-3GlcNAcb1-3Galb1-4GlcNAb1-2)Mana1-6(Galb1-3GlcNAcb1-3Galb1-4GlcNAcb1-2Mana1-3)Manb1-4GlcNAcb1-4(Fuca1-6)GlcNAcb-Sp24	30	6	21
199	Glcb1-6Glcb-Sp8	30	3	12
491	Galb1-4(Fuca1-3)GlcNAcb1-2Mana-Sp0	30	3	10
570	GlcNAcb1-3Galb1-4GlcNAcb1-3Galb1-4GlcNAcb1-3Galb1-4GlcNAcb1-3Galb1-4GlcNAcb1-2Mana1-6(GlcNAcb1-3Galb1-4GlcNAcb1-3Galb1-4GlcNAcb1-3Galb1-4GlcNAcb1-3Galb1-4GlcNAcb1-2Mana1-3)Manb1-4GlcNAcb1-4(Fuca1-6)GlcNAcb-Sp19	30	2	6
580	Galb1-4GlcNAcb1-3Galb1-4GlcNAcb1-6(Galb1-3)GalNAca-Sp14	30	3	10
113	Gala1-3Galb1-4(Fuca1-3)GlcNAcb-Sp8	29	6	20
400	Gala1-4Galb1-4GlcNAcb1-2Mana1-6(Gala1-4Galb1-4GlcNAcb1-2Mana1-3)Manb1-4GlcNAcb1-4GlcNAcb-Sp24	29	6	21
488	Galb1-4(Fuca1-3)GlcNAcb1-6(Neu5Aca2-6(Neu5Aca2-3Galb1-3)GlcNAcb1-3)Galb1-4Glc-Sp21	29	2	5
211	Mana1-2Mana1-6(Mana1-3)Mana1-6(Mana1-2Mana1-2Mana1-3)Manb1-4GlcNAcb1-4GlcNAcb-Sp12	29	13	45
251	Neu5Aca2-3Galb1-4(Fuca1-3)GlcNAcb1-3Galb1-4(Fuca1-3)GlcNAcb1-3Galb1-4(Fuca1-3)GlcNAcb-Sp0	29	4	14
296	(6P)Glcb-Sp10	29	3	10
352	(6S)GlcNAcb1-3Galb1-4GlcNAcb-Sp0	29	2	6
381	Galb1-4(Fuca1-3)GlcNAcb1-6(Fuca1-4(Fuca1-2Galb1-3)GlcNAcb1-3)Galb1-4Glc-Sp21	29	5	17
84	(3S)Galb1-4(Fuca1-3)Glcb-Sp0	29	3	10
130	Fuca1-4(Galb1-3)GlcNAcb-Sp8	29	2	7
265	Neu5Aca2-6Galb1-4(6S)GlcNAcb-Sp8	29	5	17
348	Mana1-6(Galb1-4GlcNAcb1-2Mana1-3)Manb1-4GlcNAcb1-4GlcNAcb-Sp12	29	5	17
450	GalNAca1-3(Fuca1-2)Galb1-4GlcNAcb1-2Mana1-6(GalNAca1-3(Fuca1-2)Galb1-4GlcNAcb1-2Mana1-3)Manb1-4GlcNAcb1-4(Fuca1-6)GlcNAcb-Sp22	29	2	6
454	Galb1-4GlcNAcb1-6(Galb1-4GlcNAcb1-2)Mana1-6(Galb1-4GlcNAcb1-2Mana1-3)Manb1-4GlcNAcb1-4GlcNAcb-Sp19	29	3	9
473	Neu5Aca2-3Galb1-4GlcNAcb1-2Mana-Sp0	29	9	31
445	Fuca1-2Galb1-4GlcNAcb1-6(Fuca1-2Galb1-4GlcNAcb1-3)GalNAc-Sp14	29	3	9
486	Neu5Aca2-3Galb1-3GlcNAcb1-6GalNAca-Sp14	29	5	18
504	Galb1-3GlcNAca1-3Galb1-4GlcNAcb-Sp8	29	11	38
523	GalNAcb1-4GlcNAcb1-2Mana-Sp0	29	9	30
60	Fuca1-2Galb1-3(Fuca1-4)GlcNAcb-Sp8	28	1	4
148	Galb1-3GlcNAcb1-3Galb1-4Glcb-Sp10	28	7	25
153	Galb1-4(Fuca1-3)GlcNAcb1-3Galb1-4(Fuca1-3)GlcNAcb-Sp0	28	2	7
194	GlcNAcb1-6Galb1-4GlcNAcb-Sp8	28	3	11
36	(3S)Galb1-4GlcNAcb-Sp0	28	2	9
37	(3S)Galb1-4GlcNAcb-Sp8	28	5	18
48	Neu5,9Ac$_2$a-Sp8	28	7	26
52	GlcNAcb1-2Mana1-6(GlcNAcb1-2Mana1-3)Manb1-4GlcNAcb1-4GlcNAcb-Sp12	28	6	20
122	Gala1-4Galb1-4Glcb-Sp0	28	6	20
355	KDNa2-3Galb1-4Glc-Sp0	28	5	18
593	Neu5Aca2-3Galb1-4GlcNAcb1-3Galb1-4GlcNAcb1-2Mana1-6(Neu5Aca2-3Galb1-4GlcNAcb1-3Galb1-4GlcNAcb1-2Mana1-3)Manb1-4GlcNAcb1-4GlcNAcb-Sp12	28	3	11
76	Fuca1-2Galb1-4GlcNAcb-Sp8	28	5	18
210	Mana1-6(Mana1-2Mana1-3)Mana1-6(Mana1-2Mana1-3)Manb1-4GlcNAcb1-4GlcNAcb-Sp12	28	4	16
595	Neu5Aca2-6Galb1-4GlcNAcb1-3Galb1-4GlcNAcb1-6(Neu5Aca2-6Galb1-4GlcNAcb1-3Galb1-4GlcNAcb1-3)GalNAca-Sp14	28	3	10
101	Gala1-3(Fuca1-2)Galb1-3GlcNAcb-Sp0	28	2	6
106	Gala1-3(Fuca1-2)Galb1-4Glcb-Sp0	28	9	32
200	G-ol-Sp8	28	3	11
390	Gala1-3Galb1-3GlcNAcb1-2Mana1-6(Gala1-3Galb1-3GlcNAcb1-2Mana1-3)Manb1-4GlcNAcb1-4GlcNAc-Sp19	28	2	9
39	(6S)(4S)Galb1-4GlcNAcb-Sp0	27	3	11
158	Galb1-4GalNAcb1-3(Fuca1-2)Galb1-4GlcNAcb-Sp8	27	3	11
298	Galb1-3Galb1-4GlcNAcb-Sp8	27	3	11
420	GalNAca1-3(Fuca1-2)Galb1-3GlcNAcb1-3GalNAc-Sp14	27	2	8
79	Fuca1-3GlcNAcb-Sp8	27	5	18
80	Fuca1-4GlcNAcb-Sp8	27	2	6
91	GalNAca1-3GalNAcb-Sp8	27	4	15
233	Neu5Aca2-3Galb1-3GalNAcb1-4(Neu5Aca2-3)Galb1-4Glcb-Sp0	27	12	46
283	Neu5Gca2-6GalNAca-Sp0	27	3	10
341	GlcNAca1-4Galb1-3GalNAc-Sp14	27	3	10
412	Fuca1-2Galb1-4(Fuca1-3)GlcNAcb1-3GalNAca-Sp14	27	1	5
466	Glca1-4Glca1-4Glca1-4Glcb-Sp10	27	4	16
103	Gala1-3(Fuca1-2)Galb1-4(Fuca1-3)GlcNAcb-Sp0	27	2	6
129	Galb1-3(Fuca1-4)GlcNAc-Sp8	27	6	21
179	GlcNAcb1-3GalNAca-Sp8	27	2	8
299	Neu5Aca2-6Galb1-4GlcNAcb1-2Mana1-6(Galb1-4GlcNAcb1-2Mana1-3)Manb1-4GlcNAcb1-4GlcNAcb-Sp12	27	3	13
337	GlcNAca1-4Galb1-3GlcNAcb-Sp0	27	1	5
356	KDNa2-3Galb1-3GalNAca-Sp14	27	4	13
394	Galb1-4GlcNAcb1-2Mana1-6(GlcNAcb1-2Mana1-3)Manb1-4GlcNAcb1-4GlcNAc-Sp12	27	5	17
423	Gala1-3(Fuca1-2)Galb1-4GlcNAcb1-2Mana1-6(Gala1-3(Fuca1-2)Galb1-4GlcNAcb1-2Mana1-3)Manb1-4GlcNAcb1-4(Fuca1-6)GlcNAcb-Sp22	27	2	6
451	Gala1-3(Fuca1-2)Galb1-3GlcNAcb1-2Mana1-6(Gala1-3(Fuca1-2)Galb1-3GlcNAcb1-2Mana1-3)Manb1-4GlcNAcb1-4(Fuca1-6)GlcNAcb-Sp22	27	2	9
143	Galb1-3GalNAcb1-3Gala1-4Galb1-4Glcb-Sp0	27	4	16
485	Galb1-3(Fuca1-4)GlcNAcb1-6GalNAca-Sp14	27	8	29
598	Neu5Aca2-6Galb1-4GlcNAcb1-3Galb1-4GlcNAcb1-2Mana1-6(Neu5Aca2-6Galb1-4GlcNAcb1-3Galb1-4GlcNAcb1-2Mana1-3)Manb1-4GlcNAcb1-4GlcNAcb-Sp12	27	1	5
68	Fuca1-2Galb1-3GlcNAcb-Sp8	26	2	8
392	Neu5Aca2-3Galb1-3GlcNAcb1-2Mana1-6(Neu5Aca2-3Galb1-3GlcNAcb1-2Mana1-3)Manb1-4GlcNAcb1-4GlcNAc-Sp19	26	6	21
156	Galb1-4(6S)Glcb-Sp8	26	1	3
289	Galb1-4(Fuca1-3)(6S)Glcb-Sp0	26	1	3
550	Gala1-3Galb1-4GlcNAcb1-2Mana1-6(Gala1-3Galb1-4GlcNAcb1-2Mana1-3)Manb1-4GlcNAcb1-4GlcNAc-Sp24	26	2	8
579	Galb1-4GlcNAcb1-3Galb1-4GlcNAcb1-3GalNAca-Sp14	26	2	9
225	GalNAcb1-4(Neu5Aca2-8Neu5Aca2-8Neu5Aca2-8Neu5Aca2-3)Galb1-4Glcb-Sp0	26	2	6
245	Neu5Aca2-3Galb1-3GalNAcb1-3Gala1-4Galb1-4Glcb-Sp0	26	2	7
293	Neu5Aca2-3Galb1-4GlcNAcb1-3Galb1-3GlcNAcb-Sp0	26	2	8
366	Galb1-4GlcNAcb1-2Mana1-6(Galb1-4GlcNAcb1-4(Galb1-4GlcNAcb1-2)Mana1-3)Manb1-4GlcNAcb1-4GlcNAc-Sp21	26	1	5
452	Neu5Aca2-6Galb1-4GlcNAcb1-6(Fuca1-2Galb1-3GlcNAcb1-3)Galb1-4Glc-Sp21	26	6	24
583	GlcNAcb1-3Galb1-4GlcNAcb1-3GalNAca-Sp14	26	3	13
171	Galb1-4Glcb-Sp0	26	1	4
244	Neu5Aca2-3Galb-Sp8	26	4	15
453	GalNAca1-3(Fuca1-2)Galb1-3GlcNAcb1-2Mana1-6(GalNAca1-3(Fuca1-2)Galb1-3GlcNAcb1-2Mana1-3)Manb1-4GlcNAcb1-4(Fuca1-6)GlcNAcb-Sp22	26	2	7
463	Gala1-3(Fuca1-2)Galb1-3GalNAca-Sp8	26	8	30
126	Galb1-3(Fuca1-4)GlcNAcb1-3Galb1-4(Fuca1-3)GlcNAcb-Sp0	25	3	13
127	Galb1-3GlcNAcb1-3Galb1-4(Fuca1-3)GlcNAcb-Sp0	25	2	8
294	4S(3S)Galb1-4GlcNAcb-Sp0	25	1	5
295	(6S)Galb1-4(6S)GlcNAcb-Sp0	25	9	34
307	GlcNAcb1-3Man-Sp10	25	3	11
404	GalNAcb1-3Gala1-6Galb1-4Glcb-Sp8	25	3	13
490	Gala1-3Galb1-4GlcNAcb1-6GalNAca-Sp14	25	8	31
496	Fuca1-2(6S)Galb1-3(6S)GlcNAcb-Sp0	25	2	6
531	GalNAca1-3(Fuca1-2)Galb1-3GalNAcb1-3Gala1-4Galb1-4Glc-Sp21	25	4	16
85	GalNAca1-3(Fuca1-2)Galb1-4GlcNAcb-Sp0	25	1	6
195	Glca1-4Glcb-Sp8	25	4	16
260	Fuca1-2Galb1-4(6S)Glcb-Sp0	25	1	6
344	Neu5Aca2-6Galb1-4GlcNAcb1-2Mana1-6Manb1-4GlcNAcb1-4GlcNAc-Sp12	25	5	20
347	Galb1-4GlcNAcb1-2Mana1-6Manb1-4GlcNAcb1-4GlcNAc-Sp12	25	3	11
406	Galb1-4GlcNAcb1-6(Neu5Aca2-6Galb1-3GlcNAcb1-3)Galb1-4Glc-Sp21	25	3	11
439	(6S)Galb1-3GlcNAcb-Sp0	25	4	15
62	Fuca1-2Galb1-3GalNAca-Sp14	25	2	7
160	Galb1-4GlcNAcb1-3GalNAc-Sp14	25	1	5
254	Neu5Aca2-3Galb1-4(Fuca1-3)GlcNAcb1-3Galb-Sp8	25	2	9
370	GalNAca1-3(Fuca1-2)Galb1-3GlcNAcb1-2Mana1-6(GalNAca1-3(Fuca1-2)Galb1-3GlcNAcb1-2Mana1-3)Manb1-4GlcNAcb1-4GlcNAcb-Sp20	25	3	11
414	GalNAca1-3(Fuca1-2)Galb1-4(Fuca1-3)GlcNAcb1-3GalNAc-Sp14	25	4	15
431	Galb1-4GlcNAcb1-2Mana1-6(GlcNAcb1-4)(Galb1-4GlcNAcb1-2Mana1-3)Manb1-4GlcNAcb1-4GlcNAc-Sp21	25	5	20
461	Neu5Aca2-6Galb1-4GlcNAcb1-6(Neu5Aca2-6Galb1-4GlcNAcb1-2)Mana1-6(GlcNAcb1-4)(Neu5Aca2-6Galb1-4GlcNAcb1-2Mana1-3)Manb1-4GlcNAcb1-4GlcNAcb-Sp21	25	4	15
511	(4S)GalNAcb-Sp10	25	2	6

356	KDNa2-3Galb1-3GalNAca-Sp14	27	4	13
357	Fuca1-2Galb1-3GlcNAcb1-2Mana1-6(Fuca1-2Galb1-3GlcNAcb1-2Mana1-3)Manb1-4GlcNAcb1-4GlcNAcb-Sp20	42	1	3
358	Fuca1-2Galb1-4GlcNAcb1-2Mana1-6(Fuca1-2Galb1-4GlcNAcb1-2Mana1-3)Manb1-4GlcNAcb1-4GlcNAcb-Sp20	11	19	181
359	Fuca1-2Galb1-4(Fuca1-3)GlcNAcb1-2Mana1-6(Fuca1-2Galb1-4(Fuca1-3)GlcNAcb1-2Mana1-3)Manb1-4GlcNAcb1-4GlcNAb-Sp20	242	295	122
360	Gala1-3Galb1-4GlcNAcb1-2Mana1-6(Gala1-3Galb1-4GlcNAcb1-2Mana1-3)Manb1-4GlcNAcb1-4GlcNAcb-Sp20	21	1	5
361	Galb1-4GlcNAcb1-2Mana1-6(Mana1-3)Manb1-4GlcNAcb1-4GlcNAcb-Sp12	36	4	11
362	Fuca1-4(Galb1-3)GlcNAcb1-2Mana1-6(Fuca1-4(Galb1-3)GlcNAcb1-2Mana1-3)Manb1-4GlcNAcb1-4(Fuca1-6)GlcNAcb-Sp22	48	1	2
363	Neu5Aca2-6GlcNAcb1-4GlcNAc-Sp21	21	4	17
364	Neu5Aca2-6GlcNAcb1-4GlcNAcb1-4GlcNAc-Sp21	18	2	9
365	Galb1-4(Fuca1-3)GlcNAcb1-6(Fuca1-2Galb1-4GlcNAcb1-3)Galb1-4Glc-Sp21	36	8	24
366	Galb1-4GlcNAcb1-2Mana1-6(Galb1-4GlcNAcb1-4(Galb1-4GlcNAcb1-2)Mana1-3)Manb1-4GlcNAcb1-4GlcNAc-Sp21	26	1	5
367	GalNAca1-3(Fuca1-2)Galb1-4GlcNAcb1-2Mana1-6(GalNAca1-3(Fuca1-2)Galb1-4GlcNAcb1-2Mana1-3)Manb1-4GlcNAcb1-4GlcNAcb-Sp20	32	3	8
368	Gala1-3(Fuca1-2)Galb1-4GlcNAcb1-2Mana1-6(Gala1-3(Fuca1-2)Galb1-4GlcNAcb1-2Mana1-3)Manb1-4GlcNAcb1-4GlcNAcb-Sp20	43	3	7
369	Gala1-3Galb1-4(Fuca1-3)GlcNAcb1-2Mana1-6(Gala1-3Galb1-4(Fuca1-3)GlcNAcb1-2Mana1-3)Manb1-4GlcNAcb1-4GlcNAcb-Sp20	67	10	16
370	GalNAca1-3(Fuca1-2)Galb1-3GlcNAcb1-2Mana1-6(GalNAca1-3(Fuca1-2)Galb1-3GlcNAcb1-2Mana1-3)Manb1-4GlcNAcb1-4GlcNAcb-Sp20	25	3	11
371	Fuca1-4(Fuca1-2Galb1-3)GlcNAcb1-2Mana1-3(Fuca1-4(Fuca1-2Galb1-3)GlcNAcb1-2Mana1-3)Manb1-4GlcNAcb1-4GlcNAcb-Sp19	35	7	21
372	Neu5Aca2-3Galb1-4GlcNAcb1-3GalNAc-Sp14	20	1	3
373	Neu5Aca2-6Galb1-4GlcNAcb1-3GalNAc-Sp14	31	2	7
374	Neu5Aca2-3Galb1-4(Fuca1-3)GlcNAcb1-3GalNAca-Sp14	39	6	16
375	GalNAcb1-4GlcNAcb1-2Mana1-6(GalNAcb1-4GlcNAcb1-2Mana1-3)Manb1-4GlcNAcb1-4GlcNAc-Sp12	39	8	22
376	Galb1-3GalNAca1-3(Fuca1-2)Galb1-4Glc-Sp0	15	1	9
377	Galb1-3GalNAca1-3(Fuca1-2)Galb1-4GlcNAc-Sp0	17	2	10
378	Galb1-3GlcNAcb1-3Galb1-4GlcNAcb1-6(Galb1-3GlcNAcb1-3)Galb1-4Glcb-Sp0	22	4	17
379	Galb1-4(Fuca1-3)GlcNAcb1-6(Galb1-3GlcNAcb1-3)Galb1-4Glc-Sp21	24	4	16
380	Galb1-4GlcNAcb1-6(Fuca1-4(Fuca1-2Galb1-3)GlcNAcb1-3)Galb1-4Glc-Sp21	33	2	7
381	Galb1-4(Fuca1-3)GlcNAcb1-6(Fuca1-4(Fuca1-2Galb1-3)GlcNAcb1-3)Galb1-4Glc-Sp21	29	5	17
382	Galb1-3GlcNAcb1-3Galb1-4(Fuca1-3)GlcNAcb1-6(Galb1-3GlcNAcb1-3)Galb1-4Glc-Sp21	17	2	10
383	Galb1-4GlcNAcb1-6(Galb1-4GlcNAcb1-2)Mana1-6(Galb1-4GlcNAcb1-4(Galb1-4GlcNAcb1-2)Mana1-3)Manb1-4GlcNAcb1-4GlcNAcb-Sp21	17	1	5
384	GlcNAcb1-2Mana1-6(GlcNAcb1-4(GlcNAcb1-2)Mana1-3)Manb1-4GlcNAcb1-4GlcNAc-Sp21	19	4	21
385	Fuca1-2Galb1-3GalNAca1-3(Fuca1-2)Galb1-4Glcb-Sp0	31	20	63
386	Fuca1-2Galb1-3GalNAca1-3(Fuca1-2)Galb1-4GlcNAcb-Sp0	13	3	23
387	Galb1-3GlcNAcb1-3GalNAca-Sp14	17	5	32
388	GalNAcb1-4(Neu5Aca2-3)Galb1-4GlcNAcb1-3GalNAca-Sp14	24	4	17
389	GalNAca1-3(Fuca1-2)Galb1-3GalNAca1-3(Fuca1-2)Galb1-4GlcNAcb-Sp0	16	4	25
390	Gala1-3Galb1-3GlcNAcb1-2Mana1-6(Gala1-3Galb1-3GlcNAcb1-2Mana1-3)Manb1-4GlcNAcb1-4GlcNAc-Sp19	28	2	9
391	Gala1-3Galb1-3(Fuca1-4)GlcNAcb1-2Mana1-6(Gala1-3Galb1-3(Fuca1-4)GlcNAcb1-2Mana1-3)Manb1-4GlcNAcb1-4GlcNAc-Sp19	40	6	16
392	Neu5Aca2-3Galb1-3GlcNAcb1-2Mana1-6(Neu5Aca2-3Galb1-3GlcNAcb1-2Mana1-3)Manb1-4GlcNAcb1-4GlcNAc-Sp19	26	6	21
393	GlcNAcb1-2Mana1-6(Galb1-4GlcNAcb1-2Mana1-3)Manb1-4GlcNAcb1-4GlcNAc-Sp12	16	3	16
394	Galb1-4GlcNAcb1-2Mana1-6(GlcNAcb1-2Mana1-3)Manb1-4GlcNAcb1-4GlcNAc-Sp12	27	5	17
395	Neu5Aca2-3Galb1-3GlcNAcb1-3GalNAca-Sp14	19	2	11
396	Fuca1-2Galb1-4GlcNAcb1-3GalNAca-Sp14	31	5	16
397	Galb1-4(Fuca1-3)GlcNAcb1-3GalNAca-Sp14	31	1	4
398	GalNAca1-3GalNAcb1-3Gala1-4Galb1-4GlcNAcb-Sp0	22	2	10
399	Gala1-4Galb1-3GlcNAcb1-2Mana1-6(Gala1-4Galb1-3GlcNAcb1-2Mana1-3)Manb1-4GlcNAcb1-4GlcNAcb-Sp19	19	3	15
400	Gala1-4Galb1-4GlcNAcb1-2Mana1-6(Gala1-4Galb1-4GlcNAcb1-2Mana1-3)Manb1-4GlcNAcb1-4GlcNAcb-Sp24	29	6	21
401	Gala1-3Galb1-4GlcNAcb1-3GalNAca-Sp14	19	1	7
402	Galb1-3GlcNAcb1-6Galb1-4GlcNAcb-Sp0	24	5	20
403	Galb1-3GlcNAca1-6Galb1-4GlcNAcb-Sp0	13	3	20
404	GalNAcb1-3Gala1-6Galb1-4Glcb-Sp8	25	3	13
405	Gala1-3(Fuca1-2)Galb1-4(Fuca1-3)Glcb-Sp21	21	2	10
406	Galb1-4GlcNAcb1-6(Neu5Aca2-6Galb1-3GlcNAcb1-3)Galb1-4Glc-Sp21	25	3	11
407	Galb1-3GalNAcb1-4(Neu5Aca2-8Neu5Aca2-3)Galb1-4Glcb-Sp0	51	4	8
408	Neu5Aca2-3Galb1-3GalNAcb1-4(Neu5Aca2-8Neu5Aca2-3)Galb1-4Glcb-Sp0	36	9	24
409	Gala1-3(Fuca1-2)Galb1-4GlcNAcb1-3GalNAca-Sp14	18	5	27
410	GalNAca1-3(Fuca1-2)Galb1-4GlcNAcb1-3GalNAca-Sp14	17	1	6
411	GalNAca1-3GalNAcb1-3Gala1-4Galb1-4Glcb-Sp0	18	7	39
412	Fuca1-2Galb1-4(Fuca1-3)GlcNAcb1-3GalNAca-Sp14	27	1	5
413	Gala1-3(Fuca1-2)Galb1-4(Fuca1-3)GlcNAcb1-3GalNAc-Sp14	20	7	36
414	GalNAca1-3(Fuca1-2)Galb1-4(Fuca1-3)GlcNAcb1-3GalNAc-Sp14	25	4	15
415	Galb1-4(Fuca1-3)GlcNAcb1-2Mana1-6(Galb1-4(Fuca1-3)GlcNAcb1-2Mana1-3)Manb1-4GlcNAcb1-4(Fuca1-6)GlcNAcb-Sp22	31	5	17
416	Fuca1-2Galb1-4GlcNAcb1-2Mana1-6(Fuca1-2Galb1-4GlcNAcb1-2Mana1-3)Manb1-4GlcNAcb1-4(Fuca1-6)GlcNAcb-Sp22	41	3	6
417	GlcNAcb1-2(GlcNAcb1-6)Mana1-6(GlcNAcb1-2Mana1-3)Manb1-4GlcNAcb1-4GlcNAcb-Sp19	35	2	6
418	Fuca1-2Galb1-3GlcNAcb1-3GalNAc-Sp14	33	12	36
419	Gala1-3(Fuca1-2)Galb1-3GlcNAcb1-3GalNAc-Sp14	10	3	33
420	GalNAca1-3(Fuca1-2)Galb1-3GlcNAcb1-3GalNAc-Sp14	27	2	8
421	Gala1-3Galb1-3GlcNAcb1-3GalNAc-Sp14	34	5	16
422	Fuca1-2Galb1-3GlcNAcb1-2Mana1-6(Fuca1-2Galb1-3GlcNAcb1-2Mana1-3)Manb1-4GlcNAcb1-4(Fuca1-6)GlcNAcb-Sp22	36	7	18
423	Gala1-3(Fuca1-2)Galb1-4GlcNAcb1-2Mana1-6(Gala1-3(Fuca1-2)Galb1-4GlcNAcb1-2Mana1-3)Manb1-4GlcNAcb1-4(Fuca1-6)GlcNAcb-Sp22	27	2	6
424	Galb1-3GlcNAcb1-6(Galb1-3GlcNAcb1-2)Mana1-6(Galb1-3GlcNAcb1-2Mana1-3)Manb1-4GlcNAcb1-4GlcNAcb-Sp19	32	5	15
425	Galb1-4GlcNAcb1-6(Fuca1-2Galb1-3GlcNAcb1-3)Galb1-4Glc-Sp21	19	2	10
426	Fuca1-3GlcNAcb1-6(Galb1-4GlcNAcb1-3)Galb1-4Glc-Sp21	22	3	15
427	GlcNAcb1-2Mana1-6(GlcNAcb1-4)(GlcNAcb1-2Mana1-3)Manb1-4GlcNAcb1-4GlcNAc-Sp21	14	3	18
428	GlcNAcb1-2Mana1-6(GlcNAcb1-4)(GlcNAcb1-4(GlcNAcb1-2)Mana1-3)Manb1-4GlcNAcb1-4GlcNAc-Sp21	16	1	4
429	GlcNAcb1-6(GlcNAcb1-2)Mana1-6(GlcNAcb1-4)(GlcNAcb1-2Mana1-3)Manb1-4GlcNAcb1-4GlcNAc-Sp21	19	3	15
430	GlcNAcb1-6(GlcNAcb1-2)Mana1-6(GlcNAcb1-4)(GlcNAcb1-4(GlcNAcb1-2)Mana1-3)Manb1-4GlcNAcb1-4GlcNAc-Sp21	18	4	23
431	Galb1-4GlcNAcb1-2Mana1-6(GlcNAcb1-4)(Galb1-4GlcNAcb1-2Mana1-3)Manb1-4GlcNAcb1-4GlcNAc-Sp21	25	5	20
432	Galb1-4GlcNAcb1-2Mana1-6(GlcNAcb1-4)(Galb1-4GlcNAcb1-4(Galb1-4GlcNAcb1-2)Mana1-3)Manb1-4GlcNAcb1-4GlcNAc-Sp21	21	3	15
433	Galb1-4GlcNAcb1-6(Galb1-4GlcNAcb1-2)Mana1-6(GlcNAcb1-4)(Galb1-4GlcNAcb1-2Mana1-3)Manb1-4GlcNAcb1-4GlcNAc-Sp21	20	10	52
434	Galb1-4GlcNAcb1-6(Galb1-4GlcNAcb1-2)Mana1-6(GlcNAcb1-4)(Galb1-4GlcNAcb1-4(Galb1-4GlcNAcb1-2)Mana1-3)Manb1-4GlcNAcb1-4GlcNAc-Sp21	16	1	6
435	Galb1-4Galb-Sp10	15	6	41
436	Galb1-6Galb-Sp10	19	3	16
437	Neu5Aca2-3Galb1-4GlcNAcb1-3Galb-Sp8	19	2	13
438	GalNAcb1-6GalNAcb-Sp8	17	2	9
439	(6S)Galb1-3GlcNAcb-Sp0	25	4	15
440	(6S)Galb1-3(6S)GlcNAc-Sp0	79	33	41
441	Fuca1-2Galb1-4 GlcNAcb1-2Mana1-6(Fuca1-2Galb1-4GlcNAcb1-2(Fuca1-2Galb1-4GlcNAcb1-4)Mana1-3)Manb1-4GlcNAcb1-4GlcNAcb-Sp12	40	1	3
442	Fuca1-2Galb1-4(Fuca1-3)GlcNAcb1-2Mana1-6(Fuca1-2Galb1-4(Fuca1-3)GlcNAcb1-4(Fuca1-2Galb1-4(Fuca1-3)GlcNAcb1-2)Mana1-3)Manb1-4GlcNAcb1-4GlcNAcb-Sp12	39	4	11
443	Galb1-4(Fuca1-3)GlcNAcb1-6GalNAc-Sp14	23	2	7
444	Galb1-4GlcNAcb1-2Mana-Sp0	37	7	19
445	Fuca1-2Galb1-4GlcNAcb1-6(Fuca1-2Galb1-4GlcNAcb1-3)GalNAc-Sp14	29	3	9
446	Gala1-3(Fuca1-2)Galb1-4GlcNAcb1-6(Gala1-3(Fuca1-2)Galb1-4GlcNAcb1-3)GalNAc-Sp14	21	4	18
447	GalNAca1-3(Fuca1-2)Galb1-4GlcNAcb1-6(GalNAca1-3(Fuca1-2)Galb1-4GlcNAcb1-3)GalNAc-Sp14	15	1	9
448	Neu5Aca2-8Neu5Aca2-3Galb1-3GalNAcb1-4(Neu5Aca2-8Neu5Aca2-3)Galb1-4Glcb-Sp0	34	2	5
449	GalNAcb1-4Galb1-4Glcb-Sp0	40	2	4
450	GalNAca1-3(Fuca1-2)Galb1-4GlcNAcb1-2Mana1-6(GalNAca1-3(Fuca1-2)Galb1-4GlcNAcb1-2Mana1-3)Manb1-4GlcNAcb1-4(Fuca1-6)GlcNAcb-Sp22	29	2	6
451	Gala1-3(Fuca1-2)Galb1-3GlcNAcb1-2Mana1-6(Gala1-3(Fuca1-2)Galb1-3GlcNAcb1-2Mana1-3)Manb1-4GlcNAcb1-4(Fuca1-6)GlcNAcb-Sp22	27	2	9
452	Neu5Aca2-6Galb1-4GlcNAcb1-6(Fuca1-2Galb1-3GlcNAcb1-3)Galb1-4Glc-Sp21	26	6	24
453	GalNAca1-3(Fuca1-2)Galb1-3GlcNAcb1-2Mana1-6(GalNAca1-3(Fuca1-2)Galb1-3GlcNAcb1-2Mana1-3)Manb1-4GlcNAcb1-4(Fuca1-6)GlcNAcb-Sp22	26	2	7
454	Galb1-4GlcNAcb1-6(Galb1-4GlcNAcb1-2)Mana1-6(Galb1-4GlcNAcb1-2Mana1-3)Manb1-4GlcNAcb1-4GlcNAcb-Sp19	29	3	9
455	Neu5Aca2-3Galb1-4GlcNAcb1-2Mana1-6(GlcNAcb1-4)(Neu5Aca2-3Galb1-4GlcNAcb1-2Mana1-3)Manb1-4GlcNAcb1-4GlcNAcb-Sp21	30	21	71
456	Neu5Aca2-3Galb1-4GlcNAcb1-4Mana1-6(GlcNAcb1-4)(Neu5Aca2-3Galb1-4GlcNAcb1-4(Neu5Aca2-3Galb1-4GlcNAcb1-2)Mana1-3)Manb1-4GlcNAcb1-4GlcNAcb-Sp21	13	3	20
457	Neu5Aca2-3Galb1-4GlcNAcb1-6(Neu5Aca2-3Galb1-4GlcNAcb1-2)Mana1-6(GlcNAcb1-4)(Neu5Aca2-3Galb1-4GlcNAcb1-2Mana1-3)Manb1-4GlcNAcb1-4GlcNAcb-Sp21	34	21	61
458	Neu5Aca2-3Galb1-4GlcNAcb1-6(Neu5Aca2-3Galb1-4GlcNAcb1-2)Mana1-6(GlcNAcb1-4)(Neu5Aca2-3Galb1-4GlcNAcb1-4(Neu5Aca2-3Galb1-4GlcNAcb1-2)Mana1-3)Manb1-4GlcNAcb1-4GlcNAcb-Sp21	11	1	11
459	Neu5Aca2-6Galb1-4GlcNAcb1-2Mana1-6(GlcNAcb1-4)(Neu5Aca2-6Galb1-4GlcNAcb1-2Mana1-3)Manb1-4GlcNAcb1-4GlcNAcb-Sp21	17	1	5
460	Neu5Aca2-6Galb1-4GlcNAcb1-4Mana1-6(GlcNAcb1-4)(Neu5Aca2-6Galb1-4GlcNAcb1-4(Neu5Aca2-6Galb1-4GlcNAcb1-2)Mana1-3)Manb1-4GlcNAcb1-4GlcNAcb-Sp21	17	1	3
461	Neu5Aca2-6Galb1-4GlcNAcb1-6(Neu5Aca2-6Galb1-4GlcNAcb1-2)Mana1-6(GlcNAcb1-4)(Neu5Aca2-6Galb1-4GlcNAcb1-2Mana1-3)Manb1-4GlcNAcb1-4GlcNAcb-Sp21	25	4	15
462	Neu5Aca2-6Galb1-4GlcNAcb1-6(Neu5Aca2-6Galb1-4GlcNAcb1-2)Mana1-6(GlcNAcb1-4)(Neu5Aca2-6Galb1-4GlcNAcb1-4(Neu5Aca2-6Galb1-4GlcNAcb1-2)Mana1-3)Manb1-4GlcNAcb1-4GlcNAcb-Sp21	16	2	14
463	Gala1-3(Fuca1-2)Galb1-3GalNAca-Sp8	26	8	30
464	Gala1-3(Fuca1-2)Galb1-3GalNAcb-Sp8	44	9	21
465	Glca1-6Glca1-6Glca1-6Glcb-Sp10	19	1	5

56	Neu5Aca2-6Galb1-4GlcNAcb1-2Mana1-6(Neu5Aca2-6Galb1-4GlcNAcb1-2Man-a1-3)Manb1-4GlcNAcb1-4GlcNAcb-Sp21	25	1	5
538	GlcNAcb1-3Galb1-4GlcNAcb1-3Galb1-4GlcNAcb1-2Mana1-6(GlcNAcb1-3Galb1-4GlcNAcb1-3Galb1-4GlcNAcb1-2Mana1-3)Manb1-4GlcNAcb1-4GlcNAcb-Sp25	25	2	7
351	Galb1-3GlcNAcb1-2Mana1-6(Galb1-3GlcNAcb1-2Mana1-3)Manb1-4GlcNAcb1-4(Fuca1-6)GlcNAcb-Sp22	24	1	4
379	Galb1-4(Fuca1-3)GlcNAcb1-6(Galb1-3GlcNAcb1-3)Galb1-4Glc-Sp21	24	4	16
388	GalNAcb1-4(Neu5Aca2-3)Galb1-4GlcNAcb1-3GalNAca-Sp14	24	4	17
209	Mana1-2Mana1-3Mana-Sp9	24	3	12
220	Fuca1-2(6S)Galb1-4GlcNAcb-Sp0	24	1	3
227	Neu5Aca2-8Neu5Aca2-8Neu5Aca2-3Galb1-4Glcb-Sp0	24	4	16
557	Galb1-3GlcNAcb1-6(Galb1-3)GalNAc-Sp14	24	1	5
565	Galb1-4GlcNAcb1-3Galb1-4GlcNAcb1-2Mana1-6(Galb1-4GlcNAcb1-3Galb1-4GlcNAcb1-2Mana1-3)Manb1-4GlcNAcb1-4(Fuca1-6)GlcNAcb-Sp24	24	1	3
585	GlcNAcb1-3Galb1-4GlcNAcb1-6(GlcNAcb1-3Galb1-4GlcNAcb1-3)GalNAca-Sp14	24	1	6
247	Fuca1-2(6S)Galb1-4Glcb-Sp0	24	4	16
284	Neu5Gca2-6Galb1-4GlcNAcb-Sp0	24	5	19
566	GlcNAcb1-3Galb1-4GlcNAcb1-3Galb1-4GlcNAcb1-2Mana1-6(GlcNAcb1-3Galb1-4GlcNAcb1-3Galb1-4GlcNAcb1-2Mana1-3)Manb1-4GlcNAcb1-4(Fuca1-6)GlcNAcb-Sp24	24	2	6
567	Galb1-4GlcNAcb1-3Galb1-4GlcNAcb1-3Galb1-4GlcNAcb1-2Mana1-6(Galb1-4GlcNAcb1-3Galb1-4GlcNAcb1-3Galb1-4GlcNAcb1-2Mana1-3)Manb1-4GlcNAcb1-4(Fuca1-6)GlcNAcb-Sp24	24	4	15
10	Neu5Aca-Sp11	24	4	19
81	Fucb1-3GlcNAcb-Sp8	24	5	23
402	Galb1-3GlcNAcb1-6Galb1-4GlcNAcb-Sp0	24	5	20
475	Neu5Aca2-6Galb1-4GlcNAcb1-6GalNAca-Sp14	24	5	23
513	(6P)Galb1-4GlcNAcb-SP0	24	6	27
535	GlcNAcb1-3Galb1-4GlcNAcb1-2Mana1-6(GlcNAcb1-3Galb1-4GlcNAcb1-2Mana1-3)Manb1-4GlcNAcb1-4GlcNAcb-Sp25	24	2	9
145	Galb1-3GalNAcb1-4Galb1-4Glcb-Sp8	23	3	11
246	Neu5Aca2-3Galb1-3GlcNAcb1-3Galb1-4GlcNAcb-Sp0	23	3	13
213	Mana1-6(Mana1-3)Mana-Sp9	23	3	14
235	Neu5Aca2-3GalNAca-Sp8	23	3	14
318	Neu5Aca2-8Neu5Acb-Sp17	23	2	11
517	Gala1-3(Fuca1-2)Galb1-4GlcNAcb1-2Mana-Sp0	23	4	18
191	GlcNAcb1-4GlcNAcb1-4GlcNAcb-Sp8	23	4	19
212	Mana1-2Mana1-6(Mana1-2Mana1-3)Mana1-6(Mana1-2Mana1-2Mana1-3)Manb1-4GlcNAcb1-4GlcNAcb-Sp12	23	5	23
443	Galb1-4(Fuca1-3)GlcNAcb1-6GalNAc-Sp14	23	2	7
90	GalNAca1-3(Fuca1-2)Galb-Sp18	23	5	21
281	Neu5Gca2-3Galb1-4GlcNAcb-Sp0	23	4	16
498	GalNAcb1-4(Fuca1-3)(6S)GlcNAcb-Sp8	23	3	15
555	Galb1-4GlcNAcb1-3Galb1-4GlcNAcb1-3Galb1-4GlcNAcb1-3Galb1-4GlcNAcb1-3Galb1-4GlcNAcb1-2Mana1-6(Galb1-4GlcNAcb1-3Galb1-4GlcNAcb1-3Galb1-4GlcNAcb1-3Galb1-4GlcNAcb1-3Galb1-4GlcNAcb1-2Mana1-3)Manb1-4GlcNAcb1-4GlcNAcb-Sp25	23	1	3
591	Neu5Aca2-6Galb1-4GlcNAcb1-3Galb1-4GlcNAcb1-6(Galb1-3)GalNAca-Sp14	23	5	22
64	Fuca1-2Galb1-3GalNAcb1-4(Neu5Aca2-3)Galb1-4Glcb-Sp9	22	16	73
177	GlcNAcb1-6(GlcNAcb1-3)GalNAca-Sp14	22	3	13
208	Mana1-2Mana1-6(Mana1-2Mana1-3)Mana-Sp9	22	2	8
508	(3S)GalNAcb1-4(3S)GlcNAc-Sp8	22	5	22
53	GlcNAcb1-2Mana1-6(GlcNAcb1-2Mana1-3)Manb1-4GlcNAcb1-4GlcNAcb-Sp13	22	2	10
157	Galb1-4GalNAca1-3(Fuca1-2)Galb1-4GlcNAcb-Sp8	22	1	5
182	GlcNAcb1-3Galb1-4GlcNAcb-Sp0	22	1	6
300	Galb1-4GlcNAcb1-6(Galb1-4GlcNAcb1-3)Galb1-4GlcNAc-Sp0	22	4	19
50	Mana1-6(Mana1-3)Manb1-4GlcNAcb1-4GlcNAcb-Sp12	22	4	19
140	Galb1-3GalNAca-Sp14	22	3	12
168	Galb1-4GlcNAcb-Sp0	22	4	17
316	Neu5Aca2-6Galb1-4GlcNAcb1-2Mana1-6(Neu5Aca2-3Galb1-4GlcNAcb1-2Mana1-3)Manb1-4GlcNAcb1-4GlcNAcb-Sp12	22	2	8
342	Neu5Aca2-6Galb1-4GlcNAcb1-2Mana1-6(Mana1-3)Manb1-4GlcNAcb1-4GlcNAc-Sp12	22	2	8
398	GalNAca1-3GalNAcb1-3Gala1-4Galb1-4GlcNAcb-Sp0	22	2	10
426	Fuca1-3GlcNAcb1-6(Galb1-4GlcNAcb1-3)Galb1-4Glc-Sp21	22	3	15
482	Neu5Aca2-6Galb1-4GlcNAcb1-6(Fuca1-2Galb1-4(Fuca1-3)GlcNAcb1-3)Galb1-4Glc-Sp21	22	5	21
217	Manb1-4GlcNAcb-Sp0	22	1	6
378	Galb1-3GlcNAcb1-3Galb1-4GlcNAcb1-6(Galb1-3GlcNAcb1-3)Galb1-4Glcb-Sp0	22	4	17
478	Neu5Aca2-3Galb1-4GlcNAcb1-2Mana1-6(Neu5Aca2-3Galb1-4GlcNAcb1-2Mana1-3)Manb1-4GlcNAcb1-4(Fuca1-6)GlcNAcb-Sp24	22	2	9
78	Fuca1-2Galb-Sp8	21	11	50
149	Galb1-3GlcNAcb-Sp0	21	2	10
166	Galb1-4GlcNAcb1-6(Galb1-3)GalNAca-Sp8	21	2	7
405	Gala1-3(Fuca1-2)Galb1-4(Fuca1-3)Glcb-Sp21	21	2	10
484	Gala1-3Galb1-3GlcNAcb1-6GalNAca-Sp14	21	3	12
533	Galb1-3GalNAcb1-3Gal-Sp21	21	4	20
57	Neu5Aca2-6Galb1-4GlcNAcb1-2Mana1-6(Neu5Aca2-6Galb1-4GlcNAcb1-2Mana1-3)Manb1-4GlcNAcb1-4GlcNAcb-Sp24	21	2	10
173	GlcNAca1-3Galb1-4GlcNAcb-Sp8	21	1	4
266	Neu5Aca2-6Galb1-4GlcNAcb-Sp0	21	2	10
333	GalNAca1-3(Fuca1-2)Galb1-4GlcNAcb1-3Galb1-4GlcNAcb1-3Galb1-4GlcNAcb-Sp0	21	1	7
363	Neu5Aca2-6GlcNAcb1-4GlcNAc-Sp21	21	4	17
494	Fuca1-2Galb1-4GlcNAcb1-2Mana-Sp0	21	3	14
599	GlcNAcb1-3Fuca-Sp21	21	2	10
59	Fuca1-2Galb1-3GalNAcb1-3Gala1-4Galb1-4Glcb-Sp9	21	3	13
111	Gala1-3GalNAca-Sp16	21	2	10
360	Gala1-3Galb1-4GlcNAcb1-2Mana1-6(Gala1-3Galb1-4GlcNAcb1-2Mana1-3)Manb1-4GlcNAcb1-4GlcNAcb-Sp20	21	1	5
432	Galb1-4GlcNAcb1-2Mana1-6(GlcNAcb1-4)(Galb1-4GlcNAcb1-4(Galb1-4GlcNAcb1-2)Mana1-3)Manb1-4GlcNAcb1-4GlcNAc-Sp21	21	3	15
503	Galb1-4GlcNAcb1-6(Galb1-4GlcNAcb1-2)Mana1-6(GlcNAcb1-4)Galb1-4GlcNAcb1-4(Gal b1-4GlcNAcb1-2)Mana1-3)Manb1-4GlcNAcb1-4(Fuca1-6)GlcNAc-Sp21	21	3	15
518	GalNAca1-3(Fuca1-2)Galb1-4 GlcNAcb1-2Mana-Sp0	21	3	12
553	GalNAcb1-4GlcNAcb1-3GalNAcb1-4GlcNAcb-Sp0	21	7	32
74	Fuca1-2Galb1-4GlcNAcb1-3Galb1-4GlcNAcb1-3Galb1-4GlcNAcb-Sp0	21	4	18
190	GlcNAcb1-4GlcNAcb1-4GlcNAcb1-4GlcNAcb1-4GlcNAcb1-Sp8	21	2	8
446	Gala1-3(Fuca1-2)Galb1-4GlcNAcb1-6(Gala1-3(Fuca1-2)Galb1-4GlcNAcb1-3)GalNAc-Sp14	21	4	18
161	Galb1-4GlcNAcb1-3Galb1-4(Fuca1-3)GlcNAcb1-3Galb1-4(Fuca1-3)GlcNAcb-Sp0	20	3	17
170	Galb1-4GlcNAcb-Sp23	20	4	19
186	GlcNAcb1-4-MDPLys	20	1	5
250	Neu5Aca2-3Galb1-4(Fuca1-3)(6S)GlcNAcb-Sp8	20	2	8
596	Neu5Aca2-6Galb1-4GlcNAcb1-3Galb1-4GlcNAcb1-3Galb1-4GlcNAcb1-2Mana1-6(Neu5Aca2-6Galb1-4GlcNAcb1-3Galb1-4GlcNAcb1-3Galb1-4GlcNAcb1-2Mana1-3)Manb1-4GlcNAcb1-4GlcNAcb-Sp12	20	3	14
159	Galb1-4GlcNAcb1-3GalNAca-Sp8	20	4	19
181	GlcNAcb1-3Galb-Sp8	20	3	15
216	Mana1-6(Mana1-3)Mana1-6(Mana1-3)Manb1-4GlcNAcb1-4GlcNAcb-Sp12	20	1	4
223	Neu5Aca2-3Galb1-3GalNAca-Sp8	20	2	9
413	Gala1-3(Fuca1-2)Galb1-4(Fuca1-3)GlcNAcb1-3GalNAc-Sp14	20	7	36
487	(3S)Galb1-3(Fuca1-4)GlcNAcb-Sp0	20	4	18
554	GlcNAcb1-3Galb1-4GlcNAcb1-3Galb1-4GlcNAcb1-3Galb1-4GlcNAcb1-3Galb1-4GlcNAcb1-2Mana1-6(GlcNAcb1-3Galb1-4GlcNAcb1-3Galb1-4GlcNAcb1-3Galb1-4GlcNAcb1-3Galb1-4GlcNAcb1-2Mana1-3)Manb1-4GlcNAcb1-4GlcNAcb-Sp25	20	1	4
73	Fuca1-2Galb1-4GlcNAcb1-3Galb1-4GlcNAcb-Sp0	20	3	13
154	Galb1-4(Fuca1-3)GlcNAcb1-3Galb1-4(Fuca1-3)GlcNAcb1-3Galb1-4(Fuca1-3)GlcNAcb-Sp0	20	1	3
172	Galb1-4Glcb-Sp8	20	6	31
231	GalNAcb1-4(Neu5Aca2-3)Galb1-4GlcNAcb-Sp8	20	1	3
343	Mana1-6(Neu5Aca2-6Galb1-4GlcNAcb1-2Mana1-3)Manb1-4GlcNAcb1-4GlcNAc-Sp12	20	2	8
433	Galb1-4GlcNAcb1-6(Galb1-4GlcNAcb1-2)Mana1-6(GlcNAcb1-4)(Galb1-4GlcNAcb1-2Mana1-3)Manb1-4GlcNAcb1-4GlcNAc-Sp21	20	10	52
520	Gala1-3(Fuca1-2)Galb1-3GlcNAcb1-6GalNAc-Sp14	20	2	9
541	Galb1-3GlcNAcb1-3Galb1-4GlcNAcb1-2Mana1-6(Galb1-3GlcNAcb1-3Galb1-4GlcNAcb1-2Mana1-3)Manb1-4GlcNAcb1-4GlcNAc-Sp25	20	2	8
86	GalNAca1-3(Fuca1-2)Galb1-4GlcNAcb-Sp8	20	3	13
152	Galb1-4(Fuca1-3)GlcNAcb-Sp8	20	4	21
273	Neu5Aca2-8Neu5Aca-Sp8	20	2	10
301	GlcNAcb1-6(Galb1-4GlcNAcb1-3)Galb1-4GlcNAc-Sp0	20	2	10
372	Neu5Aca2-3Galb1-4GlcNAcb1-3GalNAc-Sp14	20	1	3
65	Fuca1-2Galb1-3GlcNAcb1-3Galb1-4Glcb-Sp8	19	43	226
276	Neu5Acb2-6GalNAca-Sp8	19	6	30
303	Galb1-4GlcNAcb1-6Galb1-4GlcNAcb-Sp0	19	5	23
345	Neu5Aca2-6Galb1-4GlcNAcb1-2Mana1-3Manb1-4GlcNAcb1-4GlcNAc-Sp12	19	2	9
384	GlcNAcb1-2Mana1-6(GlcNAcb1-4(GlcNAcb1-2)Mana1-3)Manb1-4GlcNAcb1-4GlcNAc-Sp21	19	4	21
425	Galb1-4GlcNAcb1-6(Fuca1-2Galb1-3GlcNAcb1-3)Galb1-4Glc-Sp21	19	2	10
465	Glca1-6Glca1-6Glca1-6Glcb-Sp10	19	1	5
51	Mana1-6(Mana1-3)Manb1-4GlcNAcb1-4GlcNAcb-Sp13	19	4	19

466	Glca1-4Glca1-4Glca1-4Glcb-Sp10	27	4	16
467	Neu5Aca2-3Galb1-4GlcNAcb1-6(Neu5Aca2-3Galb1-4GlcNAcb1-3)GalNAca-Sp14	15	2	12
468	Fuca1-2Galb1-4(Fuca1-3)GlcNAcb1-2Mana1-6(Fuca1-2Galb1-4(Fuca1-3)GlcNAcb1-2Mana1-3)Manb1-4GlcNAcb1-4(Fuca1-6)GlcNAcb-Sp24	138	78	57
469	Fuca1-2Galb1-3(Fuca1-4)GlcNAcb1-2Mana1-6(Fuca1-2Galb1-3(Fuca1-4)GlcNAcb1-2Mana1-3)Manb1-4GlcNAcb1-4(Fuca1-6)GlcNAcb1-4(Fuca1-6)GlcNAcb-Sp19	41	2	4
470	GlcNAcb1-6(GlcNAcb1-2)Mana1-6(GlcNAcb1-2Mana1-3)Manb1-4GlcNAcb1-4(Fuca1-6)GlcNAcb-Sp24	32	4	12
471	Galb1-3GlcNAcb1-2Mana1-6(GlcNAcb1-4)(Galb1-3GlcNAcb1-2Mana1-3)Manb1-4GlcNAcb1-4GlcNAcb-Sp21	39	5	11
472	Neu5Aca2-6Galb1-4GlcNAcb1-6(Galb1-3GlcNAcb1-3)Galb1-4Glcb-Sp21	32	5	14
473	Neu5Aca2-3Galb1-4GlcNAcb1-2Mana-Sp0	29	9	31
474	Neu5Aca2-3Galb1-4GlcNAcb1-6GalNAca-Sp14	14	2	13
475	Neu5Aca2-6Galb1-4GlcNAcb1-6GalNAca-Sp14	24	5	23
476	Neu5Aca2-6Galb1-4 GlcNAcb1-6(Neu5Aca2-6Galb1-4GlcNAcb1-3)GalNAca-Sp14	17	2	11
477	Neu5Aca2-6Galb1-4GlcNAcb1-2Mana1-6(Neu5Aca2-6Galb1-4GlcNAcb1-2Mana1-3)Manb1-4GlcNAcb1-4(Fuca1-6)GlcNAcb-Sp24	34	2	5
478	Neu5Aca2-3Galb1-4GlcNAcb1-2Mana1-6(Neu5Aca2-3Galb1-4GlcNAcb1-2Mana1-3)Manb1-4GlcNAcb1-4(Fuca1-6)GlcNAcb-Sp24	22	2	9
479	Mana1-6(Mana1-3)Manb1-4GlcNAcb1-4(Fuca1-6)GlcNAcb-Sp19	41	8	19
480	Galb1-4GlcNAcb1-6(Galb1-4GlcNAcb1-2)Mana1-6(Galb1-4GlcNAcb1-2Mana1-3)Manb1-4GlcNAcb1-4(Fuca1-6)GlcNAcb-Sp24	37	5	13
481	Neu5Aca2-3Galb1-3GlcNAcb1-2Mana1-6(GlcNAcb1-4)(Neu5Aca2-3Galb1-3GlcNAcb1-2Mana1-3)Manb1-4GlcNAcb1-4GlcNAc-Sp21	43	11	25
482	Neu5Aca2-6Galb1-4GlcNAcb1-6(Fuca1-2Galb1-4(Fuca1-3)GlcNAcb1-3)Galb1-4Glc-Sp21	22	5	21
483	Galb1-3GlcNAcb1-6GalNAca-Sp14	91	25	28
484	Gala1-3Galb1-3GlcNAcb1-6GalNAca-Sp14	21	3	12
485	Galb1-3(Fuca1-4)GlcNAcb1-6GalNAca-Sp14	27	8	29
486	Neu5Aca2-3Galb1-3GlcNAcb1-6GalNAca-Sp14	29	5	18
487	(3S)Galb1-3(Fuca1-4)GlcNAcb-Sp0	20	4	18
488	Galb1-4(Fuca1-3)GlcNAcb1-6(Neu5Aca2-6(Neu5Aca2-3Galb1-3)GlcNAcb1-3)Galb1-4Glc-Sp21	29	2	5
489	Fuca1-2Galb1-4GlcNAcb1-6GalNAca-Sp14	17	3	17
490	Gala1-3Galb1-4GlcNAcb1-6GalNAca-Sp14	25	8	31
491	Galb1-4(Fuca1-3)GlcNAcb1-2Mana-Sp0	30	3	10
492	Fuca1-2(6S)Galb1-3GlcNAcb-Sp0	18	4	24
493	Gala1-3(Fuca1-2)Galb1-4GlcNAcb1-6GalNAca-Sp14	2	0	0
494	Fuca1-2Galb1-4GlcNAcb1-2Mana-Sp0	21	3	14
495	Fuca1-2Galb1-3(6S)GlcNAcb-Sp0	34	5	14
496	Fuca1-2(6S)Galb1-3(6S)GlcNAcb-Sp0	25	2	6
497	Neu5Aca2-6GalNAcb1-4(6S)GlcNAcb-Sp8	8	5	67
498	GalNAcb1-4(Fuca1-3)(6S)GlcNAcb-Sp8	23	3	15
499	(3S)GalNAcb1-4(Fuca1-3)GlcNAcb-Sp8	19	4	24
500	Fuca1-2Galb1-3GlcNAcb1-6(Fuca1-2Galb1-3GlcNAcb1-3)GalNAca-Sp14	32	3	8
501	GalNAca1-3(Fuca1-2)Galb1-3GlcNAcb1-6GalNAca-Sp14	16	2	11
502	GlcNAcb1-6(GlcNAcb1-2)Mana1-6(GlcNAcb1-4)(GlcNAcb1-4(GlcNAcb1-2)Mana1-3)Manb1-4GlcNAcb1-4(Fuca1-6)GlcNAc-Sp21	17	2	14
503	Galb1-4GlcNAcb1-6(Galb1-4GlcNAcb1-2)Mana1-6(GlcNAcb1-4)Galb1-4GlcNAcb1-4(Gal b1-4GlcNAcb1-2)Mana1-3)Manb1-4GlcNAcb1-4(Fuca1-6)GlcNAc-Sp21	21	3	15
504	Galb1-3GlcNAca1-3Galb1-4GlcNAcb-Sp8	29	11	38
505	Galb1-3(6S)GlcNAcb-Sp8	12	6	52
506	(6S)(4S)GalNAcb1-4GlcNAc-Sp8	118	118	100
507	(6S)GalNAcb1-4GlcNAc-Sp8	156	5	3
508	(3S)GalNAcb1-4(3S)GlcNAc-Sp8	22	5	22
509	GalNAcb1-4(6S)GlcNAc-Sp8	32	20	61
510	(3S)GalNAcb1-4GlcNAc-Sp8	16	1	8
511	(4S)GalNAcb-Sp10	25	2	6
512	Galb1-4(6P)GlcNAcb-Sp0	18	3	18
513	(6P)Galb1-4GlcNAcb-SP0	24	6	27
514	GalNAca1-3(Fuca1-2)Galb1-4GlcNAcb1-6GalNAc-Sp14	32	1	4
515	Neu5Aca2-6Galb1-4GlcNAcb1-2Man-Sp0	41	13	31
516	Gala1-3Galb1-4GlcNAcb1-2Mana-Sp0	17	2	14
517	Gala1-3(Fuca1-2)Galb1-4GlcNAcb1-2Mana-Sp0	23	4	18
518	GalNAca1-3(Fuca1-2)Galb1-4 GlcNAcb1-2Mana-Sp0	21	3	12
519	Galb1-3GlcNAcb1-2Mana-Sp0	32	4	11
520	Gala1-3(Fuca1-2)Galb1-3GlcNAcb1-6GalNAc-Sp14	20	2	9
521	Neu5Aca2-3Galb1-3GlcNAcb1-2Mana-Sp0	18	4	21
522	Gala1-3Galb1-3GlcNAcb1-2Mana-Sp0	15	3	24
523	GalNAcb1-4GlcNAcb1-2Mana-Sp0	29	9	30
524	Neu5Aca2-3Galb1-3GalNAcb1-4Galb1-4Glcb-Sp0	10	5	48
525	GlcNAcb1-2 Mana1-6(GlcNAcb1-4)(GlcNAcb1-2Mana1-3)Manb1-4GlcNAcb1-4(Fuca1-6)GlcNAc-Sp21	14	1	6
526	Galb1-4GlcNAcb1-2 Mana1-6(GlcNAcb1-4)(Galb1-4GlcNAcb1-2Mana1-3)Manb1-4GlcNAcb1-4(Fuca1-6)GlcNAc-Sp21	16	1	9
527	Galb1-4GlcNAcb1-2 Mana1-6(Galb1-4GlcNAcb1-4)(Galb1-4GlcNAcb1-2Mana1-3)Manb1-4GlcNAcb1-4(Fuca1-6)GlcNAc-Sp21	15	2	14
528	Fuca1-4(Galb1-3)GlcNAcb1-2 Mana-Sp0	41	13	32
529	Neu5Aca2-3Galb1-4(Fuca1-3)GlcNAcb1-2Mana-Sp0	18	2	10
530	GlcNAcb1-3Galb1-4GlcNAcb1-6(GlcNAcb1-3)Galb1-4GlcNAc-Sp0	12	1	11
531	GalNAca1-3(Fuca1-2)Galb1-3GalNAcb1-3Gala1-4Galb1-4Glc-Sp21	25	4	16
532	Gala1-3(Fuca1-2)Galb1-3GalNAcb1-3Gala1-4Galb1-4Glc-Sp21	17	2	13
533	Galb1-3GalNAcb1-3Gal-Sp21	21	4	20
534	GlcNAcb1-3Galb1-4GlcNAcb1-2Mana1-6(GlcNAcb1-3Galb1-4GlcNAcb1-2Mana1-3)Manb1-4GlcNAcb1-4GlcNAcb-Sp12	64	15	24
535	GlcNAcb1-3Galb1-4GlcNAcb1-2Mana1-6(GlcNAcb1-3Galb1-4GlcNAcb1-2Mana1-3)Manb1-4GlcNAcb1-4GlcNAcb-Sp25	24	2	9
536	Fuca1-2Galb1-4GlcNAcb1-3Galb1-4GlcNAcb1-2Mana1-6(Fuca1-2Galb1-4GlcNAcb1-3Galb1-4GlcNAcb1-2Mana1-3)Manb1-4GlcNAcb1-4GlcNAcb-Sp24	40	2	6
537	GlcNAcb1-3Galb1-4GlcNAcb1-3Galb1-4GlcNAcb1-2Mana1-6(GlcNAcb1-3Galb1-4GlcNAcb1-3Galb1-4GlcNAcb1-2Mana1-3)Manb1-4GlcNAcb1-4GlcNAcb-Sp12	30	4	12
538	GlcNAcb1-3Galb1-4GlcNAcb1-3Galb1-4GlcNAcb1-2Mana1-6(GlcNAcb1-3Galb1-4GlcNAcb1-3Galb1-4GlcNAcb1-2Mana1-3)Manb1-4GlcNAcb1-4GlcNAcb-Sp25	25	2	7
539	Galb1-4GlcNAcb1-3Galb1-4GlcNAcb1-3Galb1-4GlcNAcb1-2Mana1-6(Galb1-4GlcNAcb1-3Galb1-4GlcNAcb1-3Galb1-4GlcNAcb1-2Mana1-3)Manb1-4GlcNAcb1-4GlcNAcb-Sp12	33	2	7
540	Galb1-4GlcNAcb1-3Galb1-4GlcNAcb1-3Galb1-4GlcNAcb1-2Mana1-6(Galb1-4GlcNAcb1-3Galb1-4GlcNAcb1-3Galb1-4GlcNAcb1-2Mana1-3)Manb1-4GlcNAcb1-4GlcNAcb-Sp24	19	4	21
541	Galb1-3GlcNAcb1-3Galb1-4GlcNAcb1-2Mana1-6(Galb1-3GlcNAcb1-3Galb1-4GlcNAcb1-2Mana1-3)Manb1-4GlcNAcb1-4GlcNAc-Sp25	20	2	8
542	Neu5Gca2-8Neu5Gca2-3Galb1-4GlcNAc-Sp0	14	2	14
543	Neu5Aca2-8Neu5Gca2-3Galb1-4GlcNAc-Sp0	17	2	14
544	Neu5Gca2-8Neu5Aca2-3Galb1-4GlcNAc-Sp0	15	3	17
545	Neu5Gca2-8Neu5Gca2-3Galb1-4GlcNAcb1-3Galb1-4GlcNAc-Sp0	17	1	8
546	Neu5Gca2-8Neu5Gca2-6Galb1-4GlcNAc-Sp0	36	16	44
547	Neu5Aca2-8Neu5Aca2-3Galb1-4GlcNAc-Sp0	13	2	17
548	GlcNAcb1-3Galb1-4GlcNAcb1-6(GlcNAcb1-3Galb1-4GlcNAcb1-2)Mana1-6(GlcNAcb1-3Galb1-4GlcNAcb1-2Man a1-3)Manb1-4GlcNAcb1-4GlcNAc-Sp24	38	3	7
549	Galb1-4GlcNAcb1-3Galb1-4GlcNAcb1-6(Galb1-4GlcNAcb1-3Galb1-4GlcNAcb1-2)Mana1-6(Galb1-4GlcNAcb1-3Galb1-4GlcNAcb1-2Mana1-3)Mana1-4GlcNAcb1-4GlcNAc-Sp24	30	6	20
550	Gala1-3Galb1-4GlcNAcb1-2Mana1-6(Gala1-3Galb1-4GlcNAcb1-2Mana1-3)Manb1-4GlcNAcb1-4GlcNAc-Sp24	26	2	8
551	GlcNAcb1-3Galb1-4GlcNAcb1-6(GlcNAcb1-3Galb1-3)GalNAca-Sp14	12	2	13
552	GalNAcb1-3GlcNAcb-Sp0	17	1	6
553	GalNAcb1-4GlcNAcb1-3GalNAcb1-4GlcNAcb-Sp0	21	7	32
554	GlcNAcb1-3Galb1-4GlcNAcb1-3Galb1-4GlcNAcb1-3Galb1-4GlcNAcb1-3Galb1-4GlcNAcb1-2Mana1-6(GlcNAcb1-3Galb1-4GlcNAcb1-3Galb1-4GlcNAcb1-3Galb1-4GlcNAcb1-3Galb1-4GlcNAcb1-2Mana1-3)Manb1-4GlcNAcb1-4GlcNAcb-Sp25	20	1	4
555	Galb1-4GlcNAcb1-3Galb1-4GlcNAcb1-3Galb1-4GlcNAcb1-3Galb1-4GlcNAcb1-3Galb1-4GlcNAcb1-2Mana1-6(Galb1-4GlcNAcb1-3Galb1-4GlcNAcb1-3Galb1-4GlcNAcb1-3Galb1-4GlcNAcb1-3Galb1-4GlcNAcb1-2Mana1-3)Manb1-4GlcNAcb1-4GlcNAcb-Sp25	23	1	3
556	GlcNAb1-3Galb1-3GalNAc-Sp14	19	5	24
557	Galb1-3GlcNAcb1-6(Galb1-3)GalNAc-Sp14	24	1	5
558	Galb1-4GlcNAcb1-3Galb1-4GlcNAcb1-3Galb1-4GlcNAcb1-3Galb1-4GlcNAcb1-3Galb1-4GlcNAcb1-3Galb1-4GlcNAcb1-2Mana1-6(Galb1-4GlcNAcb1-3Galb1-4GlcNAcb1-3Galb1-4GlcNAcb1-3Galb1-4GlcNAcb1-3Galb1-4GlcNAcb1-3Galb1-4GlcNAcb1-2Mana1-3)Manb1-4GlcNAcb1-4GlcNAcb-Sp25	30	7	24
559	(3S)GlcAb1-3Galb1-4GlcNAcb1-3Galb1-4Glc-Sp0	15	2	14
560	(3S)GlcAb1-3Galb1-4GlcNAcb1-2Mana-Sp0	36	2	6
561	Galb1-3GlcNAcb1-3Galb1-4GlcNAcb1-3Galb1-4GlcNAcb1-6(Galb1-3GlcNAcb1-3Galb1-4GlcNAcb1-3Galb1-4GlcNAb1-2)Mana1-6(Galb1-3GlcNAcb1-3Galb1-4GlcNAcb1-3Galb1-4GlcNAcb1-2Mana1-3)Manb1-4GlcNAcb1-4(Fuca1-6)GlcNAcb-Sp24	30	4	14
562	Galb1-3GlcNAcb1-3Galb1-4GlcNAcb1-6(Galb1-3GlcNAcb1-3Galb1-4GlcNAb1-2)Mana1-6(Galb1-3GlcNAcb1-3Galb1-4GlcNAcb1-2Mana1-3)Manb1-4GlcNAcb1-4(Fuca1-6)GlcNAcb-Sp24	30	6	21
563	Neu5Aca2-8Neu5Aca2-3Galb1-3GalNAcb1-4(Neu5Aca2-3)Galb1-4Glc-Sp21	17	4	22
564	GlcNAcb1-3Galb1-4GlcNAcb1-2Mana1-6(GlcNAcb1-3Galb1-4GlcNAcb1-2Mana1-3)Manb1-4GlcNAcb1-4(Fuca1-6)GlcNAcb-Sp24	32	6	19
565	Galb1-4GlcNAcb1-3Galb1-4GlcNAcb1-2Mana1-6(Galb1-4GlcNAcb1-3Galb1-4GlcNAcb1-2Mana1-3)Manb1-4GlcNAcb1-4(Fuca1-6)GlcNAcb-Sp24	24	1	3
566	GlcNAcb1-3Galb1-4GlcNAcb1-3Galb1-4GlcNAcb1-2Mana1-6(GlcNAcb1-3Galb1-4GlcNAcb1-3Galb1-4GlcNAcb1-2Mana1-3)Manb1-4GlcNAcb1-4(Fuca1-6)GlcNAcb-Sp24	24	2	6
567	Galb1-4GlcNAcb1-3Galb1-4GlcNAcb1-3Galb1-4GlcNAcb1-2Mana1-6(Galb1-4GlcNAcb1-3Galb1-4GlcNAcb1-3Galb1-4GlcNAcb1-2Mana1-3)Manb1-4GlcNAcb1-4(Fuca1-6)GlcNAcb-Sp24	24	4	15
568	GlcNAcb1-3Galb1-4GlcNAcb1-3Galb1-4GlcNAcb1-3Galb1-4GlcNAcb1-2Mana1-6(GlcNAcb1-3Galb1-4GlcNAcb1-3Galb1-4GlcNAcb1-3Galb1-4GlcNAcb1-2Mana1-3)Manb1-4GlcNAcb1-4(Fuca1-6)GlcNAcb-Sp24	40	7	17
569	Galb1-4GlcNAcb1-3Galb1-4GlcNAcb1-3Galb1-4GlcNAcb1-3Galb1-4GlcNAcb1-2Mana1-6(Galb1-4GlcNAcb1-3Galb1-4GlcNAcb1-3Galb1-4GlcNAcb1-3Galb1-4GlcNAcb1-2Mana1-3)Manb1-4GlcNAcb1-4(Fuca1-6)GlcNAcb-Sp24	30	2	5
570	GlcNAcb1-3Galb1-4GlcNAcb1-3Galb1-4GlcNAcb1-3Galb1-4GlcNAcb1-3Galb1-4GlcNAcb1-2Mana1-6(GlcNAcb1-3Galb1-4GlcNAcb1-3Galb1-4GlcNAcb1-3Galb1-4GlcNAcb1-3Galb1-4GlcNAcb1-2Mana1-3)Manb1-4GlcNAcb1-4(Fuca1-6)GlcNAcb-Sp19	30	2	6

269	Neu5Aca2-6Galb1-4GlcNAcb1-3Galb1-4GlcNAcb-Sp0	19	1	6
399	Gala1-4Galb1-3GlcNAcb1-2Mana1-6(Gala1-4Galb1-3GlcNAcb1-2Mana1-3)Manb1-4GlcNAcb1-4GlcNAcb-Sp19	19	3	15
429	GlcNAcb1-6(GlcNAcb1-2)Mana1-6(GlcNAcb1-4)(GlcNAcb1-2Mana1-3)Manb1-4GlcNAcb1-4GlcNAc-Sp21	19	3	15
556	GlcNAb1-3Galb1-3GalNAc-Sp14	19	5	24
41	(6P)Mana-Sp8	19	13	69
92	GalNAca1-3Galb-Sp8	19	2	11
214	Mana1-2Mana1-2Mana1-6(Mana1-3)Mana-Sp9	19	2	13
242	Neu5Aca2-6(Neu5Aca2-3Galb1-3)GalNAca-Sp8	19	2	8
346	Galb1-4GlcNAcb1-2Mana1-3Manb1-4GlcNAcb1-4GlcNAc-Sp12	19	2	9
395	Neu5Aca2-3Galb1-3GlcNAcb1-3GalNAca-Sp14	19	2	11
437	Neu5Aca2-3Galb1-4GlcNAcb1-3Galb-Sp8	19	2	13
540	Galb1-4GlcNAcb1-3Galb1-4GlcNAcb1-3Galb1-4GlcNAcb1-2Mana1-6(Galb1-4GlcNAcb1-3Galb1-4GlcNAcb1-3Galb1-4GlcNAcb1-2Mana1-3)Manb1-4GlcNAcb1-4GlcNAcb-Sp24	19	4	21
240	Neu5Aca2-3Galb1-4(Neu5Aca2-3Galb1-3)GlcNAcb-Sp8	19	4	22
267	Neu5Aca2-6Galb1-4GlcNAcb-Sp8	19	1	7
401	Gala1-3Galb1-4GlcNAcb1-3GalNAca-Sp14	19	1	7
436	Galb1-6Galb-Sp10	19	3	16
499	(3S)GalNAcb1-4(Fuca1-3)GlcNAcb-Sp8	19	4	24
75	Fuca1-2Galb1-4GlcNAcb-Sp0	18	2	8
364	Neu5Aca2-6GlcNAcb1-4GlcNAcb1-4GlcNAc-Sp21	18	2	9
430	GlcNAcb1-6(GlcNAcb1-2)Mana1-6(GlcNAcb1-4)(GlcNAcb1-4(GlcNAcb1-2)Mana1-3)Manb1-4GlcNAcb1-4GlcNAc-Sp21	18	4	23
322	Neu5Aca2-3Galb1-4GlcNAcb1-2Mana1-6(Neu5Aca2-3Galb1-4GlcNAcb1-2Mana1-3)Manb1-4GlcNAcb1-4GlcNAcb-Sp12	18	1	8
492	Fuca1-2(6S)Galb1-3GlcNAcb-Sp0	18	4	24
262	Neu5Aca2-3Galb1-4Glcb-Sp8	18	2	11
264	Neu5Aca2-6GalNAcb1-4GlcNAcb-Sp0	18	1	3
353	KDNa2-3Galb1-4(Fuca1-3)GlcNAc-Sp0	18	4	23
411	GalNAca1-3GalNAcb1-3Gala1-4Galb1-4Glcb-Sp0	18	7	39
521	Neu5Aca2-3Galb1-3GlcNAcb1-2Mana-Sp0	18	4	21
529	Neu5Aca2-3Galb1-4(Fuca1-3)GlcNAcb1-2Mana-Sp0	18	2	10
180	GlcNAcb1-3GalNAca-Sp14	18	5	29
229	Neu5Aca2-8Neu5Aca2-8Neu5Aca-Sp8	18	2	11
313	Mana1-2Mana1-6(Mana1-3)Mana1-6(Mana1-2Mana1-2Mana1-3)Mana-Sp9	18	14	78
325	Neu5,9Ac2a2-3Galb1-4GlcNAcb-Sp0	18	6	36
409	Gala1-3(Fuca1-2)Galb1-4GlcNAcb1-3GalNAca-Sp14	18	5	27
512	Galb1-4(6P)GlcNAcb-Sp0	18	3	18
589	Galb1-4GlcNAcb1-3Galb1-3GalNAca-Sp14	18	2	10
136	Neu5Aca2-6(Galb1-3)GalNAca-Sp14	17	3	14
147	Galb1-3GlcNAcb1-3Galb1-4GlcNAcb-Sp0	17	1	6
207	Mana1-2Mana1-2Mana1-3Mana-Sp9	17	2	10
312	Mana1-6(Mana1-3)Mana1-6(Mana1-3)Manb-Sp10	17	2	10
438	GalNAcb1-6GalNAcb-Sp8	17	2	9
476	Neu5Aca2-6Galb1-4 GlcNAcb1-6(Neu5Aca2-6Galb1-4GlcNAcb1-3)GalNAca-Sp14	17	2	11
552	GalNAcb1-3GlcNAcb-Sp0	17	1	6
587	Neu5Aca2-6Galb1-4GlcNAcb1-3Galb1-4GlcNAcb1-3GalNAca-Sp14	17	2	11
134	GlcNAcb1-6(Galb1-3)GalNAca-Sp14	17	1	5
193	GlcNAcb1-6GalNAca-Sp14	17	3	17
236	Neu5Aca2-3GalNAcb1-4GlcNAcb-Sp0	17	4	25
271	Neu5Aca2-6Galb1-4Glcb-Sp8	17	5	29
319	Neu5Aca2-8Neu5Aca2-8Neu5Acb-Sp8	17	5	29
326	Neu5,9Ac2a2-3Galb1-3GlcNAcb-Sp0	17	4	22
383	Galb1-4GlcNAcb1-6(Galb1-4GlcNAcb1-2)Mana1-6(Galb1-4GlcNAcb1-4(Galb1-4GlcNAcb1-2)Mana1-3)Manb1-4GlcNAcb1-4GlcNAcb-Sp21	17	1	5
459	Neu5Aca2-6Galb1-4GlcNAcb1-2Mana1-6(GlcNAcb1-4)(Neu5Aca2-6Galb1-4GlcNAcb1-2Mana1-3)Manb1-4GlcNAcb1-4GlcNAcb-Sp21	17	1	5
543	Neu5Aca2-8Neu5Gca2-3Galb1-4GlcNAc-Sp0	17	2	14
563	Neu5Aca2-8Neu5Aca2-3Galb1-3GalNAcb1-4(Neu5Aca2-3)Galb1-4Glc-Sp21	17	4	22
590	Neu5Aca2-3Galb1-4GlcNAcb1-3Galb1-4GlcNAcb1-6(Galb1-3)GalNAca-Sp14	17	2	14
175	GlcNAcb1-2Galb1-3GalNAca-Sp8	17	4	22
176	GlcNAcb1-6(GlcNAcb1-3)GalNAca-Sp8	17	2	12
253	Neu5Aca2-3Galb1-4(Fuca1-3)GlcNAcb-Sp8	17	3	15
382	Galb1-3GlcNAcb1-3Galb1-4(Fuca1-3)GlcNAcb1-6(Galb1-3GlcNAcb1-3)Galb1-4Glc-Sp21	17	2	10
387	Galb1-3GlcNAcb1-3GalNAca-Sp14	17	5	32
410	GalNAca1-3(Fuca1-2)Galb1-4GlcNAcb1-3GalNAca-Sp14	17	1	6
489	Fuca1-2Galb1-4GlcNAcb1-6GalNAca-Sp14	17	3	17
502	GlcNAcb1-6(GlcNAcb1-2)Mana1-6(GlcNAcb1-4)(GlcNAcb1-4(GlcNAcb1-2)Mana1-3)Manb1-4GlcNAcb1-4(Fuca1-6)GlcNAc-Sp21	17	2	14
516	Gala1-3Galb1-4GlcNAcb1-2Mana-Sp0	17	2	14
532	Gala1-3(Fuca1-2)Galb1-3GalNAcb1-3Gala1-4Galb1-4Glc-Sp21	17	2	13
310	MurNAcb1-4GlcNAcb-Sp10	17	1	8
377	Galb1-3GalNAca1-3(Fuca1-2)Galb1-4GlcNAc-Sp0	17	2	10
460	Neu5Aca2-6Galb1-4GlcNAcb1-4Mana1-6(GlcNAcb1-4)(Neu5Aca2-6Galb1-4GlcNAcb1-4(Neu5Aca2-6Galb1-4GlcNAcb1-2)Mana1-3)Manb1-4GlcNAcb1-4GlcNAcb-Sp21	17	1	3
545	Neu5Gca2-8Neu5Gca2-3Galb1-4GlcNAcb1-3Galb1-4GlcNAc-Sp0	17	1	8
327	Neu5Aca2-6Galb1-4GlcNAcb1-3Galb1-3GlcNAcb-Sp0	16	1	6
393	GlcNAcb1-2Mana1-6(Galb1-4GlcNAcb1-2Mana1-3)Manb1-4GlcNAcb1-4GlcNAc-Sp12	16	3	16
462	Neu5Aca2-6Galb1-4GlcNAcb1-6(Neu5Aca2-6Galb1-4GlcNAcb1-2)Mana1-6(GlcNAcb1-4)(Neu5Aca2-6Galb1-4GlcNAcb1-4(Neu5Aca2-6Galb1-4GlcNAcb1-2)Mana1-3)Manb1-4GlcNAcb1-4GlcNAcb-Sp21	16	2	14
163	Galb1-4GlcNAcb1-3Galb1-4GlcNAcb-Sp0	16	1	7
164	Galb1-4GlcNAcb1-3Galb1-4Glcb-Sp0	16	3	18
526	Galb1-4GlcNAcb1-2 Mana1-6(GlcNAcb1-4)(Galb1-4GlcNAcb1-2Mana1-3)Manb1-4GlcNAcb1-4(Fuca1-6)GlcNAc-Sp21	16	1	9
292	Neu5Aca2-3Galb1-3GlcNAcb1-3Galb1-3GlcNAcb-Sp0	16	1	6
317	Galb1-4GlcNAcb1-2Mana1-6(Neu5Aca2-6Galb1-4GlcNAcb1-2Mana1-3)Manb1-4GlcNAcb1-4GlcNAcb-Sp12	16	2	11
434	Galb1-4GlcNAcb1-6(Galb1-4GlcNAcb1-2)Mana1-6(GlcNAcb1-4)(Galb1-4GlcNAcb1-4(Galb1-4GlcNAcb1-2)Mana1-3)Manb1-4GlcNAcb1-4GlcNAc-Sp21	16	1	6
501	GalNAca1-3(Fuca1-2)Galb1-3GlcNAcb1-6GalNAca-Sp14	16	2	11
389	GalNAca1-3(Fuca1-2)Galb1-3GalNAca1-3(Fuca1-2)Galb1-4GlcNAcb-Sp0	16	4	25
428	GlcNAcb1-2Mana1-6(GlcNAcb1-4)(GlcNAcb1-4(GlcNAcb1-2)Mana1-3)Manb1-4GlcNAcb1-4GlcNAc-Sp21	16	1	4
510	(3S)GalNAcb1-4GlcNAc-Sp8	16	1	8
274	Neu5Aca2-8Neu5Aca2-3Galb1-4Glcb-Sp0	15	1	8
315	Neu5Aca2-3Galb1-4GlcNAcb1-6(Neu5Aca2-3Galb1-3)GalNAca-Sp14	15	2	10
332	GalNAca1-3(Fuca1-2)Galb1-4GlcNAcb1-3Galb1-4GlcNAcb-Sp0	15	2	15
234	Neu5Aca2-6(Neu5Aca2-3)GalNAca-Sp8	15	2	11
287	Galb1-3GlcNAcb1-3Galb1-3GlcNAcb-Sp0	15	3	18
306	Neu5Aca2-6Galb1-4GlcNAcb1-2Mana1-6(GlcNAcb1-2Mana1-3)Manb1-4GlcNAcb1-4GlcNAcb-Sp12	15	1	9
376	Galb1-3GalNAca1-3(Fuca1-2)Galb1-4Glc-Sp0	15	1	9
435	Galb1-4Galb-Sp10	15	6	41
559	(3S)GlcAb1-3Galb1-4GlcNAcb1-3Galb1-4Glc-Sp0	15	2	14
527	Galb1-4GlcNAcb1-2 Mana1-6(Galb1-4GlcNAcb1-4)(Galb1-4GlcNAcb1-2Mana1-3)Manb1-4GlcNAcb1-4(Fuca1-6)GlcNAc-Sp21	15	2	14
544	Neu5Gca2-8Neu5Aca2-3Galb1-4GlcNAc-Sp0	15	3	17
447	GalNAca1-3(Fuca1-2)Galb1-4GlcNAcb1-6(GalNAca1-3(Fuca1-2)Galb1-4GlcNAcb1-3)GalNAc-Sp14	15	1	9
467	Neu5Aca2-3Galb1-4GlcNAcb1-6(Neu5Aca2-3Galb1-4GlcNAcb1-3)GalNAca-Sp14	15	2	12
522	Gala1-3Galb1-3GlcNAcb1-2Mana-Sp0	15	3	24
474	Neu5Aca2-3Galb1-4GlcNAcb1-6GalNAca-Sp14	14	2	13
258	Neu5Aca2-3Galb1-4GlcNAcb-Sp8	14	4	29
290	Galb1-4(Fuca1-3)GlcNAcb1-3Galb1-3(Fuca1-4)GlcNAcb-Sp0	14	16	116
427	GlcNAcb1-2Mana1-6(GlcNAcb1-4)(GlcNAcb1-2Mana1-3)Manb1-4GlcNAcb1-4GlcNAc-Sp21	14	3	18
525	GlcNAcb1-2 Mana1-6(GlcNAcb1-4)(GlcNAcb1-2Mana1-3)Manb1-4GlcNAcb1-4(Fuca1-6)GlcNAc-Sp21	14	1	6
542	Neu5Gca2-8Neu5Gca2-3Galb1-4GlcNAc-Sp0	14	2	14
285	Neu5Gca-Sp8	14	5	38
386	Fuca1-2Galb1-3GalNAca1-3(Fuca1-2)Galb1-4GlcNAcb-Sp0	13	3	23
547	Neu5Aca2-8Neu5Aca2-3Galb1-4GlcNAc-Sp0	13	2	17
584	GlcNAcb1-3Galb1-4GlcNAcb1-6(Galb1-3)GalNAca-Sp14	13	2	16

571	Galb1-4GlcNAcb1-3Galb1-4GlcNAcb1-3Galb1-4GlcNAcb1-3Galb1-4GlcNAcb1-3Galb1-4GlcNAcb1-2Mana1-6(Galb1-4GlcNAcb1-3Galb1-4GlcNAcb1-3Galb1-4GlcNAcb1-3Galb1-4GlcNAcb1-3Galb1-4GlcNAcb1-2Mana1-3)Manb1-4GlcNAcb1-4(Fuca1-6)GlcNAcb-Sp19	32	2	5
572	Galb1-4GlcNAcb1-3Galb1-4GlcNAcb1-6(Galb1-4GlcNAcb1-3Galb1-4GlcNAb1-2)Mana1-6(Galb1-4GlcNAcb1-3Galb1-4GlcNAcb1-2Mana1-3)Manb1-4GlcNAcb1-4(Fuca1-6)GlcNAcb-Sp24	40	4	11
573	GlcNAcb1-3Galb1-4GlcNAcb1-3Galb1-4GlcNAcb1-6(GlcNAcb1-3Galb1-4GlcNAcb1-3Galb1-4GlcNAb1-2)Mana1-6(GlcNAcb1-3Galb1-4GlcNAcb1-3Galb1-4GlcNAcb1-2Mana1-3)Manb1-4GlcNAcb1-4(Fuca1-6)GlcNAcb-Sp24	50	19	38
574	Galb1-4GlcNAcb1-3Galb1-4GlcNAcb1-3Galb1-4GlcNAcb1-6(Galb1-4GlcNAcb1-3Galb1-4GlcNAcb1-3Galb1-4GlcNAb1-2)Mana1-6(Galb1-4GlcNAcb1-3Galb1-4GlcNAcb1-3Galb1-4GlcNAcb1-2Mana1-3)Manb1-4GlcNAcb1-4(Fuca1-6)GlcNAcb-Sp24	34	1	4
575	GlcNAcb1-3Galb1-4GlcNAcb1-3Galb1-4GlcNAcb1-3Galb1-4GlcNAcb1-6(GlcNAcb1-3Galb1-4GlcNAcb1-3Galb1-4GlcNAcb1-3Galb1-4GlcNAb1-2)Mana1-6(GlcNAcb1-3Galb1-4GlcNAcb1-3Galb1-4GlcNAcb1-3Galb1-4GlcNAcb1-2Mana1-3)Manb1-4GlcNAcb1-4(Fuca1-6)GlcNAcb-Sp24	44	9	21
576	Galb1-4GlcNAcb1-3Galb1-4GlcNAcb1-3Galb1-4GlcNAcb1-3Galb1-4GlcNAcb1-6(Galb1-4GlcNAcb1-3Galb1-4GlcNAcb1-3Galb1-4GlcNAcb1-3Galb1-4GlcNAb1-2)Mana1-6(Galb1-4GlcNAcb1-3Galb1-4GlcNAcb1-3Galb1-4GlcNAcb1-3Galb1-4GlcNAcb1-2Mana1-3)Manb1-4GlcNAcb1-4(Fuca1-6)GlcNAcb-Sp24	36	1	4
577	GlcNAcb1-3Galb1-4GlcNAcb1-3Galb1-4GlcNAcb1-3Galb1-4GlcNAcb1-3Galb1-4GlcNAcb1-6(GlcNAcb1-3Galb1-4GlcNAcb1-3Galb1-4GlcNAcb1-3Galb1-4GlcNAcb1-3Galb1-4GlcNAb1-2)Mana1-6(GlcNAcb1-3Galb1-4GlcNAcb1-3Galb1-4GlcNAcb1-3Galb1-4GlcNAcb1-3Galb1-4GlcNAcb1-2Mana1-3)Manb1-4GlcNAcb1-4(Fuca1-6)GlcNAcb-Sp24	72	11	16
578	Galb1-4GlcNAcb1-3Galb1-4GlcNAcb1-3Galb1-4GlcNAcb1-3Galb1-4GlcNAcb1-3Galb1-4GlcNAcb1-6(Galb1-4GlcNAcb1-3Galb1-4GlcNAcb1-3Galb1-4GlcNAcb1-3Galb1-4GlcNAcb1-3Galb1-4GlcNAb1-2)Mana1-6(Galb1-4GlcNAcb1-3Galb1-4GlcNAcb1-3Galb1-4GlcNAcb1-3Galb1-4GlcNAcb1-3Galb1-4GlcNAcb1-2Mana1-3)Manb1-4GlcNAcb1-4(Fuca1-6)GlcNAcb-Sp24	41	1	2
579	Galb1-4GlcNAcb1-3Galb1-4GlcNAcb1-3GalNAca-Sp14	26	2	9
580	Galb1-4GlcNAcb1-3Galb1-4GlcNAcb1-6(Galb1-3)GalNAca-Sp14	30	3	10
581	Galb1-4GlcNAcb1-3Galb1-4GlcNAcb1-6(Galb1-4GlcNAcb1-3Galb1-4GlcNAcb1-3)GalNAca-Sp14	33	3	11
582	Neu5Aca2-3Galb1-4GlcNAcb1-3Galb1-4GlcNAcb1-3GalNAca-Sp14	11	2	22
583	GlcNAcb1-3Galb1-4GlcNAcb1-3GalNAca-Sp14	26	3	13
584	GlcNAcb1-3Galb1-4GlcNAcb1-6(Galb1-3)GalNAca-Sp14	13	2	16
585	GlcNAcb1-3Galb1-4GlcNAcb1-6(GlcNAcb1-3Galb1-4GlcNAcb1-3)GalNAca-Sp14	24	1	6
586	Neu5Aca2-3Galb1-4GlcNAcb1-3Galb1-4GlcNAcb1-6(Neu5Aca2-3Galb1-4GlcNAcb1-3Galb1-4GlcNAcb1-3)GalNAca-Sp14	13	6	47
587	Neu5Aca2-6Galb1-4GlcNAcb1-3Galb1-4GlcNAcb1-3GalNAca-Sp14	17	2	11
588	GlcNAcb1-3Galb1-4GlcNAcb1-3Galb1-4GlcNAcb1-3GalNAca-Sp14	13	2	15
589	Galb1-4GlcNAcb1-3Galb1-3GalNAca-Sp14	18	2	10
590	Neu5Aca2-3Galb1-4GlcNAcb1-3Galb1-4GlcNAcb1-6(Galb1-3)GalNAca-Sp14	17	2	14
591	Neu5Aca2-6Galb1-4GlcNAcb1-3Galb1-4GlcNAcb1-6(Galb1-3)GalNAca-Sp14	23	5	22
592	Neu5Aca2-6Galb1-4GlcNAcb1-6(Galb1-3)GalNAca-Sp14	35	3	10
593	Neu5Aca2-3Galb1-4GlcNAcb1-3Galb1-4GlcNAcb1-2Mana1-6(Neu5Aca2-3Galb1-4GlcNAcb1-3Galb1-4GlcNAcb1-2Mana1-3)Manb1-4GlcNAcb1-4GlcNAcb-Sp12	28	3	11
594	GlcNAcb1-6(Neu5Aca2-3Galb1-3)GalNAca-Sp14	13	1	11
595	Neu5Aca2-6Galb1-4GlcNAcb1-3Galb1-4GlcNAcb1-6(Neu5Aca2-6Galb1-4GlcNAcb1-3Galb1-4GlcNAcb1-3)GalNAca-Sp14	28	3	10
596	Neu5Aca2-6Galb1-4GlcNAcb1-3Galb1-4GlcNAcb1-3Galb1-4GlcNAcb1-2Mana1-6(Neu5Aca2-6Galb1-4GlcNAcb1-3Galb1-4GlcNAcb1-3Galb1-4GlcNAcb1-2Mana1-3)Manb1-4GlcNAcb1-4GlcNAcb-Sp12	20	3	14
597	Neu5Aca2-3Galb1-4GlcNAcb1-3Galb1-4GlcNAcb1-3Galb1-4GlcNAcb1-2Mana1-6(Neu5Aca2-3Galb1-4GlcNAcb1-3Galb1-4GlcNAcb1-3Galb1-4GlcNAcb1-2Mana1-3)Manb1-4GlcNAcb1-4GlcNAcb-Sp12	51	11	22
598	Neu5Aca2-6Galb1-4GlcNAcb1-3Galb1-4GlcNAcb1-2Mana1-6(Neu5Aca2-6Galb1-4GlcNAcb1-3Galb1-4GlcNAcb1-2Mana1-3)Manb1-4GlcNAcb1-4GlcNAcb-Sp12	27	1	5
599	GlcNAcb1-3Fuca-Sp21	21	2	10
600	Galb1-3GalNAcb1-4(Neu5Aca2-8Neu5Aca2-8Neu5Aca2-3)Galb1-4Glcb-Sp21	13	5	42

586	Neu5Aca2-3Galb1-4GlcNAcb1-3Galb1-4GlcNAcb1-6(Neu5Aca2-3Galb1-4GlcNAcb1-3Galb1-4GlcNAcb1-3)GalNAca-Sp14	13	6	47
304	GalNAcb1-3Galb-Sp8	13	1	11
323	Neu5Aca2-3Galb1-4GlcNAcb1-2Mana1-6(Neu5Aca2-6Galb1-4GlcNAcb1-2Mana1-3)Manb1-4GlcNAcb1-4GlcNAcb-Sp12	13	1	6
329	Neu5Aca2-6Galb1-4GlcNAcb1-3Galb1-4GlcNAcb1-3Galb1-4GlcNAcb-Sp0	13	1	6
403	Galb1-3GlcNAca1-6Galb1-4GlcNAcb-Sp0	13	3	20
594	GlcNAcb1-6(Neu5Aca2-3Galb1-3)GalNAca-Sp14	13	1	11
600	Galb1-3GalNAcb1-4(Neu5Aca2-8Neu5Aca2-8Neu5Aca2-3)Galb1-4Glcb-Sp21	13	5	42
256	Neu5Aca2-3Galb1-4GlcNAcb1-3Galb1-4GlcNAcb1-3Galb1-4GlcNAcb-Sp0	13	2	13
456	Neu5Aca2-3Galb1-4GlcNAcb1-4Mana1-6(GlcNAcb1-4)(Neu5Aca2-3Galb1-4GlcNAcb1-4(Neu5Aca2-3Galb1-4GlcNAcb1-2)Mana1-3)Manb1-4GlcNAcb1-4GlcNAcb-Sp21	13	3	20
330	Gala1-4Galb1-4GlcNAcb1-3Galb1-4Glcb-Sp0	13	1	10
588	GlcNAcb1-3Galb1-4GlcNAcb1-3Galb1-4GlcNAcb1-3GalNAca-Sp14	13	2	15
184	GlcNAcb1-3Galb1-4GlcNAcb1-3Galb1-4GlcNAcb-Sp0	12	3	24
505	Galb1-3(6S)GlcNAcb-Sp8	12	6	52
138	Neu5Aca2-6(Galb1-3)GlcNAcb1-4Galb1-4Glcb-Sp10	12	12	102
551	GlcNAcb1-3Galb1-4GlcNAcb1-6(GlcNAcb1-3Galb1-3)GalNAca-Sp14	12	2	13
530	GlcNAcb1-3Galb1-4GlcNAcb1-6(GlcNAcb1-3)Galb1-4GlcNAc-Sp0	12	1	11
458	Neu5Aca2-3Galb1-4GlcNAcb1-6(Neu5Aca2-3Galb1-4GlcNAcb1-2)Mana1-6(GlcNAcb1-4)(Neu5Aca2-3Galb1-4GlcNAcb1-4(Neu5Aca2-3Galb1-4GlcNAcb1-2)Mana1-3)Manb1-4GlcNAcb1-4GlcNAcb-Sp21	11	1	11
582	Neu5Aca2-3Galb1-4GlcNAcb1-3Galb1-4GlcNAcb1-3GalNAca-Sp14	11	2	22
218	Neu5Aca2-3Galb1-4GlcNAcb1-3Galb1-4(Fuca1-3)GlcNAcb-Sp0	11	5	43
358	Fuca1-2Galb1-4GlcNAcb1-2Mana1-6(Fuca1-2Galb1-4GlcNAcb1-2Mana1-3)Manb1-4GlcNAcb1-4GlcNAcb-Sp20	11	19	181
524	Neu5Aca2-3Galb1-3GalNAcb1-4Galb1-4Glcb-Sp0	10	5	48
308	GlcNAcb1-4GlcNAcb-Sp10	10	1	14
185	GlcNAcb1-3Galb1-4Glcb-Sp0	10	20	210
419	Gala1-3(Fuca1-2)Galb1-3GlcNAcb1-3GalNAc-Sp14	10	3	33
497	Neu5Aca2-6GalNAcb1-4(6S)GlcNAcb-Sp8	8	5	67
335	GlcNAca1-4Galb1-4GlcNAcb1-3Galb1-4GlcNAcb1-3Galb1-4GlcNAcb-Sp0	3	3	88
162	Galb1-4GlcNAcb1-3Galb1-4GlcNAcb1-3Galb1-4GlcNAcb-Sp0	2	17	869
493	Gala1-3(Fuca1-2)Galb1-4GlcNAcb1-6GalNAca-Sp14	2	0	0
115	Gala1-3Galb1-4GlcNAcb-Sp8	1	2	177
88	GlcNAcb1-3Galb1-3GalNAca-Sp8	-1	1	-40

Antibodies to a CA 19-9 Related Antigen Complex Identify a Subset of SOX9 Expressing Cells In Human Foetal Pancreas and Pancreatic Adenocarcinoma. Alison M. Farley, David R. Braxton, Jonathan Li, Karl Trounson, Subhanwita Sakar-Dey, Bhavana Nayer, Tatsuhiko Ikeda, Kevin X. Lau, Winita Hardikar, Kouichi Hasegawa, and Martin F. Pera

Supplementary Table S4. Proteomics Analysis of GCTM-5 Antigen Complex

Mascot Search Results

Host : 10.10.10.100
User : alison@anatomy
Email :
Search title : CPFP SCH6428 [16/18] 140331_alison_set3_7.mgf
MS data file : 140331_alison_set3_7.mgf.mascot
Database : SWISSPROT sprot (538010 sequences; 190998508 residues)
Timestamp : 2 Apr 2014 at 00:36:55 GMT
Protein hits : FCGBP_HUMAN RecName: Full=IgGFc-binding protein; AltName: Full=Fcgamma-binding protein antigen; Short=FcgammaBP; Flags: Prec
TRYP_PIG RecName: Full=Trypsin; EC=3.4.21.4; Flags: Precursor; - OS=Sus scrofa (Pig).
SPG1_STRSG RecName: Full=Immunoglobulin G-binding protein G; Short=IgG-binding protein G; Flags: Precursor; - OS=Streptococ
K2C5_BOVIN RecName: Full=Keratin, type II cytoskeletal 5; AltName: Full=Cytokeratin-5; Short=CK-5; AltName: Full=Keratin-5;
ALBU_BOVIN RecName: Full=Serum albumin; AltName: Full=BSA; AltName: Allergen=Bos d 6; Flags: Precursor; - OS=Bos taurus (Bo
FETUA_BOVIN RecName: Full=Alpha-2-HS-glycoprotein; AltName: Full=Asialofetuin; AltName: Full=Fetuin-A; Flags: Precursor; - O
HEMO_HUMAN RecName: Full=Hemopexin; AltName: Full=Beta-1B-glycoprotein; Flags: Precursor; - OS=Homo sapiens (Human).
SPZ1A_WHEAT RecName: Full=Serpin-Z1A; AltName: Full=TriaeZ1a; AltName: Full=WSZ1a; Short=WSZ1; AltName: Full=WSZCI; - OS=Tri
TRY3_RAT RecName: Full=Cationic trypsin-3; EC=3.4.21.4; AltName: Full=Cationic trypsin III; AltName: Full=Pretrypsinogen
NIFW_AZOCH RecName: Full=Nitrogenase-stabilizing/protective protein NifW OS=Azotobacter chroococcum mcd 1
TOM1_ASHGO RecName: Full=Probable E3 ubiquitin-protein ligase TOM1; EC=6.3.2.- OS=Ashbya gossypii (strain ATCC 10895 / CBS
ISPT_CHLTE RecName: Full=Isoprenyl transferase; EC=2.5.1.-; - OS=Chlorobium tepidum (strain ATCC 49652 / DSM 12025 / TLS).
IMCL1_ORYSJ RecName: Full=Probable isoprenylcysteine alpha-carbonyl methylesterase ICMEL1; EC=3.1.1.n2; AltName: Full=Isopre
S11IP_CHICK RecName: Full=Serine/threonine-protein kinase 11-interacting protein; - OS=Gallus gallus (Chicken).
RAD60_SCHPO RecName: Full=DNA repair protein rad60; - OS=Schizosaccharomyces pombe (strain 972 / ATCC 24843) (Fission yeast)
PNP_CHLPN RecName: Full=Polyribonucleotide nucleotidyltransferase {ECO:0000255|HAMAP-Rule:MF_01595}; EC=2.7.7.8 {ECO:00002
SYGB_AROAE RecName: Full=Glycine--tRNA ligase beta subunit; EC=6.1.1.14; AltName: Full=Glycyl-tRNA synthetase beta subunit;
L_HENDH RecName: Full=RNA-directed RNA polymerase L; Short=Protein L; AltName: Full=Large structural protein; AltName: F
HYI_DANRE RecName: Full=Putative hydroxypyruvate isomerase; EC=5.3.1.22; - OS=Danio rerio (Zebrafish) (Brachydanio rerio).
HYI_XENLA RecName: Full=Putative hydroxypyruvate isomerase; EC=5.3.1.22; - OS=Xenopus laevis (African clawed frog).

	SWISSPROT	Decoy	False discovery rate
Peptide matches above identity threshold	21	0	0.00 %
Peptide matches above homology or identity threshold	24	2	8.33 %

Mascot Score Histogram

Ions score is -10*Log(P), where P is the probability that the observed match is a random event.
Individual ions scores > 36 indicate identity or extensive homology (p<0.05).
Protein scores are derived from ions scores as a non-probabilistic basis for ranking protein hits.

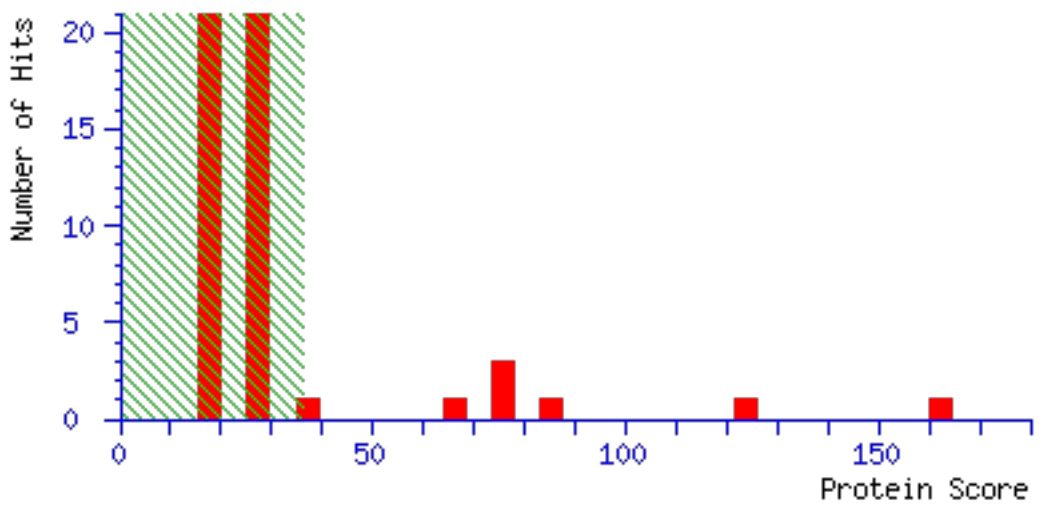

Peptide Summary Report

Format As	Peptide Summary		Help
	Significance threshold p< 0.05	Max. number of hits 20	Show Percolator scores ☐
	Standard scoring ◉ MudPIT scoring ○	Ions score or expect cut-off 0	Show sub-sets 0
	Show pop-ups ◉ Suppress pop-ups ○	Sort unassigned Decreasing Score	Require bold red ☐
	Preferred taxonomy All entries		

1. FCGBP_HUMAN **Mass:** 571639 **Score:** 162 **Matches:** 4(2) **Sequences:** 3(2) **emPAI:** 0.02
RecName: Full=IgGFc-binding protein; AltName: Full=Fcgamma-binding protein antigen; Short=FcgammaBP; Flags: Precursor; - OS=Homo sapiens
☐ Check to include this hit in error tolerant search or archive report

	Query	Observed	Mr(expt)	Mr(calc)	ppm	Miss	Score	Expect	Rank	Unique	Retention Time	Peptide
☑	14	519.2713	1036.5281	1036.5342	-5.84	0	(15)	27	1	U	672s (11.20m)	K.FYPAGDVLR.V
☑	141	519.2733	1036.5320	1036.5342	-2.06	0	29	0.39	1	U	669s (11.15m)	K.FYPAGDVLR.V
☑	293	470.5946	1408.7621	1408.7674	-3.81	0	63	0.00016	1	U	610s (10.17m)	K.AISGLTIDGHAVGAK.L
☑	294	706.8715	1411.7285	1411.7347	-4.42	0	71	2.7e-05	1	U	873s (14.55m)	R.YDLAFVVASQATK.L

2. TRYP_PIG **Mass:** 24394 **Score:** 127 **Matches:** 11(11) **Sequences:** 2(2) **emPAI:** 0.29
RecName: Full=Trypsin; EC=3.4.21.4; Flags: Precursor; - OS=Sus scrofa (Pig).
☐ Check to include this hit in error tolerant search or archive report

	Query	Observed	Mr(expt)	Mr(calc)	ppm	Miss	Score	Expect	Rank	Unique	Retention Time	Peptide
☑	45	421.7545	841.4944	841.5022	-9.15	0	(53)	0.0011	1	U	723s (12.05m)	R.VATVSLPR.S
☑	6	421.7557	841.4969	841.5022	-6.25	0	(54)	0.0014	1	U	728s (12.13m)	R.VATVSLPR.S
☑	46	421.7560	841.4974	841.5022	-5.64	0	60	0.0003	1	U	542s (9.03m)	R.VATVSLPR.S
☑	47	421.7560	841.4974	841.5022	-5.64	0	(57)	0.0007	1	U	539s (8.98m)	R.VATVSLPR.S
☑	48	421.7561	841.4976	841.5022	-5.42	0	(59)	0.00045	1	U	635s (10.58m)	R.VATVSLPR.S
☑	49	421.7561	841.4976	841.5022	-5.42	0	(57)	0.00071	1	U	632s (10.53m)	R.VATVSLPR.S
☑	50	421.7562	841.4978	841.5022	-5.21	0	(58)	0.00053	1	U	828s (13.80m)	R.VATVSLPR.S

	Query	Observed	Mr(expt)	Mr(calc)	ppm	Miss	Score	Expect	Rank	Unique	Retention Time	Peptide
☑	51	421.7563	841.4981	841.5022	-4.85	0	(60)	0.0003	1	U	834s (13.90m)	R.VATVSLPR.S
☑	52	421.7566	841.4986	841.5022	-4.21	0	(48)	0.0052	1	U	818s (13.63m)	R.VATVSLPR.S
☑	143	523.2818	1044.5490	1044.5564	-7.05	0	67	9.7e-05	1	U	507s (8.45m)	K.LSSPATLNSR.V
☑	144	523.2818	1044.5490	1044.5564	-7.05	0	(54)	0.0021	1	U	511s (8.52m)	K.LSSPATLNSR.V

3. SPG1_STRSG **Mass:** 47538 **Score:** 90 **Matches:** 2(2) **Sequences:** 1(1) **emPAI:** 0.07

RecName: Full=Immunoglobulin G-binding protein G; Short=IgG-binding protein G; Flags: Precursor; - OS=Streptococcus sp. group G.

☐ Check to include this hit in error tolerant search or archive report

	Query	Observed	Mr(expt)	Mr(calc)	ppm	Miss	Score	Expect	Rank	Unique	Retention Time	Peptide
☑	302	747.3482	1492.6819	1492.6893	-4.97	0	(76)	3.5e-06	1	U	518s (8.63m)	K.GETTTEAVDAATAEK.V
☑	303	747.3482	1492.6819	1492.6893	-4.97	0	90	1.5e-07	1	U	515s (8.58m)	K.GETTTEAVDAATAEK.V

Proteins matching the same set of peptides:

SPG2_STRSG **Mass:** 63253 **Score:** 89 **Matches:** 2(2) **Sequences:** 1(1)

RecName: Full=Immunoglobulin G-binding protein G; Short=IgG-binding protein G; Flags: Precursor; - OS=Streptococcus sp. group G.

4. K2C5_BOVIN **Mass:** 62898 **Score:** 80 **Matches:** 1(1) **Sequences:** 1(1) **emPAI:** 0.05

RecName: Full=Keratin, type II cytoskeletal 5; AltName: Full=Cytokeratin-5; Short=CK-5; AltName: Full=Keratin-5; Short=K5; AltName: Full

☐ Check to include this hit in error tolerant search or archive report

	Query	Observed	Mr(expt)	Mr(calc)	ppm	Miss	Score	Expect	Rank	Unique	Retention Time	Peptide
☑	250	651.8574	1301.7003	1301.7078	-5.79	0	80	4.6e-06	1	U	1106s (18.43m)	R.SLDLDSIIAEVK.A

Proteins matching the same set of peptides:

K2C5_MOUSE **Mass:** 61729 **Score:** 80 **Matches:** 1(1) **Sequences:** 1(1)

RecName: Full=Keratin, type II cytoskeletal 5; AltName: Full=Cytokeratin-5; Short=CK-5; AltName: Full=Keratin-5; Short=K5; AltName: Full

K2C5_RAT **Mass:** 61788 **Score:** 80 **Matches:** 1(1) **Sequences:** 1(1)

RecName: Full=Keratin, type II cytoskeletal 5; AltName: Full=Cytokeratin-5; Short=CK-5; AltName: Full=Keratin-5; Short=K5; AltName: Full

K2C6A_MOUSE **Mass:** 59299 **Score:** 80 **Matches:** 1(1) **Sequences:** 1(1)

RecName: Full=Keratin, type II cytoskeletal 6A; AltName: Full=Cytokeratin-6A; Short=CK-6A; AltName: Full=Keratin-6-alpha; Short=mK6-alph

K2C6A_RAT **Mass:** 59213 **Score:** 80 **Matches:** 1(1) **Sequences:** 1(1)

RecName: Full=Keratin, type II cytoskeletal 6A; AltName: Full=Cytokeratin-6A; Short=CK-6A; AltName: Full=Keratin-6A; Short=K6A; AltName:

K2C6B_MOUSE **Mass:** 60285 **Score:** 80 **Matches:** 1(1) **Sequences:** 1(1)

RecName: Full=Keratin, type II cytoskeletal 6B; AltName: Full=Cytokeratin-6B; Short=CK-6B; AltName: Full=Keratin-6-beta; Short=mK6-beta;

K2C75_BOVIN **Mass:** 59000 **Score:** 80 **Matches:** 1(1) **Sequences:** 1(1)

RecName: Full=Keratin, type II cytoskeletal 75; AltName: Full=Cytokeratin-75; Short=CK-75; AltName: Full=Keratin-6 hair follicle; AltNam

K2C75_MOUSE **Mass:** 59704 **Score:** 80 **Matches:** 1(1) **Sequences:** 1(1)

RecName: Full=Keratin, type II cytoskeletal 75; AltName: Full=Cytokeratin-75; Short=CK-75; AltName: Full=Keratin-6 hair follicle; Short=

K2C75_RAT **Mass:** 58991 **Score:** 80 **Matches:** 1(1) **Sequences:** 1(1)

RecName: Full=Keratin, type II cytoskeletal 75; AltName: Full=Cytokeratin-75; Short=CK-75; AltName: Full=Keratin-6 hair follicle; AltNam

K2C7_BOVIN **Mass:** 51546 **Score:** 80 **Matches:** 1(1) **Sequences:** 1(1)

RecName: Full=Keratin, type II cytoskeletal 7; AltName: Full=Cytokeratin-7; Short=CK-7; AltName: Full=Keratin-7; Short=K7; AltName: Full

K2C1_HUMAN **Mass:** 65999 **Score:** 79 **Matches:** 1(1) **Sequences:** 1(1)

RecName: Full=Keratin, type II cytoskeletal 1; AltName: Full=67 kDa cytokeratin; AltName: Full=Cytokeratin-1; Short=CK-1; AltName: Full=

K2C1_PANTR **Mass:** 65450 **Score:** 79 **Matches:** 1(1) **Sequences:** 1(1)

RecName: Full=Keratin, type II cytoskeletal 1; AltName: Full=Cytokeratin-1; Short=CK-1; AltName: Full=Keratin-1; Short=K1; AltName: Full

5. ALBU_BOVIN **Mass:** 69248 **Score:** 79 **Matches:** 3(2) **Sequences:** 2(1) **emPAI:** 0.05

RecName: Full=Serum albumin; AltName: Full=BSA; AltName: Allergen=Bos d 6; Flags: Precursor; - OS=Bos taurus (Bovine).

☐ Check to include this hit in error tolerant search or archive report

	Query	Observed	Mr(expt)	Mr(calc)	ppm	Miss	Score	Expect	Rank	Unique	Retention Time	Peptide
☑	124	501.7921	1001.5697	1001.5757	-6.00	0	13	25	1	U	664s (11.07m)	K.LVVSTQTALA.-
☑	193	582.3132	1162.6119	1162.6234	-9.89	0	66	0.00012	1	U	722s (12.03m)	K.LVNELTEFAK.T
☑	194	582.3138	1162.6129	1162.6234	-8.96	0	(56)	0.0012	1	U	724s (12.07m)	K.LVNELTEFAK.T

6. FETUA_BOVIN **Mass:** 38394 **Score:** 72 **Matches:** 2(2) **Sequences:** 1(1) **emPAI:** 0.09

RecName: Full=Alpha-2-HS-glycoprotein; AltName: Full=Asialofetuin; AltName: Full=Fetuin-A; Flags: Precursor; - OS=Bos taurus (Bovine).

☐ Check to include this hit in error tolerant search or archive report

	Query	Observed	Mr(expt)	Mr(calc)	ppm	Miss	Score	Expect	Rank	Unique	Retention Time	Peptide
☑	300	737.9173	1473.8201	1473.8304	-6.96	0	(67)	6.9e-05	1	U	660s (11.00m)	K.TPIVGQPSIPGGPVR.L
☑	301	737.9173	1473.8201	1473.8304	-6.96	0	72	2.1e-05	1	U	657s (10.95m)	K.TPIVGQPSIPGGPVR.L

7. HEMO_HUMAN **Mass:** 51643 **Score:** 66 **Matches:** 2(1) **Sequences:** 2(1) **emPAI:** 0.06

RecName: Full=Hemopexin; AltName: Full=Beta-1B-glycoprotein; Flags: Precursor; - OS=Homo sapiens (Human).

☐ Check to include this hit in error tolerant search or archive report

	Query	Observed	Mr(expt)	Mr(calc)	ppm	Miss	Score	Expect	Rank	Unique	Retention Time	Peptide
☑	30	399.2279	796.4412	796.4378	4.36	0	25	0.54	1	U	523s (8.72m)	R.LHIMAGR.R
☑	225	610.8045	1219.5944	1219.5986	-3.43	0	41	0.021	1	U	758s (12.63m)	K.NFPSPVDAAFR.Q

Proteins matching the same set of peptides:

HEMO_PONAB **Mass:** 51581 **Score:** 66 **Matches:** 2(1) **Sequences:** 2(1)

RecName: Full=Hemopexin; Flags: Precursor; - OS=Pongo abelii (Sumatran orangutan) (Pongo pygmaeus abelii).

8. SPZ1A_WHEAT **Mass:** 43091 **Score:** 33 **Matches:** 2(0) **Sequences:** 1(0)

RecName: Full=Serpin-Z1A; AltName: Full=TriaeZ1a; AltName: Full=WSZ1a; Short=WSZ1; AltName: Full=WSZCI; - OS=Triticum aestivum (Wheat).

☐ Check to include this hit in error tolerant search or archive report

	Query	Observed	Mr(expt)	Mr(calc)	ppm	Miss	Score	Expect	Rank	Unique	Retention Time	Peptide
☑	155	539.7884	1077.5622	1077.5488	12.4	0	33	0.21	1	U	570s (9.50m)	-.MATTLATDVR.L
☑	156	539.7884	1077.5622	1077.5488	12.4	0	(20)	4	1	U	566s (9.43m)	-.MATTLATDVR.L

Proteins matching the same set of peptides:
SPZ1B_WHEAT **Mass:** 43006 **Score:** 33 **Matches:** 2(0) **Sequences:** 1(0)
RecName: Full=Serpin-Z1B; AltName: Full=TriaeZ1b; AltName: Full=WSZ1b; AltName: Full=WZS2; - OS=Triticum aestivum (Wheat).
SPZ1C_WHEAT **Mass:** 42855 **Score:** 33 **Matches:** 2(0) **Sequences:** 1(0)
RecName: Full=Serpin-Z1C; AltName: Full=TriaeZ1c; AltName: Full=WSZ1c; - OS=Triticum aestivum (Wheat).
SPZ2A_WHEAT **Mass:** 43284 **Score:** 33 **Matches:** 2(0) **Sequences:** 1(0)
RecName: Full=Serpin-Z2A; AltName: Full=TriaeZ2a; AltName: Full=WSZ2a; - OS=Triticum aestivum (Wheat).
SPZ2B_WHEAT **Mass:** 42954 **Score:** 33 **Matches:** 2(0) **Sequences:** 1(0)
RecName: Full=Serpin-Z2B; AltName: Full=TriaeZ2b; AltName: Full=WSZ2b; AltName: Full=WZS3; - OS=Triticum aestivum (Wheat).
SPZ4_HORVU **Mass:** 43249 **Score:** 33 **Matches:** 2(0) **Sequences:** 1(0)
RecName: Full=Serpin-Z4; AltName: Full=BSZ4; AltName: Full=HorvuZ4; AltName: Full=Major endosperm albumin; AltName: Full=Protein Z4; Sho

---

9. TRY3_RAT **Mass:** 26252 **Score:** 30 **Matches:** 1(0) **Sequences:** 1(0) **emPAI:** 0.13
RecName: Full=Cationic trypsin-3; EC=3.4.21.4; AltName: Full=Cationic trypsin III; AltName: Full=Pretrypsinogen III; Flags: Precursor; -
☐ Check to include this hit in error tolerant search or archive report

	Query	Observed	Mr(expt)	Mr(calc)	ppm	Miss	Score	Expect	Rank	Unique	Retention Time	Peptide
☑	153	536.7826	1071.5506	1071.5672	-15.54	0	30	0.34	1	U	549s (9.15m)	K.LNSPATLNSR.V

---

10. NIFW_AZOCH **Score:** 30 **Matches:** 2(0) **Sequences:** 2(0)
RecName: Full=Nitrogenase-stabilizing/protective protein NifW OS=Azotobacter chroococcum mcd 1
☐ Check to include this hit in error tolerant search or archive report

Query	Observed	Mr(expt)	Mr(calc)	ppm	Miss	Score	Expect	Rank	Unique	Retention Time	Peptide
30	399.2279	796.4412	796.4378	4.36	0	24	0.6	4	U	523s (8.72m)	R.LHIMQR.Y
376	**747.3751**	**2239.1034**	**2239.0618**	**18.6**	**1**	**6**	**96**	**5**	**U**	**743s (12.38m)**	**K.AGDLDEHDDQARYAVVPAAAR.A**

---

11. TOM1_ASHGO **Score:** 29 **Matches:** 9(0) **Sequences:** 1(0)
RecName: Full=Probable E3 ubiquitin-protein ligase TOM1; EC=6.3.2.- OS=Ashbya gossypii (strain ATCC 10895 / CBS 109.51 / FGSC 9923 / NRR
☐ Check to include this hit in error tolerant search or archive report

Query	Observed	Mr(expt)	Mr(calc)	ppm	Miss	Score	Expect	Rank	Unique	Retention Time	Peptide
45	421.7545	841.4944	841.5021	-9.14	0	(22)	1.4	2	U	723s (12.05m)	R.IGLSSIPR.L
6	421.7557	841.4969	841.5021	-6.24	0	(24)	1.3	2	U	728s (12.13m)	R.IGLSSIPR.L
46	421.7560	841.4974	841.5021	-5.62	0	(28)	0.54	2	U	542s (9.03m)	R.IGLSSIPR.L
47	421.7560	841.4974	841.5021	-5.62	0	(26)	0.77	2	U	539s (8.98m)	R.IGLSSIPR.L
48	421.7561	841.4976	841.5021	-5.40	0	(27)	0.69	2	U	635s (10.58m)	R.IGLSSIPR.L
49	421.7561	841.4976	841.5021	-5.40	0	(27)	0.57	2	U	632s (10.53m)	R.IGLSSIPR.L
50	421.7562	841.4978	841.5021	-5.19	0	(27)	0.66	2	U	828s (13.80m)	R.IGLSSIPR.L
51	421.7563	841.4981	841.5021	-4.83	0	29	0.36	2	U	834s (13.90m)	R.IGLSSIPR.L
52	421.7566	841.4986	841.5021	-4.19	0	(20)	2.7	2	U	818s (13.63m)	R.IGLSSIPR.L

---

12. ISPT_CHLTE **Mass:** 30521 **Score:** 29 **Matches:** 1(0) **Sequences:** 1(0) **emPAI:** 0.11
RecName: Full=Isoprenyl transferase; EC=2.5.1.-; - OS=Chlorobium tepidum (strain ATCC 49652 / DSM 12025 / TLS).
☐ Check to include this hit in error tolerant search or archive report

	Query	Observed	Mr(expt)	Mr(calc)	ppm	Miss	Score	Expect	Rank	Unique	Retention Time	Peptide
☑	212	595.8175	1189.6204	1189.6190	1.20	0	29	0.58	1	U	805s (13.42m)	K.TLDETIELTR.K

---

13. IMCL1_ORYSJ **Mass:** 46728 **Score:** 28 **Matches:** 1(0) **Sequences:** 1(0)
RecName: Full=Probable isoprenylcysteine alpha-carbonyl methylesterase ICMEL1; EC=3.1.1.n2; AltName: Full=Isoprenylcysteine methylestera
☐ Check to include this hit in error tolerant search or archive report

	Query	Observed	Mr(expt)	Mr(calc)	ppm	Miss	Score	Expect	Rank	Unique	Retention Time	Peptide
☑	44	421.2897	840.5648	840.5797	-17.71	0	28	0.16	1	U	983s (16.38m)	R.LTLILLR.Y

---

14. S11IP_CHICK **Mass:** 120157 **Score:** 28 **Matches:** 1(0) **Sequences:** 1(0)
RecName: Full=Serine/threonine-protein kinase 11-interacting protein; - OS=Gallus gallus (Chicken).
☐ Check to include this hit in error tolerant search or archive report

Query	Observed	Mr(expt)	Mr(calc)	ppm	Miss	Score	Expect	Rank	Unique	Retention Time	Peptide
44	421.2897	840.5648	840.5797	-17.71	0	28	0.16	1	U	983s (16.38m)	K.LLTLILR.N

---

15. RAD60_SCHPO **Mass:** 46049 **Score:** 28 **Matches:** 1(0) **Sequences:** 1(0)
RecName: Full=DNA repair protein rad60; - OS=Schizosaccharomyces pombe (strain 972 / ATCC 24843) (Fission yeast).
☐ Check to include this hit in error tolerant search or archive report

Query	Observed	Mr(expt)	Mr(calc)	ppm	Miss	Score	Expect	Rank	Unique	Retention Time	Peptide
44	421.2897	840.5648	840.5797	-17.71	0	28	0.16	1	U	983s (16.38m)	K.LITLLLR.S

---

16. PNP_CHLPN **Score:** 28 **Matches:** 2(0) **Sequences:** 1(0)

RecName: Full=Polyribonucleotide nucleotidyltransferase {ECO:0000255|HAMAP-Rule:MF_01595}; EC=2.7.7.8 {ECO:0000255|HAMAP-Rule:MF_01595};

☐ Check to include this hit in error tolerant search or archive report

Query	Observed	Mr(expt)	Mr(calc)	ppm	Miss	Score	Expect	Rank	Unique	Retention Time	Peptide
155	539.7884	1077.5622	1077.5601	2.01	1	28	0.76	2	U	570s (9.50m)	K.SDTMRALIR.D + Oxidation (M)
156	539.7884	1077.5622	1077.5601	2.01	1	(12)	31	5	U	566s (9.43m)	K.SDTMRALIR.D + Oxidation (M)

---

17. SYGB_AROAE **Mass:** 75809 **Score:** 27 **Matches:** 1(0) **Sequences:** 1(0)

RecName: Full=Glycine--tRNA ligase beta subunit; EC=6.1.1.14; AltName: Full=Glycyl-tRNA synthetase beta subunit; Short=GlyRS; - OS=Aroma

☐ Check to include this hit in error tolerant search or archive report

	Query	Observed	Mr(expt)	Mr(calc)	ppm	Miss	Score	Expect	Rank	Unique	Retention Time	Peptide
☑	101	**478.2996**	**954.5846**	**954.5862**	**-1.68**	**0**	**27**	**0.31**	**1**	**U**	**652s (10.87m)**	**R.LAVTVPTVR.A**

---

18. L_HENDH **Score:** 25 **Matches:** 7(0) **Sequences:** 1(0)

RecName: Full=RNA-directed RNA polymerase L; Short=Protein L; AltName: Full=Large structural protein; AltName: Full=Replicase; AltName:

☐ Check to include this hit in error tolerant search or archive report

Query	Observed	Mr(expt)	Mr(calc)	ppm	Miss	Score	Expect	Rank	Unique	Retention Time	Peptide
45	421.7545	841.4944	841.4810	16.0	0	(21)	1.7	3	U	723s (12.05m)	R.LGTWLPR.G
6	421.7557	841.4969	841.4810	18.9	0	(22)	2.2	3	U	728s (12.13m)	R.LGTWLPR.G
46	421.7560	841.4974	841.4810	19.5	0	(25)	0.96	3	U	542s (9.03m)	R.LGTWLPR.G
47	421.7560	841.4974	841.4810	19.5	0	(25)	0.99	3	U	539s (8.98m)	R.LGTWLPR.G
48	421.7561	841.4976	841.4810	19.7	0	25	0.95	3	U	635s (10.58m)	R.LGTWLPR.G
49	421.7561	841.4976	841.4810	19.7	0	(25)	1	3	U	632s (10.53m)	R.LGTWLPR.G
50	421.7562	841.4978	841.4810	19.9	0	(24)	1.2	3	U	828s (13.80m)	R.LGTWLPR.G

---

19. HYI_DANRE **Mass:** 31166 **Score:** 25 **Matches:** 1(0) **Sequences:** 1(0)

RecName: Full=Putative hydroxypyruvate isomerase; EC=5.3.1.22; - OS=Danio rerio (Zebrafish) (Brachydanio rerio).

☐ Check to include this hit in error tolerant search or archive report

Query	Observed	Mr(expt)	Mr(calc)	ppm	Miss	Score	Expect	Rank	Unique	Retention Time	Peptide
30	399.2279	796.4412	796.4378	4.36	0	25	0.54	1	U	523s (8.72m)	R.IHLMAGR.V

Proteins matching the same set of peptides:

HYI_HUMAN **Mass:** 30387 **Score:** 25 **Matches:** 1(0) **Sequences:** 1(0)

RecName: Full=Putative hydroxypyruvate isomerase; EC=5.3.1.22; AltName: Full=Endothelial cell apoptosis protein E-CE1; - OS=Homo sapiens

HYI_MOUSE **Mass:** 30430 **Score:** 25 **Matches:** 1(0) **Sequences:** 1(0)

RecName: Full=Putative hydroxypyruvate isomerase; EC=5.3.1.22; - OS=Mus musculus (Mouse).

---

20. HYI_XENLA **Mass:** 30964 **Score:** 25 **Matches:** 1(0) **Sequences:** 1(0)

RecName: Full=Putative hydroxypyruvate isomerase; EC=5.3.1.22; - OS=Xenopus laevis (African clawed frog).

☐ Check to include this hit in error tolerant search or archive report

Query	Observed	Mr(expt)	Mr(calc)	ppm	Miss	Score	Expect	Rank	Unique	Retention Time	Peptide
30	399.2279	796.4412	796.4378	4.36	0	25	0.54	1	U	523s (8.72m)	R.IHIMAGR.V

Peptide matches not assigned to protein hits: (no details means no match)

	Query	Observed	Mr(expt)	Mr(calc)	ppm	Miss	Score	Expect	Rank	Unique	Retention Time	Peptide
☑	86	**457.2723**	**912.5300**	**912.5392**	**-10.17**	**1**	**25**	**0.72**	**1**		**586s (9.77m)**	**KLTVPAER**
☑	41	**412.7508**	**823.4871**	**823.5028**	**-19.06**	**0**	**25**	**0.38**	**1**		**569s (9.48m)**	**RPVSLPR**
☑	261	**659.3697**	**1316.7249**	**1316.7122**	**9.64**	**0**	**24**	**1.6**	**1**		**1138s (18.97m)**	**TLDLAMANSLLR**
☑	208	**592.8471**	**1183.6797**	**1183.6938**	**-11.91**	**1**	**24**	**1.2**	**1**		**650s (10.83m)**	**AAPRIAHLAHK**
☑	68	**434.7643**	**867.5141**	**867.5290**	**-17.17**	**1**	**24**	**0.43**	**1**		**646s (10.77m)**	**VDLRLPR**
☑	260	**659.3697**	**1316.7249**	**1316.7122**	**9.64**	**0**	**24**	**1.6**	**1**		**1142s (19.03m)**	**TLDLAMANSLLR**
☑	377	**747.7117**	**2240.1133**	**2240.1194**	**-2.71**	**2**	**23**	**1.5**	**1**		**740s (12.33m)**	**MKREFSVECNVGKPQVAYR**
☑	179	**567.8274**	**1133.6402**	**1133.6193**	**18.4**	**0**	**23**	**1.6**	**1**		**804s (13.40m)**	**IVDILHDPGR**
☑	180	**567.8274**	**1133.6402**	**1133.6193**	**18.4**	**0**	**23**	**1.6**	**1**		**807s (13.45m)**	**IVDILHDPGR**
☑	167	**557.2760**	**1112.5374**	**1112.5574**	**-18.04**	**0**	**22**	**1.3**	**1**		**517s (8.62m)**	**NQGPQESVVR**
☑	97	**475.7258**	**949.4370**	**949.4426**	**-5.96**	**0**	**22**	**1.4**	**1**		**747s (12.45m)**	**NDLTLDML + Oxidation (M)**
☑	207	**592.8353**	**1183.6560**	**1183.6561**	**-0.02**	**1**	**21**	**2**	**1**		**619s (10.32m)**	**LKTVEEAAAPR**
☑	40	**412.7508**	**823.4871**	**823.5028**	**-19.06**	**0**	**21**	**0.97**	**1**		**571s (9.52m)**	**RPVSLPR**
☑	202	**588.8102**	**1175.6057**	**1175.5921**	**11.6**	**0**	**20**	**4.2**	**1**		**788s (13.13m)**	**LTDTELDELK**
☑	109	**488.2790**	**974.5435**	**974.5583**	**-15.19**	**0**	**19**	**5.6**	**1**		**685s (11.42m)**	**LSIMLQVR + Oxidation (M)**
☑	217	**603.8166**	**1205.6187**	**1205.6193**	**-0.47**	**0**	**19**	**5.8**	**1**		**761s (12.68m)**	**WSIPYSQIGR**
☑	342	**896.8987**	**1791.7828**	**1791.7945**	**-6.55**	**0**	**18**	**1.3**	**1**		**907s (15.12m)**	**EVDGGDGGCISLEDLASR**
☑	376	747.3751	2239.1034	2239.1386	-15.69	0	18	5.7	1		743s (12.38m)	HEVVGSGVLIEELWADHPPR
☑	138	**517.2943**	**1032.5740**	**1032.5815**	**-7.27**	**0**	**17**	**6.9**	**1**		**600s (10.00m)**	**SLASVLTTNK**
☑	112	**491.2697**	**980.5249**	**980.5291**	**-4.28**	**0**	**17**	**5.9**	**1**		**557s (9.28m)**	**TLLSSFASR**
☑	17	**564.6244**	**1690.8515**	**1690.8205**	**18.3**	**0**	**16**	**10**	**1**		**535s (8.92m)**	**TIMLGDMTVLSHAMR + Oxidation (M)**
☑	183	**574.8081**	**1147.6017**	**1147.6237**	**-19.16**	**0**	**16**	**11**	**1**		**490s (8.17m)**	**VTVFNLVDNK**
☑	19	**615.8771**	**1229.7396**	**1229.7383**	**1.09**	**0**	**16**	**2.7**	**1**		**619s (10.32m)**	**KPLEIINIYK**
☑	111	**491.2697**	**980.5249**	**980.5291**	**-4.28**	**0**	**16**	**6.8**	**1**		**561s (9.35m)**	**TLLSSFASR**
☑	201	**588.8102**	**1175.6057**	**1175.5921**	**11.6**	**0**	**16**	**11**	**1**		**785s (13.08m)**	**LTDTELDELK**
☑	168	**557.3323**	**1112.6500**	**1112.6376**	**11.1**	**1**	**16**	**8.5**	**1**		**639s (10.65m)**	**VAEAMLKVPR**
☑	25	**385.2014**	**768.3883**	**768.3766**	**15.2**	**1**	**16**	**5.4**	**1**		**569s (9.48m)**	**EGVEAKH**
☑	169	**557.3323**	**1112.6500**	**1112.6376**	**11.1**	**1**	**15**	**9.7**	**1**		**637s (10.62m)**	**VAEAMLKVPR**
☑	251	**655.3217**	**1308.6288**	**1308.6534**	**-18.85**	**1**	**15**	**8.6**	**1**		**627s (10.45m)**	**EQHRIDEVQR**
☑	59	**427.7555**	**853.4965**	**853.5022**	**-6.61**	**0**	**15**	**4.8**	**1**		**618s (10.30m)**	**IIGVSTHK**
☑	98	**475.7258**	**949.4370**	**949.4426**	**-5.96**	**0**	**15**	**6.6**	**1**		**748s (12.47m)**	**NDLTLDML + Oxidation (M)**

☑	199	390.5270	1168.5593	1168.5798	-17.58	0	15	7.6	1	493s (8.22m)	MTYAIVETGGK
☑	309	758.4063	1514.7981	1514.7940	2.68	0	15	14	1	554s (9.23m)	VTLQEGATVEEAIR
☑	332	565.2776	1692.8111	1692.8319	-12.30	1	15	11	1	626s (10.43m)	LGGDVTRVYDPSAESK
☑	127	506.2629	1010.5111	1010.5033	7.80	0	14	10	1	613s (10.22m)	VYLGSETSR
☑	252	655.3217	1308.6288	1308.6534	-18.85	1	14	10	1	630s (10.50m)	EQHRIDEVQR
☑	351	617.6531	1849.9374	1849.9455	-4.36	1	14	16	1	504s (8.40m)	AKTSVQAGEIIDSSVMSK
☑	181	571.7676	1141.5206	1141.5363	-13.80	0	14	5.2	1	711s (11.85m)	EDISGSQAHAK
☑	269	446.8589	1337.5548	1337.5638	-6.73	1	14	1.5	1	482s (8.03m)	MPGRCQSDAAMR + Oxidation (M)
☑	231	625.3059	1248.5972	1248.5955	1.38	1	14	13	1	564s (9.40m)	QLRMMPGQEK + 2 Oxidation (M)
☑	102	478.3116	954.6087	954.5974	11.9	1	14	5.3	1	1020s (17.00m)	RAVEVLLR
☑	308	758.4063	1514.7981	1514.7940	2.69	1	13	19	1	551s (9.18m)	LLSQEIKDVNNDK
☑	88	460.7811	919.5477	919.5412	7.04	0	13	6.8	1	665s (11.08m)	ILISLSMK + Oxidation (M)
☑	340	882.4198	1762.8251	1762.8559	-17.47	1	13	9.9	1	763s (12.72m)	GEPYKMAILDENIDR
☑	184	575.3141	1148.6136	1148.6297	-13.99	0	13	20	1	590s (9.83m)	IGLTALMLACK + Oxidation (M)
☑	374	742.3759	2224.1059	2224.0661	17.9	2	13	17	1	753s (12.55m)	KVSADRWEFANEDFLGGQR
☑	56	425.7588	849.5030	849.4960	8.26	1	13	8.7	1	478s (7.97m)	KIIEYGK
☑	282	695.8433	1389.6720	1389.6962	-17.40	2	13	14	1	808s (13.47m)	MYTQDKSKFVK + Oxidation (M)
☑	37	412.7070	823.3994	823.4076	-9.92	0	13	9.3	1	556s (9.27m)	LSSEFGGK
☑	185	575.3142	1148.6138	1148.6297	-13.88	0	13	21	1	593s (9.88m)	IGLTALMLACK + Oxidation (M)
☑	55	425.7588	849.5030	849.4960	8.26	1	13	9.1	1	481s (8.02m)	KIIEYGK
☑	236	630.8481	1259.6817	1259.6730	6.89	0	13	22	1	1032s (17.20m)	AGMPIGVMVTLR + Oxidation (M)
☑	110	488.7867	975.5588	975.5753	-16.89	1	13	16	1	682s (11.37m)	VKAYLPTGK
☑	123	501.7921	1001.5697	1001.5580	11.7	0	12	30	1	666s (11.10m)	IAGVMVDGIK
☑	69	434.7643	867.5141	867.5290	-17.17	1	12	5.8	1	642s (10.70m)	VDLRLPR
☑	175	565.7684	1129.5223	1129.5325	-9.01	1	12	8.5	1	633s (10.55m)	KESYESMIK + Oxidation (M)
☑	176	565.7684	1129.5223	1129.5181	3.69	1	12	9.4	1	629s (10.48m)	KDVVTMMMK + 3 Oxidation (M)
☑	205	590.2850	1178.5554	1178.5568	-1.13	0	12	14	1	522s (8.70m)	AGEGQAPSTFSK
☑	11	477.8203	953.6260	953.6273	-1.42	1	12	3.2	1	1024s (17.07m)	ILDLKKPK
☑	299	730.9096	1459.8047	1459.8181	-9.14	1	12	24	1	614s (10.23m)	VVLRANVISGMSSK
☑	325	811.9060	1621.7975	1621.8102	-7.86	2	12	24	1	961s (16.02m)	MNAITTIKCPQCRK + Oxidation (M)
☑	200	585.8451	1169.6757	1169.6656	8.65	0	12	21	1	709s (11.82m)	TLGLSPLVNEK
☑	166	557.2448	1112.4750	1112.4847	-8.70	0	11	4.4	1	472s (7.87m)	EGGPNPDANSR
☑	81	896.9026	1791.7906	1791.7945	-2.18	0	11	6.8	1	893s (14.88m)	EVDGGDGGCISLEDLASR
☑	192	580.2601	1158.5056	1158.5049	0.62	0	11	5.3	1	550s (9.17m)	YAEMDVMLR + 2 Oxidation (M)
☑	16	531.7943	1061.5741	1061.5539	19.0	0	11	35	1	1046s (17.43m)	GSDALIMALR + Oxidation (M)
☑	35	404.2367	806.4589	806.4572	2.13	0	11	20	1	546s (9.10m)	ILLMSSK + Oxidation (M)
☑	189	577.8093	1153.6040	1153.6132	-7.93	0	11	22	1	714s (11.90m)	ISFFAQSLGGK
☑	267	446.5290	1336.5652	1336.5724	-5.39	0	11	3.4	1	479s (7.98m)	WGWFDGETPDK
☑	142	522.7723	1043.5301	1043.5247	5.16	0	11	23	1	638s (10.63m)	TPSPAISESR
☑	150	531.7924	1061.5702	1061.5552	14.1	2	11	41	1	1043s (17.38m)	WRMEIRR + Oxidation (M)
☑	210	593.3307	1184.6468	1184.6626	-13.29	2	11	32	1	616s (10.27m)	RADPGKTVSVR
☑	182	574.8081	1147.6017	1147.5945	6.32	2	10	40	1	493s (8.22m)	NANSASKSKNK
☑	239	422.5808	1264.7205	1264.7213	-0.62	1	10	17	1	964s (16.07m)	VKYMATQLLAK
☑	319	783.8704	1565.7263	1565.7321	-3.75	2	10	19	1	620s (10.33m)	EKSNDNDFDINKK
☑	320	783.8704	1565.7263	1565.7429	-10.63	0	10	19	1	617s (10.28m)	QCTNLEALSTLMDK
☑	339	881.9183	1761.8220	1761.8434	-12.18	1	10	21	1	750s (12.50m)	YVAFSASFRSEAGSAGR
☑	113	492.2605	982.5064	982.5196	-13.38	1	10	23	1	523s (8.72m)	DVLKTHDR
☑	209	592.8632	1183.7119	1183.6999	10.2	1	10	22	1	674s (11.23m)	KILVTPPSCVK
☑	60	427.7555	853.4965	853.5021	-6.59	0	10	15	1	615s (10.25m)	VIALGEPR
☑	345	599.2774	1794.8105	1794.8240	-7.54	1	10	12	1	792s (13.20m)	SMGVLSDSEARCGDIQK
☑	268	446.8589	1337.5548	1337.5638	-6.73	1	10	3.3	1	485s (8.08m)	MPGRCQSDAAMR + Oxidation (M)
☑	304	752.8632	1503.7119	1503.7351	-15.44	2	10	22	1	835s (13.92m)	DVKRQDLEQMDK
☑	130	508.2901	1014.5657	1014.5822	-16.24	0	10	58	1	704s (11.73m)	TASVLVGAAAR
☑	266	667.3660	1332.7175	1332.7071	7.83	0	10	44	1	1055s (17.58m)	TLDLAMANSLLR + Oxidation (M)
☑	336	857.4044	1712.7943	1712.8113	-9.91	0	10	21	1	759s (12.65m)	MAGMQLDEIIEYLR + 2 Oxidation (M)
☑	343	897.9066	1793.7987	1793.7924	3.53	0	10	11	1	782s (13.03m)	DGMQEEAIQEIAGMTR + Oxidation (M)
☑	296	473.9201	1418.7384	1418.7592	-14.62	1	10	48	1	543s (9.05m)	AVKGYLEMPQVGK
☑	338	858.4537	1714.8928	1714.9114	-10.89	1	9	49	1	679s (11.32m)	AEVWRALSSSANVLGR
☑	324	804.8968	1607.7790	1607.8076	-17.78	0	9	35	1	945s (15.75m)	ETSMGVQSALLTDIK + Oxidation (M)
☑	36	404.2367	806.4589	806.4572	2.13	0	9	28	1	549s (9.15m)	ILLMSSK + Oxidation (M)
☑	131	508.2901	1014.5657	1014.5822	-16.24	0	9	67	1	700s (11.67m)	TASVLVGAAAR
☑	213	596.3134	1190.6123	1190.5965	13.3	0	9	53	1	769s (12.82m)	NNNTDLMILK + Oxidation (M)
☑	133	509.8019	1017.5892	1017.5719	17.0	2	9	40	1	791s (13.18m)	AGFAAQRGKL
☑	196	583.2875	1164.5604	1164.5631	-2.30	1	9	37	1	701s (11.68m)	EMKALMESAR
☑	128	506.2629	1010.5111	1010.5033	7.80	0	9	37	1	611s (10.18m)	VYLGSETSR
☑	23	382.2175	762.4205	762.4137	8.99	1	9	55	1	751s (12.52m)	TYPARR
☑	149	529.7776	1057.5407	1057.5404	0.33	0	9	42	1	544s (9.07m)	ITEIENPSR
☑	137	514.2965	1026.5785	1026.5709	7.34	1	9	37	1	954s (15.90m)	TPKDLAAPSK
☑	129	507.2712	1012.5279	1012.5263	1.60	1	8	32	1	607s (10.12m)	TDMYKIVK + Oxidation (M)
☑	93	468.2438	934.4730	934.4872	-15.20	0	8	62	1	609s (10.15m)	GAIIDYQR
☑	346	901.9611	1801.9075	1801.9243	-9.32	1	8	57	1	802s (13.37m)	EILLQEEEKMIQQR + Oxidation (M)
☑	305	752.8632	1503.7119	1503.7351	-15.44	2	8	33	1	833s (13.88m)	DVKRQDLEQMDK
☑	78	448.2124	894.4103	894.4236	-14.79	0	8	27	1	574s (9.57m)	YNGLDWK
☑	65	432.7482	863.4818	863.4865	-5.38	0	8	55	1	573s (9.55m)	VLPGEPPR
☑	94	935.9440	1869.8734	1869.8965	-12.31	1	8	29	1	962s (16.03m)	ECPVMETGSLFTSGIKR + Oxidation (M)
☑	132	509.8019	1017.5892	1017.5971	-7.71	1	8	46	1	795s (13.25m)	AGGGKYKPLK
☑	9	463.8037	925.5929	925.5960	-3.34	0	8	12	1	854s (14.23m)	NNIVLILK
☑	7	443.2731	1326.7974	1326.8234	-19.58	2	8	76	1	841s (14.02m)	KNSEKLIALALK
☑	245	649.7953	1297.5761	1297.5833	-5.55	0	8	17	1	491s (8.18m)	HPGSMGANSTPSR
☑	95	472.2854	942.5562	942.5386	18.7	0	8	69	1	949s (15.82m)	DIALDAVVK
☑	190	578.8298	1155.6450	1155.6611	-13.95	1	8	57	1	493s (8.22m)	IIAVDINKDR
☑	331	564.6245	1690.8517	1690.8672	-9.18	1	8	64	1	531s (8.85m)	LTSAEMQERGIIATR + Oxidation (M)
☑	295	473.9201	1418.7384	1418.7188	13.8	0	8	69	1	547s (9.12m)	VATVDTSMGGVAGVR
☑	119	500.7523	999.4901	999.4913	-1.20	0	8	31	1	581s (9.68m)	YLGEEYVK
☑	365	970.9689	1939.9233	1939.9422	-9.70	2	8	39	1	785s (13.08m)	SFSSAVKNELCRVETDR
☑	214	596.3166	1190.6186	1190.6408	-18.59	1	8	74	1	800s (13.33m)	TGDIFVLDRR

☑	361	944.4649	1886.9152	1886.9131	1.12	2	8	51	1	852s (14.20m)	GYMYRETVVRHSMVK + 2 Oxidation (M)
☑	262	663.3440	1324.6735	1324.6874	-10.48	0	8	60	1	737s (12.28m)	EPQEATIALPEK
☑	347	913.9770	1825.9394	1825.9356	2.06	1	8	67	1	760s (12.67m)	NMAKDVHDALVEIISR + Oxidation (M)
☑	337	857.4044	1712.7943	1712.8127	-10.70	2	8	34	1	762s (12.70m)	MMGNQKLWGGRFEK + 2 Oxidation (M)
☑	292	699.9064	1397.7982	1397.7851	9.38	2	7	57	1	1008s (16.80m)	SVAALDVARRNAR
☑	118	496.7971	991.5797	991.5927	-13.04	1	7	33	1	489s (8.15m)	AARIVPHTK
☑	235	630.8481	1259.6817	1259.6730	6.89	0	7	82	1	1029s (17.15m)	AGMPIGVMVTLR + Oxidation (M)
☑	87	457.7684	913.5222	913.5094	14.1	1	7	88	1	604s (10.07m)	VATGRTGPR
☑	218	603.8180	1205.6215	1205.6227	-0.98	0	7	87	1	752s (12.53m)	DAAIVIQCAFR
☑	232	625.8045	1249.5944	1249.5721	17.9	1	7	50	1	567s (9.45m)	AMDAERAESVR + Oxidation (M)
☑	13	518.7632	1035.5118	1035.4985	12.9	0	7	70	1	530s (8.83m)	GYLAAEAGER
☑	8	463.8019	925.5893	925.5960	-7.30	0	7	24	1	850s (14.17m)	NNIVLILK
☑	171	558.2465	1114.4785	1114.5003	-19.51	1	7	13	1	552s (9.20m)	GKHSEEADSR
☑	220	606.3104	1210.6062	1210.6049	1.06	1	7	61	1	640s (10.67m)	VSKAMMANLSK + 2 Oxidation (M)
☑	134	511.7815	1021.5484	1021.5444	3.95	0	7	77	1	524s (8.73m)	ENFVSVTVK
☑	241	639.3013	1276.5881	1276.6081	-15.66	1	7	33	1	811s (13.52m)	RALSDAGMTPDK + Oxidation (M)
☑	344	898.4113	1794.8080	1794.8353	-15.17	1	7	27	1	779s (12.98m)	MSVTLADMGPSRSSANR + Oxidation (M)
☑	354	618.9703	1853.8892	1853.8573	17.2	1	6	62	1	1098s (18.30m)	MISEAIKTNAPTCTVEM + Oxidation (M)
☑	224	610.3289	1218.6433	1218.6278	12.7	0	6	1.1e+02	1	725s (12.08m)	VETQMLLGQGK + Oxidation (M)
☑	311	758.8630	1515.7115	1515.7391	-18.21	2	6	45	1	952s (15.87m)	DKGEKNFAMSYVK
☑	91	468.2248	934.4350	934.4356	-0.65	0	6	47	1	851s (14.18m)	DAASTLSDR
☑	96	472.2854	942.5562	942.5611	-5.14	1	6	1e+02	1	943s (15.72m)	TLTPSLRR
☑	162	550.2325	1098.4504	1098.4434	6.42	0	6	7.5	1	524s (8.73m)	AMAMSDSGASR + Oxidation (M)
☑	28	392.7115	783.4085	783.4239	-19.68	0	6	31	1	556s (9.27m)	AAPGGAGGVK
☑	15	523.3003	1044.5859	1044.5927	-6.49	2	6	1.7e+02	1	803s (13.38m)	ERLGKSIDK
☑	375	742.3759	2224.1059	2224.1236	-7.97	1	6	95	1	749s (12.48m)	EHVALELNTLGDPAERAAYR
☑	230	624.8483	1247.6820	1247.6696	9.95	0	6	1e+02	1	842s (14.03m)	DVILNFMVAAR
☑	188	577.8093	1153.6040	1153.6132	-7.93	0	6	70	1	718s (11.97m)	ISFFAQSLGGK
☑	106	483.2710	964.5275	964.5130	15.0	0	6	93	1	544s (9.07m)	GFFASAIPR
☑	285	699.4052	1396.7959	1396.7714	17.5	1	6	59	1	1027s (17.12m)	TYKNLEVVYIR
☑	73	440.7317	879.4489	879.4425	7.24	1	6	98	1	576s (9.60m)	AFCHFKK
☑	161	549.7343	1097.4540	1097.4733	-17.54	0	6	10	1	520s (8.67m)	TAAMLCETDK + Oxidation (M)
☑	82	456.7705	911.5264	911.5117	16.2	1	5	44	1	838s (13.97m)	FKVYSVIG
☑	114	492.2840	982.5534	982.5560	-2.61	2	5	50	1	931s (15.52m)	NTHKKLDK
☑	244	642.3793	1282.7440	1282.7397	3.37	2	5	61	1	921s (15.35m)	TYLFKINTRK
☑	2	391.2220	1170.6441	1170.6397	3.80	1	5	1.8e+02	1	689s (11.48m)	QPADKALPGFK
☑	281	695.8433	1389.6720	1389.6670	3.61	1	5	84	1	811s (13.52m)	SGAASMPSSPKQAR + Oxidation (M)
☑	229	624.8483	1247.6820	1247.6947	-10.19	0	5	1.1e+02	1	845s (14.08m)	YALGPLLNIMK + Oxidation (M)
☑	139	518.3146	1034.6147	1034.6124	2.18	2	5	35	1	810s (13.50m)	TPSFLKKSK
☑	125	505.2486	1008.4827	1008.4876	-4.84	0	5	78	1	513s (8.55m)	GTYNDNVVK
☑	378	427.3705	2558.1794	2558.1992	-7.76	0	5	48	1	572s (9.53m)	LEGPSTSECLSVPQSSEVSAVPCPR
☑	172	560.8124	1119.6103	1119.5884	19.5	1	5	1.3e+02	1	847s (14.12m)	TGSGTTKNINK
☑	191	580.2601	1158.5056	1158.5049	0.62	0	5	21	1	553s (9.22m)	YAEMDVMLR + 2 Oxidation (M)
☑	57	426.2428	850.4711	850.4661	5.93	2	5	82	1	806s (13.43m)	KYGAREK
☑	145	523.3012	1044.5879	1044.5974	-9.13	2	5	1.4e+02	1	806s (13.43m)	QVRAIMRR + Oxidation (M)
☑	284	699.4052	1396.7959	1396.7827	9.48	1	5	72	1	1037s (17.28m)	LDAIRFIVVTHN
☑	64	432.7476	863.4806	863.4752	6.16	0	5	1.2e+02	1	540s (9.00m)	AASELVFK
☑	75	443.7623	885.5101	885.5171	-7.94	0	5	1.2e+02	1	736s (12.27m)	DVAEVILK
☑	223	608.7684	1215.5221	1215.5232	-0.87	2	5	16	1	514s (8.57m)	RDMMNMMKK + 2 Oxidation (M)
☑	273	451.9311	1352.7714	1352.7564	11.0	2	5	68	1	1014s (16.90m)	NHEIKPKKVFN
☑	349	609.6548	1825.9425	1825.9547	-6.67	1	5	1.3e+02	1	761s (12.68m)	QAGLPHEVLNAKQHER
☑	163	550.7323	1099.4501	1099.4452	4.45	0	5	11	1	528s (8.80m)	MSDTTDSTSR
☑	53	425.2099	848.4052	848.4141	-10.41	0	5	99	1	1481s (24.68m)	GGDGAVAFR
☑	26	779.8384	1557.6622	1557.6916	-18.86	1	4	18	1	597s (9.95m)	MTSSSTPRMHTYK + 2 Oxidation (M)
☑	90	467.7264	933.4382	933.4477	-10.17	0	4	70	1	855s (14.25m)	NDLTLDML
☑	170	558.2431	1114.4716	1114.4639	6.89	0	4	19	1	476s (7.93m)	DHETQNESR
☑	121	501.2882	1000.5619	1000.5553	6.62	0	4	1.9e+02	1	784s (13.07m)	ELSGLGNALK
☑	364	647.6478	1939.9217	1939.9422	-10.53	2	4	88	1	789s (13.15m)	SFSSAVKNELCRVETDR
☑	328	819.9132	1637.8118	1637.8162	-2.64	1	4	1.3e+02	1	624s (10.40m)	DQDVAFRVPEPTHK
☑	43	415.2201	828.4256	828.4130	15.3	0	4	60	1	814s (13.57m)	WQPLQSA
☑	286	699.4052	1396.7959	1396.7787	12.4	1	4	84	1	1040s (17.33m)	NGGGVGDILRKPSK
☑	238	633.3669	1264.7192	1264.6962	18.2	1	4	70	1	918s (15.30m)	TYVIMGIANKR
☑	333	849.4422	1696.8699	1696.9009	-18.26	2	4	1.4e+02	1	667s (11.12m)	KTFSEKSSLIHHQR
☑	315	769.3208	1536.6271	1536.6515	-15.89	0	4	7.7	1	869s (14.48m)	TDNDGWGTNVMPSK + Oxidation (M)
☑	290	699.4060	1396.7974	1396.7786	13.4	2	4	85	1	1011s (16.85m)	ASRLKGVDVPAER
☑	18	590.2561	1178.4977	1178.5051	-6.32	0	4	45	1	654s (10.90m)	SGLEDSNADGSK
☑	219	606.3104	1210.6062	1210.5904	13.1	0	4	1.2e+02	1	643s (10.72m)	IIAFDVMGSDK + Oxidation (M)
☑	335	425.4359	1697.7145	1697.7050	5.55	0	4	18	1	572s (9.53m)	TPSTGTMSSADDLDER + Oxidation (M)
☑	291	699.4060	1396.7974	1396.8038	-4.59	2	4	94	1	1004s (16.73m)	VQSKKNTPAVTPK
☑	329	819.9132	1637.8118	1637.8229	-6.74	2	4	1.5e+02	1	620s (10.33m)	NRGMTALLKVSSCDK + Oxidation (M)
☑	107	487.7886	973.5626	973.5742	-11.94	2	4	1.7e+02	1	781s (13.02m)	EIKAMRVK
☑	306	755.8626	1509.7107	1509.7279	-11.44	1	4	99	1	715s (11.92m)	RLEVNMTDTLMR + 2 Oxidation (M)
☑	275	451.9311	1352.7714	1352.7564	11.0	2	4	91	1	1003s (16.72m)	NHEIKPKKVFN
☑	327	542.6294	1624.8662	1624.8719	-3.50	0	4	1.4e+02	1	1124s (18.73m)	MQQRPIGPLVDSLR + Oxidation (M)
☑	77	448.2124	894.4103	894.3939	18.4	0	3	84	1	577s (9.62m)	MSMAPQSK + Oxidation (M)
☑	348	913.9770	1825.9394	1825.9210	10.0	1	3	1.8e+02	1	756s (12.60m)	IKLESSAGTGHFYTTSK
☑	147	527.2709	1052.5272	1052.5363	-8.62	0	3	1.3e+02	1	690s (11.50m)	NNTNHIVNK
☑	76	443.7623	885.5101	885.5144	-4.89	2	3	1.7e+02	1	738s (12.30m)	AARDAVRK
☑	160	545.3137	1088.6129	1088.6052	7.09	0	3	1.9e+02	1	826s (13.77m)	FLPAIAGMAAK
☑	74	443.2714	884.5282	884.5331	-5.53	1	3	1.8e+02	1	844s (14.07m)	KELTPGLK
☑	297	721.4176	1440.8206	1440.8049	10.9	2	3	1.4e+02	1	1055s (17.58m)	VKTVLRQQELNN
☑	371	698.9808	2093.9206	2093.8882	15.5	0	3	39	1	525s (8.75m)	MLSSSPASCTSPSPGTDNPDK + Oxidation (M)
☑	126	505.2486	1008.4827	1008.4698	12.8	0	3	1.3e+02	1	517s (8.62m)	LCQAFENGK
☑	289	699.4060	1396.7974	1396.7926	3.46	0	3	1.1e+02	1	1001s (16.68m)	GGLVDEAALLQALK
☑	326	542.6294	1624.8662	1624.8461	12.4	0	3	1.6e+02	1	1128s (18.80m)	LINNVVGYYETVNK
☑	360	935.9493	1869.8841	1869.8626	11.5	0	3	1.1e+02	1	966s (16.10m)	TMEDQLSSLGTGSSSGVAK + Oxidation (M)

☑	135	512.2378	1022.4611	1022.4637	-2.53	0	3	93	1	538s (8.97m)	AAQMMGINR + 2 Oxidation (M)
☑	274	451.9311	1352.7714	1352.7564	11.0	2	3	1e+02	1	1009s (16.82m)	NHEIKPKKVFN
☑	353	927.9506	1853.8867	1853.9193	-17.56	0	3	1.4e+02	1	1091s (18.18m)	MISDLPHDLESEILSR
☑	357	935.9480	1869.8814	1869.8448	19.5	1	3	1.1e+02	1	1056s (17.60m)	TVQGGMTSTAAMGKGVTEE + Oxidation (M)
☑	298	721.4178	1440.8210	1440.7977	16.2	2	3	1.5e+02	1	1052s (17.53m)	YTTAKFKVTPTGK
☑	330	546.9448	1637.8127	1637.8117	0.63	1	3	1.9e+02	1	625s (10.42m)	KMLSQGLLDMSEVR + 2 Oxidation (M)
☑	355	618.9703	1853.8892	1853.9054	-8.76	1	3	1.4e+02	1	1093s (18.22m)	TFTRQQMADVSVQTAR + Oxidation (M)
☑	240	635.8013	1269.5881	1269.5659	17.5	0	3	89	1	509s (8.48m)	QAFSDALDMTR + Oxidation (M)
☑	105	483.2710	964.5275	964.5090	19.2	0	3	1.8e+02	1	548s (9.13m)	DPPGQAKPR
☑	24	382.2175	762.4205	762.4235	-3.97	2	3	2.2e+02	1	755s (12.58m)	KSAKSDK
☑	198	584.3531	1166.6916	1166.7135	-18.74	1	3	63	1	859s (14.32m)	LKIGIGKPDAR
☑	136	512.2378	1022.4611	1022.4637	-2.53	0	3	98	1	540s (9.00m)	AAQMMGINR + 2 Oxidation (M)
☑	350	610.3257	1827.9553	1827.9288	14.5	1	3	2.2e+02	1	761s (12.68m)	LYEKLAGMTGTAITESK + Oxidation (M)
☑	85	457.2620	912.5094	912.4916	19.5	0	3	1.1e+02	1	742s (12.37m)	LDNVPDLK
☑	159	545.3137	1088.6129	1088.6342	-19.57	2	3	2.1e+02	1	823s (13.72m)	KAFVAVVKNGG
☑	10	465.7745	929.5344	929.5294	5.34	2	3	2.6e+02	1	748s (12.47m)	KIRSDSPK
☑	276	451.9311	1352.7714	1352.7564	11.0	2	3	1.1e+02	1	1006s (16.77m)	NHEIKPKKVFN
☑	310	758.8630	1515.7115	1515.6875	15.9	1	2	1.1e+02	1	956s (15.93m)	EDEMKEEVIGGHK + Oxidation (M)
☑	280	694.4193	1386.8241	1386.8156	6.12	1	2	75	1	1010s (16.83m)	IESLIKNLVLMV + Oxidation (M)
☑	356	935.9477	1869.8809	1869.9117	-16.50	0	2	1.3e+02	1	1062s (17.70m)	GLAGMNTLAEYCLPFVR + Oxidation (M)
☑	84	457.2620	912.5094	912.4916	19.5	0	2	1.2e+02	1	739s (12.32m)	LDNVPDLK
☑	265	665.3015	1328.5884	1328.6143	-19.49	1	2	60	1	492s (8.20m)	DTHEDGGIVRCK
☑	288	699.4052	1396.7959	1396.7827	9.48	1	2	1.4e+02	1	1033s (17.22m)	LDAIRFIVVTHN
☑	341	890.9001	1779.7856	1779.8098	-13.59	0	2	55	1	785s (13.08m)	GPTIGGADEDQLMAYSR
☑	249	651.2835	1300.5525	1300.5751	-17.39	1	2	27	1	515s (8.58m)	MNMGGSSSTAAKK + 2 Oxidation (M)
☑	61	429.2118	856.4091	856.4225	-15.64	0	2	79	1	1476s (24.60m)	LHNQAMK + Oxidation (M)
☑	323	803.8939	1605.7732	1605.7708	1.49	1	2	2e+02	1	815s (13.58m)	SIIETMNKGGYSYK + Oxidation (M)
☑	38	412.7441	823.4736	823.4664	8.69	2	2	78	1	809s (13.48m)	KHGIQKN
☑	314	769.3208	1536.6271	1536.6085	12.1	1	2	13	1	866s (14.43m)	EENCKCGWDNPSR
☑	122	501.2882	1000.5619	1000.5777	-15.83	2	2	3.6e+02	1	778s (12.97m)	SREIVRNK
☑	165	553.8275	1105.6405	1105.6244	14.6	1	2	1.2e+02	1	832s (13.87m)	TYVIAIRDR
☑	287	699.4052	1396.7959	1396.7714	17.5	1	2	1.6e+02	1	1030s (17.17m)	TYKNLEVVYIR
☑	115	492.2840	982.5534	982.5488	4.72	0	2	1.2e+02	1	927s (15.45m)	TYFLKPSK
☑	20	619.8629	1237.7113	1237.7030	6.73	1	1	1.1e+02	1	899s (14.98m)	YTILTSSAVRK
☑	358	935.9493	1869.8841	1869.8502	18.1	0	1	1.6e+02	1	846s (14.10m)	LPEEAVHGMQNGWMGSK
☑	380	873.3532	2617.0377	2617.0407	-1.16	0	1	4.3	1	497s (8.28m)	HMDDIISGEFSSGMMADWTNDDK + Oxidation (M)
☑	242	639.3013	1276.5881	1276.5909	-2.12	1	1	1.1e+02	1	808s (13.47m)	GNNGGNNGGKNFK
☑	203	589.3409	1176.6673	1176.6503	14.5	2	1	2e+02	1	876s (14.60m)	KFDISKLVNN
☑	92	468.2326	934.4507	934.4356	16.2	0	1	2.2e+02	1	546s (9.10m)	DSITSAGER
☑	362	944.4649	1886.9152	1886.9383	-12.22	2	1	2.3e+02	1	849s (14.15m)	MVFVNKMDRTGADFLK + Oxidation (M)
☑	237	633.3669	1264.7192	1264.6962	18.2	1	1	1.4e+02	1	915s (15.25m)	TYVIMGIANKR
☑	334	849.4422	1696.8699	1696.8389	18.3	1	1	2.9e+02	1	670s (11.17m)	AMININIFMRESSR + Oxidation (M)
☑	368	984.9380	1967.8614	1967.8870	-12.97	0	1	57	1	776s (12.93m)	AGACFTAMSGSGSAVFGLYR + Oxidation (M)
☑	80	448.2562	894.4979	894.4997	-2.04	1	1	1.7e+02	1	838s (13.97m)	KLVFNMK + Oxidation (M)
☑	283	466.6057	1396.7954	1396.7827	9.08	1	1	1.8e+02	1	998s (16.63m)	LDAIRFIVVTHN
☑	206	590.2854	1178.5563	1178.5358	17.4	1	1	1.9e+02	1	514s (8.57m)	KMSLSCHVCR + Oxidation (M)
☑	140	518.3147	1034.6148	1034.6124	2.30	1	1	96	1	806s (13.43m)	YGVKILASGK
☑	363	636.9415	1907.8028	1907.7688	17.8	1	1	28	1	1095s (18.25m)	MHTYTRACCMGGWYGR + Oxidation (M)
☑	158	540.3272	1078.6399	1078.6209	17.7	2	1	1e+02	1	831s (13.85m)	LPKSLYKCK
☑	99	478.2496	954.4847	954.4811	3.80	0	1	1.9e+02	1	563s (9.38m)	FGEASAFVK
☑	366	970.9689	1939.9233	1939.9417	-9.46	2	1	2.1e+02	1	782s (13.03m)	MSMLNKKTIEDIDVCGK + Oxidation (M)
☑	108	487.7887	973.5627	973.5444	18.9	0	1	3.5e+02	1	774s (12.90m)	LTLSDNALK
☑	204	589.3410	1176.6674	1176.6503	14.5	2	0	2.5e+02	1	879s (14.65m)	KFDISKLVNN
☑	228	619.8666	1237.7186	1237.7030	12.6	1	0	1.2e+02	1	903s (15.05m)	YTILTSSAVRK
☑	21	635.8031	1269.5916	1269.5949	-2.64	0	0	1.5e+02	1	505s (8.42m)	VQSHQQASEEK
☑	359	935.9493	1869.8841	1869.8522	17.1	1	0	2.1e+02	1	848s (14.13m)	EVMLTGDLGGMEEAKMK + 2 Oxidation (M)
☑	164	553.8275	1105.6405	1105.6244	14.6	1	0	1.7e+02	1	835s (13.92m)	TYVIAIRDR
☑	307	755.8626	1509.7107	1509.7167	-4.00	0	0	2.1e+02	1	712s (11.87m)	EVTALTSQMDMLR + Oxidation (M)
☑	211	593.3663	1184.7181	1184.7241	-5.02	2	0	1.7e+02	1	677s (11.28m)	KLILDSVNRK
☑	233	628.3807	1254.7469	1254.7408	4.84	1	0	2e+02	1	933s (15.55m)	VVTAALEIQRR
☑	234	628.3807	1254.7469	1254.7296	13.8	2	0	2e+02	1	936s (15.60m)	VVVDALKERLN
☑	258	655.3804	1308.7463	1308.7401	4.74	1	0	1.6e+02	1	942s (15.70m)	GDVINVLLGPKAN
☑	79	448.2562	894.4979	894.4997	-2.04	1	0	2.1e+02	1	834s (13.90m)	KLVFNMK + Oxidation (M)
☑	54	425.2101	848.4055	848.4062	-0.76	0	0	2.8e+02	1	1475s (24.58m)	MVDEALR + Oxidation (M)
☑	379	428.0841	2562.4607	2562.4243	14.2	1	0	71	1	575s (9.58m)	LKDTILHLLAKPTINVWDDVMK
☑	1	*390.7316*	*389.7243*							*780s (13.00m)*	
☑	3	*391.2235*	*390.2162*							*693s (11.55m)*	
☑	4	*413.2369*	*412.2297*							*710s (11.83m)*	
☑	5	*419.3092*	*418.3019*							*39s (0.65m)*	
☑	12	*481.8126*	*480.8053*							*1827s (30.45m)*	
☑	22	*682.3702*	*681.3629*							*762s (12.70m)*	
☑	27	*390.7307*	*779.4469*							*783s (13.05m)*	
☑	29	*393.2081*	*784.4017*							*783s (13.05m)*	
☑	31	*399.2440*	*796.4735*							*784s (13.07m)*	
☑	32	*399.2440*	*796.4735*							*787s (13.12m)*	
☑	33	*404.2302*	*806.4458*							*784s (13.07m)*	
☑	34	*404.2302*	*806.4458*							*781s (13.02m)*	
☑	39	*412.7441*	*823.4736*							*813s (13.55m)*	
☑	42	*413.2360*	*824.4574*							*713s (11.88m)*	
☑	58	*426.2428*	*850.4711*							*809s (13.48m)*	
☑	62	*429.7110*	*857.4074*							*1473s (24.55m)*	
☑	63	*431.2616*	*860.5087*							*484s (8.07m)*	
☑	66	*434.7565*	*867.4984*							*837s (13.95m)*	
☑	67	*434.7565*	*867.4984*							*841s (14.02m)*	
☑	70	*435.2491*	*868.4837*							*734s (12.23m)*	
☑	71	*435.2491*	*868.4837*							*735s (12.25m)*	

☑	72	440.7317	879.4489	573s (9.55m)
☑	83	456.7706	911.5266	868s (14.47m)
☑	89	465.7758	929.5371	758s (12.63m)
☑	100	478.2496	954.4847	559s (9.32m)
☑	103	478.7845	955.5544	897s (14.95m)
☑	104	478.7845	955.5544	901s (15.02m)
☑	116	496.3024	990.5903	787s (13.12m)
☑	117	496.3026	990.5907	784s (13.07m)
☑	120	500.7956	999.5767	928s (15.47m)
☑	146	1046.8002	1045.7929	798s (13.30m)
☑	148	527.2709	1052.5272	687s (11.45m)
☑	151	531.8143	1061.6141	807s (13.45m)
☑	152	531.8143	1061.6141	810s (13.50m)
☑	154	538.6938	1075.3731	541s (9.02m)
☑	157	540.3272	1078.6398	834s (13.90m)
☑	173	562.3405	1122.6665	860s (14.33m)
☑	174	562.3406	1122.6666	857s (14.28m)
☑	177	567.3272	1132.6398	894s (14.90m)
☑	178	567.3272	1132.6398	898s (14.97m)
☑	186	575.8393	1149.6641	858s (14.30m)
☑	187	575.8393	1149.6641	861s (14.35m)
☑	195	582.7570	1163.4994	513s (8.55m)
☑	197	584.3531	1166.6916	862s (14.37m)
☑	215	597.8548	1193.6951	895s (14.92m)
☑	216	597.8548	1193.6951	891s (14.85m)
☑	221	606.3673	1210.7201	890s (14.83m)
☑	222	606.3673	1210.7201	892s (14.87m)
☑	226	611.3532	1220.6919	896s (14.93m)
☑	227	611.3532	1220.6919	899s (14.98m)
☑	243	641.8791	1281.7436	925s (15.42m)
☑	246	650.3928	1298.7711	938s (15.63m)
☑	247	650.3929	1298.7713	934s (15.57m)
☑	248	650.7831	1299.5516	512s (8.53m)
☑	253	437.2553	1308.7440	979s (16.32m)
☑	254	437.2553	1308.7441	954s (15.90m)
☑	255	437.2553	1308.7441	959s (15.98m)
☑	256	437.2554	1308.7443	991s (16.52m)
☑	257	437.2554	1308.7445	988s (16.47m)
☑	259	655.3804	1308.7463	944s (15.73m)
☑	263	663.8938	1325.7730	955s (15.92m)
☑	264	663.8938	1325.7730	951s (15.85m)
☑	270	672.4072	1342.7998	980s (16.33m)
☑	271	672.4072	1342.7998	984s (16.40m)
☑	272	677.3917	1352.7688	965s (16.08m)
☑	277	677.3931	1352.7716	969s (16.15m)
☑	278	685.9058	1369.7971	978s (16.30m)
☑	279	685.9058	1369.7971	981s (16.35m)
☑	312	763.8524	1525.6902	839s (13.98m)
☑	313	768.3145	1534.6144	995s (16.58m)
☑	316	771.3190	1540.6234	829s (13.82m)
☑	317	779.3191	1556.6236	839s (13.98m)
☑	318	779.3191	1556.6236	836s (13.93m)
☑	321	795.7779	1589.5413	886s (14.77m)
☑	322	795.7779	1589.5413	883s (14.72m)
☑	352	927.9506	1853.8867	1088s (18.13m)
☑	367	984.9380	1967.8614	772s (12.87m)
☑	369	665.3075	1992.9006	494s (8.23m)
☑	370	684.3168	2049.9285	494s (8.23m)
☑	372	1066.9533	2131.8921	683s (11.38m)
☑	373	1066.9533	2131.8921	680s (11.33m)
☑	381	927.3676	2779.0810	497s (8.28m)
☑	382	927.3676	2779.0810	495s (8.25m)
☑	383	981.3873	2941.1400	498s (8.30m)
☑	384	981.3873	2941.1400	495s (8.25m)
☑	385	1084.0875	3249.2406	502s (8.37m)

Search Parameters

```
Type of search         : MS/MS Ion Search
Enzyme                 : Trypsin
Variable modifications : Oxidation (M)
Mass values            : Monoisotopic
Protein Mass           : Unrestricted
Peptide Mass Tolerance : ± 20 ppm
Fragment Mass Tolerance: ± 0.2 Da
Max Missed Cleavages   : 2
Instrument type        : ESI-QUAD-TOF
Number of queries      : 385
```

Mascot Search Results

```
Host          : 10.10.10.100
User          : alison@anatomy
Email         :
Search title  : CPFP SCH6428 [17/18] 140331_alison_set3_8.mgf
MS data file  : 140331_alison_set3_8.mgf.mascot
Database      : SWISSPROT sprot (538010 sequences; 190998508 residues)
Timestamp     : 2 Apr 2014 at 00:37:24 GMT
Protein hits  : K2C1_HUMAN RecName: Full=Keratin, type II cytoskeletal 1; AltName: Full=67 kDa cytokeratin; AltName: Full=Cytokeratin-1; Sh
                OVAL_CHICK  RecName: Full=Ovalbumin; AltName: Full=Allergen Gal d II; AltName: Full=Egg albumin; AltName: Full=Plakalbumin;
                FCGBP_HUMAN RecName: Full=IgGFc-binding protein; AltName: Full=Fcgamma-binding protein antigen; Short=FcgammaBP; Flags: Prec
                K1C10_HUMAN RecName: Full=Keratin, type I cytoskeletal 10; AltName: Full=Cytokeratin-10; Short=CK-10; AltName: Full=Keratin-
                TRYP_PIG    RecName: Full=Trypsin; EC=3.4.21.4; Flags: Precursor; - OS=Sus scrofa (Pig).
                ALBU_BOVIN  RecName: Full=Serum albumin; AltName: Full=BSA; AltName: Allergen=Bos d 6; Flags: Precursor; - OS=Bos taurus (Bo
                SPG1_STRSG  RecName: Full=Immunoglobulin G-binding protein G; Short=IgG-binding protein G; Flags: Precursor; - OS=Streptococ
                HEMO_HUMAN  RecName: Full=Hemopexin; AltName: Full=Beta-1B-glycoprotein; Flags: Precursor; - OS=Homo sapiens (Human).
                HSLU_HAHCH  RecName: Full=ATP-dependent protease ATPase subunit HslU; AltName: Full=Unfoldase HslU; - OS=Hahella chejuensis
                INSL_VIGUN  RecName: Full=Insulin-like protein; Contains: RecName: Full=Insulin-like protein B chain; Contains: RecName: Ful
                NDST_CAEEL  RecName: Full=Bifunctional heparan sulfate N-deacetylase/N-sulfotransferase 1; EC=2.8.2.8; AltName: Full=Glucosa
                TGL_BACP2   RecName: Full=Protein-glutamine gamma-glutamyltransferase; EC=2.3.2.13; AltName: Full=Transglutaminase; Short=TG
                S11IP_CHICK RecName: Full=Serine/threonine-protein kinase 11-interacting protein; - OS=Gallus gallus (Chicken).
                RAD60_SCHPO RecName: Full=DNA repair protein rad60; - OS=Schizosaccharomyces pombe (strain 972 / ATCC 24843) (Fission yeast)
                IMCL1_ORYSJ RecName: Full=Probable isoprenylcysteine alpha-carbonyl methylesterase ICMEL1; EC=3.1.1.n2; AltName: Full=Isopre
                DAPAL_PYRIL RecName: Full=Uncharacterized DAPA-like lyase Pisl_0309; EC=4.-.-.-; - OS=Pyrobaculum islandicum (strain DSM 418
                ISPT_CHLTE  RecName: Full=Isoprenyl transferase; EC=2.5.1.-; - OS=Chlorobium tepidum (strain ATCC 49652 / DSM 12025 / TLS).
                MURC_FRAAA  RecName: Full=UDP-N-acetylmuramate--L-alanine ligase {ECO:0000255|HAMAP-Rule:MF_00046}; EC=6.3.2.8 {ECO:0000255|
                TOM1_ASHGO  RecName: Full=Probable E3 ubiquitin-protein ligase TOM1; EC=6.3.2.- OS=Ashbya gossypii (strain ATCC 10895 / CBS
                GP_PTPV     RecName: Full=Envelope glycoprotein; Short=GP; AltName: Full=M polyprotein; Contains: RecName: Full=Non-structur
```

	SWISSPROT	Decoy	False discovery rate
Peptide matches above identity threshold	34	0	0.00 %
Peptide matches above homology or identity threshold	42	4	9.52 %

Mascot Score Histogram

Ions score is -10*Log(P), where P is the probability that the observed match is a random event.
Individual ions scores > 37 indicate identity or extensive homology (p<0.05).
Protein scores are derived from ions scores as a non-probabilistic basis for ranking protein hits.

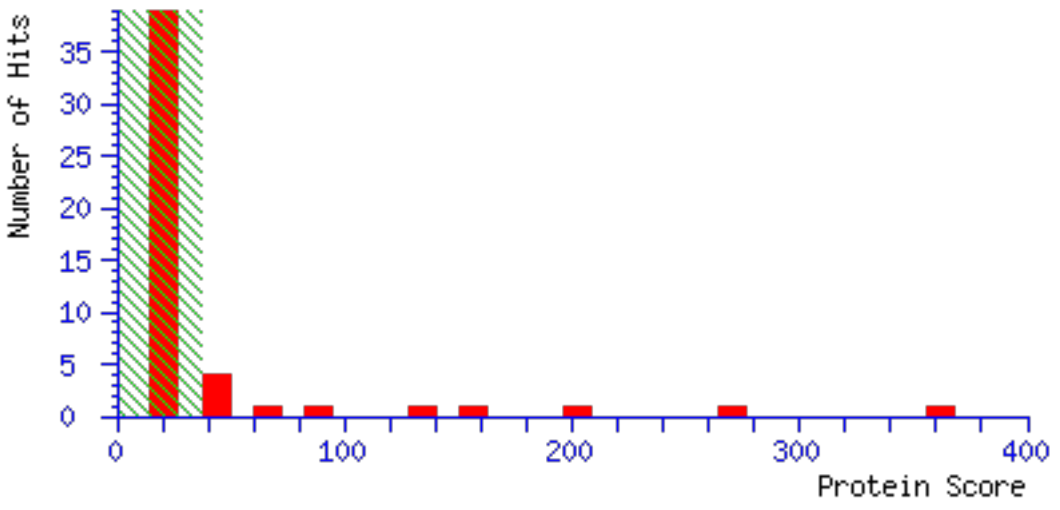

Peptide Summary Report

Format As	Peptide Summary		Help
	Significance threshold p< 0.05	Max. number of hits 20	Show Percolator scores ☐
	Standard scoring ◉ MudPIT scoring ○	Ions score or expect cut-off 0	Show sub-sets 0
	Show pop-ups ◉ Suppress pop-ups ○	Sort unassigned Decreasing Score	Require bold red ☐
	Preferred taxonomy All entries		

1. K2C1_HUMAN **Mass:** 65999 **Score:** 362 **Matches:** 9(8) **Sequences:** 7(6) **emPAI:** 0.40
RecName: Full=Keratin, type II cytoskeletal 1; AltName: Full=67 kDa cytokeratin; AltName: Full=Cytokeratin-1; Short=CK-1; AltName: Full=
☐ Check to include this hit in error tolerant search or archive report

	Query	Observed	Mr(expt)	Mr(calc)	ppm	Miss	Score	Expect	Rank	Unique	Retention Time	Peptide
☑	147	517.2639	1032.5131	1032.5087	4.28	0	38	0.05	1	U	487s (8.12m)	R.TLLEGEESR.M
☑	166	533.2669	1064.5192	1064.5138	5.05	0	29	0.37	1	U	459s (7.65m)	K.AQYEDIAQK.S
☑	259	633.3226	1264.6306	1264.6299	0.57	0	50	0.0037	1	U	580s (9.67m)	R.TNAENEFVTIK.K
☑	276	651.8631	1301.7117	1301.7078	2.95	0	(70)	4.5e-05	1	U	1118s (18.63m)	R.SLDLDSIIAEVK.A
☑	277	651.8631	1301.7117	1301.7078	2.95	0	72	3e-05	1	U	1115s (18.58m)	R.SLDLDSIIAEVK.A
☑	279	436.8904	1307.6495	1307.6469	1.98	2	59	0.00039	1	U	437s (7.28m)	R.NKYEDEINKR.T
☑	322	465.2485	1392.7236	1392.7249	-0.95	1	61	0.00031	1	U	501s (8.35m)	R.TNAENEFVTIKK.D
☑	345	738.3990	1474.7835	1474.7780	3.73	0	(53)	0.0022	1	U	536s (8.93m)	R.FLEQQNQVLQTK.W
☑	346	738.3990	1474.7835	1474.7780	3.73	0	54	0.0018	1	U	540s (9.00m)	R.FLEQQNQVLQTK.W

Proteins matching the same set of peptides:
K2C1_PANTR **Mass:** 65450 **Score:** 362 **Matches:** 9(8) **Sequences:** 7(6)
RecName: Full=Keratin, type II cytoskeletal 1; AltName: Full=Cytokeratin-1; Short=CK-1; AltName: Full=Keratin-1; Short=K1; AltName: Full

2. OVAL_CHICK **Mass:** 42854 **Score:** 276 **Matches:** 5(5) **Sequences:** 3(3) **emPAI:** 0.25
RecName: Full=Ovalbumin; AltName: Full=Allergen Gal d II; AltName: Full=Egg albumin; AltName: Full=Plakalbumin; AltName: Allergen=Gal d
☐ Check to include this hit in error tolerant search or archive report

	Query	Observed	Mr(expt)	Mr(calc)	ppm	Miss	Score	Expect	Rank	Unique	Retention Time	Peptide
[x]	380	799.3639	1596.7132	1596.7090	2.63	0	(69)	1.3e-05	1	U	522s (8.70m)	K.LTEWTSSNVMEER.K + Oxidation (M)
[x]	381	799.3639	1596.7132	1596.7090	2.63	0	93	5.8e-08	1	U	520s (8.67m)	K.LTEWTSSNVMEER.K + Oxidation (M)
[x]	399	844.4255	1686.8365	1686.8325	2.37	0	(61)	0.00027	1	U	707s (11.78m)	R.GGLEPINFQTAADQAR.E
[x]	400	844.4255	1686.8365	1686.8325	2.37	0	101	2.6e-08	1	U	703s (11.72m)	R.GGLEPINFQTAADQAR.E
[x]	409	591.9732	1772.8977	1772.8918	3.33	0	82	2.8e-06	1	U	447s (7.45m)	K.ISQAVHAAHAEINEAGR.E

3. FCGBP_HUMAN **Mass:** 571639 **Score:** 209 **Matches:** 4(3) **Sequences:** 4(3) **emPAI:** 0.02
RecName: Full=IgGFc-binding protein; AltName: Full=Fcgamma-binding protein antigen; Short=FcgammaBP; Flags: Precursor; - OS=Homo sapiens
[ ] Check to include this hit in error tolerant search or archive report

	Query	Observed	Mr(expt)	Mr(calc)	ppm	Miss	Score	Expect	Rank	Unique	Retention Time	Peptide
[x]	6	409.7425	817.4704	817.4658	5.64	0	51	0.0032	1	U	462s (7.70m)	K.LASVSVSR.T
[x]	106	481.7543	961.4941	961.4981	-4.22	0	31	0.32	1	U	484s (8.07m)	R.GNPAVSYVR.V
[x]	296	666.8495	1331.6845	1331.6834	0.82	0	51	0.0033	1	U	712s (11.87m)	R.GEVGFVLVDNQR.S
[x]	330	706.8759	1411.7373	1411.7347	1.83	0	78	5.5e-06	1	U	860s (14.33m)	R.YDLAFVVASQATK.L

4. K1C10_HUMAN **Mass:** 58792 **Score:** 148 **Matches:** 5(5) **Sequences:** 3(3) **emPAI:** 0.18
RecName: Full=Keratin, type I cytoskeletal 10; AltName: Full=Cytokeratin-10; Short=CK-10; AltName: Full=Keratin-10; Short=K10; - OS=Homo
[ ] Check to include this hit in error tolerant search or archive report

	Query	Observed	Mr(expt)	Mr(calc)	ppm	Miss	Score	Expect	Rank	Unique	Retention Time	Peptide
[x]	145	516.3032	1030.5918	1030.5910	0.80	0	46	0.0069	1	U	631s (10.52m)	R.VLDELTLTK.A
[x]	146	516.3032	1030.5918	1030.5910	0.80	0	(46)	0.0077	1	U	634s (10.57m)	R.VLDELTLTK.A
[x]	273	434.2048	1299.5926	1299.5877	3.74	1	38	0.02	1	U	439s (7.32m)	K.NHEEEMKDLR.N
[x]	316	691.3281	1380.6416	1380.6408	0.59	0	63	9e-05	1	U	507s (8.45m)	R.ALEESNYELEGK.I
[x]	317	691.3281	1380.6416	1380.6408	0.59	0	(54)	0.00082	1	U	503s (8.38m)	R.ALEESNYELEGK.I

5. TRYP_PIG **Mass:** 24394 **Score:** 145 **Matches:** 10(10) **Sequences:** 2(2) **emPAI:** 0.29
RecName: Full=Trypsin; EC=3.4.21.4; Flags: Precursor; - OS=Sus scrofa (Pig).
[ ] Check to include this hit in error tolerant search or archive report

	Query	Observed	Mr(expt)	Mr(calc)	ppm	Miss	Score	Expect	Rank	Unique	Retention Time	Peptide
[x]	47	421.7576	841.5007	841.5022	-1.71	0	(55)	0.00095	1	U	678s (11.30m)	R.VATVSLPR.S
[x]	48	421.7578	841.5011	841.5022	-1.22	0	(58)	0.00047	1	U	675s (11.25m)	R.VATVSLPR.S
[x]	49	421.7588	841.5030	841.5022	1.07	0	(57)	0.00056	1	U	495s (8.25m)	R.VATVSLPR.S
[x]	50	421.7588	841.5030	841.5022	1.07	0	58	0.00043	1	U	492s (8.20m)	R.VATVSLPR.S
[x]	51	421.7589	841.5033	841.5022	1.35	0	(58)	0.00044	1	U	586s (9.77m)	R.VATVSLPR.S
[x]	52	421.7590	841.5034	841.5022	1.49	0	(55)	0.001	1	U	582s (9.70m)	R.VATVSLPR.S
[x]	53	421.7591	841.5036	841.5022	1.71	0	(58)	0.00049	1	U	770s (12.83m)	R.VATVSLPR.S
[x]	54	421.7614	841.5083	841.5022	7.29	0	(52)	0.0019	1	U	961s (16.02m)	R.VATVSLPR.S
[x]	154	523.2865	1044.5585	1044.5564	2.03	0	(68)	8e-05	1	U	463s (7.72m)	K.LSSPATLNSR.V
[x]	155	523.2865	1044.5585	1044.5564	2.03	0	86	1.2e-06	1	U	469s (7.82m)	K.LSSPATLNSR.V

6. ALBU_BOVIN **Mass:** 69248 **Score:** 88 **Matches:** 3(2) **Sequences:** 2(1) **emPAI:** 0.05
RecName: Full=Serum albumin; AltName: Full=BSA; AltName: Allergen=Bos d 6; Flags: Precursor; - OS=Bos taurus (Bovine).
[ ] Check to include this hit in error tolerant search or archive report

	Query	Observed	Mr(expt)	Mr(calc)	ppm	Miss	Score	Expect	Rank	Unique	Retention Time	Peptide
	126	501.7966	1001.5787	1001.5757	2.99	0	6	1e+02	9	U	626s (10.43m)	K.LVVSTQTALA.-
[x]	395	547.3197	1638.9372	1638.9305	4.07	1	83	1e-06	1	U	597s (9.95m)	R.KVPQVSTPTLVEVSR.S
[x]	396	547.3197	1638.9374	1638.9305	4.22	1	(67)	4.1e-05	1	U	599s (9.98m)	R.KVPQVSTPTLVEVSR.S

7. SPG1_STRSG **Mass:** 47538 **Score:** 65 **Matches:** 1(1) **Sequences:** 1(1) **emPAI:** 0.07
RecName: Full=Immunoglobulin G-binding protein G; Short=IgG-binding protein G; Flags: Precursor; - OS=Streptococcus sp. group G.
[ ] Check to include this hit in error tolerant search or archive report

	Query	Observed	Mr(expt)	Mr(calc)	ppm	Miss	Score	Expect	Rank	Unique	Retention Time	Peptide
[x]	349	747.3533	1492.6921	1492.6893	1.90	0	65	6.4e-05	1	U	470s (7.83m)	K.GETTTEAVDAATAEK.V

Proteins matching the same set of peptides:
SPG2_STRSG **Mass:** 63253 **Score:** 64 **Matches:** 1(1) **Sequences:** 1(1)
RecName: Full=Immunoglobulin G-binding protein G; Short=IgG-binding protein G; Flags: Precursor; - OS=Streptococcus sp. group G.

8. HEMO_HUMAN **Mass:** 51643 **Score:** 40 **Matches:** 2(1) **Sequences:** 1(1) **emPAI:** 0.06
RecName: Full=Hemopexin; AltName: Full=Beta-1B-glycoprotein; Flags: Precursor; - OS=Homo sapiens (Human).
[ ] Check to include this hit in error tolerant search or archive report

	Query	Observed	Mr(expt)	Mr(calc)	ppm	Miss	Score	Expect	Rank	Unique	Retention Time	Peptide
[x]	240	610.8079	1219.6013	1219.5986	2.26	0	40	0.037	1	U	734s (12.23m)	K.NFPSPVDAAFR.Q
[x]	241	610.8079	1219.6013	1219.5986	2.26	0	(28)	0.59	1	U	737s (12.28m)	K.NFPSPVDAAFR.Q

Proteins matching the same set of peptides:
HEMO_PONAB **Mass:** 51581 **Score:** 40 **Matches:** 2(1) **Sequences:** 1(1)
RecName: Full=Hemopexin; Flags: Precursor; - OS=Pongo abelii (Sumatran orangutan) (Pongo pygmaeus abelii).

9. HSLU_HAHCH **Mass:** 49552 **Score:** 37 **Matches:** 1(0) **Sequences:** 1(0)
RecName: Full=ATP-dependent protease ATPase subunit HslU; AltName: Full=Unfoldase HslU; - OS=Hahella chejuensis (strain KCTC 2396).

Check to include this hit in error tolerant search or archive report

Query	Observed	Mr(expt)	Mr(calc)	ppm	Miss	Score	Expect	Rank	Unique	Retention Time	Peptide
☑ 158	523.7792	1045.5438	1045.5404	3.25	1	37	0.074	1	U	466s (7.77m)	K.LREGTLDDK.E

Proteins matching the same set of peptides:
HSLU_BUCBP **Mass:** 50552 **Score:** 36 **Matches:** 1(0) **Sequences:** 1(0)
RecName: Full=ATP-dependent protease ATPase subunit HslU; AltName: Full=Unfoldase HslU; - OS=Buchnera aphidicola subsp. Baizongia pistac

---

10. INSL_VIGUN **Mass:** 5766 **Score:** 33 **Matches:** 2(0) **Sequences:** 1(0) **emPAI:** 0.63
RecName: Full=Insulin-like protein; Contains: RecName: Full=Insulin-like protein B chain; Contains: RecName: Full=Insulin-like protein A
Check to include this hit in error tolerant search or archive report

Query	Observed	Mr(expt)	Mr(calc)	ppm	Miss	Score	Expect	Rank	Unique	Retention Time	Peptide
☑ 62	430.2220	858.4294	858.4276	2.16	0	33	0.16	1	U	649s (10.82m)	R.GFFYTPK.A
☑ 63	430.2220	858.4294	858.4276	2.16	0	(26)	0.72	1	U	645s (10.75m)	R.GFFYTPK.A

Proteins matching the same set of peptides:
INS_ACOCA **Mass:** 5764 **Score:** 33 **Matches:** 2(0) **Sequences:** 1(0)
RecName: Full=Insulin; Contains: RecName: Full=Insulin B chain; Contains: RecName: Full=Insulin A chain; - OS=Acomys cahirinus (Egyptian
INS_BALBO **Mass:** 5720 **Score:** 33 **Matches:** 2(0) **Sequences:** 1(0)
RecName: Full=Insulin; Contains: RecName: Full=Insulin B chain; Contains: RecName: Full=Insulin A chain; - OS=Balaenoptera borealis (Sei
INS_BALPH **Mass:** 5762 **Score:** 33 **Matches:** 2(0) **Sequences:** 1(0)
RecName: Full=Insulin; Contains: RecName: Full=Insulin B chain; Contains: RecName: Full=Insulin A chain; - OS=Balaenoptera physalus (Fin
INS_BOVIN **Mass:** 11386 **Score:** 33 **Matches:** 2(0) **Sequences:** 1(0)
RecName: Full=Insulin; Contains: RecName: Full=Insulin B chain; Contains: RecName: Full=Insulin A chain; Flags: Precursor; - OS=Bos taur
INS_CAMDR **Mass:** 5690 **Score:** 33 **Matches:** 2(0) **Sequences:** 1(0)
RecName: Full=Insulin; Contains: RecName: Full=Insulin B chain; Contains: RecName: Full=Insulin A chain; - OS=Camelus dromedarius (Drome
INS_CANFA **Mass:** 12182 **Score:** 33 **Matches:** 2(0) **Sequences:** 1(0)
RecName: Full=Insulin; Contains: RecName: Full=Insulin B chain; Contains: RecName: Full=Insulin A chain; Flags: Precursor; - OS=Canis fa
INS_CAPHI **Mass:** 5688 **Score:** 33 **Matches:** 2(0) **Sequences:** 1(0)
RecName: Full=Insulin; Contains: RecName: Full=Insulin B chain; Contains: RecName: Full=Insulin A chain; - OS=Capra hircus (Goat).
INS_CHLAE **Mass:** 12011 **Score:** 33 **Matches:** 2(0) **Sequences:** 1(0)
RecName: Full=Insulin; Contains: RecName: Full=Insulin B chain; Contains: RecName: Full=Insulin A chain; Flags: Precursor; - OS=Chloroce
INS_CRILO **Mass:** 12260 **Score:** 33 **Matches:** 2(0) **Sequences:** 1(0)
RecName: Full=Insulin; Contains: RecName: Full=Insulin B chain; Contains: RecName: Full=Insulin A chain; Flags: Precursor; - OS=Cricetul
INS_DIDVI **Mass:** 5728 **Score:** 33 **Matches:** 2(0) **Sequences:** 1(0)
RecName: Full=Insulin; Contains: RecName: Full=Insulin B chain; Contains: RecName: Full=Insulin A chain; - OS=Didelphis virginiana (Nort
INS_ELEMA **Mass:** 5748 **Score:** 33 **Matches:** 2(0) **Sequences:** 1(0)
RecName: Full=Insulin; Contains: RecName: Full=Insulin B chain; Contains: RecName: Full=Insulin A chain; - OS=Elephas maximus (Indian el
INS_FELCA **Mass:** 12061 **Score:** 33 **Matches:** 2(0) **Sequences:** 1(0)
RecName: Full=Insulin; Contains: RecName: Full=Insulin B chain; Contains: RecName: Full=Insulin A chain; Flags: Precursor; - OS=Felis ca
INS_GORGO **Mass:** 11973 **Score:** 33 **Matches:** 2(0) **Sequences:** 1(0)
RecName: Full=Insulin; Contains: RecName: Full=Insulin B chain; Contains: RecName: Full=Insulin A chain; Flags: Precursor; - OS=Gorilla
INS_HORSE **Mass:** 9140 **Score:** 33 **Matches:** 2(0) **Sequences:** 1(0)
RecName: Full=Insulin; Contains: RecName: Full=Insulin B chain; Contains: RecName: Full=Insulin A chain; Flags: Precursor; - OS=Equus ca
INS_HUMAN **Mass:** 11973 **Score:** 33 **Matches:** 2(0) **Sequences:** 1(0)
RecName: Full=Insulin; Contains: RecName: Full=Insulin B chain; Contains: RecName: Full=Insulin A chain; Flags: Precursor; - OS=Homo sap
INS_MACFA **Mass:** 11983 **Score:** 33 **Matches:** 2(0) **Sequences:** 1(0)
RecName: Full=Insulin; Contains: RecName: Full=Insulin B chain; Contains: RecName: Full=Insulin A chain; Flags: Precursor; - OS=Macaca f
INS_ONCKE **Mass:** 11706 **Score:** 33 **Matches:** 2(0) **Sequences:** 1(0)
RecName: Full=Insulin; Contains: RecName: Full=Insulin B chain; Contains: RecName: Full=Insulin A chain; Flags: Precursor; - OS=Oncorhyn
INS_PANTR **Mass:** 12017 **Score:** 33 **Matches:** 2(0) **Sequences:** 1(0)
RecName: Full=Insulin; Contains: RecName: Full=Insulin B chain; Contains: RecName: Full=Insulin A chain; Flags: Precursor; - OS=Pan trog
INS_PHYMC **Mass:** 5762 **Score:** 33 **Matches:** 2(0) **Sequences:** 1(0)
RecName: Full=Insulin; Contains: RecName: Full=Insulin B chain; Contains: RecName: Full=Insulin A chain; - OS=Physeter macrocephalus (Sp
INS_PIG **Mass:** 11664 **Score:** 33 **Matches:** 2(0) **Sequences:** 1(0)
RecName: Full=Insulin; Contains: RecName: Full=Insulin B chain; Contains: RecName: Full=Insulin A chain; Flags: Precursor; - OS=Sus scro
INS_PLAFE **Mass:** 5751 **Score:** 33 **Matches:** 2(0) **Sequences:** 1(0)
RecName: Full=Insulin; Contains: RecName: Full=Insulin B chain; Contains: RecName: Full=Insulin A chain; - OS=Platichthys flesus (Europe
INS_PONPY **Mass:** 12030 **Score:** 33 **Matches:** 2(0) **Sequences:** 1(0)
RecName: Full=Insulin; Contains: RecName: Full=Insulin B chain; Contains: RecName: Full=Insulin A chain; Flags: Precursor; - OS=Pongo py
INS_PSAOB **Mass:** 12316 **Score:** 33 **Matches:** 2(0) **Sequences:** 1(0)
RecName: Full=Insulin; Contains: RecName: Full=Insulin B chain; Contains: RecName: Full=Insulin A chain; Flags: Precursor; - OS=Psammomy
INS_RABIT **Mass:** 11830 **Score:** 33 **Matches:** 2(0) **Sequences:** 1(0)
RecName: Full=Insulin; Contains: RecName: Full=Insulin B chain; Contains: RecName: Full=Insulin A chain; Flags: Precursor; - OS=Oryctola
INS_SHEEP **Mass:** 11228 **Score:** 33 **Matches:** 2(0) **Sequences:** 1(0)
RecName: Full=Insulin; Contains: RecName: Full=Insulin B chain; Contains: RecName: Full=Insulin A chain; Flags: Precursor; - OS=Ovis ari
INS_SPETR **Mass:** 11996 **Score:** 33 **Matches:** 2(0) **Sequences:** 1(0)
RecName: Full=Insulin; Contains: RecName: Full=Insulin B chain; Contains: RecName: Full=Insulin A chain; Flags: Precursor; - OS=Spermoph
INS_VERMO **Mass:** 12599 **Score:** 33 **Matches:** 2(0) **Sequences:** 1(0)
RecName: Full=Insulin; Contains: RecName: Full=Insulin B chain; Contains: RecName: Full=Insulin A chain; Flags: Precursor; - OS=Verasper
INS1_MOUSE **Mass:** 12152 **Score:** 33 **Matches:** 2(0) **Sequences:** 1(0)
RecName: Full=Insulin-1; Contains: RecName: Full=Insulin-1 B chain; Contains: RecName: Full=Insulin-1 A chain; Flags: Precursor; - OS=Mu
INS1_RAT **Mass:** 12412 **Score:** 33 **Matches:** 2(0) **Sequences:** 1(0)
RecName: Full=Insulin-1; Contains: RecName: Full=Insulin-1 B chain; Contains: RecName: Full=Insulin-1 A chain; Flags: Precursor; - OS=Ra

---

11. NDST_CAEEL **Mass:** 98998 **Score:** 33 **Matches:** 2(0) **Sequences:** 1(0) **emPAI:** 0.03
RecName: Full=Bifunctional heparan sulfate N-deacetylase/N-sulfotransferase 1; EC=2.8.2.8; AltName: Full=Glucosaminyl N-deacetylase/N-su
Check to include this hit in error tolerant search or archive report

Query	Observed	Mr(expt)	Mr(calc)	ppm	Miss	Score	Expect	Rank	Unique	Retention Time	Peptide
☑ 207	578.8335	1155.6524	1155.6724	-17.29	1	(32)	0.21	1	U	448s (7.47m)	K.NLPVLTTSRR.G

☑	208	578.8335	1155.6524	1155.6724	-17.29	1	33	0.19	1	U	446s (7.43m)	K.NLPVLTTSRR.G

12. TGL_BACP2 **Mass:** 28393 **Score:** 31 **Matches:** 2(0) **Sequences:** 1(0) **emPAI:** 0.12
RecName: Full=Protein-glutamine gamma-glutamyltransferase; EC=2.3.2.13; AltName: Full=Transglutaminase; Short=TGase; - OS=Bacillus pumil
☐ Check to include this hit in error tolerant search or archive report

	Query	Observed	Mr(expt)	Mr(calc)	ppm	Miss	Score	Expect	Rank	Unique	Retention Time	Peptide
☑	459	755.7241	2264.1505	2264.1583	-3.42	1	(26)	1	1	U	817s (13.62m)	R.QASDLLFEVTLRSNIMNAAR.D + Oxidation (M)
☑	460	755.7241	2264.1505	2264.1583	-3.42	1	31	0.35	1	U	820s (13.67m)	R.QASDLLFEVTLRSNIMNAAR.D + Oxidation (M)

13. S11IP_CHICK **Mass:** 120157 **Score:** 30 **Matches:** 1(0) **Sequences:** 1(0)
RecName: Full=Serine/threonine-protein kinase 11-interacting protein; - OS=Gallus gallus (Chicken).
☐ Check to include this hit in error tolerant search or archive report

	Query	Observed	Mr(expt)	Mr(calc)	ppm	Miss	Score	Expect	Rank	Unique	Retention Time	Peptide
☑	46	421.2917	840.5689	840.5797	-12.76	0	30	0.094	1	U	973s (16.22m)	K.LLTLILR.N

14. RAD60_SCHPO **Mass:** 46049 **Score:** 30 **Matches:** 1(0) **Sequences:** 1(0)
RecName: Full=DNA repair protein rad60; - OS=Schizosaccharomyces pombe (strain 972 / ATCC 24843) (Fission yeast).
☐ Check to include this hit in error tolerant search or archive report

Query	Observed	Mr(expt)	Mr(calc)	ppm	Miss	Score	Expect	Rank	Unique	Retention Time	Peptide
46	421.2917	840.5689	840.5797	-12.76	0	30	0.094	1	U	973s (16.22m)	K.LITLLLR.S

15. IMCL1_ORYSJ **Score:** 30 **Matches:** 1(0) **Sequences:** 1(0)
RecName: Full=Probable isoprenylcysteine alpha-carbonyl methylesterase ICMEL1; EC=3.1.1.n2; AltName: Full=Isoprenylcysteine methylestera
☐ Check to include this hit in error tolerant search or archive report

Query	Observed	Mr(expt)	Mr(calc)	ppm	Miss	Score	Expect	Rank	Unique	Retention Time	Peptide
46	421.2917	840.5689	840.5797	-12.76	0	30	0.096	3	U	973s (16.22m)	R.LTLILLR.Y

16. DAPAL_PYRIL **Mass:** 33205 **Score:** 30 **Matches:** 1(0) **Sequences:** 1(0)
RecName: Full=Uncharacterized DAPA-like lyase Pisl_0309; EC=4.-.-.-; - OS=Pyrobaculum islandicum (strain DSM 4184 / JCM 9189).
☐ Check to include this hit in error tolerant search or archive report

	Query	Observed	Mr(expt)	Mr(calc)	ppm	Miss	Score	Expect	Rank	Unique	Retention Time	Peptide
☑	5	393.7451	785.4756	785.4759	-0.47	0	30	0.54	1	U	483s (8.05m)	K.VATQIVR.G

17. ISPT_CHLTE **Mass:** 30521 **Score:** 30 **Matches:** 2(0) **Sequences:** 1(0) **emPAI:** 0.11
RecName: Full=Isoprenyl transferase; EC=2.5.1.-; - OS=Chlorobium tepidum (strain ATCC 49652 / DSM 12025 / TLS).
☐ Check to include this hit in error tolerant search or archive report

	Query	Observed	Mr(expt)	Mr(calc)	ppm	Miss	Score	Expect	Rank	Unique	Retention Time	Peptide
☑	19	595.8217	1189.6289	1189.6190	8.28	0	30	0.47	1	U	777s (12.95m)	K.TLDETIELTR.K
☑	226	595.8227	1189.6309	1189.6190	9.96	0	(19)	5.6	1	U	773s (12.88m)	K.TLDETIELTR.K

18. MURC_FRAAA **Score:** 29 **Matches:** 1(0) **Sequences:** 1(0)
RecName: Full=UDP-N-acetylmuramate--L-alanine ligase {ECO:0000255|HAMAP-Rule:MF_00046}; EC=6.3.2.8 {ECO:0000255|HAMAP-Rule:MF_00046}; Al
☐ Check to include this hit in error tolerant search or archive report

Query	Observed	Mr(expt)	Mr(calc)	ppm	Miss	Score	Expect	Rank	Unique	Retention Time	Peptide
5	393.7451	785.4756	785.4872	-14.75	1	29	0.69	2	U	483s (8.05m)	R.VATLRAR.G

Proteins matching the same set of peptides:
MURC_FRASC **Score:** 29 **Matches:** 1(0) **Sequences:** 1(0)
RecName: Full=UDP-N-acetylmuramate--L-alanine ligase {ECO:0000255|HAMAP-Rule:MF_00046}; EC=6.3.2.8 {ECO:0000255|HAMAP-Rule:MF_00046}; Al

19. TOM1_ASHGO **Score:** 29 **Matches:** 8(0) **Sequences:** 1(0)
RecName: Full=Probable E3 ubiquitin-protein ligase TOM1; EC=6.3.2.- OS=Ashbya gossypii (strain ATCC 10895 / CBS 109.51 / FGSC 9923 / NRR
☐ Check to include this hit in error tolerant search or archive report

Query	Observed	Mr(expt)	Mr(calc)	ppm	Miss	Score	Expect	Rank	Unique	Retention Time	Peptide
47	421.7576	841.5007	841.5021	-1.70	0	(24)	1.1	2	U	678s (11.30m)	R.IGLSSIPR.L
48	421.7578	841.5011	841.5021	-1.20	0	(27)	0.58	2	U	675s (11.25m)	R.IGLSSIPR.L
49	421.7588	841.5030	841.5021	1.08	0	(27)	0.59	2	U	495s (8.25m)	R.IGLSSIPR.L
50	421.7588	841.5030	841.5021	1.08	0	(27)	0.54	2	U	492s (8.20m)	R.IGLSSIPR.L
51	421.7589	841.5033	841.5021	1.37	0	29	0.4	2	U	586s (9.77m)	R.IGLSSIPR.L
52	421.7590	841.5034	841.5021	1.51	0	(25)	0.97	2	U	582s (9.70m)	R.IGLSSIPR.L
53	421.7591	841.5036	841.5021	1.73	0	(27)	0.6	2	U	770s (12.83m)	R.IGLSSIPR.L
54	421.7614	841.5083	841.5021	7.31	0	(24)	1.3	2	U	961s (16.02m)	R.IGLSSIPR.L

20. GP_PTPV **Mass:** 146280 **Score:** 28 **Matches:** 1(0) **Sequences:** 1(0)
RecName: Full=Envelope glycoprotein; Short=GP; AltName: Full=M polyprotein; Contains: RecName: Full=Non-structural protein NSm; Contains
☐ Check to include this hit in error tolerant search or archive report

Query	Observed	Mr(expt)	Mr(calc)	ppm	Miss	Score	Expect	Rank	Unique	Retention Time	Peptide

☑	171	539.7909	1077.5673	1077.5852	-16.59	0	28	0.6	1	U	526s (8.77m)	K.CSITATITLR.A

Peptide matches not assigned to protein hits: (no details means no match)

	Query	Observed	Mr(expt)	Mr(calc)	ppm	Miss	Score	Expect	Rank	Unique	Retention Time	Peptide
☑	219	588.8145	1175.6145	1175.6034	9.43	0	28	0.75	1		762s (12.70m)	TIDETLETVR
☑	101	478.3010	954.5874	954.5862	1.28	0	28	0.26	1		615s (10.25m)	LAVTVPTVR
☑	458	747.3808	2239.1205	2239.1386	-8.05	0	25	1.1	1		720s (12.00m)	HEVVGSGVLIEELWADHPPR
☑	40	412.7537	823.4928	823.5028	-12.17	0	25	0.38	1		530s (8.83m)	RPVSLPR
☑	102	478.3011	954.5876	954.5862	1.49	0	24	0.52	1		618s (10.30m)	LAVTVPTVR
☑	290	659.3718	1316.7291	1316.7122	12.8	0	24	1.4	1		1145s (19.08m)	TLDLAMANSLLR
☑	369	781.3926	1560.7706	1560.7817	-7.12	0	22	2	1		801s (13.35m)	MAVIQQLDDALSNK + Oxidation (M)
☑	438	652.0303	1953.0690	1953.0571	6.12	0	22	1.6	1		683s (11.38m)	VAPEEHPVLLTEAPINPK
☑	182	557.3343	1112.6540	1112.6376	14.8	1	22	1.6	1		599s (9.98m)	VAEAMLKVPR
☑	72	435.7751	869.5356	869.5195	18.5	2	22	1.6	1		551s (9.18m)	KNRISPR
☑	291	659.3718	1316.7291	1316.7122	12.8	0	21	2.6	1		1148s (19.13m)	TLDLAMANSLLR
☑	98	475.7285	949.4424	949.4426	-0.29	0	21	1.6	1		719s (11.98m)	NDLTLDML + Oxidation (M)
☑	224	592.8344	1183.6543	1183.6561	-1.44	1	21	2.2	1		583s (9.72m)	LKTVEEAAAPR
☑	368	781.3926	1560.7706	1560.8008	-19.36	1	21	2.8	1		798s (13.30m)	LAFNLQVDARSDGR
☑	39	412.7537	823.4928	823.5028	-12.17	0	21	0.99	1		525s (8.75m)	RPVSLPR
☑	185	560.2986	1118.5827	1118.5753	6.62	2	20	4.1	1		989s (16.48m)	EIAREMKDK
☑	269	649.8756	1297.7367	1297.7288	6.07	1	20	2.8	1		646s (10.77m)	NLRINMTAIPR
☑	85	454.2335	906.4524	906.4487	4.05	0	20	3.5	1		601s (10.02m)	SYFLSYK
☑	112	488.2830	974.5515	974.5549	-3.51	0	20	4.8	1		654s (10.90m)	AELFLLNR
☑	228	596.3176	1190.6207	1190.6329	-10.25	1	20	4.8	1		676s (11.27m)	TLSRMPTEIK + Oxidation (M)
☑	408	881.9263	1761.8380	1761.8659	-15.86	0	20	3	1		729s (12.15m)	LNHYPHQLSGGQQQR
☑	34	404.2380	806.4614	806.4538	9.41	0	20	3	1		501s (8.35m)	IAASLGIY
☑	127	502.2933	1002.5721	1002.5896	-17.44	0	19	4.7	1		558s (9.30m)	IMASLILAR + Oxidation (M)
☑	197	569.2946	1136.5747	1136.5756	-0.75	1	19	4.9	1		613s (10.22m)	GCKMMIDVIK
☑	168	534.7622	1067.5099	1067.5247	-13.85	0	18	3	1		476s (7.93m)	SSSIFGDSLR
☑	211	582.7995	1163.5844	1163.5856	-1.00	0	18	6	1		655s (10.92m)	MIQVSQDSIK + Oxidation (M)
☑	222	590.2940	1178.5735	1178.5906	-14.50	1	18	4.8	1		477s (7.95m)	QKCFNFPPAK
☑	232	602.3361	1202.6576	1202.6619	-3.53	1	18	7.2	1		804s (13.40m)	TLNTEGVVKSR
☑	415	598.9438	1793.8095	1793.7924	9.54	0	18	1.9	1		771s (12.85m)	DGMQEEAIQEIAGMTR + Oxidation (M)
☑	183	557.3363	1112.6580	1112.6376	18.4	1	18	4.6	1		602s (10.03m)	VAEAMLKVPR
☑	457	747.3808	2239.1205	2239.1195	0.48	0	17	6.8	1		723s (12.05m)	VSFTPQQLSENFVALMDAVK + Oxidation (M)
☑	227	596.3176	1190.6207	1190.6295	-7.43	1	17	8.1	1		679s (11.32m)	EGRDFIEVVK
☑	99	475.7285	949.4424	949.4426	-0.29	0	17	4.1	1		715s (11.92m)	NDLTLDML + Oxidation (M)
☑	218	588.8145	1175.6145	1175.5921	19.0	0	17	8.4	1		764s (12.73m)	LTDTELDELK
☑	71	435.7559	869.4972	869.5123	-17.35	0	17	3.2	1		681s (11.35m)	WIVSPLR
☑	382	804.9026	1607.7906	1607.8117	-13.11	0	17	6.8	1		941s (15.68m)	LPMFGETDIDTILK + Oxidation (M)
☑	356	758.4117	1514.8088	1514.8053	2.30	0	17	8.2	1		516s (8.60m)	GQEVVLVSSGSVAAGR
☑	196	567.8315	1133.6484	1133.6332	13.4	0	17	5.3	1		787s (13.12m)	EVVLEIAFSK
☑	275	434.5847	1300.7322	1300.7285	2.86	1	17	8.1	1		550s (9.17m)	DTTKLALMRPR
☑	100	476.2837	950.5527	950.5661	-14.08	1	17	3.8	1		608s (10.13m)	EGLKHVLR
☑	403	429.0081	1712.0031	1711.9906	7.33	1	16	2.6	1		531s (8.85m)	LMNLKNLADLVLEVK
☑	215	390.5296	1168.5669	1168.5836	-14.33	0	16	5.5	1		445s (7.42m)	LQHNESVQSK
☑	414	598.9438	1793.8095	1793.7924	9.54	0	16	2.9	1		774s (12.90m)	DGMQEEAIQEIAGMTR + Oxidation (M)
☑	113	488.2830	974.5515	974.5549	-3.51	0	16	12	1		651s (10.85m)	AELFLLNR
☑	419	902.4635	1802.9124	1802.9019	5.88	1	16	11	1		762s (12.70m)	AEMEKLGMHSLLSVAR + 2 Oxidation (M)
☑	407	881.9260	1761.8374	1761.8659	-16.15	0	15	7.7	1		726s (12.10m)	LNHYPHQLSGGQQQR
☑	12	477.8215	953.6284	953.6273	1.18	1	15	1.4	1		1010s (16.83m)	ILDLKKPK
☑	385	805.3958	1608.7771	1608.8082	-19.37	1	15	9.1	1		887s (14.78m)	YRNTWDCGLQILK
☑	280	655.3251	1308.6357	1308.6534	-13.55	1	15	9.5	1		594s (9.90m)	EQHRIDEVQR
☑	423	617.6559	1849.9459	1849.9455	0.21	1	15	13	1		457s (7.62m)	AKTSVQAGEIIDSSVMSK
☑	167	534.7621	1067.5096	1067.4957	13.0	0	15	6.6	1		480s (8.00m)	NMEVFEVGK + Oxidation (M)
☑	301	671.3431	1340.6716	1340.6684	2.38	1	15	9.1	1		543s (9.05m)	EALELRDGDPAR
☑	244	615.8856	1229.7566	1229.7496	5.66	1	15	2.1	1		583s (9.72m)	KVPLVITGFTR
☑	249	625.3085	1248.6025	1248.5955	5.66	1	14	11	1		518s (8.63m)	QLRMMPGQEK + 2 Oxidation (M)
☑	299	446.5303	1336.5689	1336.5724	-2.63	0	14	2.2	1		430s (7.17m)	WGWFDGETPDK
☑	116	492.2608	982.5071	982.5083	-1.28	0	14	8.7	1		475s (7.92m)	TIIDQHEK
☑	440	983.9833	1965.9520	1965.9465	2.80	1	14	12	1		841s (14.02m)	FGTEELARIEGEMLEAR + Oxidation (M)
☑	298	667.3715	1332.7284	1332.7071	16.0	0	14	16	1		1060s (17.67m)	TLDLAMANSLLR + Oxidation (M)
☑	198	569.7975	1137.5804	1137.5787	1.53	1	14	14	1		609s (10.15m)	GAVFMAVCRGK
☑	297	667.3715	1332.7284	1332.7071	16.0	0	14	17	1		1057s (17.62m)	TLDLAMANSLLR + Oxidation (M)
☑	137	509.3016	1016.5887	1016.6018	-12.91	2	14	13	1		582s (9.70m)	FVAPEKAKK
☑	335	475.9223	1424.7450	1424.7273	12.5	1	14	14	1		503s (8.38m)	AFNRSSHITQHK
☑	21	385.2040	768.3935	768.3878	7.35	1	14	7.5	1		524s (8.73m)	EKAHER
☑	103	478.3148	954.6151	954.5974	18.5	1	14	5.9	1		1017s (16.95m)	RAVEVLLR
☑	37	412.7099	823.4053	823.4188	-16.44	0	14	9.4	1		512s (8.53m)	LSSNFTR
☑	126	501.7966	1001.5787	1001.5869	-8.22	1	14	17	1		626s (10.43m)	VNVTGISKGK
☑	365	776.8352	1551.6558	1551.6776	-14.09	0	13	2.1	1		710s (11.83m)	HDMGGDIAPYFASR + Oxidation (M)
☑	341	730.9155	1459.8164	1459.8181	-1.14	1	13	15	1		577s (9.62m)	VVLRANVISGMSSK
☑	192	565.2679	1128.5213	1128.5299	-7.64	0	13	5.8	1		612s (10.20m)	LNDDVPDDVK
☑	371	783.8749	1565.7352	1565.7243	6.94	0	13	11	1		581s (9.68m)	GCPVTAATSDLTSESK
☑	92	467.7321	933.4496	933.4628	-14.12	2	13	13	1		601s (10.02m)	QRNTSSKN
☑	57	425.7617	849.5089	849.4960	15.2	1	13	8.7	1		432s (7.20m)	KIIEYGK
☑	45	421.2588	840.5031	840.4930	12.0	1	13	7.6	1		786s (13.10m)	RNVSLPR
☑	256	629.3345	1256.6544	1256.6724	-14.35	1	13	19	1		847s (14.12m)	AEAQIAGKNLDK
☑	343	735.8637	1469.7128	1469.6867	17.7	2	13	15	1		662s (11.03m)	DYMQNLGCRARK + Oxidation (M)
☑	136	508.2918	1014.5690	1014.5822	-13.03	0	13	26	1		658s (10.97m)	TASVLVGAAAR
☑	130	503.7657	1005.5169	1005.4979	19.0	0	13	24	1		628s (10.47m)	TSVTADDLGK
☑	170	536.3255	1070.6365	1070.6448	-7.70	1	13	19	1		559s (9.32m)	LDQSIRIVK
☑	84	901.9678	1801.9210	1801.9243	-1.86	1	12	23	1		785s (13.08m)	EILLQEEEKMIQQR + Oxidation (M)
☑	366	776.8352	1551.6558	1551.6776	-14.09	0	12	2.7	1		706s (11.77m)	HDMGGDIAPYFASR + Oxidation (M)
☑	257	629.3345	1256.6544	1256.6612	-5.43	2	12	22	1		844s (14.07m)	EKEDDKVLPGK

☑	169	536.3255	1070.6365	1070.6448	-7.70	1	12	20	1	562s (9.37m)	LDQSIRIVK
☑	163	531.7971	1061.5795	1061.5982	-17.53	1	12	25	1	1040s (17.33m)	ARDVLYLGR
☑	281	655.3251	1308.6357	1308.6534	-13.55	1	12	18	1	590s (9.83m)	EQHRIDEVQR
☑	217	585.8465	1169.6784	1169.6656	11.0	0	12	18	1	678s (11.30m)	TLGLSPLVNEK
☑	413	896.9052	1791.7959	1791.7945	0.78	0	12	5.7	1	880s (14.67m)	EVDGGDGGCISLEDLASR
☑	195	567.8315	1133.6484	1133.6366	10.4	0	12	15	1	783s (13.05m)	LTLLGIETMK + Oxidation (M)
☑	77	443.7657	885.5169	885.5171	-0.22	0	12	29	1	702s (11.70m)	TIELVSPK
☑	439	983.9833	1965.9520	1965.9465	2.80	1	12	19	1	844s (14.07m)	FGTEELARIEGEMLEAR + Oxidation (M)
☑	115	492.2608	982.5071	982.5157	-8.81	1	12	15	1	479s (7.98m)	DVLMFSKK + Oxidation (M)
☑	235	606.3120	1210.6095	1210.6049	3.75	1	12	20	1	607s (10.12m)	VSKAMMANLSK + 2 Oxidation (M)
☑	357	758.4117	1514.8088	1514.7940	9.75	1	12	28	1	513s (8.55m)	LLSQEIKDVNNDK
☑	212	582.8146	1163.6146	1163.6299	-13.12	0	12	31	1	692s (11.53m)	NEVINTLHPK
☑	133	506.2703	1010.5260	1010.5331	-7.07	0	12	19	1	569s (9.48m)	MLIDHLNR
☑	332	473.9195	1418.7367	1418.7486	-8.36	1	11	32	1	502s (8.37m)	AVTLMAMRQNIR + Oxidation (M)
☑	424	617.6559	1849.9459	1849.9534	-4.05	2	11	30	1	460s (7.67m)	QGLTGPPGPDGIKGEKGDK
☑	70	435.7559	869.4972	869.5123	-17.35	0	11	12	1	684s (11.40m)	WIVSPLR
☑	225	592.8344	1183.6543	1183.6574	-2.59	1	11	21	1	579s (9.65m)	TAVHFNLARR
☑	411	890.9053	1779.7960	1779.8057	-5.44	2	11	7.6	1	768s (12.80m)	VMRQASVDDSREEDK + Oxidation (M)
☑	179	550.2351	1098.4555	1098.4724	-15.31	1	11	2.9	1	476s (7.93m)	SSSMADRSSR + Oxidation (M)
☑	370	783.8749	1565.7352	1565.7243	6.94	0	11	18	1	584s (9.73m)	GCPVTAATSDLTSESK
☑	200	575.7981	1149.5816	1149.5852	-3.16	1	11	30	1	609s (10.15m)	MLKVWNTDK + Oxidation (M)
☑	199	574.8122	1147.6098	1147.6237	-12.17	0	11	36	1	445s (7.42m)	VTVFNLVDNK
☑	44	421.2588	840.5031	840.4930	12.0	1	11	12	1	783s (13.05m)	GRGVTPVR
☑	292	663.3471	1324.6797	1324.6776	1.59	0	11	28	1	712s (11.87m)	TDALGVAQFVPAH
☑	18	536.7870	1071.5595	1071.5672	-7.24	0	11	33	1	509s (8.48m)	LNSPATLNSR
☑	274	434.5847	1300.7322	1300.7292	2.36	0	11	32	1	548s (9.13m)	YGLIYHAPLVR
☑	386	811.9103	1621.8060	1621.8060	0.00	0	11	34	1	952s (15.87m)	YVGGGLNQSVESASVR
☑	188	562.7823	1123.5501	1123.5397	9.27	1	10	26	1	670s (11.17m)	YLDEAEKEK
☑	344	735.8640	1469.7134	1469.6867	18.2	2	10	27	1	666s (11.10m)	DYMQNLGCRARK + Oxidation (M)
☑	189	562.7823	1123.5501	1123.5695	-17.32	1	10	27	1	672s (11.20m)	ALGAYGKMGEK
☑	135	508.2918	1014.5690	1014.5709	-1.94	0	10	48	1	655s (10.92m)	TSIIELSPR
☑	122	496.7987	991.5829	991.5927	-9.82	1	10	17	1	440s (7.33m)	AARIVPHTK
☑	162	529.7798	1057.5451	1057.5404	4.51	0	10	35	1	500s (8.33m)	ITEIENPSR
☑	434	970.9733	1939.9321	1939.9422	-5.20	2	10	27	1	765s (12.75m)	SFSSAVKNELCRVETDR
☑	405	857.4108	1712.8071	1712.8113	-2.44	0	10	23	1	735s (12.25m)	MAGMQLDEIIEYLR + 2 Oxidation (M)
☑	203	576.7980	1151.5815	1151.5710	9.16	0	10	33	1	682s (11.37m)	SPEELYSSLK
☑	320	695.8493	1389.6840	1389.6697	10.3	0	10	37	1	787s (13.12m)	VDLIEPESDMVK + Oxidation (M)
☑	391	819.9054	1637.7963	1637.7859	6.40	0	10	35	1	877s (14.62m)	DTGIETLFLDIPTGM + Oxidation (M)
☑	418	901.9717	1801.9288	1801.9243	2.45	1	10	47	1	788s (13.13m)	EILLQEEEKMIQQR + Oxidation (M)
☑	443	994.0412	1986.0679	1986.0494	9.31	2	10	37	1	1122s (18.70m)	DNLRKSSAVLVDGELQSR
☑	117	492.2879	982.5613	982.5488	12.8	0	10	16	1	907s (15.12m)	TYFLKPSK
☑	94	467.7329	933.4512	933.4637	-13.33	1	9	30	1	829s (13.82m)	MMGRNGIR
☑	35	404.2380	806.4614	806.4572	5.23	0	9	31	1	505s (8.42m)	ILLMSSK + Oxidation (M)
☑	436	647.6515	1939.9327	1939.9422	-4.87	2	9	33	1	775s (12.92m)	SFSSAVKNELCRVETDR
☑	153	522.8272	1043.6399	1043.6379	1.91	0	9	18	1	741s (12.35m)	AILPIIYNK
☑	404	857.4108	1712.8071	1712.8113	-2.44	0	9	29	1	738s (12.30m)	MAGMQLDEIIEYLR + 2 Oxidation (M)
☑	216	585.3309	1168.6473	1168.6676	-17.36	2	9	36	1	731s (12.18m)	RSIPKLDANR
☑	223	590.2941	1178.5737	1178.5680	4.85	0	9	41	1	469s (7.82m)	GTDFNIVNGSR
☑	416	897.9148	1793.8151	1793.7924	12.7	0	9	17	1	733s (12.22m)	DGMQEEAIQEIAGMTR + Oxidation (M)
☑	427	930.4795	1858.9444	1858.9472	-1.48	1	9	56	1	891s (14.85m)	HFCASQKSSLLQLQNR
☑	131	505.2521	1008.4896	1008.4876	1.97	0	9	38	1	468s (7.80m)	GTYNDNVVK
☑	406	857.4448	1712.8751	1712.9032	-16.42	1	9	48	1	639s (10.65m)	SLPLRHGDVVTFMNK
☑	33	404.2380	806.4614	806.4650	-4.52	0	9	35	1	508s (8.47m)	LAATYLR
☑	401	849.4476	1696.8806	1696.9009	-11.95	0	9	47	1	636s (10.60m)	VLGAHQGIANYTIGQR
☑	431	936.4703	1870.9261	1870.9247	0.75	2	9	45	1	1105s (18.42m)	MEEFQVYLELDRKR + Oxidation (M)
☑	236	606.3120	1210.6095	1210.6016	6.53	0	9	41	1	603s (10.05m)	ILAPYSMSASR + Oxidation (M)
☑	453	742.7099	2225.1078	2225.1263	-8.31	2	8	52	1	713s (11.88m)	LPYMGQSLNNAGSKSFLDKR
☑	151	522.7780	1043.5415	1043.5611	-18.78	0	8	54	1	598s (9.97m)	ALDAGQTLQK
☑	107	483.2731	964.5316	964.5164	15.7	0	8	45	1	499s (8.32m)	VATFAMAVR
☑	114	489.2594	976.5042	976.4899	14.6	0	8	66	1	589s (9.82m)	EVDNMIIK + Oxidation (M)
☑	56	425.7617	849.5089	849.4960	15.2	1	8	27	1	435s (7.25m)	KIIEYGK
☑	394	820.4201	1638.8256	1638.8552	-18.04	0	8	59	1	587s (9.78m)	VPAIMVTGSHIPFDR
☑	184	560.2983	1118.5821	1118.5680	12.7	2	8	69	1	1002s (16.70m)	SKGKNTNNEK
☑	210	582.2846	1162.5546	1162.5441	9.04	0	8	41	1	752s (12.53m)	QMFVTQHGLS + Oxidation (M)
☑	27	792.8523	1583.6901	1583.7137	-14.94	0	8	13	1	697s (11.62m)	SLGLSADQMADEFGK + Oxidation (M)
☑	205	577.2918	1152.5690	1152.5663	2.36	0	8	44	1	954s (15.90m)	ESDSVFVVSGK
☑	233	603.3256	1204.6366	1204.6373	-0.62	0	8	76	1	694s (11.57m)	TITMSDPALLK + Oxidation (M)
☑	234	603.3256	1204.6366	1204.6339	2.19	0	8	78	1	696s (11.60m)	DAVPSIYEALK
☑	264	639.3063	1276.5980	1276.6081	-7.90	1	8	31	1	784s (13.07m)	RALSDAGMTPDK + Oxidation (M)
☑	109	487.3004	972.5863	972.5678	19.0	1	8	38	1	879s (14.65m)	EKLPMVLK + Oxidation (M)
☑	321	695.8493	1389.6840	1389.6632	15.0	0	8	58	1	791s (13.18m)	MPTMPLEAELAR + 2 Oxidation (M)
☑	430	936.4703	1870.9261	1870.9611	-18.72	1	8	56	1	1108s (18.47m)	FTVITGPNMSGKSTFIR + Oxidation (M)
☑	387	812.3984	1622.7822	1622.7796	1.56	0	8	50	1	904s (15.07m)	SWLEDMTLMIVR + Oxidation (M)
☑	247	624.8529	1247.6913	1247.7138	-18.06	2	8	55	1	823s (13.72m)	KAKNLIYWGR
☑	402	849.9425	1697.8704	1697.8808	-6.16	2	8	60	1	643s (10.72m)	RRAVAAPATTAAEEER
☑	319	691.3695	1380.7244	1380.7071	12.5	1	8	62	1	600s (10.00m)	LYINCAKDINSK
☑	342	730.9155	1459.8164	1459.8181	-1.14	1	8	57	1	573s (9.55m)	VVLRANVISGMSSK
☑	17	529.2629	1056.5112	1056.5240	-12.06	0	8	86	1	616s (10.27m)	AEPANPYAPK
☑	1	382.2197	762.4248	762.4137	14.6	1	7	1.5e+02	1	718s (11.97m)	TYPARR
☑	96	467.7367	933.4588	933.4477	11.9	0	7	62	1	498s (8.30m)	AIMEELASA
☑	129	503.7657	1005.5169	1005.5091	7.79	0	7	87	1	631s (10.52m)	TGSLTDLSGR
☑	229	596.8132	1191.6118	1191.5992	10.6	0	7	81	1	673s (11.22m)	MLLGMATPSAGK + Oxidation (M)
☑	121	496.7987	991.5829	991.5927	-9.82	1	7	34	1	444s (7.40m)	AARIVPHTK
☑	417	599.2769	1794.8090	1794.8383	-16.36	2	7	25	1	778s (12.97m)	NYLEEKKEEEGSANR
☑	351	752.8699	1503.7252	1503.7351	-6.58	2	7	59	1	817s (13.62m)	DVKRQDLEQMDK
☑	392	819.9067	1637.7988	1637.7859	7.92	0	7	69	1	851s (14.18m)	DTGIETLFLDIPTGM + Oxidation (M)

☑	248	624.8529	1247.6913	1247.7098	-14.85	1	7	71	1	827s (13.78m)	AKNGHLSVVAPR
☑	206	577.8121	1153.6097	1153.6053	3.83	0	7	61	1	685s (11.42m)	IAEFLMTTTK
☑	265	639.3063	1276.5980	1276.6160	-14.05	1	7	42	1	787s (13.12m)	AAGQNARGEFEK
☑	252	627.8336	1253.6526	1253.6476	3.96	2	6	74	1	656s (10.93m)	ARHSGTSEGPKK
☑	36	811.9091	1621.8036	1621.7947	5.48	2	6	88	1	956s (15.93m)	SEKLSSSWADDAAKK
☑	437	647.6515	1939.9327	1939.9639	-16.10	1	6	66	1	772s (12.87m)	GNEYEALTSPQTSFRLK
☑	393	819.9166	1637.8187	1637.8453	-16.25	0	6	88	1	590s (9.83m)	VEALGPYLNFSFGPK
☑	66	432.7490	863.4834	863.4977	-16.59	1	6	83	1	532s (8.87m)	RLASYVR
☑	258	630.8534	1259.6922	1259.7094	-13.60	1	6	94	1	1026s (17.10m)	KMPIGVMVTLR + Oxidation (M)
☑	83	897.9162	1793.8178	1793.7924	14.2	0	6	34	1	736s (12.27m)	DGMQEEAIQEIAGMTR + Oxidation (M)
☑	134	507.2786	1012.5426	1012.5553	-12.56	0	6	55	1	565s (9.42m)	QTVGPDIGVK
☑	444	994.5373	1987.0600	1987.0837	-11.94	2	6	93	1	1112s (18.53m)	ESEKGILIDIEVTTNAKK
☑	433	961.9674	1921.9201	1921.9204	-0.11	0	6	74	1	1099s (18.32m)	RPSLDSMENQVSVDAFK
☑	383	804.9026	1607.7906	1607.7903	0.16	2	6	90	1	938s (15.63m)	KDDYQLNDRNVTK
☑	306	451.9341	1352.7804	1352.7704	7.38	0	6	52	1	1013s (16.88m)	TYLAVTYGKPIK
☑	355	756.3670	1510.7194	1510.7384	-12.61	2	6	71	1	686s (11.43m)	IPLSRCERYGSCK
☑	9	467.7320	933.4494	933.4590	-10.21	0	6	72	1	826s (13.77m)	NTMNLTPK + Oxidation (M)
☑	432	961.9674	1921.9201	1921.9204	-0.11	0	6	78	1	1095s (18.25m)	RPSLDSMENQVSVDAFK
☑	412	896.9052	1791.7959	1791.7945	0.78	0	6	26	1	884s (14.73m)	EVDGGDGGCISLEDLASR
☑	462	762.0639	2283.1698	2283.1706	-0.34	1	6	98	1	944s (15.73m)	SERNQEVIDDLQAELANVLK
☑	426	927.9573	1853.8999	1853.8777	12.0	2	6	82	1	1097s (18.28m)	VEAMLHRREGPDCWR
☑	138	509.8050	1017.5955	1017.5971	-1.54	1	6	73	1	763s (12.72m)	AGGGKYKPLK
☑	325	466.6099	1396.8078	1396.8190	-8.06	2	6	56	1	1037s (17.28m)	QQAKLGWKLIVN
☑	132	505.2521	1008.4896	1008.4737	15.8	1	5	83	1	465s (7.75m)	YTGGQGRDR
☑	334	711.8580	1421.7014	1421.7072	-4.06	1	5	98	1	858s (14.30m)	GESMDVVAETIKK + Oxidation (M)
☑	75	443.2729	884.5312	884.5192	13.6	2	5	1e+02	1	816s (13.60m)	QSAKKAPR
☑	159	526.2975	1050.5804	1050.5683	11.5	1	5	76	1	761s (12.68m)	QVENLHRR
☑	352	752.8699	1503.7252	1503.7352	-6.60	0	5	84	1	819s (13.65m)	CQLTGTGAETTVPAR
☑	318	691.3695	1380.7244	1380.7071	12.5	1	5	1.1e+02	1	603s (10.05m)	LYINCAKDINSK
☑	435	970.9733	1939.9321	1939.9428	-5.53	1	5	85	1	769s (12.82m)	KFQESNSEILWSPYGR
☑	204	577.2918	1152.5690	1152.5743	-4.61	2	5	90	1	959s (15.98m)	TDMRKMLSR + Oxidation (M)
☑	367	777.3243	1552.6341	1552.6576	-15.15	0	5	6.5	1	652s (10.87m)	DGSQMSGENQPTFR
☑	293	663.8974	1325.7802	1325.7680	9.23	2	5	70	1	937s (15.62m)	LPKRGFNKPNR
☑	446	1042.0125	2082.0105	2082.0051	2.56	1	5	99	1	814s (13.57m)	NLSNFASLGSECTVDRELK
☑	253	627.8336	1253.6526	1253.6689	-13.02	2	5	1.1e+02	1	659s (10.98m)	MLSDYKEKIK
☑	454	742.7099	2225.1078	2225.1263	-8.31	2	5	1.2e+02	1	716s (11.93m)	LPYMGQSLNNAGSKSFLDKR
☑	239	608.2639	1214.5131	1214.5134	-0.19	0	5	14	1	466s (7.77m)	FMMNPTDLCK + Oxidation (M)
☑	421	913.9831	1825.9516	1825.9210	16.8	1	5	1.2e+02	1	739s (12.32m)	IKLESSAGTGHFYTTSK
☑	14	484.7299	967.4452	967.4433	1.94	0	5	48	1	512s (8.53m)	QNMDLGFK + Oxidation (M)
☑	254	628.3843	1254.7540	1254.7408	10.6	1	5	75	1	934s (15.57m)	VVTAALEIQRR
☑	201	575.8445	1149.6744	1149.6618	10.9	0	5	42	1	834s (13.90m)	TGGLHAAVLVGR
☑	324	699.4106	1396.8067	1396.7813	18.2	0	5	71	1	996s (16.60m)	DLPEAEIVSLAIK
☑	308	451.9341	1352.7804	1352.7564	17.7	2	4	70	1	999s (16.65m)	NHEIKPKKVFN
☑	300	446.8612	1337.5618	1337.5843	-16.80	0	4	14	1	434s (7.23m)	EDISLCALECDK
☑	142	514.2993	1026.5840	1026.5750	8.81	2	4	89	1	939s (15.65m)	TYKLKDLF
☑	328	466.6099	1396.8078	1396.8190	-8.06	2	4	73	1	1033s (17.22m)	QQAKLGWKLIVN
☑	30	401.7190	801.4235	801.4344	-13.63	0	4	2.1e+02	1	508s (8.47m)	EAGVISAR
☑	295	665.2960	1328.5775	1328.5844	-5.16	1	4	27	1	555s (9.25m)	EDAAPRAEEAAAE
☑	267	641.8846	1281.7547	1281.7292	19.9	1	4	61	1	913s (15.22m)	EIIEGKDLPIR
☑	313	683.7954	1365.5763	1365.5718	3.31	0	4	18	1	985s (16.42m)	QICESDQVDSDK
☑	15	497.2903	992.5659	992.5767	-10.84	0	4	1.7e+02	1	620s (10.33m)	VAEIGLVHR
☑	74	443.2729	884.5312	884.5192	13.6	2	4	1.4e+02	1	813s (13.55m)	QSAKKAPR
☑	307	451.9341	1352.7804	1352.7564	17.7	2	4	77	1	1006s (16.77m)	NHEIKPKKVFN
☑	3	391.2269	1170.6588	1170.6509	6.71	0	4	2.6e+02	1	657s (10.95m)	AAGPVHLHLEK
☑	390	542.6332	1624.8779	1624.8719	3.67	0	4	1.2e+02	1	1136s (18.93m)	MQQRPIGPLVDSLR + Oxidation (M)
☑	420	913.9831	1825.9516	1825.9210	16.8	1	4	1.5e+02	1	742s (12.37m)	IKLESSAGTGHFYTTSK
☑	144	1030.9648	2059.9151	2059.9447	-14.36	0	4	46	1	610s (10.17m)	TSFEEGDAVNITANPNPER
☑	397	827.8914	1653.7683	1653.7953	-16.34	2	4	84	1	784s (13.07m)	ELEAEGKKLTMDMK + 2 Oxidation (M)
☑	410	890.9050	1779.7954	1779.8057	-5.80	2	4	43	1	765s (12.75m)	VMRQASVDDSREEDK + Oxidation (M)
☑	118	492.2879	982.5613	982.5488	12.8	0	4	63	1	909s (15.15m)	TYFLKPSK
☑	91	465.7788	929.5431	929.5546	-12.34	1	4	1.9e+02	1	711s (11.85m)	KVVSATTPK
☑	90	465.7779	929.5413	929.5406	0.67	2	4	2e+02	1	728s (12.13m)	NKTARVGGK
☑	429	935.9569	1869.8993	1869.9108	-6.19	1	3	1.2e+02	1	840s (14.00m)	EVREFIDFTLSEETR
☑	448	705.0159	2112.0260	2112.0278	-0.85	2	3	1.4e+02	1	914s (15.23m)	AAFMKAMGESGLRTINEMR
☑	425	927.9573	1853.8999	1853.9345	-18.66	1	3	1.4e+02	1	1102s (18.37m)	IFAVADSMEPYALRQK + Oxidation (M)
☑	388	812.8994	1623.7841	1623.8145	-18.66	1	3	1.3e+02	1	836s (13.93m)	TLPTQGIDDFFKDK
☑	43	420.7281	839.4416	839.4575	-18.94	1	3	1e+02	1	1457s (24.28m)	AMSLFKK + Oxidation (M)
☑	16	527.7454	1053.4763	1053.4767	-0.37	0	3	62	1	790s (13.17m)	SEGVEYWGK
☑	461	762.0639	2283.1698	2283.1304	17.3	2	3	1.7e+02	1	940s (15.67m)	EDIKEISMSDLKDFLESIR + Oxidation (M)
☑	251	625.8230	1249.6314	1249.6415	-8.08	1	3	1.9e+02	1	470s (7.83m)	YTRDEAAGLVR
☑	140	512.2415	1022.4685	1022.4637	4.67	0	3	1e+02	1	497s (8.28m)	AAQMMGINR + 2 Oxidation (M)
☑	141	512.2415	1022.4685	1022.4637	4.67	0	3	1e+02	1	493s (8.22m)	AAQMMGINR + 2 Oxidation (M)
☑	278	654.3078	1306.6011	1306.6187	-13.50	0	3	84	1	474s (7.90m)	GMQAVGSANLTDK + Oxidation (M)
☑	190	564.2790	1126.5434	1126.5659	-19.89	1	3	99	1	513s (8.55m)	IIYKTFNDN
☑	338	721.4239	1440.8331	1440.8201	9.05	2	3	1.3e+02	1	1051s (17.52m)	YTVLRSPHVNKK
☑	285	437.2584	1308.7532	1308.7514	1.42	1	3	78	1	969s (16.15m)	HSKKPSTLGINK
☑	348	495.9605	1484.8597	1484.8787	-12.80	2	3	1.2e+02	1	1078s (17.97m)	KGRLVAVTGSIQTR
☑	310	451.9341	1352.7804	1352.7564	17.7	2	3	1e+02	1	1009s (16.82m)	NHEIKPKKVFN
☑	377	792.8498	1583.6851	1583.6555	18.7	1	3	40	1	700s (11.67m)	RNTESQPESMDMK + 2 Oxidation (M)
☑	466	894.1380	2679.3921	2679.4021	-3.71	1	3	1.7e+02	1	1787s (29.78m)	GKHKPVYTPHVDTGDYLVVINAEK
☑	76	443.7657	885.5169	885.5283	-12.88	1	3	2.6e+02	1	705s (11.75m)	DKALQAIK
☑	161	529.7500	1057.4854	1057.4975	-11.42	0	3	60	1	629s (10.48m)	MTIADHQAR + Oxidation (M)
☑	59	426.2478	850.4810	850.4661	17.5	1	3	97	1	777s (12.95m)	KNGIYTR
☑	176	549.7404	1097.4662	1097.4812	-13.58	0	3	37	1	472s (7.87m)	EGSGMVEFSR
☑	347	495.9602	1484.8589	1484.8787	-13.32	2	3	1.2e+02	1	1073s (17.88m)	KGRLVAVTGSIQTR
☑	266	641.8846	1281.7547	1281.7405	11.1	2	3	91	1	917s (15.28m)	SKTHPAALTTKK

93	467.7321	933.4496	933.4589	-10.00	1	2	1.5e+02	1	608s (10.13m)	CKEIEGQK
354	755.3300	1508.6455	1508.6756	-19.99	1	2	36	1	784s (13.07m)	RDDIGHDPEWNR
73	440.7348	879.4551	879.4450	11.4	1	2	2.1e+02	1	532s (8.87m)	KAFDANSK
95	467.7367	933.4588	933.4589	-0.12	0	2	2e+02	1	502s (8.37m)	ALGMELER + Oxidation (M)
157	523.3053	1044.5961	1044.5815	14.0	1	2	2.2e+02	1	786s (13.10m)	KASEGVVEVK
150	522.7780	1043.5415	1043.5611	-18.78	0	2	2.4e+02	1	602s (10.03m)	ALDAGQTLQK
4	393.2125	1176.6156	1176.6260	-8.81	2	2	4.2e+02	1	754s (12.57m)	RKHFMLCVK + Oxidation (M)
336	475.9223	1424.7450	1424.7524	-5.19	1	2	2.1e+02	1	499s (8.32m)	LPDRDAVAGWVAR
87	456.7734	911.5322	911.5480	-17.32	1	2	93	1	846s (14.10m)	TYFIKIK
389	542.6332	1624.8779	1624.8719	3.67	0	2	2e+02	1	1140s (19.00m)	MQQRPIGPLVDSLR + Oxidation (M)
65	431.2629	860.5112	860.5153	-4.84	1	2	1.7e+02	1	436s (7.27m)	KPKTLMK + Oxidation (M)
89	457.2642	912.5139	912.5029	12.1	0	2	1.4e+02	1	709s (11.82m)	TPQQKPSK
464	873.3561	2617.0466	2617.0407	2.24	0	2	5	1	449s (7.48m)	HMDDIISGEFSSGMMADWTNDDK + Oxidation (M)
160	529.7500	1057.4854	1057.4975	-11.42	0	2	76	1	632s (10.53m)	MTIADHQAR + Oxidation (M)
428	935.9569	1869.8993	1869.9366	-19.99	1	2	1.9e+02	1	838s (13.97m)	LPEMQERTRPEAIER + Oxidation (M)
191	564.2790	1126.5434	1126.5506	-6.35	0	1	1.4e+02	1	515s (8.58m)	IDKPEADPDK
315	685.9094	1369.8042	1369.7817	16.4	0	1	1.6e+02	1	975s (16.25m)	ELVSLGVEGAIVGK
88	457.2642	912.5139	912.5029	12.1	0	1	1.5e+02	1	705s (11.75m)	QLVAGNSPK
31	404.2337	806.4528	806.4684	-19.31	2	1	2.3e+02	1	751s (12.52m)	KSVAMKK + Oxidation (M)
178	549.7610	1097.5074	1097.5283	-19.01	1	1	1.2e+02	1	822s (13.70m)	MMMVELKGK + 2 Oxidation (M)
309	451.9341	1352.7804	1352.7928	-9.21	2	1	1.4e+02	1	995s (16.58m)	YTYLAKAVLRR
209	582.2846	1162.5546	1162.5540	0.57	0	1	1.9e+02	1	755s (12.58m)	DANELVEMVK + Oxidation (M)
311	451.9341	1352.7804	1352.7776	2.07	2	1	1.5e+02	1	1001s (16.68m)	NILPNKSKNIVN
58	426.2471	850.4795	850.4800	-0.54	0	1	1.4e+02	1	812s (13.53m)	TYVLDLK
42	413.2387	824.4628	824.4617	1.40	2	1	1.2e+02	1	681s (11.35m)	RAKSIHN
164	531.8181	1061.6216	1061.6233	-1.55	2	1	1.5e+02	1	786s (13.10m)	YTPKIAKNK
202	575.8445	1149.6744	1149.6618	10.9	0	1	92	1	830s (13.83m)	TGGLHAAVLVGR
177	549.7610	1097.5074	1097.5175	-9.19	0	1	1.3e+02	1	816s (13.60m)	MSASAQVNYK
123	500.7980	999.5815	999.5713	10.2	2	1	3e+02	1	911s (15.18m)	AGKALKEAVN
294	663.8974	1325.7802	1325.8031	-17.20	2	1	1.7e+02	1	940s (15.67m)	IIDALTGKIIRN
449	427.8712	2134.3195	2134.2837	16.8	2	1	5.9	1	531s (8.85m)	TAVAPIERVKLLLQVQAASK
362	768.3214	1534.6282	1534.6495	-13.89	1	1	20	1	900s (15.00m)	ENNETDNNEKSNK
245	619.8714	1237.7282	1237.7282	0.00	0	1	1.1e+02	1	883s (14.72m)	LLPQLTESLPK
359	763.8618	1525.7090	1525.6905	12.1	1	1	1.6e+02	1	824s (13.73m)	NTPEQMKQMEFK + Oxidation (M)
260	633.3713	1264.7281	1264.7252	2.32	1	1	1.3e+02	1	899s (14.98m)	LVQTHGLKQNK
467	927.3747	2779.1022	2779.1402	-13.67	2	1	4.4	1	453s (7.55m)	DESCYDNAEAAFSDDEEDLNSKGKK
82	448.2605	894.5064	894.4923	15.8	0	1	1.5e+02	1	813s (13.55m)	EVIAPGGPR
246	619.8714	1237.7282	1237.7142	11.3	1	1	1.2e+02	1	886s (14.77m)	ELLNLDRLPR
80	448.2158	894.4171	894.4117	6.10	0	1	1.7e+02	1	529s (8.82m)	TEAASMAAK + Oxidation (M)
25	392.7124	783.4102	783.4239	-17.39	0	1	1.1e+02	1	511s (8.52m)	APQALER
452	741.0486	2220.1239	2220.1208	1.38	1	1	3.3e+02	1	937s (15.62m)	LGVERSQFLDLMALAGDSGNK
286	437.2584	1308.7532	1308.7514	1.41	2	1	1.4e+02	1	981s (16.35m)	SHQGSVKLKVNL
11	472.2864	942.5583	942.5498	8.95	0	0	5.2e+02	1	924s (15.40m)	TIVVQVER
174	545.3184	1088.6222	1088.6342	-11.08	2	0	3.2e+02	1	803s (13.38m)	KAFVAVVKNGG
20	382.2195	762.4245	762.4137	14.2	1	0	3.4e+02	1	722s (12.03m)	TYPARR
111	487.7918	973.5690	973.5669	2.22	1	0	2.5e+02	1	741s (12.35m)	LVSATATRR
323	699.4106	1396.8067	1396.7827	17.2	1	0	1.9e+02	1	993s (16.55m)	LDAIRFIVVTHN
105	479.2876	956.5607	956.5654	-4.95	1	0	3.7e+02	1	875s (14.58m)	ELLSRILN
262	635.8046	1269.5947	1269.5949	-0.16	0	0	1.7e+02	1	464s (7.73m)	VQSHQQASEEK
263	635.8046	1269.5947	1269.5949	-0.16	0	0	1.7e+02	1	461s (7.68m)	VQSHQQASEEK
326	466.6099	1396.8078	1396.7827	18.0	1	0	1.9e+02	1	1020s (17.00m)	LDAIRFIVVTHN
250	625.8075	1249.6005	1249.5860	11.6	0	0	2.7e+02	1	515s (8.58m)	SCVNEITIDEK
372	785.3127	1568.6109	1568.6187	-4.96	1	0	7.1	1	898s (14.97m)	TGSSGGSDKSSGDGEQN
32	404.2337	806.4528	806.4399	16.0	1	0	3.1e+02	1	748s (12.47m)	LPKAAHGN
2	391.2253	390.2180							654s (10.90m)	
7	412.7475	411.7402							782s (13.03m)	
8	461.8119	460.8046							1522s (25.37m)	
10	468.7235	467.7162							496s (8.27m)	
13	477.8243	476.8170							588s (9.80m)	
22	385.2559	768.4972							475s (7.92m)	
23	390.7338	779.4530							753s (12.55m)	
24	390.7338	779.4530							750s (12.50m)	
26	393.2165	784.4183							508s (8.47m)	
28	399.2469	796.4793							751s (12.52m)	
29	399.2469	796.4793							754s (12.57m)	
38	412.7469	823.4793							783s (13.05m)	
41	413.2387	824.4628							684s (11.40m)	
55	425.2107	848.4069							1458s (24.30m)	
60	429.2138	856.4131							1451s (24.18m)	
61	429.7145	857.4144							1454s (24.23m)	
64	431.2629	860.5112							438s (7.30m)	
67	434.7604	867.5063							812s (13.53m)	
68	434.7604	867.5063							815s (13.58m)	
69	435.2525	868.4905							688s (11.47m)	
78	448.1143	894.2140							979s (16.32m)	
79	448.2158	894.4171							533s (8.88m)	
81	448.2601	894.5057							842s (14.03m)	
86	456.7734	911.5322							843s (14.05m)	
97	472.2885	942.5625							926s (15.43m)	
104	478.7850	955.5554							878s (14.63m)	
108	484.7296	967.4447							509s (8.48m)	
110	487.7918	973.5690							746s (12.43m)	
119	496.3050	990.5954							760s (12.67m)	
120	496.3050	990.5954							757s (12.62m)	
124	501.2916	1000.5686							755s (12.58m)	
125	501.2916	1000.5686							758s (12.63m)	

☑	128	502.8164	1003.6182	958s (15.97m)
☑	139	509.8050	1017.5955	759s (12.65m)
☑	143	514.2993	1026.5840	936s (15.60m)
☑	148	518.3179	1034.6212	761s (12.68m)
☑	149	518.3179	1034.6212	764s (12.73m)
☑	152	522.8118	1043.6091	939s (15.65m)
☑	156	523.3053	1044.5961	783s (13.05m)
☑	165	531.8181	1061.6216	790s (13.17m)
☑	172	540.3299	1078.6453	787s (13.12m)
☑	173	540.3301	1078.6456	795s (13.25m)
☑	175	545.3184	1088.6222	800s (13.33m)
☑	180	553.8317	1105.6488	803s (13.38m)
☑	181	553.8317	1105.6488	806s (13.43m)
☑	186	562.3458	1122.6771	814s (13.57m)
☑	187	562.3458	1122.6771	809s (13.48m)
☑	193	567.3314	1132.6482	876s (14.60m)
☑	194	567.3314	1132.6482	880s (14.67m)
☑	213	584.3580	1166.7015	846s (14.10m)
☑	214	584.3581	1166.7016	843s (14.05m)
☑	220	589.3448	1176.6750	854s (14.23m)
☑	221	589.3448	1176.6750	856s (14.27m)
☑	230	597.8585	1193.7024	869s (14.48m)
☑	231	597.8585	1193.7024	866s (14.43m)
☑	237	606.3720	1210.7294	864s (14.40m)
☑	238	606.3720	1210.7294	866s (14.43m)
☑	242	611.3576	1220.7007	882s (14.70m)
☑	243	611.3576	1220.7007	885s (14.75m)
☑	255	628.3843	1254.7541	936s (15.60m)
☑	261	633.3713	1264.7281	903s (15.05m)
☑	268	429.0897	1284.2473	528s (8.80m)
☑	270	650.3987	1298.7829	939s (15.65m)
☑	271	650.3994	1298.7842	920s (15.33m)
☑	272	650.7884	1299.5621	467s (7.78m)
☑	282	437.2583	1308.7532	974s (16.23m)
☑	283	437.2583	1308.7532	970s (16.17m)
☑	284	437.2583	1308.7532	977s (16.28m)
☑	287	437.2586	1308.7540	947s (15.78m)
☑	288	655.3847	1308.7548	931s (15.52m)
☑	289	655.3847	1308.7548	927s (15.45m)
☑	302	672.4103	1342.8061	974s (16.23m)
☑	303	672.4104	1342.8062	972s (16.20m)
☑	304	451.9338	1352.7797	971s (16.18m)
☑	305	677.3973	1352.7800	962s (16.03m)
☑	312	677.3975	1352.7804	955s (15.92m)
☑	314	683.7954	1365.5763	982s (16.37m)
☑	327	466.6099	1396.8078	1023s (17.05m)
☑	329	466.6099	1396.8078	1025s (17.08m)
☑	331	708.4247	1414.8349	1030s (17.17m)
☑	333	711.3583	1420.7020	861s (14.35m)
☑	337	721.4239	1440.8331	1047s (17.45m)
☑	339	729.6377	1457.2609	874s (14.57m)
☑	340	729.9381	1457.8616	1054s (17.57m)
☑	350	748.3085	1494.6025	689s (11.48m)
☑	353	755.3210	1508.6275	756s (12.60m)
☑	358	759.3680	1516.7215	943s (15.72m)
☑	360	763.8618	1525.7090	821s (13.68m)
☑	361	768.3193	1534.6239	965s (16.08m)
☑	363	771.3207	1540.6269	808s (13.47m)
☑	364	771.8260	1541.6374	810s (13.50m)
☑	373	785.3127	1568.6109	901s (15.02m)
☑	374	785.8759	1569.7372	574s (9.57m)
☑	375	788.2993	1574.5841	869s (14.48m)
☑	376	791.3096	1580.6047	870s (14.50m)
☑	378	795.7803	1589.5461	871s (14.52m)
☑	379	795.7803	1589.5461	867s (14.45m)
☑	384	805.3633	1608.7120	523s (8.72m)
☑	398	837.3642	1672.7139	467s (7.78m)
☑	422	616.5818	1846.7236	774s (12.90m)
☑	441	984.9445	1967.8744	752s (12.53m)
☑	442	984.9445	1967.8744	749s (12.48m)
☑	445	665.2901	1992.8484	767s (12.78m)
☑	447	1042.0125	2082.0105	811s (13.52m)
☑	450	427.8712	2134.3195	528s (8.80m)
☑	451	721.0452	2160.1139	510s (8.50m)
☑	455	746.0565	2235.1476	490s (8.17m)
☑	456	746.0565	2235.1476	494s (8.23m)
☑	463	427.6791	2560.0306	531s (8.85m)
☑	465	894.1380	2679.3921	1790s (29.83m)
☑	468	927.3747	2779.1022	449s (7.48m)
☑	469	981.3949	2941.1628	450s (7.50m)
☑	470	1046.8113	3137.4122	788s (13.13m)

Search Parameters

```
Type of search         : MS/MS Ion Search
Enzyme                 : Trypsin
```

```
Variable modifications : Oxidation (M)
Mass values            : Monoisotopic
Protein Mass           : Unrestricted
Peptide Mass Tolerance : ± 20 ppm
Fragment Mass Tolerance: ± 0.2 Da
Max Missed Cleavages   : 2
Instrument type        : ESI-QUAD-TOF
Number of queries      : 470
```

Mascot: http://www.matrixscience.com/

Mascot Search Results

Host : 10.10.10.100
User : alison@anatomy
Email :
Search title : CPFP SCH6549 [2/25] april9_2014_alison_af10.mgf
MS data file : april9_2014_alison_af10.mgf.mascot
Database : UNIPROT (26617536 sequences; 8639402574 residues)
Taxonomy : Homo sapiens (human) (132434 sequences)
Timestamp : 16 Apr 2014 at 23:57:28 GMT
Warning : There is an error in a configuration file: Error in the configuration file ../config/mascot.dat - database echidna2014small_
Protein hits : FCGBP_HUMAN RecName: Full=IgGFc-binding protein; AltName: Full=Fcgamma-binding protein antigen; Short=FcgammaBP; Flags: Pre
HEMO_HUMAN RecName: Full=Hemopexin; AltName: Full=Beta-1B-glycoprotein; Flags: Precursor; - OS=Homo sapiens (Human).
ACTA_HUMAN RecName: Full=Actin, aortic smooth muscle; AltName: Full=Alpha-actin-2; AltName: Full=Cell growth-inhibiting ge
A0N4V7_HUMAN SubName: Full=HCG2039797; SubName: Full=Possible J 56 gene segment; Flags: Fragment; - OS=Homo sapiens (Human).
K2C1_HUMAN RecName: Full=Keratin, type II cytoskeletal 1; AltName: Full=67 kDa cytokeratin; AltName: Full=Cytokeratin-1; S
B3KQZ8_HUMAN SubName: Full=cDNA FLJ33349 fis, clone BRACE2003905 {ECO:0000313|EMBL:BAG52210.1} OS=Homo sapiens (Human)
J3KSK6_HUMAN SubName: Full=Protein-methionine sulfoxide oxidase MICAL3; Flags: Fragment; - OS=Homo sapiens (Human).
H0Y4J2_HUMAN SubName: Full=39S ribosomal protein L37, mitochondrial {ECO:0000313|Ensembl:ENSP00000392216}; Flags: Fragment O
H0YEG8_HUMAN SubName: Full=Nucleobindin-2; Flags: Fragment; - OS=Homo sapiens (Human).
Q8N6K3_HUMAN SubName: Full=SNX25 protein; - OS=Homo sapiens (Human).
FETUA_HUMAN RecName: Full=Alpha-2-HS-glycoprotein; AltName: Full=Alpha-2-Z-globulin; AltName: Full=Ba-alpha-2-glycoprotein;
O10A7_HUMAN RecName: Full=Olfactory receptor 10A7; AltName: Full=Olfactory receptor OR12-6; - OS=Homo sapiens (Human).
B2R747_HUMAN SubName: Full=cDNA, FLJ93281, highly similar to Homo sapiens calcium/calmodulin-dependent protein kinase IV (CA
B4DX30_HUMAN SubName: Full=Long-chain-fatty-acid--CoA ligase 5; SubName: Full=cDNA FLJ52792, highly similar to Long-chain-fa
H0Y9B1_HUMAN SubName: Full=Interleukin-6 receptor subunit alpha; Flags: Fragment; - OS=Homo sapiens (Human).
CCAR1_HUMAN RecName: Full=Cell division cycle and apoptosis regulator protein 1; AltName: Full=Cell cycle and apoptosis reg
PLXA3_HUMAN RecName: Full=Plexin-A3; AltName: Full=Plexin-4; AltName: Full=Semaphorin receptor SEX; Flags: Precursor OS=Hom
E9PP40_HUMAN SubName: Full=Leucine-rich repeat-containing protein 14; Flags: Fragment; - OS=Homo sapiens (Human).
Q6UXV8_HUMAN SubName: Full=KCNQ2; - OS=Homo sapiens (Human).
GEMI5_HUMAN RecName: Full=Gem-associated protein 5; Short=Gemin5 OS=Homo sapiens (Human)

	UNIPROT	Decoy	False discovery rate
Peptide matches above identity threshold	13	2	15.38 %
Peptide matches above homology or identity threshold	16	9	56.25 %

Mascot Score Histogram

Ions score is -10*Log(P), where P is the probability that the observed match is a random event.
Individual ions scores > 29 indicate identity or extensive homology (p<0.05).
Protein scores are derived from ions scores as a non-probabilistic basis for ranking protein hits.

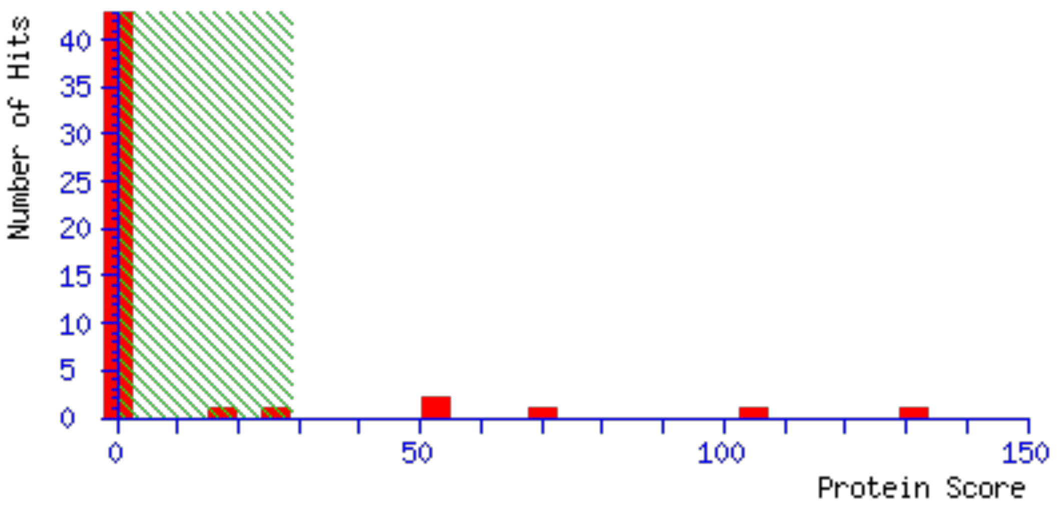

Peptide Summary Report

Format As	Peptide Summary		Help
	Significance threshold p< 0.05	Max. number of hits 20	Show Percolator scores
	Standard scoring MudPIT scoring	Ions score or expect cut-off 0	Show sub-sets 0
	Show pop-ups Suppress pop-ups	Sort unassigned Decreasing Score	Require bold red
	Preferred taxonomy All entries		

1. FCGBP_HUMAN **Mass:** 571639 **Score:** 131 **Matches:** 2(2) **Sequences:** 1(1) **emPAI:** 0.01
RecName: Full=IgGFc-binding protein; AltName: Full=Fcgamma-binding protein antigen; Short=FcgammaBP; Flags: Precursor; - OS=Homo sapiens
Check to include this hit in error tolerant search or archive report

Query	Observed	Mr(expt)	Mr(calc)	ppm	Miss	Score	Expect	Rank	Unique	Retention Time	Peptide
393	706.8766	1411.7387	1411.7347	2.81	0	(60)	6.7e-05	1	U	866s (14.43m)	R.YDLAFVVASQATK.L
394	706.8766	1411.7387	1411.7347	2.81	0	89	7.6e-08	1	U	869s (14.48m)	R.YDLAFVVASQATK.L

2. HEMO_HUMAN **Mass:** 51643 **Score:** 103 **Matches:** 4(4) **Sequences:** 2(2) **emPAI:** 0.13
RecName: Full=Hemopexin; AltName: Full=Beta-1B-glycoprotein; Flags: Precursor; - OS=Homo sapiens (Human).
Check to include this hit in error tolerant search or archive report

Query	Observed	Mr(expt)	Mr(calc)	ppm	Miss	Score	Expect	Rank	Unique	Retention Time	Peptide
234	571.3007	1140.5868	1140.5815	4.61	0	(46)	0.0015	1	U	618s (10.30m)	K.GGYTLVSGYPK.R
235	571.3007	1140.5868	1140.5815	4.61	0	47	0.0011	1	U	614s (10.23m)	K.GGYTLVSGYPK.R
272	610.8097	1219.6048	1219.5986	5.13	0	(42)	0.005	1	U	757s (12.62m)	K.NFPSPVDAAFR.Q
273	610.8097	1219.6048	1219.5986	5.13	0	42	0.0047	1	U	761s (12.68m)	K.NFPSPVDAAFR.Q

3. ACTA_HUMAN **Mass:** 41982 **Score:** 71 **Matches:** 2(2) **Sequences:** 2(2) **emPAI:** 0.16

RecName: Full=Actin, aortic smooth muscle; AltName: Full=Alpha-actin-2; AltName: Full=Cell growth-inhibiting gene 46 protein; Flags: Pre

☐ Check to include this hit in error tolerant search or archive report

	Query	Observed	Mr(expt)	Mr(calc)	ppm	Miss	Score	Expect	Rank	Unique	Retention Time	Peptide
☑	48	398.2378	794.4611	794.4650	-4.86	0	49	0.00041	1	U	529s (8.82m)	K.IIAPPER.K
☑	246	581.3126	1160.6107	1160.6111	-0.34	0	46	0.0021	1	U	612s (10.20m)	K.EITALAPSTMK.I

Proteins matching the same set of peptides:

ACTB_HUMAN **Mass:** 41710 **Score:** 71 **Matches:** 2(2) **Sequences:** 2(2)
RecName: Full=Actin, cytoplasmic 1; AltName: Full=Beta-actin; Contains: RecName: Full=Actin, cytoplasmic 1, N-terminally processed; - OS
ACTC_HUMAN **Mass:** 41992 **Score:** 71 **Matches:** 2(2) **Sequences:** 2(2)
RecName: Full=Actin, alpha cardiac muscle 1; AltName: Full=Alpha-cardiac actin; Flags: Precursor; - OS=Homo sapiens (Human).
ACTG_HUMAN **Mass:** 41766 **Score:** 71 **Matches:** 2(2) **Sequences:** 2(2)
RecName: Full=Actin, cytoplasmic 2; AltName: Full=Gamma-actin; Contains: RecName: Full=Actin, cytoplasmic 2, N-terminally processed; - O
ACTH_HUMAN **Mass:** 41850 **Score:** 71 **Matches:** 2(2) **Sequences:** 2(2)
RecName: Full=Actin, gamma-enteric smooth muscle; AltName: Full=Alpha-actin-3; AltName: Full=Gamma-2-actin; AltName: Full=Smooth muscle
ACTS_HUMAN **Mass:** 42024 **Score:** 71 **Matches:** 2(2) **Sequences:** 2(2)
RecName: Full=Actin, alpha skeletal muscle; AltName: Full=Alpha-actin-1; Flags: Precursor; - OS=Homo sapiens (Human).
F1BXA6_HUMAN **Mass:** 12188 **Score:** 71 **Matches:** 2(2) **Sequences:** 2(2)
SubName: Full=Beta-actin; Flags: Fragment; - OS=Homo sapiens (Human).
Q1KLZ0_HUMAN **Mass:** 41710 **Score:** 71 **Matches:** 2(2) **Sequences:** 2(2)
SubName: Full=HCG15971, isoform CRA_a; SubName: Full=PS1TP5-binding protein 1; - OS=Homo sapiens (Human).
D2JYH4_HUMAN **Mass:** 41982 **Score:** 71 **Matches:** 2(2) **Sequences:** 2(2)
SubName: Full=Actin, alpha 2, smooth muscle, aorta; SubName: Full=Actin, alpha 2, smooth muscle, aorta, isoform CRA_a; - OS=Homo sapiens
B3KPP5_HUMAN **Mass:** 20930 **Score:** 71 **Matches:** 2(2) **Sequences:** 2(2)
SubName: Full=cDNA FLJ32030 fis, clone NTONG2000040, highly similar to Actin, alpha cardiac; - OS=Homo sapiens (Human).
B3KUD3_HUMAN **Mass:** 37800 **Score:** 71 **Matches:** 2(2) **Sequences:** 2(2)
SubName: Full=cDNA FLJ39583 fis, clone SKMUS2004897, highly similar to ACTIN, ALPHA SKELETAL MUSCLE; - OS=Homo sapiens (Human).
B3KWQ3_HUMAN **Mass:** 28193 **Score:** 71 **Matches:** 2(2) **Sequences:** 2(2)
SubName: Full=cDNA FLJ43573 fis, clone RECTM2001691, highly similar to Actin, cytoplasmic 2; - OS=Homo sapiens (Human).
A4UCT3_HUMAN **Mass:** 14727 **Score:** 71 **Matches:** 2(2) **Sequences:** 2(2)
SubName: Full=Beta-actin; Flags: Fragment; - OS=Homo sapiens (Human).
B4DUI8_HUMAN **Mass:** 37339 **Score:** 71 **Matches:** 2(2) **Sequences:** 2(2)
SubName: Full=cDNA FLJ52761, highly similar to Actin, aortic smooth muscle; - OS=Homo sapiens (Human).
B4DVQ0_HUMAN **Mass:** 37325 **Score:** 71 **Matches:** 2(2) **Sequences:** 2(2)
SubName: Full=cDNA FLJ58286, highly similar to Actin, cytoplasmic 2; - OS=Homo sapiens (Human).
B4DW52_HUMAN **Mass:** 38608 **Score:** 71 **Matches:** 2(2) **Sequences:** 2(2)
SubName: Full=cDNA FLJ55253, highly similar to Actin, cytoplasmic 1; - OS=Homo sapiens (Human).
B4E315_HUMAN **Mass:** 37105 **Score:** 71 **Matches:** 2(2) **Sequences:** 2(2)
SubName: Full=cDNA FLJ51195, highly similar to Actin, gamma-enteric smooth muscle; - OS=Homo sapiens (Human).
B4E335_HUMAN **Mass:** 39201 **Score:** 71 **Matches:** 2(2) **Sequences:** 2(2)
SubName: Full=cDNA FLJ52842, highly similar to Actin, cytoplasmic 1; - OS=Homo sapiens (Human).
B4E3A4_HUMAN **Mass:** 39774 **Score:** 71 **Matches:** 2(2) **Sequences:** 2(2)
SubName: Full=cDNA FLJ57283, highly similar to Actin, cytoplasmic 2; - OS=Homo sapiens (Human).
A5GZ75_HUMAN **Mass:** 16847 **Score:** 71 **Matches:** 2(2) **Sequences:** 2(2)
SubName: Full=Beta-actin; Flags: Fragment; - OS=Homo sapiens (Human).
Q5T8M7_HUMAN **Mass:** 37800 **Score:** 71 **Matches:** 2(2) **Sequences:** 2(2)
SubName: Full=Actin, alpha 1, skeletal muscle; SubName: Full=Actin, alpha skeletal muscle; - OS=Homo sapiens (Human).
Q5T8M8_HUMAN **Mass:** 32028 **Score:** 71 **Matches:** 2(2) **Sequences:** 2(2)
SubName: Full=Actin, alpha 1, skeletal muscle; SubName: Full=Actin, alpha skeletal muscle; - OS=Homo sapiens (Human).
A6NL76_HUMAN **Mass:** 32254 **Score:** 71 **Matches:** 2(2) **Sequences:** 2(2)
SubName: Full=Actin, alpha skeletal muscle; - OS=Homo sapiens (Human).
Q6PJ43_HUMAN **Mass:** 29393 **Score:** 71 **Matches:** 2(2) **Sequences:** 2(2)
SubName: Full=ACTG1 protein; Flags: Fragment; - OS=Homo sapiens (Human).
B7Z6P1_HUMAN **Mass:** 38554 **Score:** 71 **Matches:** 2(2) **Sequences:** 2(2)
SubName: Full=cDNA FLJ53662, highly similar to Actin, alpha skeletal muscle; - OS=Homo sapiens (Human).
B7ZAP6_HUMAN **Mass:** 33080 **Score:** 71 **Matches:** 2(2) **Sequences:** 2(2)
SubName: Full=cDNA, FLJ79260, highly similar to Actin, cytoplasmic 2; - OS=Homo sapiens (Human).
Q7Z7J6_HUMAN **Mass:** 28133 **Score:** 71 **Matches:** 2(2) **Sequences:** 2(2)
SubName: Full=Actin alpha 1 skeletal muscle protein; - OS=Homo sapiens (Human).
A8K3K1_HUMAN **Mass:** 42020 **Score:** 71 **Matches:** 2(2) **Sequences:** 2(2)
SubName: Full=cDNA FLJ78096, highly similar to Homo sapiens actin, alpha, cardiac muscle (ACTC), mRNA; - OS=Homo sapiens (Human).
Q8WVW5_HUMAN **Mass:** 40477 **Score:** 71 **Matches:** 2(2) **Sequences:** 2(2)
SubName: Full=Putative uncharacterized protein; Flags: Fragment; - OS=Homo sapiens (Human).
E9PG30_HUMAN **Mass:** 37059 **Score:** 71 **Matches:** 2(2) **Sequences:** 2(2)
SubName: Full=Actin, gamma-enteric smooth muscle; - OS=Homo sapiens (Human).
Q53G76_HUMAN **Mass:** 41694 **Score:** 71 **Matches:** 2(2) **Sequences:** 2(2)
SubName: Full=Beta actin variant; Flags: Fragment; - OS=Homo sapiens (Human).
Q53G99_HUMAN **Mass:** 41738 **Score:** 71 **Matches:** 2(2) **Sequences:** 2(2)
SubName: Full=Beta actin variant; Flags: Fragment; - OS=Homo sapiens (Human).
Q53GK6_HUMAN **Mass:** 41696 **Score:** 71 **Matches:** 2(2) **Sequences:** 2(2)
SubName: Full=Beta actin variant; Flags: Fragment; - OS=Homo sapiens (Human).
Q96DE1_HUMAN **Mass:** 17713 **Score:** 71 **Matches:** 2(2) **Sequences:** 2(2)
SubName: Full=Putative uncharacterized protein; Flags: Fragment; - OS=Homo sapiens (Human).
Q96FU6_HUMAN **Mass:** 18439 **Score:** 71 **Matches:** 2(2) **Sequences:** 2(2)
SubName: Full=ACTG1 protein; Flags: Fragment; - OS=Homo sapiens (Human).

---

4. A0N4V7_HUMAN **Mass:** 2212 **Score:** 50 **Matches:** 6(5) **Sequences:** 1(1) **emPAI:** 1.81
SubName: Full=HCG2039797; SubName: Full=Possible J 56 gene segment; Flags: Fragment; - OS=Homo sapiens (Human).

☐ Check to include this hit in error tolerant search or archive report

	Query	Observed	Mr(expt)	Mr(calc)	ppm	Miss	Score	Expect	Rank	Unique	Retention Time	Peptide
☑	4	421.7567	841.4988	841.5022	-4.02	0	(30)	0.055	1	U	722s (12.03m)	K.GITLSVRP.-

[x]	71	421.7573	841.5001	841.5022	-2.48	0	(29)	0.069	1	U	726s (12.10m)	K.GITLSVRP.-
[x]	72	421.7592	841.5038	841.5022	1.92	0	(31)	0.042	1	U	544s (9.07m)	K.GITLSVRP.-
[x]	73	421.7592	841.5038	841.5022	1.92	0	33	0.025	1	U	541s (9.02m)	K.GITLSVRP.-
[x]	74	421.7601	841.5056	841.5022	4.08	0	(30)	0.048	1	U	633s (10.55m)	K.GITLSVRP.-
[x]	75	421.7601	841.5056	841.5022	4.08	0	(32)	0.033	1	U	637s (10.62m)	K.GITLSVRP.-

---

5. K2C1_HUMAN **Mass:** 65999 **Score:** 50 **Matches:** 1(1) **Sequences:** 1(1) **emPAI:** 0.05
RecName: Full=Keratin, type II cytoskeletal 1; AltName: Full=67 kDa cytokeratin; AltName: Full=Cytokeratin-1; Short=CK-1; AltName: Full=
[ ] Check to include this hit in error tolerant search or archive report

	Query	Observed	Mr(expt)	Mr(calc)	ppm	Miss	Score	Expect	Rank	Unique	Retention Time	Peptide
[x]	90	437.7554	873.4963	873.4920	5.01	0	50	0.00094	1	U	579s (9.65m)	R.SLVNLGGSK.S

Proteins matching the same set of peptides:
H6VRF8_HUMAN **Mass:** 66013 **Score:** 50 **Matches:** 1(1) **Sequences:** 1(1)
SubName: Full=Keratin 1; - OS=Homo sapiens (Human).
H6VRG0_HUMAN **Mass:** 65930 **Score:** 50 **Matches:** 1(1) **Sequences:** 1(1)
SubName: Full=Keratin 1; - OS=Homo sapiens (Human).
H6VRG1_HUMAN **Mass:** 66086 **Score:** 50 **Matches:** 1(1) **Sequences:** 1(1)
SubName: Full=Keratin 1; - OS=Homo sapiens (Human).
H6VRG2_HUMAN **Mass:** 66026 **Score:** 50 **Matches:** 1(1) **Sequences:** 1(1)
SubName: Full=Keratin 1; - OS=Homo sapiens (Human).
H6VRG3_HUMAN **Mass:** 66071 **Score:** 50 **Matches:** 1(1) **Sequences:** 1(1)
SubName: Full=Keratin 1; - OS=Homo sapiens (Human).

---

6. B3KQZ8_HUMAN **Score:** 30 **Matches:** 1(1) **Sequences:** 1(1) **emPAI:** 0.04
SubName: Full=cDNA FLJ33349 fis, clone BRACE2003905 {ECO:0000313|EMBL:BAG52210.1} OS=Homo sapiens (Human)
[ ] Check to include this hit in error tolerant search or archive report

Query	Observed	Mr(expt)	Mr(calc)	ppm	Miss	Score	Expect	Rank	Unique	Retention Time	Peptide
48	398.2378	794.4611	794.4650	-4.86	0	30	0.036	2	U	529s (8.82m)	R.LLPAEPR.L

Proteins matching the same set of peptides:
K1614_HUMAN **Score:** 30 **Matches:** 1(1) **Sequences:** 1(1)
RecName: Full=Uncharacterized protein KIAA1614 OS=Homo sapiens (Human)

---

7. J3KSK6_HUMAN **Mass:** 55408 **Score:** 15 **Matches:** 5(0) **Sequences:** 1(0) **emPAI:** 0.06
SubName: Full=Protein-methionine sulfoxide oxidase MICAL3; Flags: Fragment; - OS=Homo sapiens (Human).
[ ] Check to include this hit in error tolerant search or archive report

	Query	Observed	Mr(expt)	Mr(calc)	ppm	Miss	Score	Expect	Rank	Unique	Retention Time	Peptide
[x]	296	422.5834	1264.7283	1264.7213	5.57	1	(6)	11	1	U	959s (15.98m)	K.VKYMATQLLAK.F
[x]	297	422.5834	1264.7283	1264.7213	5.57	1	15	1.5	1	U	965s (16.08m)	K.VKYMATQLLAK.F
[x]	298	422.5834	1264.7283	1264.7213	5.57	1	(1)	35	1	U	974s (16.23m)	K.VKYMATQLLAK.F
[x]	299	422.5834	1264.7283	1264.7213	5.57	1	(11)	4.1	1	U	962s (16.03m)	K.VKYMATQLLAK.F
[x]	300	422.5836	1264.7291	1264.7213	6.16	1	(7)	10	1	U	932s (15.53m)	K.VKYMATQLLAK.F

Proteins matching the same set of peptides:
MICA3_HUMAN **Mass:** 224156 **Score:** 15 **Matches:** 5(0) **Sequences:** 1(0)
RecName: Full=Protein-methionine sulfoxide oxidase MICAL3; EC=1.14.13.-; AltName: Full=Molecule interacting with CasL protein 3; Short=M

---

8. H0Y4J2_HUMAN **Score:** 0 **Matches:** 5(0) **Sequences:** 3(0)
SubName: Full=39S ribosomal protein L37, mitochondrial {ECO:0000313|Ensembl:ENSP00000392216}; Flags: Fragment OS=Homo sapiens (Human)
[ ] Check to include this hit in error tolerant search or archive report

Query	Observed	Mr(expt)	Mr(calc)	ppm	Miss	Score	Expect	Rank	Unique	Retention Time	Peptide
120	466.2714	930.5282	930.5321	-4.18	0	1	67	3	U	611s (10.18m)	-.CSLLLQVR.G
127	471.7927	941.5708	941.5658	5.32	0	(8)	7.8	3	U	650s (10.83m)	-.NSLLLQVR.G
128	471.7927	941.5708	941.5658	5.32	0	20	0.52	2	U	645s (10.75m)	-.NSLLLQVR.G
144	488.2858	974.5571	974.5583	-1.22	0	(5)	19	3	U	684s (11.40m)	-.MSLLLQVR.G + Oxidation (M)
145	488.2858	974.5571	974.5583	-1.22	0	6	18	3	U	681s (11.35m)	-.MSLLLQVR.G + Oxidation (M)

---

9. H0YEG8_HUMAN **Mass:** 23746 **Score:** 0 **Matches:** 3(0) **Sequences:** 2(0)
SubName: Full=Nucleobindin-2; Flags: Fragment; - OS=Homo sapiens (Human).
[ ] Check to include this hit in error tolerant search or archive report

	Query	Observed	Mr(expt)	Mr(calc)	ppm	Miss	Score	Expect	Rank	Unique	Retention Time	Peptide
	176	518.7771	1035.5397	1035.5349	4.64	1	5	29	4	U	534s (8.90m)	-.FTLNEEKR.K
	241	577.8119	1153.6091	1153.6203	-9.69	2	(15)	3.7	2	U	508s (8.47m)	-.HTLNEEKRK.E
[x]	242	577.8119	1153.6091	1153.6203	-9.69	2	21	0.95	1	U	506s (8.43m)	-.HTLNEEKRK.E

---

10. Q8N6K3_HUMAN **Mass:** 64718 **Score:** 0 **Matches:** 2(0) **Sequences:** 1(0)
SubName: Full=SNX25 protein; - OS=Homo sapiens (Human).
[ ] Check to include this hit in error tolerant search or archive report

	Query	Observed	Mr(expt)	Mr(calc)	ppm	Miss	Score	Expect	Rank	Unique	Retention Time	Peptide
[x]	127	471.7927	941.5708	941.5770	-6.59	2	(21)	0.36	1	U	650s (10.83m)	M.KADLLRAR.N
[x]	128	471.7927	941.5708	941.5770	-6.59	2	24	0.18	1	U	645s (10.75m)	M.KADLLRAR.N

---

11. FETUA_HUMAN **Mass:** 39300 **Score:** 0 **Matches:** 1(0) **Sequences:** 1(0)
RecName: Full=Alpha-2-HS-glycoprotein; AltName: Full=Alpha-2-Z-globulin; AltName: Full=Ba-alpha-2-glycoprotein; AltName: Full=Fetuin-A;
☐ Check to include this hit in error tolerant search or archive report

	Query	Observed	Mr(expt)	Mr(calc)	ppm	Miss	Score	Expect	Rank	Unique	Retention Time	Peptide
☑	40	387.7008	773.3870	773.3820	6.43	0	23	0.29	1	U	495s (8.25m)	R.AHYDLR.H

Proteins matching the same set of peptides:
B7Z8Q2_HUMAN **Mass:** 46597 **Score:** 0 **Matches:** 1(0) **Sequences:** 1(0)
SubName: Full=cDNA FLJ55606, highly similar to Alpha-2-HS-glycoprotein; - OS=Homo sapiens (Human).
C9JV77_HUMAN **Mass:** 39387 **Score:** 0 **Matches:** 1(0) **Sequences:** 1(0)
SubName: Full=Alpha-2-HS-glycoprotein; - OS=Homo sapiens (Human).

12. O10A7_HUMAN **Mass:** 35670 **Score:** 0 **Matches:** 2(0) **Sequences:** 1(0)
RecName: Full=Olfactory receptor 10A7; AltName: Full=Olfactory receptor OR12-6; - OS=Homo sapiens (Human).
☐ Check to include this hit in error tolerant search or archive report

	Query	Observed	Mr(expt)	Mr(calc)	ppm	Miss	Score	Expect	Rank	Unique	Retention Time	Peptide
☑	69	421.2928	840.5710	840.5797	-10.33	0	(19)	0.14	1	U	978s (16.30m)	R.IIITILR.M
☑	70	421.2928	840.5710	840.5797	-10.33	0	21	0.078	1	U	976s (16.27m)	R.IIITILR.M

13. B2R747_HUMAN **Mass:** 51907 **Score:** 0 **Matches:** 2(0) **Sequences:** 1(0)
SubName: Full=cDNA, FLJ93281, highly similar to Homo sapiens calcium/calmodulin-dependent protein kinase IV (CAMK4), mRNA; - OS=Homo sap
☐ Check to include this hit in error tolerant search or archive report

	Query	Observed	Mr(expt)	Mr(calc)	ppm	Miss	Score	Expect	Rank	Unique	Retention Time	Peptide
☑	256	592.8363	1183.6581	1183.6561	1.75	1	(20)	0.64	1	U	618s (10.30m)	K.LKTVEEAAAPR.E
☑	257	592.8363	1183.6581	1183.6561	1.75	1	20	0.53	1	U	622s (10.37m)	K.LKTVEEAAAPR.E

Proteins matching the same set of peptides:
KCC4_HUMAN **Mass:** 51893 **Score:** 0 **Matches:** 2(0) **Sequences:** 1(0)
RecName: Full=Calcium/calmodulin-dependent protein kinase type IV; Short=CaMK IV; EC=2.7.11.17; AltName: Full=CaM kinase-GR; - OS=Homo s

14. B4DX30_HUMAN **Mass:** 51692 **Score:** 0 **Matches:** 1(0) **Sequences:** 1(0)
SubName: Full=Long-chain-fatty-acid--CoA ligase 5; SubName: Full=cDNA FLJ52792, highly similar to Long-chain-fatty-acid--CoA ligase 5 (E
☐ Check to include this hit in error tolerant search or archive report

	Query	Observed	Mr(expt)	Mr(calc)	ppm	Miss	Score	Expect	Rank	Unique	Retention Time	Peptide
☑	173	517.8055	1033.5964	1033.5920	4.31	1	19	0.62	1	U	678s (11.30m)	R.LLNRIYDK.V

Proteins matching the same set of peptides:
ACSL5_HUMAN **Mass:** 75942 **Score:** 0 **Matches:** 1(0) **Sequences:** 1(0)
RecName: Full=Long-chain-fatty-acid--CoA ligase 5; EC=6.2.1.3; AltName: Full=Long-chain acyl-CoA synthetase 5; Short=LACS 5; - OS=Homo s
A6GV77_HUMAN **Mass:** 73231 **Score:** 0 **Matches:** 1(0) **Sequences:** 1(0)
SubName: Full=Fatty acid coenzyme A ligase 5; EC=6.2.1.3; - OS=Homo sapiens (Human).

15. H0Y9B1_HUMAN **Mass:** 33014 **Score:** 0 **Matches:** 3(0) **Sequences:** 2(0)
SubName: Full=Interleukin-6 receptor subunit alpha; Flags: Fragment; - OS=Homo sapiens (Human).
☐ Check to include this hit in error tolerant search or archive report

	Query	Observed	Mr(expt)	Mr(calc)	ppm	Miss	Score	Expect	Rank	Unique	Retention Time	Peptide
	12	477.8229	953.6312	953.6246	6.91	2	9	1.8	2	U	879s (14.65m)	-.QRRLLLR.S
	132	478.3146	954.6146	954.6086	6.21	2	(9)	2	2	U	1015s (16.92m)	-.ERRLLLR.S
☑	133	478.3146	954.6146	954.6086	6.21	2	9	1.9	1	U	1012s (16.87m)	-.ERRLLLR.S

16. CCAR1_HUMAN **Mass:** 132739 **Score:** 0 **Matches:** 2(0) **Sequences:** 2(0)
RecName: Full=Cell division cycle and apoptosis regulator protein 1; AltName: Full=Cell cycle and apoptosis regulatory protein 1; Short=
☐ Check to include this hit in error tolerant search or archive report

	Query	Observed	Mr(expt)	Mr(calc)	ppm	Miss	Score	Expect	Rank	Unique	Retention Time	Peptide
☑	99	448.2604	894.5063	894.4997	7.33	1	6	10	1	U	865s (14.42m)	K.FLVGMKGK.D + Oxidation (M)
☑	12	477.8229	953.6312	953.6386	-7.69	1	11	0.95	1	U	879s (14.65m)	K.LLNKVVLR.E

17. PLXA3_HUMAN **Score:** 0 **Matches:** 2(0) **Sequences:** 2(0)
RecName: Full=Plexin-A3; AltName: Full=Plexin-4; AltName: Full=Semaphorin receptor SEX; Flags: Precursor OS=Homo sapiens (Human)
☐ Check to include this hit in error tolerant search or archive report

	Query	Observed	Mr(expt)	Mr(calc)	ppm	Miss	Score	Expect	Rank	Unique	Retention Time	Peptide
	17	513.8019	1025.5892	1025.5869	2.26	0	9	4.6	4	U	653s (10.88m)	K.LAPNLTELR.A
	253	588.3223	1174.6300	1174.6306	-0.53	2	8	13	2	U	795s (13.25m)	R.KTQDADRTLK.R

18. E9PP40_HUMAN **Mass:** 33526 **Score:** 0 **Matches:** 2(0) **Sequences:** 1(0)
SubName: Full=Leucine-rich repeat-containing protein 14; Flags: Fragment; - OS=Homo sapiens (Human).
☐ Check to include this hit in error tolerant search or archive report

	Query	Observed	Mr(expt)	Mr(calc)	ppm	Miss	Score	Expect	Rank	Unique	Retention Time	Peptide
☑	334	658.8802	1315.7458	1315.7361	7.40	1	17	1	1	U	1141s (19.02m)	R.VDLRFNNLGLR.G
☑	335	658.8802	1315.7458	1315.7361	7.40	1	(17)	1.1	1	U	1144s (19.07m)	R.VDLRFNNLGLR.G

Proteins matching the same set of peptides:
LRC14_HUMAN **Mass:** 54478 **Score:** 0 **Matches:** 2(0) **Sequences:** 1(0)
RecName: Full=Leucine-rich repeat-containing protein 14; - OS=Homo sapiens (Human).

---

19. Q6UXV8_HUMAN **Mass:** 10118 **Score:** 0 **Matches:** 2(0) **Sequences:** 1(0)
SubName: Full=KCNQ2; - OS=Homo sapiens (Human).
☐ Check to include this hit in error tolerant search or archive report

	Query	Observed	Mr(expt)	Mr(calc)	ppm	Miss	Score	Expect	Rank	Unique	Retention Time	Peptide
☑	62	**412.7543**	**823.4941**	**823.4817**	**15.1**	**0**	**17**	**0.42**	**1**	**U**	**572s (9.53m)**	**R.WRPLPR.L**
☑	63	**412.7543**	**823.4941**	**823.4817**	**15.1**	**0**	**(8)**	**3.3**	**1**	**U**	**570s (9.50m)**	**R.WRPLPR.L**

---

20. GEMI5_HUMAN **Score:** 0 **Matches:** 2(0) **Sequences:** 2(0)
RecName: Full=Gem-associated protein 5; Short=Gemin5 OS=Homo sapiens (Human)
☐ Check to include this hit in error tolerant search or archive report

Query	Observed	Mr(expt)	Mr(calc)	ppm	Miss	Score	Expect	Rank	Unique	Retention Time	Peptide
128	471.7927	941.5708	941.5658	5.36	1	12	3.5	3	U	645s (10.75m)	R.EAIAIAKAR.L
246	581.3126	1160.6107	1160.5972	11.7	1	5	28	3	U	612s (10.20m)	R.LTEANQRMAK.F

Proteins matching the same set of peptides:
B7ZLC9_HUMAN **Score:** 0 **Matches:** 2(0) **Sequences:** 2(0)
SubName: Full=GEMIN5 protein {ECO:0000313|EMBL:AAI43725.1} OS=Homo sapiens (Human)

Peptide matches not assigned to protein hits: (no details means no match)

	Query	Observed	Mr(expt)	Mr(calc)	ppm	Miss	Score	Expect	Rank	Unique	Retention Time	Peptide
☑	81	**427.7584**	**853.5023**	**853.5134**	**-13.00**	**1**	**16**	**0.65**	**1**		**617s (10.28m)**	**VDVRLPR**
☑	17	513.8019	1025.5892	1025.5869	2.24	0	16	0.96	1		653s (10.88m)	SLSLSLPGPR
☑	241	577.8119	1153.6091	1153.6131	-3.46	0	15	3.4	1		508s (8.47m)	HITIEDLWK
☑	160	**502.2923**	**1002.5700**	**1002.5532**	**16.7**	**0**	**15**	**2.2**	**1**		**595s (9.92m)**	**MLSLSLSPR**
☑	20	**530.2804**	**1058.5463**	**1058.5356**	**10.1**	**0**	**15**	**5.6**	**1**		**601s (10.02m)**	**GTGSGALPSGQK**
☑	116	**464.3003**	**926.5859**	**926.5913**	**-5.75**	**1**	**14**	**1.1**	**1**		**848s (14.13m)**	**RADVLLLK**
☑	181	**523.2856**	**1044.5566**	**1044.5716**	**-14.32**	**0**	**14**	**4**	**1**		**517s (8.62m)**	**LSSAHVYLR**
☑	122	**467.7334**	**933.4522**	**933.4655**	**-14.21**	**1**	**13**	**3.9**	**1**		**849s (14.15m)**	**LTKSELDE**
☑	151	**495.2945**	**988.5744**	**988.5706**	**3.88**	**0**	**13**	**2.7**	**1**		**684s (11.40m)**	**FLSGLLSPR**
☑	462	**522.5964**	**1564.7674**	**1564.7594**	**5.12**	**1**	**13**	**15**	**1**		**486s (8.10m)**	**INAPSQESDSHPRK**
☑	39	**385.7642**	**769.5138**	**769.5035**	**13.5**	**2**	**13**	**0.97**	**1**		**520s (8.67m)**	**IRARVR**
☑	320	**655.3267**	**1308.6389**	**1308.6649**	**-19.84**	**1**	**12**	**7**	**1**		**630s (10.50m)**	**YAMAWGVVERK**
☑	494	**881.9254**	**1761.8363**	**1761.8646**	**-16.06**	**0**	**12**	**3.7**	**1**		**766s (12.77m)**	**DAHSVITYQITSGNTR**
☑	7	**445.2687**	**888.5229**	**888.5181**	**5.38**	**0**	**12**	**5.3**	**1**		**673s (11.22m)**	**FLNLNLR**
☑	38	**385.2046**	**768.3947**	**768.3878**	**8.97**	**1**	**12**	**2.6**	**1**		**570s (9.50m)**	**EKAHER**
☑	27	**593.3342**	**1184.6539**	**1184.6765**	**-19.09**	**0**	**12**	**13**	**1**		**615s (10.25m)**	**VLSTDILTAPR**
☑	226	**564.3211**	**1126.6276**	**1126.6095**	**16.1**	**1**	**11**	**4.2**	**1**		**1252s (20.87m)**	**EGRALPATGQK**
☑	161	**502.2923**	**1002.5700**	**1002.5532**	**16.7**	**0**	**11**	**5.4**	**1**		**593s (9.88m)**	**MLSLSLSPR**
☑	37	**385.2046**	**768.3947**	**768.3878**	**8.97**	**1**	**11**	**3**	**1**		**567s (9.45m)**	**EKAHER**
☑	252	**588.3223**	**1174.6300**	**1174.6268**	**2.72**	**0**	**11**	**6.7**	**1**		**798s (13.30m)**	**TLVLVSPGDMK + Oxidation (M)**
☑	455	**513.5923**	**1537.7550**	**1537.7848**	**-19.43**	**2**	**11**	**6.3**	**1**		**490s (8.17m)**	**RAADVEPSSPEPKR**
☑	121	**467.7299**	**933.4453**	**933.4590**	**-14.62**	**0**	**11**	**5.6**	**1**		**638s (10.63m)**	**QVMAEVNK + Oxidation (M)**
☑	316	**434.5851**	**1300.7336**	**1300.7173**	**12.5**	**2**	**11**	**5.2**	**1**		**586s (9.77m)**	**MKEEVKGIPVR + Oxidation (M)**
☑	253	588.3223	1174.6300	1174.6268	2.72	0	11	7.6	1		795s (13.25m)	TLVLVSPGDMK + Oxidation (M)
☑	21	**534.7943**	**1067.5740**	**1067.5685**	**5.16**	**0**	**10**	**4.9**	**1**		**721s (12.02m)**	**LMNIFSSLK + Oxidation (M)**
☑	180	**523.2856**	**1044.5566**	**1044.5716**	**-14.32**	**0**	**10**	**8.8**	**1**		**510s (8.50m)**	**LSSAHVYLR**
☑	132	478.3146	954.6146	954.5974	18.0	1	10	1.6	1		1015s (16.92m)	NLLKGAALR
☑	11	**467.7275**	**933.4405**	**933.4556**	**-16.19**	**0**	**10**	**7.2**	**1**		**641s (10.68m)**	**NIGSETWK**
☑	501	**897.9149**	**1793.8153**	**1793.8288**	**-7.49**	**1**	**10**	**4.2**	**1**		**781s (13.02m)**	**NGDEKSPLGTEIMMTR + Oxidation (M)**
☑	464	**783.8772**	**1565.7398**	**1565.7429**	**-1.95**	**2**	**10**	**7.5**	**1**		**616s (10.27m)**	**EMDAIQAKEEMKK + Oxidation (M)**
☑	261	**596.3188**	**1190.6230**	**1190.6004**	**19.0**	**2**	**10**	**8.8**	**1**		**711s (11.85m)**	**DVSRTDRTNK**
☑	176	518.7771	1035.5397	1035.5574	-17.06	2	10	8.8	1		534s (8.90m)	TKHTHKER
☑	214	**549.7828**	**1097.5511**	**1097.5717**	**-18.77**	**1**	**10**	**7.6**	**1**		**617s (10.28m)**	**KELPPNGDTK**
☑	80	**427.7584**	**853.5023**	**853.5134**	**-13.00**	**1**	**9**	**3.1**	**1**		**620s (10.33m)**	**VDVRLPR**
☑	115	**463.8019**	**925.5892**	**925.5961**	**-7.42**	**0**	**9**	**2.6**	**1**		**846s (14.10m)**	**DVLIVLVR**
☑	227	**567.3287**	**1132.6429**	**1132.6353**	**6.72**	**0**	**9**	**7.7**	**1**		**836s (13.93m)**	**VVQLLPDGHR**
☑	527	**747.0535**	**2238.1386**	**2238.1757**	**-16.57**	**0**	**9**	**8.7**	**1**		**758s (12.63m)**	**IGGSVHLIVNNQLGYTTPAER**
☑	371	**457.6114**	**1369.8123**	**1369.8333**	**-15.37**	**0**	**9**	**3.3**	**1**		**982s (16.37m)**	**LPPSNPLPLVPVK**
☑	524	**742.3825**	**2224.1256**	**2224.1310**	**-2.43**	**2**	**9**	**9.7**	**1**		**753s (12.55m)**	**KLMLDTWNESIFSNIKNR + Oxidation (M)**
☑	188	**531.7968**	**1061.5790**	**1061.5982**	**-18.04**	**0**	**9**	**11**	**1**		**1042s (17.37m)**	**SHSPVLPGIR**
☑	120	466.2714	930.5282	930.5321	-4.18	2	9	11	1		611s (10.18m)	GPVSMKGKK
☑	362	**457.2742**	**1368.8007**	**1368.7837**	**12.4**	**1**	**9**	**4.3**	**1**		**985s (16.42m)**	**QAQQVIQKGITR**
☑	187	**529.2884**	**1056.5621**	**1056.5498**	**11.7**	**1**	**9**	**9.4**	**1**		**539s (8.98m)**	**LGEPQARMR**
☑	375	**697.8529**	**1393.6913**	**1393.6950**	**-2.62**	**0**	**8**	**10**	**1**		**762s (12.70m)**	**EQSQTPGANLPPR**
☑	18	**516.3028**	**1030.5911**	**1030.5771**	**13.6**	**1**	**8**	**15**	**1**		**740s (12.33m)**	**ISPKSTGSVR**
☑	162	**505.2715**	**1008.5285**	**1008.5352**	**-6.69**	**1**	**8**	**12**	**1**		**748s (12.47m)**	**KAEGPSPPAR**
☑	198	**534.8568**	**1067.6990**	**1067.6815**	**16.4**	**2**	**8**	**0.97**	**1**		**1232s (20.53m)**	**GVRELLRIL**
☑	95	**446.2452**	**890.4759**	**890.4895**	**-15.34**	**0**	**8**	**14**	**1**		**568s (9.47m)**	**VVGLAMGTK + Oxidation (M)**
☑	225	**564.3210**	**1126.6274**	**1126.6207**	**5.96**	**2**	**8**	**9.6**	**1**		**1256s (20.93m)**	**VQDKTGRAPR**
☑	238	**571.8159**	**1141.6173**	**1141.5979**	**17.0**	**0**	**8**	**13**	**1**		**591s (9.85m)**	**LEESLDALPR**
☑	336	**659.3746**	**1316.7346**	**1316.7234**	**8.47**	**2**	**8**	**11**	**1**		**1138s (18.97m)**	**SKEEVMAVRLR**
☑	172	**516.3000**	**1030.5855**	**1030.5811**	**4.23**	**0**	**8**	**14**	**1**		**742s (12.37m)**	**TVPNIFVNK**
☑	88	**435.7559**	**869.4972**	**869.4971**	**0.13**	**0**	**8**	**5.7**	**1**		**719s (11.98m)**	**TPSIPSIR**
☑	463	**783.8772**	**1565.7398**	**1565.7541**	**-9.14**	**0**	**8**	**13**	**1**		**619s (10.32m)**	**ATCIGNNSAAAVSMLK + Oxidation (M)**
☑	145	488.2858	974.5571	974.5621	-5.13	2	7	11	1		681s (11.35m)	LSKASSRAR
☑	189	**531.7968**	**1061.5790**	**1061.5982**	**-18.02**	**1**	**7**	**18**	**1**		**1038s (17.30m)**	**KHEIIHTGK**
☑	13	**491.2754**	**1470.8043**	**1470.7943**	**6.81**	**1**	**7**	**26**	**1**		**560s (9.33m)**	**SFPSSKALITHQR**

☑	500	897.9149	1793.8153	1793.8028	7.00	0	7	8.1	1	785s (13.08m)	TGTTGQSGAESGTTEPSAR
☑	211	549.7414	1097.4682	1097.4811	-11.75	0	7	4	1	522s (8.70m)	YECEIQASR
☑	212	549.7414	1097.4682	1097.4569	10.4	1	7	4	1	524s (8.73m)	FACPMCDRR
☑	152	495.2945	988.5744	988.5739	0.48	1	7	11	1	688s (11.47m)	MVALKGINK + Oxidation (M)
☑	245	578.8343	1155.6541	1155.6513	2.47	1	7	14	1	497s (8.28m)	GWRVPIVSSR
☑	109	913.9782	1825.9418	1825.9100	17.4	2	7	15	1	759s (12.65m)	AMVTETMMKLRNELK + 2 Oxidation (M)
☑	144	488.2858	974.5571	974.5621	-5.13	2	7	14	1	684s (11.40m)	LSKASSRAR
☑	140	483.2756	964.5365	964.5263	10.6	0	7	12	1	545s (9.08m)	ATVLLSMSK + Oxidation (M)
☑	337	662.8573	1323.7000	1323.7259	-19.55	2	6	29	1	724s (12.07m)	EPNIDRSRLPK
☑	169	512.7348	1023.4551	1023.4734	-17.84	0	6	9.4	1	551s (9.18m)	GDPGPPGQSGR
☑	24	557.3386	1112.6625	1112.6740	-10.26	1	6	20	1	639s (10.65m)	AVKMPVALLR + Oxidation (M)
☑	197	534.7987	1067.5828	1067.5685	13.4	0	6	13	1	716s (11.93m)	LMNIFSSLK + Oxidation (M)
☑	10	464.2806	926.5467	926.5410	6.15	2	6	15	1	1043s (17.38m)	ELRRTPR
☑	130	475.7298	949.4450	949.4287	17.1	0	6	20	1	745s (12.42m)	MGSGAGELGR + Oxidation (M)
☑	526	744.0775	2229.2106	2229.2303	-8.85	2	6	12	1	986s (16.43m)	WSLVCKLNTRTTILAATNPK
☑	102	454.7629	907.5113	907.5028	9.32	2	6	12	1	580s (9.67m)	WFRSGKK
☑	504	901.9721	1801.9296	1801.9547	-13.93	1	6	21	1	801s (13.35m)	INLTIHQRTHVEEGR
☑	57	407.7596	813.5046	813.4960	10.6	0	6	18	1	677s (11.28m)	QDVVLIK
☑	251	585.8462	1169.6778	1169.6629	12.8	2	5	13	1	704s (11.73m)	ASLRDLRSPR
☑	287	624.8530	1247.6914	1247.6808	8.48	2	5	18	1	841s (14.02m)	YGKLSRHLMK + Oxidation (M)
☑	68	421.2601	840.5056	840.5181	-14.90	1	5	11	1	816s (13.60m)	LKGALSPR
☑	129	475.7298	949.4450	949.4287	17.1	0	5	23	1	742s (12.37m)	MGSGAGELGR + Oxidation (M)
☑	286	624.8530	1247.6914	1247.6808	8.48	2	5	19	1	837s (13.95m)	YGKLSRHLMK + Oxidation (M)
☑	496	890.9067	1779.7988	1779.8023	-1.98	2	5	8.4	1	789s (13.15m)	RSSSEESENAEKWNK
☑	64	414.7636	827.5126	827.5229	-12.40	0	5	23	1	696s (11.60m)	LIQSIVR
☑	19	516.3128	1030.6110	1030.6022	8.53	2	5	27	1	1093s (18.22m)	ELKKSGELK
☑	319	655.3267	1308.6389	1308.6530	-10.76	1	5	37	1	627s (10.45m)	LMKSAIGEGMTR + Oxidation (M)
☑	117	465.7787	929.5428	929.5519	-9.74	2	5	26	1	741s (12.35m)	RLASATRR
☑	34	744.0781	2229.2124	2229.2391	-11.94	2	5	15	1	989s (16.48m)	VACALTLPFICRKPSLWRR
☑	302	635.3341	1268.6537	1268.6724	-14.78	0	5	22	1	804s (13.40m)	QHENSSLLLTK
☑	305	641.8844	1281.7542	1281.7769	-17.70	1	5	8.6	1	921s (15.35m)	RVAVVQAILEGK
☑	94	443.7662	885.5179	885.5283	-11.78	1	5	31	1	731s (12.18m)	EEIRLVK
☑	425	730.9182	1459.8219	1459.8372	-10.47	1	5	19	1	613s (10.22m)	RPLDPLLTSRHR
☑	233	569.3109	1136.6072	1136.6131	-5.20	1	4	21	1	743s (12.38m)	IRTWFWTK
☑	249	390.5300	1168.5681	1168.5766	-7.31	2	4	33	1	496s (8.27m)	LMRDMKTMK + Oxidation (M)
☑	317	434.5851	1300.7336	1300.7173	12.5	2	4	23	1	590s (9.83m)	MKEEVKGIPVR + Oxidation (M)
☑	397	710.8657	1419.7169	1419.6904	18.7	1	4	31	1	970s (16.17m)	FSHMHLMAFRK + Oxidation (M)
☑	76	849.4513	1696.8881	1696.8744	8.06	1	4	83	1	683s (11.38m)	HTKELSPTSDTNLVR
☑	525	742.3825	2224.1256	2224.1310	-2.43	2	4	31	1	750s (12.50m)	KLMLDTWNESIFSNIKNR + Oxidation (M)
☑	177	522.7817	1043.5489	1043.5611	-11.72	0	4	31	1	642s (10.70m)	NATAATSPALK
☑	5	426.2466	850.4787	850.4773	1.64	2	4	38	1	810s (13.50m)	KTYQRR
☑	528	747.0535	2238.1386	2238.1790	-18.07	2	4	29	1	755s (12.58m)	LLPPAQAKEEAGGVAAVSGSCKR
☑	443	752.8706	1503.7266	1503.7529	-17.44	1	4	28	1	834s (13.90m)	ASSDEKDSVLNIAR
☑	30	651.8778	1301.7411	1301.7316	7.27	2	4	18	1	805s (13.42m)	KRHLHAVEGQK
☑	220	561.3148	1120.6151	1120.6353	-17.97	1	4	24	1	670s (11.17m)	SRSPIIHSPK
☑	529	747.3815	2239.1228	2239.1360	-5.91	2	4	32	1	743s (12.38m)	TAYPRFRYDQLMHLVWK + Oxidation (M)
☑	447	508.2605	1521.7598	1521.7796	-13.03	1	4	42	1	486s (8.10m)	MHRLVVMSQVYK + 2 Oxidation (M)
☑	250	585.8462	1169.6778	1169.6629	12.8	2	4	21	1	708s (11.80m)	ASLRDLRSPR
☑	184	526.3047	1050.5949	1050.5822	12.1	1	3	17	1	785s (13.08m)	LDKNLPSHK
☑	427	491.5791	1471.7154	1471.7392	-16.23	1	3	27	1	504s (8.40m)	RKPHTNGDAPSHR
☑	138	479.7677	957.5208	957.5131	8.02	0	3	48	1	774s (12.90m)	ITINGESPK
☑	149	492.7503	983.4861	983.4924	-6.39	0	3	24	1	583s (9.72m)	EQAPDISPK
☑	531	762.0613	2283.1622	2283.1338	12.4	0	3	32	1	937s (15.62m)	STSQILPSMSVSICASSTEVLK + Oxidation (M)
☑	25	561.3118	1120.6090	1120.5876	19.0	2	3	69	1	666s (11.10m)	KAKFDGAQEK
☑	309	644.8691	1287.7236	1287.7108	9.92	0	3	24	1	749s (12.48m)	MLAVDAVIAELK + Oxidation (M)
☑	460	777.8410	1553.6675	1553.6666	0.57	1	3	8.2	1	870s (14.50m)	SRSSSSSNASGNGGDGK
☑	270	606.8111	1211.6077	1211.6259	-15.01	1	3	28	1	640s (10.67m)	DLVRDTSPRPG
☑	113	461.2624	920.5102	920.5001	11.1	2	3	30	1	533s (8.88m)	KMEELKK + Oxidation (M)
☑	112	460.7848	919.5551	919.5412	15.1	0	3	10	1	665s (11.08m)	ILVSLTMK + Oxidation (M)
☑	280	615.8838	1229.7530	1229.7496	2.81	0	3	4.1	1	619s (10.32m)	GRPLLLPPPSAL
☑	493	881.9254	1761.8363	1761.8614	-14.26	2	3	29	1	769s (12.82m)	LCEPSGTGAGIMGRKNR + Oxidation (M)
☑	213	549.7828	1097.5511	1097.5717	-18.77	1	3	35	1	621s (10.35m)	KELPPNGDTK
☑	194	534.2704	1066.5262	1066.5268	-0.50	2	3	30	1	522s (8.70m)	RSDAGSYRR
☑	171	514.2998	1026.5850	1026.5934	-8.17	1	3	24	1	952s (15.87m)	QIENVLRR
☑	134	478.7476	955.4806	955.4763	4.45	0	3	22	1	575s (9.58m)	LGNFSYQK
☑	306	641.8844	1281.7542	1281.7517	1.94	1	3	13	1	918s (15.30m)	RATPINLASAIR
☑	399	474.9187	1421.7343	1421.7449	-7.45	2	3	43	1	521s (8.68m)	KTEVLMENFRR
☑	318	651.8761	1301.7376	1301.7316	4.58	2	3	28	1	808s (13.47m)	KRHLHAVEGQK
☑	202	540.2966	1078.5787	1078.5883	-8.95	1	3	43	1	552s (9.20m)	TLQRAEHPK
☑	150	492.7503	983.4861	983.4924	-6.39	0	3	29	1	580s (9.67m)	EQAPDISPK
☑	259	595.3303	1188.6460	1188.6350	9.26	1	2	43	1	811s (13.52m)	EKETALTELR
☑	89	871.4371	870.4298	870.4195	11.8	0	2	1.3e+02	1	967s (16.12m)	DPQLNER
☑	429	737.9251	1473.8356	1473.8389	-2.25	2	2	29	1	657s (10.95m)	RPGRERPPPTRR
☑	58	815.3688	814.3616	814.3569	5.67	1	2	46	1	597s (9.95m)	NDREGPQ
☑	520	707.3443	2119.0110	2119.0368	-12.15	1	2	39	1	596s (9.93m)	DATSKVALVYGQMNEPPGAR + Oxidation (M)
☑	519	1056.9711	2111.9275	2111.9000	13.0	1	2	18	1	759s (12.65m)	DWLQEQMSASSSREPSCR + Oxidation (M)
☑	503	900.9282	1799.8419	1799.8214	11.4	0	2	36	1	983s (16.38m)	EVTEFYVQNENSVDK
☑	260	397.5739	1189.6998	1189.6931	5.60	1	2	18	1	543s (9.05m)	IIEKQPGHIR
☑	446	508.2605	1521.7598	1521.7796	-13.03	1	2	65	1	490s (8.17m)	MHRLVVMSQVYK + 2 Oxidation (M)
☑	266	601.3578	1200.7010	1200.7051	-3.36	2	2	31	1	557s (9.28m)	VTSNLRIRSR
☑	372	463.2849	1386.8329	1386.8096	16.8	2	2	8.8	1	1014s (16.90m)	PKLRIPGDGLGHK
☑	509	970.9760	1939.9375	1939.9639	-13.64	1	2	49	1	782s (13.03m)	GNEYEALTSPQTSFRLK
☑	402	479.9103	1436.7090	1436.6864	15.7	1	2	51	1	488s (8.13m)	GCRLPSISSTTCGR
☑	508	970.9760	1939.9375	1939.9639	-13.64	1	2	51	1	786s (13.10m)	GNEYEALTSPQTSFRLK
☑	498	594.2973	1779.8701	1779.8653	2.71	2	1	58	1	525s (8.75m)	AWRETNVGQNTTRFT

☑	33	743.4323	1484.8501	1484.8385	7.82	2	1	88	1	1086s (18.10m)	DAMAKIQAKVPGLK + Oxidation (M)
☑	32	690.8840	1379.7535	1379.7265	19.6	2	1	43	1	584s (9.73m)	EMQKIMTSLKR + Oxidation (M)
☑	484	827.3968	1652.7790	1652.7724	3.99	0	1	46	1	818s (13.63m)	CPELLANYCDMLLR
☑	54	404.2403	806.4661	806.4650	1.36	1	1	28	1	547s (9.12m)	ILYKDR
☑	516	685.9806	2054.9191	2054.9520	-15.64	1	1	24	1	494s (8.23m)	PQFCILSGGGESKAGFSAGWG
☑	262	596.3188	1190.6230	1190.6004	19.0	2	1	63	1	714s (11.90m)	DVSRTDRTNK
☑	279	615.8838	1229.7530	1229.7496	2.81	0	1	6.3	1	622s (10.37m)	GRPLLLPPPSAL
☑	35	382.2209	762.4272	762.4276	-0.46	0	1	80	1	757s (12.62m)	YGVLSPK
☑	530	762.0613	2283.1622	2283.1304	13.9	2	1	53	1	933s (15.55m)	EDIKEISMSDLKDFLESIR + Oxidation (M)
☑	483	827.3968	1652.7790	1652.7789	0.06	1	1	48	1	820s (13.67m)	AEEIMAAAEKEAMLF
☑	453	769.3254	1536.6363	1536.6258	6.80	0	1	7.3	1	867s (14.45m)	NNMTASMFDLSMK + 3 Oxidation (M)
☑	359	677.3981	1352.7817	1352.7672	10.7	1	1	24	1	1009s (16.82m)	HIIVAVKCMLLN
☑	166	509.8062	1017.5978	1017.6083	-10.35	2	1	28	1	784s (13.07m)	RVAVFNGKK
☑	114	461.2624	920.5102	920.5001	11.1	2	1	49	1	531s (8.85m)	KMEELKK + Oxidation (M)
☑	338	662.8573	1323.7000	1323.7259	-19.55	2	1	95	1	727s (12.12m)	EPNIDRSRLPK
☑	398	710.8657	1419.7169	1419.7358	-13.31	1	1	65	1	967s (16.12m)	EGKVYGQAEAAIGK
☑	311	648.8346	1295.6546	1295.6339	16.0	1	1	59	1	502s (8.37m)	SVVLHSHCCRR
☑	158	501.2923	1000.5701	1000.5553	14.9	0	1	71	1	839s (13.98m)	AATAATSPALK
☑	93	443.7657	885.5168	885.5171	-0.35	0	1	75	1	723s (12.05m)	TLLSPDLK
☑	444	758.4117	1514.8087	1514.8304	-14.27	2	1	63	1	558s (9.30m)	ISLKLANEDKEQK
☑	472	799.8687	1597.7228	1597.7415	-11.72	0	1	47	1	676s (11.27m)	QCSPQTFLLCCLSR
☑	55	404.2403	806.4661	806.4650	1.36	1	1	32	1	550s (9.17m)	ILYKDR
☑	77	425.7620	849.5095	849.5185	-10.56	0	1	23	1	483s (8.05m)	LVLGGHVR
☑	65	414.7636	827.5126	827.5089	4.43	2	1	62	1	692s (11.53m)	ARAKQVR
☑	513	994.0447	1986.0747	1986.1010	-13.23	2	1	55	1	1107s (18.45m)	VSPAHSPPENGLVKARSLK
☑	135	478.7476	955.4806	955.4723	8.65	0	0	40	1	571s (9.52m)	ISGQDSPPR
☑	111	458.2340	914.4534	914.4457	8.45	0	0	38	1	644s (10.73m)	EAPELNSR
☑	186	527.2751	1052.5357	1052.5390	-3.12	0	0	59	1	685s (11.42m)	TEIELSYAK
☑	199	535.7911	1069.5677	1069.5630	4.40	0	0	56	1	596s (9.93m)	MLFGYKPSK
☑	344	664.4020	1326.7894	1326.7871	1.75	2	0	23	1	948s (15.80m)	ELQKIIKDVNK
☑	442	752.8706	1503.7266	1503.7504	-15.79	1	0	68	1	837s (13.95m)	RDALFMDLPTGPR + Oxidation (M)
☑	139	483.2756	964.5365	964.5454	-9.17	1	0	53	1	549s (9.15m)	GITIRSYR
☑	282	619.8704	1237.7263	1237.7030	18.8	1	0	25	1	898s (14.97m)	YTILTSSAVRK
☑	474	536.6637	1606.9693	1606.9446	15.4	1	0	9.5	1	1113s (18.55m)	LGEKQLPILSVPWK
☑	1	*390.7346*	*389.7274*							*823s (13.72m)*	
☑	2	*391.2258*	*390.2185*							*706s (11.77m)*	
☑	3	*391.2260*	*390.2188*							*703s (11.72m)*	
☑	6	*443.2736*	*442.2663*							*844s (14.07m)*	
☑	8	*461.8122*	*460.8049*							*1510s (25.17m)*	
☑	9	*463.8070*	*462.7997*							*1816s (30.27m)*	
☑	14	*492.2795*	*491.2722*							*893s (14.88m)*	
☑	15	*496.3045*	*495.2973*							*787s (13.12m)*	
☑	16	*497.2922*	*496.2849*							*652s (10.87m)*	
☑	22	*536.1644*	*535.1571*							*1086s (18.10m)*	
☑	23	*553.8493*	*552.8420*							*1155s (19.25m)*	
☑	26	*575.8460*	*574.8387*							*850s (14.17m)*	
☑	28	*624.2773*	*623.2701*							*979s (16.32m)*	
☑	29	*639.3045*	*638.2972*							*807s (13.45m)*	
☑	31	*658.7831*	*657.7758*							*519s (8.65m)*	
☑	36	*382.2209*	*762.4272*							*754s (12.57m)*	
☑	41	*779.8488*	*778.8415*							*594s (9.90m)*	
☑	42	*391.2045*	*780.3944*							*614s (10.23m)*	
☑	43	*391.2045*	*780.3944*							*611s (10.18m)*	
☑	44	*392.7487*	*783.4829*							*829s (13.82m)*	
☑	45	*392.7487*	*783.4829*							*826s (13.77m)*	
☑	46	*784.8955*	*783.8882*							*854s (14.23m)*	
☑	47	*791.3073*	*790.3000*							*880s (14.67m)*	
☑	49	*399.2473*	*796.4801*							*783s (13.05m)*	
☑	50	*399.2473*	*796.4801*							*787s (13.12m)*	
☑	51	*806.8479*	*805.8406*							*885s (14.75m)*	
☑	52	*404.2340*	*806.4534*							*784s (13.07m)*	
☑	53	*404.2340*	*806.4534*							*781s (13.02m)*	
☑	56	*811.9072*	*810.8999*							*960s (16.00m)*	
☑	59	*409.7430*	*817.4715*							*530s (8.83m)*	
☑	60	*412.7462*	*823.4779*							*784s (13.07m)*	
☑	61	*412.7473*	*823.4800*							*809s (13.48m)*	
☑	66	*839.9099*	*838.9026*							*821s (13.68m)*	
☑	67	*421.2601*	*840.5056*							*812s (13.53m)*	
☑	78	*425.7620*	*849.5095*							*479s (7.98m)*	
☑	79	*426.2477*	*850.4808*							*806s (13.43m)*	
☑	82	*431.2640*	*860.5134*							*488s (8.13m)*	
☑	83	*431.2640*	*860.5134*							*485s (8.08m)*	
☑	84	*434.7593*	*867.5040*							*813s (13.55m)*	
☑	85	*434.7593*	*867.5040*							*810s (13.50m)*	
☑	86	*435.2521*	*868.4896*							*735s (12.25m)*	
☑	87	*435.2706*	*868.5265*							*1010s (16.83m)*	
☑	91	*440.7328*	*879.4510*							*574s (9.57m)*	
☑	92	*443.2735*	*884.5323*							*839s (13.98m)*	
☑	96	*448.2153*	*894.4160*							*574s (9.57m)*	
☑	97	*448.2153*	*894.4160*							*578s (9.63m)*	
☑	98	*448.2588*	*894.5031*							*835s (13.92m)*	
☑	100	*452.2791*	*902.5436*							*739s (12.32m)*	
☑	101	*452.2792*	*902.5439*							*741s (12.35m)*	
☑	103	*456.7724*	*911.5303*							*835s (13.92m)*	
☑	104	*456.7737*	*911.5328*							*865s (14.42m)*	
☑	105	*457.2662*	*912.5179*							*741s (12.35m)*	

☑	106	457.2662	912.5179	744s (12.40m)
☑	107	457.2827	912.5508	1040s (17.33m)
☑	108	457.2827	912.5508	1037s (17.28m)
☑	110	913.9884	912.9811	763s (12.72m)
☑	118	465.7787	929.5428	745s (12.42m)
☑	119	465.7990	929.5834	1040s (17.33m)
☑	123	467.7380	933.4615	548s (9.13m)
☑	124	470.2725	938.5304	864s (14.40m)
☑	125	471.3059	940.5973	868s (14.47m)
☑	126	471.3059	940.5973	866s (14.43m)
☑	131	478.2591	954.5036	565s (9.42m)
☑	136	478.7865	955.5585	896s (14.93m)
☑	137	478.7865	955.5585	900s (15.00m)
☑	141	972.1756	971.1683	993s (16.55m)
☑	142	487.7926	973.5707	779s (12.98m)
☑	143	487.7926	973.5707	775s (12.92m)
☑	146	490.2791	978.5437	532s (8.87m)
☑	147	490.2849	978.5553	582s (9.70m)
☑	148	492.2870	982.5594	924s (15.40m)
☑	153	496.3054	990.5963	784s (13.07m)
☑	154	496.7989	991.5832	489s (8.15m)
☑	155	500.7994	999.5843	929s (15.48m)
☑	156	500.7994	999.5843	926s (15.43m)
☑	157	501.2923	1000.5701	836s (13.93m)
☑	159	502.1851	1002.3556	748s (12.47m)
☑	163	505.2715	1008.5285	746s (12.43m)
☑	164	507.2759	1012.5373	605s (10.08m)
☑	165	509.3116	1016.6087	929s (15.48m)
☑	167	509.8062	1017.5978	788s (13.13m)
☑	168	512.7348	1023.4551	555s (9.25m)
☑	170	514.2998	1026.5850	956s (15.93m)
☑	174	518.3189	1034.6233	829s (13.82m)
☑	175	518.3189	1034.6233	832s (13.87m)
☑	178	522.7817	1043.5489	638s (10.63m)
☑	179	522.8145	1043.6145	953s (15.88m)
☑	182	523.3049	1044.5953	806s (13.43m)
☑	183	523.3049	1044.5953	803s (13.38m)
☑	185	527.2751	1052.5357	689s (11.48m)
☑	190	531.8187	1061.6229	811s (13.52m)
☑	191	531.8187	1061.6229	814s (13.57m)
☑	192	531.8368	1061.6590	1132s (18.87m)
☑	193	531.8368	1061.6590	1129s (18.82m)
☑	195	534.2928	1066.5710	701s (11.68m)
☑	196	534.7712	1067.5277	686s (11.43m)
☑	200	536.3130	1070.6115	982s (16.37m)
☑	201	536.3130	1070.6115	985s (16.42m)
☑	203	540.2968	1078.5790	542s (9.03m)
☑	204	540.3320	1078.6495	817s (13.62m)
☑	205	540.3321	1078.6496	814s (13.57m)
☑	206	544.8251	1087.6356	982s (16.37m)
☑	207	545.3180	1088.6215	826s (13.77m)
☑	208	545.3364	1088.6583	1154s (19.23m)
☑	209	545.3364	1088.6583	1157s (19.28m)
☑	210	545.8194	1089.6242	824s (13.73m)
☑	215	553.8321	1105.6497	830s (13.83m)
☑	216	553.8321	1105.6497	827s (13.78m)
☑	217	553.8505	1105.6864	1158s (19.30m)
☑	218	555.8029	1109.5912	646s (10.77m)
☑	219	555.8029	1109.5912	642s (10.70m)
☑	221	562.3447	1122.6748	830s (13.83m)
☑	222	562.3447	1122.6748	833s (13.88m)
☑	223	564.2776	1126.5406	557s (9.28m)
☑	224	564.2776	1126.5406	562s (9.37m)
☑	228	567.3315	1132.6484	930s (15.50m)
☑	229	567.3315	1132.6484	926s (15.43m)
☑	230	567.3318	1132.6491	847s (14.12m)
☑	231	567.3493	1132.6840	1186s (19.77m)
☑	232	569.3109	1136.6072	746s (12.43m)
☑	236	571.7751	1141.5356	868s (14.47m)
☑	237	571.7752	1141.5358	861s (14.35m)
☑	239	575.8448	1149.6750	857s (14.28m)
☑	240	575.8620	1149.7095	1182s (19.70m)
☑	243	577.8160	1153.6175	717s (11.95m)
☑	244	577.8160	1153.6175	713s (11.88m)
☑	247	583.3248	1164.6351	840s (14.00m)
☑	248	583.3248	1164.6351	836s (13.93m)
☑	254	589.3447	1176.6748	888s (14.80m)
☑	255	589.3447	1176.6748	890s (14.83m)
☑	258	595.3303	1188.6460	806s (13.43m)
☑	263	597.8575	1193.7005	904s (15.07m)
☑	264	597.8575	1193.7005	901s (15.02m)
☑	265	600.7792	1199.5438	581s (9.68m)
☑	267	606.3156	1210.6166	643s (10.72m)
☑	268	606.3714	1210.7282	875s (14.58m)
☑	269	606.3714	1210.7283	877s (14.62m)

☑	271	608.2691	1214.5237	514s (8.57m)
☑	274	611.3573	1220.7001	893s (14.88m)
☑	275	611.3573	1220.7001	891s (14.85m)
☑	276	407.9081	1220.7024	936s (15.60m)
☑	277	407.9081	1220.7024	943s (15.72m)
☑	278	615.8020	1229.5895	914s (15.23m)
☑	281	618.8421	1235.6697	1038s (17.30m)
☑	283	620.3700	1238.7253	894s (14.90m)
☑	284	623.2693	1244.5240	514s (8.57m)
☑	285	624.2798	1246.5449	982s (16.37m)
☑	288	625.7650	1249.5154	545s (9.08m)
☑	289	628.3842	1254.7538	927s (15.45m)
☑	290	628.3842	1254.7539	930s (15.50m)
☑	291	630.2784	1258.5423	518s (8.63m)
☑	292	630.8534	1259.6923	1024s (17.07m)
☑	293	630.8534	1259.6923	1028s (17.13m)
☑	294	633.3707	1264.7268	909s (15.15m)
☑	295	633.3710	1264.7274	916s (15.27m)
☑	301	635.3341	1268.6537	807s (13.45m)
☑	303	635.8062	1269.5979	511s (8.52m)
☑	304	635.8062	1269.5979	509s (8.48m)
☑	307	428.2600	1281.7582	959s (15.98m)
☑	308	428.2600	1281.7582	968s (16.13m)
☑	310	432.2761	1293.8065	577s (9.62m)
☑	312	650.3966	1298.7786	931s (15.52m)
☑	313	650.3966	1298.7786	932s (15.53m)
☑	314	650.7898	1299.5650	515s (8.58m)
☑	315	650.7898	1299.5650	511s (8.52m)
☑	321	437.2588	1308.7546	1001s (16.68m)
☑	322	437.2588	1308.7546	984s (16.40m)
☑	323	437.2588	1308.7546	998s (16.63m)
☑	324	437.2588	1308.7546	981s (16.35m)
☑	325	437.2588	1308.7546	979s (16.32m)
☑	326	437.2588	1308.7546	988s (16.47m)
☑	327	437.2588	1308.7546	991s (16.52m)
☑	328	437.2588	1308.7546	995s (16.58m)
☑	329	655.3846	1308.7547	939s (15.65m)
☑	330	655.3846	1308.7547	944s (15.73m)
☑	331	437.2596	1308.7570	955s (15.92m)
☑	332	437.2596	1308.7570	966s (16.10m)
☑	333	437.2596	1308.7570	963s (16.05m)
☑	339	663.8974	1325.7803	951s (15.85m)
☑	340	442.9341	1325.7804	981s (16.35m)
☑	341	442.9341	1325.7804	985s (16.42m)
☑	342	442.9341	1325.7805	988s (16.47m)
☑	343	442.9346	1325.7819	956s (15.93m)
☑	345	668.2572	1334.4998	535s (8.92m)
☑	346	672.4113	1342.8081	957s (15.95m)
☑	347	672.4113	1342.8081	954s (15.90m)
☑	348	451.9343	1352.7811	969s (16.15m)
☑	349	451.9343	1352.7811	977s (16.28m)
☑	350	677.3981	1352.7817	1029s (17.15m)
☑	351	677.3981	1352.7817	1025s (17.08m)
☑	352	677.3981	1352.7817	1022s (17.03m)
☑	353	677.3981	1352.7817	1013s (16.88m)
☑	354	677.3981	1352.7817	1016s (16.93m)
☑	355	677.3981	1352.7817	1019s (16.98m)
☑	356	677.3981	1352.7817	1006s (16.77m)
☑	357	677.3981	1352.7817	1000s (16.67m)
☑	358	677.3981	1352.7817	1003s (16.72m)
☑	360	677.8737	1353.7329	907s (15.12m)
☑	361	677.8737	1353.7329	911s (15.18m)
☑	363	685.9109	1369.8072	970s (16.17m)
☑	364	457.6100	1369.8082	1014s (16.90m)
☑	365	457.6100	1369.8082	1011s (16.85m)
☑	366	457.6100	1369.8082	1017s (16.95m)
☑	367	457.6100	1369.8082	1021s (17.02m)
☑	368	457.6100	1369.8082	1004s (16.73m)
☑	369	457.6100	1369.8082	1008s (16.80m)
☑	370	685.9115	1369.8085	966s (16.10m)
☑	373	694.4244	1386.8342	983s (16.38m)
☑	374	694.4244	1386.8342	980s (16.33m)
☑	376	699.4115	1396.8085	996s (16.60m)
☑	377	466.6102	1396.8088	1047s (17.45m)
☑	378	466.6102	1396.8088	1044s (17.40m)
☑	379	466.6102	1396.8088	1018s (16.97m)
☑	380	466.6102	1396.8088	1021s (17.02m)
☑	381	466.6102	1396.8088	1041s (17.35m)
☑	382	466.6102	1396.8088	1037s (17.28m)
☑	383	466.6102	1396.8088	1027s (17.12m)
☑	384	466.6102	1396.8088	1034s (17.23m)
☑	385	466.6102	1396.8088	1031s (17.18m)
☑	386	699.4117	1396.8088	992s (16.53m)
☑	387	466.6105	1396.8095	1011s (16.85m)
☑	388	466.6105	1396.8095	1014s (16.90m)
☑	389	466.6105	1396.8095	1005s (16.75m)

390	466.6105	1396.8095	1008s (16.80m)
391	466.6105	1396.8095	998s (16.63m)
392	466.6105	1396.8095	1002s (16.70m)
395	472.2858	1413.8355	1005s (16.75m)
396	472.2858	1413.8355	999s (16.65m)
400	716.4373	1430.8601	1006s (16.77m)
401	716.4375	1430.8605	1025s (17.08m)
403	721.4234	1440.8323	1042s (17.37m)
404	721.4234	1440.8323	1055s (17.58m)
405	721.4234	1440.8323	1062s (17.70m)
406	721.4234	1440.8323	1045s (17.42m)
407	721.4234	1440.8323	1048s (17.47m)
408	721.4234	1440.8323	1051s (17.52m)
409	721.4234	1440.8323	1059s (17.65m)
410	721.4234	1440.8323	1072s (17.87m)
411	721.4234	1440.8323	1065s (17.75m)
412	481.2852	1440.8337	1015s (16.92m)
413	481.2852	1440.8337	1012s (16.87m)
414	721.4244	1440.8342	1039s (17.32m)
415	721.4244	1440.8342	1029s (17.15m)
416	721.4244	1440.8342	1035s (17.25m)
417	721.4244	1440.8342	1026s (17.10m)
418	721.4244	1440.8342	1031s (17.18m)
419	721.4244	1440.8342	1019s (16.98m)
420	721.4244	1440.8342	1023s (17.05m)
421	722.8169	1443.6192	512s (8.53m)
422	486.9608	1457.8607	1024s (17.07m)
423	486.9608	1457.8607	1028s (17.13m)
424	730.9182	1459.8219	615s (10.25m)
426	489.9126	1466.7159	489s (8.15m)
428	737.9251	1473.8356	660s (11.00m)
430	738.4507	1474.8869	1049s (17.48m)
431	738.4507	1474.8869	1052s (17.53m)
432	743.4373	1484.8600	1085s (18.08m)
433	743.4373	1484.8600	1080s (18.00m)
434	743.4373	1484.8600	1077s (17.95m)
435	495.9607	1484.8604	1041s (17.35m)
436	495.9607	1484.8604	1045s (17.42m)
437	751.9490	1501.8834	1075s (17.92m)
438	751.9490	1501.8834	1080s (18.00m)
439	751.9490	1501.8834	1078s (17.97m)
440	501.6364	1501.8874	1047s (17.45m)
441	501.6364	1501.8874	1050s (17.50m)
445	760.4634	1518.9123	1081s (18.02m)
448	765.4506	1528.8867	1101s (18.35m)
449	765.4506	1528.8867	1094s (18.23m)
450	510.6367	1528.8884	1057s (17.62m)
451	510.6367	1528.8884	1067s (17.78m)
452	765.9582	1529.9018	1096s (18.27m)
454	769.3254	1536.6363	870s (14.50m)
456	771.3249	1540.6352	828s (13.80m)
457	773.9641	1545.9136	1098s (18.30m)
458	516.3119	1545.9138	1068s (17.80m)
459	777.8379	1553.6611	878s (14.63m)
461	779.8494	1557.6843	597s (9.95m)
465	784.8940	1567.7735	850s (14.17m)
466	525.3118	1572.9136	1110s (18.50m)
467	525.3120	1572.9142	1093s (18.22m)
468	787.4644	1572.9143	1120s (18.67m)
469	795.7822	1589.5499	881s (14.68m)
470	795.7822	1589.5499	883s (14.72m)
471	798.8871	1595.7597	819s (13.65m)
473	799.8687	1597.7228	679s (11.32m)
475	805.4175	1608.8205	1162s (19.37m)
476	805.4175	1608.8205	1165s (19.42m)
477	807.3469	1612.6792	881s (14.68m)
478	539.9871	1616.9396	1111s (18.52m)
479	539.9871	1616.9396	1114s (18.57m)
480	817.8281	1633.6416	663s (11.05m)
481	817.8291	1633.6436	646s (10.77m)
482	545.6625	1633.9656	1137s (18.95m)
485	556.2780	1665.8121	487s (8.12m)
486	838.1729	1674.3312	957s (15.95m)
487	849.4457	1696.8767	679s (11.32m)
488	857.4108	1712.8071	758s (12.63m)
489	857.4108	1712.8071	756s (12.60m)
490	867.8613	1733.7081	512s (8.53m)
491	871.4412	1740.8678	964s (16.07m)
492	881.8565	1761.6984	606s (10.10m)
495	882.3572	1762.6998	609s (10.15m)
497	890.9067	1779.7988	785s (13.08m)
499	892.8892	1783.7638	934s (15.57m)
502	900.9282	1799.8419	987s (16.45m)
505	606.3075	1815.9006	831s (13.85m)
506	967.4500	1932.8855	588s (9.80m)

☑	507	967.4500	1932.8855	584s (9.73m)
☑	510	647.6539	1939.9399	788s (13.13m)
☑	511	984.9473	1967.8801	773s (12.88m)
☑	512	984.9473	1967.8801	776s (12.93m)
☑	514	994.0447	1986.0747	1104s (18.40m)
☑	515	514.2380	2052.9229	521s (8.68m)
☑	517	691.3119	2070.9138	484s (8.07m)
☑	518	691.3119	2070.9138	481s (8.02m)
☑	521	426.1317	2125.6220	573s (9.55m)
☑	522	426.8022	2128.9745	573s (9.55m)
☑	523	741.0490	2220.1252	941s (15.68m)
☑	532	807.4205	2419.2396	1260s (21.00m)
☑	533	837.7572	2510.2497	1032s (17.20m)
☑	534	838.0888	2511.2445	1036s (17.27m)
☑	535	972.1758	2913.5055	990s (16.50m)
☑	536	729.3855	2913.5130	983s (16.38m)
☑	537	729.3855	2913.5130	986s (16.43m)
☑	538	729.6318	2914.4981	869s (14.48m)
☑	539	729.6318	2914.4981	867s (14.45m)
☑	540	1032.5454	3094.6143	1217s (20.28m)
☑	541	794.6641	3174.6274	1087s (18.12m)
☑	542	794.6641	3174.6274	1090s (18.17m)
☑	543	1116.5623	3346.6652	1088s (18.13m)
☑	544	1116.5623	3346.6652	1091s (18.18m)
☑	545	837.9209	3347.6544	961s (16.02m)
☑	546	837.9236	3347.6653	1126s (18.77m)
☑	547	1126.8304	4503.2925	1277s (21.28m)
☑	548	904.8657	4519.2919	1229s (20.48m)
☑	549	904.8657	4519.2919	1233s (20.55m)
☑	550	1130.8348	4519.3100	1291s (21.52m)

Search Parameters

```
Type of search         : MS/MS Ion Search
Enzyme                 : Trypsin
Variable modifications : Oxidation (M)
Mass values            : Monoisotopic
Protein Mass           : Unrestricted
Peptide Mass Tolerance : ± 20 ppm
Fragment Mass Tolerance: ± 0.2 Da
Max Missed Cleavages   : 2
Instrument type        : ESI-QUAD-TOF
Number of queries      : 550
```

Mascot Search Results

Host	: 10.10.10.100
User	: alison@anatomy
Email	:
Search title	: CPFP SCH6549 [20/25] april9_2014_alison_af4.mgf
MS data file	: april9_2014_alison_af4.mgf.mascot
Database	: UNIPROT (26617536 sequences; 8639402574 residues)
Taxonomy	: Homo sapiens (human) (132434 sequences)
Timestamp	: 17 Apr 2014 at 00:04:38 GMT
Warning	: There is an error in a configuration file: Error in the configuration file ../config/mascot.dat - database echidna2014small_
Protein hits	: ACTB_HUMAN RecName: Full=Actin, cytoplasmic 1; AltName: Full=Beta-actin; Contains: RecName: Full=Actin, cytoplasmic 1, N-t
	FCGBP_HUMAN RecName: Full=IgGFc-binding protein; AltName: Full=Fcgamma-binding protein antigen; Short=FcgammaBP; Flags: Pre
	HEMO_HUMAN RecName: Full=Hemopexin; AltName: Full=Beta-1B-glycoprotein; Flags: Precursor; - OS=Homo sapiens (Human).
	K22E_HUMAN RecName: Full=Keratin, type II cytoskeletal 2 epidermal; AltName: Full=Cytokeratin-2e; Short=CK-2e; AltName: Fu
	B3KP88_HUMAN SubName: Full=cDNA FLJ31415 fis, clone NT2NE2000284, highly similar to Galectin-3-binding protein; - OS=Homo sa
	B2R853_HUMAN SubName: Full=cDNA, FLJ93744, highly similar to Homo sapiens keratin 6E (KRT6E), mRNA; - OS=Homo sapiens (Human
	K1C10_HUMAN RecName: Full=Keratin, type I cytoskeletal 10; AltName: Full=Cytokeratin-10; Short=CK-10; AltName: Full=Keratin
	H6VRG2_HUMAN SubName: Full=Keratin 1; - OS=Homo sapiens (Human).
	ENOA_HUMAN RecName: Full=Alpha-enolase; EC=4.2.1.11; AltName: Full=2-phospho-D-glycerate hydro-lyase; AltName: Full=C-myc
	A0N4V7_HUMAN SubName: Full=HCG2039797; SubName: Full=Possible J 56 gene segment; Flags: Fragment; - OS=Homo sapiens (Human).
	K2C8_HUMAN RecName: Full=Keratin, type II cytoskeletal 8; AltName: Full=Cytokeratin-8; Short=CK-8; AltName: Full=Keratin-8
	INS_HUMAN RecName: Full=Insulin; Contains: RecName: Full=Insulin B chain; Contains: RecName: Full=Insulin A chain; Flags:
	POTEE_HUMAN RecName: Full=POTE ankyrin domain family member E; AltName: Full=ANKRD26-like family C member 1A; AltName: Full
	Q0QET7_HUMAN RecName: Full=Glyceraldehyde-3-phosphate dehydrogenase; EC=1.2.1.12; Flags: Fragment; - OS=Homo sapiens (Human)
	Q8N6K3_HUMAN SubName: Full=SNX25 protein; - OS=Homo sapiens (Human).
	H0YB18_HUMAN SubName: Full=Putative hydroxypyruvate isomerase; Flags: Fragment; - OS=Homo sapiens (Human).
	WNT1_HUMAN RecName: Full=Proto-oncogene Wnt-1; AltName: Full=Proto-oncogene Int-1 homolog; Flags: Precursor; - OS=Homo sap
	B2R747_HUMAN SubName: Full=cDNA, FLJ93281, highly similar to Homo sapiens calcium/calmodulin-dependent protein kinase IV (CA
	IGHG1_HUMAN RecName: Full=Ig gamma-1 chain C region; - OS=Homo sapiens (Human).
	AHNK2_HUMAN RecName: Full=Protein AHNAK2; - OS=Homo sapiens (Human).

	UNIPROT	Decoy	False discovery rate
Peptide matches above identity threshold	34	2	5.88 %
Peptide matches above homology or identity threshold	48	13	27.08 %

Mascot Score Histogram

Ions score is -10*Log(P), where P is the probability that the observed match is a random event.
Individual ions scores > 30 indicate identity or extensive homology (p<0.05).
Protein scores are derived from ions scores as a non-probabilistic basis for ranking protein hits.

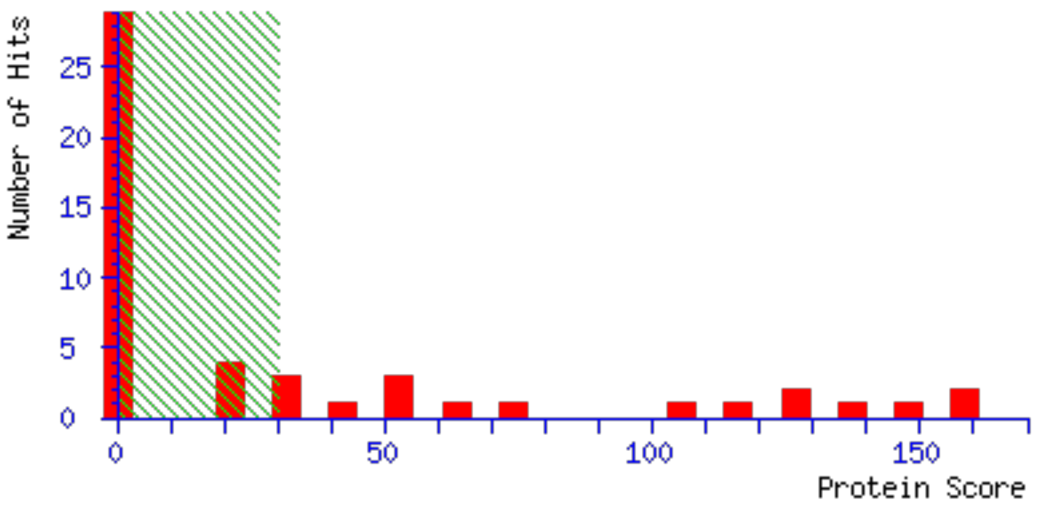

Peptide Summary Report

Format As	Peptide Summary		Help
	Significance threshold p< 0.05	Max. number of hits 20	Show Percolator scores ☐
	Standard scoring ○ MudPIT scoring ⦿	Ions score or expect cut-off 0	Show sub-sets 0
	Show pop-ups ⦿ Suppress pop-ups ○	Sort unassigned Decreasing Score	Require bold red ☐
	Preferred taxonomy All entries		

1. ACTB_HUMAN **Mass:** 41710 **Score:** 158 **Matches:** 5(5) **Sequences:** 4(4) **emPAI:** 0.36
RecName: Full=Actin, cytoplasmic 1; AltName: Full=Beta-actin; Contains: RecName: Full=Actin, cytoplasmic 1, N-terminally processed; - OS
☐ Check to include this hit in error tolerant search or archive report

	Query	Observed	Mr(expt)	Mr(calc)	ppm	Miss	Score	Expect	Rank	Unique	Retention Time	Peptide
☑	36	398.2380	794.4615	794.4650	-4.39	0	39	0.0041	1	U	482s (8.03m)	K.IIAPPER.K
☑	222	566.7690	1131.5234	1131.5197	3.28	0	55	0.00015	1	U	512s (8.53m)	R.GYSFTTTAER.E
☑	240	581.3143	1160.6141	1160.6111	2.62	0	66	2.4e-05	1	U	572s (9.53m)	K.EITALAPSTMK.I
☑	241	581.3143	1160.6141	1160.6111	2.62	0	(39)	0.011	1	U	576s (9.60m)	K.EITALAPSTMK.I
☑	380	506.2408	1515.7007	1515.6954	3.51	0	48	0.00072	1		476s (7.93m)	K.QEYDESGPSIVHR.K

Proteins matching the same set of peptides:
ACTG_HUMAN **Mass:** 41766 **Score:** 158 **Matches:** 5(5) **Sequences:** 4(4)
RecName: Full=Actin, cytoplasmic 2; AltName: Full=Gamma-actin; Contains: RecName: Full=Actin, cytoplasmic 2, N-terminally processed; - O
Q1KLZ0_HUMAN **Mass:** 41710 **Score:** 158 **Matches:** 5(5) **Sequences:** 4(4)
SubName: Full=HCG15971, isoform CRA_a; SubName: Full=PS1TP5-binding protein 1; - OS=Homo sapiens (Human).
B3KWQ3_HUMAN **Mass:** 28193 **Score:** 158 **Matches:** 5(5) **Sequences:** 4(4)
SubName: Full=cDNA FLJ43573 fis, clone RECTM2001691, highly similar to Actin, cytoplasmic 2; - OS=Homo sapiens (Human).
B4DVQ0_HUMAN **Mass:** 37325 **Score:** 158 **Matches:** 5(5) **Sequences:** 4(4)
SubName: Full=cDNA FLJ58286, highly similar to Actin, cytoplasmic 2; - OS=Homo sapiens (Human).

B4DW52_HUMAN **Mass:** 38608 **Score:** 158 **Matches:** 5(5) **Sequences:** 4(4)
SubName: Full=cDNA FLJ55253, highly similar to Actin, cytoplasmic 1; - OS=Homo sapiens (Human).
B4E335_HUMAN **Mass:** 39201 **Score:** 158 **Matches:** 5(5) **Sequences:** 4(4)
SubName: Full=cDNA FLJ52842, highly similar to Actin, cytoplasmic 1; - OS=Homo sapiens (Human).
B4E3A4_HUMAN **Mass:** 39774 **Score:** 158 **Matches:** 5(5) **Sequences:** 4(4)
SubName: Full=cDNA FLJ57283, highly similar to Actin, cytoplasmic 2; - OS=Homo sapiens (Human).
Q6PJ43_HUMAN **Mass:** 29393 **Score:** 158 **Matches:** 5(5) **Sequences:** 4(4)
SubName: Full=ACTG1 protein; Flags: Fragment; - OS=Homo sapiens (Human).
B7ZAP6_HUMAN **Mass:** 33080 **Score:** 158 **Matches:** 5(5) **Sequences:** 4(4)
SubName: Full=cDNA, FLJ79260, highly similar to Actin, cytoplasmic 2; - OS=Homo sapiens (Human).
Q8WVW5_HUMAN **Mass:** 40477 **Score:** 158 **Matches:** 5(5) **Sequences:** 4(4)
SubName: Full=Putative uncharacterized protein; Flags: Fragment; - OS=Homo sapiens (Human).
Q53G76_HUMAN **Mass:** 41694 **Score:** 158 **Matches:** 5(5) **Sequences:** 4(4)
SubName: Full=Beta actin variant; Flags: Fragment; - OS=Homo sapiens (Human).
Q53G99_HUMAN **Mass:** 41738 **Score:** 158 **Matches:** 5(5) **Sequences:** 4(4)
SubName: Full=Beta actin variant; Flags: Fragment; - OS=Homo sapiens (Human).
Q53GK6_HUMAN **Mass:** 41696 **Score:** 158 **Matches:** 5(5) **Sequences:** 4(4)
SubName: Full=Beta actin variant; Flags: Fragment; - OS=Homo sapiens (Human).

---

2. FCGBP_HUMAN **Mass:** 571639 **Score:** 154 **Matches:** 6(5) **Sequences:** 4(4) **emPAI:** 0.02
RecName: Full=IgGFc-binding protein; AltName: Full=Fcgamma-binding protein antigen; Short=FcgammaBP; Flags: Precursor; - OS=Homo sapiens
☐ Check to include this hit in error tolerant search or archive report

	Query	Observed	Mr(expt)	Mr(calc)	ppm	Miss	Score	Expect	Rank	Unique	Retention Time	Peptide
☑	125	481.7563	961.4981	961.4981	-0.02	0	38	0.015	1	U	489s (8.15m)	R.GNPAVSYVR.V
☑	126	481.7563	961.4981	961.4981	-0.02	0	(36)	0.023	1	U	487s (8.12m)	R.GNPAVSYVR.V
☑	167	519.2754	1036.5361	1036.5342	1.90	0	(27)	0.15	1	U	636s (10.60m)	K.FYPAGDVLR.V
☑	168	519.2754	1036.5361	1036.5342	1.90	0	32	0.046	1	U	632s (10.53m)	K.FYPAGDVLR.V
☑	349	705.3874	1408.7602	1408.7674	-5.11	0	63	2.9e-05	1	U	576s (9.60m)	K.AISGLTIDGHAVGAK.L
☑	357	712.8547	1423.6949	1423.6943	0.44	0	64	3.9e-05	1	U	536s (8.93m)	R.GATTSPGVYELSSR.C

---

3. HEMO_HUMAN **Mass:** 51643 **Score:** 144 **Matches:** 11(5) **Sequences:** 6(3) **emPAI:** 0.28
RecName: Full=Hemopexin; AltName: Full=Beta-1B-glycoprotein; Flags: Precursor; - OS=Homo sapiens (Human).
☐ Check to include this hit in error tolerant search or archive report

	Query	Observed	Mr(expt)	Mr(calc)	ppm	Miss	Score	Expect	Rank	Unique	Retention Time	Peptide
☑	25	383.2005	764.3864	764.3857	0.89	0	(18)	1	1	U	622s (10.37m)	K.GEFVWK.S
☑	26	383.2005	764.3864	764.3857	0.89	0	30	0.067	1	U	617s (10.28m)	K.GEFVWK.S
☑	37	399.2260	796.4374	796.4378	-0.49	0	28	0.057	1	U	478s (7.97m)	R.LHIMAGR.R
☑	128	487.2800	972.5454	972.5433	2.19	0	24	0.35	1	U	1209s (20.15m)	R.LWWLDLK.S
☑	129	487.2800	972.5454	972.5433	2.19	0	(23)	0.5	1	U	1206s (20.10m)	R.LWWLDLK.S
☑	219	565.3313	1128.6481	1128.6444	3.29	1	(29)	0.087	1	U	1055s (17.58m)	R.RLWWLDLK.S
☑	220	565.3313	1128.6481	1128.6444	3.29	1	29	0.076	1	U	1051s (17.52m)	R.RLWWLDLK.S
☑	227	571.2997	1140.5848	1140.5815	2.84	0	60	7.3e-05	1	U	578s (9.63m)	K.GGYTLVSGYPK.R
☑	228	571.2997	1140.5848	1140.5815	2.84	0	(60)	7.5e-05	1	U	575s (9.58m)	K.GGYTLVSGYPK.R
☑	269	610.8079	1219.6012	1219.5986	2.18	0	(51)	0.00064	1	U	730s (12.17m)	K.NFPSPVDAAFR.Q
☑	270	610.8079	1219.6012	1219.5986	2.18	0	58	0.00012	1	U	733s (12.22m)	K.NFPSPVDAAFR.Q

---

4. K22E_HUMAN **Mass:** 65393 **Score:** 138 **Matches:** 4(4) **Sequences:** 4(4) **emPAI:** 0.22
RecName: Full=Keratin, type II cytoskeletal 2 epidermal; AltName: Full=Cytokeratin-2e; Short=CK-2e; AltName: Full=Epithelial keratin-2e;
☐ Check to include this hit in error tolerant search or archive report

	Query	Observed	Mr(expt)	Mr(calc)	ppm	Miss	Score	Expect	Rank	Unique	Retention Time	Peptide
☑	202	554.2759	1106.5373	1106.5356	1.51	0	45	0.0023	1		468s (7.80m)	K.AQYEEIAQR.S
☑	263	604.8109	1207.6073	1207.6085	-0.99	0	61	9.3e-05	1	U	591s (9.85m)	R.TAAENDFVTLK.K
☑	320	665.3682	1328.7218	1328.7187	2.34	0	50	0.0015	1		1105s (18.42m)	R.NLDLDSIIAEVK.A
☑	368	738.3950	1474.7755	1474.7780	-1.67	0	52	0.00088	1		542s (9.03m)	R.FLEQQNQVLQTK.W

---

5. B3KP88_HUMAN **Mass:** 54136 **Score:** 132 **Matches:** 2(2) **Sequences:** 1(1) **emPAI:** 0.06
SubName: Full=cDNA FLJ31415 fis, clone NT2NE2000284, highly similar to Galectin-3-binding protein; - OS=Homo sapiens (Human).
☐ Check to include this hit in error tolerant search or archive report

	Query	Observed	Mr(expt)	Mr(calc)	ppm	Miss	Score	Expect	Rank	Unique	Retention Time	Peptide
☑	38	799.8423	1597.6701	1597.6685	1.01	0	(61)	9.8e-06	1	U	596s (9.93m)	K.YSSDYFQAPSDYR.Y
☑	408	799.8431	1597.6717	1597.6685	2.00	0	90	1e-08	1	U	592s (9.87m)	K.YSSDYFQAPSDYR.Y

Proteins matching the same set of peptides:
LG3BP_HUMAN **Mass:** 65289 **Score:** 132 **Matches:** 2(2) **Sequences:** 1(1)
RecName: Full=Galectin-3-binding protein; AltName: Full=Basement membrane autoantigen p105; AltName: Full=Lectin galactoside-binding sol
B4DDG4_HUMAN **Mass:** 54648 **Score:** 132 **Matches:** 2(2) **Sequences:** 1(1)
SubName: Full=cDNA FLJ54583, highly similar to Galectin-3-binding protein; - OS=Homo sapiens (Human).
B4DI70_HUMAN **Mass:** 46391 **Score:** 132 **Matches:** 2(2) **Sequences:** 1(1)
SubName: Full=cDNA FLJ53509, highly similar to Galectin-3-binding protein; - OS=Homo sapiens (Human).
B4DVE1_HUMAN **Mass:** 64056 **Score:** 132 **Matches:** 2(2) **Sequences:** 1(1)
SubName: Full=cDNA FLJ53478, highly similar to Galectin-3-binding protein; - OS=Homo sapiens (Human).
B4DWA8_HUMAN **Mass:** 52365 **Score:** 132 **Matches:** 2(2) **Sequences:** 1(1)
SubName: Full=cDNA FLJ53427, highly similar to Galectin-3-binding protein; - OS=Homo sapiens (Human).

---

6. B2R853_HUMAN **Mass:** 59990 **Score:** 126 **Matches:** 4(3) **Sequences:** 4(3) **emPAI:** 0.17
SubName: Full=cDNA, FLJ93744, highly similar to Homo sapiens keratin 6E (KRT6E), mRNA; - OS=Homo sapiens (Human).
☐ Check to include this hit in error tolerant search or archive report

	Query	Observed	Mr(expt)	Mr(calc)	ppm	Miss	Score	Expect	Rank	Unique	Retention Time	Peptide
	202	554.2759	1106.5373	1106.5356	1.51	0	45	0.0023	1		468s (7.80m)	K.AQYEEIAQR.S
	260	602.3337	1202.6528	1202.6295	19.4	0	6	21	6	U	799s (13.32m)	K.WTLLQEQGTK.T
	320	665.3682	1328.7218	1328.7187	2.34	0	50	0.0015	1		1105s (18.42m)	R.NLDLDSIIAEVK.A
☑	347	704.3587	1406.7027	1406.7041	-0.99	0	80	8.6e-07	1	U	911s (15.18m)	K.ADTLTDEINFLR.A

Proteins matching the same set of peptides:

K2C6A_HUMAN **Mass:** 60008 **Score:** 126 **Matches:** 4(3) **Sequences:** 4(3)
RecName: Full=Keratin, type II cytoskeletal 6A; AltName: Full=Cytokeratin-6A; Short=CK-6A; AltName: Full=Cytokeratin-6D; Short=CK-6D; Al
K2C6B_HUMAN **Mass:** 60030 **Score:** 126 **Matches:** 4(3) **Sequences:** 4(3)
RecName: Full=Keratin, type II cytoskeletal 6B; AltName: Full=Cytokeratin-6B; Short=CK-6B; AltName: Full=Keratin-6B; Short=K6B; AltName:
K2C6C_HUMAN **Mass:** 59988 **Score:** 126 **Matches:** 4(3) **Sequences:** 4(3)
RecName: Full=Keratin, type II cytoskeletal 6C; AltName: Full=Cytokeratin-6C; Short=CK-6C; AltName: Full=Cytokeratin-6E; Short=CK-6E; Al
B4DRR0_HUMAN **Mass:** 57803 **Score:** 126 **Matches:** 4(3) **Sequences:** 4(3)
SubName: Full=cDNA FLJ53910, highly similar to Keratin, type II cytoskeletal 6A; - OS=Homo sapiens (Human).
B4DRY0_HUMAN **Mass:** 58643 **Score:** 126 **Matches:** 4(3) **Sequences:** 4(3)
SubName: Full=cDNA FLJ54379, highly similar to Keratin, type II cytoskeletal 6E; - OS=Homo sapiens (Human).
B4DWU6_HUMAN **Mass:** 55765 **Score:** 126 **Matches:** 4(3) **Sequences:** 4(3)
SubName: Full=cDNA FLJ51361, highly similar to Keratin, type II cytoskeletal 6A; - OS=Homo sapiens (Human).
A8K2I0_HUMAN **Mass:** 59994 **Score:** 126 **Matches:** 4(3) **Sequences:** 4(3)
SubName: Full=cDNA FLJ78504, highly similar to Homo sapiens keratin 6A (KRT6A), mRNA; - OS=Homo sapiens (Human).

---

7. K1C10_HUMAN **Mass:** 58792 **Score:** 118 **Matches:** 5(3) **Sequences:** 4(2) **emPAI:** 0.18
RecName: Full=Keratin, type I cytoskeletal 10; AltName: Full=Cytokeratin-10; Short=CK-10; AltName: Full=Keratin-10; Short=K10; - OS=Homo
☐ Check to include this hit in error tolerant search or archive report

	Query	Observed	Mr(expt)	Mr(calc)	ppm	Miss	Score	Expect	Rank	Unique	Retention Time	Peptide
☑	12	497.2594	992.5042	992.4927	11.6	0	32	0.12	1	U	491s (8.18m)	K.YENEVALR.Q
☑	164	516.3033	1030.5920	1030.5910	1.00	0	(51)	0.0005	1	U	635s (10.58m)	R.VLDELTLTK.A
☑	165	516.3033	1030.5920	1030.5910	1.00	0	53	0.00032	1	U	631s (10.52m)	R.VLDELTLTK.A
☑	340	695.8468	1389.6789	1389.6736	3.88	0	11	6.6	1	U	573s (9.55m)	K.QSLEASLAETEGR.Y
☑	370	498.5813	1492.7221	1492.7270	-3.28	1	54	0.00045	1	U	452s (7.53m)	R.SQYEQLAEQNRK.D

---

8. H6VRG2_HUMAN **Mass:** 66026 **Score:** 109 **Matches:** 3(3) **Sequences:** 3(3) **emPAI:** 0.16
SubName: Full=Keratin 1; - OS=Homo sapiens (Human).
☐ Check to include this hit in error tolerant search or archive report

	Query	Observed	Mr(expt)	Mr(calc)	ppm	Miss	Score	Expect	Rank	Unique	Retention Time	Peptide
☑	81	437.7538	873.4930	873.4920	1.16	0	57	0.0002	1	U	537s (8.95m)	R.SLVNLGGSK.S
	320	665.3682	1328.7218	1328.7187	2.34	0	50	0.0015	1		1105s (18.42m)	R.NLDLDSIIAEVK.A
	368	738.3950	1474.7755	1474.7780	-1.67	0	52	0.00088	1		542s (9.03m)	R.FLEQQNQVLQTK.W

---

9. ENOA_HUMAN **Mass:** 47139 **Score:** 70 **Matches:** 1(1) **Sequences:** 1(1) **emPAI:** 0.07
RecName: Full=Alpha-enolase; EC=4.2.1.11; AltName: Full=2-phospho-D-glycerate hydro-lyase; AltName: Full=C-myc promoter-binding protein;
☐ Check to include this hit in error tolerant search or archive report

	Query	Observed	Mr(expt)	Mr(calc)	ppm	Miss	Score	Expect	Rank	Unique	Retention Time	Peptide
☑	358	713.3678	1424.7211	1424.7187	1.69	0	70	8.3e-06	1	U	819s (13.65m)	R.YISPDQLADLYK.S

Proteins matching the same set of peptides:

E2DRY6_HUMAN **Mass:** 36532 **Score:** 70 **Matches:** 1(1) **Sequences:** 1(1)
RecName: Full=Enolase; EC=4.2.1.11; - OS=Homo sapiens (Human).
Q9BT62_HUMAN **Mass:** 29864 **Score:** 70 **Matches:** 1(1) **Sequences:** 1(1)
RecName: Full=Enolase; EC=4.2.1.11; Flags: Fragment; - OS=Homo sapiens (Human).
Q96GV1_HUMAN **Mass:** 20402 **Score:** 70 **Matches:** 1(1) **Sequences:** 1(1)
RecName: Full=Enolase; EC=4.2.1.11; Flags: Fragment; - OS=Homo sapiens (Human).

---

10. A0N4V7_HUMAN **Mass:** 2212 **Score:** 59 **Matches:** 9(7) **Sequences:** 1(1) **emPAI:** 1.81
SubName: Full=HCG2039797; SubName: Full=Possible J 56 gene segment; Flags: Fragment; - OS=Homo sapiens (Human).
☐ Check to include this hit in error tolerant search or archive report

	Query	Observed	Mr(expt)	Mr(calc)	ppm	Miss	Score	Expect	Rank	Unique	Retention Time	Peptide
☑	55	421.7581	841.5016	841.5022	-0.60	0	(30)	0.053	1	U	498s (8.30m)	K.GITLSVRP.-
☑	56	421.7581	841.5016	841.5022	-0.60	0	(30)	0.051	1	U	495s (8.25m)	K.GITLSVRP.-
☑	57	421.7584	841.5022	841.5022	0.12	0	33	0.024	1	U	688s (11.47m)	K.GITLSVRP.-
☑	58	421.7584	841.5022	841.5022	0.12	0	(30)	0.048	1	U	684s (11.40m)	K.GITLSVRP.-
☑	59	421.7585	841.5025	841.5022	0.38	0	(30)	0.051	1	U	589s (9.82m)	K.GITLSVRP.-
☑	60	421.7585	841.5025	841.5022	0.38	0	(30)	0.052	1	U	591s (9.85m)	K.GITLSVRP.-
☑	61	421.7587	841.5028	841.5022	0.78	0	(27)	0.097	1	U	792s (13.20m)	K.GITLSVRP.-
☑	62	421.7587	841.5029	841.5022	0.90	0	(32)	0.035	1	U	795s (13.25m)	K.GITLSVRP.-
☑	63	421.7590	841.5035	841.5022	1.64	0	(27)	0.12	1	U	886s (14.77m)	K.GITLSVRP.-

---

11. K2C8_HUMAN **Mass:** 53671 **Score:** 57 **Matches:** 3(1) **Sequences:** 2(1) **emPAI:** 0.06
RecName: Full=Keratin, type II cytoskeletal 8; AltName: Full=Cytokeratin-8; Short=CK-8; AltName: Full=Keratin-8; Short=K8; AltName: Full
☐ Check to include this hit in error tolerant search or archive report

	Query	Observed	Mr(expt)	Mr(calc)	ppm	Miss	Score	Expect	Rank	Unique	Retention Time	Peptide
	143	500.7892	999.5638	999.5600	3.76	0	(2)	58	2	U	1041s (17.35m)	R.LQAEIEGLK.G
	144	500.7892	999.5638	999.5600	3.76	0	3	49	2	U	1038s (17.30m)	R.LQAEIEGLK.G
☑	19	565.3137	1128.6128	1128.6138	-0.90	0	57	0.0003	1	U	639s (10.65m)	K.LSELEAALQR.A

Proteins matching the same set of peptides:
Q7L4M3_HUMAN **Mass:** 30802 **Score:** 57 **Matches:** 3(1) **Sequences:** 2(1)
SubName: Full=KRT8 protein; - OS=Homo sapiens (Human).
F8VXB4_HUMAN **Mass:** 56573 **Score:** 57 **Matches:** 3(1) **Sequences:** 2(1)
SubName: Full=Keratin, type II cytoskeletal 8; - OS=Homo sapiens (Human).
Q969I0_HUMAN **Mass:** 41083 **Score:** 57 **Matches:** 3(1) **Sequences:** 2(1)
SubName: Full=KRT8 protein; Flags: Fragment; - OS=Homo sapiens (Human).

---

12. INS_HUMAN **Mass:** 11973 **Score:** 54 **Matches:** 2(2) **Sequences:** 1(1) **emPAI:** 0.29
RecName: Full=Insulin; Contains: RecName: Full=Insulin B chain; Contains: RecName: Full=Insulin A chain; Flags: Precursor; - OS=Homo sap
Check to include this hit in error tolerant search or archive report

Query	Observed	Mr(expt)	Mr(calc)	ppm	Miss	Score	Expect	Rank	Unique	Retention Time	Peptide
69	430.2216	858.4287	858.4276	1.25	0	46	0.0018	1	U	644s (10.73m)	R.GFFYTPK.T
70	430.2216	858.4287	858.4276	1.25	0	(33)	0.035	1	U	647s (10.78m)	R.GFFYTPK.T

Proteins matching the same set of peptides:
Q1WM24_HUMAN **Mass:** 21508 **Score:** 54 **Matches:** 2(2) **Sequences:** 1(1)
SubName: Full=INSIGF long transcript variant; SubName: Full=INSIGF short transcript variant; - OS=Homo sapiens (Human).
I3WAC9_HUMAN **Mass:** 11973 **Score:** 54 **Matches:** 2(2) **Sequences:** 1(1)
SubName: Full=Preproinsulin; - OS=Homo sapiens (Human).
A6XGL2_HUMAN **Mass:** 10952 **Score:** 54 **Matches:** 2(2) **Sequences:** 1(1)
SubName: Full=Insulin; SubName: Full=Insulin A chain; - OS=Homo sapiens (Human).
F6MZK5_HUMAN **Mass:** 10154 **Score:** 54 **Matches:** 2(2) **Sequences:** 1(1)
SubName: Full=Insulin; Flags: Precursor; Fragment; - OS=Homo sapiens (Human).
F8WCM5_HUMAN **Mass:** 21524 **Score:** 54 **Matches:** 2(2) **Sequences:** 1(1)
SubName: Full=Protein INS-IGF2; - OS=Homo sapiens (Human).
C9JNR5_HUMAN **Mass:** 10625 **Score:** 54 **Matches:** 2(2) **Sequences:** 1(1)
SubName: Full=Insulin A chain; Flags: Fragment; - OS=Homo sapiens (Human).

---

13. POTEE_HUMAN **Mass:** 121286 **Score:** 48 **Matches:** 2(1) **Sequences:** 2(1) **emPAI:** 0.03
RecName: Full=POTE ankyrin domain family member E; AltName: Full=ANKRD26-like family C member 1A; AltName: Full=Prostate, ovary, testis-
Check to include this hit in error tolerant search or archive report

Query	Observed	Mr(expt)	Mr(calc)	ppm	Miss	Score	Expect	Rank	Unique	Retention Time	Peptide
202	554.2759	1106.5373	1106.5277	8.61	0	7	14	2	U	468s (7.80m)	K.MSQELEINK.D + Oxidation (M)
380	506.2408	1515.7007	1515.6954	3.51	0	48	0.00072	1		476s (7.93m)	K.QEYDESGPSIVHR.K

---

14. Q0QET7_HUMAN **Mass:** 24605 **Score:** 45 **Matches:** 1(1) **Sequences:** 1(1) **emPAI:** 0.14
RecName: Full=Glyceraldehyde-3-phosphate dehydrogenase; EC=1.2.1.12; Flags: Fragment; - OS=Homo sapiens (Human).
Check to include this hit in error tolerant search or archive report

Query	Observed	Mr(expt)	Mr(calc)	ppm	Miss	Score	Expect	Rank	Unique	Retention Time	Peptide
352	706.3956	1410.7767	1410.7831	-4.48	0	45	0.0021	1	U	655s (10.92m)	R.GALQNIIPASTGAAK.A

Proteins matching the same set of peptides:
Q2TSD0_HUMAN **Mass:** 36026 **Score:** 45 **Matches:** 1(1) **Sequences:** 1(1)
RecName: Full=Glyceraldehyde-3-phosphate dehydrogenase; EC=1.2.1.12; - OS=Homo sapiens (Human).
G3P_HUMAN **Mass:** 36030 **Score:** 45 **Matches:** 1(1) **Sequences:** 1(1)
RecName: Full=Glyceraldehyde-3-phosphate dehydrogenase; Short=GAPDH; EC=1.2.1.12; AltName: Full=Peptidyl-cysteine S-nitrosylase GAPDH; E
A4UCT1_HUMAN **Mass:** 17292 **Score:** 45 **Matches:** 1(1) **Sequences:** 1(1)
RecName: Full=Glyceraldehyde-3-phosphate dehydrogenase; EC=1.2.1.12; Flags: Fragment; - OS=Homo sapiens (Human).
Q5ZEY3_HUMAN **Mass:** 9195 **Score:** 45 **Matches:** 1(1) **Sequences:** 1(1)
SubName: Full=Glyceraldehyde-3-phosphate dehydrogenase; EC=1.2.1.12; Flags: Fragment; - OS=Homo sapiens (Human).
E7EUT4_HUMAN **Mass:** 31528 **Score:** 45 **Matches:** 1(1) **Sequences:** 1(1)
RecName: Full=Glyceraldehyde-3-phosphate dehydrogenase; EC=1.2.1.12; - OS=Homo sapiens (Human).
E7EUT5_HUMAN **Mass:** 27853 **Score:** 45 **Matches:** 1(1) **Sequences:** 1(1)
RecName: Full=Glyceraldehyde-3-phosphate dehydrogenase; EC=1.2.1.12; - OS=Homo sapiens (Human).

---

15. Q8N6K3_HUMAN **Mass:** 64718 **Score:** 30 **Matches:** 2(1) **Sequences:** 1(1) **emPAI:** 0.05
SubName: Full=SNX25 protein; - OS=Homo sapiens (Human).
Check to include this hit in error tolerant search or archive report

Query	Observed	Mr(expt)	Mr(calc)	ppm	Miss	Score	Expect	Rank	Unique	Retention Time	Peptide
110	471.7899	941.5653	941.5770	-12.45	2	(25)	0.18	1	U	612s (10.20m)	M.KADLLRAR.N
111	471.7899	941.5653	941.5770	-12.45	2	30	0.051	1	U	609s (10.15m)	M.KADLLRAR.N

---

16. H0YB18_HUMAN **Mass:** 17688 **Score:** 28 **Matches:** 1(1) **Sequences:** 1(1) **emPAI:** 0.19
SubName: Full=Putative hydroxypyruvate isomerase; Flags: Fragment; - OS=Homo sapiens (Human).
Check to include this hit in error tolerant search or archive report

Query	Observed	Mr(expt)	Mr(calc)	ppm	Miss	Score	Expect	Rank	Unique	Retention Time	Peptide
37	399.2260	796.4374	796.4378	-0.49	0	28	0.057	1	U	478s (7.97m)	R.IHLMAGR.V

Proteins matching the same set of peptides:
H0YB70_HUMAN **Mass:** 20737 **Score:** 28 **Matches:** 1(1) **Sequences:** 1(1)
SubName: Full=Putative hydroxypyruvate isomerase; Flags: Fragment; - OS=Homo sapiens (Human).
HYI_HUMAN **Mass:** 30387 **Score:** 28 **Matches:** 1(1) **Sequences:** 1(1)

RecName: Full=Putative hydroxypyruvate isomerase; EC=5.3.1.22; AltName: Full=Endothelial cell apoptosis protein E-CE1; - OS=Homo sapiens
G4XUV3_HUMAN Mass: 23964 Score: 28 Matches: 1(1) Sequences: 1(1)
SubName: Full=Hydroxypyruvate isomerase; - OS=Homo sapiens (Human).
Q5T014_HUMAN Mass: 18112 Score: 28 Matches: 1(1) Sequences: 1(1)
SubName: Full=Hydroxypyruvate isomerase homolog (E. coli); - OS=Homo sapiens (Human).
Q5T017_HUMAN Mass: 20226 Score: 28 Matches: 1(1) Sequences: 1(1)
SubName: Full=Hydroxypyruvate isomerase homolog (E. coli); SubName: Full=cDNA FLJ38925 fis, clone NT2NE2012243, highly similar to Homo s
F6UJY9_HUMAN Mass: 23128 Score: 28 Matches: 1(1) Sequences: 1(1)
SubName: Full=Putative hydroxypyruvate isomerase; Flags: Fragment; - OS=Homo sapiens (Human).
E7EWH8_HUMAN Mass: 30330 Score: 28 Matches: 1(1) Sequences: 1(1)
SubName: Full=Putative hydroxypyruvate isomerase; - OS=Homo sapiens (Human).

---

17. WNT1_HUMAN Mass: 40955 Score: 26 Matches: 2(0) Sequences: 1(0) emPAI: 0.08
RecName: Full=Proto-oncogene Wnt-1; AltName: Full=Proto-oncogene Int-1 homolog; Flags: Precursor; - OS=Homo sapiens (Human).
☐ Check to include this hit in error tolerant search or archive report

	Query	Observed	Mr(expt)	Mr(calc)	ppm	Miss	Score	Expect	Rank	Unique	Retention Time	Peptide
☑	143	500.7892	999.5638	999.5461	17.7	1	(13)	4.4	1	U	1041s (17.35m)	R.AVGDVLRDR.F
☑	144	500.7892	999.5638	999.5461	17.7	1	26	0.21	1	U	1038s (17.30m)	R.AVGDVLRDR.F

---

18. B2R747_HUMAN Mass: 51907 Score: 25 Matches: 1(0) Sequences: 1(0) emPAI: 0.06
SubName: Full=cDNA, FLJ93281, highly similar to Homo sapiens calcium/calmodulin-dependent protein kinase IV (CAMK4), mRNA; - OS=Homo sap
☐ Check to include this hit in error tolerant search or archive report

	Query	Observed	Mr(expt)	Mr(calc)	ppm	Miss	Score	Expect	Rank	Unique	Retention Time	Peptide
☑	250	**592.8359**	**1183.6573**	**1183.6561**	**1.03**	**1**	**25**	**0.17**	**1**	**U**	**586s (9.77m)**	**K.LKTVEEAAAPR.E**

Proteins matching the same set of peptides:
KCC4_HUMAN Mass: 51893 Score: 25 Matches: 1(0) Sequences: 1(0)
RecName: Full=Calcium/calmodulin-dependent protein kinase type IV; Short=CaMK IV; EC=2.7.11.17; AltName: Full=CaM kinase-GR; - OS=Homo s

---

19. IGHG1_HUMAN Mass: 36083 Score: 20 Matches: 1(0) Sequences: 1(0) emPAI: 0.09
RecName: Full=Ig gamma-1 chain C region; - OS=Homo sapiens (Human).
☐ Check to include this hit in error tolerant search or archive report

	Query	Observed	Mr(expt)	Mr(calc)	ppm	Miss	Score	Expect	Rank	Unique	Retention Time	Peptide
☑	471	**937.4695**	**1872.9245**	**1872.9146**	**5.29**	**0**	**20**	**0.73**	**1**	**U**	**957s (15.95m)**	**K.TTPPVLDSDGSFFLYSK.L**

Proteins matching the same set of peptides:
Q5EFE5_HUMAN Mass: 52329 Score: 20 Matches: 1(0) Sequences: 1(0)
SubName: Full=Anti-RhD monoclonal T125 gamma1 heavy chain; Flags: Precursor; - OS=Homo sapiens (Human).
Q6GMX6_HUMAN Mass: 51051 Score: 20 Matches: 1(0) Sequences: 1(0)
SubName: Full=IGH@ protein; - OS=Homo sapiens (Human).
Q6MZQ6_HUMAN Mass: 52010 Score: 20 Matches: 1(0) Sequences: 1(0)
SubName: Full=Putative uncharacterized protein DKFZp686G11190; - OS=Homo sapiens (Human).
Q6MZV7_HUMAN Mass: 52088 Score: 20 Matches: 1(0) Sequences: 1(0)
SubName: Full=Putative uncharacterized protein DKFZp686C11235; - OS=Homo sapiens (Human).
Q6N089_HUMAN Mass: 51692 Score: 20 Matches: 1(0) Sequences: 1(0)
SubName: Full=Putative uncharacterized protein DKFZp686P15220; - OS=Homo sapiens (Human).
Q6N094_HUMAN Mass: 52579 Score: 20 Matches: 1(0) Sequences: 1(0)
SubName: Full=Putative uncharacterized protein DKFZp686O01196; - OS=Homo sapiens (Human).
Q6N095_HUMAN Mass: 52327 Score: 20 Matches: 1(0) Sequences: 1(0)
SubName: Full=Putative uncharacterized protein DKFZp686K03196; - OS=Homo sapiens (Human).
Q6N096_HUMAN Mass: 50895 Score: 20 Matches: 1(0) Sequences: 1(0)
SubName: Full=Putative uncharacterized protein DKFZp686I15196; - OS=Homo sapiens (Human).
Q6N097_HUMAN Mass: 52725 Score: 20 Matches: 1(0) Sequences: 1(0)
SubName: Full=Putative uncharacterized protein DKFZp686H20196; - OS=Homo sapiens (Human).
Q6PYX1_HUMAN Mass: 38138 Score: 20 Matches: 1(0) Sequences: 1(0)
SubName: Full=Hepatitis B virus receptor binding protein; Flags: Fragment; - OS=Homo sapiens (Human).
Q7Z351_HUMAN Mass: 52819 Score: 20 Matches: 1(0) Sequences: 1(0)
SubName: Full=Putative uncharacterized protein DKFZp686N02209; - OS=Homo sapiens (Human).
A8K008_HUMAN Mass: 51564 Score: 20 Matches: 1(0) Sequences: 1(0)
SubName: Full=cDNA FLJ78387; - OS=Homo sapiens (Human).

---

20. AHNK2_HUMAN Mass: 616242 Score: 20 Matches: 1(0) Sequences: 1(0) emPAI: 0.01
RecName: Full=Protein AHNAK2; - OS=Homo sapiens (Human).
☐ Check to include this hit in error tolerant search or archive report

	Query	Observed	Mr(expt)	Mr(calc)	ppm	Miss	Score	Expect	Rank	Unique	Retention Time	Peptide
☑	115	**476.2712**	**950.5279**	**950.5185**	**9.81**	**0**	**20**	**0.51**	**1**	**U**	**465s (7.75m)**	**R.TFSTQIVR.E**

Peptide matches not assigned to protein hits: (no details means no match)

	Query	Observed	Mr(expt)	Mr(calc)	ppm	Miss	Score	Expect	Rank	Unique	Retention Time	Peptide
☑	252	**593.3344**	**1184.6543**	**1184.6765**	**-18.72**	**0**	**26**	**0.23**	**1**		**579s (9.65m)**	**VLSTDILTAPR**
☑	86	**442.7696**	**883.5247**	**883.5351**	**-11.81**	**1**	**25**	**0.11**	**1**		**498s (8.30m)**	**LAAARLNR**
☑	154	**507.7840**	**1013.5535**	**1013.5618**	**-8.17**	**1**	**22**	**0.48**	**1**		**526s (8.77m)**	**RVSELSAPR**
☑	17	**563.3352**	**1124.6559**	**1124.6666**	**-9.51**	**1**	**20**	**0.49**	**1**		**861s (14.35m)**	**NLIQGKSIPR**
☑	203	**557.2810**	**1112.5474**	**1112.5574**	**-8.98**	**0**	**20**	**0.57**	**1**		**477s (7.95m)**	**NQGPQESVVR**
☑	54	**421.2931**	**840.5717**	**840.5797**	**-9.47**	**0**	**20**	**0.041**	**1**		**963s (16.05m)**	**IIITILR**
☑	315	**658.8800**	**1315.7454**	**1315.7361**	**7.14**	**1**	**19**	**0.7**	**1**		**1146s (19.10m)**	**VDLRFNNLGLR**

☑	213	563.2924	1124.5701	1124.5760	-5.25	2	19	0.89	1	681s (11.35m)	CRIFTRSDK
☑	87	445.2670	888.5193	888.5181	1.39	0	19	0.91	1	638s (10.63m)	FLNLNLR
☑	140	495.2922	988.5699	988.5528	17.3	0	19	0.71	1	654s (10.90m)	FVAICAPLR
☑	39	400.7660	799.5174	799.5167	0.93	0	18	0.85	1	643s (10.72m)	NLSLLIK
☑	454	897.9151	1793.8156	1793.8028	7.17	0	17	0.84	1	763s (12.72m)	TGTTGQSGAESGTTEPSAR
☑	3	446.2162	890.4179	890.4167	1.33	0	16	2.8	1	510s (8.50m)	LMEDLDR
☑	316	658.8800	1315.7454	1315.7361	7.14	1	16	1.3	1	1143s (19.05m)	VDLRFNNLGLR
☑	53	421.2931	840.5717	840.5797	-9.47	0	16	0.093	1	959s (15.98m)	IIITILR
☑	10	477.8256	953.6366	953.6246	12.6	2	16	0.25	1	1202s (20.03m)	QRRLLLR
☑	46	407.7771	813.5397	813.5436	-4.80	1	16	0.46	1	785s (13.08m)	LTILAKR
☑	48	412.7530	823.4915	823.4817	12.0	0	14	0.79	1	533s (8.88m)	WRPLPR
☑	2	445.2679	888.5213	888.5181	3.57	0	13	3.2	1	634s (10.57m)	FLNLNLR
☑	119	478.3010	954.5873	954.5974	-10.56	1	13	1.3	1	618s (10.30m)	LVKVINNR
☑	260	602.3337	1202.6528	1202.6407	10.0	1	13	4.6	1	799s (13.32m)	TINKAWVESR
☑	139	495.2922	988.5699	988.5528	17.3	0	13	2.9	1	650s (10.83m)	FVAICAPLR
☑	299	426.9128	1277.7165	1277.7132	2.52	1	13	2.5	1	548s (9.13m)	YGPVVKVVFDR
☑	180	529.7810	1057.5474	1057.5516	-3.99	1	12	5.7	1	496s (8.27m)	AINVAGRDDK
☑	246	588.3213	1174.6281	1174.6445	-13.99	1	12	5.1	1	766s (12.77m)	TLLDTEDKLK
☑	15	560.2986	559.2913	559.2966	-9.35	0	12	13	1	980s (16.33m)	GIENK
☑	298	426.9128	1277.7165	1277.6914	19.6	2	12	2.7	1	547s (9.12m)	MSFQGKKSIPR
☑	142	500.7595	999.5045	999.4913	13.3	0	12	3.4	1	540s (9.00m)	YLGEEYVK
☑	28	385.2030	768.3915	768.3878	4.83	1	12	3.5	1	527s (8.78m)	EKAHER
☑	231	574.8086	1147.6026	1147.5945	7.06	1	11	5.8	1	447s (7.45m)	ETRSSSTRPK
☑	147	502.2939	1002.5733	1002.5611	12.2	1	11	4.8	1	553s (9.22m)	DFLRSIPR
☑	120	478.3010	954.5873	954.5974	-10.56	1	11	1.9	1	614s (10.23m)	LVKVINNR
☑	51	414.7634	827.5122	827.5229	-12.94	0	11	5.4	1	662s (11.03m)	LIQSIVR
☑	384	512.6114	1534.8125	1534.8355	-14.97	1	11	7.8	1	460s (7.67m)	LREYLEGSNLITK
☑	217	564.3218	1126.6291	1126.6095	17.4	1	11	4.5	1	1259s (20.98m)	EGRALPATGQK
☑	123	478.3133	954.6121	954.5974	15.4	1	11	1.4	1	1013s (16.88m)	NLLKGAALR
☑	329	673.8543	1345.6941	1345.7210	-19.98	2	11	10	1	796s (13.27m)	APCRMKVSIPTK + Oxidation (M)
☑	73	431.7742	861.5337	861.5324	1.59	0	11	1.2	1	626s (10.43m)	IVGYVLAK
☑	8	472.2886	942.5627	942.5498	13.6	0	11	7.8	1	921s (15.35m)	TLATTLAPR
☑	441	881.9270	1761.8395	1761.8646	-14.25	0	11	5.1	1	728s (12.13m)	DAHSVITYQITSGNTR
☑	112	473.2811	944.5477	944.5556	-8.37	1	11	8	1	704s (11.73m)	VAFLSPRR
☑	29	385.2030	768.3915	768.3878	4.83	1	11	4.7	1	524s (8.73m)	EKAHER
☑	1	398.2214	1191.6424	1191.6467	-3.67	2	10	10	1	524s (8.73m)	DSLKKALMMR
☑	95	450.7659	899.5172	899.5188	-1.86	0	10	7.6	1	479s (7.98m)	LTINNVAR
☑	44	407.7574	813.5003	813.5072	-8.50	1	10	4.2	1	643s (10.72m)	KAQNLLK
☑	281	623.8441	1245.6735	1245.6717	1.48	0	10	8	1	698s (11.63m)	QFLSLRPEEK
☑	91	450.2710	898.5274	898.5348	-8.24	1	10	5.2	1	601s (10.02m)	TISGIPRR
☑	82	875.4576	874.4503	874.4396	12.3	0	10	25	1	1292s (21.53m)	TEEDILR
☑	282	623.8441	1245.6735	1245.6605	10.5	1	10	8.2	1	695s (11.58m)	FKVEEGVVPDK
☑	189	535.3237	1068.6328	1068.6404	-7.09	1	10	3	1	775s (12.92m)	ARTSVLPGLR
☑	122	478.3133	954.6121	954.6086	3.59	2	10	1.8	1	1011s (16.85m)	ERRLLLR
☑	501	742.3802	2224.1188	2224.1310	-5.48	2	10	8.7	1	731s (12.18m)	KLMLDTWNESIFSNIKNR + Oxidation (M)
☑	133	491.2741	980.5336	980.5515	-18.25	1	10	5.6	1	523s (8.72m)	NRGIPATPR
☑	113	475.7286	949.4426	949.4328	10.4	0	10	5.5	1	710s (11.83m)	WGTPGGMTK + Oxidation (M)
☑	187	535.3151	1068.6157	1068.6152	0.47	1	10	4.1	1	644s (10.73m)	LNGLAAQRAR
☑	383	763.3895	1524.7644	1524.7622	1.47	0	10	7.8	1	780s (13.00m)	VFFFCMPAPTIPR
☑	458	901.9710	1801.9273	1801.9547	-15.19	1	10	8.7	1	786s (13.10m)	INLTIHQRTHVEEGR
☑	212	563.2924	1124.5701	1124.5760	-5.25	2	10	7.6	1	677s (11.28m)	CRIFTRSDK
☑	218	564.3218	1126.6291	1126.6095	17.4	1	10	6.3	1	1261s (21.02m)	EGRALPATGQK
☑	421	822.9371	1643.8596	1643.8267	20.0	1	10	16	1	906s (15.10m)	ETSQILEFQEKHR
☑	397	783.8748	1565.7350	1565.7541	-12.20	0	10	8	1	583s (9.72m)	ATCIGNNSAAAVSMLK + Oxidation (M)
☑	106	467.7313	933.4480	933.4655	-18.71	1	9	8.2	1	821s (13.68m)	LTKSELDE
☑	134	491.2741	980.5336	980.5515	-18.25	1	9	6.1	1	518s (8.63m)	NRGIPATPR
☑	9	475.2945	474.2872	474.2802	14.9	0	9	5.5	1	475s (7.92m)	AASVK
☑	497	737.7083	2210.1032	2210.0723	14.0	2	9	10	1	721s (12.02m)	GAHVDARNGRGETALSAACGAAR
☑	75	432.7499	863.4852	863.4977	-14.46	1	9	7.5	1	532s (8.87m)	REPLPPR
☑	257	596.3182	1190.6219	1190.6004	18.1	2	9	11	1	675s (11.25m)	DVSRTDRTNK
☑	364	729.8792	1457.7438	1457.7337	6.91	1	9	14	1	867s (14.45m)	KTAMFDFNTVLR + Oxidation (M)
☑	455	897.9154	1793.8162	1793.8288	-6.99	1	9	5.4	1	760s (12.67m)	NGDEKSPLGTEIMMTR + Oxidation (M)
☑	206	557.8110	1113.6075	1113.6142	-6.01	0	9	13	1	897s (14.95m)	LNTILQEQR
☑	451	598.9443	1793.8109	1793.8195	-4.80	1	9	5.4	1	777s (12.95m)	VFMYDFAASNWSKGR + Oxidation (M)
☑	350	706.3729	1410.7313	1410.7229	6.01	2	9	9.7	1	797s (13.28m)	EAGAPGRAWGRQR
☑	185	534.8549	1067.6952	1067.6815	12.9	2	9	0.73	1	1239s (20.65m)	GVRELLRIL
☑	49	412.7530	823.4915	823.4817	12.0	0	8	3.2	1	528s (8.80m)	WRPLPR
☑	105	467.7294	933.4442	933.4590	-15.78	0	8	9.9	1	601s (10.02m)	QVMAEVNK + Oxidation (M)
☑	502	742.3802	2224.1188	2224.1310	-5.47	2	8	12	1	734s (12.23m)	KLMLDTWNESIFSNIKNR + Oxidation (M)
☑	317	659.3728	1316.7310	1316.7057	19.3	1	8	10	1	1138s (18.97m)	RGMLENCILLR
☑	459	901.9710	1801.9273	1801.9468	-10.82	1	8	12	1	784s (13.07m)	AAGQSLRAMVENVTLAR + Oxidation (M)
☑	31	386.6754	771.3362	771.3511	-19.31	0	8	2.2	1	617s (10.28m)	NEAADPR
☑	45	407.7574	813.5003	813.5072	-8.50	1	8	6.8	1	642s (10.70m)	KAQNLLK
☑	85	881.9280	1761.8415	1761.8646	-13.11	0	8	11	1	725s (12.08m)	DAHSVITYQITSGNTR
☑	235	577.2918	1152.5690	1152.5638	4.60	0	8	12	1	952s (15.87m)	LFLEGMHYK + Oxidation (M)
☑	396	783.8748	1565.7350	1565.7169	11.6	2	8	12	1	580s (9.67m)	EESETANKNDSSKK
☑	127	483.2735	964.5324	964.5263	6.37	0	8	9.5	1	502s (8.37m)	ATVLLSMSK + Oxidation (M)
☑	452	598.9443	1793.8109	1793.8195	-4.80	1	8	6.7	1	773s (12.88m)	VFMYDFAASNWSKGR + Oxidation (M)
☑	528	888.4655	2662.3745	2662.3669	2.85	2	8	11	1	1156s (19.27m)	NAMLKSICEVLDLERSGVNSELVK + Oxidation (M)
☑	406	798.3950	1594.7755	1594.8025	-16.92	0	8	70	1	1008s (16.80m)	SSIPVVCGYITVSDR
☑	264	606.3133	1210.6121	1210.6202	-6.70	0	7	10	1	606s (10.10m)	LIGCPVCIEHK
☑	179	529.2839	1056.5533	1056.5498	3.28	1	7	11	1	492s (8.20m)	LGEPQARMR
☑	254	595.8190	1189.6234	1189.6013	18.6	0	7	15	1	776s (12.93m)	CELATLQDIGK
☑	322	444.2117	1329.6132	1329.6387	-19.18	1	7	6.4	1	449s (7.48m)	NFIAMGKNYEK + Oxidation (M)
☑	132	488.2853	974.5561	974.5621	-6.16	2	7	12	1	650s (10.83m)	LSKASSRAR
☑	205	557.3372	1112.6598	1112.6553	3.97	0	7	11	1	602s (10.03m)	LSQTLSLVPR

☑	183	534.7954	1067.5763	1067.5876	-10.58	2	7	10	1	614s (10.23m)	RRSYLFLGG
☑	247	588.3213	1174.6281	1174.6445	-13.99	1	7	17	1	769s (12.82m)	TLLDTEDKLK
☑	306	649.8760	1297.7375	1297.7354	1.61	1	7	17	1	648s (10.80m)	INQTLDTKLPR
☑	155	508.2932	1014.5719	1014.5570	14.7	1	7	20	1	666s (11.10m)	AGTASRLSPR
☑	251	593.3344	1184.6543	1184.6765	-18.72	0	7	19	1	581s (9.68m)	VLSTDILTAPR
☑	103	463.2784	924.5422	924.5327	10.3	1	7	6.1	1	785s (13.08m)	MAAHLVKR
☑	197	549.7835	1097.5525	1097.5717	-17.42	1	7	14	1	578s (9.63m)	KELPPNGDTK
☑	286	626.3603	1250.7060	1250.6846	17.1	0	7	7.7	1	943s (15.72m)	FILLVSGFCPR
☑	188	535.3237	1068.6328	1068.6404	-7.09	1	7	6.2	1	772s (12.87m)	ARTSVLPGLR
☑	265	606.3133	1210.6121	1210.6306	-15.25	1	7	12	1	602s (10.03m)	DLRESPAQAPK
☑	256	596.3182	1190.6219	1190.6030	15.9	1	7	18	1	679s (11.32m)	LTKSELDEEK
☑	76	435.2851	868.5556	868.5606	-5.84	2	7	2.5	1	468s (7.80m)	RKIISPR
☑	436	857.9594	1713.9043	1713.9122	-4.59	2	7	18	1	649s (10.82m)	ARPSTKTPESSAAQRK
☑	262	603.3264	1204.6382	1204.6200	15.1	0	7	18	1	693s (11.55m)	ANVNAIDYLGR
☑	443	592.6460	1774.9163	1774.9499	-18.90	1	7	20	1	472s (7.87m)	LIGTEKSLPQGSMSSIK
☑	94	450.7609	899.5072	899.5076	-0.51	0	7	16	1	528s (8.80m)	IDVTLSPR
☑	301	641.8904	1281.7662	1281.7517	11.3	1	7	4.3	1	929s (15.48m)	VNLNVLREAVR
☑	453	598.9443	1793.8109	1793.8028	4.54	0	6	9	1	770s (12.83m)	TGTTGQSGAESGTTEPSAR
☑	21	629.3361	628.3289	628.3293	-0.64	0	6	43	1	839s (13.98m)	ASAAGPR
☑	114	475.7286	949.4426	949.4539	-11.84	0	6	12	1	714s (11.90m)	LTLADMDR + Oxidation (M)
☑	199	552.8078	1103.6011	1103.5836	15.9	0	6	21	1	845s (14.08m)	HGQNSLALHK
☑	385	513.5882	1537.7427	1537.7343	5.48	1	6	15	1	444s (7.40m)	KLMVFFSSCMSVK + 2 Oxidation (M)
☑	200	552.8078	1103.6011	1103.5897	10.3	0	6	22	1	841s (14.02m)	MDLVPSVTVK + Oxidation (M)
☑	156	508.2932	1014.5719	1014.5570	14.7	1	6	26	1	668s (11.13m)	AGTASRLSPR
☑	318	662.8556	1323.6966	1323.7081	-8.70	2	6	32	1	699s (11.65m)	YLSQMRNLRK + Oxidation (M)
☑	131	488.2853	974.5561	974.5621	-6.16	2	6	17	1	653s (10.88m)	LSKASSRAR
☑	410	804.9025	1607.7904	1607.8011	-6.65	1	6	26	1	936s (15.60m)	MIRNTDINLLMDK + 2 Oxidation (M)
☑	198	549.7835	1097.5525	1097.5717	-17.42	1	6	19	1	581s (9.68m)	KELPPNGDTK
☑	186	535.3151	1068.6157	1068.6179	-2.06	0	6	10	1	647s (10.78m)	NIVSVLAPEK
☑	244	585.8472	1169.6799	1169.6590	17.8	2	6	13	1	674s (11.23m)	AKMVPEKVPR + Oxidation (M)
☑	32	777.3251	776.3178	776.3123	7.07	0	6	7.3	1	754s (12.57m)	MPEGGDR + Oxidation (M)
☑	334	683.7938	1365.5729	1365.5516	15.7	0	6	8.8	1	976s (16.27m)	CGTPGWPCCLSDK
☑	184	534.8549	1067.6952	1067.6815	12.9	2	5	1.5	1	1242s (20.70m)	GVRELLRIL
☑	14	523.2877	1566.8414	1566.8340	4.69	1	5	49	1	759s (12.65m)	CFHQIIKALLASHS
☑	283	624.8527	1247.6909	1247.6808	8.09	2	5	19	1	819s (13.65m)	YGKLSRHLMK + Oxidation (M)
☑	233	575.3192	1148.6238	1148.6190	4.24	0	5	24	1	554s (9.23m)	LGTLSPAFSTR
☑	335	457.2573	1368.7500	1368.7765	-19.36	1	5	20	1	514s (8.57m)	DNAHLKILLLGY
☑	13	522.2912	1042.5679	1042.5481	19.0	0	5	43	1	834s (13.90m)	AMGEQLLPGK
☑	287	626.3603	1250.7060	1250.6846	17.1	0	5	12	1	938s (15.63m)	FILLVSGFCPR
☑	230	571.7706	1141.5266	1141.5404	-12.11	0	5	12	1	677s (11.28m)	VQDDFISYR
☑	214	563.2928	1124.5711	1124.5536	15.6	0	5	23	1	671s (11.18m)	EKPVCGTTYK
☑	302	641.8904	1281.7662	1281.7517	11.3	1	5	6.6	1	926s (15.43m)	VNLNVLREAVR
☑	135	491.7803	981.5460	981.5284	18.0	0	5	14	1	490s (8.17m)	SGPTWLPPK
☑	109	468.2354	934.4562	934.4430	14.1	0	5	26	1	505s (8.42m)	SPGMTVAEK + Oxidation (M)
☑	107	467.7313	933.4480	933.4655	-18.71	1	5	25	1	818s (13.63m)	LTKSELDE
☑	303	644.8673	1287.7200	1287.6969	18.0	1	5	21	1	720s (12.00m)	VMSIPKGNSALR + Oxidation (M)
☑	481	660.7030	1979.0871	1979.1125	-12.84	0	5	16	1	1033s (17.22m)	CNIAVILLTDLIIDHSVK
☑	307	649.8760	1297.7375	1297.7354	1.61	1	4	32	1	645s (10.75m)	INQTLDTKLPR
☑	6	467.7235	933.4325	933.4152	18.6	0	4	19	1	599s (9.98m)	AGSESAEQR
☑	121	478.3122	954.6098	954.6086	1.22	2	4	6.2	1	920s (15.33m)	RLAALQKR
☑	11	483.2744	964.5342	964.5454	-11.58	1	4	46	1	506s (8.43m)	GITIRSYR
☑	432	849.4469	1696.8791	1696.9009	-12.81	2	4	27	1	648s (10.80m)	KTFSEKSSLIHHQR
☑	431	849.4469	1696.8791	1696.9009	-12.81	2	4	27	1	645s (10.75m)	KTFSEKSSLIHHQR
☑	90	450.2710	898.5274	898.5236	4.25	1	4	20	1	604s (10.07m)	TLKLPDGR
☑	468	617.6555	1849.9447	1849.9244	11.0	0	4	48	1	462s (7.70m)	LSQTPTHMPTILDDPGK
☑	312	655.3243	1308.6340	1308.6530	-14.49	1	4	41	1	592s (9.87m)	LMKSAIGEGMTR + Oxidation (M)
☑	229	571.7706	1141.5266	1141.5476	-18.40	1	4	14	1	673s (11.22m)	GEAGRNGAPGEK
☑	367	491.5757	1471.7052	1471.7201	-10.12	0	4	20	1	459s (7.65m)	QQAANLLQDMGQR
☑	201	553.2888	1104.5631	1104.5676	-4.08	0	4	39	1	723s (12.05m)	ASAVIHDQHK
☑	319	662.8556	1323.6966	1323.6935	2.31	0	4	49	1	696s (11.60m)	IIDVDALWSHR
☑	116	477.8159	953.6173	953.6134	4.09	1	4	6.9	1	925s (15.42m)	RLLNLAVR
☑	344	698.9114	1395.8082	1395.7834	17.8	2	4	13	1	993s (16.55m)	TLQTPKQKEPAR
☑	354	710.8673	1419.7201	1419.7358	-11.03	1	4	36	1	956s (15.93m)	EGKVYGQAEAAIGK
☑	513	767.7224	2300.1453	2300.1088	15.9	2	4	32	1	905s (15.08m)	RLWKFFWEMDEAESWIK
☑	348	704.9148	1407.8150	1407.7908	17.2	0	4	12	1	1250s (20.83m)	MVLPPLPAALQSR + Oxidation (M)
☑	417	813.4525	1624.8904	1624.9123	-13.49	2	4	22	1	790s (13.17m)	VSPTMKVTHFLPRL
☑	239	578.8322	1155.6499	1155.6612	-9.74	1	4	31	1	451s (7.52m)	KVLAVTDSPAR
☑	4	451.2546	900.4947	900.4777	18.9	1	4	81	1	808s (13.47m)	GREVLDGR
☑	311	651.8751	1301.7356	1301.7316	3.09	2	4	24	1	788s (13.13m)	KRHLHAVEGQK
☑	124	479.7639	957.5133	957.4992	14.8	1	3	42	1	750s (12.50m)	GRELAEQR
☑	148	502.2939	1002.5733	1002.5611	12.2	1	3	30	1	557s (9.28m)	DFLRSIPR
☑	323	666.8752	1331.7359	1331.7119	18.0	0	3	30	1	1001s (16.68m)	TLVPEGMLTSLR + Oxidation (M)
☑	74	432.7499	863.4852	863.4688	19.1	0	3	28	1	535s (8.92m)	VIGGICFR
☑	243	390.5287	1168.5642	1168.5766	-10.68	2	3	40	1	449s (7.48m)	LMRDMKTMK + Oxidation (M)
☑	379	758.4089	1514.8033	1514.8304	-17.86	2	3	37	1	516s (8.60m)	ISLKLANEDKEQK
☑	210	558.8062	1115.5978	1115.6087	-9.79	1	3	47	1	801s (13.35m)	FKSHTVELR
☑	20	590.2832	589.2759	589.2820	-10.32	0	3	83	1	484s (8.07m)	QSAER
☑	485	665.3368	1992.9884	1992.9939	-2.72	0	3	38	1	848s (14.13m)	EPASAPQAPTLPTTAPAMAR + Oxidation (M)
☑	526	646.3487	2581.3658	2581.3673	-0.56	1	3	25	1	852s (14.20m)	MNLDSLSLALSQISYLVDNLTKK + Oxidation (M)
☑	333	683.7938	1365.5729	1365.5516	15.7	0	3	15	1	974s (16.23m)	CGTPGWPCCLSDK
☑	512	767.3936	2299.1589	2299.1348	10.5	0	3	1.7e+02	1	902s (15.03m)	WPIVQAFALEGIGGDGFFTMK + Oxidation (M)
☑	345	698.9114	1395.8082	1395.8085	-0.25	1	3	15	1	997s (16.62m)	KVEISIPASSLPR
☑	411	537.2818	1608.8235	1608.8260	-1.51	1	3	38	1	1172s (19.53m)	AFYVNVLNEEQRK
☑	435	857.9594	1713.9043	1713.9348	-17.82	1	3	41	1	652s (10.87m)	SPCPLRLLPPAGPGSPR
☑	310	651.8751	1301.7356	1301.7316	3.09	2	3	26	1	785s (13.08m)	KRHLHAVEGQK
☑	473	941.4289	1880.8432	1880.8580	-7.90	1	3	16	1	758s (12.63m)	LYQEKDAFQEEYYR

☑	255	596.3179	1190.6212	1190.6077	11.3	1	3	45	1	769s (12.82m)	ECILNRTQSK
☑	304	644.8673	1287.7200	1287.7088	8.71	1	3	30	1	724s (12.07m)	WYPTLPTRVR
☑	522	426.0190	2550.0700	2550.1009	-12.10	0	3	3.3	1	534s (8.90m)	MAFELATGDYLFEPHSGEDYSR + Oxidation (M)
☑	175	526.3030	1050.5915	1050.5709	19.6	1	3	23	1	756s (12.60m)	KEPIPENPK
☑	278	619.3187	1236.6228	1236.6319	-7.31	1	3	40	1	493s (8.22m)	GLTKVNICMSR + Oxidation (M)
☑	273	615.8836	1229.7527	1229.7496	2.52	0	3	4.6	1	582s (9.70m)	GRPLLLPPPSAL
☑	438	871.4402	1740.8658	1740.8361	17.0	0	3	35	1	961s (16.02m)	MSPTPNSCPLPLPLCR + Oxidation (M)
☑	245	585.8472	1169.6799	1169.6880	-6.99	2	2	27	1	678s (11.30m)	GITVKQKSPGR
☑	16	560.3011	1118.5876	1118.5754	10.9	0	2	1.2e+02	1	822s (13.70m)	MLENLLSQR + Oxidation (M)
☑	41	404.2404	806.4662	806.4650	1.46	1	2	22	1	508s (8.47m)	ILYKDR
☑	226	567.8315	1133.6485	1133.6332	13.5	0	2	26	1	779s (12.98m)	LITDSYPVVK
☑	234	575.3192	1148.6238	1148.6190	4.24	0	2	48	1	551s (9.18m)	LGTLSPAFSTR
☑	151	505.2501	1008.4856	1008.4698	15.7	0	2	34	1	472s (7.87m)	LCQAFENGK
☑	416	811.9106	1621.8066	1621.8100	-2.10	1	2	49	1	951s (15.85m)	EGSSSWLQWKSLSK
☑	204	557.3372	1112.6598	1112.6740	-12.76	1	2	34	1	605s (10.08m)	AVKMPVALLR + Oxidation (M)
☑	529	888.4655	2662.3745	2662.3669	2.85	2	2	40	1	1159s (19.32m)	NAMLKSICEVLDLERSGVNSELVK + Oxidation (M)
☑	40	404.2404	806.4662	806.4650	1.46	1	2	24	1	504s (8.40m)	ILYKDR
☑	163	514.7843	1027.5540	1027.5523	1.68	1	2	40	1	483s (8.05m)	QAGATPTRAR
☑	423	827.3963	1652.7781	1652.7724	3.42	0	2	38	1	803s (13.38m)	CPELLANYCDMLLR
☑	477	970.9748	1939.9350	1939.9639	-14.93	1	2	43	1	767s (12.78m)	GNEYEALTSPQTSFRLK
☑	268	610.3352	1218.6558	1218.6577	-1.54	1	2	54	1	695s (11.58m)	RCLTTPMLLR + Oxidation (M)
☑	407	798.8864	1595.7583	1595.7838	-15.98	0	2	34	1	802s (13.37m)	LMHSNSLNNSNIPR
☑	467	616.5817	1846.7232	1846.7527	-15.94	0	2	0.91	1	771s (12.85m)	ETGPEDPSSMPEESSPR + Oxidation (M)
☑	305	648.8288	1295.6431	1295.6582	-11.67	0	2	69	1	457s (7.62m)	RPGAGPLEDAASR
☑	505	749.7383	2246.1932	2246.1762	7.54	2	2	39	1	926s (15.43m)	SKVGELNERLGMLMETIQAK
☑	478	970.9748	1939.9350	1939.9639	-14.93	1	2	46	1	764s (12.73m)	GNEYEALTSPQTSFRLK
☑	102	461.2628	920.5110	920.5001	11.9	2	2	42	1	488s (8.13m)	KMEELKK + Oxidation (M)
☑	448	892.9150	1783.8155	1783.8233	-4.35	0	2	31	1	1295s (21.58m)	DVVRPYQTMSNPMSK + 2 Oxidation (M)
☑	324	666.8752	1331.7359	1331.7119	18.0	0	2	45	1	996s (16.60m)	TLVPEGMLTSLR + Oxidation (M)
☑	373	748.8759	1495.7373	1495.7453	-5.37	1	2	51	1	813s (13.55m)	VTQTELMRESFR
☑	351	706.3729	1410.7313	1410.7329	-1.14	2	1	50	1	792s (13.20m)	KKGIFASPCGLYQ
☑	242	583.3220	1164.6294	1164.6476	-15.56	2	1	50	1	817s (13.62m)	KASSPNLHRR
☑	152	507.2784	1012.5423	1012.5526	-10.18	1	1	34	1	567s (9.45m)	RNASKPQGR
☑	464	913.9839	1825.9533	1825.9223	17.0	1	1	52	1	741s (12.35m)	AYSQSPRHALYPPSPR
☑	381	508.2590	1521.7553	1521.7497	3.65	1	1	69	1	444s (7.40m)	QVPVYDREIMEK + Oxidation (M)
☑	237	577.8155	1153.6165	1153.6019	12.6	0	1	86	1	682s (11.37m)	YLDEFLLNK
☑	22	667.3175	1998.9306	1998.9662	-17.81	0	1	1.2e+02	1	506s (8.43m)	MPFFLTPFEPAHIDGHK + Oxidation (M)
☑	97	454.2344	906.4542	906.4481	6.76	0	1	59	1	604s (10.07m)	VSMEAVQK + Oxidation (M)
☑	216	564.2775	1126.5405	1126.5407	-0.23	0	1	43	1	518s (8.63m)	LHVDPEDFR
☑	98	458.2338	914.4530	914.4458	7.95	0	1	32	1	604s (10.07m)	TVDPEQAR
☑	424	827.3963	1652.7781	1652.7724	3.42	0	1	48	1	800s (13.33m)	CPELLANYCDMLLR
☑	289	629.8639	1257.7133	1257.7081	4.14	1	1	81	1	711s (11.85m)	YLIPKGDLSPR
☑	267	608.2615	1214.5084	1214.4907	14.6	0	1	11	1	469s (7.82m)	MEMESAAASTR + 2 Oxidation (M)
☑	372	747.8178	1493.6211	1493.5915	19.8	0	1	8	1	705s (11.75m)	MYDFTQCENTSR
☑	492	1042.0119	2082.0093	2081.9761	15.9	0	1	64	1	807s (13.45m)	ENCIMQEDESYLLQVLR
☑	462	606.3052	1815.8938	1815.9189	-13.81	2	1	61	1	812s (13.53m)	KLDLLCKTSHLSDTGW
☑	395	781.3915	1560.7684	1560.7580	6.72	1	1	87	1	793s (13.22m)	GVTGAGGWGSAAVCRGR
☑	65	425.7605	849.5064	849.5185	-14.16	0	1	23	1	437s (7.28m)	LVLGGHVR
☑	52	414.7634	827.5122	827.5089	3.90	2	1	61	1	658s (10.97m)	ARAKQVR
☑	108	467.7337	933.4529	933.4590	-6.46	1	1	69	1	501s (8.35m)	MDPTAGSKK
☑	160	511.7852	1021.5558	1021.5556	0.15	0	1	55	1	572s (9.53m)	SVTIYLGNR
☑	361	479.9077	1436.7012	1436.6864	10.3	1	1	60	1	443s (7.38m)	GCRLPSISSTTCGR
☑	569	901.8648	4504.2878	4504.3294	-9.24	2	1	28	1	1198s (19.97m)	VFRSSPQFGVTLLTYELLQRWFYIDFGGVKPMGSEPVPK
☑	450	896.9067	1791.7989	1791.8224	-13.11	0	1	28	1	893s (14.88m)	GHPIPHGGMQGGFGGQSR + Oxidation (M)
☑	343	697.8529	1393.6913	1393.7024	-7.91	2	1	65	1	737s (12.28m)	QGFMEKTGPKQK + Oxidation (M)
☑	211	560.2992	1118.5839	1118.5680	14.2	1	0	81	1	984s (16.40m)	RSSQSAPTTGK
☑	5	461.2620	1380.7640	1380.7435	14.9	1	0	1e+02	1	529s (8.82m)	CPAEAVPPAGLTKK
☑	295	635.8051	1269.5956	1269.5949	0.55	0	0	40	1	463s (7.72m)	VQSHQQASEEK
☑	66	426.2208	850.4270	850.4405	-15.84	0	0	64	1	571s (9.52m)	IICMTVR + Oxidation (M)
☑	336	690.8444	1379.6742	1379.6537	14.9	0	0	73	1	672s (11.20m)	SMENLQSCISLR
☑	425	827.8904	1653.7662	1653.7953	-17.61	2	0	43	1	783s (13.05m)	ELEAEGKKLTMDMK + 2 Oxidation (M)
☑	290	630.2740	1258.5334	1258.5104	18.3	1	0	12	1	473s (7.88m)	EKASSSCSCVCR
☑	327	667.8364	1333.6582	1333.6812	-17.25	1	0	78	1	619s (10.32m)	WLLGMKNNQSK + Oxidation (M)
☑	80	437.2201	872.4256	872.4352	-10.93	0	0	48	1	600s (10.00m)	VQDLENR
☑	326	667.3689	1332.7233	1332.7038	14.7	0	0	72	1	990s (16.50m)	TISGSYQLTVHK
☑	449	896.9063	1791.7981	1791.8224	-13.52	0	0	32	1	883s (14.72m)	GHPIPHGGMQGGFGGQSR + Oxidation (M)
☑	325	667.3405	1332.6664	1332.6456	15.6	1	0	80	1	615s (10.25m)	SSLAVRDPAMDR + Oxidation (M)
☑	284	624.8527	1247.6909	1247.6834	6.07	2	0	63	1	823s (13.72m)	VKDINSSTKTR
☑	7	*472.2868*	*471.2795*							*924s (15.40m)*	
☑	18	*564.3545*	*563.3472*							*651s (10.85m)*	
☑	23	*685.7914*	*684.7841*							*889s (14.82m)*	
☑	24	*746.0621*	*745.0549*							*500s (8.33m)*	
☑	27	*384.2348*	*766.4550*							*798s (13.30m)*	
☑	30	*385.2608*	*768.5071*							*478s (7.97m)*	
☑	33	*779.3261*	*778.3188*							*823s (13.72m)*	
☑	34	*391.2035*	*780.3924*							*571s (9.52m)*	
☑	35	*391.2035*	*780.3924*							*568s (9.47m)*	
☑	42	*406.2485*	*810.4825*							*829s (13.82m)*	
☑	43	*406.2485*	*810.4825*							*830s (13.83m)*	
☑	47	*409.7432*	*817.4719*							*486s (8.10m)*	
☑	50	*414.7610*	*827.5075*							*831s (13.85m)*	
☑	64	*425.7605*	*849.5064*							*439s (7.32m)*	
☑	67	*428.2611*	*854.5076*							*860s (14.33m)*	
☑	68	*428.2611*	*854.5076*							*863s (14.38m)*	
☑	71	*431.2628*	*860.5111*							*442s (7.37m)*	
☑	72	*431.2628*	*860.5111*							*440s (7.33m)*	

☑	77	436.7749	871.5351	863s (14.38m)
☑	78	436.7750	871.5355	860s (14.33m)
☑	79	437.2201	872.4256	603s (10.05m)
☑	83	440.2506	878.4867	535s (8.92m)
☑	84	881.8562	880.8489	570s (9.50m)
☑	88	445.7895	889.5644	733s (12.22m)
☑	89	445.7895	889.5644	740s (12.33m)
☑	92	450.2757	898.5368	894s (14.90m)
☑	93	450.2757	898.5368	891s (14.85m)
☑	96	453.3434	904.6722	1112s (18.53m)
☑	99	458.2338	914.4530	608s (10.13m)
☑	100	458.7888	915.5630	894s (14.90m)
☑	101	458.7888	915.5630	892s (14.87m)
☑	104	463.6744	925.3343	604s (10.07m)
☑	117	478.2540	954.4935	525s (8.75m)
☑	118	478.2540	954.4935	521s (8.68m)
☑	130	487.8092	973.6039	1059s (17.65m)
☑	136	984.9437	983.9364	748s (12.47m)
☑	137	494.3015	986.5884	955s (15.92m)
☑	138	494.3015	986.5884	960s (16.00m)
☑	141	497.2905	992.5665	618s (10.30m)
☑	145	502.1835	1002.3525	715s (11.92m)
☑	146	502.1835	1002.3525	717s (11.95m)
☑	149	502.8161	1003.6177	956s (15.93m)
☑	150	504.7777	1007.5409	515s (8.58m)
☑	153	507.7769	1013.5392	569s (9.48m)
☑	157	509.8225	1017.6305	1092s (18.20m)
☑	158	1021.7732	1020.7659	1316s (21.93m)
☑	159	511.7852	1021.5558	574s (9.57m)
☑	161	511.7884	1021.5623	482s (8.03m)
☑	162	511.7884	1021.5623	479s (7.98m)
☑	166	516.3155	1030.6165	987s (16.45m)
☑	169	522.7794	1043.5443	605s (10.08m)
☑	170	523.3228	1044.6310	1122s (18.70m)
☑	171	524.7071	1047.3996	515s (8.58m)
☑	172	524.7071	1047.3996	511s (8.52m)
☑	173	524.8270	1047.6394	988s (16.47m)
☑	174	525.3646	1048.7147	480s (8.00m)
☑	176	526.3047	1050.5949	759s (12.65m)
☑	177	527.2727	1052.5308	654s (10.90m)
☑	178	527.2727	1052.5308	650s (10.83m)
☑	181	531.7955	1061.5764	1032s (17.20m)
☑	182	531.7955	1061.5764	1028s (17.13m)
☑	190	535.7838	1069.5530	492s (8.20m)
☑	191	538.3293	1074.6440	1017s (16.95m)
☑	192	538.6977	1075.3809	502s (8.37m)
☑	193	538.6977	1075.3809	499s (8.32m)
☑	194	545.3363	1088.6580	1152s (19.20m)
☑	195	549.7406	1097.4667	480s (8.00m)
☑	196	549.7406	1097.4667	476s (7.93m)
☑	207	1116.5574	1115.5501	1100s (18.33m)
☑	208	1116.5665	1115.5592	1107s (18.45m)
☑	209	1116.5670	1115.5597	1103s (18.38m)
☑	215	564.2775	1126.5405	516s (8.60m)
☑	221	565.8102	1129.6057	477s (7.95m)
☑	223	567.3307	1132.6469	874s (14.57m)
☑	224	567.3308	1132.6471	872s (14.53m)
☑	225	567.8315	1133.6484	781s (13.02m)
☑	232	574.8093	1147.6041	450s (7.50m)
☑	236	577.8155	1153.6165	685s (11.42m)
☑	238	578.8322	1155.6499	453s (7.55m)
☑	248	589.3436	1176.6727	895s (14.92m)
☑	249	589.3436	1176.6727	898s (14.97m)
☑	253	595.8190	1189.6234	772s (12.87m)
☑	258	600.7733	1199.5320	541s (9.02m)
☑	259	600.7733	1199.5320	538s (8.97m)
☑	261	603.3264	1204.6382	694s (11.57m)
☑	266	608.2615	1214.5084	472s (7.87m)
☑	271	611.3564	1220.6982	925s (15.42m)
☑	272	611.3564	1220.6982	922s (15.37m)
☑	274	617.2696	1232.5247	953s (15.88m)
☑	275	617.2696	1232.5247	949s (15.82m)
☑	276	412.5433	1234.6082	458s (7.63m)
☑	277	618.3422	1234.6699	675s (11.25m)
☑	279	623.2653	1244.5161	473s (7.88m)
☑	280	623.2653	1244.5161	469s (7.82m)
☑	285	625.8418	1249.6691	629s (10.48m)
☑	288	629.3346	1256.6547	842s (14.03m)
☑	291	630.8525	1259.6904	1021s (17.02m)
☑	292	630.8525	1259.6904	1018s (16.97m)
☑	293	633.3697	1264.7249	950s (15.83m)
☑	294	635.8051	1269.5956	466s (7.77m)
☑	296	639.3063	1276.5981	782s (13.03m)
☑	297	639.3063	1276.5981	785s (13.08m)
☑	300	427.2466	1278.7181	534s (8.90m)
☑	308	650.7870	1299.5595	469s (7.82m)

☑	309	651.2859	1300.5573	466s (7.77m)
☑	313	655.3243	1308.6340	595s (9.92m)
☑	314	658.7854	1315.5562	474s (7.90m)
☑	321	665.7631	1329.5116	786s (13.10m)
☑	328	672.2905	1342.5664	464s (7.73m)
☑	330	677.8743	1353.7341	899s (14.98m)
☑	331	677.8743	1353.7341	896s (14.93m)
☑	332	682.3810	1362.7474	734s (12.23m)
☑	337	690.8444	1379.6742	669s (11.15m)
☑	338	690.8868	1379.7590	545s (9.08m)
☑	339	690.8868	1379.7590	541s (9.02m)
☑	341	695.8476	1389.6807	786s (13.10m)
☑	342	695.8476	1389.6807	789s (13.15m)
☑	346	469.5537	1405.6392	459s (7.65m)
☑	353	710.8673	1419.7201	953s (15.88m)
☑	355	711.3589	1420.7032	934s (15.57m)
☑	356	711.3589	1420.7032	858s (14.30m)
☑	359	718.3041	1434.5936	503s (8.38m)
☑	360	718.3041	1434.5936	499s (8.32m)
☑	362	722.8132	1443.6119	470s (7.83m)
☑	363	722.8132	1443.6119	467s (7.78m)
☑	365	732.3190	1462.6235	513s (8.55m)
☑	366	489.9112	1466.7119	443s (7.38m)
☑	369	739.4056	1476.7967	722s (12.03m)
☑	371	747.8178	1493.6211	709s (11.82m)
☑	374	748.8759	1495.7373	809s (13.48m)
☑	375	754.8288	1507.6430	782s (13.03m)
☑	376	754.8288	1507.6430	779s (12.98m)
☑	377	758.3791	1514.7437	718s (11.97m)
☑	378	758.4089	1514.8033	519s (8.65m)
☑	382	763.3895	1524.7644	783s (13.05m)
☑	386	771.3244	1540.6342	802s (13.37m)
☑	387	771.3244	1540.6342	805s (13.42m)
☑	388	777.3229	1552.6313	655s (10.92m)
☑	389	777.3229	1552.6313	651s (10.85m)
☑	390	779.3286	1556.6427	820s (13.67m)
☑	391	519.9184	1556.7333	460s (7.67m)
☑	392	519.9184	1556.7333	456s (7.60m)
☑	393	779.8501	1557.6857	557s (9.28m)
☑	394	780.3501	1558.6856	555s (9.25m)
☑	398	784.8962	1567.7777	835s (13.92m)
☑	399	784.8962	1567.7777	832s (13.87m)
☑	400	791.3099	1580.6052	868s (14.47m)
☑	401	791.3101	1580.6056	864s (14.40m)
☑	402	792.3278	1582.6411	806s (13.43m)
☑	403	795.7808	1589.5470	865s (14.42m)
☑	404	795.7808	1589.5470	862s (14.37m)
☑	405	530.8590	1589.5553	866s (14.43m)
☑	409	804.9025	1607.7904	933s (15.55m)
☑	412	805.4194	1608.8243	1169s (19.48m)
☑	413	805.4194	1608.8243	1166s (19.43m)
☑	414	540.5751	1618.7036	450s (7.50m)
☑	415	811.9106	1621.8066	954s (15.90m)
☑	418	815.3706	1628.7267	562s (9.37m)
☑	419	815.3706	1628.7267	558s (9.30m)
☑	420	819.9147	1637.8148	593s (9.88m)
☑	422	550.5999	1648.7779	446s (7.43m)
☑	426	553.2709	1656.7909	461s (7.68m)
☑	427	556.2772	1665.8098	441s (7.35m)
☑	428	833.9236	1665.8327	762s (12.70m)
☑	429	833.9236	1665.8327	760s (12.67m)
☑	430	837.9252	1673.8357	1094s (18.23m)
☑	433	428.6386	1710.5253	532s (8.87m)
☑	434	857.4094	1712.8042	740s (12.33m)
☑	437	871.4402	1740.8658	957s (15.95m)
☑	439	881.4269	1760.8392	1245s (20.75m)
☑	440	881.8569	1761.6992	573s (9.55m)
☑	442	882.4143	1762.8140	971s (16.18m)
☑	444	890.9050	1779.7955	766s (12.77m)
☑	445	890.9050	1779.7955	763s (12.72m)
☑	446	892.8881	1783.7617	923s (15.38m)
☑	447	892.9150	1783.8155	1292s (21.53m)
☑	456	900.9309	1799.8472	981s (16.35m)
☑	457	900.9309	1799.8472	978s (16.30m)
☑	460	603.6709	1807.9910	1165s (19.42m)
☑	461	606.3052	1815.8938	808s (13.47m)
☑	463	912.4254	1822.8363	1015s (16.92m)
☑	465	913.9839	1825.9533	738s (12.30m)
☑	466	616.5817	1846.7232	774s (12.90m)
☑	469	927.9576	1853.9007	1099s (18.32m)
☑	470	928.4613	1854.9080	1097s (18.28m)
☑	472	941.4289	1880.8432	761s (12.68m)
☑	474	945.4338	1888.8530	493s (8.22m)
☑	475	967.4458	1932.8770	552s (9.20m)
☑	476	967.4458	1932.8770	549s (9.15m)
☑	479	984.9469	1967.8791	751s (12.52m)

480	660.7030	1979.0871	1029s (17.15m)
482	994.0438	1986.0731	1113s (18.55m)
483	994.5382	1987.0618	1110s (18.50m)
484	994.9780	1987.9414	1004s (16.73m)
486	667.6854	2000.0343	948s (15.80m)
487	1013.9488	2025.8831	745s (12.42m)
488	685.9790	2054.9151	448s (7.47m)
489	691.3109	2070.9109	435s (7.25m)
490	691.3109	2070.9109	438s (7.30m)
491	1042.0119	2082.0093	810s (13.50m)
493	1056.9714	2111.9282	735s (12.25m)
494	1070.5313	2139.0480	1299s (21.65m)
495	1070.7820	2139.5494	1296s (21.60m)
496	539.7758	2155.0741	483s (8.05m)
498	738.0384	2211.0934	717s (11.95m)
499	741.0478	2220.1216	939s (15.65m)
500	741.0478	2220.1216	935s (15.58m)
503	742.7095	2225.1067	712s (11.87m)
504	559.7930	2235.1431	497s (8.28m)
506	1130.3368	2258.6590	1303s (21.72m)
507	754.0590	2259.1553	1313s (21.88m)
508	1130.8306	2259.6466	1306s (21.77m)
509	1134.8319	2267.6492	1290s (21.50m)
510	762.3888	2284.1447	880s (14.67m)
511	762.3888	2284.1447	882s (14.70m)
514	801.0995	2400.2766	1027s (17.12m)
515	801.0995	2400.2766	1030s (17.17m)
516	807.4197	2419.2373	1271s (21.18m)
517	807.4211	2419.2413	1263s (21.05m)
518	826.7740	2477.3002	1066s (17.77m)
519	826.7740	2477.3002	1062s (17.70m)
520	847.0991	2538.2755	1048s (17.47m)
521	847.0991	2538.2755	1045s (17.42m)
523	426.5801	2553.4367	530s (8.83m)
524	427.4857	2558.8706	531s (8.85m)
525	427.4909	2558.9020	531s (8.85m)
527	646.3487	2581.3659	855s (14.25m)
530	683.9475	2731.7609	539s (8.98m)
531	927.3746	2779.1020	453s (7.55m)
532	927.4798	2779.4177	1310s (21.83m)
533	927.8152	2780.4237	1327s (22.12m)
534	933.4188	2797.2345	876s (14.60m)
535	729.3840	2913.5069	991s (16.52m)
536	729.3840	2913.5069	994s (16.57m)
537	972.1777	2913.5114	998s (16.63m)
538	729.6299	2914.4905	864s (14.40m)
539	981.3938	2941.1595	454s (7.57m)
540	774.6605	3094.6131	1234s (20.57m)
541	1032.5459	3094.6160	1229s (20.48m)
542	1032.5460	3094.6163	1218s (20.30m)
543	1047.1531	3138.4374	787s (13.12m)
544	794.6632	3174.6236	1102s (18.37m)
545	794.6632	3174.6236	1098s (18.30m)
546	837.6752	3346.6717	1096s (18.27m)
547	837.6753	3346.6723	1087s (18.12m)
548	837.6753	3346.6723	1090s (18.17m)
549	837.9229	3347.6625	967s (16.12m)
550	837.9229	3347.6625	964s (16.07m)
551	843.6456	3370.5533	470s (7.83m)
552	843.6456	3370.5533	467s (7.78m)
553	849.2130	3392.8228	1136s (18.93m)
554	849.2130	3392.8228	1139s (18.98m)
555	1017.7779	4067.0827	1361s (22.68m)
556	1017.7779	4067.0827	1364s (22.73m)
557	1021.5243	4082.0679	1350s (22.50m)
558	1021.7792	4083.0877	1355s (22.58m)
559	1066.7844	4263.1083	1345s (22.42m)
560	1070.5341	4278.1073	1284s (21.40m)
561	1126.8288	4503.2860	1302s (21.70m)
562	1126.8288	4503.2860	1293s (21.55m)
563	1126.8288	4503.2860	1300s (21.67m)
564	1126.8288	4503.2860	1297s (21.62m)
565	901.6648	4503.2875	1287s (21.45m)
566	901.6648	4503.2875	1289s (21.48m)
567	1127.0760	4504.2748	1305s (21.75m)
568	901.8648	4504.2876	1193s (19.88m)
570	753.8914	4517.3045	1308s (21.80m)
571	1134.3330	4533.3030	1277s (21.28m)
572	907.6686	4533.3068	1256s (20.93m)
573	907.8702	4534.3146	1253s (20.88m)
574	1063.9527	5314.7270	1467s (24.45m)
575	1063.9527	5314.7270	1470s (24.50m)
576	927.4840	5558.8601	1412s (23.53m)
577	927.4840	5558.8601	1419s (23.65m)
578	927.4840	5558.8601	1414s (23.57m)
579	927.8181	5560.8649	1322s (22.03m)

☑	580	*1113.1804*	*5560.8654*	*1409s (23.48m)*
☑	581	*1113.1804*	*5560.8654*	*1416s (23.60m)*

Search Parameters

```
Type of search         : MS/MS Ion Search
Enzyme                 : Trypsin
Variable modifications : Oxidation (M)
Mass values            : Monoisotopic
Protein Mass           : Unrestricted
Peptide Mass Tolerance : ± 20 ppm
Fragment Mass Tolerance: ± 0.2 Da
Max Missed Cleavages   : 2
Instrument type        : ESI-QUAD-TOF
Number of queries      : 581
```

Mascot: http://www.matrixscience.com/

Mascot Search Results

Host	: 10.10.10.100
User	: alison@anatomy
Email	:
Search title	: CPFP SCH6428 [13/18] 140331_alison_set3_4.mgf
MS data file	: 140331_alison_set3_4.mgf.mascot
Database	: SWISSPROT sprot (538010 sequences; 190998508 residues)
Timestamp	: 2 Apr 2014 at 00:35:41 GMT
Protein hits	: HS90A_BOVIN RecName: Full=Heat shock protein HSP 90-alpha; - OS=Bos taurus (Bovine).
	HS90B_BOVIN RecName: Full=Heat shock protein HSP 90-beta; - OS=Bos taurus (Bovine).
	ACTN4_BOVIN RecName: Full=Alpha-actinin-4; AltName: Full=F-actin cross-linking protein; AltName: Full=Non-muscle alpha-actin
	CO3_HUMAN RecName: Full=Complement C3; AltName: Full=C3 and PZP-like alpha-2-macroglobulin domain-containing protein 1; Co
	ACTN4_CHICK RecName: Full=Alpha-actinin-4; AltName: Full=F-actin cross-linking protein; AltName: Full=Non-muscle alpha-actin
	LG3BP_HUMAN RecName: Full=Galectin-3-binding protein; AltName: Full=Basement membrane autoantigen p105; AltName: Full=Lectin
	HSP83_DROPS RecName: Full=Heat shock protein 83; AltName: Full=HSP 82; - OS=Drosophila pseudoobscura pseudoobscura (Fruit fl
	PDIA1_HUMAN RecName: Full=Protein disulfide-isomerase; Short=PDI; EC=5.3.4.1; AltName: Full=Cellular thyroid hormone-binding
	PGBM_HUMAN RecName: Full=Basement membrane-specific heparan sulfate proteoglycan core protein; Short=HSPG; AltName: Full=Pe
	HSP90_BRUPA RecName: Full=Heat shock protein 90; - OS=Brugia pahangi (Filarial nematode worm).
	HS90B_DANRE RecName: Full=Heat shock protein HSP 90-beta; - OS=Danio rerio (Zebrafish) (Brachydanio rerio).
	1433B_BOVIN RecName: Full=14-3-3 protein beta/alpha; AltName: Full=Protein kinase C inhibitor protein 1; Short=KCIP-1; Conta
	BGH3_HUMAN RecName: Full=Transforming growth factor-beta-induced protein ig-h3; Short=Beta ig-h3; AltName: Full=Kerato-epit
	AGRIN_HUMAN RecName: Full=Agrin; Flags: Precursor; - OS=Homo sapiens (Human).
	K2C1_HUMAN RecName: Full=Keratin, type II cytoskeletal 1; AltName: Full=67 kDa cytokeratin; AltName: Full=Cytokeratin-1; Sh
	ACT_MANSE RecName: Full=Actin, muscle; Flags: Precursor; - OS=Manduca sexta (Tobacco hawkmoth) (Tobacco hornworm).
	H2B1B_MOUSE RecName: Full=Histone H2B type 1-B; AltName: Full=h2B-143; - OS=Mus musculus (Mouse).
	K2C8_HUMAN RecName: Full=Keratin, type II cytoskeletal 8; AltName: Full=Cytokeratin-8; Short=CK-8; AltName: Full=Keratin-8;
	TRYP_PIG RecName: Full=Trypsin; EC=3.4.21.4; Flags: Precursor; - OS=Sus scrofa (Pig).
	CALMA_ARBPU RecName: Full=Calmodulin-alpha; Short=CaM A; Flags: Fragment; - OS=Arbacia punctulata (Punctuate sea urchin).

	SWISSPROT	Decoy	False discovery rate
Peptide matches above identity threshold	123	1	0.81 %
Peptide matches above homology or identity threshold	137	4	2.92 %

Mascot Score Histogram

Ions score is -10*Log(P), where P is the probability that the observed match is a random event.
Individual ions scores > 37 indicate identity or extensive homology (p<0.05).
Protein scores are derived from ions scores as a non-probabilistic basis for ranking protein hits.

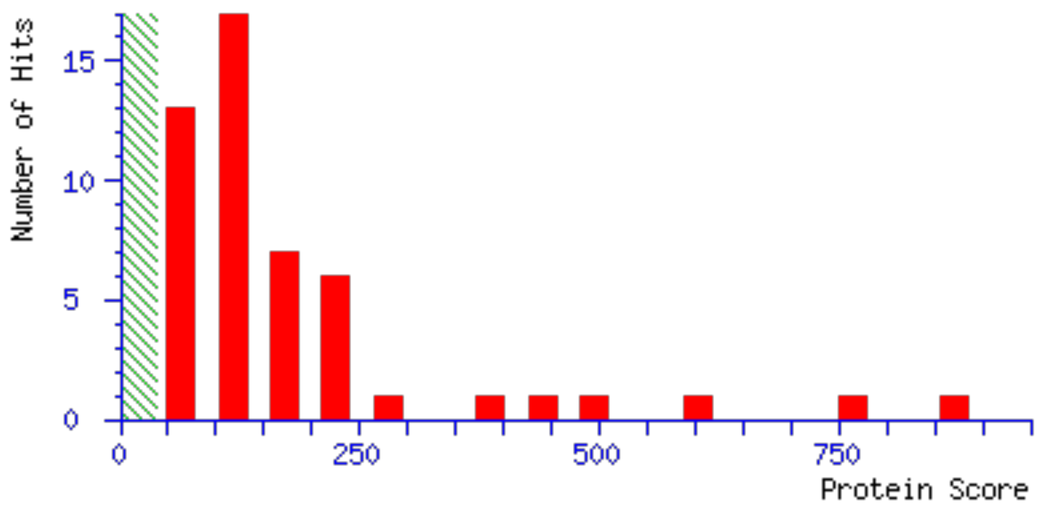

Peptide Summary Report

Format As	Peptide Summary		Help
	Significance threshold p< 0.05	Max. number of hits 20	Show Percolator scores
	Standard scoring MudPIT scoring	Ions score or expect cut-off 0	Show sub-sets 0
	Show pop-ups Suppress pop-ups	Sort unassigned Decreasing Score	Require bold red
	Preferred taxonomy All entries		

1. HS90A_BOVIN **Mass:** 84678 **Score:** 871 **Matches:** 21(18) **Sequences:** 16(13) **emPAI:** 0.64
RecName: Full=Heat shock protein HSP 90-alpha; - OS=Bos taurus (Bovine).
Check to include this hit in error tolerant search or archive report

Query	Observed	Mr(expt)	Mr(calc)	ppm	Miss	Score	Expect	Rank	Unique	Retention Time	Peptide
41	408.2592	814.5038	814.5065	-3.33	0	42	0.026	1	U	811s (13.52m)	R.ALLFVPR.R
42	408.2592	814.5038	814.5065	-3.33	0	(40)	0.035	1	U	815s (13.58m)	R.ALLFVPR.R
78	451.2634	900.5122	900.5181	-6.62	0	12	36	3		543s (9.05m)	K.TKPIWTR.N
94	474.7237	947.4328	947.4389	-6.44	0	33	0.077	1		562s (9.37m)	K.FYEQFSK.N
123	520.2492	1038.4838	1038.4869	-3.01	0	42	0.01	1		516s (8.60m)	R.YESLTDPSK.L
155	576.2807	1150.5469	1150.5506	-3.18	0	49	0.0034	1		518s (8.63m)	K.YIDQEELNK.T
162	390.1967	1167.5682	1167.5632	4.29	0	38	0.041	1		490s (8.17m)	K.LGIHEDSQNR.K
185	612.8139	1223.6133	1223.6186	-4.34	0	(44)	0.015	1	U	549s (9.15m)	K.HIYYITGETK.D
186	612.8139	1223.6133	1223.6186	-4.34	0	47	0.0077	1	U	546s (9.10m)	K.HIYYITGETK.D
206	646.3171	1290.6196	1290.6303	-8.30	0	75	8.1e-06	1		552s (9.20m)	R.ELISNSSDALDK.I
212	656.2859	1310.5573	1310.5626	-4.07	0	65	1.7e-05	1		534s (8.90m)	K.EDQTEYLEER.R
222	675.3679	1348.7212	1348.7272	-4.43	0	(77)	8.2e-06	1	U	763s (12.72m)	R.TLTIVDTGIGMTK.A
223	675.3679	1348.7212	1348.7272	-4.43	0	80	4e-06	1	U	760s (12.67m)	R.TLTIVDTGIGMTK.A
234	708.8239	1415.6333	1415.6303	2.07	0	50	0.0012	1		602s (10.03m)	K.EGLELPEDEEEK.K
253	757.3908	1512.7670	1512.7784	-7.52	0	77	7.4e-06	1		751s (12.52m)	R.GVVDSEDLPLNISR.E
263	513.9209	1538.7409	1538.7464	-3.57	1	59	0.00034	1		540s (9.00m)	R.YESLTDPSKLDSGK.E
287	558.2756	1671.8051	1671.8203	-9.07	2	5	79	3		533s (8.88m)	K.EGLELPEDEEEKKK.Q
310	917.3889	1832.7632	1832.7741	-5.94	0	(74)	1.3e-06	1	U	648s (10.80m)	R.NPDDITNEEYGEFYK.S

☑	311	917.3889	1832.7632	1832.7741	-5.94	0	93	1.3e-08	1	U	651s (10.85m)	R.NPDDITNEEYGEFYK.S
☑	328	672.3498	2014.0276	2014.0371	-4.69	1	104	1.3e-08	1		614s (10.23m)	K.VILHLKEDQTEYLEER.R
☑	329	672.3498	2014.0276	2014.0371	-4.69	1	(98)	5.8e-08	1		611s (10.18m)	K.VILHLKEDQTEYLEER.R

Proteins matching the same set of peptides:

HS90A_HORSE **Mass:** 84717 **Score:** 871 **Matches:** 21(18) **Sequences:** 16(13)
RecName: Full=Heat shock protein HSP 90-alpha; - OS=Equus caballus (Horse).
HS90A_HUMAN **Mass:** 84607 **Score:** 871 **Matches:** 21(18) **Sequences:** 16(13)
RecName: Full=Heat shock protein HSP 90-alpha; AltName: Full=Heat shock 86 kDa; Short=HSP 86; Short=HSP86; AltName: Full=Renal carcinoma
HS90A_MACFA **Mass:** 84736 **Score:** 871 **Matches:** 21(18) **Sequences:** 16(13)
RecName: Full=Heat shock protein HSP 90-alpha; - OS=Macaca fascicularis (Crab-eating macaque) (Cynomolgus monkey).
HS90A_PIG **Mass:** 84722 **Score:** 871 **Matches:** 21(18) **Sequences:** 16(13)
RecName: Full=Heat shock protein HSP 90-alpha; - OS=Sus scrofa (Pig).

---

2. HS90B_BOVIN **Mass:** 83201 **Score:** 769 **Matches:** 18(15) **Sequences:** 14(11) **emPAI:** 0.59
RecName: Full=Heat shock protein HSP 90-beta; - OS=Bos taurus (Bovine).
☐ Check to include this hit in error tolerant search or archive report

	Query	Observed	Mr(expt)	Mr(calc)	ppm	Miss	Score	Expect	Rank	Unique	Retention Time	Peptide
	78	451.2634	900.5122	900.5181	-6.62	0	12	36	3		543s (9.05m)	K.TKPIWTR.N
	123	520.2492	1038.4838	1038.4869	-3.01	0	42	0.01	1		516s (8.60m)	R.YESLTDPSK.L
	155	576.2807	1150.5469	1150.5506	-3.18	0	49	0.0034	1		518s (8.63m)	K.YIDQEELNK.T
☑	158	580.7917	1159.5688	1159.5761	-6.31	0	47	0.0052	1		601s (10.02m)	K.SIYYITGESK.E
☑	175	597.8228	1193.6310	1193.6404	-7.86	0	(56)	0.00087	1	U	667s (11.12m)	K.IDIIPNPQER.T
☑	176	597.8228	1193.6310	1193.6404	-7.86	0	70	3.3e-05	1	U	663s (11.05m)	K.IDIIPNPQER.T
☑	195	625.3044	1248.5943	1248.6098	-12.41	0	25	0.9	1	U	547s (9.12m)	K.EQVANSAFVER.V
	212	656.2859	1310.5573	1310.5626	-4.07	0	65	1.7e-05	1		534s (8.90m)	K.EDQTEYLEER.R
	222	675.3679	1348.7212	1348.7272	-4.43	0	(77)	8.2e-06	1	U	763s (12.72m)	R.TLTLVDTGIGMTK.A
	223	675.3679	1348.7212	1348.7272	-4.43	0	80	4e-06	1	U	760s (12.67m)	R.TLTLVDTGIGMTK.A
	234	708.8239	1415.6333	1415.6303	2.07	0	50	0.0012	1		602s (10.03m)	K.EGLELPEDEEEK.K
	253	757.3908	1512.7670	1512.7784	-7.52	0	77	7.4e-06	1		751s (12.52m)	R.GVVDSEDLPLNISR.E
	263	513.9209	1538.7409	1538.7464	-3.57	1	59	0.00034	1		540s (9.00m)	R.YESLTDPSKLDSGK.E
	287	558.2756	1671.8051	1671.8203	-9.07	2	5	79	3		533s (8.88m)	K.EGLELPEDEEEKKK.M
☑	316	924.4005	1846.7864	1846.7897	-1.77	0	82	2.8e-07	1	U	664s (11.07m)	R.NPDDITQEEYGEFYK.S
☑	317	924.4005	1846.7864	1846.7897	-1.77	0	(77)	8.8e-07	1	U	661s (11.02m)	R.NPDDITQEEYGEFYK.S
	328	672.3498	2014.0276	2014.0371	-4.69	1	104	1.3e-08	1		614s (10.23m)	K.VILHLKEDQTEYLEER.R
	329	672.3498	2014.0276	2014.0371	-4.69	1	(98)	5.8e-08	1		611s (10.18m)	K.VILHLKEDQTEYLEER.R

Proteins matching the same set of peptides:

HS90B_HORSE **Mass:** 83185 **Score:** 769 **Matches:** 18(15) **Sequences:** 14(11)
RecName: Full=Heat shock protein HSP 90-beta; - OS=Equus caballus (Horse).
HS90B_HUMAN **Mass:** 83212 **Score:** 769 **Matches:** 18(15) **Sequences:** 14(11)
RecName: Full=Heat shock protein HSP 90-beta; Short=HSP 90; AltName: Full=Heat shock 84 kDa; Short=HSP 84; Short=HSP84; - OS=Homo sapier
HS90B_MACFA **Mass:** 83185 **Score:** 769 **Matches:** 18(15) **Sequences:** 14(11)
RecName: Full=Heat shock protein HSP 90-beta; - OS=Macaca fascicularis (Crab-eating macaque) (Cynomolgus monkey).
HS90B_MOUSE **Mass:** 83229 **Score:** 769 **Matches:** 18(15) **Sequences:** 14(11)
RecName: Full=Heat shock protein HSP 90-beta; AltName: Full=Heat shock 84 kDa; Short=HSP 84; Short=HSP84; AltName: Full=Tumor-specific t
HS90B_PONAB **Mass:** 83186 **Score:** 769 **Matches:** 18(15) **Sequences:** 14(11)
RecName: Full=Heat shock protein HSP 90-beta; - OS=Pongo abelii (Sumatran orangutan) (Pongo pygmaeus abelii).
HS90B_RAT **Mass:** 83229 **Score:** 769 **Matches:** 18(15) **Sequences:** 14(11)
RecName: Full=Heat shock protein HSP 90-beta; AltName: Full=Heat shock 84 kDa; Short=HSP 84; Short=HSP84; - OS=Rattus norvegicus (Rat).

---

3. ACTN4_BOVIN **Mass:** 104862 **Score:** 599 **Matches:** 16(11) **Sequences:** 11(7) **emPAI:** 0.28
RecName: Full=Alpha-actinin-4; AltName: Full=F-actin cross-linking protein; AltName: Full=Non-muscle alpha-actinin 4; - OS=Bos taurus (E
☐ Check to include this hit in error tolerant search or archive report

	Query	Observed	Mr(expt)	Mr(calc)	ppm	Miss	Score	Expect	Rank	Unique	Retention Time	Peptide
☑	39	406.2542	810.4938	810.4963	-3.12	0	23	0.43	1	U	526s (8.77m)	K.VQQLVPK.R
☑	203	645.2966	1288.5787	1288.5823	-2.76	0	12	7.6	1		831s (13.85m)	K.SFSTALYGESDL.-
☑	209	433.8918	1298.6535	1298.6619	-6.41	0	31	0.23	1		523s (8.72m)	R.HRPELIEYDK.L
	214	442.5639	1324.6700	1324.6735	-2.64	1	15	13	2	U	492s (8.20m)	K.RDHALLEEQSK.Q
☑	224	676.8147	1351.6149	1351.6190	-3.07	0	(66)	4.1e-05	1		582s (9.70m)	K.GISQEQMQEFR.A
☑	225	676.8147	1351.6149	1351.6190	-3.07	0	82	1.1e-06	1		578s (9.63m)	K.GISQEQMQEFR.A
☑	261	769.3855	1536.7564	1536.7671	-6.98	0	(35)	0.12	1		724s (12.07m)	R.FAIQDISVEETSAK.E
☑	262	769.3855	1536.7564	1536.7671	-6.98	0	52	0.0022	1		719s (11.98m)	R.FAIQDISVEETSAK.E
☑	288	559.3030	1674.8871	1674.8941	-4.13	1	90	5e-07	1	U	679s (11.32m)	K.VLAGDKNFITAEELR.R
☑	73	871.4066	1740.7986	1740.8054	-3.88	0	(51)	0.0033	1		764s (12.73m)	R.ETTDTDTADQVIASFK.V
☑	293	871.4070	1740.7995	1740.8054	-3.38	0	93	1e-07	1		761s (12.68m)	R.ETTDTDTADQVIASFK.V
☑	302	896.9307	1791.8468	1791.8502	-1.89	0	(51)	0.0022	1	U	707s (11.78m)	R.MAPYQGPDAVPGALDYK.S
☑	303	896.9307	1791.8468	1791.8502	-1.89	0	79	3.5e-06	1	U	703s (11.72m)	R.MAPYQGPDAVPGALDYK.S
☑	307	606.2939	1815.8599	1815.8672	-4.06	0	(48)	0.0035	1		677s (11.28m)	K.MLDAEDIVNTARPDEK.A
☑	308	606.2939	1815.8599	1815.8672	-4.06	0	63	0.00013	1		674s (11.23m)	K.MLDAEDIVNTARPDEK.A
☑	323	640.9799	1919.9179	1919.9225	-2.39	1	61	0.00022	1		717s (11.95m)	K.AGTQIENIDEDFRDGLK.L

Proteins matching the same set of peptides:

ACTN4_HUMAN **Mass:** 104788 **Score:** 599 **Matches:** 16(11) **Sequences:** 11(7)
RecName: Full=Alpha-actinin-4; AltName: Full=F-actin cross-linking protein; AltName: Full=Non-muscle alpha-actinin 4; - OS=Homo sapiens
ACTN4_PONAB **Mass:** 104789 **Score:** 599 **Matches:** 16(11) **Sequences:** 11(7)
RecName: Full=Alpha-actinin-4; AltName: Full=F-actin cross-linking protein; AltName: Full=Non-muscle alpha-actinin 4; - OS=Pongo abelii

---

4. CO3_HUMAN **Mass:** 187030 **Score:** 503 **Matches:** 8(8) **Sequences:** 7(7) **emPAI:** 0.13
RecName: Full=Complement C3; AltName: Full=C3 and PZP-like alpha-2-macroglobulin domain-containing protein 1; Contains: RecName: Full=Co
☐ Check to include this hit in error tolerant search or archive report

	Query	Observed	Mr(expt)	Mr(calc)	ppm	Miss	Score	Expect	Rank	Unique	Retention Time	Peptide
☑	74	443.7646	885.5147	885.5171	-2.73	0	(47)	0.0098	1	U	659s (10.98m)	R.ISLPESLK.R
☑	75	443.7646	885.5147	885.5171	-2.73	0	50	0.0043	1	U	656s (10.93m)	R.ISLPESLK.R
☑	111	501.7765	1001.5383	1001.5393	-0.97	0	56	0.0013	1	U	548s (9.13m)	K.TGLQEVEVK.A
☑	245	735.8875	1469.7604	1469.7726	-8.30	0	111	2.9e-09	1	U	650s (10.83m)	R.IPIEDGSGEVVLSR.K
☑	320	619.2862	1854.8368	1854.8483	-6.20	1	39	0.015	1	U	521s (8.68m)	R.SEETKENEGFTVTAEGK.G
☑	331	719.6991	2156.0755	2156.0783	-1.29	0	83	1.7e-06	1	U	964s (16.07m)	R.ILLQGTPVAQMTEDAVDAER.L
☑	332	733.3800	2197.1182	2197.1227	-2.05	0	81	2.9e-06	1	U	697s (11.62m)	R.VPVAVQGEDTVQSLTQGDGVAK.L
☑	83	919.0988	2754.2745	2754.2832	-3.16	1	84	7.5e-07	1	U	583s (9.72m)	R.EGVQKEDIPPADLSDQVPDTESETR.I

5. ACTN4_CHICK **Mass:** 104142 **Score:** 416 **Matches:** 13(8) **Sequences:** 8(5) **emPAI:** 0.20

RecName: Full=Alpha-actinin-4; AltName: Full=F-actin cross-linking protein; AltName: Full=Non-muscle alpha-actinin 4; - OS=Gallus gallus

☐ Check to include this hit in error tolerant search or archive report

Query	Observed	Mr(expt)	Mr(calc)	ppm	Miss	Score	Expect	Rank	Unique	Retention Time	Peptide
203	645.2966	1288.5787	1288.5823	-2.76	0	12	7.6	1		831s (13.85m)	K.SFSTALYGESDL.-
209	433.8918	1298.6535	1298.6619	-6.41	0	31	0.23	1		523s (8.72m)	R.HRPELIEYDK.L
224	676.8147	1351.6149	1351.6190	-3.07	0	(66)	4.1e-05	1		582s (9.70m)	K.GISQEQMQEFR.A
225	676.8147	1351.6149	1351.6190	-3.07	0	82	1.1e-06	1		578s (9.63m)	K.GISQEQMQEFR.A
261	769.3855	1536.7564	1536.7671	-6.98	0	(35)	0.12	1		724s (12.07m)	R.FAIQDISVEETSAK.E
262	769.3855	1536.7564	1536.7671	-6.98	0	52	0.0022	1		719s (11.98m)	R.FAIQDISVEETSAK.E
73	871.4066	1740.7986	1740.8054	-3.88	0	(51)	0.0033	1		764s (12.73m)	R.ETTDTDTADQVIASFK.V
293	871.4070	1740.7995	1740.8054	-3.38	0	93	1e-07	1		761s (12.68m)	R.ETTDTDTADQVIASFK.V
302	896.9307	1791.8468	1791.8614	-8.15	1	(12)	14	2	U	707s (11.78m)	R.MAPYRGPDAAPGALDYK.S
303	896.9307	1791.8468	1791.8614	-8.15	1	23	1.2	2	U	703s (11.72m)	R.MAPYRGPDAAPGALDYK.S
307	606.2939	1815.8599	1815.8672	-4.06	0	(48)	0.0035	1		677s (11.28m)	K.MLDAEDIVNTARPDEK.A
308	606.2939	1815.8599	1815.8672	-4.06	0	63	0.00013	1		674s (11.23m)	K.MLDAEDIVNTARPDEK.A
323	640.9799	1919.9179	1919.9225	-2.39	1	61	0.00022	1		717s (11.95m)	K.AGTQIENIDEDFRDGLK.L

6. LG3BP_HUMAN **Mass:** 65289 **Score:** 374 **Matches:** 9(8) **Sequences:** 5(4) **emPAI:** 0.28

RecName: Full=Galectin-3-binding protein; AltName: Full=Basement membrane autoantigen p105; AltName: Full=Lectin galactoside-binding sol

☐ Check to include this hit in error tolerant search or archive report

	Query	Observed	Mr(expt)	Mr(calc)	ppm	Miss	Score	Expect	Rank	Unique	Retention Time	Peptide
☑	120	515.2681	1028.5216	1028.5251	-3.40	0	32	0.15	1	U	512s (8.53m)	R.STHTLDLSR.E
☑	216	442.8876	1325.6409	1325.6463	-4.10	0	(58)	0.00041	1	U	523s (8.72m)	R.ASHEEVEGLVEK.I
☑	217	442.8876	1325.6409	1325.6463	-4.10	0	69	3.4e-05	1	U	519s (8.65m)	R.ASHEEVEGLVEK.I
☑	226	678.3897	1354.7648	1354.7708	-4.36	0	(61)	0.0002	1	U	959s (15.98m)	R.SDLAVPSELALLK.A
☑	227	678.3897	1354.7648	1354.7708	-4.36	0	66	6e-05	1	U	962s (16.03m)	R.SDLAVPSELALLK.A
☑	272	796.8958	1591.7770	1591.7842	-4.49	0	110	3.1e-09	1	U	944s (15.73m)	R.ELSEALGQIFDSQR.G
☑	273	796.8962	1591.7778	1591.7842	-3.98	0	(78)	5.4e-06	1	U	941s (15.68m)	R.ELSEALGQIFDSQR.G
☑	274	799.8368	1597.6589	1597.6685	-5.97	0	(87)	6.7e-08	1	U	631s (10.52m)	K.YSSDYFQAPSDYR.Y
☑	275	799.8368	1597.6589	1597.6685	-5.97	0	96	7.7e-09	1	U	627s (10.45m)	K.YSSDYFQAPSDYR.Y

Proteins matching the same set of peptides:

LG3BP_PONAB **Mass:** 65225 **Score:** 374 **Matches:** 9(8) **Sequences:** 5(4)

RecName: Full=Galectin-3-binding protein; AltName: Full=Lectin galactoside-binding soluble 3-binding protein; Flags: Precursor; - OS=Por

7. HSP83_DROPS **Mass:** 81720 **Score:** 299 **Matches:** 7(5) **Sequences:** 6(4) **emPAI:** 0.17

RecName: Full=Heat shock protein 83; AltName: Full=HSP 82; - OS=Drosophila pseudoobscura pseudoobscura (Fruit fly).

☐ Check to include this hit in error tolerant search or archive report

Query	Observed	Mr(expt)	Mr(calc)	ppm	Miss	Score	Expect	Rank	Unique	Retention Time	Peptide
78	451.2634	900.5122	900.5181	-6.62	0	12	36	3		543s (9.05m)	K.TKPIWTR.N
94	474.7237	947.4328	947.4389	-6.44	0	33	0.077	1		562s (9.37m)	K.FYEQFSK.N
123	520.2492	1038.4838	1038.4869	-3.01	0	42	0.01	1		516s (8.60m)	R.YESLTDPSK.L
253	757.3908	1512.7670	1512.7784	-7.52	0	77	7.4e-06	1		751s (12.52m)	K.GVVDSEDLPLNISR.E
263	513.9209	1538.7409	1538.7464	-3.57	1	59	0.00034	1		540s (9.00m)	R.YESLTDPSKLDSGK.E
310	917.3889	1832.7632	1832.7741	-5.94	0	(56)	7.3e-05	2	U	648s (10.80m)	R.NPDDISQEEYGEFYK.S
311	917.3889	1832.7632	1832.7741	-5.94	0	76	7.2e-07	2	U	651s (10.85m)	R.NPDDISQEEYGEFYK.S

8. PDIA1_HUMAN **Mass:** 57081 **Score:** 234 **Matches:** 4(3) **Sequences:** 4(3) **emPAI:** 0.18

RecName: Full=Protein disulfide-isomerase; Short=PDI; EC=5.3.4.1; AltName: Full=Cellular thyroid hormone-binding protein; AltName: Full=

☐ Check to include this hit in error tolerant search or archive report

	Query	Observed	Mr(expt)	Mr(calc)	ppm	Miss	Score	Expect	Rank	Unique	Retention Time	Peptide
☑	66	431.7330	861.4515	861.4596	-9.37	0	22	3.2	1	U	519s (8.65m)	K.ALAPEYAK.A
☑	179	601.8021	1201.5897	1201.5979	-6.81	0	47	0.0061	1	U	912s (15.20m)	R.EADDIVNWLK.K
☑	10	484.5696	1450.6870	1450.6939	-4.80	0	67	5.3e-05	1	U	505s (8.42m)	K.YKPESEELTAER.I
☑	299	890.9167	1779.8188	1779.8275	-4.91	0	99	2.2e-08	1	U	615s (10.25m)	K.VDATEESDLAQQYGVR.G

Proteins matching the same set of peptides:

PDIA1_PONAB **Mass:** 56998 **Score:** 234 **Matches:** 4(3) **Sequences:** 4(3)

RecName: Full=Protein disulfide-isomerase; Short=PDI; EC=5.3.4.1; AltName: Full=Cellular thyroid hormone-binding protein; AltName: Full=

9. PGBM_HUMAN **Mass:** 468532 **Score:** 225 **Matches:** 3(3) **Sequences:** 3(3) **emPAI:** 0.02

RecName: Full=Basement membrane-specific heparan sulfate proteoglycan core protein; Short=HSPG; AltName: Full=Perlecan; Short=PLC; Conta

☐ Check to include this hit in error tolerant search or archive report

Query	Observed	Mr(expt)	Mr(calc)	ppm	Miss	Score	Expect	Rank	Unique	Retention Time	Peptide

Query	Observed	Mr(expt)	Mr(calc)	ppm	Miss	Score	Expect	Rank	Unique	Retention Time	Peptide
142	563.8238	1125.6330	1125.6394	-5.61	0	63	0.00012	1	U	733s (12.22m)	R.LEGDTLIIPR.V
264	776.3678	1550.7210	1550.7212	-0.15	0	76	4.9e-06	1	U	579s (9.65m)	R.SIEYSPQLEDAGSR.E
335	770.7535	2309.2387	2309.2478	-3.96	0	86	7.4e-07	1	U	720s (12.00m)	R.AELLVTEAPSKPITVTVEEQR.S

10. HSP90_BRUPA **Mass:** 82553 **Score:** 225 **Matches:** 6(3) **Sequences:** 5(3) **emPAI:** 0.12
RecName: Full=Heat shock protein 90; - OS=Brugia pahangi (Filarial nematode worm).
Check to include this hit in error tolerant search or archive report

Query	Observed	Mr(expt)	Mr(calc)	ppm	Miss	Score	Expect	Rank	Unique	Retention Time	Peptide
78	451.2634	900.5122	900.5181	-6.62	0	12	36	3		543s (9.05m)	K.TKPIWTR.N
94	474.7237	947.4328	947.4389	-6.44	0	33	0.077	1		562s (9.37m)	K.FYEQFSK.N
206	646.3171	1290.6196	1290.6303	-8.30	0	75	8.1e-06	1		552s (9.20m)	R.ELISNSSDALDK.I
253	757.3908	1512.7670	1512.7784	-7.52	0	77	7.4e-06	1		751s (12.52m)	K.GVVDSEDLPLNISR.E
310	917.3889	1832.7632	1832.7740	-5.94	0	(19)	0.35	3		648s (10.80m)	R.NPDDISNEEYAEFYK.S
311	917.3889	1832.7632	1832.7740	-5.94	0	28	0.047	3		651s (10.85m)	R.NPDDISNEEYAEFYK.S

11. HS90B_DANRE **Mass:** 83305 **Score:** 222 **Matches:** 5(4) **Sequences:** 5(4) **emPAI:** 0.17
RecName: Full=Heat shock protein HSP 90-beta; - OS=Danio rerio (Zebrafish) (Brachydanio rerio).
Check to include this hit in error tolerant search or archive report

Query	Observed	Mr(expt)	Mr(calc)	ppm	Miss	Score	Expect	Rank	Unique	Retention Time	Peptide
78	451.2634	900.5122	900.5181	-6.62	0	12	36	3		543s (9.05m)	K.TKPIWTR.N
155	576.2807	1150.5469	1150.5506	-3.18	0	49	0.0034	1		518s (8.63m)	K.YIDQEELNK.T
158	580.7917	1159.5688	1159.5761	-6.31	0	47	0.0052	1		601s (10.02m)	K.SIYYITGESK.D
162	390.1967	1167.5682	1167.5632	4.29	0	38	0.041	1		490s (8.17m)	K.LGIHEDSQNR.K
253	757.3908	1512.7670	1512.7784	-7.52	0	77	7.4e-06	1		751s (12.52m)	R.GVVDSEDLPLNISR.E

12. 1433B_BOVIN **Mass:** 28064 **Score:** 210 **Matches:** 6(3) **Sequences:** 4(3) **emPAI:** 0.40
RecName: Full=14-3-3 protein beta/alpha; AltName: Full=Protein kinase C inhibitor protein 1; Short=KCIP-1; Contains: RecName: Full=14-3-
Check to include this hit in error tolerant search or archive report

Query	Observed	Mr(expt)	Mr(calc)	ppm	Miss	Score	Expect	Rank	Unique	Retention Time	Peptide
79	452.2618	902.5091	902.5073	1.98	0	37	0.082	1	U	506s (8.43m)	R.VISSIEQK.T
80	454.2613	906.5080	906.5174	-10.43	0	(36)	0.089	1		669s (11.15m)	R.NLLSVAYK.N
81	454.2613	906.5080	906.5174	-10.43	0	40	0.032	1		672s (11.20m)	R.NLLSVAYK.N
167	591.7844	1181.5542	1181.5564	-1.87	0	(15)	6.6	1	U	514s (8.57m)	R.YLSEVASGDNK.Q
168	591.7844	1181.5542	1181.5564	-1.87	0	54	0.00074	1	U	510s (8.50m)	R.YLSEVASGDNK.Q
276	533.5833	1597.7279	1597.7332	-3.30	0	78	2.6e-06	1	U	487s (8.12m)	K.AVTEQGHELSNEER.N

Proteins matching the same set of peptides:
1433B_CHICK **Mass:** 27890 **Score:** 210 **Matches:** 6(3) **Sequences:** 4(3)
RecName: Full=14-3-3 protein beta/alpha; - OS=Gallus gallus (Chicken).
1433B_HUMAN **Mass:** 28065 **Score:** 210 **Matches:** 6(3) **Sequences:** 4(3)
RecName: Full=14-3-3 protein beta/alpha; AltName: Full=Protein 1054; AltName: Full=Protein kinase C inhibitor protein 1; Short=KCIP-1; C
1433B_MACFA **Mass:** 28065 **Score:** 210 **Matches:** 6(3) **Sequences:** 4(3)
RecName: Full=14-3-3 protein beta/alpha; Contains: RecName: Full=14-3-3 protein beta/alpha, N-terminally processed; - OS=Macaca fascicul
1433B_PONAB **Mass:** 28065 **Score:** 210 **Matches:** 6(3) **Sequences:** 4(3)
RecName: Full=14-3-3 protein beta/alpha; Contains: RecName: Full=14-3-3 protein beta/alpha, N-terminally processed; - OS=Pongo abelii (S
1433B_RAT **Mass:** 28037 **Score:** 210 **Matches:** 6(3) **Sequences:** 4(3)
RecName: Full=14-3-3 protein beta/alpha; AltName: Full=Prepronerve growth factor RNH-1; AltName: Full=Protein kinase C inhibitor protein
1433B_SHEEP **Mass:** 22040 **Score:** 210 **Matches:** 6(3) **Sequences:** 4(3)
RecName: Full=14-3-3 protein beta/alpha; AltName: Full=Protein kinase C inhibitor protein 1; Short=KCIP-1; Contains: RecName: Full=14-3-

13. BGH3_HUMAN **Mass:** 74634 **Score:** 208 **Matches:** 6(5) **Sequences:** 4(3) **emPAI:** 0.14
RecName: Full=Transforming growth factor-beta-induced protein ig-h3; Short=Beta ig-h3; AltName: Full=Kerato-epithelin; AltName: Full=RGD
Check to include this hit in error tolerant search or archive report

Query	Observed	Mr(expt)	Mr(calc)	ppm	Miss	Score	Expect	Rank	Unique	Retention Time	Peptide
136	552.2628	1102.5110	1102.5117	-0.69	0	51	0.0013	1	U	748s (12.47m)	R.YGTLFTMDR.V
137	552.2628	1102.5110	1102.5117	-0.69	0	(34)	0.058	1	U	750s (12.50m)	R.YGTLFTMDR.V
214	442.5639	1324.6700	1324.6484	16.3	0	7	73	4	U	492s (8.20m)	R.QAGLGNHLSGSER.L
232	704.3520	1406.6895	1406.6929	-2.40	0	66	7.5e-05	1	U	1007s (16.78m)	R.GDELADSALEIFK.Q
58	850.9095	1699.8045	1699.8206	-9.48	0	84	1e-06	1	U	860s (14.33m)	R.EGVYTVFAPTNEAFR.A
59	850.9147	1699.8149	1699.8206	-3.35	0	(62)	0.00016	1	U	864s (14.40m)	R.EGVYTVFAPTNEAFR.A

14. AGRIN_HUMAN **Mass:** 214706 **Score:** 186 **Matches:** 3(3) **Sequences:** 3(3) **emPAI:** 0.05
RecName: Full=Agrin; Flags: Precursor; - OS=Homo sapiens (Human).
Check to include this hit in error tolerant search or archive report

Query	Observed	Mr(expt)	Mr(calc)	ppm	Miss	Score	Expect	Rank	Unique	Retention Time	Peptide
114	505.2498	1008.4850	1008.4876	-2.60	0	56	0.00066	1	U	585s (9.75m)	K.SELFGETAR.S
208	648.3245	1294.6344	1294.6405	-4.71	0	69	4e-05	1	U	1178s (19.63m)	R.SIESTLDDLFR.N
228	690.3446	1378.6746	1378.6769	-1.65	0	61	0.00024	1	U	683s (11.38m)	K.EPLYVGGAPDFSK.L

Proteins matching the same set of peptides:
AGRIN_MOUSE **Mass:** 207403 **Score:** 185 **Matches:** 3(3) **Sequences:** 3(3)
RecName: Full=Agrin; Flags: Precursor; - OS=Mus musculus (Mouse).

15. K2C1_HUMAN **Mass:** 65999 **Score:** 170 **Matches:** 2(2) **Sequences:** 2(2) **emPAI:** 0.10
RecName: Full=Keratin, type II cytoskeletal 1; AltName: Full=67 kDa cytokeratin; AltName: Full=Cytokeratin-1; Short=CK-1; AltName: Full=

☐ Check to include this hit in error tolerant search or archive report

	Query	Observed	Mr(expt)	Mr(calc)	ppm	Miss	Score	Expect	Rank	Unique	Retention Time	Peptide
☑	18	639.3510	1276.6873	1276.7027	-12.00	0	72	6.1e-05	1	U	929s (15.48m)	K.LALDLEIATYR.T
☑	291	563.3248	1686.9525	1686.9628	-6.12	1	98	3.5e-08	1	U	716s (11.93m)	R.SLVNLGGSKSISISVAR.G

Proteins matching the same set of peptides:
K2C1_PANTR **Mass:** 65450 **Score:** 170 **Matches:** 2(2) **Sequences:** 2(2)
RecName: Full=Keratin, type II cytoskeletal 1; AltName: Full=Cytokeratin-1; Short=CK-1; AltName: Full=Keratin-1; Short=K1; AltName: Full

---

16. ACT_MANSE **Mass:** 41750 **Score:** 167 **Matches:** 6(5) **Sequences:** 4(3) **emPAI:** 0.36
RecName: Full=Actin, muscle; Flags: Precursor; - OS=Manduca sexta (Tobacco hawkmoth) (Tobacco hornworm).
☐ Check to include this hit in error tolerant search or archive report

	Query	Observed	Mr(expt)	Mr(calc)	ppm	Miss	Score	Expect	Rank	Unique	Retention Time	Peptide
☑	31	398.2371	794.4596	794.4650	-6.85	0	(37)	0.024	1		529s (8.82m)	K.IIAPPER.K
☑	32	398.2371	794.4596	794.4650	-6.85	0	41	0.0096	1		526s (8.77m)	K.IIAPPER.K
☑	145	566.7660	1131.5175	1131.5197	-1.91	0	55	0.00045	1		556s (9.27m)	R.GYSFTTTAER.E
☑	164	586.2857	1170.5568	1170.5638	-5.99	0	27	0.45	1		513s (8.55m)	R.HQGVMVGMGQK.D
☑	326	654.3068	1959.8985	1959.9036	-2.65	0	44	0.006	1		741s (12.35m)	K.YPIEHGIITNWDDMEK.I
☑	327	654.3068	1959.8985	1959.9036	-2.65	0	(36)	0.037	1		745s (12.42m)	K.YPIEHGIITNWDDMEK.I

Proteins matching the same set of peptides:
ACT_MAYDE **Mass:** 41790 **Score:** 167 **Matches:** 6(5) **Sequences:** 4(3)
RecName: Full=Actin; Flags: Precursor; - OS=Mayetiola destructor (Hessian fly).
ACT1_ARTSX **Mass:** 41759 **Score:** 167 **Matches:** 6(5) **Sequences:** 4(3)
RecName: Full=Actin, clone 205; Flags: Precursor; - OS=Artemia sp. (Brine shrimp).
ACT1_BACDO **Mass:** 41673 **Score:** 167 **Matches:** 6(5) **Sequences:** 4(3)
RecName: Full=Actin, indirect flight muscle; Flags: Precursor; - OS=Bactrocera dorsalis (Oriental fruit fly) (Dacus dorsalis).
ACT2_ARTSX **Mass:** 41757 **Score:** 167 **Matches:** 6(5) **Sequences:** 4(3)
RecName: Full=Actin, clone 211; Flags: Precursor; - OS=Artemia sp. (Brine shrimp).
ACT2_BACDO **Mass:** 41776 **Score:** 167 **Matches:** 6(5) **Sequences:** 4(3)
RecName: Full=Actin-2, muscle-specific; Flags: Precursor; - OS=Bactrocera dorsalis (Oriental fruit fly) (Dacus dorsalis).
ACT2_BOMMO **Mass:** 41776 **Score:** 167 **Matches:** 6(5) **Sequences:** 4(3)
RecName: Full=Actin, muscle-type A2; Flags: Precursor; - OS=Bombyx mori (Silk moth).
ACT3_BACDO **Mass:** 41789 **Score:** 167 **Matches:** 6(5) **Sequences:** 4(3)
RecName: Full=Actin-3, muscle-specific; Flags: Precursor; - OS=Bactrocera dorsalis (Oriental fruit fly) (Dacus dorsalis).
ACT3_DROME **Mass:** 41808 **Score:** 167 **Matches:** 6(5) **Sequences:** 4(3)
RecName: Full=Actin-57B; Flags: Precursor; - OS=Drosophila melanogaster (Fruit fly).
ACT4_DROME **Mass:** 41760 **Score:** 167 **Matches:** 6(5) **Sequences:** 4(3)
RecName: Full=Actin, larval muscle; AltName: Full=Actin-79B; Flags: Precursor; - OS=Drosophila melanogaster (Fruit fly).
ACT5_BACDO **Mass:** 41744 **Score:** 167 **Matches:** 6(5) **Sequences:** 4(3)
RecName: Full=Actin-5, muscle-specific; Flags: Precursor; - OS=Bactrocera dorsalis (Oriental fruit fly) (Dacus dorsalis).
ACT5_DROME **Mass:** 41775 **Score:** 167 **Matches:** 6(5) **Sequences:** 4(3)
RecName: Full=Actin-87E; Flags: Precursor; - OS=Drosophila melanogaster (Fruit fly).
ACT6_DROME **Mass:** 41673 **Score:** 167 **Matches:** 6(5) **Sequences:** 4(3)
RecName: Full=Actin, indirect flight muscle; AltName: Full=Actin-88F; Flags: Precursor; - OS=Drosophila melanogaster (Fruit fly).
ACT6_DROSI **Mass:** 41673 **Score:** 167 **Matches:** 6(5) **Sequences:** 4(3)
RecName: Full=Actin, indirect flight muscle; AltName: Full=Actin-88F; Flags: Precursor; - OS=Drosophila simulans (Fruit fly).

---

17. H2B1B_MOUSE **Mass:** 13944 **Score:** 162 **Matches:** 5(5) **Sequences:** 3(3) **emPAI:** 0.92
RecName: Full=Histone H2B type 1-B; AltName: Full=h2B-143; - OS=Mus musculus (Mouse).
☐ Check to include this hit in error tolerant search or archive report

	Query	Observed	Mr(expt)	Mr(calc)	ppm	Miss	Score	Expect	Rank	Unique	Retention Time	Peptide
☑	95	477.3030	952.5915	952.5957	-4.35	0	(45)	0.0021	1		774s (12.90m)	R.LLLPGELAK.H
☑	96	477.3030	952.5915	952.5957	-4.35	0	50	0.00079	1		769s (12.82m)	R.LLLPGELAK.H
☑	148	569.2765	1136.5384	1136.5390	-0.50	0	56	0.00058	1	U	599s (9.98m)	K.ESYSVYVYK.V
☑	149	569.2765	1136.5384	1136.5390	-0.50	0	(50)	0.0024	1	U	596s (9.93m)	K.ESYSVYVYK.V
☑	163	390.1972	1167.5698	1167.5884	-15.96	0	56	0.00064	1		482s (8.03m)	K.QVHPDTGISSK.A

Proteins matching the same set of peptides:
H2B1C_HUMAN **Mass:** 13898 **Score:** 162 **Matches:** 5(5) **Sequences:** 3(3)
RecName: Full=Histone H2B type 1-C/E/F/G/I; AltName: Full=Histone H2B.1 A; AltName: Full=Histone H2B.a; Short=H2B/a; AltName: Full=Histo
H2B1C_MOUSE **Mass:** 13898 **Score:** 162 **Matches:** 5(5) **Sequences:** 3(3)
RecName: Full=Histone H2B type 1-C/E/G; - OS=Mus musculus (Mouse).
H2B1D_HUMAN **Mass:** 13928 **Score:** 162 **Matches:** 5(5) **Sequences:** 3(3)
RecName: Full=Histone H2B type 1-D; AltName: Full=HIRA-interacting protein 2; AltName: Full=Histone H2B.1 B; AltName: Full=Histone H2B.b
H2B1F_MOUSE **Mass:** 13928 **Score:** 162 **Matches:** 5(5) **Sequences:** 3(3)
RecName: Full=Histone H2B type 1-F/J/L; AltName: Full=H2B 291A; - OS=Mus musculus (Mouse).
H2B1H_HUMAN **Mass:** 13884 **Score:** 162 **Matches:** 5(5) **Sequences:** 3(3)
RecName: Full=Histone H2B type 1-H; AltName: Full=Histone H2B.j; Short=H2B/j; - OS=Homo sapiens (Human).
H2B1H_MOUSE **Mass:** 13912 **Score:** 162 **Matches:** 5(5) **Sequences:** 3(3)
RecName: Full=Histone H2B type 1-H; AltName: Full=h2B-221; - OS=Mus musculus (Mouse).
H2B1K_BOVIN **Mass:** 13867 **Score:** 162 **Matches:** 5(5) **Sequences:** 3(3)
RecName: Full=Histone H2B type 1-K; - OS=Bos taurus (Bovine).
H2B1K_HUMAN **Mass:** 13882 **Score:** 162 **Matches:** 5(5) **Sequences:** 3(3)
RecName: Full=Histone H2B type 1-K; Short=H2B K; AltName: Full=HIRA-interacting protein 1; - OS=Homo sapiens (Human).
H2B1K_MOUSE **Mass:** 13912 **Score:** 162 **Matches:** 5(5) **Sequences:** 3(3)
RecName: Full=Histone H2B type 1-K; - OS=Mus musculus (Mouse).
H2B1L_HUMAN **Mass:** 13944 **Score:** 162 **Matches:** 5(5) **Sequences:** 3(3)
RecName: Full=Histone H2B type 1-L; AltName: Full=Histone H2B.c; Short=H2B/c; - OS=Homo sapiens (Human).

H2B1M_HUMAN Mass: 13981 Score: 162 Matches: 5(5) Sequences: 3(3)
RecName: Full=Histone H2B type 1-M; AltName: Full=Histone H2B.e; Short=H2B/e; - OS=Homo sapiens (Human).
H2B1M_MOUSE Mass: 13928 Score: 162 Matches: 5(5) Sequences: 3(3)
RecName: Full=Histone H2B type 1-M; AltName: Full=H2B 291B; - OS=Mus musculus (Mouse).
H2B1N_BOVIN Mass: 13915 Score: 162 Matches: 5(5) Sequences: 3(3)
RecName: Full=Histone H2B type 1-N; - OS=Bos taurus (Bovine).
H2B1N_HUMAN Mass: 13914 Score: 162 Matches: 5(5) Sequences: 3(3)
RecName: Full=Histone H2B type 1-N; AltName: Full=Histone H2B.d; Short=H2B/d; - OS=Homo sapiens (Human).
H2B1P_MOUSE Mass: 13984 Score: 162 Matches: 5(5) Sequences: 3(3)
RecName: Full=Histone H2B type 1-P; - OS=Mus musculus (Mouse).
H2B1_BOVIN Mass: 13898 Score: 162 Matches: 5(5) Sequences: 3(3)
RecName: Full=Histone H2B type 1; - OS=Bos taurus (Bovine).
H2B1_PONAB Mass: 13898 Score: 162 Matches: 5(5) Sequences: 3(3)
RecName: Full=Histone H2B type 1; - OS=Pongo abelii (Sumatran orangutan) (Pongo pygmaeus abelii).
H2B1_RAT Mass: 13982 Score: 162 Matches: 5(5) Sequences: 3(3)
RecName: Full=Histone H2B type 1; - OS=Rattus norvegicus (Rat).
H2B2B_MOUSE Mass: 13912 Score: 162 Matches: 5(5) Sequences: 3(3)
RecName: Full=Histone H2B type 2-B; AltName: Full=H2b 616; - OS=Mus musculus (Mouse).
H2B2F_HUMAN Mass: 13912 Score: 162 Matches: 5(5) Sequences: 3(3)
RecName: Full=Histone H2B type 2-F; - OS=Homo sapiens (Human).
H2BFS_HUMAN Mass: 13936 Score: 162 Matches: 5(5) Sequences: 3(3)
RecName: Full=Histone H2B type F-S; AltName: Full=Histone H2B.s; Short=H2B/s; - OS=Homo sapiens (Human).

---

18. K2C8_HUMAN Mass: 53671 Score: 158 Matches: 3(3) Sequences: 2(2) **emPAI:** 0.13
RecName: Full=Keratin, type II cytoskeletal 8; AltName: Full=Cytokeratin-8; Short=CK-8; AltName: Full=Keratin-8; Short=K8; AltName: Full
☐ Check to include this hit in error tolerant search or archive report

	Query	Observed	Mr(expt)	Mr(calc)	ppm	Miss	Score	Expect	Rank	Unique	Retention Time	Peptide
	18	639.3510	1276.6873	1276.7027	-12.00	0	72	6.1e-05	1		929s (15.48m)	K.LALDIEIATYR.K
☑	235	710.3747	1418.7349	1418.7405	-3.94	0	(68)	7.3e-05	1	U	994s (16.57m)	R.LEGLTDEINFLR.Q
☑	236	710.3747	1418.7349	1418.7405	-3.94	0	86	1.1e-06	1	U	990s (16.50m)	R.LEGLTDEINFLR.Q

Proteins matching the same set of peptides:
K2C8_MESAU Mass: 12966 Score: 158 Matches: 3(3) Sequences: 2(2)
RecName: Full=Keratin, type II cytoskeletal 8; AltName: Full=Cytokeratin-8; Short=CK-8; AltName: Full=Keratin-8; Short=K8; Flags: Fragme
K2C8_RAT Mass: 53985 Score: 158 Matches: 3(3) Sequences: 2(2)
RecName: Full=Keratin, type II cytoskeletal 8; AltName: Full=Cytokeratin endo A; AltName: Full=Cytokeratin-8; Short=CK-8; AltName: Full=

---

19. TRYP_PIG Mass: 24394 Score: 147 Matches: 9(9) Sequences: 2(2) **emPAI:** 0.29
RecName: Full=Trypsin; EC=3.4.21.4; Flags: Precursor; - OS=Sus scrofa (Pig).
☐ Check to include this hit in error tolerant search or archive report

	Query	Observed	Mr(expt)	Mr(calc)	ppm	Miss	Score	Expect	Rank	Unique	Retention Time	Peptide
☑	46	421.7557	841.4969	841.5022	-6.28	0	(50)	0.0031	1	U	839s (13.98m)	R.VATVSLPR.S
☑	47	421.7557	841.4969	841.5022	-6.21	0	(59)	0.00037	1	U	732s (12.20m)	R.VATVSLPR.S
☑	48	421.7561	841.4977	841.5022	-5.26	0	(50)	0.0031	1	U	759s (12.65m)	R.VATVSLPR.S
☑	49	421.7564	841.4983	841.5022	-4.59	0	(55)	0.00095	1	U	633s (10.55m)	R.VATVSLPR.S
☑	50	421.7564	841.4983	841.5022	-4.59	0	(53)	0.0014	1	U	630s (10.50m)	R.VATVSLPR.S
☑	51	421.7565	841.4984	841.5022	-4.45	0	(57)	0.00067	1	U	538s (8.97m)	R.VATVSLPR.S
☑	52	421.7565	841.4984	841.5022	-4.45	0	60	0.0003	1	U	542s (9.03m)	R.VATVSLPR.S
☑	128	523.2821	1044.5496	1044.5564	-6.49	0	(71)	3.7e-05	1	U	509s (8.48m)	K.LSSPATLNSR.V
☑	129	523.2821	1044.5496	1044.5564	-6.49	0	86	1.1e-06	1	U	517s (8.62m)	K.LSSPATLNSR.V

---

20. CALMA_ARBPU Mass: 16037 Score: 145 Matches: 4(2) Sequences: 2(1) **emPAI:** 0.21
RecName: Full=Calmodulin-alpha; Short=CaM A; Flags: Fragment; - OS=Arbacia punctulata (Punctuate sea urchin).
☐ Check to include this hit in error tolerant search or archive report

	Query	Observed	Mr(expt)	Mr(calc)	ppm	Miss	Score	Expect	Rank	Unique	Retention Time	Peptide
☑	36	403.2152	804.4158	804.4164	-0.67	0	(24)	1.5	1	U	545s (9.08m)	K.ELGTVMR.S
☑	37	403.2155	804.4164	804.4164	0.05	0	37	0.079	1	U	542s (9.03m)	K.ELGTVMR.S
☑	314	615.6337	1843.8792	1843.8840	-2.59	1	108	3.8e-09	1	U	737s (12.28m)	K.EAFSLFDKDGDGTITTK.E
☑	315	615.6337	1843.8792	1843.8840	-2.59	1	(86)	7e-07	1	U	734s (12.23m)	K.EAFSLFDKDGDGTITTK.E

Proteins matching the same set of peptides:
CALMA_HALRO Mass: 16800 Score: 145 Matches: 4(2) Sequences: 2(1)
RecName: Full=Calmodulin-A; Short=CaM A; - OS=Halocynthia roretzi (Sea squirt).
CALMB_ARBPU Mass: 15554 Score: 145 Matches: 4(2) Sequences: 2(1)
RecName: Full=Calmodulin-beta; Short=Cam B; Flags: Fragment; - OS=Arbacia punctulata (Punctuate sea urchin).
CALMB_HALRO Mass: 16830 Score: 145 Matches: 4(2) Sequences: 2(1)
RecName: Full=Calmodulin-B; Short=CaM B; - OS=Halocynthia roretzi (Sea squirt).
CALM_AGABI Mass: 16773 Score: 145 Matches: 4(2) Sequences: 2(1)
RecName: Full=Calmodulin; Short=CaM; - OS=Agaricus bisporus (Common mushroom).
CALM_ALEFU Mass: 16780 Score: 145 Matches: 4(2) Sequences: 2(1)
RecName: Full=Calmodulin; Short=CaM; - OS=Alexandrium fundyense (Dinoflagellate).
CALM_ANAPL Mass: 16827 Score: 145 Matches: 4(2) Sequences: 2(1)
RecName: Full=Calmodulin; Short=CaM; - OS=Anas platyrhynchos (Domestic duck) (Anas boschas).
CALM_APLCA Mass: 16800 Score: 145 Matches: 4(2) Sequences: 2(1)
RecName: Full=Calmodulin; Short=CaM; - OS=Aplysia californica (California sea hare).
CALM_BLAEM Mass: 16772 Score: 145 Matches: 4(2) Sequences: 2(1)
RecName: Full=Calmodulin; Short=CaM; - OS=Blastocladiella emersonii (Aquatic fungus).
CALM_BOVIN Mass: 16827 Score: 145 Matches: 4(2) Sequences: 2(1)
RecName: Full=Calmodulin; Short=CaM; - OS=Bos taurus (Bovine).

CALM_CAEEL **Mass:** 16814 **Score:** 145 **Matches:** 4(2) **Sequences:** 2(1)
RecName: Full=Calmodulin; Short=CaM; - OS=Caenorhabditis elegans.
CALM_CHICK **Mass:** 16827 **Score:** 145 **Matches:** 4(2) **Sequences:** 2(1)
RecName: Full=Calmodulin; Short=CaM; - OS=Gallus gallus (Chicken).
CALM_CIOIN **Mass:** 16826 **Score:** 145 **Matches:** 4(2) **Sequences:** 2(1)
RecName: Full=Calmodulin; Short=CaM; AltName: Full=Ci-CaM; - OS=Ciona intestinalis (Transparent sea squirt) (Ascidia intestinalis).
CALM_CTEID **Mass:** 16827 **Score:** 145 **Matches:** 4(2) **Sequences:** 2(1)
RecName: Full=Calmodulin; Short=CaM; - OS=Ctenopharyngodon idella (Grass carp) (Leuciscus idella).
CALM_DANRE **Mass:** 16827 **Score:** 145 **Matches:** 4(2) **Sequences:** 2(1)
RecName: Full=Calmodulin; Short=CaM; - OS=Danio rerio (Zebrafish) (Brachydanio rerio).
CALM_DROME **Mass:** 16800 **Score:** 145 **Matches:** 4(2) **Sequences:** 2(1)
RecName: Full=Calmodulin; Short=CaM; - OS=Drosophila melanogaster (Fruit fly).
CALM_ELEEL **Mass:** 16799 **Score:** 145 **Matches:** 4(2) **Sequences:** 2(1)
RecName: Full=Calmodulin; Short=CaM; - OS=Electrophorus electricus (Electric eel).
CALM_EPIAK **Mass:** 16809 **Score:** 145 **Matches:** 4(2) **Sequences:** 2(1)
RecName: Full=Calmodulin; Short=CaM; - OS=Epinephelus akaara (Hong Kong grouper) (Serranus akaara).
CALM_EUGGR **Mass:** 16845 **Score:** 145 **Matches:** 4(2) **Sequences:** 2(1)
RecName: Full=Calmodulin; Short=CaM; - OS=Euglena gracilis.
CALM_GECJA **Mass:** 16827 **Score:** 145 **Matches:** 4(2) **Sequences:** 2(1)
RecName: Full=Calmodulin; Short=CaM; - OS=Gecko japonicus (Japanese gecko).
CALM_HALOK **Mass:** 16743 **Score:** 145 **Matches:** 4(2) **Sequences:** 2(1)
RecName: Full=Calmodulin; Short=CaM; - OS=Halichondria okadai (Marine sponge) (Reniera okadai).
CALM_HETTR **Mass:** 16796 **Score:** 145 **Matches:** 4(2) **Sequences:** 2(1)
RecName: Full=Calmodulin; Short=CaM; - OS=Heterocapsa triquetra (Dinoflagellate) (Glenodinium triquetrum).
CALM_HUMAN **Mass:** 16827 **Score:** 145 **Matches:** 4(2) **Sequences:** 2(1)
RecName: Full=Calmodulin; Short=CaM; - OS=Homo sapiens (Human).
CALM_KARMI **Mass:** 16780 **Score:** 145 **Matches:** 4(2) **Sequences:** 2(1)
RecName: Full=Calmodulin; Short=CaM; - OS=Karlodinium micrum (Dinoflagellate).
CALM_LOCMI **Mass:** 16800 **Score:** 145 **Matches:** 4(2) **Sequences:** 2(1)
RecName: Full=Calmodulin; Short=CaM; - OS=Locusta migratoria (Migratory locust).
CALM_LUMRU **Mass:** 16830 **Score:** 145 **Matches:** 4(2) **Sequences:** 2(1)
RecName: Full=Calmodulin; Short=CaM; - OS=Lumbricus rubellus (Humus earthworm).
CALM_MACPY **Mass:** 16798 **Score:** 145 **Matches:** 4(2) **Sequences:** 2(1)
RecName: Full=Calmodulin; Short=CaM; - OS=Macrocystis pyrifera (Giant kelp).
CALM_METSE **Mass:** 16827 **Score:** 145 **Matches:** 4(2) **Sequences:** 2(1)
RecName: Full=Calmodulin; Short=CaM; - OS=Metridium senile (Brown sea anemone) (Frilled sea anemone).
CALM_MOUSE **Mass:** 16827 **Score:** 145 **Matches:** 4(2) **Sequences:** 2(1)
RecName: Full=Calmodulin; Short=CaM; - OS=Mus musculus (Mouse).
CALM_MYXGL **Mass:** 16826 **Score:** 145 **Matches:** 4(2) **Sequences:** 2(1)
RecName: Full=Calmodulin; Short=CaM; - OS=Myxine glutinosa (Atlantic hagfish).
CALM_ONCSP **Mass:** 16827 **Score:** 145 **Matches:** 4(2) **Sequences:** 2(1)
RecName: Full=Calmodulin; Short=CaM; - OS=Oncorhynchus sp. (Salmon).
CALM_OREMO **Mass:** 16835 **Score:** 145 **Matches:** 4(2) **Sequences:** 2(1)
RecName: Full=Calmodulin; Short=CaM; - OS=Oreochromis mossambicus (Mozambique tilapia) (Tilapia mossambica).
CALM_ORYLA **Mass:** 15348 **Score:** 145 **Matches:** 4(2) **Sequences:** 2(1)
RecName: Full=Calmodulin-A; Short=CaM A; Flags: Fragment; - OS=Oryzias latipes (Medaka fish) (Japanese ricefish).
CALM_PATSP **Mass:** 16802 **Score:** 145 **Matches:** 4(2) **Sequences:** 2(1)
RecName: Full=Calmodulin; Short=CaM; - OS=Patinopecten sp. (Scallop).
CALM_PAXIN **Mass:** 16773 **Score:** 145 **Matches:** 4(2) **Sequences:** 2(1)
RecName: Full=Calmodulin; Short=CaM; - OS=Paxillus involutus (Naked brimcap).
CALM_PERFV **Mass:** 16827 **Score:** 145 **Matches:** 4(2) **Sequences:** 2(1)
RecName: Full=Calmodulin; Short=CaM; - OS=Perca flavescens (Yellow perch) (Morone flavescens).
CALM_PFIPI **Mass:** 16780 **Score:** 145 **Matches:** 4(2) **Sequences:** 2(1)
RecName: Full=Calmodulin; Short=CaM; - OS=Pfiesteria piscicida (Dinoflagellate).
CALM_PHYIN **Mass:** 16814 **Score:** 145 **Matches:** 4(2) **Sequences:** 2(1)
RecName: Full=Calmodulin; Short=CaM; - OS=Phytophthora infestans (Potato late blight fungus).
CALM_PLAFA **Mass:** 16920 **Score:** 145 **Matches:** 4(2) **Sequences:** 2(1)
RecName: Full=Calmodulin; Short=CaM; - OS=Plasmodium falciparum.
CALM_PLECO **Mass:** 16828 **Score:** 145 **Matches:** 4(2) **Sequences:** 2(1)
RecName: Full=Calmodulin; Short=CaM; - OS=Pleurotus cornucopiae (Cornucopia mushroom).
CALM_PLEOS **Mass:** 16814 **Score:** 145 **Matches:** 4(2) **Sequences:** 2(1)
RecName: Full=Calmodulin; Short=CaM; - OS=Pleurotus ostreatus (Oyster mushroom) (White-rot fungus).
CALM_PONAB **Mass:** 16827 **Score:** 145 **Matches:** 4(2) **Sequences:** 2(1)
RecName: Full=Calmodulin; Short=CaM; - OS=Pongo abelii (Sumatran orangutan) (Pongo pygmaeus abelii).
CALM_PROMN **Mass:** 16780 **Score:** 145 **Matches:** 4(2) **Sequences:** 2(1)
RecName: Full=Calmodulin; Short=CaM; - OS=Prorocentrum minimum (Dinoflagellate).
CALM_PYTSP **Mass:** 16814 **Score:** 145 **Matches:** 4(2) **Sequences:** 2(1)
RecName: Full=Calmodulin; Short=CaM; - OS=Pythium splendens (Leaf rot fungus).
CALM_PYUSP **Mass:** 16801 **Score:** 145 **Matches:** 4(2) **Sequences:** 2(1)
RecName: Full=Calmodulin; Short=CaM; - OS=Pyuridae sp. (Sea squirt).
CALM_RABIT **Mass:** 16827 **Score:** 145 **Matches:** 4(2) **Sequences:** 2(1)
RecName: Full=Calmodulin; Short=CaM; - OS=Oryctolagus cuniculus (Rabbit).
CALM_RAT **Mass:** 16827 **Score:** 145 **Matches:** 4(2) **Sequences:** 2(1)
RecName: Full=Calmodulin; Short=CaM; - OS=Rattus norvegicus (Rat).
CALM_RENRE **Mass:** 16829 **Score:** 145 **Matches:** 4(2) **Sequences:** 2(1)
RecName: Full=Calmodulin; Short=CaM; - OS=Renilla reniformis (Sea pansy).
CALM_SACJA **Mass:** 16757 **Score:** 145 **Matches:** 4(2) **Sequences:** 2(1)
RecName: Full=Calmodulin; Short=CaM; - OS=Saccharina japonica (Sweet kelp) (Laminaria japonica).
CALM_SHEEP **Mass:** 16827 **Score:** 145 **Matches:** 4(2) **Sequences:** 2(1)
RecName: Full=Calmodulin; Short=CaM; - OS=Ovis aries (Sheep).
CALM_STIJA **Mass:** 16816 **Score:** 145 **Matches:** 4(2) **Sequences:** 2(1)
RecName: Full=Calmodulin; Short=CaM; - OS=Stichopus japonicus (Sea cucumber).
CALM_STRIE **Mass:** 17585 **Score:** 145 **Matches:** 4(2) **Sequences:** 2(1)
RecName: Full=Calmodulin; Short=CaM; - OS=Strongylocentrotus intermedius (Sea urchin).

CALM_STYLE **Mass:** 16755 **Score:** 145 **Matches:** 4(2) **Sequences:** 2(1)
RecName: Full=Calmodulin; Short=CaM; - OS=Stylonychia lemnae.
CALM_SUBDO **Mass:** 16788 **Score:** 145 **Matches:** 4(2) **Sequences:** 2(1)
RecName: Full=Calmodulin; Short=CaM; - OS=Suberites domuncula (Sponge).
CALM_TETPY **Mass:** 16797 **Score:** 145 **Matches:** 4(2) **Sequences:** 2(1)
RecName: Full=Calmodulin; Short=CaM; - OS=Tetrahymena pyriformis.
CALM_TORCA **Mass:** 16827 **Score:** 145 **Matches:** 4(2) **Sequences:** 2(1)
RecName: Full=Calmodulin; Short=CaM; - OS=Torpedo californica (Pacific electric ray).
CALM_TRYBB **Mass:** 16828 **Score:** 145 **Matches:** 4(2) **Sequences:** 2(1)
RecName: Full=Calmodulin; Short=CaM; - OS=Trypanosoma brucei brucei.
CALM_TRYBG **Mass:** 16828 **Score:** 145 **Matches:** 4(2) **Sequences:** 2(1)
RecName: Full=Calmodulin; Short=CaM; - OS=Trypanosoma brucei gambiense.
CALM_TRYCR **Mass:** 16814 **Score:** 145 **Matches:** 4(2) **Sequences:** 2(1)
RecName: Full=Calmodulin; Short=CaM; - OS=Trypanosoma cruzi.
CALM_XENLA **Mass:** 16827 **Score:** 145 **Matches:** 4(2) **Sequences:** 2(1)
RecName: Full=Calmodulin; Short=CaM; - OS=Xenopus laevis (African clawed frog).
CALN_CHICK **Mass:** 14838 **Score:** 145 **Matches:** 4(2) **Sequences:** 2(1)
RecName: Full=Neo-calmodulin; Short=NeoCaM; Flags: Fragment; - OS=Gallus gallus (Chicken).
CALM1_BRAFL **Mass:** 16800 **Score:** 145 **Matches:** 4(2) **Sequences:** 2(1)
RecName: Full=Calmodulin-1; Short=CaM 1; - OS=Branchiostoma floridae (Florida lancelet) (Amphioxus).
CALM1_BRALA **Mass:** 16800 **Score:** 145 **Matches:** 4(2) **Sequences:** 2(1)
RecName: Full=Calmodulin-1; Short=CaM 1; - OS=Branchiostoma lanceolatum (Common lancelet) (Amphioxus).
CALM2_BRALA **Mass:** 16814 **Score:** 145 **Matches:** 4(2) **Sequences:** 2(1)
RecName: Full=Calmodulin-2; Short=CaM 2; - OS=Branchiostoma lanceolatum (Common lancelet) (Amphioxus).
CALM_PLAF7 **Mass:** 16920 **Score:** 145 **Matches:** 4(2) **Sequences:** 2(1)
RecName: Full=Calmodulin; Short=CaM; - OS=Plasmodium falciparum (isolate 3D7).

Peptide matches not assigned to protein hits: (no details means no match)

Query	Observed	Mr(expt)	Mr(calc)	ppm	Miss	Score	Expect	Rank	Unique	Retention Time	Peptide
247	737.9195	1473.8245	1473.8304	-3.99	0	81	2.4e-06	1		657s (10.95m)	TPIVGQPSIPGGPVR
38	807.4004	1612.7862	1612.7944	-5.08	0	79	1.1e-05	1		883s (14.72m)	ETEVIDPQDLLEGR
278	807.4016	1612.7886	1612.7944	-3.60	0	78	4.7e-06	1		880s (14.67m)	ETEVIDPQDLLEGR
229	690.3554	1378.6962	1378.6980	-1.32	0	74	1.4e-05	1		810s (13.50m)	GLAFTDVDVDSIK
219	666.8501	1331.6856	1331.6932	-5.71	0	74	1.9e-05	1		585s (9.75m)	GILAADESTGSIAK
277	806.4017	1610.7889	1610.8039	-9.31	0	72	1.9e-05	1		700s (11.67m)	TVEIPDPVEAGEEVK
246	737.9195	1473.8245	1473.8304	-3.99	0	70	3.1e-05	1		660s (11.00m)	TPIVGQPSIPGGPVR
215	663.3738	1324.7330	1324.7463	-10.02	0	66	6.2e-05	1		550s (9.17m)	DNIQGITKPAIR
184	610.8041	1219.5937	1219.5986	-4.02	0	64	0.00012	1		762s (12.70m)	NFPSPVDAAFR
19	655.8523	1309.6900	1309.6952	-3.91	0	63	0.00015	1		954s (15.90m)	TVTAMDVVYALK
150	571.3007	1140.5869	1140.6026	-13.77	0	63	0.00013	1		686s (11.43m)	GDLGIEIPAEK
134	542.8174	1083.6202	1083.6288	-7.86	0	62	0.00021	1		951s (15.85m)	ALAAVLEELR
171	593.8227	1185.6308	1185.6394	-7.26	0	62	0.00028	1		727s (12.12m)	GPSVFPLAPSSK
20	687.8283	1373.6420	1373.6422	-0.17	0	61	0.00014	1		531s (8.85m)	VESLEQEAANER
218	666.8501	1331.6856	1331.6932	-5.71	0	61	0.00039	1		589s (9.82m)	GILAADESTGSIAK
91	472.7677	943.5209	943.5240	-3.23	0	61	0.00046	1		692s (11.53m)	AGIQFPVGR
93	474.2357	946.4569	946.4542	2.89	0	60	0.0003	1		529s (8.82m)	VEAMLNDR
92	473.2513	944.4881	944.4927	-4.85	0	59	0.00057	1		555s (9.25m)	ELVLDNSR
156	577.8087	1153.6028	1153.6091	-5.52	0	59	0.00039	1		507s (8.45m)	HTLNQIDSVK
90	472.7674	943.5203	943.5240	-3.91	0	56	0.0013	1		696s (11.60m)	AGIQFPVGR
313	920.9019	1839.7893	1839.7992	-5.36	0	55	0.00015	1		985s (16.42m)	IDFYFDENPYFENK
182	604.8043	1207.5941	1207.5972	-2.60	0	55	0.00097	1		673s (11.22m)	VEVTEFEDIK
285	826.4101	1650.8056	1650.8100	-2.69	0	55	0.001	1		803s (13.38m)	SQEQLAAELAEYTAK
183	610.8041	1219.5937	1219.5986	-4.02	0	53	0.0015	1		759s (12.65m)	NFPSPVDAAFR
139	552.7948	1103.5751	1103.5764	-1.18	0	53	0.002	1		1002s (16.70m)	IGFPWSEIR
84	920.9022	1839.7899	1839.7992	-5.03	0	53	0.00032	1		982s (16.37m)	IDFYFDENPYFENK
29	772.3813	1542.7480	1542.7566	-5.55	0	52	0.0044	1		647s (10.78m)	SYTITGLQPGTDYK
267	778.9286	1555.8427	1555.8497	-4.54	0	51	0.0034	1		1055s (17.58m)	LDQLIYIPLPDEK
172	593.8227	1185.6308	1185.6394	-7.26	0	50	0.0041	1		729s (12.15m)	GPSVFPLAPSSK
21	717.8631	1433.7116	1433.7191	-5.17	0	49	0.0044	1		612s (10.20m)	PYQYPALTPEQK
239	479.2459	1434.7157	1434.7256	-6.85	0	44	0.015	1		520s (8.67m)	HYQHVLAVDPEK
177	599.8338	1197.6530	1197.6605	-6.21	1	39	0.04	1		527s (8.78m)	KLEENEVIPK
178	599.8338	1197.6530	1197.6605	-6.21	1	37	0.058	1		524s (8.73m)	KLEENEVIPK
130	523.7763	1045.5381	1045.5404	-2.17	1	37	0.093	1		513s (8.55m)	LREGTLDDK
85	465.7558	929.4970	929.5004	-3.66	0	33	0.21	1		608s (10.13m)	VPVLMESR
65	430.7304	859.4462	859.4552	-10.46	0	32	0.24	1		607s (10.12m)	WPTTLSR
159	581.3086	1160.6026	1160.5925	8.74	0	30	0.53	1		605s (10.08m)	EDGLIITGESK
64	430.2187	858.4229	858.4276	-5.49	0	29	0.28	1		685s (11.42m)	GFFYTPK
240	723.8399	1445.6652	1445.6820	-11.64	0	28	0.25	1		566s (9.43m)	EVDEQMINVQNK
166	588.8127	1175.6109	1175.6034	6.41	0	28	0.71	1		790s (13.17m)	TIDETLETVR
35	401.2612	800.5079	800.5007	8.92	0	28	0.32	1		655s (10.92m)	DVILITK
56	425.7630	849.5114	849.5184	-8.29	0	28	0.27	1		584s (9.73m)	HIQLAIR
116	509.2710	1016.5275	1016.5331	-5.51	0	27	0.93	1		649s (10.82m)	VWVYPPEK
238	717.8643	1433.7140	1433.7191	-3.55	0	26	0.87	1		614s (10.23m)	PYQYPALTPEQK
132	539.7888	1077.5629	1077.5488	13.1	0	26	1.1	1		570s (9.50m)	MATTLATDVR
153	576.2793	1150.5441	1150.5546	-9.11	0	25	0.72	1		640s (10.67m)	ESYSIYVYK
99	478.2968	954.5791	954.5862	-7.48	0	25	0.62	1		657s (10.95m)	LAVTVPTVR
152	574.8130	1147.6114	1147.6085	2.57	1	25	1.4	1		495s (8.25m)	EAVTGSVEKTK
71	435.7703	869.5261	869.5334	-8.46	0	25	0.57	1		597s (9.95m)	VVSLSIPR
72	435.7703	869.5261	869.5334	-8.46	0	25	0.58	1		594s (9.90m)	VVSLSIPR
43	412.7511	823.4876	823.5028	-18.48	0	24	0.4	1		572s (9.53m)	RPVSLPR
7	463.7999	925.5853	925.5709	15.6	2	24	0.35	1		625s (10.42m)	RKVLADPK
16	618.7934	1235.5723	1235.5638	6.85	1	24	0.72	1		546s (9.10m)	DKGLSPAMMDR + Oxidation (M)
108	500.7533	999.4920	999.4913	0.76	0	24	0.77	1		581s (9.68m)	YLGEEYVK
57	425.7631	849.5117	849.5184	-7.89	0	24	0.66	1		588s (9.80m)	HIQLAIR
154	576.2807	1150.5468	1150.5288	15.7	0	24	1.1	1		510s (8.50m)	QSQMDQISSK

☑	44	412.7511	823.4876	823.5028	-18.48	0	23	0.57	1	574s (9.57m)	RPVSLPR
☑	26	383.1999	764.3853	764.3857	-0.57	0	22	2.2	1	659s (10.98m)	GEFVWK
☑	13	552.2752	1102.5359	1102.5255	9.45	0	22	3.6	1	537s (8.95m)	LDDLEQNTR
☑	202	639.3316	1276.6487	1276.6485	0.13	0	22	2.9	1	730s (12.17m)	LTPMEFGNLQK
☑	103	486.7646	971.5146	971.5148	-0.21	1	21	2.7	1	530s (8.83m)	NLRGDIER
☑	70	435.7540	869.4935	869.4971	-4.12	0	21	1.4	1	715s (11.92m)	VVTDLAPR
☑	25	383.1999	764.3853	764.3857	-0.57	0	21	2.6	1	662s (11.03m)	GEFVWK
☑	30	394.7334	787.4522	787.4552	-3.77	0	21	5.2	1	592s (9.87m)	GSAVVSLR
☑	147	567.8265	1133.6384	1133.6193	16.8	0	21	2.7	1	809s (13.48m)	IVDILHDPGR
☑	214	442.5639	1324.6700	1324.6921	-16.72	1	21	3.1	1	492s (8.20m)	DGRVHILGEMAK
☑	34	401.2612	800.5079	800.5007	8.92	0	21	1.7	1	654s (10.90m)	LVDITLK
☑	33	399.2263	796.4381	796.4378	0.39	0	21	1.4	1	522s (8.70m)	IHIMAGR
☑	12	551.8016	1101.5886	1101.6070	-16.74	0	20	4.7	1	1068s (17.80m)	TFDLPATIPK
☑	189	622.3470	1242.6795	1242.7044	-20.00	2	20	4.4	1	806s (13.43m)	GELLSRVRGEK
☑	135	551.8012	1101.5877	1101.5931	-4.85	1	19	5.8	1	1071s (17.85m)	WGVTIQDRK
☑	146	567.8265	1133.6384	1133.6193	16.8	0	19	4	1	812s (13.53m)	IVDILHDPGR
☑	294	585.9548	1754.8426	1754.8509	-4.71	1	19	3.6	1	647s (10.78m)	MSQIETKEFQTEIR + Oxidation (M)
☑	248	750.8399	1499.6652	1499.6656	-0.25	0	19	1.3	1	560s (9.33m)	WCDDYFFIVHR
☑	191	622.3474	1242.6803	1242.7044	-19.42	2	19	6.3	1	710s (11.83m)	GELLSRVRGEK
☑	141	557.7883	1113.5620	1113.5818	-17.78	1	18	4.4	1	520s (8.67m)	SFYKVEVSR
☑	117	509.2882	1016.5618	1016.5655	-3.57	0	18	8.1	1	637s (10.62m)	VAPIQGFSAK
☑	5	451.2615	1350.7628	1350.7693	-4.86	2	18	14	1	539s (8.98m)	KTTMLFVIRDK
☑	196	625.8014	1249.5882	1249.6125	-19.39	1	17	4.2	1	544s (9.07m)	QEYEKLGPMR
☑	4	447.2334	892.4522	892.4555	-3.68	0	17	14	1	500s (8.33m)	HPTWPQK
☑	63	430.2187	858.4229	858.4276	-5.49	0	17	4.5	1	690s (11.50m)	GFFYTPK
☑	69	435.7540	869.4935	869.4971	-4.10	0	17	3.9	1	718s (11.97m)	VVEALSPR
☑	8	463.8025	925.5903	925.5961	-6.16	0	16	1.8	1	856s (14.27m)	LLVAVAGGVK
☑	237	475.9199	1424.7379	1424.7273	7.48	1	16	9.2	1	545s (9.08m)	AFNRSSHITQHK
☑	124	521.3053	1040.5960	1040.5978	-1.75	0	15	8.4	1	638s (10.63m)	IVLQIDNAR
☑	112	502.2900	1002.5655	1002.5570	8.42	1	15	13	1	578s (9.63m)	TLAVSRNSR
☑	78	451.2634	900.5122	900.5280	-17.60	0	14	22	1	543s (9.05m)	VINLIDSK
☑	305	897.9065	1793.7984	1793.8028	-2.44	0	14	3.8	1	795s (13.25m)	TGTTGQSGAESGTTEPSAR
☑	201	639.3017	1276.5888	1276.5791	7.54	0	14	6.1	1	809s (13.48m)	INGMPEVQDMK + Oxidation (M)
☑	11	495.2585	1482.7536	1482.7687	-10.16	1	14	30	1	756s (12.60m)	LIGRMFDSLMLR + 2 Oxidation (M)
☑	181	603.8185	1205.6225	1205.6193	2.67	0	14	18	1	766s (12.77m)	WSIPYSQIGR
☑	197	626.8118	1251.6091	1251.6281	-15.24	1	14	12	1	980s (16.33m)	VAKIMEGFGER + Oxidation (M)
☑	210	655.3194	1308.6242	1308.6197	3.42	0	14	11	1	635s (10.58m)	SGYTAAIDAPESK
☑	165	588.8127	1175.6109	1175.6186	-6.56	0	14	19	1	793s (13.22m)	TLNYINPDVK
☑	113	502.2908	1002.5671	1002.5532	13.9	1	13	17	1	595s (9.92m)	KMVIAEANK
☑	334	747.0460	2238.1162	2238.1368	-9.19	0	13	16	1	763s (12.72m)	VVGIADINCWYNHPIINAAR
☑	220	667.3647	1332.7149	1332.7071	5.82	0	13	20	1	1003s (16.72m)	TLDLAMANSLLR + Oxidation (M)
☑	138	552.2826	1102.5506	1102.5692	-16.89	0	13	15	1	540s (9.00m)	EVDALGGIMAK
☑	252	754.8697	1507.7249	1507.7194	3.62	0	13	15	1	626s (10.43m)	EIFQESWEAELK
☑	190	622.3474	1242.6803	1242.6819	-1.35	1	13	23	1	714s (11.90m)	KDVLLEDNVAK
☑	221	667.3647	1332.7149	1332.7071	5.82	0	13	22	1	1006s (16.77m)	TLDLAMANSLLR + Oxidation (M)
☑	86	466.2338	930.4531	930.4407	13.4	0	13	12	1	561s (9.35m)	VDQAEDVR
☑	151	381.8760	1142.6062	1142.6084	-1.96	0	13	19	1	646s (10.77m)	NAHDVVFLTK
☑	140	557.3345	1112.6544	1112.6376	15.1	1	13	15	1	639s (10.65m)	VAEAMLKVPR
☑	300	890.9280	1779.8414	1779.8614	-11.20	2	13	14	1	771s (12.85m)	RRYEDAIPFEMPEK
☑	211	655.3194	1308.6242	1308.6197	3.42	0	12	16	1	630s (10.50m)	SGYTAAIDAPESK
☑	133	539.7888	1077.5629	1077.5488	13.1	0	12	30	1	573s (9.55m)	MATTLATDVR
☑	24	382.7144	763.4143	763.4116	3.61	0	12	22	1	584s (9.73m)	IFIEDK
☑	6	457.7659	913.5173	913.5094	8.73	1	12	32	1	607s (10.12m)	VATGRTGPR
☑	9	466.2427	930.4708	930.4845	-14.70	0	11	54	1	867s (14.45m)	VIGMDVPGK + Oxidation (M)
☑	304	897.9065	1793.7984	1793.8028	-2.44	0	11	7.2	1	798s (13.30m)	TGTTGQSGAESGTTEPSAR
☑	180	602.3086	1202.6027	1202.6078	-4.20	0	11	29	1	914s (15.23m)	VMGQAGVLDAAR + Oxidation (M)
☑	188	617.2650	1232.5154	1232.5206	-4.14	0	11	3.2	1	971s (16.18m)	YALPYDMCNK + Oxidation (M)
☑	170	593.3326	1184.6506	1184.6626	-10.12	1	11	30	1	620s (10.33m)	RGQTSASILPR
☑	187	617.2650	1232.5154	1232.5343	-15.31	0	11	3.3	1	974s (16.23m)	FGSMSTATSSNK + Oxidation (M)
☑	54	425.7586	849.5026	849.4960	7.86	1	11	16	1	480s (8.00m)	KIIEYGK
☑	169	592.8638	1183.7130	1183.7289	-13.35	1	10	22	1	676s (11.27m)	LIQRIDVTVK
☑	53	849.4425	848.4353	848.4352	0.09	1	10	1.1e+02	1	684s (11.40m)	GSSKLSDR
☑	306	901.9618	1801.9091	1801.8855	13.1	0	10	37	1	807s (13.45m)	QVDQPMHIIIGNMFK + 2 Oxidation (M)
☑	268	782.3760	1562.7374	1562.7399	-1.60	0	10	24	1	817s (13.62m)	DQLLNQVINEFCQ
☑	213	659.3776	1316.7406	1316.7639	-17.65	0	10	32	1	1151s (19.18m)	CILVNIFGGILR
☑	2	420.2237	1257.6494	1257.6313	14.4	1	10	69	1	1486s (24.77m)	GDAAAAAQNVDKK
☑	330	700.3572	2098.0497	2098.0303	9.24	2	9	41	1	847s (14.12m)	ENAEYHAARERQGIIEGR
☑	243	733.8434	1465.6722	1465.6725	-0.24	0	9	19	1	550s (9.17m)	EVQEWVFSEGEK
☑	250	752.8638	1503.7131	1503.6913	14.5	1	9	28	1	836s (13.93m)	GERVTEQSAEDQR
☑	280	813.4400	1624.8655	1624.8970	-19.44	1	9	39	1	1132s (18.87m)	DILLGTEITHKVMR
☑	110	501.2837	1000.5529	1000.5488	4.14	0	9	67	1	712s (11.87m)	MVLAGQVQR
☑	55	425.7586	849.5026	849.4960	7.82	0	9	25	1	483s (8.05m)	QTLIFTK
☑	244	733.8434	1465.6722	1465.6759	-2.53	1	9	22	1	553s (9.22m)	KMEPAEDFEQVK + Oxidation (M)
☑	301	890.9280	1779.8414	1779.8751	-18.94	2	9	35	1	767s (12.78m)	RFSDVDEDISRQSVK
☑	40	813.4407	1624.8668	1624.8355	19.2	1	9	1.1e+02	1	1128s (18.80m)	MAVHRGSALVAPASDK + Oxidation (M)
☑	298	881.9203	1761.8259	1761.8355	-5.44	1	9	32	1	754s (12.57m)	ELARYSGLTEAMSYR + Oxidation (M)
☑	249	752.8638	1503.7131	1503.7351	-14.64	2	9	33	1	844s (14.07m)	DVKRQDLEQMDK
☑	279	812.4020	1622.7895	1622.7796	6.09	0	8	49	1	968s (16.13m)	SWLEDMTLNMIVR + Oxidation (M)
☑	115	507.2755	1012.5364	1012.5553	-18.65	0	8	35	1	608s (10.13m)	QTVGPDIGVK
☑	292	849.4384	1696.8622	1696.8321	17.7	0	8	55	1	681s (11.35m)	APHPYGVPAPSAGAYSR
☑	160	582.2823	1162.5500	1162.5553	-4.60	2	8	38	1	782s (13.03m)	TKAERSGHMF
☑	194	624.8483	1247.6821	1247.6874	-4.19	1	8	60	1	846s (14.10m)	NLDNLKGTFVK
☑	88	472.2856	942.5566	942.5386	19.2	0	8	72	1	949s (15.82m)	TLDILPSGK
☑	287	558.2756	1671.8051	1671.8315	-15.82	0	8	42	1	533s (8.88m)	NGDGVVDISELQEGLK
☑	127	522.7778	1043.5410	1043.5247	15.6	0	8	62	1	644s (10.73m)	TPSPAISESR
☑	109	501.2837	1000.5529	1000.5375	15.4	0	8	84	1	709s (11.82m)	MTPLSSPLR

☑	173	595.3274	1188.6403	1188.6251	12.8	0	8	77	1	824s (13.73m)	TAGVTEIHPHK
☑	269	782.3760	1562.7374	1562.7511	-8.77	0	8	42	1	813s (13.55m)	NMIHETNEHTLPK
☑	270	783.8702	1565.7258	1565.7429	-10.94	0	8	35	1	622s (10.37m)	QCTNLEALSTLMDK
☑	174	595.3274	1188.6403	1188.6350	4.44	0	8	80	1	821s (13.68m)	DSGLTQSLLQK
☑	283	819.9103	1637.8060	1637.7784	16.8	0	8	58	1	627s (10.45m)	DTGENLETPSSFTIK
☑	67	432.7473	863.4801	863.4865	-7.36	0	8	58	1	575s (9.58m)	VATLAAYR
☑	333	742.3767	2224.1083	2224.1310	-10.19	2	8	65	1	757s (12.62m)	KLMLDTWNESIFSNIKNR + Oxidation (M)
☑	230	695.8434	1389.6723	1389.6962	-17.21	2	7	54	1	816s (13.60m)	MYTQDKSKFVK + Oxidation (M)
☑	207	646.8091	1291.6037	1291.6264	-17.64	0	7	38	1	565s (9.42m)	TLQVCIMTPDR + Oxidation (M)
☑	122	517.2948	1032.5751	1032.5638	11.0	0	7	64	1	600s (10.00m)	TGLLSAGALCK
☑	14	560.2944	1118.5743	1118.5641	9.11	0	7	2.1e+02	1	857s (14.28m)	LENMENLLK + Oxidation (M)
☑	17	618.7972	1235.5799	1235.5571	18.4	0	7	40	1	549s (9.15m)	NEWDAFALDR
☑	297	881.9203	1761.8259	1761.8434	-9.92	1	7	43	1	758s (12.63m)	YVAFSASFRSEAGSAGR
☑	107	495.2611	988.5076	988.4978	9.94	1	7	84	1	753s (12.55m)	GFPKDNPSK
☑	23	750.3379	1498.6612	1498.6717	-7.04	0	7	66	1	563s (9.38m)	EDMVMVLMDGSLK + 2 Oxidation (M)
☑	15	561.2998	560.2926	560.2918	1.33	0	7	2.4e+02	1	670s (11.17m)	ASVER
☑	121	517.2948	1032.5751	1032.5716	3.37	0	7	76	1	598s (9.97m)	YNGGALLGLR
☑	233	706.3184	1410.6221	1410.6409	-13.30	1	6	24	1	694s (11.57m)	TRTETTTDAQGCK
☑	337	894.1343	2679.3812	2679.3789	0.85	1	6	74	1	1849s (30.82m)	LAQNAMGFGTAITAASEKESIELISK
☑	231	695.8434	1389.6723	1389.6922	-14.29	1	6	69	1	813s (13.55m)	KAEQQAAEQMLK + Oxidation (M)
☑	104	492.7453	983.4760	983.4937	-18.04	1	6	37	1	584s (9.73m)	QSWGGGKHK
☑	251	752.8638	1503.7131	1503.7178	-3.13	0	6	58	1	840s (14.00m)	INDDPHRPNANNK
☑	89	472.2856	942.5566	942.5386	19.1	0	6	1.1e+02	1	946s (15.77m)	TTLPGEVVK
☑	256	507.9220	1520.7442	1520.7439	0.20	2	6	1e+02	1	634s (10.57m)	RKEEMVPSNIMR + 2 Oxidation (M)
☑	105	492.7453	983.4760	983.4937	-18.04	1	6	43	1	581s (9.68m)	QSWGGGKHK
☑	22	747.0558	2238.1456	2238.1685	-10.21	1	6	1e+02	1	766s (12.77m)	AWGFLTDSELQTLTLRYPK
☑	282	544.9704	1631.8893	1631.8804	5.47	1	6	92	1	610s (10.17m)	VKTVVGIEGVEEMVK + Oxidation (M)
☑	28	384.2328	766.4510	766.4450	7.91	0	5	23	1	835s (13.92m)	THQLIR
☑	286	827.4254	1652.8362	1652.8297	3.95	0	5	1e+02	1	978s (16.30m)	IEQELAYAEEIFAK
☑	257	762.8511	1523.6877	1523.6886	-0.59	1	5	41	1	618s (10.30m)	QQDGAMESSQTKAK + Oxidation (M)
☑	254	758.8647	1515.7149	1515.7391	-15.98	2	5	60	1	957s (15.95m)	DKGEKNFAMSYVK
☑	281	544.9704	1631.8893	1631.8631	16.1	1	5	97	1	609s (10.15m)	NVADLEGVVAGYAKAR
☑	193	624.8483	1247.6821	1247.6947	-10.10	0	5	1.1e+02	1	850s (14.17m)	YALGPLLNIMK + Oxidation (M)
☑	1	391.1990	1170.5750	1170.5716	2.90	1	5	1.5e+02	1	613s (10.22m)	GMRRPYGYR + Oxidation (M)
☑	98	478.2522	954.4899	954.4957	-6.11	0	5	60	1	565s (9.42m)	GGPMHTVLK + Oxidation (M)
☑	258	762.8511	1523.6877	1523.6886	-0.59	1	5	43	1	615s (10.25m)	QQDGAMESSQTKAK + Oxidation (M)
☑	3	434.2285	1299.6636	1299.6531	8.06	0	5	1.8e+02	1	584s (9.73m)	AGTTPGSQGRPSGK
☑	125	1042.0010	2081.9875	2082.0211	-16.12	2	5	82	1	827s (13.78m)	SLPDIHSRANNPTSRMMR
☑	192	624.3318	1246.6490	1246.6703	-17.14	2	5	1.5e+02	1	687s (11.45m)	QGAKDMKTQLK
☑	325	647.6486	1939.9241	1939.9165	3.87	1	5	82	1	791s (13.18m)	AGTAGMQNVKAMVDSGMIK + 2 Oxidation (M)
☑	119	512.2384	1022.4623	1022.4781	-15.43	1	5	63	1	539s (8.98m)	DNAQEKYR
☑	157	577.8099	1153.6053	1153.5979	6.47	1	5	1e+02	1	719s (11.98m)	YNEKSVSVTK
☑	296	587.6709	1759.9910	1759.9655	14.5	0	4	71	1	557s (9.28m)	GAPLIGVTAAYGLALAMR + Oxidation (M)
☑	336	630.3116	2517.2172	2517.1693	19.0	0	4	1.2e+02	1	501s (8.35m)	MLENVIDSEYTFAINGSSASTPR + Oxidation (M)
☑	318	927.9506	1853.8867	1853.8573	15.9	1	3	1.2e+02	1	1097s (18.28m)	MISEAIKTNAPTCTVEM + Oxidation (M)
☑	106	494.2987	986.5828	986.5760	6.92	1	3	1.7e+02	1	977s (16.28m)	TEIVKELR
☑	200	639.3017	1276.5888	1276.5969	-6.35	1	3	71	1	812s (13.53m)	KALEEAGGDMEK
☑	259	769.3210	1536.6274	1536.6085	12.3	1	3	9.7	1	871s (14.52m)	EENCKCGWDNPSR
☑	255	758.8647	1515.7149	1515.6987	10.7	0	3	1e+02	1	960s (16.00m)	TGSPSEGAHVSAAMAK + Oxidation (M)
☑	27	384.2328	766.4510	766.4450	7.91	0	3	39	1	834s (13.90m)	THQLIR
☑	319	927.9506	1853.8867	1853.8573	15.9	1	3	1.4e+02	1	1100s (18.33m)	MISEAIKTNAPTCTVEM + Oxidation (M)
☑	290	840.9063	1679.7981	1679.8049	-4.07	1	3	1.3e+02	1	837s (13.95m)	GTASLAGSGRMWSNAAK + Oxidation (M)
☑	324	970.9665	1939.9185	1939.9231	-2.35	0	3	1.2e+02	1	788s (13.13m)	GISNMMLSLTIENSEGTK + Oxidation (M)
☑	204	645.2966	1288.5787	1288.5944	-12.15	0	3	60	1	826s (13.77m)	MSAFSMAPLFR + 2 Oxidation (M)
☑	205	645.7945	1289.5745	1289.5968	-17.31	1	3	49	1	934s (15.57m)	RMSCHALASGNK + Oxidation (M)
☑	97	478.2522	954.4899	954.4771	13.4	0	3	1e+02	1	562s (9.37m)	LDVHTADGK
☑	118	512.2384	1022.4623	1022.4637	-1.38	0	3	96	1	543s (9.05m)	AAQMMGINR + 2 Oxidation (M)
☑	161	582.2823	1162.5500	1162.5540	-3.44	0	3	1.3e+02	1	784s (13.07m)	DANELVEMVK + Oxidation (M)
☑	295	587.6709	1759.9910	1759.9581	18.7	1	3	1.1e+02	1	559s (9.32m)	GAVTQVRVEFLGTAQGK
☑	309	913.9760	1825.9374	1825.9100	15.0	2	3	2.1e+02	1	764s (12.73m)	AMVTETMMKLRNELK + 2 Oxidation (M)
☑	126	522.7778	1043.5410	1043.5247	15.6	0	3	2.1e+02	1	646s (10.77m)	TPSPAISESR
☑	61	429.2117	856.4089	856.4225	-15.90	0	3	71	1	1479s (24.65m)	LHNQAMK + Oxidation (M)
☑	102	970.9770	1939.9395	1939.9422	-1.34	2	2	1.6e+02	1	785s (13.08m)	SFSSAVKNELCRVETDR
☑	60	428.2590	854.5035	854.4909	14.8	1	2	76	1	894s (14.90m)	CVKRPPR
☑	289	840.4054	1678.7963	1678.8270	-18.30	0	2	1.5e+02	1	814s (13.57m)	MTVVSVSMPESLLDR + Oxidation (M)
☑	198	635.8029	1269.5913	1269.5886	2.15	0	2	1e+02	1	511s (8.52m)	FAWGPIMSAMK + 2 Oxidation (M)
☑	241	729.8757	1457.7369	1457.7442	-5.01	0	2	2.4e+02	1	878s (14.63m)	LEQPIIEYYYK
☑	68	865.4061	864.3988	864.4090	-11.76	0	2	3.3e+02	1	586s (9.77m)	AQAYQER
☑	144	564.2744	1126.5342	1126.5506	-14.53	0	2	1.1e+02	1	558s (9.30m)	IDKPEADPDK
☑	265	777.8327	1553.6508	1553.6272	15.2	1	2	26	1	891s (14.85m)	ENCEAAEREMNMK
☑	321	935.4455	1868.8764	1868.8938	-9.29	1	2	1.7e+02	1	854s (14.23m)	GTYNDEVAIMVETQKR + Oxidation (M)
☑	260	769.3212	1536.6278	1536.6085	12.5	1	1	15	1	874s (14.57m)	EENCKCGWDNPSR
☑	322	954.4381	1906.8616	1906.8262	18.6	2	1	1.1e+02	1	628s (10.47m)	KPDDDTRKGCGDDCLNR
☑	100	480.7980	959.5814	959.5698	12.1	2	1	1.4e+02	1	950s (15.83m)	MARIRLGK + Oxidation (M)
☑	284	822.8977	1643.7808	1643.7975	-10.15	2	0	2.2e+02	1	576s (9.60m)	RGQESRLQAEDEAR
☑	101	480.7980	959.5814	959.5950	-14.13	2	0	1.6e+02	1	947s (15.78m)	MAVIKVRK + Oxidation (M)
☑	242	729.8757	1457.7369	1457.7258	7.61	0	0	3.8e+02	1	879s (14.65m)	IPLSEIMDMHIK + 2 Oxidation (M)
☑	45	*421.2529*	*840.4913*							*1707s (28.45m)*	
☑	62	*429.2117*	*856.4089*							*1482s (24.70m)*	
☑	76	*450.2725*	*898.5305*							*922s (15.37m)*	
☑	77	*450.2725*	*898.5305*							*918s (15.30m)*	
☑	82	*458.7861*	*915.5576*							*919s (15.32m)*	
☑	87	*467.7355*	*933.4563*							*545s (9.08m)*	
☑	131	*536.1674*	*1070.3202*							*336s (5.60m)*	
☑	143	*564.2744*	*1126.5342*							*563s (9.38m)*	
☑	199	*635.8029*	*1269.5913*							*508s (8.47m)*	

☑	266	777.8327	1553.6508	887s (14.78m)
☑	271	785.3073	1568.6001	916s (15.27m)
☑	312	918.3833	1834.7520	641s (10.68m)
☑	338	933.0864	2796.2374	881s (14.68m)
☑	339	933.0864	2796.2374	884s (14.73m)
☑	340	843.6397	3370.5297	514s (8.57m)
☑	341	843.6397	3370.5297	511s (8.52m)

Search Parameters

```
Type of search         : MS/MS Ion Search
Enzyme                 : Trypsin
Variable modifications : Oxidation (M)
Mass values            : Monoisotopic
Protein Mass           : Unrestricted
Peptide Mass Tolerance : ± 20 ppm
Fragment Mass Tolerance: ± 0.2 Da
Max Missed Cleavages   : 2
Instrument type        : ESI-QUAD-TOF
Number of queries      : 341
```

Mascot Search Results

```
Host          : 10.10.10.100
User          : alison@anatomy
Email         :
Search title  : CPFP SCH6428 [12/18] 140331_alison_set3_3.mgf
MS data file  : 140331_alison_set3_3.mgf.mascot
Database      : SWISSPROT sprot (538010 sequences; 190998508 residues)
Timestamp     : 2 Apr 2014 at 00:35:10 GMT
Protein hits  : HS90A_BOVIN RecName: Full=Heat shock protein HSP 90-alpha; - OS=Bos taurus (Bovine).
                K2C1_HUMAN  RecName: Full=Keratin, type II cytoskeletal 1; AltName: Full=67 kDa cytokeratin; AltName: Full=Cytokeratin-1; Sh
                HS90B_HORSE RecName: Full=Heat shock protein HSP 90-beta; - OS=Equus caballus (Horse).
                K1C10_HUMAN RecName: Full=Keratin, type I cytoskeletal 10; AltName: Full=Cytokeratin-10; Short=CK-10; AltName: Full=Keratin-
                ALBU_BOVIN  RecName: Full=Serum albumin; AltName: Full=BSA; AltName: Allergen=Bos d 6; Flags: Precursor; - OS=Bos taurus (Bo
                LG3BP_HUMAN RecName: Full=Galectin-3-binding protein; AltName: Full=Basement membrane autoantigen p105; AltName: Full=Lectin
                K1C10_CANFA RecName: Full=Keratin, type I cytoskeletal 10; AltName: Full=Cytokeratin-10; Short=CK-10; AltName: Full=Epitheli
                ACTN4_BOVIN RecName: Full=Alpha-actinin-4; AltName: Full=F-actin cross-linking protein; AltName: Full=Non-muscle alpha-actin
                K1C9_HUMAN  RecName: Full=Keratin, type I cytoskeletal 9; AltName: Full=Cytokeratin-9; Short=CK-9; AltName: Full=Keratin-9;
                CO3_HUMAN   RecName: Full=Complement C3; AltName: Full=C3 and PZP-like alpha-2-macroglobulin domain-containing protein 1; Co
                K22E_HUMAN  RecName: Full=Keratin, type II cytoskeletal 2 epidermal; AltName: Full=Cytokeratin-2e; Short=CK-2e; AltName: Ful
                1433Z_BOVIN RecName: Full=14-3-3 protein zeta/delta; AltName: Full=Factor activating exoenzyme S; Short=FAS; AltName: Full=P
                ACTN4_CHICK RecName: Full=Alpha-actinin-4; AltName: Full=F-actin cross-linking protein; AltName: Full=Non-muscle alpha-actin
                APOA1_BOVIN RecName: Full=Apolipoprotein A-I; Short=Apo-AI; Short=ApoA-I; AltName: Full=Apolipoprotein A1; Contains: RecName
                TRY1_BOVIN  RecName: Full=Cationic trypsin; EC=3.4.21.4; AltName: Full=Beta-trypsin; Contains: RecName: Full=Alpha-trypsin c
                ACTA_PHYPO  RecName: Full=Actin, plasmodial isoform; - OS=Physarum polycephalum (Slime mold).
                ACTB_BOVIN  RecName: Full=Actin, cytoplasmic 1; AltName: Full=Beta-actin; Contains: RecName: Full=Actin, cytoplasmic 1, N-te
                FINC_BOVIN  RecName: Full=Fibronectin; Short=FN; Contains: RecName: Full=Anastellin; Flags: Precursor; - OS=Bos taurus (Bovi
                K1C3_XENLA  RecName: Full=Keratin, type I cytoskeletal 47 kDa; Flags: Fragment; - OS=Xenopus laevis (African clawed frog).
                H4_ACRAS    RecName: Full=Histone H4; - OS=Acrolepiopsis assectella (Leek moth).
```

	SWISSPROT	Decoy	False discovery rate
Peptide matches above identity threshold	124	0	0.00 %
Peptide matches above homology or identity threshold	142	2	1.41 %

Mascot Score Histogram

Ions score is -10*Log(P), where P is the probability that the observed match is a random event.
Individual ions scores > 37 indicate identity or extensive homology (p<0.05).
Protein scores are derived from ions scores as a non-probabilistic basis for ranking protein hits.

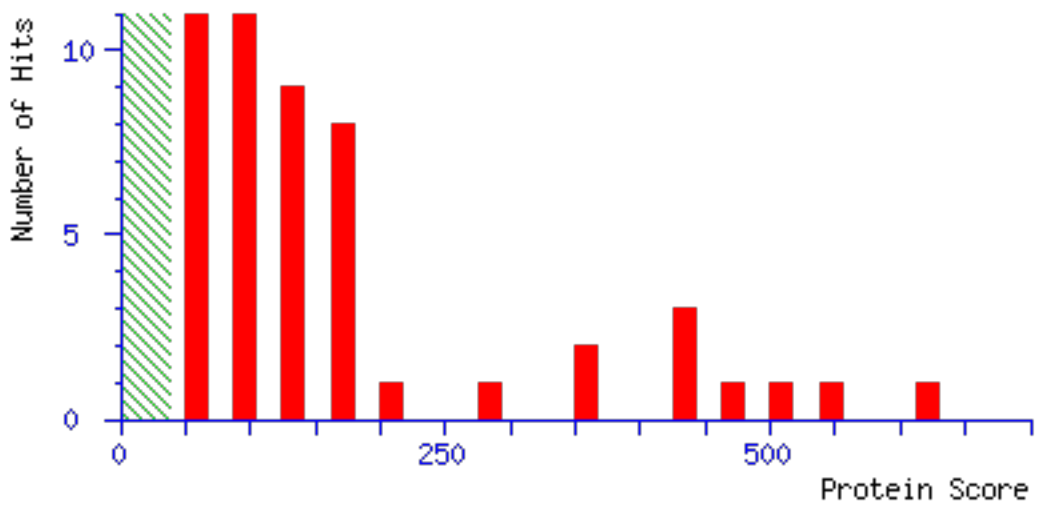

Peptide Summary Report

Format As	Peptide Summary		Help
	Significance threshold p< 0.05	Max. number of hits 20	Show Percolator scores
	Standard scoring ◉ MudPIT scoring ○	Ions score or expect cut-off 0	Show sub-sets 0
	Show pop-ups ◉ Suppress pop-ups ○	Sort unassigned Decreasing Score	Require bold red
	Preferred taxonomy All entries		

1. HS90A_BOVIN **Mass:** 84678 **Score:** 622 **Matches:** 18(14) **Sequences:** 11(8) **emPAI:** 0.35
RecName: Full=Heat shock protein HSP 90-alpha; - OS=Bos taurus (Bovine).
Check to include this hit in error tolerant search or archive report

Query	Observed	Mr(expt)	Mr(calc)	ppm	Miss	Score	Expect	Rank	Unique	Retention Time	Peptide
30	408.2577	814.5009	814.5065	-6.87	0	43	0.017	1	U	828s (13.80m)	R.ALLFVPR.R
31	408.2577	814.5009	814.5065	-6.87	0	(41)	0.031	1	U	825s (13.75m)	R.ALLFVPR.R
59	451.2642	900.5138	900.5181	-4.84	0	27	1.2	1		548s (9.13m)	K.TKPIWTR.N
60	451.2642	900.5139	900.5181	-4.71	0	(15)	18	1		551s (9.18m)	K.TKPIWTR.N
74	474.7247	947.4348	947.4389	-4.31	0	31	0.12	1		566s (9.43m)	K.FYEQFSK.N
130	576.2808	1150.5471	1150.5506	-3.06	0	46	0.0056	1		525s (8.75m)	K.YIDQEELNK.T
170	612.8124	1223.6102	1223.6186	-6.88	0	(42)	0.02	1	U	557s (9.28m)	K.HIYYITGETK.D
171	612.8124	1223.6102	1223.6186	-6.88	0	43	0.017	1	U	554s (9.23m)	K.HIYYITGETK.D
174	618.3022	1234.5899	1234.5942	-3.45	0	55	0.00069	1	U	560s (9.33m)	K.DQVANSAFVER.L
175	621.8527	1241.6909	1241.6979	-5.68	0	64	0.00016	1		765s (12.75m)	K.ADLINNLGTIAK.S
176	621.8527	1241.6909	1241.6979	-5.68	0	(46)	0.01	1		768s (12.80m)	K.ADLINNLGTIAK.S
203	656.2866	1310.5587	1310.5626	-3.01	0	11	4.6	1		542s (9.03m)	K.EDQTEYLEER.R
220	675.3614	1348.7083	1348.7272	-14.04	0	(67)	8.2e-05	1	U	779s (12.98m)	R.TLTIVDTGIGMTK.A
221	675.3614	1348.7083	1348.7272	-14.04	0	83	2.1e-06	1	U	776s (12.93m)	R.TLTIVDTGIGMTK.A
341	917.3919	1832.7693	1832.7741	-2.61	0	(87)	7.1e-08	1	U	666s (11.10m)	R.NPDDITNEEYGEFYK.S
342	917.3919	1832.7693	1832.7741	-2.61	0	102	2e-09	1	U	663s (11.05m)	R.NPDDITNEEYGEFYK.S
361	672.3538	2014.0395	2014.0371	1.21	1	(97)	7.5e-08	1		627s (10.45m)	K.VILHLKEDQTEYLEER.R
362	672.3538	2014.0395	2014.0371	1.21	1	115	1.3e-09	1		623s (10.38m)	K.VILHLKEDQTEYLEER.R

Proteins matching the same set of peptides:
HS90A_HORSE **Mass:** 84717 **Score:** 622 **Matches:** 18(14) **Sequences:** 11(8)
RecName: Full=Heat shock protein HSP 90-alpha; - OS=Equus caballus (Horse).
HS90A_HUMAN **Mass:** 84607 **Score:** 622 **Matches:** 18(14) **Sequences:** 11(8)
RecName: Full=Heat shock protein HSP 90-alpha; AltName: Full=Heat shock 86 kDa; Short=HSP 86; Short=HSP86; AltName: Full=Renal carcinoma
HS90A_MACFA **Mass:** 84736 **Score:** 622 **Matches:** 18(14) **Sequences:** 11(8)
RecName: Full=Heat shock protein HSP 90-alpha; - OS=Macaca fascicularis (Crab-eating macaque) (Cynomolgus monkey).
HS90A_PIG **Mass:** 84722 **Score:** 622 **Matches:** 18(14) **Sequences:** 11(8)
RecName: Full=Heat shock protein HSP 90-alpha; - OS=Sus scrofa (Pig).

---

2. K2C1_HUMAN **Mass:** 65999 **Score:** 556 **Matches:** 14(13) **Sequences:** 8(7) **emPAI:** 0.55
RecName: Full=Keratin, type II cytoskeletal 1; AltName: Full=67 kDa cytokeratin; AltName: Full=Cytokeratin-1; Short=CK-1; AltName: Full=

☐ Check to include this hit in error tolerant search or archive report

	Query	Observed	Mr(expt)	Mr(calc)	ppm	Miss	Score	Expect	Rank	Unique	Retention Time	Peptide
☑	182	633.3200	1264.6255	1264.6299	-3.50	0	38	0.054	1	U	630s (10.50m)	R.TNAENEFVTIK.K
☑	188	639.3559	1276.6972	1276.7027	-4.24	0	(70)	3.7e-05	1	U	947s (15.78m)	K.LALDLEIATYR.T
☑	189	639.3559	1276.6972	1276.7027	-4.24	0	75	1.2e-05	1	U	950s (15.83m)	K.LALDLEIATYR.T
☑	197	651.8573	1301.7000	1301.7078	-6.02	0	70	4.7e-05	1		1128s (18.80m)	R.SLDLDSIIAEVK.A
☑	198	651.8573	1301.7000	1301.7078	-6.02	0	(69)	5.9e-05	1		1130s (18.83m)	R.SLDLDSIIAEVK.A
☑	229	465.2465	1392.7178	1392.7249	-5.10	1	77	7.4e-06	1	U	554s (9.23m)	R.TNAENEFVTIKK.D
☑	253	738.3768	1474.7391	1474.7416	-1.67	0	(67)	8.7e-05	1	U	823s (13.72m)	K.WELLQQVDTSTR.T
☑	254	738.3768	1474.7391	1474.7416	-1.67	0	67	8e-05	1	U	819s (13.65m)	K.WELLQQVDTSTR.T
☑	255	738.3921	1474.7696	1474.7780	-5.65	0	38	0.081	1		595s (9.92m)	R.FLEQQNQVLQTK.W
☑	305	563.3262	1686.9567	1686.9628	-3.63	1	(109)	2.7e-09	1	U	732s (12.20m)	R.SLVNLGGSKSISISVAR.G
☑	306	563.3262	1686.9567	1686.9628	-3.63	1	(107)	4.3e-09	1	U	727s (12.12m)	R.SLVNLGGSKSISISVAR.G
☑	307	844.4860	1686.9575	1686.9628	-3.16	1	(79)	3.1e-06	1	U	738s (12.30m)	R.SLVNLGGSKSISISVAR.G
☑	308	844.4860	1686.9575	1686.9628	-3.16	1	113	1.3e-09	1	U	734s (12.23m)	R.SLVNLGGSKSISISVAR.G
☑	316	858.9258	1715.8371	1715.8438	-3.89	0	82	2.5e-06	1	U	625s (10.42m)	K.QISNLQQSISDAEQR.G

Proteins matching the same set of peptides:
K2C1_PANTR **Mass:** 65450 **Score:** 556 **Matches:** 14(13) **Sequences:** 8(7)
RecName: Full=Keratin, type II cytoskeletal 1; AltName: Full=Cytokeratin-1; Short=CK-1; AltName: Full=Keratin-1; Short=K1; AltName: Full

---

3. HS90B_HORSE **Mass:** 83185 **Score:** 525 **Matches:** 16(11) **Sequences:** 10(6) **emPAI:** 0.36
RecName: Full=Heat shock protein HSP 90-beta; - OS=Equus caballus (Horse).

☐ Check to include this hit in error tolerant search or archive report

	Query	Observed	Mr(expt)	Mr(calc)	ppm	Miss	Score	Expect	Rank	Unique	Retention Time	Peptide
	59	451.2642	900.5138	900.5181	-4.84	0	27	1.2	1		548s (9.13m)	K.TKPIWTR.N
	60	451.2642	900.5139	900.5181	-4.71	0	(15)	18	1		551s (9.18m)	K.TKPIWTR.N
	130	576.2808	1150.5471	1150.5506	-3.06	0	46	0.0056	1		525s (8.75m)	K.YIDQEELNK.T
☑	133	580.7921	1159.5696	1159.5761	-5.62	0	30	0.3	1	U	615s (10.25m)	K.SIYYITGESK.E
☑	153	597.8254	1193.6363	1193.6404	-3.44	0	(50)	0.0037	1	U	678s (11.30m)	K.IDIIPNPQER.T
☑	154	597.8254	1193.6363	1193.6404	-3.44	0	56	0.00086	1	U	675s (11.25m)	K.IDIIPNPQER.T
	175	621.8527	1241.6909	1241.6979	-5.68	0	64	0.00016	1		765s (12.75m)	K.ADLINNLGTIAK.S
	176	621.8527	1241.6909	1241.6979	-5.68	0	(46)	0.01	1		768s (12.80m)	K.ADLINNLGTIAK.S
☑	180	625.3094	1248.6042	1248.6098	-4.52	0	19	3.8	1	U	555s (9.25m)	K.EQVANSAFVER.V
	203	656.2866	1310.5587	1310.5626	-3.01	0	11	4.6	1		542s (9.03m)	K.EDQTEYLEER.R
	220	675.3614	1348.7083	1348.7272	-14.04	0	(67)	8.2e-05	1	U	779s (12.98m)	R.TLTLVDTGIGMTK.A
	221	675.3614	1348.7083	1348.7272	-14.04	0	83	2.1e-06	1	U	776s (12.93m)	R.TLTLVDTGIGMTK.A
☑	66	924.3994	1846.7843	1846.7897	-2.92	0	(42)	0.0086	1	U	679s (11.32m)	R.NPDDITQEEYGEFYK.S
☑	346	924.4019	1846.7893	1846.7897	-0.21	0	74	2.1e-06	1	U	676s (11.27m)	R.NPDDITQEEYGEFYK.S
	361	672.3538	2014.0395	2014.0371	1.21	1	(97)	7.5e-08	1		627s (10.45m)	K.VILHLKEDQTEYLEER.R
	362	672.3538	2014.0395	2014.0371	1.21	1	115	1.3e-09	1		623s (10.38m)	K.VILHLKEDQTEYLEER.R

Proteins matching the same set of peptides:
HS90B_HUMAN **Mass:** 83212 **Score:** 525 **Matches:** 16(11) **Sequences:** 10(6)
RecName: Full=Heat shock protein HSP 90-beta; Short=HSP 90; AltName: Full=Heat shock 84 kDa; Short=HSP 84; Short=HSP84; - OS=Homo sapien
HS90B_MACFA **Mass:** 83185 **Score:** 525 **Matches:** 16(11) **Sequences:** 10(6)
RecName: Full=Heat shock protein HSP 90-beta; - OS=Macaca fascicularis (Crab-eating macaque) (Cynomolgus monkey).
HS90B_MOUSE **Mass:** 83229 **Score:** 525 **Matches:** 16(11) **Sequences:** 10(6)
RecName: Full=Heat shock protein HSP 90-beta; AltName: Full=Heat shock 84 kDa; Short=HSP 84; Short=HSP84; AltName: Full=Tumor-specific t
HS90B_PONAB **Mass:** 83186 **Score:** 525 **Matches:** 16(11) **Sequences:** 10(6)
RecName: Full=Heat shock protein HSP 90-beta; - OS=Pongo abelii (Sumatran orangutan) (Pongo pygmaeus abelii).
HS90B_RAT **Mass:** 83229 **Score:** 525 **Matches:** 16(11) **Sequences:** 10(6)
RecName: Full=Heat shock protein HSP 90-beta; AltName: Full=Heat shock 84 kDa; Short=HSP 84; Short=HSP84; - OS=Rattus norvegicus (Rat).

---

4. K1C10_HUMAN **Mass:** 58792 **Score:** 481 **Matches:** 11(8) **Sequences:** 8(6) **emPAI:** 0.39
RecName: Full=Keratin, type I cytoskeletal 10; AltName: Full=Cytokeratin-10; Short=CK-10; AltName: Full=Keratin-10; Short=K10; - OS=Homo

☐ Check to include this hit in error tolerant search or archive report

	Query	Observed	Mr(expt)	Mr(calc)	ppm	Miss	Score	Expect	Rank	Unique	Retention Time	Peptide
☑	113	532.8062	1063.5979	1063.6026	-4.38	1	48	0.0046	1		564s (9.40m)	R.LASYLDKVR.A
☑	160	601.3070	1200.5995	1200.6098	-8.63	0	77	5e-06	1		612s (10.20m)	R.QSVEADINGLR.R
☑	173	412.2295	1233.6667	1233.6717	-4.02	1	60	0.00036	1		553s (9.22m)	R.LKYENEVALR.Q
☑	13	679.3589	1356.7033	1356.7110	-5.65	1	(14)	39	1		572s (9.53m)	R.QSVEADINGLRR.V
☑	224	679.3609	1356.7073	1356.7110	-2.72	1	26	0.97	1		568s (9.47m)	R.QSVEADINGLRR.V
☑	225	691.3211	1380.6277	1380.6408	-9.53	0	(44)	0.0058	1		558s (9.30m)	R.ALEESNYELEGK.I
☑	226	691.3211	1380.6277	1380.6408	-9.53	0	68	2.3e-05	1		555s (9.25m)	R.ALEESNYELEGK.I
☑	242	717.8833	1433.7520	1433.7626	-7.43	1	38	0.071	1		598s (9.97m)	K.IRLENEIQTYR.S
☑	314	854.3888	1706.7630	1706.7649	-1.09	0	(90)	1.1e-07	1	U	711s (11.85m)	K.GSLGGGFSSGGFSGGSFSR.G

315	854.3888	1706.7630	1706.7649	-1.09	0	110	1.2e-09	1	U	709s (11.82m)	K.GSLGGGFSSGGFSGGSFSR.G
83	968.7926	2903.3559	2903.3753	-6.68	0	54	0.00073	1	U	990s (16.50m)	R.NVSTGDVNVEMNAAPGVDLTQLLNMR.S + 2 Oxidati[illegible]

---

5. ALBU_BOVIN **Mass:** 69248 **Score:** 437 **Matches:** 12(9) **Sequences:** 7(6) **emPAI:** 0.32
RecName: Full=Serum albumin; AltName: Full=BSA; AltName: Allergen=Bos d 6; Flags: Precursor; - OS=Bos taurus (Bovine).
Check to include this hit in error tolerant search or archive report

Query	Observed	Mr(expt)	Mr(calc)	ppm	Miss	Score	Expect	Rank	Unique	Retention Time	Peptide
90	501.7924	1001.5703	1001.5757	-5.40	0	(1)	3.9e+02	10	U	683s (11.38m)	K.LVVSTQTALA.-
91	501.7924	1001.5703	1001.5757	-5.40	0	13	26	1	U	678s (11.30m)	K.LVVSTQTALA.-
97	507.8102	1013.6058	1013.6121	-6.22	0	55	0.0013	1	U	863s (14.38m)	K.QTALVELLK.H
137	582.3162	1162.6179	1162.6234	-4.73	0	62	0.00026	1	U	736s (12.27m)	K.LVNELTEFAK.T
138	582.3162	1162.6179	1162.6234	-4.73	0	(54)	0.0018	1	U	741s (12.35m)	K.LVNELTEFAK.T
199	653.3567	1304.6988	1304.7088	-7.70	0	(39)	0.054	1	U	609s (10.15m)	K.HLVDEPQNLIK.Q
200	653.3567	1304.6988	1304.7088	-7.70	0	52	0.0029	1	U	605s (10.08m)	K.HLVDEPQNLIK.Q
238	708.3419	1414.6693	1414.6803	-7.77	0	84	7.8e-07	1	U	838s (13.97m)	K.TVMENFVAFVDK.C + Oxidation (M)
260	756.4232	1510.8318	1510.8355	-2.43	0	88	4.5e-07	1	U	702s (11.70m)	K.VPQVSTPTLVEVSR.S
261	756.4232	1510.8318	1510.8355	-2.43	0	(47)	0.0055	1	U	706s (11.77m)	K.VPQVSTPTLVEVSR.S
277	784.3715	1566.7285	1566.7354	-4.44	0	(70)	2e-05	1	U	1182s (19.70m)	K.DAFLGSFLYEYSR.R
278	784.3715	1566.7285	1566.7354	-4.44	0	82	1.1e-06	1	U	1179s (19.65m)	K.DAFLGSFLYEYSR.R

---

6. LG3BP_HUMAN **Mass:** 65289 **Score:** 428 **Matches:** 10(7) **Sequences:** 6(5) **emPAI:** 0.34
RecName: Full=Galectin-3-binding protein; AltName: Full=Basement membrane autoantigen p105; AltName: Full=Lectin galactoside-binding sol[illegible]
Check to include this hit in error tolerant search or archive report

Query	Observed	Mr(expt)	Mr(calc)	ppm	Miss	Score	Expect	Rank	Unique	Retention Time	Peptide
102	515.2658	1028.5170	1028.5251	-7.84	0	30	0.24	1	U	517s (8.62m)	R.STHTLDLSR.E
161	603.7859	1205.5572	1205.5465	8.83	0	(40)	0.019	1	U	727s (12.12m)	K.AVDTWSWGER.A
162	603.7859	1205.5572	1205.5465	8.83	0	45	0.0048	1	U	724s (12.07m)	K.AVDTWSWGER.A
211	663.8268	1325.6391	1325.6463	-5.41	0	(10)	27	1	U	532s (8.87m)	R.ASHEEVEGLVEK.I
213	442.8877	1325.6413	1325.6463	-3.76	0	74	1e-05	1	U	527s (8.78m)	R.ASHEEVEGLVEK.I
222	678.3896	1354.7647	1354.7708	-4.43	0	66	6.8e-05	1	U	983s (16.38m)	R.SDLAVPSELALLK.A
223	678.3896	1354.7647	1354.7708	-4.43	0	(33)	0.12	1	U	978s (16.30m)	R.SDLAVPSELALLK.A
26	796.8929	1591.7712	1591.7842	-8.14	0	110	3.2e-09	1	U	960s (16.00m)	R.ELSEALGQIFDSQR.G
284	799.8380	1597.6615	1597.6685	-4.38	0	102	1.9e-09	1	U	645s (10.75m)	K.YSSDYFQAPSDYR.Y
285	799.8380	1597.6615	1597.6685	-4.38	0	(93)	1.6e-08	1	U	641s (10.68m)	K.YSSDYFQAPSDYR.Y

---

7. K1C10_CANFA **Mass:** 57676 **Score:** 427 **Matches:** 10(7) **Sequences:** 7(5) **emPAI:** 0.32
RecName: Full=Keratin, type I cytoskeletal 10; AltName: Full=Cytokeratin-10; Short=CK-10; AltName: Full=Epithelial keratin-10; AltName:[illegible]
Check to include this hit in error tolerant search or archive report

Query	Observed	Mr(expt)	Mr(calc)	ppm	Miss	Score	Expect	Rank	Unique	Retention Time	Peptide
113	532.8062	1063.5979	1063.6026	-4.38	1	48	0.0046	1		564s (9.40m)	R.LASYLDKVR.A
160	601.3070	1200.5995	1200.6098	-8.63	0	77	5e-06	1		612s (10.20m)	R.QSVEADINGLR.R
173	412.2295	1233.6667	1233.6717	-4.02	1	60	0.00036	1		553s (9.22m)	R.LKYENEVALR.Q
13	679.3589	1356.7033	1356.7110	-5.65	1	(14)	39	1		572s (9.53m)	R.QSVEADINGLRR.V
224	679.3609	1356.7073	1356.7110	-2.72	1	26	0.97	1		568s (9.47m)	R.QSVEADINGLRR.V
225	691.3211	1380.6277	1380.6408	-9.53	0	(44)	0.0058	1		558s (9.30m)	R.ALEESNYELEGK.I
226	691.3211	1380.6277	1380.6408	-9.53	0	68	2.3e-05	1		555s (9.25m)	R.ALEESNYELEGK.I
242	717.8833	1433.7520	1433.7626	-7.43	1	38	0.071	1		598s (9.97m)	K.IRLENEIQTYR.S
314	854.3888	1706.7630	1706.7649	-1.09	0	(90)	1.1e-07	1	U	711s (11.85m)	K.GSIGGGFSSGGFSGGSFSR.G
315	854.3888	1706.7630	1706.7649	-1.09	0	110	1.2e-09	1	U	709s (11.82m)	K.GSIGGGFSSGGFSGGSFSR.G

---

8. ACTN4_BOVIN **Mass:** 104862 **Score:** 360 **Matches:** 8(5) **Sequences:** 6(4) **emPAI:** 0.17
RecName: Full=Alpha-actinin-4; AltName: Full=F-actin cross-linking protein; AltName: Full=Non-muscle alpha-actinin 4; - OS=Bos taurus (B[illegible]
Check to include this hit in error tolerant search or archive report

Query	Observed	Mr(expt)	Mr(calc)	ppm	Miss	Score	Expect	Rank	Unique	Retention Time	Peptide
193	645.2949	1288.5753	1288.5823	-5.45	0	12	8.1	1		850s (14.17m)	K.SFSTALYGESDL.-
194	645.2949	1288.5753	1288.5823	-5.45	0	(6)	26	1		846s (14.10m)	K.SFSTALYGESDL.-
20	769.3997	1536.7849	1536.7671	11.6	0	30	0.89	1		737s (12.28m)	R.FAIQDISVEETSAK.E
270	516.9233	1547.7482	1547.7580	-6.33	1	55	0.00097	1	U	541s (9.02m)	K.HRDYETATLSDIK.A
301	559.3042	1674.8909	1674.8941	-1.89	1	57	0.00096	1	U	694s (11.57m)	K.VLAGDKNFITAEELR.R
321	871.4083	1740.8021	1740.8054	-1.89	0	(84)	8.7e-07	1		780s (13.00m)	R.ETTDTDTADQVIASFK.V
322	871.4083	1740.8021	1740.8054	-1.89	0	94	7.8e-08	1		777s (12.95m)	R.ETTDTDTADQVIASFK.V
333	896.9312	1791.8478	1791.8502	-1.31	0	113	1.4e-09	1	U	719s (11.98m)	R.MAPYQGPDAVPGALDYK.S

Proteins matching the same set of peptides:
ACTN4_HUMAN **Mass:** 104788 **Score:** 360 **Matches:** 8(5) **Sequences:** 6(4)
RecName: Full=Alpha-actinin-4; AltName: Full=F-actin cross-linking protein; AltName: Full=Non-muscle alpha-actinin 4; - OS=Homo sapiens
ACTN4_PONAB **Mass:** 104789 **Score:** 360 **Matches:** 8(5) **Sequences:** 6(4)
RecName: Full=Alpha-actinin-4; AltName: Full=F-actin cross-linking protein; AltName: Full=Non-muscle alpha-actinin 4; - OS=Pongo abelii

---

9. K1C9_HUMAN **Mass:** 62027 **Score:** 353 **Matches:** 4(3) **Sequences:** 4(3) **emPAI:** 0.17
RecName: Full=Keratin, type I cytoskeletal 9; AltName: Full=Cytokeratin-9; Short=CK-9; AltName: Full=Keratin-9; Short=K9; - OS=Homo sapi[illegible]
Check to include this hit in error tolerant search or archive report

Query	Observed	Mr(expt)	Mr(calc)	ppm	Miss	Score	Expect	Rank	Unique	Retention Time	Peptide
338	604.9533	1811.8381	1811.8511	-7.16	1	134	7.1e-12	1	U	509s (8.48m)	R.SGGGGGGGLGSGGSIRSSYSR.F
347	926.4607	1850.9069	1850.9196	-6.86	1	9	46	1	U	793s (13.22m)	K.TLNDMRQEYEQLIAK.N
375	733.6561	2197.9466	2197.9373	4.19	1	97	1.2e-08	1	U	501s (8.35m)	R.FSSSGGGGGGGRFSSSSGYGGGSSR.V

	Query	Observed	Mr(expt)	Mr(calc)	ppm	Miss	Score	Expect	Rank	Unique	Retention Time	Peptide
[x]	391	1075.0939	3222.2600	3222.2744	-4.46	0	114	1.8e-11	1	U	504s (8.40m)	R.GGSGGSHGGGSGFGGESGGSYGGGEEASGSGGGYGGGSGK

10. CO3_HUMAN **Mass:** 187030 **Score:** 285 **Matches:** 4(4) **Sequences:** 3(3) **emPAI:** 0.05
RecName: Full=Complement C3; AltName: Full=C3 and PZP-like alpha-2-macroglobulin domain-containing protein 1; Contains: RecName: Full=Co
[ ] Check to include this hit in error tolerant search or archive report

	Query	Observed	Mr(expt)	Mr(calc)	ppm	Miss	Score	Expect	Rank	Unique	Retention Time	Peptide
[x]	89	501.7697	1001.5248	1001.5393	-14.45	0	51	0.003	1	U	556s (9.27m)	K.TGLQEVEVK.A
[x]	251	735.8895	1469.7645	1469.7726	-5.48	0	(75)	1e-05	1	U	665s (11.08m)	R.IPIEDGSGEVVLSR.K
[x]	252	735.8895	1469.7645	1469.7726	-5.48	0	128	6e-11	1	U	661s (11.02m)	R.IPIEDGSGEVVLSR.K
[x]	371	1079.0439	2156.0733	2156.0783	-2.31	0	106	8.9e-09	1	U	979s (16.32m)	R.ILLQGTPVAQMTEDAVDAER.L

11. K22E_HUMAN **Mass:** 65393 **Score:** 227 **Matches:** 7(3) **Sequences:** 5(2) **emPAI:** 0.22
RecName: Full=Keratin, type II cytoskeletal 2 epidermal; AltName: Full=Cytokeratin-2e; Short=CK-2e; AltName: Full=Epithelial keratin-2e;
[ ] Check to include this hit in error tolerant search or archive report

	Query	Observed	Mr(expt)	Mr(calc)	ppm	Miss	Score	Expect	Rank	Unique	Retention Time	Peptide
[x]	6	519.2647	1036.5149	1036.5189	-3.90	0	40	0.061	1	U	604s (10.07m)	R.YLDGLTAER.T
[x]	214	665.3643	1328.7140	1328.7187	-3.58	0	54	0.0014	1		1125s (18.75m)	R.NLDLDSIIAEVK.A
[x]	215	665.3643	1328.7140	1328.7187	-3.58	0	(32)	0.25	1		1132s (18.87m)	R.NLDLDSIIAEVK.A
[x]	218	668.8520	1335.6895	1335.7034	-10.41	1	11	31	1	U	565s (9.42m)	R.TAAENDFVTLKK.D
[x]	246	730.9007	1459.7867	1459.7922	-3.75	0	83	2.1e-06	1	U	1080s (18.00m)	K.VDLLNQEIEFLK.V
[x]	247	730.9015	1459.7884	1459.7922	-2.63	0	(57)	0.00093	1	U	1085s (18.08m)	K.VDLLNQEIEFLK.V
	255	738.3921	1474.7696	1474.7780	-5.65	0	38	0.081	1		595s (9.92m)	R.FLEQQNQVLQTK.W

12. 1433Z_BOVIN **Mass:** 27728 **Score:** 187 **Matches:** 4(4) **Sequences:** 3(3) **emPAI:** 0.41
RecName: Full=14-3-3 protein zeta/delta; AltName: Full=Factor activating exoenzyme S; Short=FAS; AltName: Full=Protein kinase C inhibito
[ ] Check to include this hit in error tolerant search or archive report

	Query	Observed	Mr(expt)	Mr(calc)	ppm	Miss	Score	Expect	Rank	Unique	Retention Time	Peptide
[x]	61	454.2634	906.5123	906.5174	-5.71	0	(43)	0.015	1		686s (11.43m)	R.NLLSVAYK.N
[x]	62	454.2634	906.5123	906.5174	-5.71	0	50	0.0027	1		681s (11.35m)	R.NLLSVAYK.N
[x]	190	427.2228	1278.6467	1278.6456	0.89	1	49	0.0051	1	U	517s (8.62m)	R.YLAEVAAGDDKK.G
[x]	269	774.8569	1547.6993	1547.7063	-4.51	0	88	2e-07	1	U	512s (8.53m)	K.SVTEQGAELSNEER.N

Proteins matching the same set of peptides:
1433Z_CHICK **Mass:** 27758 **Score:** 187 **Matches:** 4(4) **Sequences:** 3(3)
RecName: Full=14-3-3 protein zeta; - OS=Gallus gallus (Chicken).
1433Z_HUMAN **Mass:** 27728 **Score:** 187 **Matches:** 4(4) **Sequences:** 3(3)
RecName: Full=14-3-3 protein zeta/delta; AltName: Full=Protein kinase C inhibitor protein 1; Short=KCIP-1; - OS=Homo sapiens (Human).
1433Z_MOUSE **Mass:** 27754 **Score:** 187 **Matches:** 4(4) **Sequences:** 3(3)
RecName: Full=14-3-3 protein zeta/delta; AltName: Full=Protein kinase C inhibitor protein 1; Short=KCIP-1; AltName: Full=SEZ-2; - OS=Mus
1433Z_PONAB **Mass:** 27728 **Score:** 187 **Matches:** 4(4) **Sequences:** 3(3)
RecName: Full=14-3-3 protein zeta/delta; - OS=Pongo abelii (Sumatran orangutan) (Pongo pygmaeus abelii).
1433Z_RAT **Mass:** 27754 **Score:** 187 **Matches:** 4(4) **Sequences:** 3(3)
RecName: Full=14-3-3 protein zeta/delta; AltName: Full=Mitochondrial import stimulation factor S1 subunit; AltName: Full=Protein kinase
1433Z_SHEEP **Mass:** 27839 **Score:** 187 **Matches:** 4(4) **Sequences:** 3(3)
RecName: Full=14-3-3 protein zeta/delta; AltName: Full=Protein kinase C inhibitor protein 1; Short=KCIP-1; - OS=Ovis aries (Sheep).

13. ACTN4_CHICK **Mass:** 104142 **Score:** 173 **Matches:** 6(2) **Sequences:** 4(1) **emPAI:** 0.06
RecName: Full=Alpha-actinin-4; AltName: Full=F-actin cross-linking protein; AltName: Full=Non-muscle alpha-actinin 4; - OS=Gallus gallus
[ ] Check to include this hit in error tolerant search or archive report

Query	Observed	Mr(expt)	Mr(calc)	ppm	Miss	Score	Expect	Rank	Unique	Retention Time	Peptide
193	645.2949	1288.5753	1288.5823	-5.45	0	12	8.1	1		850s (14.17m)	K.SFSTALYGESDL.-
194	645.2949	1288.5753	1288.5823	-5.45	0	(6)	26	1		846s (14.10m)	K.SFSTALYGESDL.-
20	769.3997	1536.7849	1536.7671	11.6	0	30	0.89	1		737s (12.28m)	R.FAIQDISVEETSAK.E
321	871.4083	1740.8021	1740.8054	-1.89	0	(84)	8.7e-07	1		780s (13.00m)	R.ETTDTDTADQVIASFK.V
322	871.4083	1740.8021	1740.8054	-1.89	0	94	7.8e-08	1		777s (12.95m)	R.ETTDTDTADQVIASFK.V
333	896.9312	1791.8478	1791.8614	-7.57	1	37	0.055	2	U	719s (11.98m)	R.MAPYRGPDAAPGALDYK.S

14. APOA1_BOVIN **Mass:** 30258 **Score:** 165 **Matches:** 4(4) **Sequences:** 2(2) **emPAI:** 0.23
RecName: Full=Apolipoprotein A-I; Short=Apo-AI; Short=ApoA-I; AltName: Full=Apolipoprotein A1; Contains: RecName: Full=Truncated apolipo
[ ] Check to include this hit in error tolerant search or archive report

	Query	Observed	Mr(expt)	Mr(calc)	ppm	Miss	Score	Expect	Rank	Unique	Retention Time	Peptide
[x]	166	608.8403	1215.6661	1215.6710	-4.02	0	61	0.0004	1	U	944s (15.73m)	K.VSILAAIDEASK.K
[x]	167	608.8403	1215.6661	1215.6710	-4.02	0	(52)	0.0029	1	U	946s (15.77m)	K.VSILAAIDEASK.K
[x]	280	788.9106	1575.8066	1575.8144	-4.95	0	104	1.6e-08	1	U	1057s (17.62m)	K.LLDNWDTLASTLSK.V
[x]	281	788.9106	1575.8066	1575.8144	-4.95	0	(95)	1.3e-07	1	U	1054s (17.57m)	K.LLDNWDTLASTLSK.V

15. TRY1_BOVIN **Mass:** 25769 **Score:** 161 **Matches:** 5(5) **Sequences:** 2(2) **emPAI:** 0.44
RecName: Full=Cationic trypsin; EC=3.4.21.4; AltName: Full=Beta-trypsin; Contains: RecName: Full=Alpha-trypsin chain 1; Contains: RecNam
[ ] Check to include this hit in error tolerant search or archive report

	Query	Observed	Mr(expt)	Mr(calc)	ppm	Miss	Score	Expect	Rank	Unique	Retention Time	Peptide
[x]	372	721.6878	2162.0417	2162.0491	-3.45	0	(72)	1.9e-05	1	U	742s (12.37m)	R.LGEDNINVVEGNEQFISASK.S
[x]	373	1082.0292	2162.0438	2162.0491	-2.47	0	(65)	9.4e-05	1	U	738s (12.30m)	R.LGEDNINVVEGNEQFISASK.S
[x]	374	1082.0292	2162.0438	2162.0491	-2.47	0	93	1.7e-07	1	U	735s (12.25m)	R.LGEDNINVVEGNEQFISASK.S

	Query	Observed	Mr(expt)	Mr(calc)	ppm	Miss	Score	Expect	Rank	Unique	Retention Time	Peptide
☑	378	763.7222	2288.1449	2288.1470	-0.96	0	68	5.2e-05	1	U	765s (12.75m)	K.SIVHPSYNSNTLNNDIMLIK.L + Oxidation (M)
☑	379	763.7222	2288.1449	2288.1470	-0.96	0	(68)	6e-05	1	U	769s (12.82m)	K.SIVHPSYNSNTLNNDIMLIK.L + Oxidation (M)

---

16. ACTA_PHYPO **Mass:** 41773 **Score:** 157 **Matches:** 6(5) **Sequences:** 3(3) **emPAI:** 0.36
RecName: Full=Actin, plasmodial isoform; – OS=Physarum polycephalum (Slime mold).
☐ Check to include this hit in error tolerant search or archive report

	Query	Observed	Mr(expt)	Mr(calc)	ppm	Miss	Score	Expect	Rank	Unique	Retention Time	Peptide
☑	25	398.2380	794.4614	794.4650	-4.54	0	38	0.023	1		530s (8.83m)	K.IIAPPER.K
☑	134	581.3127	1160.6108	1160.6111	-0.20	0	53	0.0022	1	U	626s (10.43m)	K.ELTALAPSTMK.I
☑	135	581.3127	1160.6108	1160.6111	-0.20	0	(49)	0.0062	1	U	623s (10.38m)	K.ELTALAPSTMK.I
☑	141	589.3102	1176.6057	1176.6060	-0.22	0	(27)	0.92	1	U	568s (9.47m)	K.ELTALAPSTMK.I + Oxidation (M)
☑	357	652.0240	1953.0501	1953.0571	-3.58	0	(61)	0.00021	1		722s (12.03m)	R.VAPEEHPVLLTEAPLNPK.A
☑	358	652.0240	1953.0501	1953.0571	-3.58	0	66	8.1e-05	1		725s (12.08m)	R.VAPEEHPVLLTEAPLNPK.A

Proteins matching the same set of peptides:
ACT1_ACACA **Mass:** 41649 **Score:** 157 **Matches:** 6(5) **Sequences:** 3(3)
RecName: Full=Actin-1; – OS=Acanthamoeba castellanii (Amoeba).
ACT1_DICDI **Mass:** 41706 **Score:** 157 **Matches:** 6(5) **Sequences:** 3(3)
RecName: Full=Major actin; AltName: Full=Actin A1; AltName: Full=Actin A12; AltName: Full=Actin A8; AltName: Full=Actin III; AltName: Fu
ACT3_DICDI **Mass:** 41814 **Score:** 157 **Matches:** 6(5) **Sequences:** 3(3)
RecName: Full=Actin-3; AltName: Full=Actin-3-sub 1; – OS=Dictyostelium discoideum (Slime mold).
ACT10_DICDI **Mass:** 41720 **Score:** 157 **Matches:** 6(5) **Sequences:** 3(3)
RecName: Full=Actin-10; – OS=Dictyostelium discoideum (Slime mold).
ACT23_DICDI **Mass:** 39938 **Score:** 157 **Matches:** 6(5) **Sequences:** 3(3)
RecName: Full=Putative actin-23; – OS=Dictyostelium discoideum (Slime mold).

---

17. ACTB_BOVIN **Mass:** 41710 **Score:** 157 **Matches:** 6(5) **Sequences:** 3(3) **emPAI:** 0.36
RecName: Full=Actin, cytoplasmic 1; AltName: Full=Beta-actin; Contains: RecName: Full=Actin, cytoplasmic 1, N-terminally processed; – OS
☐ Check to include this hit in error tolerant search or archive report

Query	Observed	Mr(expt)	Mr(calc)	ppm	Miss	Score	Expect	Rank	Unique	Retention Time	Peptide
25	398.2380	794.4614	794.4650	-4.54	0	38	0.023	1		530s (8.83m)	K.IIAPPER.K
134	581.3127	1160.6108	1160.6111	-0.20	0	53	0.0022	1	U	626s (10.43m)	K.EITALAPSTMK.I
135	581.3127	1160.6108	1160.6111	-0.20	0	(49)	0.0062	1	U	623s (10.38m)	K.EITALAPSTMK.I
141	589.3102	1176.6057	1176.6060	-0.22	0	(27)	0.92	1	U	568s (9.47m)	K.EITALAPSTMK.I + Oxidation (M)
357	652.0240	1953.0501	1953.0571	-3.58	0	(61)	0.00021	1		722s (12.03m)	R.VAPEEHPVLLTEAPLNPK.A
358	652.0240	1953.0501	1953.0571	-3.58	0	66	8.1e-05	1		725s (12.08m)	R.VAPEEHPVLLTEAPLNPK.A

Proteins matching the same set of peptides:
ACTB_CAMDR **Mass:** 41775 **Score:** 157 **Matches:** 6(5) **Sequences:** 3(3)
RecName: Full=Actin, cytoplasmic 1; AltName: Full=Beta-actin; Contains: RecName: Full=Actin, cytoplasmic 1, N-terminally processed; – OS
ACTB_CANFA **Mass:** 41710 **Score:** 157 **Matches:** 6(5) **Sequences:** 3(3)
RecName: Full=Actin, cytoplasmic 1; AltName: Full=Beta-actin; Contains: RecName: Full=Actin, cytoplasmic 1, N-terminally processed; – OS
ACTB_CAVPO **Mass:** 41710 **Score:** 157 **Matches:** 6(5) **Sequences:** 3(3)
RecName: Full=Actin, cytoplasmic 1; AltName: Full=Beta-actin; Contains: RecName: Full=Actin, cytoplasmic 1, N-terminally processed; – OS
ACTB_CERPY **Mass:** 40419 **Score:** 157 **Matches:** 6(5) **Sequences:** 3(3)
RecName: Full=Actin, cytoplasmic 1; AltName: Full=Beta-actin; – OS=Cercopithecus pygerythrus (Vervet monkey).
ACTB_CHICK **Mass:** 41710 **Score:** 157 **Matches:** 6(5) **Sequences:** 3(3)
RecName: Full=Actin, cytoplasmic 1; AltName: Full=Beta-actin; Contains: RecName: Full=Actin, cytoplasmic 1, N-terminally processed; – OS
ACTB_CHLAE **Mass:** 41710 **Score:** 157 **Matches:** 6(5) **Sequences:** 3(3)
RecName: Full=Actin, cytoplasmic 1; AltName: Full=Beta-actin; Contains: RecName: Full=Actin, cytoplasmic 1, N-terminally processed; – OS
ACTB_CRIGR **Mass:** 41711 **Score:** 157 **Matches:** 6(5) **Sequences:** 3(3)
RecName: Full=Actin, cytoplasmic 1; AltName: Full=Beta-actin; Contains: RecName: Full=Actin, cytoplasmic 1, N-terminally processed; – OS
ACTB_HORSE **Mass:** 41710 **Score:** 157 **Matches:** 6(5) **Sequences:** 3(3)
RecName: Full=Actin, cytoplasmic 1; AltName: Full=Beta-actin; Contains: RecName: Full=Actin, cytoplasmic 1, N-terminally processed; – OS
ACTB_HUMAN **Mass:** 41710 **Score:** 157 **Matches:** 6(5) **Sequences:** 3(3)
RecName: Full=Actin, cytoplasmic 1; AltName: Full=Beta-actin; Contains: RecName: Full=Actin, cytoplasmic 1, N-terminally processed; – OS
ACTB_MACFA **Mass:** 41710 **Score:** 157 **Matches:** 6(5) **Sequences:** 3(3)
RecName: Full=Actin, cytoplasmic 1; AltName: Full=Beta-actin; Contains: RecName: Full=Actin, cytoplasmic 1, N-terminally processed; – OS
ACTB_MESAU **Mass:** 41710 **Score:** 157 **Matches:** 6(5) **Sequences:** 3(3)
RecName: Full=Actin, cytoplasmic 1; AltName: Full=Beta-actin; Contains: RecName: Full=Actin, cytoplasmic 1, N-terminally processed; – OS
ACTB_MOUSE **Mass:** 41710 **Score:** 157 **Matches:** 6(5) **Sequences:** 3(3)
RecName: Full=Actin, cytoplasmic 1; AltName: Full=Beta-actin; Contains: RecName: Full=Actin, cytoplasmic 1, N-terminally processed; – OS
ACTB_OREMO **Mass:** 41740 **Score:** 157 **Matches:** 6(5) **Sequences:** 3(3)
RecName: Full=Actin, cytoplasmic 1; AltName: Full=Beta-actin; Contains: RecName: Full=Actin, cytoplasmic 1, N-terminally processed; – OS
ACTB_PANTR **Mass:** 41710 **Score:** 157 **Matches:** 6(5) **Sequences:** 3(3)
RecName: Full=Actin, cytoplasmic 1; AltName: Full=Beta-actin; Contains: RecName: Full=Actin, cytoplasmic 1, N-terminally processed; – OS
ACTB_PIG **Mass:** 41710 **Score:** 157 **Matches:** 6(5) **Sequences:** 3(3)
RecName: Full=Actin, cytoplasmic 1; AltName: Full=Beta-actin; Contains: RecName: Full=Actin, cytoplasmic 1, N-terminally processed; – OS
ACTB_PONAB **Mass:** 41710 **Score:** 157 **Matches:** 6(5) **Sequences:** 3(3)
RecName: Full=Actin, cytoplasmic 1; AltName: Full=Beta-actin; Contains: RecName: Full=Actin, cytoplasmic 1, N-terminally processed; – OS
ACTB_RAT **Mass:** 41710 **Score:** 157 **Matches:** 6(5) **Sequences:** 3(3)
RecName: Full=Actin, cytoplasmic 1; AltName: Full=Beta-actin; Contains: RecName: Full=Actin, cytoplasmic 1, N-terminally processed; – OS
ACTB_SHEEP **Mass:** 41710 **Score:** 157 **Matches:** 6(5) **Sequences:** 3(3)
RecName: Full=Actin, cytoplasmic 1; AltName: Full=Beta-actin; Contains: RecName: Full=Actin, cytoplasmic 1, N-terminally processed; – OS
ACTB_SIGHI **Mass:** 41692 **Score:** 157 **Matches:** 6(5) **Sequences:** 3(3)
RecName: Full=Actin, cytoplasmic 1; AltName: Full=Beta-actin; Contains: RecName: Full=Actin, cytoplasmic 1, N-terminally processed; – OS
ACTB_SPECI **Mass:** 41710 **Score:** 157 **Matches:** 6(5) **Sequences:** 3(3)
RecName: Full=Actin, cytoplasmic 1; AltName: Full=Beta-actin; Contains: RecName: Full=Actin, cytoplasmic 1, N-terminally processed; – OS
ACTB_TRIVU **Mass:** 41710 **Score:** 157 **Matches:** 6(5) **Sequences:** 3(3)
RecName: Full=Actin, cytoplasmic 1; AltName: Full=Beta-actin; Contains: RecName: Full=Actin, cytoplasmic 1, N-terminally processed; – OS

ACTB_XENBO Mass: 41821 Score: 157 Matches: 6(5) Sequences: 3(3)
RecName: Full=Actin, cytoplasmic 1; AltName: Full=Beta actin; - OS=Xenopus borealis (Kenyan clawed frog).
ACTB_XENLA Mass: 41740 Score: 157 Matches: 6(5) Sequences: 3(3)
RecName: Full=Actin, cytoplasmic 1; AltName: Full=Beta-actin; AltName: Full=Cytoplasmic beta-actin; Contains: RecName: Full=Actin, cytop
ACTB_XENTR Mass: 41726 Score: 157 Matches: 6(5) Sequences: 3(3)
RecName: Full=Actin, cytoplasmic 1; AltName: Full=Beta-actin; Contains: RecName: Full=Actin, cytoplasmic 1, N-terminally processed; - OS
ACTC_BRABE Mass: 41678 Score: 157 Matches: 6(5) Sequences: 3(3)
RecName: Full=Actin, cytoplasmic; AltName: Full=BbCA1; Contains: RecName: Full=Actin, cytoplasmic, N-terminally processed; - OS=Branchic
ACTC_BRAFL Mass: 41694 Score: 157 Matches: 6(5) Sequences: 3(3)
RecName: Full=Actin, cytoplasmic; AltName: Full=BfCA1; Contains: RecName: Full=Actin, cytoplasmic, N-terminally processed; - OS=Branchic
ACTG_ANSAN Mass: 41851 Score: 157 Matches: 6(5) Sequences: 3(3)
RecName: Full=Actin, cytoplasmic 2; AltName: Full=Gamma-actin; Contains: RecName: Full=Actin, cytoplasmic 2, N-terminally processed; - C
ACTG_BOVIN Mass: 41766 Score: 157 Matches: 6(5) Sequences: 3(3)
RecName: Full=Actin, cytoplasmic 2; AltName: Full=Gamma-actin; Contains: RecName: Full=Actin, cytoplasmic 2, N-terminally processed; - C
ACTG_CHICK Mass: 41766 Score: 157 Matches: 6(5) Sequences: 3(3)
RecName: Full=Actin, cytoplasmic 2; AltName: Full=Gamma-actin; Contains: RecName: Full=Actin, cytoplasmic 2, N-terminally processed; - C
ACTG_HUMAN Mass: 41766 Score: 157 Matches: 6(5) Sequences: 3(3)
RecName: Full=Actin, cytoplasmic 2; AltName: Full=Gamma-actin; Contains: RecName: Full=Actin, cytoplasmic 2, N-terminally processed; - C
ACTG_MOUSE Mass: 41766 Score: 157 Matches: 6(5) Sequences: 3(3)
RecName: Full=Actin, cytoplasmic 2; AltName: Full=Gamma-actin; Contains: RecName: Full=Actin, cytoplasmic 2, N-terminally processed; - C
ACTG_RANLE Mass: 41752 Score: 157 Matches: 6(5) Sequences: 3(3)
RecName: Full=Actin, cytoplasmic 2; AltName: Full=Cytoplasmic actin type 5; AltName: Full=Gamma-actin; Contains: RecName: Full=Actin, cy
ACTG_RAT Mass: 41766 Score: 157 Matches: 6(5) Sequences: 3(3)
RecName: Full=Actin, cytoplasmic 2; AltName: Full=Gamma-actin; Contains: RecName: Full=Actin, cytoplasmic 2, N-terminally processed; - C
ACTG_TRISC Mass: 41724 Score: 157 Matches: 6(5) Sequences: 3(3)
RecName: Full=Actin, cytoplasmic 2; AltName: Full=Gamma-actin; Contains: RecName: Full=Actin, cytoplasmic 2, N-terminally processed; - C
ACTG_TRIVU Mass: 41766 Score: 157 Matches: 6(5) Sequences: 3(3)
RecName: Full=Actin, cytoplasmic 2; AltName: Full=Gamma-actin; Contains: RecName: Full=Actin, cytoplasmic 2, N-terminally processed; - C
ACTG_XENLA Mass: 41766 Score: 157 Matches: 6(5) Sequences: 3(3)
RecName: Full=Actin, cytoplasmic 2; AltName: Full=Gamma-actin; Contains: RecName: Full=Actin, cytoplasmic 2, N-terminally processed; - C
ACTG_XENTR Mass: 41738 Score: 157 Matches: 6(5) Sequences: 3(3)
RecName: Full=Actin, cytoplasmic 2; AltName: Full=Actin, cytoplasmic, type 5; AltName: Full=Gamma-actin; Contains: RecName: Full=Actin,
ACTM_STYPL Mass: 42327 Score: 157 Matches: 6(5) Sequences: 3(3)
RecName: Full=Actin, muscle; - OS=Styela plicata (Sea squirt) (Ascidia plicata).
ACT_BRUMA Mass: 41683 Score: 157 Matches: 6(5) Sequences: 3(3)
RecName: Full=Actin; - OS=Brugia malayi (Filarial nematode worm).
ACT_PLAMG Mass: 41735 Score: 157 Matches: 6(5) Sequences: 3(3)
RecName: Full=Actin, adductor muscle; Flags: Precursor; - OS=Placopecten magellanicus (Sea scallop).
ACT1_CAEEL Mass: 41769 Score: 157 Matches: 6(5) Sequences: 3(3)
RecName: Full=Actin-1/3; Flags: Precursor; - OS=Caenorhabditis elegans.
ACT1_DROME Mass: 41795 Score: 157 Matches: 6(5) Sequences: 3(3)
RecName: Full=Actin-5C; Flags: Precursor; - OS=Drosophila melanogaster (Fruit fly).
ACTB1_TAKRU Mass: 41740 Score: 157 Matches: 6(5) Sequences: 3(3)
RecName: Full=Actin, cytoplasmic 1; AltName: Full=Beta-actin A; Contains: RecName: Full=Actin, cytoplasmic 1, N-terminally processed; -
ACT2_CAEEL Mass: 41751 Score: 157 Matches: 6(5) Sequences: 3(3)
RecName: Full=Actin-2; Flags: Precursor; - OS=Caenorhabditis elegans.
ACT2_MOLOC Mass: 42236 Score: 157 Matches: 6(5) Sequences: 3(3)
RecName: Full=Actin, muscle-type; AltName: Full=A2; - OS=Molgula oculata (Sea squirt).
ACT2_ONCVO Mass: 41792 Score: 157 Matches: 6(5) Sequences: 3(3)
RecName: Full=Actin-2; Flags: Precursor; - OS=Onchocerca volvulus.
ACT3A_HELAM Mass: 41806 Score: 157 Matches: 6(5) Sequences: 3(3)
RecName: Full=Actin, cytoplasmic A3a; Flags: Precursor; - OS=Helicoverpa armigera (Cotton bollworm) (Heliothis armigera).
ACT3B_HELAM Mass: 41795 Score: 157 Matches: 6(5) Sequences: 3(3)
RecName: Full=Actin-A3b, cytoplasmic; Flags: Precursor; - OS=Helicoverpa armigera (Cotton bollworm) (Heliothis armigera).
ACT3_LIMPO Mass: 41781 Score: 157 Matches: 6(5) Sequences: 3(3)
RecName: Full=Actin-3; Flags: Precursor; - OS=Limulus polyphemus (Atlantic horseshoe crab).
ACTB3_TAKRU Mass: 41756 Score: 157 Matches: 6(5) Sequences: 3(3)
RecName: Full=Actin, cytoplasmic 3; AltName: Full=Beta-actin C; Contains: RecName: Full=Actin, cytoplasmic 3, N-terminally processed; -
ACT4_ARTSX Mass: 41811 Score: 157 Matches: 6(5) Sequences: 3(3)
RecName: Full=Actin, clone 403; Flags: Precursor; - OS=Artemia sp. (Brine shrimp).
ACT4_BOMMO Mass: 41795 Score: 157 Matches: 6(5) Sequences: 3(3)
RecName: Full=Actin, cytoplasmic A4; Flags: Precursor; - OS=Bombyx mori (Silk moth).
ACT4_CAEEL Mass: 41751 Score: 157 Matches: 6(5) Sequences: 3(3)
RecName: Full=Actin-4; Flags: Precursor; - OS=Caenorhabditis elegans.
ACT5C_ANOGA Mass: 41795 Score: 157 Matches: 6(5) Sequences: 3(3)
RecName: Full=Actin-5C; AltName: Full=Actin-1D, cytoplasmic; Flags: Precursor; - OS=Anopheles gambiae (African malaria mosquito).
ACT5_CHICK Mass: 41809 Score: 157 Matches: 6(5) Sequences: 3(3)
RecName: Full=Actin, cytoplasmic type 5; - OS=Gallus gallus (Chicken).
ACT5_XENLA Mass: 41823 Score: 157 Matches: 6(5) Sequences: 3(3)
RecName: Full=Actin, cytoplasmic type 5; - OS=Xenopus laevis (African clawed frog).
ACT8_XENLA Mass: 41821 Score: 157 Matches: 6(5) Sequences: 3(3)
RecName: Full=Actin, cytoplasmic type 8; - OS=Xenopus laevis (African clawed frog).

---

18. FINC_BOVIN **Mass:** 271983 **Score:** 157 **Matches:** 2(2) **Sequences:** 2(2) **emPAI:** 0.02
RecName: Full=Fibronectin; Short=FN; Contains: RecName: Full=Anastellin; Flags: Precursor; - OS=Bos taurus (Bovine).
☐ Check to include this hit in error tolerant search or archive report

	Query	Observed	Mr(expt)	Mr(calc)	ppm	Miss	Score	Expect	Rank	Unique	Retention Time	Peptide
☑	14	690.3549	1378.6952	1378.6980	-2.03	0	65	0.00041	1	U	829s (13.82m)	K.GLAFTDVDVDSIK.I
☑	21	772.3847	1542.7549	1542.7566	-1.10	0	92	4.5e-07	1	U	662s (11.03m)	R.SYTITGLQPGTDYK.I

Proteins matching the same set of peptides:
FINC_HUMAN **Mass:** 262460 **Score:** 157 **Matches:** 2(2) **Sequences:** 2(2)

RecName: Full=Fibronectin; Short=FN; AltName: Full=Cold-insoluble globulin; Short=CIG; Contains: RecName: Full=Anastellin; Contains: Rec
FINC_MOUSE **Mass:** 272368 **Score:** 157 **Matches:** 2(2) **Sequences:** 2(2)
RecName: Full=Fibronectin; Short=FN; Contains: RecName: Full=Anastellin; Flags: Precursor; - OS=Mus musculus (Mouse).
FINC_RAT **Mass:** 272341 **Score:** 157 **Matches:** 2(2) **Sequences:** 2(2)
RecName: Full=Fibronectin; Short=FN; Contains: RecName: Full=Anastellin; Flags: Precursor; - OS=Rattus norvegicus (Rat).

---

19. K1C3_XENLA **Mass:** 29888 **Score:** 156 **Matches:** 4(2) **Sequences:** 3(2) **emPAI:** 0.24
RecName: Full=Keratin, type I cytoskeletal 47 kDa; Flags: Fragment; - OS=Xenopus laevis (African clawed frog).
☐ Check to include this hit in error tolerant search or archive report

	Query	Observed	Mr(expt)	Mr(calc)	ppm	Miss	Score	Expect	Rank	Unique	Retention Time	Peptide
☑	103	**515.2949**	**1028.5752**	**1028.5866**	**-11.11**	**0**	**53**	**0.0019**	**1**		**711s (11.85m)**	**R.VLDELTLAR.G**
	160	601.3070	1200.5995	1200.6098	-8.63	0	77	5e-06	1		612s (10.20m)	R.QSVEADINGLR.R
	13	679.3589	1356.7033	1356.7110	-5.65	1	(14)	39	1		572s (9.53m)	R.QSVEADINGLRR.V
	224	679.3609	1356.7073	1356.7110	-2.72	1	26	0.97	1		568s (9.47m)	R.QSVEADINGLRR.V

---

20. H4_ACRAS **Mass:** 11374 **Score:** 149 **Matches:** 4(2) **Sequences:** 3(2) **emPAI:** 0.70
RecName: Full=Histone H4; - OS=Acrolepiopsis assectella (Leek moth).
☐ Check to include this hit in error tolerant search or archive report

	Query	Observed	Mr(expt)	Mr(calc)	ppm	Miss	Score	Expect	Rank	Unique	Retention Time	Peptide
☑	88	**495.2884**	**988.5622**	**988.5706**	**-8.43**	**0**	**31**	**0.31**	**1**	**U**	**804s (13.40m)**	**K.VFLENVIR.D**
☑	202	**655.8514**	**1309.6883**	**1309.6952**	**-5.24**	**0**	**54**	**0.0013**	**1**	**U**	**971s (16.18m)**	**K.TVTAMDVVYALK.R**
☑	209	**663.3782**	**1324.7418**	**1324.7463**	**-3.40**	**0**	**64**	**9.4e-05**	**1**	**U**	**557s (9.28m)**	**R.DNIQGITKPAIR.R**
☑	210	**663.3782**	**1324.7418**	**1324.7463**	**-3.40**	**0**	**(35)**	**0.061**	**1**	**U**	**561s (9.35m)**	**R.DNIQGITKPAIR.R**

Proteins matching the same set of peptides:
H4_ACRFO **Mass:** 11374 **Score:** 149 **Matches:** 4(2) **Sequences:** 3(2)
RecName: Full=Histone H4; - OS=Acropora formosa (Staghorn coral).
H4_APLCA **Mass:** 11307 **Score:** 149 **Matches:** 4(2) **Sequences:** 3(2)
RecName: Full=Histone H4; - OS=Aplysia californica (California sea hare).
H4_ASCSU **Mass:** 11373 **Score:** 149 **Matches:** 4(2) **Sequences:** 3(2)
RecName: Full=Histone H4; - OS=Ascaris suum (Pig roundworm) (Ascaris lumbricoides).
H4_ASEAQ **Mass:** 11374 **Score:** 149 **Matches:** 4(2) **Sequences:** 3(2)
RecName: Full=Histone H4; - OS=Asellus aquaticus (Water hoglouse).
H4_BOVIN **Mass:** 11360 **Score:** 149 **Matches:** 4(2) **Sequences:** 3(2)
RecName: Full=Histone H4; AltName: Full=H4.1; - OS=Bos taurus (Bovine).
H4_CAEEL **Mass:** 11362 **Score:** 149 **Matches:** 4(2) **Sequences:** 3(2)
RecName: Full=Histone H4; - OS=Caenorhabditis elegans.
H4_CAIMO **Mass:** 11360 **Score:** 149 **Matches:** 4(2) **Sequences:** 3(2)
RecName: Full=Histone H4; - OS=Cairina moschata (Muscovy duck).
H4_CHAVR **Mass:** 11360 **Score:** 149 **Matches:** 4(2) **Sequences:** 3(2)
RecName: Full=Histone H4; - OS=Chaetopterus variopedatus (Parchment worm).
H4_CHICK **Mass:** 11360 **Score:** 149 **Matches:** 4(2) **Sequences:** 3(2)
RecName: Full=Histone H4; - OS=Gallus gallus (Chicken).
H4_CHITH **Mass:** 11374 **Score:** 149 **Matches:** 4(2) **Sequences:** 3(2)
RecName: Full=Histone H4; - OS=Chironomus thummi thummi (Midge).
H4_DENKL **Mass:** 11374 **Score:** 149 **Matches:** 4(2) **Sequences:** 3(2)
RecName: Full=Histone H4; - OS=Dendronephthya klunzingeri (Klunzinger's soft coral).
H4_DROER **Mass:** 11374 **Score:** 149 **Matches:** 4(2) **Sequences:** 3(2)
RecName: Full=Histone H4; - OS=Drosophila erecta (Fruit fly).
H4_DROHY **Mass:** 11374 **Score:** 149 **Matches:** 4(2) **Sequences:** 3(2)
RecName: Full=Histone H4; - OS=Drosophila hydei (Fruit fly).
H4_DROMA **Mass:** 11374 **Score:** 149 **Matches:** 4(2) **Sequences:** 3(2)
RecName: Full=Histone H4; - OS=Drosophila mauritiana (Fruit fly).
H4_DROME **Mass:** 11374 **Score:** 149 **Matches:** 4(2) **Sequences:** 3(2)
RecName: Full=Histone H4; - OS=Drosophila melanogaster (Fruit fly).
H4_DROOR **Mass:** 11374 **Score:** 149 **Matches:** 4(2) **Sequences:** 3(2)
RecName: Full=Histone H4; - OS=Drosophila orena (Fruit fly).
H4_DROSE **Mass:** 11374 **Score:** 149 **Matches:** 4(2) **Sequences:** 3(2)
RecName: Full=Histone H4; - OS=Drosophila sechellia (Fruit fly).
H4_DROSI **Mass:** 11374 **Score:** 149 **Matches:** 4(2) **Sequences:** 3(2)
RecName: Full=Histone H4; - OS=Drosophila simulans (Fruit fly).
H4_DROTE **Mass:** 11374 **Score:** 149 **Matches:** 4(2) **Sequences:** 3(2)
RecName: Full=Histone H4; - OS=Drosophila teissieri (Fruit fly).
H4_DROYA **Mass:** 11374 **Score:** 149 **Matches:** 4(2) **Sequences:** 3(2)
RecName: Full=Histone H4; - OS=Drosophila yakuba (Fruit fly).
H4_HOLTU **Mass:** 11362 **Score:** 149 **Matches:** 4(2) **Sequences:** 3(2)
RecName: Full=Histone H4; - OS=Holothuria tubulosa (Sea cucumber).
H4_HUMAN **Mass:** 11360 **Score:** 149 **Matches:** 4(2) **Sequences:** 3(2)
RecName: Full=Histone H4; - OS=Homo sapiens (Human).
H4_LITVA **Mass:** 11366 **Score:** 149 **Matches:** 4(2) **Sequences:** 3(2)
RecName: Full=Histone H4; - OS=Litopenaeus vannamei (Whiteleg shrimp) (Penaeus vannamei).
H4_LYTPI **Mass:** 11362 **Score:** 149 **Matches:** 4(2) **Sequences:** 3(2)
RecName: Full=Histone H4; - OS=Lytechinus pictus (Painted sea urchin).
H4_MACFA **Mass:** 11360 **Score:** 149 **Matches:** 4(2) **Sequences:** 3(2)
RecName: Full=Histone H4; - OS=Macaca fascicularis (Crab-eating macaque) (Cynomolgus monkey).
H4_MASBA **Mass:** 11903 **Score:** 149 **Matches:** 4(2) **Sequences:** 3(2)
RecName: Full=Histone H4; - OS=Mastigamoeba balamuthi (Phreatamoeba balamuthi).
H4_MOUSE **Mass:** 11360 **Score:** 149 **Matches:** 4(2) **Sequences:** 3(2)
RecName: Full=Histone H4; - OS=Mus musculus (Mouse).
H4_MYRRU **Mass:** 11374 **Score:** 149 **Matches:** 4(2) **Sequences:** 3(2)
RecName: Full=Histone H4; - OS=Myrmica ruginodis (Red ant).

H4_MYTCA Mass: 11360 Score: 149 Matches: 4(2) Sequences: 3(2)
RecName: Full=Histone H4; - OS=Mytilus californianus (California mussel).
H4_MYTCH Mass: 11388 Score: 149 Matches: 4(2) Sequences: 3(2)
RecName: Full=Histone H4; - OS=Mytilus chilensis (Chilean blue mussel).
H4_MYTED Mass: 11360 Score: 149 Matches: 4(2) Sequences: 3(2)
RecName: Full=Histone H4; - OS=Mytilus edulis (Blue mussel).
H4_MYTGA Mass: 11360 Score: 149 Matches: 4(2) Sequences: 3(2)
RecName: Full=Histone H4; - OS=Mytilus galloprovincialis (Mediterranean mussel).
H4_MYTTR Mass: 11360 Score: 149 Matches: 4(2) Sequences: 3(2)
RecName: Full=Histone H4; - OS=Mytilus trossulus (Blue mussel).
H4_OLILU Mass: 11416 Score: 149 Matches: 4(2) Sequences: 3(2)
RecName: Full=Histone H4; - OS=Olisthodiscus luteus (Marine phytoflagellate) (Heterosigma akashiwo).
H4_ONCMY Mass: 11360 Score: 149 Matches: 4(2) Sequences: 3(2)
RecName: Full=Histone H4; - OS=Oncorhynchus mykiss (Rainbow trout) (Salmo gairdneri).
H4_ORENI Mass: 11360 Score: 149 Matches: 4(2) Sequences: 3(2)
RecName: Full=Histone H4; - OS=Oreochromis niloticus (Nile tilapia) (Tilapia nilotica).
H4_PARLI Mass: 11362 Score: 149 Matches: 4(2) Sequences: 3(2)
RecName: Full=Histone H4; - OS=Paracentrotus lividus (Common sea urchin).
H4_PIG Mass: 11360 Score: 149 Matches: 4(2) Sequences: 3(2)
RecName: Full=Histone H4; - OS=Sus scrofa (Pig).
H4_PISBR Mass: 11362 Score: 149 Matches: 4(2) Sequences: 3(2)
RecName: Full=Histone H4; - OS=Pisaster brevispinus (Short spined sea star) (Asterias brevispina).
H4_PISOC Mass: 11362 Score: 149 Matches: 4(2) Sequences: 3(2)
RecName: Full=Histone H4; - OS=Pisaster ochraceus (Ochre sea star) (Asterias ochracea).
H4_PLADU Mass: 11360 Score: 149 Matches: 4(2) Sequences: 3(2)
RecName: Full=Histone H4; - OS=Platynereis dumerilii (Dumeril's clam worm).
H4_PONAB Mass: 11360 Score: 149 Matches: 4(2) Sequences: 3(2)
RecName: Full=Histone H4; - OS=Pongo abelii (Sumatran orangutan) (Pongo pygmaeus abelii).
H4_PSAMI Mass: 11362 Score: 149 Matches: 4(2) Sequences: 3(2)
RecName: Full=Histone H4; - OS=Psammechinus miliaris (Green sea urchin) (Echinus miliaris).
H4_PYCHE Mass: 11362 Score: 149 Matches: 4(2) Sequences: 3(2)
RecName: Full=Histone H4; - OS=Pycnopodia helianthoides (Sunflower sea star).
H4_PYRSA Mass: 11418 Score: 149 Matches: 4(2) Sequences: 3(2)
RecName: Full=Histone H4; - OS=Pyrenomonas salina.
H4_RAT Mass: 11360 Score: 149 Matches: 4(2) Sequences: 3(2)
RecName: Full=Histone H4; Contains: RecName: Full=Osteogenic growth peptide; Short=OGP; - OS=Rattus norvegicus (Rat).
H4_RHYAM Mass: 11374 Score: 149 Matches: 4(2) Sequences: 3(2)
RecName: Full=Histone H4; - OS=Rhynchosciara americana (Fungus gnat).
H4_STRPU Mass: 11362 Score: 149 Matches: 4(2) Sequences: 3(2)
RecName: Full=Histone H4; - OS=Strongylocentrotus purpuratus (Purple sea urchin).
H4_TIGCA Mass: 11374 Score: 149 Matches: 4(2) Sequences: 3(2)
RecName: Full=Histone H4; - OS=Tigriopus californicus (Marine copepod).
H4_URECA Mass: 11360 Score: 149 Matches: 4(2) Sequences: 3(2)
RecName: Full=Histone H4; - OS=Urechis caupo (Innkeeper worm) (Spoonworm).
H4_XENBO Mass: 11360 Score: 149 Matches: 4(2) Sequences: 3(2)
RecName: Full=Histone H4; - OS=Xenopus borealis (Kenyan clawed frog).
H4_XENLA Mass: 11360 Score: 149 Matches: 4(2) Sequences: 3(2)
RecName: Full=Histone H4; - OS=Xenopus laevis (African clawed frog).
H4_XENTR Mass: 11360 Score: 149 Matches: 4(2) Sequences: 3(2)
RecName: Full=Histone H4; - OS=Xenopus tropicalis (Western clawed frog) (Silurana tropicalis).
H48_CHICK Mass: 11432 Score: 149 Matches: 4(2) Sequences: 3(2)
RecName: Full=Histone H4 type VIII; - OS=Gallus gallus (Chicken).

Peptide matches not assigned to protein hits: (no details means no match)

	Query	Observed	Mr(expt)	Mr(calc)	ppm	Miss	Score	Expect	Rank	Unique	Retention Time	Peptide
☑	330	890.9176	1779.8205	1779.8275	-3.91	0	140	1.6e-12	1		628s (10.47m)	VDATEESDLAQQYGVR
☑	283	797.4031	1592.7917	1592.8046	-8.11	0	81	3e-06	1		1019s (16.98m)	SVLGDVGITEVFSDR
☑	345	615.6323	1843.8751	1843.8840	-4.83	1	80	2.7e-06	1		749s (12.48m)	EAFSLFDKDGDGTITTK
☑	286	533.5815	1597.7226	1597.7332	-6.66	0	73	8.5e-06	1		491s (8.18m)	AVTEQGHELSNEER
☑	109	523.2814	1044.5482	1044.5564	-7.83	0	72	3e-05	1		514s (8.57m)	LSSPATLNSR
☑	207	662.3108	1322.6071	1322.6103	-2.41	0	71	1.6e-05	1		652s (10.87m)	SAGDVDTLAFDGR
☑	311	850.9132	1699.8119	1699.8206	-5.08	0	68	3.9e-05	1		881s (14.68m)	EGVYTVFAPTNEAFR
☑	240	710.3738	1418.7330	1418.7405	-5.27	0	68	7.4e-05	1		1008s (16.80m)	LEGLTDEINFLR
☑	22	776.3603	1550.7061	1550.7212	-9.77	0	67	7.4e-05	1		592s (9.87m)	SIEYSPQLEDAGSR
☑	73	472.7676	943.5207	943.5240	-3.48	0	63	0.00025	1		707s (11.78m)	AGIQFPVGR
☑	128	571.3032	1140.5918	1140.6026	-9.47	0	62	0.00015	1		698s (11.63m)	GDLGIEIPAEK
☑	287	806.4061	1610.7976	1610.8039	-3.93	0	62	0.0002	1		714s (11.90m)	TVEIPDPVEAGEEVK
☑	155	599.3268	1196.6391	1196.6401	-0.80	0	62	0.00017	1		659s (10.98m)	LDIDSPPITAR
☑	147	593.8250	1185.6354	1185.6394	-3.38	0	60	0.00044	1		745s (12.42m)	GPSVFPLAPSSK
☑	7	523.2804	1044.5463	1044.5564	-9.63	0	60	0.00094	1		518s (8.63m)	LSSPATLNSR
☑	38	421.7560	841.4975	841.5022	-5.52	0	58	0.00046	1		547s (9.12m)	VATVSLPR
☑	40	421.7561	841.4975	841.5022	-5.47	0	58	0.00047	1		637s (10.62m)	VATVSLPR
☑	236	705.8144	1409.6143	1409.6212	-4.85	0	58	0.00014	1		705s (11.75m)	EQFLDGDGWTSR
☑	72	472.7676	943.5207	943.5240	-3.48	0	57	0.0011	1		710s (11.83m)	AGIQFPVGR
☑	39	421.7561	841.4975	841.5022	-5.47	0	57	0.0007	1		640s (10.67m)	VATVSLPR
☑	241	717.8668	1433.7190	1433.7191	-0.00	0	56	0.00098	1		620s (10.33m)	PYQYPALTPEQK
☑	37	421.7560	841.4975	841.5022	-5.52	0	55	0.0011	1		544s (9.07m)	VATVSLPR
☑	318	869.4530	1736.8915	1736.8985	-4.01	0	55	0.0013	1		1802s (30.03m)	AGTDLLNFLSSFIDPK
☑	41	421.7567	841.4988	841.5022	-4.02	0	55	0.0011	1		751s (12.52m)	VATVSLPR
☑	132	577.8086	1153.6027	1153.6091	-5.59	0	54	0.0011	1		511s (8.52m)	HTLNQIDSVK
☑	169	610.8040	1219.5934	1219.5986	-4.23	0	54	0.0013	1		778s (12.97m)	NFPSPVDAAFR
☑	164	604.8016	1207.5887	1207.5972	-7.04	0	54	0.0014	1		689s (11.48m)	VEVTEFEDIK
☑	168	610.8040	1219.5934	1219.5986	-4.23	0	49	0.004	1		775s (12.92m)	NFPSPVDAAFR
☑	77	477.3029	952.5912	952.5957	-4.72	0	46	0.0017	1		788s (13.13m)	LILPGELAK

☑	76	477.3029	952.5912	952.5957	-4.72	0	45	0.0022	1	792s (13.20m)	LILPGELAK
☑	1	390.2013	1167.5821	1167.5884	-5.38	0	41	0.049	1	487s (8.12m)	QVHPDTGISSK
☑	266	508.9444	1523.8114	1523.8308	-12.73	1	41	0.024	1	503s (8.38m)	TKEGVVHGVATVAEK
☑	157	599.8335	1197.6524	1197.6605	-6.69	1	32	0.21	1	531s (8.85m)	KLEENEVIPK
☑	48	430.2191	858.4236	858.4276	-4.65	0	31	0.16	1	697s (11.62m)	GFFYTPK
☑	234	703.8597	1405.7049	1405.6983	4.65	2	31	0.32	1	1422s (23.70m)	GDKDSMEIARLR + Oxidation (M)
☑	106	516.7986	1031.5827	1031.5876	-4.70	0	30	0.39	1	528s (8.80m)	YRPGTVALR
☑	33	412.7507	823.4869	823.5028	-19.31	0	28	0.21	1	581s (9.68m)	RPVSLPR
☑	183	634.8401	1267.6656	1267.6673	-1.36	1	26	0.79	1	545s (9.08m)	FDPVRGEVPPR
☑	32	412.7507	823.4869	823.5028	-19.31	0	25	0.41	1	583s (9.72m)	RPVSLPR
☑	11	600.8525	1199.6905	1199.6986	-6.72	1	25	1.3	1	588s (9.80m)	SATRITVSLPR
☑	54	435.7706	869.5267	869.5334	-7.77	0	23	0.75	1	604s (10.07m)	VVSLSIPR
☑	42	422.7527	843.4909	843.4814	11.3	1	23	3	1	522s (8.70m)	KDLINNK
☑	53	435.7706	869.5267	869.5334	-7.77	0	23	0.87	1	601s (10.02m)	VVSLSIPR
☑	93	502.2892	1002.5638	1002.5710	-7.10	1	23	2.2	1	590s (9.83m)	VDVTGISKGK
☑	344	921.9558	1841.8970	1841.9087	-6.34	0	22	1.9	1	1096s (18.27m)	EAGIPEFYDYDVALIK
☑	98	509.2950	1016.5754	1016.5655	9.79	0	22	2.5	1	648s (10.80m)	VAPIQGFSAK
☑	271	776.8341	1551.6536	1551.6776	-15.49	0	22	0.32	1	760s (12.67m)	HDMGGDIAPYFASR + Oxidation (M)
☑	27	401.2605	800.5065	800.5007	7.17	0	21	1.5	1	664s (11.07m)	LVDITLK
☑	205	659.3677	1316.7208	1316.7122	6.53	0	20	3.5	1	1162s (19.37m)	TLDLAMANSLLR
☑	149	594.8389	1187.6632	1187.6808	-14.87	2	20	3.9	1	826s (13.77m)	RAGACVSVVAKK
☑	24	393.7432	785.4719	785.4759	-5.18	0	20	5.6	1	539s (8.98m)	VATQIVR
☑	51	435.7544	869.4943	869.4971	-3.20	0	20	1.8	1	731s (12.18m)	VVTDLAPR
☑	52	435.7544	869.4943	869.4971	-3.20	0	20	1.8	1	733s (12.22m)	VVTDLAPR
☑	156	599.8335	1197.6524	1197.6605	-6.69	1	20	3.1	1	533s (8.88m)	KLEENEVIPK
☑	110	523.2817	1044.5487	1044.5564	-7.28	0	19	5.5	1	655s (10.92m)	LSSPATLNSR
☑	272	776.8341	1551.6536	1551.6776	-15.49	0	19	0.53	1	756s (12.60m)	HDMGGDIAPYFASR + Oxidation (M)
☑	112	523.7784	1045.5422	1045.5404	1.75	1	19	5.3	1	618s (10.30m)	LREGTLDDK
☑	317	866.3947	1730.7748	1730.8079	-19.13	1	19	1.3	1	1248s (20.80m)	GKMHSSEEMAEILNR
☑	159	400.5538	1198.6395	1198.6306	7.41	0	19	4.1	1	527s (8.78m)	QGSDQVIPLSR
☑	117	1078.0847	2154.1549	2154.1255	13.6	0	19	4.1	1	1175s (19.58m)	INTFGNDTNIIHNIKPCLK
☑	124	558.8235	1115.6325	1115.6186	12.4	1	19	5.1	1	584s (9.73m)	EVDKTVAVAGK
☑	140	588.8132	1175.6119	1175.6186	-5.71	0	19	6.3	1	818s (13.63m)	TLNYINPDVK
☑	17	383.1974	764.3803	764.3857	-7.14	0	18	5	1	673s (11.22m)	GEFVWK
☑	336	897.9066	1793.7986	1793.7924	3.49	0	18	1.5	1	809s (13.48m)	DGMQEEAIQEIAGMTR + Oxidation (M)
☑	8	571.3473	570.3400	570.3489	-15.65	0	18	7	1	587s (9.78m)	VSIPR
☑	125	559.3272	1116.6398	1116.6251	13.1	1	17	5.6	1	587s (9.78m)	GIVTTVRDTR
☑	96	507.7674	1013.5202	1013.5254	-5.09	1	17	4.1	1	740s (12.33m)	DGVSDRLPR
☑	177	622.3464	1242.6783	1242.6932	-12.00	1	17	9.6	1	728s (12.13m)	SVLLDEKAVNR
☑	191	644.8095	1287.6045	1287.6104	-4.60	0	17	4	1	708s (11.80m)	MGFGFISMINR + Oxidation (M)
☑	196	650.3729	1298.7313	1298.7419	-8.17	1	17	8.4	1	695s (11.58m)	ATGRATQGVIGIR
☑	143	590.8525	1179.6905	1179.6910	-0.46	0	16	3	1	1452s (24.20m)	AARPPVIGMIR
☑	131	576.2811	1150.5476	1150.5441	3.03	0	16	6.8	1	514s (8.57m)	AFMQSLHSSK + Oxidation (M)
☑	63	457.7658	913.5169	913.5094	8.30	1	16	12	1	617s (10.28m)	VATGRTGPR
☑	120	551.8199	1101.6252	1101.6328	-6.94	1	16	10	1	587s (9.78m)	MGKNTVIALR
☑	185	638.8266	1275.6387	1275.6571	-14.45	2	15	12	1	597s (9.95m)	ASRKWSDADIK
☑	92	502.2892	1002.5638	1002.5710	-7.10	1	15	13	1	586s (9.77m)	VDVTGISKGK
☑	204	658.8759	1315.7372	1315.7388	-1.16	0	15	13	1	1165s (19.42m)	DVDAILFVVPTK
☑	56	443.2305	884.4464	884.4352	12.7	0	15	5.1	1	661s (11.02m)	AEGGELGPR
☑	148	594.8360	1187.6574	1187.6485	7.47	0	14	16	1	585s (9.75m)	FMGKPGVLSPR
☑	118	543.8165	1085.6184	1085.6015	15.5	1	14	16	1	587s (9.78m)	INKCGVISPR
☑	273	782.3751	1562.7357	1562.7536	-11.44	1	14	10	1	830s (13.83m)	LLENTERSEETSR
☑	136	581.3374	1160.6603	1160.6513	7.74	2	14	13	1	584s (9.73m)	DRISTVKNTK
☑	217	667.3654	1332.7162	1332.7071	6.86	0	14	18	1	1021s (17.02m)	TLDLAMANSLLR + Oxidation (M)
☑	363	514.7552	2054.9915	2054.9653	12.8	0	14	14	1	498s (8.30m)	GSTMPMDGYISGLLNDIQK + Oxidation (M)
☑	9	588.8333	1175.6520	1175.6332	16.0	0	13	18	1	588s (9.80m)	AVTVTSLQAMR
☑	119	543.8165	1085.6184	1085.6056	11.8	0	13	20	1	583s (9.72m)	IMTAVWIPR
☑	145	591.8477	1181.6809	1181.6591	18.5	0	13	9.2	1	584s (9.73m)	MQLAIVAVGHK + Oxidation (M)
☑	368	713.3948	2137.1625	2137.1929	-14.21	2	13	13	1	1402s (23.37m)	DACLVANVLDPRIKQEIIK
☑	142	590.3513	1178.6880	1178.7097	-18.36	1	13	8.3	1	1455s (24.25m)	VFTKLLMIAK + Oxidation (M)
☑	376	1100.0712	2198.1278	2198.1513	-10.68	0	13	20	1	712s (11.87m)	LMGLVLIMLSTQMFLDGIR + 3 Oxidation (M)
☑	186	639.3003	1276.5860	1276.5791	5.39	0	12	8.3	1	828s (13.80m)	INGMPEVQDMK + Oxidation (M)
☑	298	827.4203	1652.8261	1652.7940	19.4	0	12	20	1	996s (16.60m)	LHQSISQMTGEAHAK + Oxidation (M)
☑	219	671.8418	1341.6689	1341.6749	-4.46	1	12	17	1	594s (9.90m)	GAAVQGPRTQSDR
☑	144	591.8477	1181.6809	1181.6768	3.44	0	12	12	1	588s (9.80m)	GITLPGAVDAIR
☑	331	890.9320	1779.8494	1779.8751	-14.44	2	12	18	1	786s (13.10m)	RFSDVDEDISRQSVK
☑	121	551.8199	1101.6252	1101.6328	-6.94	1	12	25	1	583s (9.72m)	MGKNTVIALR
☑	259	754.8754	1507.7361	1507.7188	11.5	2	12	23	1	640s (10.67m)	KSIMQEEEKNEK + Oxidation (M)
☑	201	654.3043	1306.5941	1306.6194	-19.30	0	12	9.9	1	529s (8.82m)	TWFNPADVETK
☑	5	478.2831	954.5517	954.5498	2.00	0	11	25	1	701s (11.68m)	IVDGVPLSR
☑	10	588.8334	1175.6523	1175.6299	19.1	1	11	29	1	584s (9.73m)	ASLLWRDTSK
☑	67	462.7933	923.5721	923.5552	18.3	0	11	4.4	1	1062s (17.70m)	AQPGVLLAR
☑	158	599.8446	1197.6747	1197.6830	-6.87	1	11	24	1	585s (9.75m)	LAGDLTRISPR
☑	129	571.8315	1141.6484	1141.6680	-17.12	2	11	31	1	591s (9.85m)	KRQASTAKPR
☑	107	517.2944	1032.5743	1032.5815	-7.00	0	11	31	1	607s (10.12m)	SLASVLTTNK
☑	165	608.3497	1214.6849	1214.6871	-1.76	0	10	41	1	588s (9.80m)	GDTVVTIGGIVGK
☑	85	492.7470	983.4794	983.4672	12.4	0	10	17	1	593s (9.88m)	EQAVHGESK
☑	239	708.8474	1415.6803	1415.7045	-17.06	0	10	26	1	834s (13.90m)	YHPIDIETSVSR
☑	257	752.8631	1503.7116	1503.6841	18.3	0	10	23	1	856s (14.27m)	VENSEPPFETEAR
☑	208	442.2265	1323.6578	1323.6825	-18.64	2	10	35	1	669s (11.15m)	IISRCQVCMKK + Oxidation (M)
☑	326	881.9211	1761.8276	1761.7952	18.4	0	10	26	1	770s (12.83m)	MTVTPSGSNGAGSAAPEGR + Oxidation (M)
☑	101	512.2375	1022.4604	1022.4637	-3.21	0	10	20	1	549s (9.15m)	AAQMMGINR + 2 Oxidation (M)
☑	163	603.8332	1205.6519	1205.6517	0.18	1	9	42	1	585s (9.75m)	TAVAEGFVRTR
☑	348	927.9504	1853.8863	1853.8573	15.7	1	9	31	1	1116s (18.60m)	ETMVLVCGPEAMEKASK + 2 Oxidation (M)
☑	359	983.9763	1965.9381	1965.9465	-4.31	1	9	32	1	874s (14.57m)	FGTEELARIEGEMLEAR + Oxidation (M)
☑	3	466.2422	930.4698	930.4658	4.30	0	9	93	1	886s (14.77m)	DPEAASTIK

☑	258	752.8631	1503.7116	1503.7174	-3.80	1	9	28	1	860s (14.33m)	MDRSIMPIDSPAR + Oxidation (M)
☑	150	596.3146	1190.6146	1190.6118	2.43	1	9	53	1	1201s (20.02m)	AKMFTEIHAK + Oxidation (M)
☑	250	733.8427	1465.6709	1465.6541	11.5	0	9	20	1	559s (9.32m)	EIMNVVACEGTER + Oxidation (M)
☑	274	782.3751	1562.7357	1562.7536	-11.44	1	9	31	1	832s (13.87m)	LLENTERSEETSR
☑	4	466.2425	930.4705	930.4559	15.7	0	9	97	1	888s (14.80m)	DLAAAEWR
☑	212	663.8274	1325.6402	1325.6332	5.26	1	9	33	1	534s (8.90m)	QLMYCVRQNR + Oxidation (M)
☑	276	783.8713	1565.7279	1565.7461	-11.58	1	9	28	1	631s (10.52m)	IDDEDTVFEIDKK
☑	43	425.7581	849.5016	849.4960	6.62	0	9	25	1	483s (8.05m)	QTLIFTK
☑	151	596.3222	1190.6298	1190.6295	0.20	1	9	59	1	821s (13.68m)	GFLQDLKQDK
☑	65	458.7854	915.5563	915.5389	19.0	1	9	33	1	936s (15.60m)	ELTNAKIK
☑	383	858.7765	2573.3078	2573.3013	2.50	1	9	48	1	702s (11.70m)	VDTILEKDAYFPGEEVQGTVHVK
☑	114	1070.0825	2138.1505	2138.1809	-14.21	2	9	43	1	1392s (23.20m)	KVLDMYINYEIIVKEIR
☑	146	592.8660	1183.7175	1183.7037	11.7	2	9	27	1	691s (11.52m)	LLRRLDAEAK
☑	289	813.4395	1624.8645	1624.8461	11.4	1	9	45	1	1140s (19.00m)	DLINIKLDFYDTR
☑	100	512.2375	1022.4604	1022.4637	-3.21	0	8	25	1	552s (9.20m)	AAQMMGINR + 2 Oxidation (M)
☑	335	897.9066	1793.7986	1793.7924	3.49	0	8	14	1	805s (13.42m)	DGMQEEAIQEIAGMTR + Oxidation (M)
☑	122	557.8339	1113.6533	1113.6618	-7.64	1	8	48	1	590s (9.83m)	NRTVTAALLR
☑	187	639.3003	1276.5860	1276.6081	-17.32	1	8	23	1	831s (13.85m)	RALSDAGMTPDK + Oxidation (M)
☑	19	384.2330	766.4514	766.4450	8.41	0	8	12	1	845s (14.08m)	THQLIR
☑	249	733.8427	1465.6709	1465.6725	-1.08	0	8	26	1	562s (9.37m)	EVQEWVFSEGEK
☑	265	762.8482	1523.6818	1523.6748	4.60	0	8	21	1	627s (10.45m)	EGHMMDALYAQVK + 2 Oxidation (M)
☑	248	731.8356	1461.6566	1461.6843	-19.00	1	8	21	1	1210s (20.17m)	TYTAKMQEMVTK + 2 Oxidation (M)
☑	245	730.3753	1458.7361	1458.7639	-19.07	1	8	68	1	894s (14.90m)	KVPEEIMVEELK + Oxidation (M)
☑	46	428.2587	854.5029	854.4861	19.6	2	8	29	1	907s (15.12m)	TYSTKKK
☑	355	970.9654	1939.9163	1939.9422	-13.31	2	8	40	1	802s (13.37m)	SFSSAVKNELCRVETDR
☑	69	936.4402	1870.8658	1870.8340	17.0	2	8	99	1	753s (12.55m)	GGKHGSEGKHGSGGSSMGGGK + Oxidation (M)
☑	310	849.4434	1696.8722	1696.8970	-14.61	1	7	68	1	696s (11.60m)	NGFIKALYEMVQIR + Oxidation (M)
☑	216	667.3654	1332.7162	1332.7071	6.86	0	7	75	1	1018s (16.97m)	TLDLAMANSLLR + Oxidation (M)
☑	370	719.0587	2154.1543	2154.1532	0.52	1	7	58	1	1177s (19.62m)	GTSLETIIKAVENLEPVEGR
☑	18	384.2330	766.4514	766.4450	8.41	0	7	15	1	848s (14.13m)	THQLIR
☑	256	746.3768	1490.7391	1490.7109	18.9	1	7	70	1	987s (16.45m)	EGMPEIAMDLKNK + Oxidation (M)
☑	340	913.9782	1825.9418	1825.9100	17.4	2	7	74	1	783s (13.05m)	AMVTETMMKLRNELK + 2 Oxidation (M)
☑	192	644.8095	1287.6045	1287.6104	-4.60	0	7	36	1	704s (11.73m)	MGFGFISMINR + Oxidation (M)
☑	99	509.2950	1016.5754	1016.5767	-1.26	2	7	73	1	651s (10.85m)	FHGKVSSKK
☑	235	704.8526	1407.6907	1407.6630	19.7	1	7	74	1	1022s (17.03m)	DDAVGEAYDKVAR
☑	293	816.9532	1631.8918	1631.8804	6.99	1	7	67	1	624s (10.40m)	VKTVVGIEGVEEMVK + Oxidation (M)
☑	228	695.8417	1389.6689	1389.6446	17.5	0	7	59	1	829s (13.82m)	ELGGDAPTMQEVK + Oxidation (M)
☑	381	850.7471	2549.2196	2549.2366	-6.68	1	7	58	1	1013s (16.88m)	MTAAIIDYGSGNLHSAAKALECAAR + Oxidation (M
☑	179	625.3054	1248.5962	1248.5955	0.58	1	7	65	1	578s (9.63m)	QLRMMPGQEK + 2 Oxidation (M)
☑	282	795.8856	1589.7566	1589.7831	-16.70	1	7	54	1	535s (8.92m)	QNSLSGMLDGKATPR + Oxidation (M)
☑	296	825.4113	1648.8080	1648.7991	5.39	0	7	75	1	1167s (19.45m)	MPHTGSQHTLQATPK + Oxidation (M)
☑	36	839.3981	1676.7816	1676.8006	-11.30	1	7	1.3e+02	1	955s (15.92m)	ADAEEGTFEIKTPNR
☑	297	827.4203	1652.8261	1652.8270	-0.58	1	7	76	1	1000s (16.67m)	QPKALSPEEQAHYR
☑	84	490.7546	979.4947	979.5087	-14.30	1	7	77	1	629s (10.48m)	RGYSVDVGK
☑	123	557.8339	1113.6533	1113.6394	12.5	0	7	72	1	582s (9.70m)	IVTETLPSVR
☑	275	783.8713	1565.7279	1565.7429	-9.55	0	6	48	1	635s (10.58m)	QCTNLEALSTLMDK
☑	230	697.8683	1393.7220	1393.7023	14.1	0	6	97	1	558s (9.30m)	EIQNAGMYAILR + Oxidation (M)
☑	354	967.4702	1932.9259	1932.9509	-12.92	2	6	68	1	1069s (17.82m)	KEACADVARQIDLGAAMR + Oxidation (M)
☑	351	952.4293	1902.8440	1902.8566	-6.60	1	6	21	1	763s (12.72m)	DPEMFKEFVNEMMIK + Oxidation (M)
☑	44	428.1732	854.3319	854.3406	-10.18	0	6	6	1	566s (9.43m)	TYEDDGR
☑	90	501.7924	1001.5703	1001.5757	-5.37	1	6	1.3e+02	1	683s (11.38m)	VIEEVSKAK
☑	349	928.4566	1854.8986	1854.8901	4.61	1	6	76	1	1119s (18.65m)	HYAGDVTYDVRGFLDK
☑	47	428.2587	854.5029	854.4861	19.6	2	6	44	1	905s (15.08m)	TYTKSKK
☑	2	413.2622	1236.7648	1236.7594	4.39	2	6	33	1	272s (4.53m)	LLNLFKFSKK
☑	45	428.1732	854.3319	854.3197	14.3	0	6	6.3	1	563s (9.38m)	MNMGGCSR
☑	227	695.8417	1389.6689	1389.6670	1.35	1	6	76	1	831s (13.85m)	KAAGEINPCTSGSR
☑	377	742.3788	2224.1145	2224.1236	-4.10	1	6	1e+02	1	773s (12.88m)	EHVALELNTLGDPAERAAYR
☑	352	952.4293	1902.8440	1902.8566	-6.60	1	6	23	1	767s (12.78m)	DPEMFKEFVNEMMIK + Oxidation (M)
☑	237	705.8288	1409.6430	1409.6310	8.51	0	5	48	1	798s (13.30m)	EDPSSNQETYLK
☑	288	812.8930	1623.7715	1623.7603	6.91	0	5	72	1	869s (14.48m)	QMHYSDDLDFILK
☑	178	624.3336	1246.6526	1246.6306	17.6	1	5	1.3e+02	1	701s (11.68m)	GSDKHIAYAASK
☑	369	1070.0867	2138.1588	2138.1524	3.00	0	5	84	1	1395s (23.25m)	NLLTGGIFVYPPTITHPNGK
☑	302	838.8895	1675.7645	1675.7876	-13.76	0	5	44	1	766s (12.77m)	DNAMHGLVVYDDLSK
☑	87	494.2986	986.5827	986.5985	-15.98	2	5	1.2e+02	1	995s (16.58m)	TRIRVSQK
☑	299	833.3998	1664.7849	1664.7868	-1.14	0	5	75	1	839s (13.98m)	AGLFDVSHMGEFEVK
☑	386	894.1321	2679.3744	2679.4021	-10.31	1	5	1.1e+02	1	1842s (30.70m)	GKHKPVYTPHVDTGDYLVVINAEK
☑	264	762.8482	1523.6818	1523.7038	-14.41	0	5	41	1	631s (10.52m)	EDTHAHLMAENIK + Oxidation (M)
☑	49	430.7195	859.4244	859.4188	6.49	0	5	1.2e+02	1	700s (11.67m)	HNYTTPK
☑	50	432.7450	863.4754	863.4766	-1.37	1	5	1.3e+02	1	586s (9.77m)	RSIFWR
☑	15	713.7236	2138.1489	2138.1513	-1.12	1	5	1e+02	1	1398s (23.30m)	NILLICAAGMSTSLLVTKMK + 2 Oxidation (M)
☑	75	949.9418	1897.8691	1897.8658	1.76	0	5	53	1	890s (14.83m)	IVMLGSMPAGEAGDIMMK + 3 Oxidation (M)
☑	105	516.3119	1030.6092	1030.5957	13.1	1	5	75	1	1020s (17.00m)	TKAVVMLNR
☑	384	876.1343	2625.3811	2625.3974	-6.18	0	5	1e+02	1	1364s (22.73m)	ATLDAAVAAVQNGATVIIGGGDTATVAAK
☑	295	825.4113	1648.8080	1648.7978	6.21	1	5	1.2e+02	1	1170s (19.50m)	LSKSQDVDGSPLMEK + Oxidation (M)
☑	108	517.2944	1032.5743	1032.5604	13.5	0	5	1.3e+02	1	614s (10.23m)	IFNPVTQSK
☑	184	635.8016	1269.5886	1269.5886	0.04	0	4	62	1	515s (8.58m)	FAWGPIMSAMK + 2 Oxidation (M)
☑	350	943.4446	1884.8747	1884.8399	18.4	2	4	73	1	1554s (25.90m)	FMIRYFECNGYRDR + Oxidation (M)
☑	172	617.2641	1232.5136	1232.5352	-17.51	1	4	12	1	989s (16.48m)	GCYSMVGKMGGK + Oxidation (M)
☑	332	890.9320	1779.8494	1779.8396	5.52	1	4	1e+02	1	782s (13.03m)	RMCLTNALWSGSDQAK
☑	309	849.4434	1696.8722	1696.8970	-14.61	1	4	1.4e+02	1	699s (11.65m)	NGFIKALYEMVQIR + Oxidation (M)
☑	343	921.4065	1840.7984	1840.8349	-19.79	0	4	23	1	1003s (16.72m)	THTGEKPHACQFPGCTK
☑	290	542.6290	1624.8651	1624.8685	-2.13	1	4	1.2e+02	1	1148s (19.13m)	DIILHDARDLAFAR
☑	328	882.4150	1762.8155	1762.8342	-10.61	1	4	72	1	766s (12.77m)	DNGCGIAKDELAMALAR + Oxidation (M)
☑	64	458.7854	915.5563	915.5389	19.0	1	4	1e+02	1	940s (15.67m)	TLNEIKAK
☑	70	472.2850	942.5554	942.5498	5.93	1	4	1.7e+02	1	962s (16.03m)	TPQKQLTK
☑	364	1042.0036	2081.9927	2081.9953	-1.22	1	4	1.1e+02	1	843s (14.05m)	THHSRDSWLALLDEAGMK + Oxidation (M)

☑	365	1042.0036	2081.9927	2082.0211	-13.62	2	4	1.1e+02	1	847s (14.12m)	SLPDIHSRANNPTSRMMR
☑	385	882.1286	2643.3639	2643.3253	14.6	2	4	1.5e+02	1	1267s (21.12m)	DSVGAVVLGDYEHLREGDVAKTTGR
☑	294	816.9532	1631.8918	1631.8856	3.84	2	4	1.5e+02	1	622s (10.37m)	TNLAIYQQQRRGGK
☑	387	894.1329	2679.3768	2679.3282	18.1	1	3	1.5e+02	1	1838s (30.63m)	MSSQTPVVTVDGPSGAGKGTLCMLLSK + Oxidation
☑	152	596.8092	1191.6037	1191.6030	0.64	2	3	1.8e+02	1	765s (12.75m)	DLTEMARRGK + Oxidation (M)
☑	68	463.2976	924.5805	924.5869	-6.84	0	3	42	1	1065s (17.75m)	TVRPIAIR
☑	325	587.6708	1759.9905	1759.9733	9.77	1	3	95	1	567s (9.45m)	LISNGAYGAVYLVRHK
☑	303	840.4051	1678.7957	1678.8270	-18.65	0	3	1.3e+02	1	833s (13.88m)	MTVVSVSMPESLLDR + Oxidation (M)
☑	312	853.9047	1705.7948	1705.7730	12.8	0	3	1.1e+02	1	899s (14.98m)	LSGEALMGDGHYGIDR + Oxidation (M)
☑	111	1046.4926	1045.4853	1045.5040	-17.83	0	3	2.8e+02	1	1041s (17.35m)	EQGSSPLNSK
☑	71	472.2850	942.5554	942.5498	5.93	1	3	2.2e+02	1	965s (16.08m)	TPQKQLTK
☑	324	585.3277	1752.9613	1752.9621	-0.48	1	3	1.5e+02	1	1008s (16.80m)	HIGKTEDVLETASLLK
☑	58	450.2725	898.5304	898.5137	18.6	0	3	1.4e+02	1	935s (15.58m)	LPGFRPGR
☑	231	698.8510	1395.6874	1395.6915	-2.95	1	2	1.9e+02	1	781s (13.02m)	MDETKSTNLLTK + Oxidation (M)
☑	380	817.7549	2450.2427	2450.2596	-6.89	2	2	2.1e+02	1	790s (13.17m)	GVADVCRKLGVPLLLNCAYSMGR + Oxidation (M)
☑	16	381.1885	760.3624	760.3715	-12.04	0	2	1.5e+02	1	493s (8.22m)	AVETADR
☑	366	697.3836	2089.1289	2089.1064	10.7	2	2	1.9e+02	1	693s (11.55m)	MIRIFLTGYMGAGKTTLGK + 2 Oxidation (M)
☑	181	633.3168	1264.6191	1264.6339	-11.72	1	2	1.9e+02	1	557s (9.28m)	KESYSVYVYK
☑	300	833.3998	1664.7849	1664.7862	-0.73	0	2	1.4e+02	1	835s (13.92m)	TMLEQMAQALATGER + Oxidation (M)
☑	243	724.3806	1446.7467	1446.7462	0.32	0	2	2.8e+02	1	1189s (19.82m)	LDMVLVMTVEPGK + Oxidation (M)
☑	57	450.2725	898.5304	898.5236	7.55	0	2	1.8e+02	1	934s (15.57m)	TDKPAVIR
☑	126	564.2740	1126.5334	1126.5506	-15.29	0	2	1.2e+02	1	567s (9.45m)	IDKPEADPDK
☑	304	840.9006	1679.7867	1679.7890	-1.36	0	2	1.5e+02	1	853s (14.22m)	IGFEIGIENEEDTSK
☑	329	882.8787	1763.7428	1763.7647	-12.44	0	1	31	1	1274s (21.23m)	NMGFLYAPSYHSSMK + 2 Oxidation (M)
☑	12	626.9797	1877.9174	1877.9404	-12.28	0	1	2.5e+02	1	694s (11.57m)	ASISSLGAGSGMDLGSLLDK
☑	263	758.8617	1515.7088	1515.7347	-17.09	0	1	1.6e+02	1	978s (16.30m)	MTVMSLTATLMAAK + 3 Oxidation (M)
☑	95	502.8123	1003.6101	1003.5927	17.4	2	0	1.4e+02	1	993s (16.55m)	QLAKRFNK
☑	139	582.3506	1162.6866	1162.7074	-17.86	2	0	1.2e+02	1	1108s (18.47m)	KKIYGSIINK
☑	390	724.3779	2893.4825	2893.4419	14.0	2	0	3.1e+02	1	1193s (19.88m)	DLVIASKAWSESIYAKDMGVEIPEDK
☑	262	758.8617	1515.7088	1515.7390	-19.91	2	0	1.7e+02	1	976s (16.27m)	ARTDVQRDAEEAR
☑	334	897.4356	1792.8566	1792.8739	-9.63	0	0	2.5e+02	1	715s (11.92m)	MELLFEIGMEEIPAR + Oxidation (M)
☑	291	542.6290	1624.8651	1624.8685	-2.13	1	0	3.1e+02	1	1143s (19.05m)	DIILHDARDLAFAR
☑	104	516.3119	1030.6092	1030.5923	16.3	1	0	2.2e+02	1	1025s (17.08m)	FVRAILAQN
☑	23	*392.7470*	*783.4794*							*849s (14.15m)*	
☑	28	*814.8696*	*813.8624*							*913s (15.22m)*	
☑	29	*815.3664*	*814.3591*							*909s (15.15m)*	
☑	34	*414.7596*	*827.5046*							*877s (14.62m)*	
☑	35	*414.7596*	*827.5046*							*879s (14.65m)*	
☑	55	*436.7724*	*871.5302*							*908s (15.13m)*	
☑	78	*478.2507*	*954.4869*							*575s (9.58m)*	
☑	79	*478.2507*	*954.4869*							*570s (9.50m)*	
☑	80	*478.7909*	*955.5672*							*698s (11.63m)*	
☑	81	*480.7996*	*959.5846*							*963s (16.05m)*	
☑	82	*480.7996*	*959.5846*							*967s (16.12m)*	
☑	86	*494.2986*	*986.5827*							*992s (16.53m)*	
☑	94	*502.8123*	*1003.6101*							*995s (16.58m)*	
☑	115	*538.3241*	*1074.6336*							*1051s (17.52m)*	
☑	116	*538.3241*	*1074.6336*							*1048s (17.47m)*	
☑	127	*564.2740*	*1126.5334*							*571s (9.52m)*	
☑	195	*645.3041*	*1288.5936*							*951s (15.85m)*	
☑	206	*662.3072*	*1322.5998*							*1337s (22.28m)*	
☑	232	*700.7596*	*1399.5047*							*816s (13.60m)*	
☑	233	*700.7596*	*1399.5047*							*813s (13.55m)*	
☑	244	*724.3806*	*1446.7467*							*1196s (19.93m)*	
☑	267	*769.3204*	*1536.6262*							*893s (14.88m)*	
☑	268	*769.3204*	*1536.6262*							*895s (14.92m)*	
☑	279	*785.3031*	*1568.5917*							*937s (15.62m)*	
☑	292	*815.7831*	*1629.5517*							*808s (13.47m)*	
☑	313	*853.9047*	*1705.7948*							*896s (14.93m)*	
☑	319	*869.8908*	*1737.7670*							*927s (15.45m)*	
☑	320	*869.8908*	*1737.7670*							*931s (15.52m)*	
☑	323	*585.3277*	*1752.9613*							*1006s (16.77m)*	
☑	327	*882.3833*	*1762.7521*							*1271s (21.18m)*	
☑	337	*906.3646*	*1810.7146*							*494s (8.23m)*	
☑	339	*913.8934*	*1825.7723*							*836s (13.93m)*	
☑	353	*953.9510*	*1905.8874*							*638s (10.63m)*	
☑	356	*970.9654*	*1939.9163*							*806s (13.43m)*	
☑	360	*992.9232*	*1983.8319*							*602s (10.03m)*	
☑	367	*1057.9250*	*2113.8354*							*1378s (22.97m)*	
☑	382	*850.7471*	*2549.2196*							*1009s (16.82m)*	
☑	388	*933.0842*	*2796.2307*							*903s (15.05m)*	
☑	389	*933.4193*	*2797.2362*							*906s (15.10m)*	
☑	392	*843.6409*	*3370.5344*							*519s (8.65m)*	
☑	393	*843.6409*	*3370.5344*							*516s (8.60m)*	

Search Parameters

```
Type of search         : MS/MS Ion Search
Enzyme                 : Trypsin
Variable modifications : Oxidation (M)
Mass values            : Monoisotopic
Protein Mass           : Unrestricted
Peptide Mass Tolerance : ± 20 ppm
Fragment Mass Tolerance: ± 0.2 Da
Max Missed Cleavages   : 2
Instrument type        : ESI-QUAD-TOF
Number of queries      : 393
```

Mascot: http://www.matrixscience.com/

Mascot Search Results

Host	:	10.10.10.100
User	:	alison@anatomy
Email	:	
Search title	:	CPFP SCH6428 [1/18] 140331_alison_set3_10.mgf
MS data file	:	140331_alison_set3_10.mgf.mascot
Database	:	SWISSPROT sprot (538010 sequences; 190998508 residues)
Timestamp	:	2 Apr 2014 at 00:30:15 GMT
Protein hits	:	TRYP_PIG RecName: Full=Trypsin; EC=3.4.21.4; Flags: Precursor; - OS=Sus scrofa (Pig).
		HEMO_HUMAN RecName: Full=Hemopexin; AltName: Full=Beta-1B-glycoprotein; Flags: Precursor; - OS=Homo sapiens (Human).
		LG3BP_HUMAN RecName: Full=Galectin-3-binding protein; AltName: Full=Basement membrane autoantigen p105; AltName: Full=Lectin
		K2C5_BOVIN RecName: Full=Keratin, type II cytoskeletal 5; AltName: Full=Cytokeratin-5; Short=CK-5; AltName: Full=Keratin-5;
		DCD_HUMAN RecName: Full=Dermcidin; EC=3.4.-.-; AltName: Full=Preproteolysin; Contains: RecName: Full=Survival-promoting pe
		GP_PTPV RecName: Full=Envelope glycoprotein; Short=GP; AltName: Full=M polyprotein; Contains: RecName: Full=Non-structur
		TRY1_RAT RecName: Full=Anionic trypsin-1; EC=3.4.21.4; AltName: Full=Anionic trypsin I; AltName: Full=Pretrypsinogen I; A
		ACTA_BOVIN RecName: Full=Actin, aortic smooth muscle; AltName: Full=Alpha-actin-2; Flags: Precursor; - OS=Bos taurus (Bovin
		HSLU_HAHCH RecName: Full=ATP-dependent protease ATPase subunit HslU; AltName: Full=Unfoldase HslU; - OS=Hahella chejuensis
		TOM1_ASHGO RecName: Full=Probable E3 ubiquitin-protein ligase TOM1; EC=6.3.2.- OS=Ashbya gossypii (strain ATCC 10895 / CBS
		K1C10_BOVIN RecName: Full=Keratin, type I cytoskeletal 10; AltName: Full=Cytokeratin VIB; AltName: Full=Cytokeratin-10; Shor
		SYGB_AROAE RecName: Full=Glycine--tRNA ligase beta subunit; EC=6.1.1.14; AltName: Full=Glycyl-tRNA synthetase beta subunit;
		MLE_DICDI RecName: Full=Myosin, essential light chain; AltName: Full=EMLC; AltName: Full=Myosin light chain alkali OS=Dict
		NDST_CAEEL RecName: Full=Bifunctional heparan sulfate N-deacetylase/N-sulfotransferase 1; EC=2.8.2.8; AltName: Full=Glucosa
		PANB_ACIAC RecName: Full=3-methyl-2-oxobutanoate hydroxymethyltransferase; EC=2.1.2.11; AltName: Full=Ketopantoate hydroxym
		TGL_BACP2 RecName: Full=Protein-glutamine gamma-glutamyltransferase; EC=2.3.2.13; AltName: Full=Transglutaminase; Short=TG
		ISPT_CHLTE RecName: Full=Isoprenyl transferase; EC=2.5.1.-; - OS=Chlorobium tepidum (strain ATCC 49652 / DSM 12025 / TLS).
		SPZ1A_WHEAT RecName: Full=Serpin-Z1A; AltName: Full=TriaeZ1a; AltName: Full=WSZ1a; Short=WSZ1; AltName: Full=WSZCI OS=Tritic
		LEU3B_ASPNG RecName: Full=3-isopropylmalate dehydrogenase B; Short=3-IPM-DH B; Short=IMDH B; EC=1.1.1.85; AltName: Full=Beta
		ARGR_SHEFN RecName: Full=Arginine repressor; - OS=Shewanella frigidimarina (strain NCIMB 400).

	SWISSPROT	Decoy	False discovery rate
Peptide matches above identity threshold	19	0	0.00 %
Peptide matches above homology or identity threshold	24	2	8.33 %

Mascot Score Histogram

Ions score is -10*Log(P), where P is the probability that the observed match is a random event.
Individual ions scores > 36 indicate identity or extensive homology (p<0.05).
Protein scores are derived from ions scores as a non-probabilistic basis for ranking protein hits.

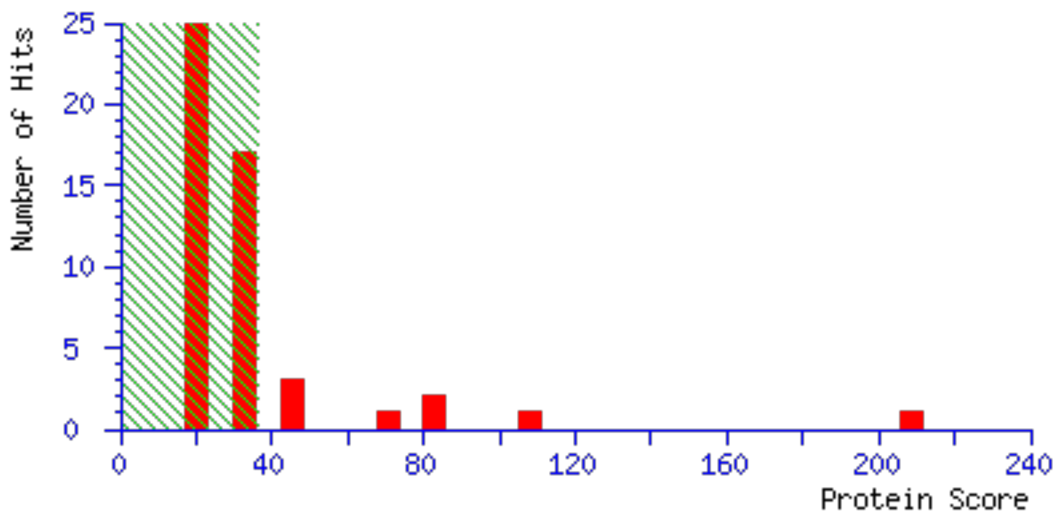

Peptide Summary Report

Format As	Peptide Summary		Help
	Significance threshold p< 0.05	Max. number of hits 20	Show Percolator scores
	Standard scoring ◉ MudPIT scoring ○	Ions score or expect cut-off 0	Show sub-sets 0
	Show pop-ups ◉ Suppress pop-ups ○	Sort unassigned Decreasing Score	Require bold red
	Preferred taxonomy All entries		

1. TRYP_PIG **Mass:** 24394 **Score:** 208 **Matches:** 10(10) **Sequences:** 3(3) **emPAI:** 0.47
RecName: Full=Trypsin; EC=3.4.21.4; Flags: Precursor; - OS=Sus scrofa (Pig).
☐ Check to include this hit in error tolerant search or archive report

	Query	Observed	Mr(expt)	Mr(calc)	ppm	Miss	Score	Expect	Rank	Unique	Retention Time	Peptide
☑	49	421.7572	841.4999	841.5022	-2.62	0	(51)	0.0025	1	U	679s (11.32m)	R.VATVSLPR.S
☑	50	421.7572	841.4999	841.5022	-2.62	0	(56)	0.0007	1	U	676s (11.27m)	R.VATVSLPR.S
☑	51	421.7586	841.5027	841.5022	0.71	0	62	0.00019	1	U	767s (12.78m)	R.VATVSLPR.S
☑	52	421.7591	841.5036	841.5022	1.68	0	(60)	0.00028	1	U	492s (8.20m)	R.VATVSLPR.S
☑	53	421.7591	841.5036	841.5022	1.68	0	(57)	0.0006	1	U	495s (8.25m)	R.VATVSLPR.S
☑	54	421.7592	841.5038	841.5022	1.99	0	(56)	0.00072	1	U	588s (9.80m)	R.VATVSLPR.S
☑	55	421.7592	841.5038	841.5022	1.99	0	(58)	0.00045	1	U	585s (9.75m)	R.VATVSLPR.S
☑	154	523.2856	1044.5567	1044.5564	0.32	0	74	1.8e-05	1	U	462s (7.70m)	K.LSSPATLNSR.V
☑	22	737.7066	2210.0980	2210.0967	0.56	0	72	2.2e-05	1	U	729s (12.15m)	R.LGEHNIDVLEGNEQFINAAK.I
☑	23	737.7095	2210.1066	2210.0967	4.47	0	(69)	4.8e-05	1	U	726s (12.10m)	R.LGEHNIDVLEGNEQFINAAK.I

2. HEMO_HUMAN **Mass:** 51643 **Score:** 107 **Matches:** 3(3) **Sequences:** 2(2) **emPAI:** 0.13
RecName: Full=Hemopexin; AltName: Full=Beta-1B-glycoprotein; Flags: Precursor; - OS=Homo sapiens (Human).
☐ Check to include this hit in error tolerant search or archive report

	Query	Observed	Mr(expt)	Mr(calc)	ppm	Miss	Score	Expect	Rank	Unique	Retention Time	Peptide
☑	192	571.3003	1140.5860	1140.5815	3.91	0	58	0.00047	1	U	577s (9.62m)	K.GGYTLVSGYPK.R

	Query	Observed	Mr(expt)	Mr(calc)	ppm	Miss	Score	Expect	Rank	Unique	Retention Time	Peptide
☑	232	610.8090	1219.6034	1219.5986	3.95	0	(42)	0.024	1	U	739s (12.32m)	K.NFPSPVDAAFR.Q
☑	233	610.8090	1219.6034	1219.5986	3.95	0	49	0.0043	1	U	736s (12.27m)	K.NFPSPVDAAFR.Q

Proteins matching the same set of peptides:

HEMO_PONAB **Mass:** 51581 **Score:** 107 **Matches:** 3(3) **Sequences:** 2(2)
RecName: Full=Hemopexin; Flags: Precursor; - OS=Pongo abelii (Sumatran orangutan) (Pongo pygmaeus abelii).

---

3. LG3BP_HUMAN **Mass:** 65289 **Score:** 79 **Matches:** 1(1) **Sequences:** 1(1) **emPAI:** 0.05
RecName: Full=Galectin-3-binding protein; AltName: Full=Basement membrane autoantigen p105; AltName: Full=Lectin galactoside-binding sol
☐ Check to include this hit in error tolerant search or archive report

	Query	Observed	Mr(expt)	Mr(calc)	ppm	Miss	Score	Expect	Rank	Unique	Retention Time	Peptide
☑	341	799.8420	1597.6694	1597.6685	0.59	0	79	5.5e-07	1	U	593s (9.88m)	K.YSSDYFQAPSDYR.Y

Proteins matching the same set of peptides:

LG3BP_PONAB **Mass:** 65225 **Score:** 79 **Matches:** 1(1) **Sequences:** 1(1)
RecName: Full=Galectin-3-binding protein; AltName: Full=Lectin galactoside-binding soluble 3-binding protein; Flags: Precursor; - OS=Pon

---

4. K2C5_BOVIN **Mass:** 62898 **Score:** 78 **Matches:** 1(1) **Sequences:** 1(1) **emPAI:** 0.05
RecName: Full=Keratin, type II cytoskeletal 5; AltName: Full=Cytokeratin-5; Short=CK-5; AltName: Full=Keratin-5; Short=K5; AltName: Full
☐ Check to include this hit in error tolerant search or archive report

	Query	Observed	Mr(expt)	Mr(calc)	ppm	Miss	Score	Expect	Rank	Unique	Retention Time	Peptide
☑	267	651.8626	1301.7106	1301.7078	2.14	0	78	5.8e-06	1	U	1116s (18.60m)	R.SLDLDSIIAEVK.A

Proteins matching the same set of peptides:

K2C5_MOUSE **Mass:** 61729 **Score:** 78 **Matches:** 1(1) **Sequences:** 1(1)
RecName: Full=Keratin, type II cytoskeletal 5; AltName: Full=Cytokeratin-5; Short=CK-5; AltName: Full=Keratin-5; Short=K5; AltName: Full
K2C5_RAT **Mass:** 61788 **Score:** 78 **Matches:** 1(1) **Sequences:** 1(1)
RecName: Full=Keratin, type II cytoskeletal 5; AltName: Full=Cytokeratin-5; Short=CK-5; AltName: Full=Keratin-5; Short=K5; AltName: Full
K2C6A_MOUSE **Mass:** 59299 **Score:** 78 **Matches:** 1(1) **Sequences:** 1(1)
RecName: Full=Keratin, type II cytoskeletal 6A; AltName: Full=Cytokeratin-6A; Short=CK-6A; AltName: Full=Keratin-6-alpha; Short=mK6-alph
K2C6A_RAT **Mass:** 59213 **Score:** 78 **Matches:** 1(1) **Sequences:** 1(1)
RecName: Full=Keratin, type II cytoskeletal 6A; AltName: Full=Cytokeratin-6A; Short=CK-6A; AltName: Full=Keratin-6A; Short=K6A; AltName:
K2C6B_MOUSE **Mass:** 60285 **Score:** 78 **Matches:** 1(1) **Sequences:** 1(1)
RecName: Full=Keratin, type II cytoskeletal 6B; AltName: Full=Cytokeratin-6B; Short=CK-6B; AltName: Full=Keratin-6-beta; Short=mK6-beta;
K2C75_BOVIN **Mass:** 59000 **Score:** 78 **Matches:** 1(1) **Sequences:** 1(1)
RecName: Full=Keratin, type II cytoskeletal 75; AltName: Full=Cytokeratin-75; Short=CK-75; AltName: Full=Keratin-6 hair follicle; AltNam
K2C75_MOUSE **Mass:** 59704 **Score:** 78 **Matches:** 1(1) **Sequences:** 1(1)
RecName: Full=Keratin, type II cytoskeletal 75; AltName: Full=Cytokeratin-75; Short=CK-75; AltName: Full=Keratin-6 hair follicle; Short=
K2C75_RAT **Mass:** 58991 **Score:** 78 **Matches:** 1(1) **Sequences:** 1(1)
RecName: Full=Keratin, type II cytoskeletal 75; AltName: Full=Cytokeratin-75; Short=CK-75; AltName: Full=Keratin-6 hair follicle; AltNam
K2C7_BOVIN **Mass:** 51546 **Score:** 78 **Matches:** 1(1) **Sequences:** 1(1)
RecName: Full=Keratin, type II cytoskeletal 7; AltName: Full=Cytokeratin-7; Short=CK-7; AltName: Full=Keratin-7; Short=K7; AltName: Full
K2C1_HUMAN **Mass:** 65999 **Score:** 78 **Matches:** 1(1) **Sequences:** 1(1)
RecName: Full=Keratin, type II cytoskeletal 1; AltName: Full=67 kDa cytokeratin; AltName: Full=Cytokeratin-1; Short=CK-1; AltName: Full=
K2C1_PANTR **Mass:** 65450 **Score:** 78 **Matches:** 1(1) **Sequences:** 1(1)
RecName: Full=Keratin, type II cytoskeletal 1; AltName: Full=Cytokeratin-1; Short=CK-1; AltName: Full=Keratin-1; Short=K1; AltName: Full

---

5. DCD_HUMAN **Mass:** 11277 **Score:** 65 **Matches:** 2(2) **Sequences:** 1(1) **emPAI:** 0.30
RecName: Full=Dermcidin; EC=3.4.-.-; AltName: Full=Preproteolysin; Contains: RecName: Full=Survival-promoting peptide; Contains: RecName
☐ Check to include this hit in error tolerant search or archive report

	Query	Observed	Mr(expt)	Mr(calc)	ppm	Miss	Score	Expect	Rank	Unique	Retention Time	Peptide
☑	201	581.2890	1160.5634	1160.5561	6.27	0	(63)	0.00015	1	U	582s (9.70m)	K.DAVEDLESVGK.G
☑	202	581.2890	1160.5634	1160.5561	6.29	0	65	9.5e-05	1	U	586s (9.77m)	K.DAVEDLESVGK.G

---

6. GP_PTPV **Mass:** 146280 **Score:** 45 **Matches:** 2(1) **Sequences:** 1(1) **emPAI:** 0.02
RecName: Full=Envelope glycoprotein; Short=GP; AltName: Full=M polyprotein; Contains: RecName: Full=Non-structural protein NSm; Contains
☐ Check to include this hit in error tolerant search or archive report

	Query	Observed	Mr(expt)	Mr(calc)	ppm	Miss	Score	Expect	Rank	Unique	Retention Time	Peptide
☑	168	539.7912	1077.5679	1077.5852	-16.03	0	(14)	15	1	U	530s (8.83m)	K.CSITATITLR.A
☑	169	539.7912	1077.5679	1077.5852	-16.03	0	45	0.013	1	U	526s (8.77m)	K.CSITATITLR.A

---

7. TRY1_RAT **Score:** 43 **Matches:** 2(1) **Sequences:** 1(1) **emPAI:** 0.12
RecName: Full=Anionic trypsin-1; EC=3.4.21.4; AltName: Full=Anionic trypsin I; AltName: Full=Pretrypsinogen I; AltName: Full=Serine prot
☐ Check to include this hit in error tolerant search or archive report

	Query	Observed	Mr(expt)	Mr(calc)	ppm	Miss	Score	Expect	Rank	Unique	Retention Time	Peptide
	22	737.7066	2210.0980	2210.0967	0.56	0	43	0.02	2	U	729s (12.15m)	R.LGEHNINVLEGDEQFINAAK.I
	23	737.7095	2210.1066	2210.0967	4.47	0	(25)	1.1	2	U	726s (12.10m)	R.LGEHNINVLEGDEQFINAAK.I

---

8. ACTA_BOVIN **Mass:** 41982 **Score:** 41 **Matches:** 1(1) **Sequences:** 1(1) **emPAI:** 0.08
RecName: Full=Actin, aortic smooth muscle; AltName: Full=Alpha-actin-2; Flags: Precursor; - OS=Bos taurus (Bovine).
☐ Check to include this hit in error tolerant search or archive report

	Query	Observed	Mr(expt)	Mr(calc)	ppm	Miss	Score	Expect	Rank	Unique	Retention Time	Peptide
☑	31	398.2374	794.4602	794.4650	-6.05	0	41	0.0093	1	U	478s (7.97m)	K.IIAPPER.K

Proteins matching the same set of peptides:
ACTA_CHICK **Mass:** 41968 **Score:** 41 **Matches:** 1(1) **Sequences:** 1(1)
RecName: Full=Actin, aortic smooth muscle; AltName: Full=Alpha-actin; Flags: Precursor; - OS=Gallus gallus (Chicken).
ACTA_HUMAN **Mass:** 41982 **Score:** 41 **Matches:** 1(1) **Sequences:** 1(1)
RecName: Full=Actin, aortic smooth muscle; AltName: Full=Alpha-actin-2; AltName: Full=Cell growth-inhibiting gene 46 protein; Flags: Pre
ACTA_LIMPO **Mass:** 41748 **Score:** 41 **Matches:** 1(1) **Sequences:** 1(1)
RecName: Full=Actin, acrosomal process isoform; AltName: Full=Actin-5; Flags: Precursor; - OS=Limulus polyphemus (Atlantic horseshoe cra
ACTA_MOUSE **Mass:** 41982 **Score:** 41 **Matches:** 1(1) **Sequences:** 1(1)
RecName: Full=Actin, aortic smooth muscle; AltName: Full=Alpha-actin-2; Flags: Precursor; - OS=Mus musculus (Mouse).
ACTA_PHYPO **Mass:** 41773 **Score:** 41 **Matches:** 1(1) **Sequences:** 1(1)
RecName: Full=Actin, plasmodial isoform; - OS=Physarum polycephalum (Slime mold).
ACTA_RABIT **Mass:** 41982 **Score:** 41 **Matches:** 1(1) **Sequences:** 1(1)
RecName: Full=Actin, aortic smooth muscle; AltName: Full=Alpha-actin-2; Flags: Precursor; - OS=Oryctolagus cuniculus (Rabbit).
ACTA_RAT **Mass:** 41982 **Score:** 41 **Matches:** 1(1) **Sequences:** 1(1)
RecName: Full=Actin, aortic smooth muscle; AltName: Full=Alpha-actin-2; Flags: Precursor; - OS=Rattus norvegicus (Rat).
ACTA_STRPU **Mass:** 41822 **Score:** 41 **Matches:** 1(1) **Sequences:** 1(1)
RecName: Full=Actin, cytoskeletal 1A; AltName: Full=Actin, cytoskeletal IA; Flags: Precursor; - OS=Strongylocentrotus purpuratus (Purple
ACTBL_HUMAN **Mass:** 41976 **Score:** 41 **Matches:** 1(1) **Sequences:** 1(1)
RecName: Full=Beta-actin-like protein 2; AltName: Full=Kappa-actin; - OS=Homo sapiens (Human).
ACTBL_MOUSE **Mass:** 41977 **Score:** 41 **Matches:** 1(1) **Sequences:** 1(1)
RecName: Full=Beta-actin-like protein 2; AltName: Full=Kappa-actin; - OS=Mus musculus (Mouse).
ACTB_BOSMU **Mass:** 41722 **Score:** 41 **Matches:** 1(1) **Sequences:** 1(1)
RecName: Full=Actin, cytoplasmic 1; AltName: Full=Beta-actin; Contains: RecName: Full=Actin, cytoplasmic 1, N-terminally processed; - OS
ACTB_BOVIN **Mass:** 41710 **Score:** 41 **Matches:** 1(1) **Sequences:** 1(1)
RecName: Full=Actin, cytoplasmic 1; AltName: Full=Beta-actin; Contains: RecName: Full=Actin, cytoplasmic 1, N-terminally processed; - OS
ACTB_CAMDR **Mass:** 41775 **Score:** 41 **Matches:** 1(1) **Sequences:** 1(1)
RecName: Full=Actin, cytoplasmic 1; AltName: Full=Beta-actin; Contains: RecName: Full=Actin, cytoplasmic 1, N-terminally processed; - OS
ACTB_CANFA **Mass:** 41710 **Score:** 41 **Matches:** 1(1) **Sequences:** 1(1)
RecName: Full=Actin, cytoplasmic 1; AltName: Full=Beta-actin; Contains: RecName: Full=Actin, cytoplasmic 1, N-terminally processed; - OS
ACTB_CAVPO **Mass:** 41710 **Score:** 41 **Matches:** 1(1) **Sequences:** 1(1)
RecName: Full=Actin, cytoplasmic 1; AltName: Full=Beta-actin; Contains: RecName: Full=Actin, cytoplasmic 1, N-terminally processed; - OS
ACTB_CERPY **Mass:** 40419 **Score:** 41 **Matches:** 1(1) **Sequences:** 1(1)
RecName: Full=Actin, cytoplasmic 1; AltName: Full=Beta-actin; - OS=Cercopithecus pygerythrus (Vervet monkey).
ACTB_CHICK **Mass:** 41710 **Score:** 41 **Matches:** 1(1) **Sequences:** 1(1)
RecName: Full=Actin, cytoplasmic 1; AltName: Full=Beta-actin; Contains: RecName: Full=Actin, cytoplasmic 1, N-terminally processed; - OS
ACTB_CHLAE **Mass:** 41710 **Score:** 41 **Matches:** 1(1) **Sequences:** 1(1)
RecName: Full=Actin, cytoplasmic 1; AltName: Full=Beta-actin; Contains: RecName: Full=Actin, cytoplasmic 1, N-terminally processed; - OS
ACTB_CRIGR **Mass:** 41711 **Score:** 41 **Matches:** 1(1) **Sequences:** 1(1)
RecName: Full=Actin, cytoplasmic 1; AltName: Full=Beta-actin; Contains: RecName: Full=Actin, cytoplasmic 1, N-terminally processed; - OS
ACTB_CTEID **Mass:** 41726 **Score:** 41 **Matches:** 1(1) **Sequences:** 1(1)
RecName: Full=Actin, cytoplasmic 1; AltName: Full=Beta-actin; Contains: RecName: Full=Actin, cytoplasmic 1, N-terminally processed; - OS
ACTB_CYPCA **Mass:** 41726 **Score:** 41 **Matches:** 1(1) **Sequences:** 1(1)
RecName: Full=Actin, cytoplasmic 1; AltName: Full=Beta-actin; Contains: RecName: Full=Actin, cytoplasmic 1, N-terminally processed; - OS
ACTB_HORSE **Mass:** 41710 **Score:** 41 **Matches:** 1(1) **Sequences:** 1(1)
RecName: Full=Actin, cytoplasmic 1; AltName: Full=Beta-actin; Contains: RecName: Full=Actin, cytoplasmic 1, N-terminally processed; - OS
ACTB_HUMAN **Mass:** 41710 **Score:** 41 **Matches:** 1(1) **Sequences:** 1(1)
RecName: Full=Actin, cytoplasmic 1; AltName: Full=Beta-actin; Contains: RecName: Full=Actin, cytoplasmic 1, N-terminally processed; - OS
ACTB_MACFA **Mass:** 41710 **Score:** 41 **Matches:** 1(1) **Sequences:** 1(1)
RecName: Full=Actin, cytoplasmic 1; AltName: Full=Beta-actin; Contains: RecName: Full=Actin, cytoplasmic 1, N-terminally processed; - OS
ACTB_MESAU **Mass:** 41710 **Score:** 41 **Matches:** 1(1) **Sequences:** 1(1)
RecName: Full=Actin, cytoplasmic 1; AltName: Full=Beta-actin; Contains: RecName: Full=Actin, cytoplasmic 1, N-terminally processed; - OS
ACTB_MOUSE **Mass:** 41710 **Score:** 41 **Matches:** 1(1) **Sequences:** 1(1)
RecName: Full=Actin, cytoplasmic 1; AltName: Full=Beta-actin; Contains: RecName: Full=Actin, cytoplasmic 1, N-terminally processed; - OS
ACTB_OREMO **Mass:** 41740 **Score:** 41 **Matches:** 1(1) **Sequences:** 1(1)
RecName: Full=Actin, cytoplasmic 1; AltName: Full=Beta-actin; Contains: RecName: Full=Actin, cytoplasmic 1, N-terminally processed; - OS
ACTB_ORYLA **Mass:** 41712 **Score:** 41 **Matches:** 1(1) **Sequences:** 1(1)
RecName: Full=Actin, cytoplasmic 1; AltName: Full=Beta-actin; AltName: Full=OlCA1; Contains: RecName: Full=Actin, cytoplasmic 1, N-termi
ACTB_PANTR **Mass:** 41710 **Score:** 41 **Matches:** 1(1) **Sequences:** 1(1)
RecName: Full=Actin, cytoplasmic 1; AltName: Full=Beta-actin; Contains: RecName: Full=Actin, cytoplasmic 1, N-terminally processed; - OS
ACTB_PIG **Mass:** 41710 **Score:** 41 **Matches:** 1(1) **Sequences:** 1(1)
RecName: Full=Actin, cytoplasmic 1; AltName: Full=Beta-actin; Contains: RecName: Full=Actin, cytoplasmic 1, N-terminally processed; - OS
ACTB_PONAB **Mass:** 41710 **Score:** 41 **Matches:** 1(1) **Sequences:** 1(1)
RecName: Full=Actin, cytoplasmic 1; AltName: Full=Beta-actin; Contains: RecName: Full=Actin, cytoplasmic 1, N-terminally processed; - OS
ACTB_RABIT **Mass:** 41729 **Score:** 41 **Matches:** 1(1) **Sequences:** 1(1)
RecName: Full=Actin, cytoplasmic 1; AltName: Full=Beta-actin; Contains: RecName: Full=Actin, cytoplasmic 1, N-terminally processed; - OS
ACTB_RAT **Mass:** 41710 **Score:** 41 **Matches:** 1(1) **Sequences:** 1(1)
RecName: Full=Actin, cytoplasmic 1; AltName: Full=Beta-actin; Contains: RecName: Full=Actin, cytoplasmic 1, N-terminally processed; - OS
ACTB_SALSA **Mass:** 41756 **Score:** 41 **Matches:** 1(1) **Sequences:** 1(1)
RecName: Full=Actin, cytoplasmic 1; AltName: Full=Beta-actin; Contains: RecName: Full=Actin, cytoplasmic 1, N-terminally processed; - OS
ACTB_SHEEP **Mass:** 41710 **Score:** 41 **Matches:** 1(1) **Sequences:** 1(1)
RecName: Full=Actin, cytoplasmic 1; AltName: Full=Beta-actin; Contains: RecName: Full=Actin, cytoplasmic 1, N-terminally processed; - OS
ACTB_SIGHI **Mass:** 41692 **Score:** 41 **Matches:** 1(1) **Sequences:** 1(1)
RecName: Full=Actin, cytoplasmic 1; AltName: Full=Beta-actin; Contains: RecName: Full=Actin, cytoplasmic 1, N-terminally processed; - OS
ACTB_SPECI **Mass:** 41710 **Score:** 41 **Matches:** 1(1) **Sequences:** 1(1)
RecName: Full=Actin, cytoplasmic 1; AltName: Full=Beta-actin; Contains: RecName: Full=Actin, cytoplasmic 1, N-terminally processed; - OS
ACTB_STRPU **Mass:** 41739 **Score:** 41 **Matches:** 1(1) **Sequences:** 1(1)
RecName: Full=Actin, cytoskeletal 1B; AltName: Full=Actin, cytoskeletal IB; Flags: Precursor; - OS=Strongylocentrotus purpuratus (Purple
ACTB_TRIVU **Mass:** 41710 **Score:** 41 **Matches:** 1(1) **Sequences:** 1(1)
RecName: Full=Actin, cytoplasmic 1; AltName: Full=Beta-actin; Contains: RecName: Full=Actin, cytoplasmic 1, N-terminally processed; - OS
ACTB_XENBO **Mass:** 41821 **Score:** 41 **Matches:** 1(1) **Sequences:** 1(1)
RecName: Full=Actin, cytoplasmic 1; AltName: Full=Beta actin; - OS=Xenopus borealis (Kenyan clawed frog).
ACTB_XENLA **Mass:** 41740 **Score:** 41 **Matches:** 1(1) **Sequences:** 1(1)

RecName: Full=Actin, cytoplasmic 1; AltName: Full=Beta-actin; AltName: Full=Cytoplasmic beta-actin; Contains: RecName: Full=Actin, cytop
ACTB_XENTR Mass: 41726 Score: 41 Matches: 1(1) Sequences: 1(1)
RecName: Full=Actin, cytoplasmic 1; AltName: Full=Beta-actin; Contains: RecName: Full=Actin, cytoplasmic 1, N-terminally processed; - OS
ACTC_BIOAL Mass: 41884 Score: 41 Matches: 1(1) Sequences: 1(1)
RecName: Full=Actin, cytoplasmic; Flags: Precursor; - OS=Biomphalaria alexandrina (Bloodfluke planorb).
ACTC_BIOGL Mass: 41854 Score: 41 Matches: 1(1) Sequences: 1(1)
RecName: Full=Actin, cytoplasmic; Flags: Precursor; - OS=Biomphalaria glabrata (Bloodfluke planorb) (Freshwater snail).
ACTC_BIOOB Mass: 41848 Score: 41 Matches: 1(1) Sequences: 1(1)
RecName: Full=Actin, cytoplasmic; Flags: Precursor; - OS=Biomphalaria obstructa (Bloodfluke planorb).
ACTC_BIOPF Mass: 41843 Score: 41 Matches: 1(1) Sequences: 1(1)
RecName: Full=Actin, cytoplasmic; Flags: Precursor; - OS=Biomphalaria pfeifferi (Bloodfluke planorb).
ACTC_BIOTE Mass: 41866 Score: 41 Matches: 1(1) Sequences: 1(1)
RecName: Full=Actin, cytoplasmic; Flags: Precursor; - OS=Biomphalaria tenagophila (Bloodfluke planorb).
ACTC_BOVIN Mass: 41992 Score: 41 Matches: 1(1) Sequences: 1(1)
RecName: Full=Actin, alpha cardiac muscle 1; AltName: Full=Alpha-cardiac actin; Flags: Precursor; - OS=Bos taurus (Bovine).
ACTC_BRABE Mass: 41678 Score: 41 Matches: 1(1) Sequences: 1(1)
RecName: Full=Actin, cytoplasmic; AltName: Full=BbCA1; Contains: RecName: Full=Actin, cytoplasmic, N-terminally processed; - OS=Branchic
ACTC_BRAFL Mass: 41694 Score: 41 Matches: 1(1) Sequences: 1(1)
RecName: Full=Actin, cytoplasmic; AltName: Full=BfCA1; Contains: RecName: Full=Actin, cytoplasmic, N-terminally processed; - OS=Branchic
ACTC_BRALA Mass: 41686 Score: 41 Matches: 1(1) Sequences: 1(1)
RecName: Full=Actin, cytoplasmic; Contains: RecName: Full=Actin, cytoplasmic, N-terminally processed; - OS=Branchiostoma lanceolatum (Co
ACTC_CHICK Mass: 41992 Score: 41 Matches: 1(1) Sequences: 1(1)
RecName: Full=Actin, alpha cardiac muscle 1; AltName: Full=Alpha-cardiac actin; Flags: Precursor; - OS=Gallus gallus (Chicken).
ACTC_HALRO Mass: 41775 Score: 41 Matches: 1(1) Sequences: 1(1)
RecName: Full=Actin, nonmuscle; - OS=Halocynthia roretzi (Sea squirt).
ACTC_HELTI Mass: 41898 Score: 41 Matches: 1(1) Sequences: 1(1)
RecName: Full=Actin, cytoplasmic; Flags: Precursor; - OS=Helisoma trivolvis (Snail).
ACTC_HUMAN Mass: 41992 Score: 41 Matches: 1(1) Sequences: 1(1)
RecName: Full=Actin, alpha cardiac muscle 1; AltName: Full=Alpha-cardiac actin; Flags: Precursor; - OS=Homo sapiens (Human).
ACTC_MOUSE Mass: 41992 Score: 41 Matches: 1(1) Sequences: 1(1)
RecName: Full=Actin, alpha cardiac muscle 1; AltName: Full=Alpha-cardiac actin; Flags: Precursor; - OS=Mus musculus (Mouse).
ACTC_PISOC Mass: 41822 Score: 41 Matches: 1(1) Sequences: 1(1)
RecName: Full=Actin, cytoplasmic; Flags: Precursor; - OS=Pisaster ochraceus (Ochre sea star) (Asterias ochracea).
ACTC_RAT Mass: 41992 Score: 41 Matches: 1(1) Sequences: 1(1)
RecName: Full=Actin, alpha cardiac muscle 1; AltName: Full=Alpha-cardiac actin; Flags: Precursor; - OS=Rattus norvegicus (Rat).
ACTC_STRPU Mass: 41774 Score: 41 Matches: 1(1) Sequences: 1(1)
RecName: Full=Actin, cytoskeletal 2A; AltName: Full=Actin, cytoskeletal IIA; Flags: Precursor; - OS=Strongylocentrotus purpuratus (Purpl
ACTC_STYPL Mass: 41903 Score: 41 Matches: 1(1) Sequences: 1(1)
RecName: Full=Actin, cytoplasmic; - OS=Styela plicata (Sea squirt) (Ascidia plicata).
ACTC_TAKRU Mass: 41948 Score: 41 Matches: 1(1) Sequences: 1(1)
RecName: Full=Actin, alpha cardiac; Flags: Precursor; - OS=Takifugu rubripes (Japanese pufferfish) (Fugu rubripes).
ACTC_XENLA Mass: 41989 Score: 41 Matches: 1(1) Sequences: 1(1)
RecName: Full=Actin, alpha cardiac muscle 1; AltName: Full=Actin alpha 1; AltName: Full=Alpha-cardiac actin; Flags: Precursor; - OS=Xeno
ACTC_XENTR Mass: 41992 Score: 41 Matches: 1(1) Sequences: 1(1)
RecName: Full=Actin, alpha cardiac muscle 1; AltName: Full=Actin alpha 1; AltName: Full=Alpha-cardiac actin; Flags: Precursor; - OS=Xeno
ACTD_STRPU Mass: 41772 Score: 41 Matches: 1(1) Sequences: 1(1)
RecName: Full=Actin, cytoskeletal 2B; AltName: Full=Actin, cytoskeletal IIB; Flags: Precursor; - OS=Strongylocentrotus purpuratus (Purpl
ACTE_STRPU Mass: 41877 Score: 41 Matches: 1(1) Sequences: 1(1)
RecName: Full=Actin, cytoskeletal 3A; AltName: Full=Actin, cytoskeletal IIIA; Flags: Precursor; - OS=Strongylocentrotus purpuratus (Purp
ACTF_STRPU Mass: 41805 Score: 41 Matches: 1(1) Sequences: 1(1)
RecName: Full=Actin, cytoskeletal 3B; AltName: Full=Actin, cytoskeletal IIIB; Flags: Precursor; - OS=Strongylocentrotus purpuratus (Purp
ACTG_ACRCH Mass: 41581 Score: 41 Matches: 1(1) Sequences: 1(1)
RecName: Full=Actin, gamma; - OS=Acremonium chrysogenum (Cephalosporium acremonium).
ACTG_ANSAN Mass: 41851 Score: 41 Matches: 1(1) Sequences: 1(1)
RecName: Full=Actin, cytoplasmic 2; AltName: Full=Gamma-actin; Contains: RecName: Full=Actin, cytoplasmic 2, N-terminally processed; - O
ACTG_BOVIN Mass: 41766 Score: 41 Matches: 1(1) Sequences: 1(1)
RecName: Full=Actin, cytoplasmic 2; AltName: Full=Gamma-actin; Contains: RecName: Full=Actin, cytoplasmic 2, N-terminally processed; - O
ACTG_CHICK Mass: 41766 Score: 41 Matches: 1(1) Sequences: 1(1)
RecName: Full=Actin, cytoplasmic 2; AltName: Full=Gamma-actin; Contains: RecName: Full=Actin, cytoplasmic 2, N-terminally processed; - O
ACTG_EMENI Mass: 41613 Score: 41 Matches: 1(1) Sequences: 1(1)
RecName: Full=Actin, gamma; - OS=Emericella nidulans (strain FGSC A4 / ATCC 38163 / CBS 112.46 / NRRL 194 / M139) (Aspergillus nidulans)
ACTG_HUMAN Mass: 41766 Score: 41 Matches: 1(1) Sequences: 1(1)
RecName: Full=Actin, cytoplasmic 2; AltName: Full=Gamma-actin; Contains: RecName: Full=Actin, cytoplasmic 2, N-terminally processed; - O
ACTG_MOUSE Mass: 41766 Score: 41 Matches: 1(1) Sequences: 1(1)
RecName: Full=Actin, cytoplasmic 2; AltName: Full=Gamma-actin; Contains: RecName: Full=Actin, cytoplasmic 2, N-terminally processed; - O
ACTG_PENCH Mass: 41730 Score: 41 Matches: 1(1) Sequences: 1(1)
RecName: Full=Actin, gamma; - OS=Penicillium chrysogenum (Penicillium notatum).
ACTG_RANLE Mass: 41752 Score: 41 Matches: 1(1) Sequences: 1(1)
RecName: Full=Actin, cytoplasmic 2; AltName: Full=Cytoplasmic actin type 5; AltName: Full=Gamma-actin; Contains: RecName: Full=Actin, cy
ACTG_RAT Mass: 41766 Score: 41 Matches: 1(1) Sequences: 1(1)
RecName: Full=Actin, cytoplasmic 2; AltName: Full=Gamma-actin; Contains: RecName: Full=Actin, cytoplasmic 2, N-terminally processed; - O
ACTG_TRISC Mass: 41724 Score: 41 Matches: 1(1) Sequences: 1(1)
RecName: Full=Actin, cytoplasmic 2; AltName: Full=Gamma-actin; Contains: RecName: Full=Actin, cytoplasmic 2, N-terminally processed; - O
ACTG_TRIVU Mass: 41766 Score: 41 Matches: 1(1) Sequences: 1(1)
RecName: Full=Actin, cytoplasmic 2; AltName: Full=Gamma-actin; Contains: RecName: Full=Actin, cytoplasmic 2, N-terminally processed; - O
ACTG_XENLA Mass: 41766 Score: 41 Matches: 1(1) Sequences: 1(1)
RecName: Full=Actin, cytoplasmic 2; AltName: Full=Gamma-actin; Contains: RecName: Full=Actin, cytoplasmic 2, N-terminally processed; - O
ACTG_XENTR Mass: 41738 Score: 41 Matches: 1(1) Sequences: 1(1)
RecName: Full=Actin, cytoplasmic 2; AltName: Full=Actin, cytoplasmic, type 5; AltName: Full=Gamma-actin; Contains: RecName: Full=Actin,
ACTH_BOVIN Mass: 41850 Score: 41 Matches: 1(1) Sequences: 1(1)
RecName: Full=Actin, gamma-enteric smooth muscle; AltName: Full=Gamma-2-actin; AltName: Full=Smooth muscle gamma-actin; Flags: Precursor
ACTH_CHICK Mass: 41850 Score: 41 Matches: 1(1) Sequences: 1(1)
RecName: Full=Actin, gamma-enteric smooth muscle; AltName: Full=Alpha-actin-3; AltName: Full=Gamma-2-actin; AltName: Full=Smooth muscle
ACTH_HUMAN Mass: 41850 Score: 41 Matches: 1(1) Sequences: 1(1)

RecName: Full=Actin, gamma-enteric smooth muscle; AltName: Full=Alpha-actin-3; AltName: Full=Gamma-2-actin; AltName: Full=Smooth muscle
ACTH_MOUSE **Mass:** 41850 **Score:** 41 **Matches:** 1(1) **Sequences:** 1(1)
RecName: Full=Actin, gamma-enteric smooth muscle; AltName: Full=Alpha-actin-3; AltName: Full=Gamma-2-actin; AltName: Full=Smooth muscle
ACTH_RAT **Mass:** 41850 **Score:** 41 **Matches:** 1(1) **Sequences:** 1(1)
RecName: Full=Actin, gamma-enteric smooth muscle; AltName: Full=Alpha-actin-3; AltName: Full=Gamma-2-actin; AltName: Full=Smooth muscle
ACTM_APLCA **Mass:** 41739 **Score:** 41 **Matches:** 1(1) **Sequences:** 1(1)
RecName: Full=Actin, muscle; Flags: Precursor; - OS=Aplysia californica (California sea hare).
ACTM_BRABE **Mass:** 42035 **Score:** 41 **Matches:** 1(1) **Sequences:** 1(1)
RecName: Full=Actin, muscle; AltName: Full=BbMA1; Flags: Precursor; - OS=Branchiostoma belcheri (Amphioxus).
ACTM_BRAFL **Mass:** 41904 **Score:** 41 **Matches:** 1(1) **Sequences:** 1(1)
RecName: Full=Actin, muscle; - OS=Branchiostoma floridae (Florida lancelet) (Amphioxus).
ACTM_BRALA **Mass:** 42160 **Score:** 41 **Matches:** 1(1) **Sequences:** 1(1)
RecName: Full=Actin, muscle; Flags: Precursor; - OS=Branchiostoma lanceolatum (Common lancelet) (Amphioxus).
ACTM_CIOSA **Mass:** 42076 **Score:** 41 **Matches:** 1(1) **Sequences:** 1(1)
RecName: Full=Actin, muscle; - OS=Ciona savignyi (Pacific transparent sea squirt).
ACTM_HELER **Mass:** 41721 **Score:** 41 **Matches:** 1(1) **Sequences:** 1(1)
RecName: Full=Actin, cytoskeletal; AltName: Full=M; Flags: Precursor; - OS=Heliocidaris erythrogramma (Sea urchin).
ACTM_HELTB **Mass:** 41735 **Score:** 41 **Matches:** 1(1) **Sequences:** 1(1)
RecName: Full=Actin, cytoskeletal; AltName: Full=M; Flags: Precursor; - OS=Heliocidaris tuberculata (Sea urchin).
ACTM_LYTPI **Mass:** 19286 **Score:** 41 **Matches:** 1(1) **Sequences:** 1(1)
RecName: Full=Actin, muscle; AltName: Full=LPM; Flags: Fragment; - OS=Lytechinus pictus (Painted sea urchin).
ACTM_MOLOC **Mass:** 42019 **Score:** 41 **Matches:** 1(1) **Sequences:** 1(1)
RecName: Full=Actin, larval muscle-type; AltName: Full=A1; - OS=Molgula oculata (Sea squirt).
ACTM_PISOC **Mass:** 41925 **Score:** 41 **Matches:** 1(1) **Sequences:** 1(1)
RecName: Full=Actin, muscle; Flags: Precursor; - OS=Pisaster ochraceus (Ochre sea star) (Asterias ochracea).
ACTM_STRPU **Mass:** 41530 **Score:** 41 **Matches:** 1(1) **Sequences:** 1(1)
RecName: Full=Actin, muscle; Flags: Precursor; - OS=Strongylocentrotus purpuratus (Purple sea urchin).
ACTM_STYCL **Mass:** 42040 **Score:** 41 **Matches:** 1(1) **Sequences:** 1(1)
RecName: Full=Actin, muscle; - OS=Styela clava (Sea squirt).
ACTM_STYPL **Mass:** 42327 **Score:** 41 **Matches:** 1(1) **Sequences:** 1(1)
RecName: Full=Actin, muscle; - OS=Styela plicata (Sea squirt) (Ascidia plicata).
ACTN_STYCL **Mass:** 42016 **Score:** 41 **Matches:** 1(1) **Sequences:** 1(1)
RecName: Full=Actin, muscle; - OS=Styela clava (Sea squirt).
ACTSA_TAKRU **Mass:** 41918 **Score:** 41 **Matches:** 1(1) **Sequences:** 1(1)
RecName: Full=Actin, alpha skeletal muscle A; AltName: Full=Alpha-actin-1 A; Flags: Precursor; - OS=Takifugu rubripes (Japanese pufferfi
ACTSB_TAKRU **Mass:** 41950 **Score:** 41 **Matches:** 1(1) **Sequences:** 1(1)
RecName: Full=Actin, alpha skeletal muscle B; AltName: Full=Alpha-actin-1 B; Flags: Precursor; - OS=Takifugu rubripes (Japanese pufferfi
ACTS_ATRMM **Mass:** 42034 **Score:** 41 **Matches:** 1(1) **Sequences:** 1(1)
RecName: Full=Actin, alpha skeletal muscle; AltName: Full=Alpha-actin-1; Flags: Precursor; - OS=Atractaspis microlepidota microlepidota.
ACTS_BOVIN **Mass:** 42024 **Score:** 41 **Matches:** 1(1) **Sequences:** 1(1)
RecName: Full=Actin, alpha skeletal muscle; AltName: Full=Alpha-actin-1; Flags: Precursor; - OS=Bos taurus (Bovine).
ACTS_CARAU **Mass:** 41944 **Score:** 41 **Matches:** 1(1) **Sequences:** 1(1)
RecName: Full=Actin, alpha skeletal muscle; AltName: Full=Alpha-actin-1; Flags: Precursor; - OS=Carassius auratus (Goldfish).
ACTS_CHICK **Mass:** 42024 **Score:** 41 **Matches:** 1(1) **Sequences:** 1(1)
RecName: Full=Actin, alpha skeletal muscle; AltName: Full=Alpha-actin-1; Flags: Precursor; - OS=Gallus gallus (Chicken).
ACTS_CYPCA **Mass:** 41932 **Score:** 41 **Matches:** 1(1) **Sequences:** 1(1)
RecName: Full=Actin, alpha skeletal muscle; AltName: Full=Alpha-actin-1; Flags: Precursor; - OS=Cyprinus carpio (Common carp).
ACTS_HUMAN **Mass:** 42024 **Score:** 41 **Matches:** 1(1) **Sequences:** 1(1)
RecName: Full=Actin, alpha skeletal muscle; AltName: Full=Alpha-actin-1; Flags: Precursor; - OS=Homo sapiens (Human).
ACTS_MOUSE **Mass:** 42024 **Score:** 41 **Matches:** 1(1) **Sequences:** 1(1)
RecName: Full=Actin, alpha skeletal muscle; AltName: Full=Alpha-actin-1; Flags: Precursor; - OS=Mus musculus (Mouse).
ACTS_OREMO **Mass:** 41918 **Score:** 41 **Matches:** 1(1) **Sequences:** 1(1)
RecName: Full=Actin, alpha skeletal muscle; AltName: Full=Alpha-actin-1; Flags: Precursor; - OS=Oreochromis mossambicus (Mozambique tila
ACTS_ORYLA **Mass:** 41932 **Score:** 41 **Matches:** 1(1) **Sequences:** 1(1)
RecName: Full=Actin, alpha skeletal muscle; AltName: Full=Alpha-actin-1; AltName: Full=OlMA1; Flags: Precursor; - OS=Oryzias latipes (Me
ACTS_PIG **Mass:** 42024 **Score:** 41 **Matches:** 1(1) **Sequences:** 1(1)
RecName: Full=Actin, alpha skeletal muscle; AltName: Full=Alpha-actin-1; Flags: Precursor; - OS=Sus scrofa (Pig).
ACTS_PLEWA **Mass:** 14093 **Score:** 41 **Matches:** 1(1) **Sequences:** 1(1)
RecName: Full=Actin, alpha skeletal muscle; Flags: Fragment; - OS=Pleurodeles waltl (Iberian ribbed newt).
ACTS_PONAB **Mass:** 42024 **Score:** 41 **Matches:** 1(1) **Sequences:** 1(1)
RecName: Full=Actin, alpha skeletal muscle; AltName: Full=Alpha-actin-1; Flags: Precursor; - OS=Pongo abelii (Sumatran orangutan) (Pongo
ACTS_RABIT **Mass:** 42024 **Score:** 41 **Matches:** 1(1) **Sequences:** 1(1)
RecName: Full=Actin, alpha skeletal muscle; AltName: Full=Alpha-actin-1; Flags: Precursor; - OS=Oryctolagus cuniculus (Rabbit).
ACTS_RAT **Mass:** 42024 **Score:** 41 **Matches:** 1(1) **Sequences:** 1(1)
RecName: Full=Actin, alpha skeletal muscle; AltName: Full=Alpha-actin-1; Flags: Precursor; - OS=Rattus norvegicus (Rat).
ACTY_LIMPO **Mass:** 41762 **Score:** 41 **Matches:** 1(1) **Sequences:** 1(1)
RecName: Full=Actin-11; Flags: Precursor; - OS=Limulus polyphemus (Atlantic horseshoe crab).
ACT_AJECG **Mass:** 41583 **Score:** 41 **Matches:** 1(1) **Sequences:** 1(1)
RecName: Full=Actin; - OS=Ajellomyces capsulata (strain G186AR / H82 / ATCC MYA-2454 / RMSCC 2432) (Darling's disease fungus) (Histoplas
ACT_ASHGO **Mass:** 41706 **Score:** 41 **Matches:** 1(1) **Sequences:** 1(1)
RecName: Full=Actin; - OS=Ashbya gossypii (strain ATCC 10895 / CBS 109.51 / FGSC 9923 / NRRL Y-1056) (Yeast) (Eremothecium gossypii).
ACT_BOTFU **Mass:** 41613 **Score:** 41 **Matches:** 1(1) **Sequences:** 1(1)
RecName: Full=Actin; - OS=Botryotinia fuckeliana (Noble rot fungus) (Botrytis cinerea).
ACT_BRUMA **Mass:** 41683 **Score:** 41 **Matches:** 1(1) **Sequences:** 1(1)
RecName: Full=Actin; - OS=Brugia malayi (Filarial nematode worm).
ACT_CALFI **Mass:** 31274 **Score:** 41 **Matches:** 1(1) **Sequences:** 1(1)
RecName: Full=Actin; Flags: Fragment; - OS=Calanus finmarchicus (Calanus tonsus).
ACT_CANAX **Mass:** 41726 **Score:** 41 **Matches:** 1(1) **Sequences:** 1(1)
RecName: Full=Actin; - OS=Candida albicans (Yeast).
ACT_CANDC **Mass:** 41712 **Score:** 41 **Matches:** 1(1) **Sequences:** 1(1)
RecName: Full=Actin; - OS=Candida dubliniensis (strain CD36 / ATCC MYA-646 / CBS 7987 / NCPF 3949 / NRRL Y-17841) (Yeast).
ACT_CANGA **Mass:** 41663 **Score:** 41 **Matches:** 1(1) **Sequences:** 1(1)
RecName: Full=Actin; - OS=Candida glabrata (strain ATCC 2001 / CBS 138 / JCM 3761 / NBRC 0622 / NRRL Y-65) (Yeast) (Torulopsis glabrata)
ACT_CHIOP **Mass:** 20790 **Score:** 41 **Matches:** 1(1) **Sequences:** 1(1)

RecName: Full=Actin, muscle; AltName: Full=Alpha-actin; Flags: Fragments; - OS=Chionoecetes opilio (Crab-beetle).
ACT_EXODE Mass: 41680 Score: 41 Matches: 1(1) Sequences: 1(1)
RecName: Full=Actin; - OS=Exophiala dermatitidis (Black yeast) (Wangiella dermatitidis).
ACT_GAEGA Mass: 41581 Score: 41 Matches: 1(1) Sequences: 1(1)
RecName: Full=Actin; - OS=Gaeumannomyces graminis var. avenae.
ACT_HYDVU Mass: 41770 Score: 41 Matches: 1(1) Sequences: 1(1)
RecName: Full=Actin, non-muscle 6.2; - OS=Hydra vulgaris (Hydra) (Hydra attenuata).
ACT_HYPJE Mass: 41718 Score: 41 Matches: 1(1) Sequences: 1(1)
RecName: Full=Actin; - OS=Hypocrea jecorina (Trichoderma reesei).
ACT_KLULA Mass: 41632 Score: 41 Matches: 1(1) Sequences: 1(1)
RecName: Full=Actin; - OS=Kluyveromyces lactis (strain ATCC 8585 / CBS 2359 / DSM 70799 / NBRC 1267 / NRRL Y-1140 / WM37) (Yeast) (Candi
ACT_LUMRU Mass: 41297 Score: 41 Matches: 1(1) Sequences: 1(1)
RecName: Full=Actin; Flags: Fragment; - OS=Lumbricus rubellus (Humus earthworm).
ACT_MANSE Mass: 41750 Score: 41 Matches: 1(1) Sequences: 1(1)
RecName: Full=Actin, muscle; Flags: Precursor; - OS=Manduca sexta (Tobacco hawkmoth) (Tobacco hornworm).
ACT_MAYDE Mass: 41790 Score: 41 Matches: 1(1) Sequences: 1(1)
RecName: Full=Actin; Flags: Precursor; - OS=Mayetiola destructor (Hessian fly).
ACT_NEUCR Mass: 41581 Score: 41 Matches: 1(1) Sequences: 1(1)
RecName: Full=Actin; - OS=Neurospora crassa (strain ATCC 24698 / 74-OR23-1A / CBS 708.71 / DSM 1257 / FGSC 987).
ACT_PICAD Mass: 41676 Score: 41 Matches: 1(1) Sequences: 1(1)
RecName: Full=Actin; - OS=Pichia angusta (strain ATCC 26012 / NRRL Y-7560 / DL-1) (Yeast) (Hansenula polymorpha).
ACT_PICGU Mass: 40345 Score: 41 Matches: 1(1) Sequences: 1(1)
RecName: Full=Actin; - OS=Meyerozyma guilliermondii (strain ATCC 6260 / CBS 566 / DSM 6381 / JCM 1539 / NBRC 10279 / NRRL Y-324) (Yeast)
ACT_PLAMG Mass: 41735 Score: 41 Matches: 1(1) Sequences: 1(1)
RecName: Full=Actin, adductor muscle; Flags: Precursor; - OS=Placopecten magellanicus (Sea scallop).
ACT_PROCL Mass: 36101 Score: 41 Matches: 1(1) Sequences: 1(1)
RecName: Full=Actin; Flags: Fragment; - OS=Procambarus clarkii (Red swamp crayfish).
ACT_SACBA Mass: 41663 Score: 41 Matches: 1(1) Sequences: 1(1)
RecName: Full=Actin; - OS=Saccharomyces bayanus (Yeast) (Saccharomyces uvarum).
ACT_THELA Mass: 41609 Score: 41 Matches: 1(1) Sequences: 1(1)
RecName: Full=Actin; - OS=Thermomyces lanuginosus (Humicola lanuginosa).
ACT_YEAST Mass: 41663 Score: 41 Matches: 1(1) Sequences: 1(1)
RecName: Full=Actin; - OS=Saccharomyces cerevisiae (strain ATCC 204508 / S288c) (Baker's yeast).
ACT1_ACACA Mass: 41649 Score: 41 Matches: 1(1) Sequences: 1(1)
RecName: Full=Actin-1; - OS=Acanthamoeba castellanii (Amoeba).
ACT1_AEDAE Mass: 41646 Score: 41 Matches: 1(1) Sequences: 1(1)
RecName: Full=Actin-1; Flags: Precursor; - OS=Aedes aegypti (Yellowfever mosquito) (Culex aegypti).
ACT1_ARTSX Mass: 41759 Score: 41 Matches: 1(1) Sequences: 1(1)
RecName: Full=Actin, clone 205; Flags: Precursor; - OS=Artemia sp. (Brine shrimp).
ACT1_BACDO Mass: 41673 Score: 41 Matches: 1(1) Sequences: 1(1)
RecName: Full=Actin, indirect flight muscle; Flags: Precursor; - OS=Bactrocera dorsalis (Oriental fruit fly) (Dacus dorsalis).
ACT1_BOMMO Mass: 41849 Score: 41 Matches: 1(1) Sequences: 1(1)
RecName: Full=Actin, muscle-type A1; Flags: Precursor; - OS=Bombyx mori (Silk moth).
ACT1_CAEEL Mass: 41769 Score: 41 Matches: 1(1) Sequences: 1(1)
RecName: Full=Actin-1/3; Flags: Precursor; - OS=Caenorhabditis elegans.
ACT1_DICDI Mass: 41706 Score: 41 Matches: 1(1) Sequences: 1(1)
RecName: Full=Major actin; AltName: Full=Actin A1; AltName: Full=Actin A12; AltName: Full=Actin A8; AltName: Full=Actin III; AltName: Fu
ACT1_DROME Mass: 41795 Score: 41 Matches: 1(1) Sequences: 1(1)
RecName: Full=Actin-5C; Flags: Precursor; - OS=Drosophila melanogaster (Fruit fly).
ACT1_ECHGR Mass: 41801 Score: 41 Matches: 1(1) Sequences: 1(1)
RecName: Full=Actin-1; - OS=Echinococcus granulosus.
ACT1_HALRO Mass: 42018 Score: 41 Matches: 1(1) Sequences: 1(1)
RecName: Full=Actin, muscle 1A; - OS=Halocynthia roretzi (Sea squirt).
ACT1_HELER Mass: 41761 Score: 41 Matches: 1(1) Sequences: 1(1)
RecName: Full=Actin CyI, cytoplasmic; Flags: Precursor; - OS=Heliocidaris erythrogramma (Sea urchin).
ACT1_HELTB Mass: 41761 Score: 41 Matches: 1(1) Sequences: 1(1)
RecName: Full=Actin CyI, cytoplasmic; Flags: Precursor; - OS=Heliocidaris tuberculata (Sea urchin).
ACT1_LUMTE Mass: 41819 Score: 41 Matches: 1(1) Sequences: 1(1)
RecName: Full=Actin-1; Flags: Precursor; - OS=Lumbricus terrestris (Common earthworm).
ACT1_LYTPI Mass: 41815 Score: 41 Matches: 1(1) Sequences: 1(1)
RecName: Full=Actin, cytoskeletal 1; AltName: Full=LPC1; Flags: Precursor; - OS=Lytechinus pictus (Painted sea urchin).
ACT1_ONCVO Mass: 41745 Score: 41 Matches: 1(1) Sequences: 1(1)
RecName: Full=Actin-1; Flags: Precursor; - OS=Onchocerca volvulus.
ACT1_PODCA Mass: 41740 Score: 41 Matches: 1(1) Sequences: 1(1)
RecName: Full=Actin-1/2; - OS=Podocoryne carnea.
ACT1_SACKO Mass: 41865 Score: 41 Matches: 1(1) Sequences: 1(1)
RecName: Full=Actin-1; Flags: Precursor; - OS=Saccoglossus kowalevskii (Acorn worm).
ACT1_STRFN Mass: 41800 Score: 41 Matches: 1(1) Sequences: 1(1)
RecName: Full=Actin-15A; Flags: Precursor; - OS=Strongylocentrotus franciscanus (Giant red sea urchin).
ACTB1_DANRE Mass: 41740 Score: 41 Matches: 1(1) Sequences: 1(1)
RecName: Full=Actin, cytoplasmic 1; AltName: Full=Beta-actin-1; Contains: RecName: Full=Actin, cytoplasmic 1, N-terminally processed; -
ACTB1_TAKRU Mass: 41740 Score: 41 Matches: 1(1) Sequences: 1(1)
RecName: Full=Actin, cytoplasmic 1; AltName: Full=Beta-actin A; Contains: RecName: Full=Actin, cytoplasmic 1, N-terminally processed; -
ACT2_ARTSX Mass: 41757 Score: 41 Matches: 1(1) Sequences: 1(1)
RecName: Full=Actin, clone 211; Flags: Precursor; - OS=Artemia sp. (Brine shrimp).
ACT2_BACDO Mass: 41776 Score: 41 Matches: 1(1) Sequences: 1(1)
RecName: Full=Actin-2, muscle-specific; Flags: Precursor; - OS=Bactrocera dorsalis (Oriental fruit fly) (Dacus dorsalis).
ACT2_BOMMO Mass: 41776 Score: 41 Matches: 1(1) Sequences: 1(1)
RecName: Full=Actin, muscle-type A2; Flags: Precursor; - OS=Bombyx mori (Silk moth).
ACT2_CAEEL Mass: 41751 Score: 41 Matches: 1(1) Sequences: 1(1)
RecName: Full=Actin-2; Flags: Precursor; - OS=Caenorhabditis elegans.
ACT2_HALRO Mass: 41987 Score: 41 Matches: 1(1) Sequences: 1(1)
RecName: Full=Actin, muscle 2/4/4A; - OS=Halocynthia roretzi (Sea squirt).
ACT2_LUMTE Mass: 41813 Score: 41 Matches: 1(1) Sequences: 1(1)

RecName: Full=Actin-2; - OS=Lumbricus terrestris (Common earthworm).
ACT2_LYTPI Mass: 41847 Score: 41 Matches: 1(1) Sequences: 1(1)
RecName: Full=Actin, cytoskeletal 2; AltName: Full=LPC2; Flags: Precursor; - OS=Lytechinus pictus (Painted sea urchin).
ACT2_MOLOC Mass: 42236 Score: 41 Matches: 1(1) Sequences: 1(1)
RecName: Full=Actin, muscle-type; AltName: Full=A2; - OS=Molgula oculata (Sea squirt).
ACT2_ONCVO Mass: 41792 Score: 41 Matches: 1(1) Sequences: 1(1)
RecName: Full=Actin-2; Flags: Precursor; - OS=Onchocerca volvulus.
ACT2_SACKO Mass: 41856 Score: 41 Matches: 1(1) Sequences: 1(1)
RecName: Full=Actin-2; Flags: Precursor; - OS=Saccoglossus kowalevskii (Acorn worm).
ACT2_STRFN Mass: 41772 Score: 41 Matches: 1(1) Sequences: 1(1)
RecName: Full=Actin-15B; Flags: Precursor; - OS=Strongylocentrotus franciscanus (Giant red sea urchin).
ACT2_XENLA Mass: 41962 Score: 41 Matches: 1(1) Sequences: 1(1)
RecName: Full=Actin, alpha skeletal muscle 2; AltName: Full=Actin alpha 2; Short=alpha2; Short=alpha2p; Flags: Precursor; - OS=Xenopus l
ACT2_XENTR Mass: 42006 Score: 41 Matches: 1(1) Sequences: 1(1)
RecName: Full=Actin, alpha cardiac muscle 2; AltName: Full=Actin alpha 2; Short=alpha2T; Flags: Precursor; - OS=Xenopus tropicalis (West
ACTB2_DANRE Mass: 41726 Score: 41 Matches: 1(1) Sequences: 1(1)
RecName: Full=Actin, cytoplasmic 2; AltName: Full=Beta-actin-2; Contains: RecName: Full=Actin, cytoplasmic 2, N-terminally processed; -
ACTB2_TAKRU Mass: 41740 Score: 41 Matches: 1(1) Sequences: 1(1)
RecName: Full=Actin, cytoplasmic 2; AltName: Full=Beta-actin B; Contains: RecName: Full=Actin, cytoplasmic 2, N-terminally processed; -
ACT3A_HELAM Mass: 41806 Score: 41 Matches: 1(1) Sequences: 1(1)
RecName: Full=Actin, cytoplasmic A3a; Flags: Precursor; - OS=Helicoverpa armigera (Cotton bollworm) (Heliothis armigera).
ACT3B_HELAM Mass: 41795 Score: 41 Matches: 1(1) Sequences: 1(1)
RecName: Full=Actin-A3b, cytoplasmic; Flags: Precursor; - OS=Helicoverpa armigera (Cotton bollworm) (Heliothis armigera).
ACT3_ARTSX Mass: 36867 Score: 41 Matches: 1(1) Sequences: 1(1)
RecName: Full=Actin, clone 302; Flags: Fragment; - OS=Artemia sp. (Brine shrimp).
ACT3_BACDO Mass: 41789 Score: 41 Matches: 1(1) Sequences: 1(1)
RecName: Full=Actin-3, muscle-specific; Flags: Precursor; - OS=Bactrocera dorsalis (Oriental fruit fly) (Dacus dorsalis).
ACT3_BOMMO Mass: 41891 Score: 41 Matches: 1(1) Sequences: 1(1)
RecName: Full=Actin, cytoplasmic A3; Flags: Precursor; - OS=Bombyx mori (Silk moth).
ACT3_DICDI Mass: 41814 Score: 41 Matches: 1(1) Sequences: 1(1)
RecName: Full=Actin-3; AltName: Full=Actin-3-sub 1; - OS=Dictyostelium discoideum (Slime mold).
ACT3_DROME Mass: 41808 Score: 41 Matches: 1(1) Sequences: 1(1)
RecName: Full=Actin-57B; Flags: Precursor; - OS=Drosophila melanogaster (Fruit fly).
ACT3_LIMPO Mass: 41781 Score: 41 Matches: 1(1) Sequences: 1(1)
RecName: Full=Actin-3; Flags: Precursor; - OS=Limulus polyphemus (Atlantic horseshoe crab).
ACT3_LYTPI Mass: 19367 Score: 41 Matches: 1(1) Sequences: 1(1)
RecName: Full=Actin, cytoskeletal 3; AltName: Full=LPC3; Flags: Fragment; - OS=Lytechinus pictus (Painted sea urchin).
ACT3_PODCA Mass: 41800 Score: 41 Matches: 1(1) Sequences: 1(1)
RecName: Full=Actin-3; - OS=Podocoryne carnea.
ACT3_XENLA Mass: 41957 Score: 41 Matches: 1(1) Sequences: 1(1)
RecName: Full=Actin, alpha skeletal muscle 3; AltName: Full=Actin alpha 3; AltName: Full=Femoral (alpha 3) actin; Flags: Precursor; - OS
ACT3_XENTR Mass: 41957 Score: 41 Matches: 1(1) Sequences: 1(1)
RecName: Full=Actin, alpha sarcomeric/skeletal; AltName: Full=Actin alpha 3; Flags: Precursor; - OS=Xenopus tropicalis (Western clawed f
ACTB3_TAKRU Mass: 41756 Score: 41 Matches: 1(1) Sequences: 1(1)
RecName: Full=Actin, cytoplasmic 3; AltName: Full=Beta-actin C; Contains: RecName: Full=Actin, cytoplasmic 3, N-terminally processed; -
ACT4_ARTSX Mass: 41811 Score: 41 Matches: 1(1) Sequences: 1(1)
RecName: Full=Actin, clone 403; Flags: Precursor; - OS=Artemia sp. (Brine shrimp).
ACT4_BOMMO Mass: 41795 Score: 41 Matches: 1(1) Sequences: 1(1)
RecName: Full=Actin, cytoplasmic A4; Flags: Precursor; - OS=Bombyx mori (Silk moth).
ACT4_CAEEL Mass: 41751 Score: 41 Matches: 1(1) Sequences: 1(1)
RecName: Full=Actin-4; Flags: Precursor; - OS=Caenorhabditis elegans.
ACT4_DROME Mass: 41760 Score: 41 Matches: 1(1) Sequences: 1(1)
RecName: Full=Actin, larval muscle; AltName: Full=Actin-79B; Flags: Precursor; - OS=Drosophila melanogaster (Fruit fly).
ACT4_LYTPI Mass: 17138 Score: 41 Matches: 1(1) Sequences: 1(1)
RecName: Full=Actin, cytoskeletal 4; AltName: Full=LPC4; Flags: Fragment; - OS=Lytechinus pictus (Painted sea urchin).
ACT5C_ANOGA Mass: 41795 Score: 41 Matches: 1(1) Sequences: 1(1)
RecName: Full=Actin-5C; AltName: Full=Actin-1D, cytoplasmic; Flags: Precursor; - OS=Anopheles gambiae (African malaria mosquito).
ACT5_BACDO Mass: 41744 Score: 41 Matches: 1(1) Sequences: 1(1)
RecName: Full=Actin-5, muscle-specific; Flags: Precursor; - OS=Bactrocera dorsalis (Oriental fruit fly) (Dacus dorsalis).
ACT5_CHICK Mass: 41809 Score: 41 Matches: 1(1) Sequences: 1(1)
RecName: Full=Actin, cytoplasmic type 5; - OS=Gallus gallus (Chicken).
ACT5_DROME Mass: 41775 Score: 41 Matches: 1(1) Sequences: 1(1)
RecName: Full=Actin-87E; Flags: Precursor; - OS=Drosophila melanogaster (Fruit fly).
ACT5_XENLA Mass: 41823 Score: 41 Matches: 1(1) Sequences: 1(1)
RecName: Full=Actin, cytoplasmic type 5; - OS=Xenopus laevis (African clawed frog).
ACT6_DIPDE Mass: 41497 Score: 41 Matches: 1(1) Sequences: 1(1)
RecName: Full=Actin-6; Flags: Fragment; - OS=Diphyllobothrium dendriticum (Tapeworm).
ACT6_DROME Mass: 41673 Score: 41 Matches: 1(1) Sequences: 1(1)
RecName: Full=Actin, indirect flight muscle; AltName: Full=Actin-88F; Flags: Precursor; - OS=Drosophila melanogaster (Fruit fly).
ACT6_DROSI Mass: 41673 Score: 41 Matches: 1(1) Sequences: 1(1)
RecName: Full=Actin, indirect flight muscle; AltName: Full=Actin-88F; Flags: Precursor; - OS=Drosophila simulans (Fruit fly).
ACT8_XENLA Mass: 41821 Score: 41 Matches: 1(1) Sequences: 1(1)
RecName: Full=Actin, cytoplasmic type 8; - OS=Xenopus laevis (African clawed frog).
ACT10_DICDI Mass: 41720 Score: 41 Matches: 1(1) Sequences: 1(1)
RecName: Full=Actin-10; - OS=Dictyostelium discoideum (Slime mold).
ACT17_DICDI Mass: 41545 Score: 41 Matches: 1(1) Sequences: 1(1)
RecName: Full=Actin-17; AltName: Full=Actin-2-sub 2; - OS=Dictyostelium discoideum (Slime mold).
ACT18_DICDI Mass: 42504 Score: 41 Matches: 1(1) Sequences: 1(1)
RecName: Full=Actin-18; AltName: Full=Actin-3-sub 2; - OS=Dictyostelium discoideum (Slime mold).
ACT23_DICDI Mass: 39938 Score: 41 Matches: 1(1) Sequences: 1(1)
RecName: Full=Putative actin-23; - OS=Dictyostelium discoideum (Slime mold).
ACT24_DICDI Mass: 42111 Score: 41 Matches: 1(1) Sequences: 1(1)
RecName: Full=Putative actin-24; - OS=Dictyostelium discoideum (Slime mold).
ACT25_DICDI Mass: 43188 Score: 41 Matches: 1(1) Sequences: 1(1)

RecName: Full=Putative actin-25; - OS=Dictyostelium discoideum (Slime mold).

---

9. HSLU_HAHCH **Mass:** 49552 **Score:** 37 **Matches:** 2(0) **Sequences:** 1(0)
RecName: Full=ATP-dependent protease ATPase subunit HslU; AltName: Full=Unfoldase HslU; - OS=Hahella chejuensis (strain KCTC 2396).
☐ Check to include this hit in error tolerant search or archive report

	Query	Observed	Mr(expt)	Mr(calc)	ppm	Miss	Score	Expect	Rank	Unique	Retention Time	Peptide
☑	157	523.7779	1045.5413	1045.5404	0.91	1	37	0.078	1	U	465s (7.75m)	K.LREGTLDDK.E
☑	158	523.7798	1045.5450	1045.5404	4.47	1	(25)	1.3	1	U	568s (9.47m)	K.LREGTLDDK.E

Proteins matching the same set of peptides:
HSLU_BUCBP **Mass:** 50552 **Score:** 36 **Matches:** 2(0) **Sequences:** 1(0)
RecName: Full=ATP-dependent protease ATPase subunit HslU; AltName: Full=Unfoldase HslU; - OS=Buchnera aphidicola subsp. Baizongia pistac

---

10. TOM1_ASHGO **Score:** 35 **Matches:** 8(0) **Sequences:** 2(0)
RecName: Full=Probable E3 ubiquitin-protein ligase TOM1; EC=6.3.2.- OS=Ashbya gossypii (strain ATCC 10895 / CBS 109.51 / FGSC 9923 / NRR
☐ Check to include this hit in error tolerant search or archive report

Query	Observed	Mr(expt)	Mr(calc)	ppm	Miss	Score	Expect	Rank	Unique	Retention Time	Peptide
49	421.7572	841.4999	841.5021	-2.60	0	(23)	1.4	2	U	679s (11.32m)	R.IGLSSIPR.L
50	421.7572	841.4999	841.5021	-2.60	0	(26)	0.76	2	U	676s (11.27m)	R.IGLSSIPR.L
51	421.7586	841.5027	841.5021	0.73	0	30	0.3	2	U	767s (12.78m)	R.IGLSSIPR.L
52	421.7591	841.5036	841.5021	1.70	0	(27)	0.54	2	U	492s (8.20m)	R.IGLSSIPR.L
53	421.7591	841.5036	841.5021	1.70	0	(27)	0.64	2	U	495s (8.25m)	R.IGLSSIPR.L
54	421.7592	841.5038	841.5021	2.01	0	(27)	0.59	2	U	588s (9.80m)	R.IGLSSIPR.L
55	421.7592	841.5038	841.5021	2.01	0	(27)	0.55	2	U	585s (9.75m)	R.IGLSSIPR.L
175	**549.7428**	**1097.4711**	**1097.4924**	**-19.36**	**1**	**5**	**25**	**4**	**U**	**477s (7.95m)**	**K.MNVERYDR.A + Oxidation (M)**

---

11. K1C10_BOVIN **Mass:** 54815 **Score:** 35 **Matches:** 1(0) **Sequences:** 1(0) **emPAI:** 0.06
RecName: Full=Keratin, type I cytoskeletal 10; AltName: Full=Cytokeratin VIB; AltName: Full=Cytokeratin-10; Short=CK-10; AltName: Full=C
☐ Check to include this hit in error tolerant search or archive report

	Query	Observed	Mr(expt)	Mr(calc)	ppm	Miss	Score	Expect	Rank	Unique	Retention Time	Peptide
☑	15	516.3038	1030.5929	1030.5910	1.87	0	35	0.24	1	U	637s (10.62m)	R.VLDELTLTK.T

Proteins matching the same set of peptides:
K1C10_CANFA **Mass:** 57676 **Score:** 35 **Matches:** 1(0) **Sequences:** 1(0)
RecName: Full=Keratin, type I cytoskeletal 10; AltName: Full=Cytokeratin-10; Short=CK-10; AltName: Full=Epithelial keratin-10; AltName:
K1C10_HUMAN **Mass:** 58792 **Score:** 35 **Matches:** 1(0) **Sequences:** 1(0)
RecName: Full=Keratin, type I cytoskeletal 10; AltName: Full=Cytokeratin-10; Short=CK-10; AltName: Full=Keratin-10; Short=K10; - OS=Homo
K1C15_SHEEP **Mass:** 48740 **Score:** 35 **Matches:** 1(0) **Sequences:** 1(0)
RecName: Full=Keratin, type I cytoskeletal 15; AltName: Full=Cytokeratin-15; Short=CK-15; AltName: Full=Keratin-15; Short=K15; - OS=Ovis

---

12. SYGB_AROAE **Mass:** 75809 **Score:** 33 **Matches:** 2(0) **Sequences:** 1(0)
RecName: Full=Glycine--tRNA ligase beta subunit; EC=6.1.1.14; AltName: Full=Glycyl-tRNA synthetase beta subunit; Short=GlyRS; - OS=Aroma
☐ Check to include this hit in error tolerant search or archive report

	Query	Observed	Mr(expt)	Mr(calc)	ppm	Miss	Score	Expect	Rank	Unique	Retention Time	Peptide
	114	**478.3013**	**954.5880**	**954.5862**	**1.91**	**0**	**(26)**	**0.38**	**2**	**U**	**619s (10.32m)**	**R.LAVTVPTVR.A**
☑	115	478.3013	954.5880	954.5862	1.91	0	33	0.077	1	U	614s (10.23m)	R.LAVTVPTVR.A

---

13. MLE_DICDI **Score:** 33 **Matches:** 2(0) **Sequences:** 1(0)
RecName: Full=Myosin, essential light chain; AltName: Full=EMLC; AltName: Full=Myosin light chain alkali OS=Dictyostelium discoideum (Sl
☐ Check to include this hit in error tolerant search or archive report

Query	Observed	Mr(expt)	Mr(calc)	ppm	Miss	Score	Expect	Rank	Unique	Retention Time	Peptide
201	581.2890	1160.5634	1160.5860	-19.46	0	(30)	0.28	2	U	582s (9.70m)	K.VSVEDIGACLR.S
202	581.2890	1160.5634	1160.5860	-19.44	0	33	0.16	2	U	586s (9.77m)	K.VSVEDIGACLR.S

---

14. NDST_CAEEL **Mass:** 98998 **Score:** 32 **Matches:** 1(0) **Sequences:** 1(0)
RecName: Full=Bifunctional heparan sulfate N-deacetylase/N-sulfotransferase 1; EC=2.8.2.8; AltName: Full=Glucosaminyl N-deacetylase/N-su
☐ Check to include this hit in error tolerant search or archive report

	Query	Observed	Mr(expt)	Mr(calc)	ppm	Miss	Score	Expect	Rank	Unique	Retention Time	Peptide
☑	200	578.8327	1155.6509	1155.6724	-18.57	1	32	0.25	1	U	449s (7.48m)	K.NLPVLTTSRR.G

---

15. PANB_ACIAC **Mass:** 30838 **Score:** 32 **Matches:** 2(0) **Sequences:** 1(0)
RecName: Full=3-methyl-2-oxobutanoate hydroxymethyltransferase; EC=2.1.2.11; AltName: Full=Ketopantoate hydroxymethyltransferase; Short=
☐ Check to include this hit in error tolerant search or archive report

	Query	Observed	Mr(expt)	Mr(calc)	ppm	Miss	Score	Expect	Rank	Unique	Retention Time	Peptide
☑	42	412.7536	823.4927	823.5028	-12.24	0	32	0.077	1	U	528s (8.80m)	R.RPVSLPR.L
☑	43	412.7536	823.4927	823.5028	-12.24	0	(23)	0.58	1	U	531s (8.85m)	R.RPVSLPR.L

Proteins matching the same set of peptides:
PANB_ACISJ **Mass:** 30989 **Score:** 32 **Matches:** 2(0) **Sequences:** 1(0)
RecName: Full=3-methyl-2-oxobutanoate hydroxymethyltransferase; EC=2.1.2.11; AltName: Full=Ketopantoate hydroxymethyltransferase; Short=
PANB_DELAS **Mass:** 30863 **Score:** 32 **Matches:** 2(0) **Sequences:** 1(0)
RecName: Full=3-methyl-2-oxobutanoate hydroxymethyltransferase; EC=2.1.2.11; AltName: Full=Ketopantoate hydroxymethyltransferase; Short=

16. TGL_BACP2 **Mass:** 28393 **Score:** 30 **Matches:** 1(0) **Sequences:** 1(0) **emPAI:** 0.12
RecName: Full=Protein-glutamine gamma-glutamyltransferase; EC=2.3.2.13; AltName: Full=Transglutaminase; Short=TGase; - OS=Bacillus pumil
☐ Check to include this hit in error tolerant search or archive report

Query	Observed	Mr(expt)	Mr(calc)	ppm	Miss	Score	Expect	Rank	Unique	Retention Time	Peptide
399	755.7228	2264.1465	2264.1583	-5.18	1	30	0.41	1	U	820s (13.67m)	R.QASDLLFEVTLRSNIMNAAR.D + Oxidation (M)

17. ISPT_CHLTE **Mass:** 30521 **Score:** 30 **Matches:** 1(0) **Sequences:** 1(0) **emPAI:** 0.11
RecName: Full=Isoprenyl transferase; EC=2.5.1.-; - OS=Chlorobium tepidum (strain ATCC 49652 / DSM 12025 / TLS).
☐ Check to include this hit in error tolerant search or archive report

Query	Observed	Mr(expt)	Mr(calc)	ppm	Miss	Score	Expect	Rank	Unique	Retention Time	Peptide
215	595.8220	1189.6293	1189.6190	8.68	0	30	0.45	1	U	781s (13.02m)	K.TLDETIELTR.K

18. SPZ1A_WHEAT **Score:** 29 **Matches:** 2(0) **Sequences:** 1(0)
RecName: Full=Serpin-Z1A; AltName: Full=TriaeZ1a; AltName: Full=WSZ1a; Short=WSZ1; AltName: Full=WSZCI OS=Triticum aestivum (Wheat)
☐ Check to include this hit in error tolerant search or archive report

Query	Observed	Mr(expt)	Mr(calc)	ppm	Miss	Score	Expect	Rank	Unique	Retention Time	Peptide
168	539.7912	1077.5679	1077.5488	17.7	0	(9)	47	4	U	530s (8.83m)	-.MATTLATDVR.L
169	539.7912	1077.5679	1077.5488	17.7	0	29	0.48	2	U	526s (8.77m)	-.MATTLATDVR.L

Proteins matching the same set of peptides:
SPZ1B_WHEAT **Score:** 29 **Matches:** 2(0) **Sequences:** 1(0)
RecName: Full=Serpin-Z1B; AltName: Full=TriaeZ1b; AltName: Full=WSZ1b; AltName: Full=WZS2 OS=Triticum aestivum (Wheat)
SPZ1C_WHEAT **Score:** 29 **Matches:** 2(0) **Sequences:** 1(0)
RecName: Full=Serpin-Z1C; AltName: Full=TriaeZ1c; AltName: Full=WSZ1c OS=Triticum aestivum (Wheat)
SPZ2A_WHEAT **Score:** 29 **Matches:** 2(0) **Sequences:** 1(0)
RecName: Full=Serpin-Z2A; AltName: Full=TriaeZ2a; AltName: Full=WSZ2a OS=Triticum aestivum (Wheat)
SPZ2B_WHEAT **Score:** 29 **Matches:** 2(0) **Sequences:** 1(0)
RecName: Full=Serpin-Z2B; AltName: Full=TriaeZ2b; AltName: Full=WSZ2b; AltName: Full=WZS3 OS=Triticum aestivum (Wheat)
SPZ4_HORVU **Score:** 29 **Matches:** 2(0) **Sequences:** 1(0)
RecName: Full=Serpin-Z4; AltName: Full=BSZ4; AltName: Full=HorvuZ4; AltName: Full=Major endosperm albumin; AltName: Full=Protein Z4; Sho

19. LEU3B_ASPNG **Mass:** 40100 **Score:** 28 **Matches:** 1(0) **Sequences:** 1(0)
RecName: Full=3-isopropylmalate dehydrogenase B; Short=3-IPM-DH B; Short=IMDH B; EC=1.1.1.85; AltName: Full=Beta-IPM dehydrogenase B; -
☐ Check to include this hit in error tolerant search or archive report

Query	Observed	Mr(expt)	Mr(calc)	ppm	Miss	Score	Expect	Rank	Unique	Retention Time	Peptide
61	428.7665	855.5185	855.5178	0.90	0	28	0.43	1	U	544s (9.07m)	R.LAAELALR.H

20. ARGR_SHEFN **Mass:** 16955 **Score:** 28 **Matches:** 2(0) **Sequences:** 1(0)
RecName: Full=Arginine repressor; - OS=Shewanella frigidimarina (strain NCIMB 400).
☐ Check to include this hit in error tolerant search or archive report

Query	Observed	Mr(expt)	Mr(calc)	ppm	Miss	Score	Expect	Rank	Unique	Retention Time	Peptide
207	588.8136	1175.6126	1175.6034	7.83	0	(24)	1.9	1	U	761s (12.68m)	K.TIDETLETVR.S
208	588.8136	1175.6126	1175.6034	7.83	0	28	0.76	1	U	764s (12.73m)	K.TIDETLETVR.S

Peptide matches not assigned to protein hits: (no details means no match)

Query	Observed	Mr(expt)	Mr(calc)	ppm	Miss	Score	Expect	Rank	Unique	Retention Time	Peptide
30	393.7459	785.4772	785.4872	-12.66	1	28	0.96	1		486s (8.10m)	VATLRAR
198	576.7982	1151.5818	1151.6009	-16.59	0	27	0.61	1		682s (11.37m)	MAVQQYGVLK + Oxidation (M)
114	478.3013	954.5880	954.5862	1.92	0	26	0.34	1		619s (10.32m)	LVDGIGILR
256	426.9125	1277.7156	1277.7204	-3.77	1	25	0.74	1		549s (9.15m)	LSDRVTVVHPR
88	450.2681	898.5216	898.5236	-2.20	1	25	0.86	1		529s (8.82m)	TAVKEVPR
397	747.3808	2239.1207	2239.1386	-7.98	0	24	1.4	1		721s (12.02m)	HEVVGSGVLIEELWADHPPR
278	659.3709	1316.7273	1316.7122	11.5	0	24	1.6	1		1150s (19.17m)	TLDLAMANSLLR
277	659.3709	1316.7273	1316.7122	11.5	0	23	1.7	1		1146s (19.10m)	TLDLAMANSLLR
37	404.2386	806.4626	806.4538	11.0	0	23	1.3	1		509s (8.48m)	IAASLGIY
166	536.7863	1071.5580	1071.5672	-8.58	0	23	2	1		510s (8.50m)	LNSPATLNSR
128	492.2548	982.4949	982.4794	15.9	0	22	1.4	1		478s (7.97m)	TIIFDSGCK
108	475.7289	949.4432	949.4426	0.58	0	20	2.2	1		720s (12.00m)	NDLTLDML + Oxidation (M)
64	430.2231	858.4316	858.4276	4.70	0	19	3.4	1		652s (10.87m)	GFFYTPK
364	598.9436	1793.8090	1793.7924	9.27	0	19	1.4	1		779s (12.98m)	DGMQEEAIQEIAGMTR + Oxidation (M)
7	412.7108	823.4070	823.4222	-18.43	1	18	3.1	1		512s (8.53m)	ISSMSKR + Oxidation (M)
365	598.9436	1793.8090	1793.8028	3.47	0	18	1.9	1		775s (12.92m)	TGTTGQSGAESGTTEPSAR
48	421.2595	840.5044	840.5181	-16.30	1	18	2.7	1		787s (13.12m)	VKAASLPR
39	404.2386	806.4626	806.4538	11.0	0	17	4.9	1		505s (8.42m)	IAASLGIY
86	449.7537	897.4928	897.4920	0.88	0	17	3.2	1		697s (11.62m)	EPTVLPSR
235	615.8856	1229.7567	1229.7383	14.9	0	17	1.1	1		583s (9.72m)	KPLEIINIYK
127	491.2726	980.5305	980.5291	1.49	0	17	5.2	1		515s (8.58m)	TLLSSFASR
59	427.7581	853.5017	853.5022	-0.52	0	17	2.3	1		581s (9.68m)	IIGVSTHK
66	431.7753	861.5361	861.5324	4.32	0	17	1.5	1		628s (10.47m)	IVYNILK
191	567.8313	1133.6480	1133.6332	13.1	0	17	5.2	1		787s (13.12m)	EVVLEIAFSK
214	592.8365	1183.6585	1183.6561	2.06	1	17	6.4	1		582s (9.70m)	LKTVEEAAAPR
102	467.7332	933.4518	933.4589	-7.62	1	16	7.8	1		602s (10.03m)	KVGENMEK
224	602.3340	1202.6534	1202.6731	-16.35	1	16	12	1		806s (13.43m)	TLRSSNIGSIR
213	592.8365	1183.6585	1183.6673	-7.44	1	16	7.6	1		586s (9.77m)	RATLATLADPR

☑	38	404.2386	806.4626	806.4538	11.0	0	16	7	1	502s (8.37m)	IAASLGIY
☑	268	655.3241	1308.6337	1308.6422	-6.50	0	16	8	1	590s (9.83m)	VAEHNDIAVDAR
☑	87	449.7537	897.4928	897.4920	0.88	0	15	4.8	1	701s (11.68m)	EPTVLPSR
☑	248	422.5829	1264.7270	1264.7213	4.53	1	15	4.9	1	947s (15.78m)	VKYMATQLLAK
☑	126	491.2726	980.5305	980.5403	-9.98	0	15	7.8	1	520s (8.67m)	TLLDHAGVR
☑	369	901.9712	1801.9279	1801.9243	1.98	1	15	13	1	789s (13.15m)	EILLQEEEKMIQQR + Oxidation (M)
☑	269	655.3241	1308.6337	1308.6534	-15.08	1	15	8.8	1	592s (9.87m)	EQHRIDEVQR
☑	398	747.3808	2239.1207	2239.1286	-3.55	1	15	12	1	724s (12.07m)	HNGFNKVWHIEGGIIEYAR
☑	217	596.3179	1190.6213	1190.6403	-15.98	0	15	14	1	680s (11.33m)	TLGTPIAMMIK + Oxidation (M)
☑	16	518.7756	1035.5366	1035.5349	1.65	0	15	12	1	491s (8.18m)	VTAAAFSNGAK
☑	164	534.7662	1067.5177	1067.5335	-14.72	1	15	7.5	1	479s (7.98m)	RWFCLTSR
☑	181	557.3373	1112.6600	1112.6666	-5.91	1	15	9.6	1	603s (10.05m)	VDRGITLALR
☑	216	596.3179	1190.6213	1190.6295	-6.94	1	15	16	1	683s (11.38m)	EGRDFIEVVK
☑	138	503.7642	1005.5139	1005.5091	4.77	0	15	14	1	634s (10.57m)	TGSLTDLSGR
☑	57	425.7613	849.5081	849.4960	14.3	1	14	6.8	1	431s (7.18m)	KIIEYGK
☑	281	667.3704	1332.7263	1332.7071	14.4	0	14	17	1	1061s (17.68m)	TLDLAMANSLLR + Oxidation (M)
☑	342	804.9031	1607.7916	1607.8090	-10.78	2	14	14	1	941s (15.68m)	MNIERADFVDRVK + Oxidation (M)
☑	262	649.8755	1297.7365	1297.7466	-7.79	2	14	12	1	649s (10.82m)	IKNEANNRIVK
☑	323	758.4133	1514.8120	1514.7940	11.9	0	14	18	1	517s (8.62m)	VTLQEGATVEEAIR
☑	1	382.2206	1143.6401	1143.6499	-8.56	2	14	35	1	719s (11.98m)	QELEIKKEK
☑	368	599.2760	1794.8062	1794.8319	-14.32	1	14	5	1	782s (13.03m)	HYMRSQTVEGVTDTR + Oxidation (M)
☑	284	446.5323	1336.5751	1336.5817	-4.89	0	14	3.2	1	432s (7.20m)	NSDMDTVVPESK + Oxidation (M)
☑	109	475.7289	949.4432	949.4426	0.58	0	13	11	1	716s (11.93m)	NDLTLDML + Oxidation (M)
☑	312	475.9223	1424.7452	1424.7273	12.6	1	13	15	1	506s (8.43m)	AFNRSSHITQHK
☑	336	783.8740	1565.7335	1565.7243	5.88	0	13	10	1	584s (9.73m)	GCPVTAATSDLTSESK
☑	246	629.3353	1256.6561	1256.6585	-1.94	1	13	18	1	844s (14.07m)	NVVSIGENRNR
☑	177	550.2337	1098.4528	1098.4620	-8.38	1	13	1.5	1	484s (8.07m)	AKMMSCADSR
☑	137	502.7993	1003.5840	1003.5702	13.7	0	13	13	1	561s (9.35m)	FANAELVIK
☑	56	425.7613	849.5081	849.4960	14.3	1	13	8.8	1	434s (7.23m)	KIIEYGK
☑	190	567.8313	1133.6480	1133.6332	13.1	0	13	12	1	784s (13.07m)	EVVLEIAFSK
☑	110	476.2824	950.5502	950.5371	13.7	0	13	8.7	1	612s (10.20m)	MPPAPAIVR
☑	261	649.8755	1297.7365	1297.7428	-4.85	0	13	15	1	646s (10.77m)	LMNPTAVIGLAAK
☑	146	512.2416	1022.4687	1022.4637	4.90	0	13	11	1	496s (8.27m)	AAQMMGINR + 2 Oxidation (M)
☑	392	738.0367	2211.0882	2211.0875	0.32	2	13	19	1	708s (11.80m)	MGKDTLSEIVTSIRNADMAK + 2 Oxidation (M)
☑	359	857.4111	1712.8076	1712.8113	-2.16	0	13	13	1	743s (12.38m)	MAGMQLDEIIEYLR + 2 Oxidation (M)
☑	211	590.2950	1178.5754	1178.5788	-2.84	0	13	17	1	480s (8.00m)	VGGTMVQVGMGK + Oxidation (M)
☑	116	478.3156	954.6166	954.6226	-6.19	1	12	5.2	1	1019s (16.98m)	EALKLLIR
☑	187	565.7717	1129.5289	1129.5325	-3.19	1	12	9.4	1	592s (9.87m)	KESYESMIK + Oxidation (M)
☑	161	531.7965	1061.5784	1061.5982	-18.62	1	12	26	1	1039s (17.32m)	ARDVLYLGR
☑	194	574.8132	1147.6119	1147.5945	15.1	2	12	26	1	448s (7.47m)	NANSASKSKNK
☑	12	463.8074	925.6002	925.6073	-7.66	1	12	6.1	1	836s (13.93m)	ILRNGLLK
☑	160	529.7812	1057.5479	1057.5516	-3.48	1	12	22	1	500s (8.33m)	AINVAGRDDK
☑	76	881.9270	1761.8395	1761.8434	-2.21	1	12	18	1	730s (12.17m)	YVAFSASFRSEAGSAGR
☑	366	897.9125	1793.8105	1793.8362	-14.30	0	12	7.7	1	762s (12.70m)	VLLMDEPMGALDAMTR + 2 Oxidation (M)
☑	26	385.2034	768.3922	768.3878	5.71	1	12	12	1	525s (8.75m)	EKAHER
☑	373	617.6576	1849.9511	1849.9455	3.03	1	12	29	1	460s (7.67m)	AKTSVQAGEIIDSSVMSK
☑	91	454.2328	906.4511	906.4487	2.61	0	11	29	1	605s (10.08m)	SYFLSYK
☑	367	897.9125	1793.8105	1793.8028	4.31	0	11	9.5	1	765s (12.75m)	TGTTGQSGAESGTTEPSAR
☑	335	783.8740	1565.7335	1565.7429	-5.98	2	11	19	1	587s (9.78m)	EMDAIQAKEEMKK + Oxidation (M)
☑	103	467.7332	933.4518	933.4338	19.3	1	11	27	1	605s (10.08m)	KMNEAGER
☑	223	598.3022	1194.5899	1194.5856	3.63	0	11	27	1	615s (10.25m)	WLVMDAASFR
☑	395	742.7083	2225.1030	2225.1249	-9.85	2	11	31	1	717s (11.95m)	EGTISSAIGKKVLEDMFAEGK + Oxidation (M)
☑	147	512.2416	1022.4687	1022.4637	4.90	0	11	18	1	493s (8.22m)	AAQMMGINR + 2 Oxidation (M)
☑	68	432.7490	863.4835	863.4865	-3.44	0	11	30	1	528s (8.80m)	VATLAAYR
☑	282	667.3704	1332.7263	1332.7071	14.4	0	10	38	1	1064s (17.73m)	TLDLAMANSLLR + Oxidation (M)
☑	295	453.9266	1358.7579	1358.7632	-3.87	2	10	34	1	441s (7.35m)	KMKYLGVYLTK + Oxidation (M)
☑	343	804.9031	1607.7916	1607.7647	16.8	1	10	34	1	938s (15.63m)	MKNNLINMDEINK + 2 Oxidation (M)
☑	175	549.7428	1097.4711	1097.4812	-9.14	0	10	8	1	477s (7.95m)	MTDNFADLR + Oxidation (M)
☑	362	896.9027	1791.7909	1791.8250	-19.05	0	10	9.2	1	887s (14.78m)	AGFDENVAVMGEWVPR + Oxidation (M)
☑	219	596.3196	1190.6246	1190.6116	11.0	2	10	47	1	778s (12.97m)	SKSSQGASGARR
☑	351	819.9179	1637.8211	1637.8447	-14.37	1	10	39	1	590s (9.83m)	LAKMGIQVAQDGTYK + Oxidation (M)
☑	133	496.7983	991.5821	991.5927	-10.70	1	10	18	1	442s (7.37m)	AARIVPHTK
☑	19	665.2960	664.2888	664.2962	-11.27	0	10	28	1	556s (9.27m)	MTAGNR + Oxidation (M)
☑	180	557.3373	1112.6600	1112.6666	-5.91	2	10	31	1	606s (10.10m)	LGTVVNNRKL
☑	346	812.3996	1622.7846	1622.7635	13.0	0	10	35	1	905s (15.08m)	NTSVSTTTSPDVEGTK
☑	370	902.4607	1802.9068	1802.8873	10.9	0	9	47	1	762s (12.70m)	MANDLLDFAEIQQAPK
☑	176	550.2336	1098.4527	1098.4612	-7.72	0	9	3.8	1	479s (7.98m)	CSTSSTPSSSR
☑	293	677.3979	1352.7812	1352.8027	-15.93	0	9	23	1	957s (15.95m)	VVDILALGQNIAK
☑	67	432.7490	863.4835	863.4725	12.7	2	9	40	1	532s (8.87m)	RQKYNR
☑	111	477.8204	953.6262	953.6386	-12.90	1	9	5.7	1	1013s (16.88m)	GKALALLLR
☑	96	461.2790	920.5435	920.5443	-0.91	1	9	22	1	439s (7.32m)	IPIEKHGK
☑	283	668.3321	1334.6496	1334.6538	-3.19	2	9	39	1	653s (10.88m)	DASASVDSRGRSK
☑	47	421.2595	840.5044	840.5181	-16.30	1	9	19	1	783s (13.05m)	VKAASLPR
☑	182	560.2985	1118.5824	1118.5720	9.33	1	9	55	1	1002s (16.70m)	KSWITEAER
☑	225	603.8209	1205.6273	1205.6074	16.5	0	9	54	1	684s (11.40m)	AVAMSSTDGLVR
☑	344	811.9104	1621.8062	1621.7916	8.99	2	9	49	1	963s (16.05m)	DLNKEVDRLMSMR + Oxidation (M)
☑	139	503.7642	1005.5139	1005.5277	-13.73	1	9	51	1	631s (10.52m)	LMKTGANAGK + Oxidation (M)
☑	360	857.4111	1712.8076	1712.8113	-2.16	0	9	30	1	740s (12.33m)	MAGMQLDEIIEYLR + 2 Oxidation (M)
☑	159	527.7790	1053.5434	1053.5528	-8.99	0	9	39	1	657s (10.95m)	SAAIMYAVTK
☑	89	901.9676	1801.9206	1801.8846	20.0	1	9	55	1	792s (13.20m)	TPVNFEDKEIGETPAR
☑	121	484.7301	967.4457	967.4359	10.1	0	9	20	1	509s (8.48m)	VDNSYNTR
☑	239	624.8537	1247.6928	1247.7159	-18.54	1	9	46	1	828s (13.80m)	KVITTEIMSVK
☑	90	453.7514	905.4882	905.4752	14.3	2	8	59	1	798s (13.30m)	MEKASGRK
☑	36	404.2341	806.4536	806.4399	17.0	1	8	46	1	750s (12.50m)	LPKAAHGN
☑	230	606.8056	1211.5966	1211.5895	5.88	0	8	38	1	623s (10.38m)	IGGGPGDAADVQR
☑	317	752.8692	1503.7238	1503.7351	-7.51	2	8	42	1	820s (13.67m)	DVKRQDLEQMDK

☑	58	426.2468	850.4791	850.4661	15.3	2	8	28	1	810s (13.50m)	YGKKAER
☑	21	706.8571	1411.6997	1411.6732	18.8	0	8	46	1	784s (13.07m)	DGIDLSTVQWAAH
☑	152	522.7778	1043.5411	1043.5611	-19.17	0	8	59	1	602s (10.03m)	ALDAGQTLQK
☑	318	752.8692	1503.7238	1503.7351	-7.51	2	8	43	1	819s (13.65m)	DVKRQDLEQMDK
☑	356	849.4469	1696.8792	1696.8970	-10.48	1	8	60	1	647s (10.78m)	NGFIKALYEMVQIR + Oxidation (M)
☑	245	629.3353	1256.6561	1256.6612	-4.08	2	8	61	1	847s (14.12m)	EKEDDKVLPGK
☑	77	881.9300	1761.8455	1761.8434	1.16	1	8	48	1	727s (12.12m)	YVAFSASFRSEAGSAGR
☑	72	435.7568	869.4991	869.5123	-15.23	0	8	28	1	694s (11.57m)	WLVTPVR
☑	62	429.2134	856.4123	856.4014	12.8	0	8	23	1	1459s (24.32m)	AMHHAFK + Oxidation (M)
☑	363	896.9027	1791.7909	1791.7945	-2.02	0	8	16	1	883s (14.72m)	EVDGGDGGCISLEDLASR
☑	384	983.9832	1965.9519	1965.9322	10.0	1	7	57	1	848s (14.13m)	DALLCDAMTLVNLRGCDK + Oxidation (M)
☑	231	610.3347	1218.6549	1218.6720	-14.05	1	7	78	1	704s (11.73m)	KPEELHKNPK
☑	302	695.8486	1389.6826	1389.6697	9.23	0	7	61	1	788s (13.13m)	VDLIEPESDMVK + Oxidation (M)
☑	385	983.9832	1965.9519	1965.9465	2.74	1	7	59	1	845s (14.08m)	FGTEELARIEGEMLEAR + Oxidation (M)
☑	315	735.8639	1469.7132	1469.6899	15.9	1	7	55	1	664s (11.07m)	DYGGNDRITFGQK
☑	353	827.8904	1653.7662	1653.7781	-7.15	0	7	35	1	785s (13.08m)	EPSATPGAQMGVPPGSR + Oxidation (M)
☑	63	429.2135	856.4125	856.4014	13.0	0	7	25	1	1455s (24.25m)	AMHHAFK + Oxidation (M)
☑	142	508.2904	1014.5663	1014.5822	-15.67	0	7	93	1	659s (10.98m)	TASVLVGAAAR
☑	241	625.8223	1249.6299	1249.6302	-0.20	1	7	73	1	473s (7.88m)	AAAYAAQEAEKK
☑	319	753.3414	1504.6682	1504.6690	-0.52	0	7	19	1	815s (13.58m)	VQMFESNGSFLCK + Oxidation (M)
☑	220	596.8115	1191.6085	1191.6135	-4.21	0	7	80	1	674s (11.23m)	YDGLIEQLGGK
☑	226	603.8209	1205.6273	1205.6438	-13.69	1	7	85	1	680s (11.33m)	TIGDTLRCTVK
☑	140	505.2516	1008.4885	1008.4989	-10.22	0	7	55	1	472s (7.87m)	LVDPSHGER
☑	195	575.3192	1148.6238	1148.6302	-5.56	1	7	87	1	553s (9.22m)	SRSIYSAPIR
☑	377	935.9570	1869.8995	1869.9366	-19.86	1	7	57	1	838s (13.97m)	LPEMQERTRPEAIER + Oxidation (M)
☑	25	385.2034	768.3922	768.3878	5.71	1	7	39	1	527s (8.78m)	EKAHER
☑	144	509.8049	1017.5952	1017.5971	-1.89	1	7	56	1	761s (12.68m)	AGGGKYKPLK
☑	238	624.8537	1247.6928	1247.7098	-13.68	1	7	71	1	832s (13.87m)	AKNGHLSVVAPR
☑	285	446.8619	1337.5640	1337.5632	0.60	0	7	9.1	1	435s (7.25m)	GPMPPDTMFSDK + Oxidation (M)
☑	74	437.2206	872.4266	872.4352	-9.81	1	7	40	1	604s (10.07m)	ARENPSSL
☑	240	625.8223	1249.6299	1249.6303	-0.25	0	7	83	1	470s (7.83m)	FEQNTVQGSIK
☑	354	564.6334	1690.8784	1690.8930	-8.65	0	7	1e+02	1	487s (8.12m)	TIFESLDIGWELLR
☑	44	413.2389	824.4632	824.4756	-15.04	1	6	34	1	678s (11.30m)	ILGPPKSN
☑	143	508.2942	1014.5738	1014.5934	-19.33	1	6	1.2e+02	1	671s (11.18m)	SLALSAAARR
☑	199	577.2945	1152.5745	1152.5882	-11.93	0	6	73	1	956s (15.93m)	MAASATTMLLK + Oxidation (M)
☑	17	522.7801	1043.5456	1043.5611	-14.88	0	6	88	1	600s (10.00m)	ALDAGQTLQK
☑	288	451.9342	1352.7808	1352.7565	18.0	2	6	47	1	1010s (16.83m)	SHFLKDTLKHK
☑	242	627.8347	1253.6547	1253.6689	-11.30	2	6	74	1	660s (11.00m)	MLSDYKEKIK
☑	150	518.3169	1034.6193	1034.6012	17.5	0	6	23	1	761s (12.68m)	TELYALVVK
☑	186	565.7717	1129.5289	1129.5186	9.11	0	6	39	1	589s (9.82m)	TANSGMSPTHK
☑	113	478.2533	954.4920	954.4957	-3.82	0	6	50	1	519s (8.65m)	GGPMHTVLK + Oxidation (M)
☑	73	437.2206	872.4266	872.4352	-9.84	0	6	47	1	601s (10.02m)	VQDEGVAR
☑	2	391.2054	1170.5945	1170.5716	19.5	1	6	1.2e+02	1	571s (9.52m)	GMRRPYGYR + Oxidation (M)
☑	326	763.8719	1525.7292	1525.7494	-13.21	1	6	64	1	824s (13.73m)	CDRGLAQCHTVPVK
☑	227	606.3134	1210.6123	1210.6202	-6.56	0	6	82	1	606s (10.10m)	LIGCPVCIEHK
☑	254	639.3063	1276.5980	1276.6160	-14.11	1	6	51	1	788s (13.13m)	AAGQNARGEFEK
☑	255	639.3063	1276.5980	1276.6160	-14.11	1	6	51	1	790s (13.17m)	AAGQNARGEFEK
☑	80	443.7650	885.5154	885.5283	-14.64	0	6	1.3e+02	1	710s (11.83m)	AAATAAAVLK
☑	382	647.6516	1939.9330	1939.9422	-4.73	2	6	75	1	772s (12.87m)	SFSSAVKNELCRVETDR
☑	376	935.9570	1869.8995	1869.9052	-3.04	2	6	75	1	841s (14.02m)	SHDCKYIVKCLGCFVR
☑	387	985.4452	1968.8759	1968.8418	17.3	1	6	30	1	756s (12.60m)	MTDTQNPKSSNQMWGGR + 2 Oxidation (M)
☑	107	472.2886	942.5627	942.5611	1.72	1	6	1.1e+02	1	930s (15.50m)	TLTPSLRR
☑	383	647.6516	1939.9330	1939.9422	-4.73	2	6	77	1	776s (12.93m)	SFSSAVKNELCRVETDR
☑	307	466.6099	1396.8078	1396.8190	-8.04	2	5	57	1	1023s (17.05m)	QQAKLGWKLIVN
☑	141	505.2516	1008.4885	1008.4989	-10.22	0	5	80	1	468s (7.80m)	LVDPSHGER
☑	294	677.3979	1352.7812	1352.7564	18.3	0	5	57	1	955s (15.92m)	AGNQLFHAAALLK
☑	272	437.2589	1308.7548	1308.7514	2.64	1	5	46	1	979s (16.32m)	HSKKPSTLGINK
☑	34	401.7202	801.4259	801.4344	-10.64	1	5	1.5e+02	1	508s (8.47m)	DALEKAR
☑	303	695.8486	1389.6826	1389.7099	-19.70	2	5	1e+02	1	791s (13.18m)	EVVESEGERKTK
☑	345	811.9104	1621.8062	1621.7981	4.97	1	5	1.2e+02	1	958s (15.97m)	MSTIAKDQTQINEK + Oxidation (M)
☑	347	812.8982	1623.7818	1623.7966	-9.11	1	5	81	1	837s (13.95m)	YRMDPPSEIYPLK + Oxidation (M)
☑	390	1042.0116	2082.0086	2082.0197	-5.35	2	5	94	1	812s (13.53m)	TAMSSNLELASAEVMKRGR + 2 Oxidation (M)
☑	316	746.3461	1490.6776	1490.7035	-17.39	0	5	41	1	690s (11.50m)	SIAVMLQDDAADAR + Oxidation (M)
☑	393	742.3800	2224.1183	2224.1236	-2.37	1	5	1.2e+02	1	734s (12.23m)	EHVALELNTLGDPAERAAYR
☑	212	590.2958	1178.5771	1178.5568	17.2	0	5	1.1e+02	1	469s (7.82m)	GENGFVGDLGSK
☑	402	429.2453	2569.4280	2569.4421	-5.49	0	5	45	1	532s (8.87m)	LLGPLPFPHHPIHTVPSEEVLLK
☑	394	742.3800	2224.1183	2224.1205	-0.98	1	5	1.2e+02	1	737s (12.28m)	VHRLMSGPLGGDQQMGSLVAR + Oxidation (M)
☑	374	927.9582	1853.9018	1853.9280	-14.14	2	5	1.1e+02	1	1100s (18.33m)	LPETYKCVRCGLGYPR
☑	98	465.2866	928.5587	928.5454	14.3	1	5	1.3e+02	1	846s (14.10m)	IPSSITRR
☑	299	691.3722	1380.7298	1380.7289	0.66	0	5	1.1e+02	1	603s (10.05m)	LYPISYEQGLAK
☑	60	428.2621	854.5097	854.4974	14.5	1	5	49	1	869s (14.48m)	RLNPVEK
☑	148	514.2999	1026.5852	1026.5974	-11.86	2	5	86	1	936s (15.60m)	KAFIAKVHN
☑	401	429.2453	2569.4280	2569.4088	7.45	2	5	48	1	532s (8.87m)	ANRTSASAARGLGLAQSLPIFAEIR
☑	104	467.7372	933.4599	933.4590	1.00	0	5	1.1e+02	1	503s (8.38m)	IDMINQGK + Oxidation (M)
☑	404	894.1390	2679.3950	2679.4021	-2.62	1	5	1.1e+02	1	1788s (29.80m)	GKHKPVYTPHVDTGDYLVVINAEK
☑	3	391.2266	1170.6581	1170.6509	6.10	0	5	2.3e+02	1	662s (11.03m)	AAGPVHLHLEK
☑	324	758.8696	1515.7247	1515.7391	-9.53	2	4	86	1	948s (15.80m)	DKGEKNFAMSYVK
☑	249	422.5829	1264.7270	1264.7213	4.53	1	4	62	1	954s (15.90m)	VKYMATQLLAK
☑	290	451.9342	1352.7808	1352.8027	-16.22	1	4	71	1	998s (16.63m)	ELKGTKPVEKPK
☑	165	536.2929	1070.5713	1070.5833	-11.17	1	4	1.2e+02	1	507s (8.45m)	TGHSVTSVRK
☑	371	913.8998	1825.7851	1825.8040	-10.35	1	4	20	1	802s (13.37m)	LDAYKADDATMGEGPEK + Oxidation (M)
☑	352	820.4065	1638.7985	1638.8188	-12.43	1	4	1.3e+02	1	894s (14.90m)	FPRMTDYVVPSGVR + Oxidation (M)
☑	243	628.3848	1254.7550	1254.7408	11.3	1	4	65	1	935s (15.58m)	VVTAALEIQRR
☑	155	523.3051	1044.5956	1044.5815	13.5	1	4	1.6e+02	1	777s (12.95m)	KASDGIVEVK
☑	92	456.7719	911.5292	911.5440	-16.17	1	4	60	1	811s (13.52m)	KQAPVELK
☑	101	465.7785	929.5425	929.5545	-13.00	1	4	1.7e+02	1	714s (11.90m)	EITAKIQK

☑	134	500.7994	999.5843	999.5825	1.84	2	4	1.6e+02	1	906s (15.10m)	EIKEAVRR
☑	4	391.2267	780.4389	780.4494	-13.38	1	4	2.7e+02	1	667s (11.12m)	KAPPSGPK
☑	204	582.2859	1162.5572	1162.5726	-13.27	0	4	1.1e+02	1	756s (12.60m)	LDVMQLADMK
☑	228	606.3713	1210.7281	1210.7285	-0.29	1	4	60	1	864s (14.40m)	LKIPVIASNEK
☑	350	819.9179	1637.8211	1637.8447	-14.37	1	4	1.6e+02	1	594s (9.90m)	LAKMGIQVAQDGTYK + Oxidation (M)
☑	330	768.8244	1535.6342	1535.6596	-16.50	0	4	12	1	901s (15.02m)	MEDLNANDMDAAVK
☑	379	941.4268	1880.8391	1880.8495	-5.55	0	4	49	1	763s (12.72m)	MAGLIQSGMDEGEAIESK + Oxidation (M)
☑	313	728.3598	1454.7051	1454.7010	2.87	1	4	1.2e+02	1	616s (10.27m)	YKMNICNKPSNK + Oxidation (M)
☑	337	785.3328	1568.6511	1568.6752	-15.36	0	4	14	1	874s (14.57m)	YHETPGMAMDVFR + Oxidation (M)
☑	325	758.8696	1515.7247	1515.7537	-19.16	1	4	1.1e+02	1	945s (15.75m)	MQKPCKENEGKPK
☑	5	391.2270	1170.6593	1170.6509	7.12	0	4	2.6e+02	1	644s (10.73m)	AAGPVHLHLEK
☑	291	451.9342	1352.7808	1352.7564	18.0	2	4	88	1	1001s (16.68m)	NHEIKPKKVFN
☑	236	619.8708	1237.7270	1237.7030	19.4	1	3	68	1	890s (14.83m)	YTILTSSAVRK
☑	244	628.3848	1254.7551	1254.7772	-17.60	2	3	77	1	937s (15.62m)	EIGALVRTKLR
☑	355	849.4469	1696.8792	1696.8858	-3.85	1	3	1.8e+02	1	650s (10.83m)	TQKIWSAYLEAIMK + Oxidation (M)
☑	380	970.9718	1939.9291	1939.9639	-17.95	1	3	1.2e+02	1	766s (12.77m)	GNEYEALTSPQTSFRLK
☑	403	894.1390	2679.3950	2679.4103	-5.71	1	3	1.5e+02	1	1791s (29.85m)	MMFQFLSQSFFCVVGLLIRILR + 2 Oxidation (M)
☑	35	404.2341	806.4536	806.4650	-14.12	2	3	1.6e+02	1	751s (12.52m)	AIAKYKN
☑	193	572.7418	1143.4691	1143.4900	-18.31	0	3	16	1	465s (7.75m)	MSMDSVISSR + 2 Oxidation (M)
☑	297	683.7951	1365.5756	1365.5830	-5.40	0	3	24	1	982s (16.37m)	LENCDLENSSSR
☑	82	448.2152	894.4159	894.4097	6.95	0	3	97	1	533s (8.88m)	HGQWDPR
☑	95	457.2657	912.5169	912.5141	3.10	1	3	1e+02	1	711s (11.85m)	IGPDLSRR
☑	8	426.2445	850.4744	850.4661	9.75	1	3	2.8e+02	1	777s (12.95m)	TYIGKNR
☑	218	596.3196	1190.6246	1190.6263	-1.44	2	3	2.3e+02	1	774s (12.90m)	DRAMKMLQAK
☑	386	984.9444	1967.8742	1967.8564	9.05	0	3	52	1	753s (12.55m)	LGLDMSQTMAIGDAENDR + 2 Oxidation (M)
☑	311	472.2848	1413.8325	1413.8191	9.52	2	3	98	1	1026s (17.10m)	ELKALAKALGASDK
☑	124	487.7917	973.5689	973.5709	-2.02	2	3	1.5e+02	1	738s (12.30m)	LFSHKKSK
☑	85	448.2601	894.5056	894.4997	6.66	0	3	97	1	814s (13.57m)	MFLSILR + Oxidation (M)
☑	292	451.9342	1352.7808	1352.7564	18.0	2	2	1.1e+02	1	1005s (16.75m)	NHEIKPKKVFN
☑	120	484.7301	967.4457	967.4545	-9.14	1	2	82	1	513s (8.55m)	EAKNTFCR
☑	81	443.7651	885.5156	885.4994	18.4	0	2	2.8e+02	1	706s (11.77m)	MVIPDALK
☑	375	927.9582	1853.9018	1853.9345	-17.66	1	2	1.9e+02	1	1103s (18.38m)	IFAVADSMEPYALRQK + Oxidation (M)
☑	304	698.3497	1394.6849	1394.6726	8.83	1	2	1.9e+02	1	739s (12.32m)	MFYDMKLYLR + Oxidation (M)
☑	320	755.3217	1508.6289	1508.6540	-16.68	1	2	22	1	759s (12.65m)	DYICEFCARSFR
☑	247	630.8529	1259.6913	1259.6721	15.2	0	2	2.4e+02	1	1029s (17.15m)	GALLVAGTTSDAGK
☑	372	913.8998	1825.7851	1825.7941	-4.93	0	2	33	1	799s (13.32m)	AGTPMHEVNSWPDEEK
☑	331	771.3253	1540.6361	1540.6610	-16.14	0	2	20	1	812s (13.53m)	MTCSQTLGNNNTNK + Oxidation (M)
☑	13	470.2741	1407.8004	1407.7874	9.21	1	2	2.2e+02	1	840s (14.00m)	RQIYIQTFLLN
☑	361	890.9033	1779.7920	1779.8057	-7.71	2	2	61	1	768s (12.80m)	VMRQASVDDSREEDK + Oxidation (M)
☑	173	545.3182	1088.6219	1088.6342	-11.34	2	2	2.3e+02	1	804s (13.40m)	KAFVAVVKNGG
☑	162	531.8184	1061.6223	1061.6233	-0.94	2	2	1.3e+02	1	784s (13.07m)	YTPKIAKNK
☑	301	694.4250	1386.8354	1386.8156	14.3	1	2	62	1	1003s (16.72m)	IESLIKNLVLMV + Oxidation (M)
☑	381	970.9718	1939.9291	1939.9639	-17.95	1	2	1.8e+02	1	769s (12.82m)	GNEYEALTSPQTSFRLK
☑	209	589.3461	1176.6777	1176.6866	-7.57	1	2	1.5e+02	1	855s (14.25m)	TYEKLLLAAR
☑	40	412.7470	823.4795	823.4664	15.9	2	2	95	1	786s (13.10m)	KHGIQKN
☑	172	544.2328	1086.4510	1086.4479	2.83	0	2	26	1	469s (7.82m)	DNDGHSTWR
☑	135	500.7994	999.5843	999.5713	13.0	1	2	2.7e+02	1	910s (15.17m)	ILVGEVTRN
☑	265	650.7891	1299.5636	1299.5733	-7.51	2	1	49	1	470s (7.83m)	MTRERMMGEK + 2 Oxidation (M)
☑	289	451.9342	1352.7808	1352.7704	7.69	0	1	1.4e+02	1	996s (16.60m)	TYLAVTYGKPIK
☑	118	478.7863	955.5580	955.5451	13.5	1	1	2e+02	1	878s (14.63m)	ISPRTPASK
☑	6	393.2099	1176.6080	1176.6285	-17.42	1	1	4.8e+02	1	755s (12.58m)	DLRLASSIMR + Oxidation (M)
☑	310	466.6099	1396.8078	1396.7827	18.0	1	1	1.5e+02	1	1028s (17.13m)	LDAIRFIVVTHN
☑	321	755.3334	1508.6522	1508.6540	-1.21	1	1	57	1	785s (13.08m)	DYICEFCARSFR
☑	131	496.3053	990.5960	990.6087	-12.74	1	1	89	1	760s (12.67m)	HTPIRVIR
☑	170	540.3317	1078.6488	1078.6287	18.6	1	1	72	1	790s (13.17m)	GKPFFKSLR
☑	314	735.3662	1468.7179	1468.7020	10.8	1	1	2.2e+02	1	677s (11.28m)	MFSGEKINWTEK
☑	14	501.2915	1500.8527	1500.8453	4.98	1	1	5.9e+02	1	758s (12.63m)	IFWNILKLQNVN
☑	179	553.8313	1105.6480	1105.6318	14.7	1	1	99	1	808s (13.47m)	LLSFMLRIN
☑	156	523.3051	1044.5956	1044.6152	-18.74	2	1	3.2e+02	1	780s (13.00m)	ISSRIASRR
☑	106	470.2728	938.5311	938.5226	9.14	0	1	1.2e+02	1	875s (14.58m)	FKPVYTGK
☑	24	382.2204	762.4262	762.4137	16.4	1	1	3.1e+02	1	723s (12.05m)	TYPARR
☑	185	564.2776	1126.5406	1126.5407	-0.07	0	1	1.6e+02	1	516s (8.60m)	WAESGQPGAPK
☑	84	448.2601	894.5056	894.4997	6.66	1	1	1.5e+02	1	810s (13.50m)	KLVFNMK + Oxidation (M)
☑	279	663.8974	1325.7802	1325.7554	18.7	0	1	1.9e+02	1	940s (15.67m)	ILNLINTLEGLN
☑	237	619.8708	1237.7270	1237.7030	19.4	1	0	1.3e+02	1	885s (14.75m)	YTILTSSAVRK
☑	305	699.4110	1396.8075	1396.7827	17.8	1	0	1.9e+02	1	993s (16.55m)	YTTIALRYAVAR
☑	300	694.4250	1386.8354	1386.8156	14.3	1	0	88	1	1006s (16.77m)	IESLIKNLVLMV + Oxidation (M)
☑	378	936.4719	1870.9293	1870.9512	-11.72	1	0	3.3e+02	1	1107s (18.45m)	NLIEWLPGANMWAGKR + Oxidation (M)
☑	20	665.7612	1329.5079	1329.5185	-8.00	0	0	11	1	791s (13.18m)	QEMLLDTCCCR + Oxidation (M)
☑	253	635.8052	1269.5959	1269.5846	8.94	0	0	1.8e+02	1	464s (7.73m)	MAQHEVSMPPK + Oxidation (M)
☑	29	393.2098	784.4050	784.4079	-3.70	0	0	2e+02	1	758s (12.63m)	SPQAVVAGG
☑	9	435.2710	434.2638							998s (16.63m)	
☑	10	448.1196	447.1123							952s (15.87m)	
☑	11	463.8068	462.7995							1839s (30.65m)	
☑	18	611.3535	610.3462							877s (14.62m)	
☑	27	390.7335	779.4524							757s (12.62m)	
☑	28	390.7335	779.4524							754s (12.57m)	
☑	32	399.2469	796.4792							751s (12.52m)	
☑	33	399.2469	796.4792							755s (12.58m)	
☑	41	412.7470	823.4795							783s (13.05m)	
☑	45	413.2389	824.4632							675s (11.25m)	
☑	46	415.2263	828.4380							787s (13.12m)	
☑	65	431.2636	860.5126							437s (7.28m)	
☑	69	434.7601	867.5056							810s (13.50m)	
☑	70	434.7601	867.5056							813s (13.55m)	
☑	71	435.2522	868.4898							700s (11.67m)	

☑	75	441.2423	880.4701	533s (8.88m)
☑	78	443.2725	884.5304	813s (13.55m)
☑	79	443.2725	884.5304	817s (13.62m)
☑	83	448.2152	894.4159	529s (8.82m)
☑	93	456.7724	911.5303	843s (14.05m)
☑	94	457.2657	912.5169	707s (11.78m)
☑	97	463.8081	925.6016	1845s (30.75m)
☑	99	465.2866	928.5587	850s (14.17m)
☑	100	465.7785	929.5425	716s (11.93m)
☑	105	467.7372	933.4599	499s (8.32m)
☑	112	478.2533	954.4920	523s (8.72m)
☑	117	478.7863	955.5580	875s (14.58m)
☑	119	483.2745	964.5344	504s (8.40m)
☑	122	487.3000	972.5855	879s (14.65m)
☑	123	487.3000	972.5855	882s (14.70m)
☑	125	487.7917	973.5689	735s (12.25m)
☑	129	492.2868	982.5590	906s (15.10m)
☑	130	492.2868	982.5590	909s (15.15m)
☑	132	496.3053	990.5960	764s (12.73m)
☑	136	501.2912	1000.5678	752s (12.53m)
☑	145	509.8049	1017.5952	752s (12.53m)
☑	149	514.2999	1026.5852	940s (15.67m)
☑	151	518.3183	1034.6220	784s (13.07m)
☑	153	522.8124	1043.6103	943s (15.72m)
☑	163	531.8184	1061.6223	787s (13.12m)
☑	167	538.6957	1075.3768	497s (8.28m)
☑	171	540.3317	1078.6489	796s (13.27m)
☑	174	545.3182	1088.6219	801s (13.35m)
☑	178	553.8313	1105.6480	805s (13.42m)
☑	183	562.3446	1122.6746	814s (13.57m)
☑	184	562.3446	1122.6746	811s (13.52m)
☑	188	567.3315	1132.6485	879s (14.65m)
☑	189	567.3315	1132.6485	876s (14.60m)
☑	196	575.8450	1149.6755	835s (13.92m)
☑	197	575.8450	1149.6755	827s (13.78m)
☑	203	582.2859	1162.5572	759s (12.65m)
☑	205	584.3582	1166.7017	846s (14.10m)
☑	206	584.3585	1166.7024	852s (14.20m)
☑	210	589.3461	1176.6777	858s (14.30m)
☑	221	597.8572	1193.6998	869s (14.48m)
☑	222	597.8572	1193.6998	872s (14.53m)
☑	229	606.3713	1210.7281	866s (14.43m)
☑	234	611.3585	1220.7024	880s (14.67m)
☑	250	633.3711	1264.7277	907s (15.12m)
☑	251	633.3711	1264.7277	903s (15.05m)
☑	252	635.8052	1269.5959	466s (7.77m)
☑	257	427.8271	1280.4596	532s (8.87m)
☑	258	428.0588	1281.1546	536s (8.93m)
☑	259	641.8849	1281.7552	908s (15.13m)
☑	260	641.8857	1281.7568	911s (15.18m)
☑	263	650.3974	1298.7802	921s (15.35m)
☑	264	650.3976	1298.7806	918s (15.30m)
☑	266	650.7891	1299.5636	467s (7.78m)
☑	270	655.3837	1308.7529	928s (15.47m)
☑	271	655.3837	1308.7529	932s (15.53m)
☑	273	437.2589	1308.7548	971s (16.18m)
☑	274	437.2589	1308.7548	968s (16.13m)
☑	275	437.2589	1308.7548	981s (16.35m)
☑	276	437.2589	1308.7548	974s (16.23m)
☑	280	663.8974	1325.7802	944s (15.73m)
☑	286	672.4108	1342.8071	971s (16.18m)
☑	287	672.4108	1342.8071	976s (16.27m)
☑	296	683.7951	1365.5756	985s (16.42m)
☑	298	685.9106	1369.8067	972s (16.20m)
☑	306	699.4110	1396.8075	999s (16.65m)
☑	308	466.6099	1396.8078	1033s (17.22m)
☑	309	466.6099	1396.8078	1025s (17.08m)
☑	322	755.8691	1509.7236	687s (11.45m)
☑	327	764.3525	1526.6904	821s (13.68m)
☑	328	768.3187	1534.6229	966s (16.10m)
☑	329	768.3187	1534.6229	969s (16.15m)
☑	332	771.3253	1540.6361	809s (13.48m)
☑	333	779.3261	1556.6377	825s (13.75m)
☑	334	779.3261	1556.6377	822s (13.70m)
☑	338	785.8105	1569.6064	898s (14.97m)
☑	339	795.7818	1589.5491	870s (14.50m)
☑	340	795.7818	1589.5491	867s (14.45m)
☑	348	542.6332	1624.8779	1143s (19.05m)
☑	349	542.6332	1624.8779	1139s (18.98m)
☑	357	425.7604	1699.0125	531s (8.85m)
☑	358	427.8217	1707.2575	531s (8.85m)
☑	388	665.2890	1992.8453	768s (12.80m)
☑	389	1042.0116	2082.0086	815s (13.58m)
☑	391	426.7117	2128.5221	531s (8.85m)
☑	396	746.0529	2235.1370	494s (8.23m)
☑	400	759.0300	2274.0682	539s (8.98m)

☑	405	*927.3742*	*2779.1008*	*450s (7.50m)*
☑	406	*981.3927*	*2941.1562*	*451s (7.52m)*

Search Parameters

```
Type of search         : MS/MS Ion Search
Enzyme                 : Trypsin
Variable modifications : Oxidation (M)
Mass values            : Monoisotopic
Protein Mass           : Unrestricted
Peptide Mass Tolerance : ± 20 ppm
Fragment Mass Tolerance: ± 0.2 Da
Max Missed Cleavages   : 2
Instrument type        : ESI-QUAD-TOF
Number of queries      : 406
```

Mascot: http://www.matrixscience.com/

Supplementary Table 5. Differentially expressed genes in GCTM-5 positive and negative subpopulations of SW1990 and CFPAC-1 pancreatic ductal adenocarcinoma cell lines

Antibodies to a CA 19-9 Related Antigen Complex Identify a Subset of SOX9 Expressing Cells In Human Foetal Pancreas and Pancreatic Adenocarcinoma. Alison M. Farley, David R. Braxton, Jonathan Li, Karl Trounson, Subhanwita Sakar-Dey, Bhavana Nayer, Tatsuhiko Ikeda, Kevin X. Lau, Winita Hardikar, Kouichi Hasegawa, and Martin F. Pera

rownames(d	SW1990_neg	SW1990_pos	CFPAC-1_neg	CFPAC-1_pos	SW1990 p.valu	log2 foldchar	CFPAC-1 p.valu	log2 foldchange (CFPAC-1)
KRT6A	4.73891675	60.8080177	0.13172684	5.53789097	1.6646E-88	3.68163234	3.00E-56	5.39371538
SPTSSB	0.10200905	0.3115095	0.11321707	3.74686319	0.00526281	1.61057894	1.70E-25	5.04851984
ALPP	2.6380685	13.2758061	0.01063424	0.34579831	2.5296E-20	2.33124553	1.99E-05	5.02314239
SYT16	0.10693628	0.58996144	0.01066048	0.32949422	9.0091E-07	2.46386928	2.66E-07	4.94990922
ALPPL2	0.58823119	7.35038932	0.00611234	0.16835835	1.1749E-17	3.64336547	5.38E-05	4.78366641
PTPRR	1.91330384	5.85636576	0.09470408	2.27873588	3.7667E-14	1.61393966	1.41E-17	4.58866332
EYA1	0.02679477	0.54233035	0.00375556	0.08156446	4.0789E-21	4.33914874	9.82E-05	4.44083961
ARHGAP40	1.82600868	14.1316386	0.07550032	1.52863234	3.0689E-30	2.95216323	2.78E-13	4.33961487
IVL	0.4295109	6.08247145	0.09548324	1.5151928	6.3656E-26	3.82389099	4.18E-15	3.98811001
PGBD5	0.40709548	0.78488984	0.00961475	0.14522042	0.00378292	0.94712298	0.00107282	3.91685186
IL1RN	17.5711984	141.858425	0.16646585	2.39470188	5.2046E-32	3.01316734	5.12E-11	3.8465479
SERPINB2	17.738483	103.128498	0.00790471	0.10358924	1.9538E-44	2.53948851	0.00771142	3.71201702
FST	4.16847679	22.7939006	0.15179998	1.93723864	7.5531E-43	2.45105562	5.49E-14	3.67375818
NCCRP1	0.07449807	3.01475212	0.01545279	0.19442359	3.9085E-19	5.33869245	0.00140901	3.65326388
CCL28	1.90051055	11.3890594	1.66407345	20.1984202	9.0002E-15	2.58318967	4.84E-38	3.60145144
MYB	0.05267623	0.23868802	0.04348872	0.46846346	3.6655E-05	2.17990221	9.84E-10	3.42922332
ZNF385B	0.13382256	0.43832716	0.02403181	0.25039795	9.9986E-05	1.71168671	1.84E-05	3.38120545
HES4	6.23183856	11.4827206	3.36266948	34.6128502	1.6192E-05	0.88173473	2.71E-06	3.36362886
GAS6	13.7676636	28.3475238	0.13286657	1.34021144	4.6537E-14	1.04193896	2.13E-08	3.33441058
LRRC26	0.10662002	0.60454927	0.09176804	0.91184837	1.5964E-05	2.5033816	0.00054541	3.31273017
IL1R2	0.05479013	0.89132613	0.13884903	1.37831826	4.7663E-10	4.02396544	4.32E-08	3.31132
C4orf19	0.59461285	3.7530839	0.08260245	0.80702687	3.8325E-29	2.658054	5.47E-17	3.28836018
FUT3	1.12924636	29.2596135	0.45701319	4.456802	5.1102E-45	4.69547854	2.76E-27	3.28570115
KRT6B	0.21411665	3.48319692	0.01400751	0.13412056	9.4963E-30	4.02394317	0.00426314	3.25925804
NPNT	0	0.04200622	0.18341276	1.70721508	0.00219711	1	3.71E-13	3.21847894
NID2	2.70559785	7.58834643	0.38989313	3.36932356	1.5433E-17	1.48783813	9.49E-29	3.11130833
SPRR3	0.53138813	17.7802132	1.74494126	13.8238232	1.4612E-47	5.0643628	6.33E-15	2.9859063
OR4C6	0.56261938	1.37594383	0.06960385	0.49467989	0.00123751	1.29019041	0.0003437	2.82925626
VTCN1	0.01100722	0.61744875	1.15787111	8.10823536	2.6112E-12	5.80979778	9.15E-15	2.80791331
SYK	0.00539266	0.06731489	0.11176749	0.72143088	0.0041714	3.64185725	1.69E-11	2.69036053
GJB2	2.27400024	6.23384644	0.66539608	4.23777471	2.3821E-07	1.45489021	5.83E-14	2.67102163
CXCL5	276.763169	436.125796	36.5941362	230.584225	0.00073669	0.65609236	2.31E-24	2.65560942
ARL14	7.2553054	24.8549754	4.58262568	28.4617471	5.1914E-25	1.77642643	9.05E-35	2.63477787
VEPH1	0.42727267	2.24608077	0.43389563	2.64118591	1.8446E-24	2.39418087	1.20E-23	2.60576588
ABCA12	0.06317665	0.23630031	0.01392221	0.08194185	0.00134054	1.90315811	0.00012526	2.55721224
TH	0.10066611	0.67940797	0.15693829	0.92238556	1.179E-05	2.75470006	7.78E-09	2.55517254
CST6	14.5899458	86.5455027	3.28496662	18.6015636	1.4439E-41	2.56848433	6.93E-12	2.50147328
NTNG1	0.69367255	1.93920238	0.93158671	5.24439354	7.9417E-07	1.48313668	9.89E-13	2.49301399
LRRN4	0.07946475	0.9200604	2.40959469	13.5302312	1.2682E-11	3.53334171	5.82E-21	2.48932409
ZBED2	5.92924706	59.4406592	2.83389833	15.9062399	1.6321E-49	3.3255293	1.35E-26	2.48873293
CYBB	0.01038896	0.12770783	0.02273587	0.12385095	2.4111E-06	3.61972354	0.00031004	2.44556264
CTPS	10.2460657	13.9384519	2.60524816	14.1699448	0.00828974	0.44400028	2.24E-35	2.44334143
FER1L6	0	0.03420777	0.02997482	0.16010186	0.00125793	1	0.00026265	2.41716707
TFCP2L1	0.13616168	0.95860914	0.317716	1.68715831	1.4809E-19	2.81562192	7.56E-12	2.40878572
CDC25A	6.64846362	11.4998212	1.18959694	6.29942669	2.0378E-06	0.79051853	2.22E-23	2.40474769
MEOX1	6.39612717	20.4777946	0.06807109	0.35747787	3.0501E-08	1.67878982	0.00592663	2.39273976
AIF1L	10.8643273	29.0886205	2.60316256	13.61697	3.4242E-21	1.42085603	4.78E-17	2.3870684
RIMS2	0.12370952	0.3922272	0.00886127	0.04547384	0.00078052	1.66473303	0.01169813	2.35945133
GINS2	28.297724	40.3522686	5.30186287	26.5441407	0.00173013	0.51196377	1.75E-26	2.32382218
MCM5	8.15936698	11.6130507	1.91423178	9.53496401	0.00851658	0.50921788	5.44E-20	2.31646197
FAM111B	18.3400447	31.1332109	9.66907924	47.9288649	4.2737E-08	0.76345722	1.69E-45	2.30944436
CDC6	28.5866505	49.9588973	10.9528779	54.238533	1.921E-08	0.80540005	1.15E-47	2.30800816
LINC00261	0.17118821	0.4273251	0.05038424	0.24840406	9.3903E-05	1.31975076	0.00332807	2.30164429
KRT81	57.6761155	265.288648	56.4170361	270.037917	7.9303E-34	2.20151703	5.93E-10	2.25895922
MCM10	7.69797114	10.5370539	1.88066832	8.70298161	0.00354861	0.45292139	2.03E-31	2.21026431
JPH1	3.63905301	13.4985545	1.76171843	8.0591677	1.5511E-36	1.89116995	3.30E-27	2.19364749
E2F1	46.1505421	63.9404747	7.8915107	35.8547087	0.00047716	0.47038186	6.85E-33	2.18378919
MCM4	37.9755352	51.8677336	8.36200513	37.9741519	0.00289446	0.44976703	5.25E-47	2.18309691
CD83	1.68157465	2.89658681	1.98105229	8.82750606	0.00414049	0.78454108	7.46E-26	2.15573894
LINC00460	5.52012592	11.8661668	0.18429279	0.81190474	1.2896E-06	1.10408089	0.00053726	2.13931083
MARC1	6.17268575	18.8137012	2.22222248	9.33472292	3.9034E-20	1.60781345	9.56E-27	2.07060394
UHRF1	10.1161373	14.2278322	2.36480059	9.9185286	0.0057431	0.49205735	1.02E-29	2.06840758
PCNA-AS1	140.211513	199.87615	60.4171676	251.874612	0.00028017	0.51150151	1.49E-28	2.05967525
FUT9	0	0.01431983	0.02324379	0.09506276	0.00355007	1	0.00141274	2.03203485
INHBB	8.2304021	16.3478939	6.6068914	26.8908117	5.1199E-08	0.99006996	1.31E-23	2.02506977
C16orf59	9.96432061	13.3228477	1.8941217	7.64298128	0.01027084	0.41905914	1.03E-11	2.01260647
PLA2G4F	0.00928931	0.83552091	0.30035611	1.20351692	1.2987E-27	6.49096083	2.23E-09	2.00251051
LOC728643	18.5123535	38.3860223	12.8162524	50.8937382	8.4691E-06	1.05209275	7.01E-21	1.9895137
TMEM160	8.71397514	14.0762335	3.87105087	15.2462004	0.00620912	0.69185844	0.00017726	1.97765257
MIR205HG	2.54673765	20.15686	0.27275844	1.07285409	6.8675E-27	2.98454866	0.00332526	1.97575816
MCM2	24.9651965	34.8493722	10.0978882	39.1495051	0.00057998	0.4812144	3.08E-39	1.95494046
SLC43A3	39.1996477	56.3384821	1.69235802	6.49336468	1.8638E-05	0.52328001	1.24E-11	1.93993143
TTYH1	0.05179366	0.50799888	0.05190079	0.19904633	6.1077E-06	3.29397781	0.00684706	1.9392759
PCNA	97.7238244	129.043123	41.3630454	158.030793	0.0035913	0.40107104	4.34E-37	1.93379139
NMU	2.79706225	10.7535559	0.81487372	3.10375549	2.6039E-15	1.94282953	8.45E-05	1.9293665
GLDC	0.23475669	0.71054898	0.42698745	1.59273709	1.2391E-06	1.59776783	3.54E-10	1.89924257
SLC4A11	4.5779076	14.0605157	0.89353458	3.32554021	6.8669E-18	1.61888926	3.39E-15	1.89599325
SNORD125	10.5872493	20.1896141	1.39472395	5.14048908	0.01008586	0.93128552	0.00652471	1.88192602
ERVMER34-1	0	0.09866236	1.99769723	7.35906483	0.00219147	1	2.49E-17	1.8811845
PIGW	33.3492779	45.5934677	5.26864461	19.2647817	0.00576616	0.45117162	1.79E-26	1.87046206
KCP	0.17241481	2.65999224	0.94301356	3.44217572	1.4515E-50	3.94746641	2.11E-10	1.86797033
SPRR1B	2.72837301	115.805939	1.60492571	5.83668935	4.7748E-58	5.40752453	1.21E-06	1.86264377
SNORA76	7.68464321	16.8002167	2.35427732	8.52325378	0.00037269	1.12842966	3.10E-05	1.85612002
CDK5R1	4.70554045	6.72174511	0.49948688	1.80803242	0.00047239	0.5144754	2.77E-08	1.85590188
MIR3653	9.38722791	18.2596835	1.35579707	4.90603662	0.00919525	0.95989067	0.00662256	1.85541674
STRA13	45.9998151	70.7269246	24.0592502	86.6593677	2.8993E-06	0.62063147	6.37E-24	1.84876403
ST6GALNAC5	0	0.20817815	1.97694533	7.04194587	1.2396E-05	1	1.90E-09	1.83270116
ARHGEF26	3.48693314	7.55451797	1.40189778	4.97584973	3.8797E-06	1.115381	1.81E-16	1.82756175
FIGN	3.34819718	5.81734695	0.6796202	2.39167427	7.7509E-06	0.79697686	1.97E-13	1.81522028
ALPK3	0.33982697	1.18883611	1.59733141	5.61270562	4.5933E-16	1.8066776	1.03E-22	1.81303272
MCM6	9.90604345	13.4621521	5.23013401	18.3310713	0.00268312	0.44252821	2.63E-27	1.80937128
SAA2	10.5832553	19.5666367	0.52159252	1.82764403	3.8167E-06	0.88661234	0.00061414	1.80899001
DSCC1	7.80949732	11.7359274	2.21762017	7.63088025	0.00125542	0.58763025	2.58E-19	1.7828372
MCM3	41.3141188	58.5294696	14.6505036	50.2240171	0.0012473	0.50252831	1.30E-24	1.77742716
F3	182.896324	691.4037	139.780584	475.123662	8.2228E-46	1.91850225	1.14E-17	1.76513908
ESCO2	12.2090231	16.3198446	2.50757718	8.51602198	0.00609702	0.41867956	2.60E-20	1.76388557
SLC9A2	0.43503916	1.22304891	0.75742609	2.55183681	3.4991E-09	1.49126491	1.61E-13	1.75235905
NOX5	0.07006198	0.30910512	0.05638894	0.18880875	1.2095E-05	2.14139394	0.00881946	1.74344152

rownames(d	SW1990_neg	SW1990_pos	CFPAC-1_neg	CFPAC-1_pos	SW1990 p.valu	log2 foldchar	CFPAC-1 p.valu	log2 foldchange (CFPAC-1)
PLA2G4F	0.00928931	0.83552091	0.30035611	1.20351692	1.2987E-27	6.49096083	2.23E-09	2.00251051
VTCN1	0.01100722	0.61744875	1.15787111	8.10823536	2.6112E-12	5.80979778	9.15E-15	2.80791331
FUT6	0.01034054	0.56882534	0.1555352	0.35483626	7.9695E-20	5.7816026	0.00501559	1.18991231
BSPRY	0.16377536	7.25620538	15.8662512	25.1136554	2.3035E-49	5.46942507	3.99E-05	0.66251074
SPRR1B	2.72837301	115.805939	1.60492571	5.83668935	4.7748E-58	5.40752453	1.21E-06	1.86264377
NCCRP1	0.07449807	3.01475212	0.01545279	0.19442359	3.9085E-19	5.33869245	0.00140901	3.65326388
SPRR2A	3.62753963	139.666739	0	0.30180277	2.33E-116	5.2668533	0.00240218	1
C2orf54	0.2468981	8.6586954	0.83035842	2.23137969	1.0332E-58	5.13216203	7.16E-05	1.42612991
SPRR3	0.53138813	17.7802132	1.74494126	13.8238232	1.4612E-47	5.0643628	6.33E-15	2.9859063
UPK2	1.86967014	50.2399054	2.25294057	6.26206135	5.3891E-91	4.74797808	9.50E-06	1.47482838
STEAP4	0.77969704	20.2711045	5.05232723	12.3951678	1.9688E-70	4.70036724	1.65E-09	1.29475781
FUT3	1.12924636	29.2596135	0.45701319	4.456802	5.1102E-45	4.69547854	2.76E-27	3.28570115
MARVELD3	0.54227125	11.4386299	3.11715533	5.06109169	3.4183E-49	4.39875576	0.0012764	0.69921856
EYA1	0.02679477	0.54233035	0.00375556	0.08156446	4.0789E-21	4.33914874	9.82E-05	4.44083961
DSG3	0.00584368	0.10229717	0.49822638	1.55565135	0.00047824	4.12974526	3.80E-10	1.64264545
IL1R2	0.05479013	0.89132613	0.13884903	1.37831826	4.7663E-10	4.02396544	4.32E-08	3.31132
KRT6B	0.21411665	3.48319692	0.01400751	0.13412056	9.4963E-30	4.02394317	0.00426314	3.25925804
KCP	0.17241481	2.65999224	0.94301356	3.44217572	1.4515E-50	3.94746641	2.11E-10	1.86797033
RNF223	0.32790781	4.98344296	5.38705732	9.78890314	7.1209E-31	3.92578066	0.0005649	0.8616498
TMEM125	0.86077116	12.6985318	11.9698727	18.192851	2.5837E-34	3.88288815	0.0005134	0.60396383
EPHA1	0.56922429	8.38433103	2.69081412	5.4598046	1.2043E-63	3.88062655	4.39E-08	1.02080658
TMEM30B	2.54073043	37.2536184	42.4779307	56.1889572	7.1363E-60	3.87406534	0.0111874	0.40357314
C19orf77	0.06726726	0.95787179	0.329241	0.93480322	6.3045E-08	3.83185618	0.00394886	1.50551868
IVL	0.4295109	6.08247145	0.09548324	1.5151928	6.3656E-26	3.82389099	4.18E-15	3.98811001
LY6D	16.7945848	218.869482	1.00331901	3.0234767	1.9062E-47	3.70400277	3.91E-06	1.59142807
KRT6A	4.73891675	60.8080177	0.13172684	5.53789097	1.6646E-88	3.68163234	3.00E-56	5.39371538
ALPPL2	0.58823119	7.35038932	0.00611234	0.16835835	1.1749E-17	3.64336547	5.38E-05	4.78366641
SYK	0.00539266	0.06731489	0.11176749	0.72143088	0.0041714	3.64185725	1.69E-11	2.69036053
CYBB	0.01038896	0.12770783	0.02273587	0.12385095	2.4111E-06	3.61972354	0.00031004	2.44556264
SULT2B1	0.20227054	2.43415518	0.5902086	1.91064968	1.6338E-11	3.58906306	3.58E-06	1.69476643
LRRN4	0.07946475	0.9200604	2.40959469	13.5302312	1.2682E-11	3.53334171	5.82E-21	2.48932409
FLJ43826	0.00800011	0.088436	0	0.09242612	0.00740743	3.46654147	0.00049207	1
FGFR2	0.28648171	3.16302867	0.4994975	0.99263436	7.5918E-36	3.46479168	0.00072615	0.99078493
C6orf15	11.1735379	116.847073	14.6549	45.5639661	5.6112E-83	3.38646363	6.20E-17	1.63651021
ZSCAN12P1	0.29624235	3.07559537	4.04979192	7.76174849	2.2984E-39	3.37601592	5.11E-05	0.9385339
ZBED2	5.92924706	59.4406592	2.83389833	15.9062399	1.6321E-49	3.3255293	1.35E-26	2.48873293
SYT8	1.3341817	13.1487005	0.51347079	1.05410402	1.1999E-47	3.30089316	0.00512818	1.03766312
TTYH1	0.05179366	0.50799888	0.05190079	0.19904633	6.1077E-06	3.29397781	0.00684706	1.9392759
LCP1	0.56879549	5.44031412	1.55762097	4.00352861	8.468E-45	3.25770802	0.00046958	1.3619279
LOC100130(	0.5237253	4.50862847	2.70062241	8.10721599	6.885E-22	3.10580643	1.36E-11	1.58591464
ANKRD2	0.06756903	0.56952217	0.70826481	2.36825971	2.4888E-05	3.07531799	3.01E-06	1.74146653
PAPL	2.03903969	16.7949761	0.63099488	1.12840944	6.0548E-30	3.04206798	0.00263455	0.83859043
SEMA4A	0.39216092	3.21317175	2.08637786	4.71366817	1.3219E-22	3.03448044	2.67E-09	1.17584973
C9orf152	0.10545648	0.86159697	3.19820531	6.02369944	8.4764E-09	3.0303654	3.36E-05	0.91338723
IL1RN	17.5711984	141.858425	0.16646585	2.39470188	5.2046E-32	3.01316734	5.12E-11	3.8465479
TRIM29	0.99141773	7.9261949	3.50472963	9.29573586	4.1396E-11	2.99906347	1.33E-09	1.40726592
PROM2	2.44194912	19.3798395	14.0430431	22.5835109	1.2517E-55	2.98845157	4.88E-06	0.68541419
MIR205HG	2.54673765	20.15686	0.27275844	1.07285409	6.8675E-27	2.98454866	0.00332526	1.97575816
ARHGAP40	1.82600868	14.1316386	0.07550032	1.52863234	3.0689E-30	2.95216323	2.78E-13	4.33961487
ECEL1P2	0.10537634	0.81187628	0	0.5398943	0.00011415	2.9457089	3.11E-06	1
IL36RN	0.13322062	1.01474972	0	0.15095397	7.4737E-09	2.92923465	2.41E-06	1
LOC100128:	4.74327033	35.6570114	8.38058237	16.0702298	2.9707E-59	2.91023179	4.15E-08	0.93926815
TSPAN18	2.32197566	17.2766635	1.92918146	5.07837834	1.5451E-50	2.89539988	9.91E-11	1.39637903
GSDMC	0.60005249	4.29703606	10.3335499	14.6068053	1.681E-33	2.84018128	0.00256152	0.49930473
EPN3	2.36539565	16.8619285	6.33840662	9.28106227	1.6252E-65	2.83361612	0.00744313	0.55016972
TFCP2L1	0.13616168	0.95860914	0.317716	1.68715831	1.4809E-19	2.81562192	7.56E-12	2.40878572
GRHL2	4.70098997	33.0156179	13.7395028	28.5523653	6.2138E-73	2.81211214	2.86E-10	1.05528047
SLC44A3	0.11266494	0.77777645	1.92139237	3.57408497	8.7216E-11	2.78731688	0.00021854	0.89542177
MAL	0.03569303	0.24637404	0	0.47238161	0.00584292	2.78713575	5.19E-05	1
TH	0.10066611	0.67940797	0.15693829	0.92238556	1.179E-05	2.75470006	7.78E-09	2.55517254
CCDC64	0.46986322	3.15607062	0.94024425	2.41187047	1.1716E-27	2.74781674	7.91E-08	1.35904494
IL23A	2.06790535	13.6765755	2.09279599	3.60310278	5.2581E-14	2.72546498	0.00428623	0.78380812
MYO1D	6.09600316	40.0067436	8.45959109	17.1552318	8.1401E-54	2.71430765	6.75E-11	1.01998878
SOX15	2.84643047	18.6528505	5.15093671	12.9792078	8.4427E-37	2.71217035	8.19E-07	1.33329561
OVOL2	1.36683901	8.82567332	5.58735052	8.98775985	2.0139E-28	2.69086302	0.00156021	0.68579725
AQP3	0.1507088	0.97164402	3.10563102	8.70633976	1.9759E-05	2.6886642	2.49E-12	1.48717989
C4orf19	0.59461285	3.7530839	0.08260245	0.80702687	3.8325E-29	2.658054	5.47E-17	3.28836018
SLC6A14	1.85768098	11.2837547	25.8374879	43.3835291	2.0024E-18	2.60267254	0.00011335	0.74768161
CORO1A	2.95804243	17.7558796	0.68936895	1.88047524	5.4454E-47	2.58558219	2.22E-06	1.44774908
CCL28	1.90051055	11.3890594	1.66407345	20.1984202	9.0002E-15	2.58318967	4.84E-38	3.60145144
CST6	14.5899458	86.5455027	3.28496662	18.6015636	1.4439E-41	2.56848433	6.93E-12	2.50147328
CDRT1	0.07710181	0.45216933	0	0.11348942	5.679E-08	2.55202644	4.28E-05	1
AIM1L	1.1289495	6.6007962	4.28843009	6.1411766	2.948E-37	2.54765911	0.00209104	0.51806549
COBL	0.54143328	3.15882442	4.80426381	9.32838281	5.0533E-31	2.54453228	4.55E-10	0.95731162
SERPINB2	17.738483	103.128498	0.00790471	0.10358924	1.9538E-44	2.53948851	0.00771142	3.71201702
GALNT3	7.85814458	45.1952946	35.0844724	53.7064257	4.3502E-42	2.52391196	0.00030732	0.61426204
SCEL	11.5923789	66.4393609	4.56266493	7.66058119	2.099E-48	2.51886155	0.00103713	0.74757714
USP2	0.15756629	0.89681559	0.17856862	0.37766005	5.268E-13	2.50885244	0.00378409	1.08060957
LRRC26	0.10662002	0.60454927	0.09176804	0.91184837	1.5964E-05	2.5033816	0.00054541	3.31273017
GPR115	0.61996581	3.50922518	8.54186374	14.0847695	1.8105E-11	2.50089196	8.43E-05	0.72151316
SCN5A	0.17343516	0.98081255	0.03648312	0.11047385	7.448E-13	2.49958108	0.0028136	1.59840396
CHST4	3.7398759	20.9591663	0.64822508	1.38877812	1.6124E-32	2.48651903	0.00307097	1.09924937
SYT16	0.10693628	0.58996144	0.01066048	0.32949422	9.0091E-07	2.46386928	2.66E-07	4.94990922
FST	4.16847679	22.7939006	0.15179998	1.93723864	7.5531E-43	2.45105562	5.49E-14	3.67375818
VEPH1	0.42727267	2.24608077	0.43389563	2.64118591	1.8446E-24	2.39418087	1.20E-23	2.60576588
TBC1D30	0.42244031	2.14588929	1.50275922	2.65142109	1.9801E-27	2.34475622	7.15E-06	0.81915194
ALPP	2.6380685	13.2758061	0.01063424	0.34579831	2.5296E-20	2.33124553	1.99E-05	5.02314239
HSD11B2	0.36659825	1.84153795	0.86175126	1.74454999	4.793E-11	2.32863934	0.002012	1.01751153
LINC00514	0.44759786	2.24569021	0.97715273	1.70489318	4.9252E-11	2.32688389	0.00316916	0.80302537
GIPC2	0.10310236	0.51501221	0.29316943	0.8838004	3.2172E-08	2.32052923	0.00044694	1.59198589
SERPINF2	0.26779871	1.3303334	2.00402453	3.19983843	5.6628E-10	2.31256692	0.00311459	0.6750989
MOB3B	0.15027394	0.74592727	3.6510395	6.7984514	3.7172E-10	2.3114401	0.00059857	0.89689888
C1orf116	19.6523771	95.9056285	42.6518964	58.3166291	8.1505E-62	2.28691166	0.00415814	0.45129744
GRHL1	0.64205874	3.10767907	0.95206428	2.30625909	7.1665E-15	2.27506033	2.29E-07	1.27642371
SLC39A4	2.85692504	13.1693633	9.35712551	14.5101055	1.4262E-35	2.20465051	8.05E-05	0.63292069
KRT81	57.6761155	265.288648	56.4170361	270.037917	7.9303E-34	2.20151703	5.93E-10	2.25895922
RASAL1	0.40966837	1.8841325	0.81797627	1.84060246	1.7508E-13	2.20137201	3.30E-06	1.17004716
ADAMTS9	0.25861673	1.18846342	0.59983152	1.25311154	8.1711E-12	2.20020996	2.23E-06	1.06288559
MYB	0.05267623	0.23868802	0.04348872	0.46846346	3.6655E-05	2.17990221	9.84E-10	3.42922332
